# Transition Metal‐Free Formal C─H/C─H Coupling of Arylacetamides and Sulfoxides: An Interrupted Pummerer/[2,3]‐sigmatropic Rearrangement Sequence

**DOI:** 10.1002/anie.202511703

**Published:** 2025-07-29

**Authors:** Shibo Zhang, Ying Xin Lou, Allya Larroza, Ben W. Joynson, Ciro Romano, David J. Procter

**Affiliations:** ^1^ Department of Chemistry University of Manchester Oxford Road Manchester M13 9PL UK; ^2^ Laboratório de Síntese Orgânica Limpa‐LASOL‐CCQFA Universidade Federal de Pelotas Brazil

**Keywords:** Arylacetamides, Cross‐coupling, Pummerer, Sigmatropic rearrangement, Sulfoxide

## Abstract

Arylacetamides are important structural motifs found in many bioactive compound classes. Given their significance, the selective decoration of arylacetamides by transition metal‐catalyzed C─H activation has attracted considerable attention. Here, we report a two‐step transition metal‐free formal C─H/C─H coupling of arylacetamides with sulfoxides that proceeds via the formation and rearrangement of little‐known α‐amido sulfonium salts; the amide motif “catches” the sulfoxide partner prior to its delivery to the *ortho*‐position of the aromatic ring by rearrangement of a sulfur ylide intermediate. The approach delivers alkylated arylacetamides bearing sulfide functional handles that can be exploited in downstream manipulations. This protocol has allowed the synthesis of a dopamine D1 receptor allosteric modulator without recourse to the use of transition metals.

## Graphical abstract

A two‐step formal C─H/C─H coupling of arylacetamides with sulfoxides proceeds via the formation and rearrangement of little‐known α‐amido sulfonium salts and provides an alternative to transition metal‐catalyzed C─H activation. The protocol is showcased in a short synthesis of a dopamine D1 receptor allosteric modulator.

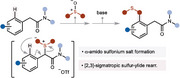

Arylacetamides are important motifs commonly found in drugs and agrochemicals (Scheme 1a).^[^ ^1^, ^2^, ^3^, ^4^, ^5^, ^6^ ^]^ Given their biological significance and interest in the use of amide motifs to direct transition metal‐catalyzed C─H activation,^[^ ^7^ ^]^ the selective decoration of arylacetamides by C─H activation has attracted considerable attention (Scheme 1b).^[^ ^8^, ^9^, ^10^, ^11^, ^12^, ^13^, ^14^, ^15^, ^16^, ^17^, ^18^, ^19^, ^20^, ^21^, ^22^, ^23^, ^24^ ^]^ Pd‐ and Ru‐transition metal catalysts are typically employed at elevated temperatures to achieve C─H arylation,^[^ ^8^, ^9^, ^10^, ^11^, ^12^ ^]^ alkenylation,^[^ ^13^, ^14^, ^15^, ^16^ ^]^ alkynylation,^[^ ^17^ ^]^ acylation,^[^ ^18^ ^]^ and, less‐commonly, C─H alkylation,^[^ ^19^ ^]^ in addition to forming C─X bonds^[^ ^20^, ^21^ ^]^ and related methods for *meta* C─H functionalization of arylacetamides.^[^ ^22^, ^23^, ^24^ ^]^

**Scheme 1:**
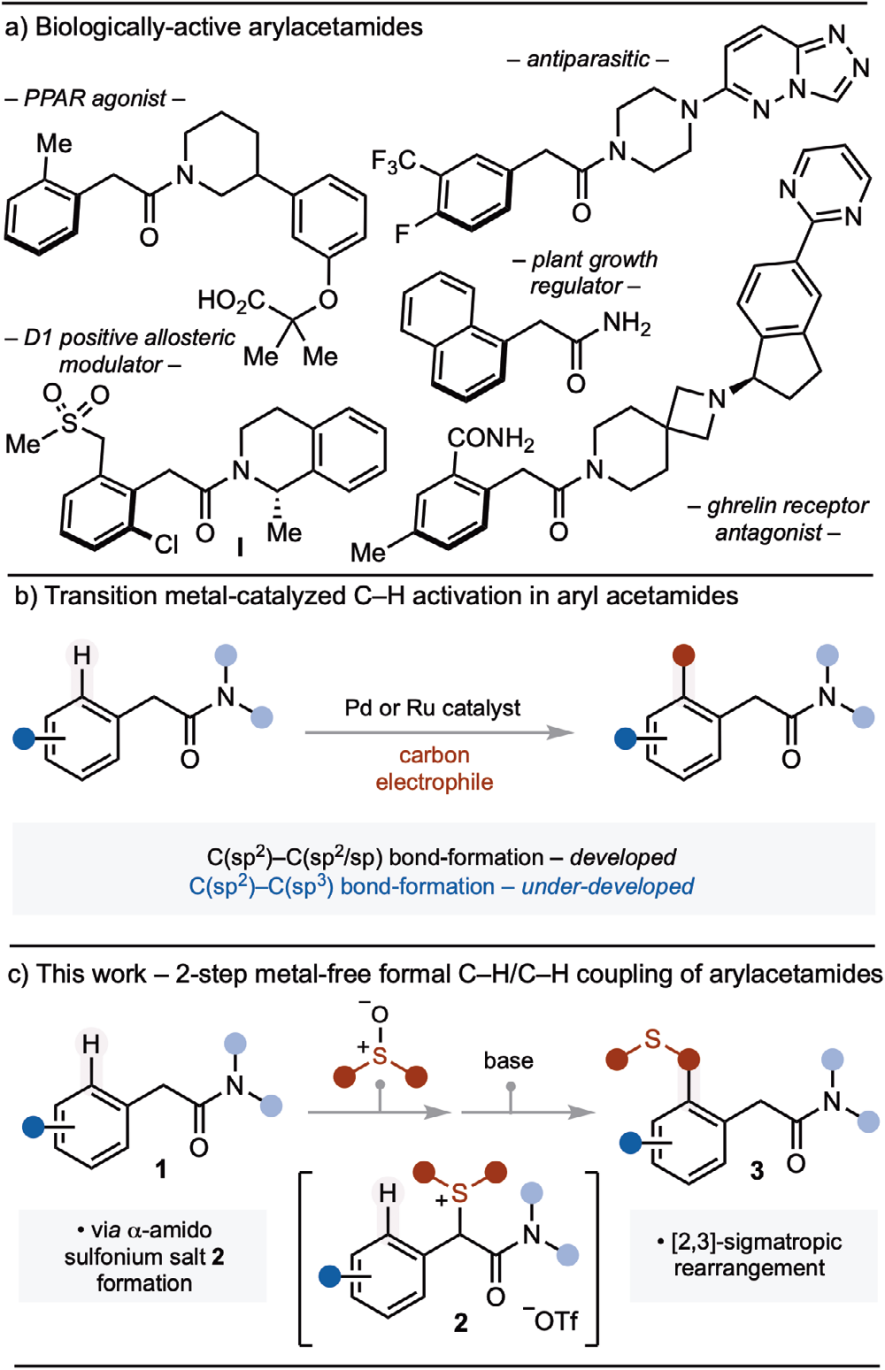
a) Arylacetamides are important motifs in bioactive molecules. b) Established Pd and Ru‐catalyzed C─H activation strategies for the decoration of arylacetamides. c) α‐Amido sulfonium salt formation and [2,3]‐sigmatropic rearrangement constitute a formal transition metal‐free C─H/C─H coupling of arylacetamides and sulfoxides. Tf = 1,1,1‐trifluoromethanesulfonyl.

The development of complementary approaches that allow similar transformations to be achieved without recourse to the use of metals, by exploiting very different reactivity, is an important field of synthesis.^[^ ^25^, ^26^ ^]^ In particular, the harnessing of sulfoxide chemistry has emerged as a powerful approach when attempting to mimic transformations enabled by transition metal‐catalyzed C─H activation.^[^ ^27^, ^28^, ^29^, ^30^, ^31^, ^32^, ^33^, ^34^, ^35^, ^36^, ^37^, ^38^ ^]^ Such processes often involve the in situ formation of sulfonium salt intermediates by addition of one coupling partner to a sulfoxide moiety present on the other fragment to be attached; subsequent [3,3]‐sigmatropic rearrangement triggers C─C bond formation, formally at the expense of a C─H bond. This approach has been used to achieve the formal C─H alkylation,^[^ ^39^, ^40^, ^41^, ^42^, ^43^ ^]^ allylation,^[^ ^44^, ^45^, ^46^, ^47^, ^48^ ^]^ propargylation,^[^ ^49^, ^50^, ^51^, ^52^ ^]^ and arylation^[^ ^53^, ^54^, ^55^, ^56^, ^57^, ^58^, ^59^, ^60^, ^61^, ^62^, ^63^ ^]^ of (hetero)aromatic systems.

In a single, isolated report, Movassaghi described an α‐sulfenylation of amides that proceeds via the formation and in situ demethylation of α‐amido sulfonium salts.^[^ ^64^ ^]^ Further study of the properties of the sulfonium salts was not reported. We have recently exploited α‐amido sulfonium salts, readily prepared from amides, as bench‐stable and user‐friendly precursors of α‐amidyl radicals in metal‐free photocatalytic C(sp^3^)─C(sp^2^) and C(sp^3^)─C(sp^3^) bond‐forming cross‐coupling reactions that realize the formal α‐alkylation, α‐alkenylation, and α‐arylation of amides.^[^ ^65^ ^]^

Herein, we report the development of a formal C─H/C─H coupling of arylacetamides **1** with sulfoxides that proceeds via the formation and rearrangement of little‐known α‐amido sulfonium salts **2**; the amide motif “catches” the sulfoxide partner prior to its delivery to the *ortho*‐position of the aromatic ring by rearrangement of sulfur‐ylides.^[^ ^32^, ^66^, ^67^, ^68^, ^69^, ^70^, ^71^, ^72^, ^73^, ^74^, ^75^, ^76^, ^77^, ^78^, ^79^, ^80^, ^81^ ^]^ The approach delivers alkylated arylacetamides **3** bearing benzylic sulfide functional handles that can be exploited in downstream manipulations (Scheme 1c).

Using our adaptation of the conditions of Movassaghi and coworkers,^[^ ^64^, ^65^ ^]^ α‐amido sulfonium salt **2a** was prepared in 74% yield and isolated as a bench‐stable solid either by trituration from hexane followed by recrystallization from ethyl acetate or by column chromatography. We next studied the deprotonation of **2a** to form the necessary sulfur ylide for [2,3]‐sigmatropic rearrangement^[^ ^66^, ^67^, ^68^, ^69^, ^70^, ^71^, ^72^, ^73^, ^74^, ^75^, ^76^, ^77^, ^78^, ^79^, ^80^, ^81^ ^]^ and formation of the formal C─H/C─H alkylation product **3a** (Table 1). Of note, related rearrangements involve sulfur‐ylides derived from esters, formed by transition‐metal catalyzed^[^ ^73^, ^75^, ^76^, ^77^, ^78^ ^]^ or photochemical^[^ ^81^ ^]^ carbene addition to sulfides using aryl diazo acetates as carbene precursors.^[^ ^67^, ^68^, ^69^, ^70^, ^71^, ^80^, ^81^ ^]^ This route to ylides can limit structural diversity in the coupling partners and necessitates the handling of sensitive diazo species.

**Table 1:** Optimization of the rearrangement of α‐amido sulfonium salt **2a** to **3a**.

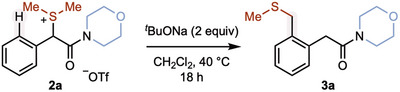			
Entry^a)^	Deviations	Conversion (%)	Yield (%)^b)^
1	None	>99	>99
2	KOH instead of *^t^* BuONa	>99	98
3	K_2_CO_3_ instead of *^t^* BuONa	55	25
4	TMG instead of *^t^* BuONa	>99	90
5	DBU instead of *^t^* BuONa	>99	12
6	NEt_3_ instead of *^t^* BuONa	>99	<5
7	MeCN instead of CH_2_Cl_2_	>99	97
8	2 Equiv *^t^* BuOH in CH_2_Cl_2_	60%	38%
9	Room temperature instead of 40 °C	<5	N.D.

^a)^ Reactions run on 0.1 mmol scale.

^b)^ Calculated by ^1^H NMR analysis of the crude product mixture using CH_3_NO_2_ as an internal standard. TMG = 1,1,3,3‐tetramethylguanidine. DBU = 1,8‐diazabicyclo[5.4.0]undec‐7‐ene.

Exposure of **2a** in CH_2_Cl_2_ to ^*t*^BuONa at 40 °C gave **3a** in near quantitative yield (entry 1). The use of KOH gave similar results (entry 2); however, the use of weaker inorganic bases was less effective (entry 3). Alternative organic bases could be employed (entries 4–6), with TMG being the most effective (entry 4). MeCN solvent could also be used (entry 7); however, the addition of alcohol solvent was less effective (entry 8). Finally, heating above ambient temperature was shown to be important; reaction at room temperature gave little conversion (entry 9). In all cases, products of competing 1,2‐rearrangement were not observed.

With optimal conditions for the rearrangement step in hand, we moved on to evaluate the scope of the 2‐step process. Scheme 2 sets out the isolated yields for the formation of sulfonium salts **2**—from arylacetamides **1**—and their subsequent rearrangement to give decorated arylacetamides **3**. Variation of the aryl ring in the arylacetamide substrates **1** showed the compatibility of the process with electron‐donating alkyl (**3b**‐**3e**) and alkoxy (**3f** and **3g**) substituents and electron‐withdrawing halide (**3g**‐**3i**) and trifluoromethyl (**3j**) groups. Finally, naphthyl acetamide **1k** was converted to **3k** in moderate yield over 2 steps. Varying the amino motif in the arylacetamide substrates **1** illustrated compatibility with piperazinyl (**3l**), pyrrolidinyl (**3m**), piperidinyl (**3n**), substituted morpholinyl (**3o**), dimethyl amino (**3p**), and methoxy methyl amino (**3q**) groups. Lower yields were obtained working with Weinreb amide motif (**2q** and **3q**) presumably due to the increased electrophilicity of the amide carbonyl leading to side reactions. In addition, arylacetamides derived from the drug molecules maprotiline and fluoxetine underwent 2‐step transformation to **3r** and **3s**, respectively. Finally, variation of the sulfoxide coupling partner allowed a range of sulfide units to be installed. For example, symmetrical acyclic sulfoxides, *d*_6_‐DMSO, and diethyl sulfoxide gave labeled (**3t**) and branched (**3u**) alkylation products. Unsymmetrical acyclic sulfoxides, ethyl methyl sulfoxide and phenyl methyl sulfoxide could also be employed to give **3v** and **3w**, respectively. With ethyl methyl sulfoxide, rearrangement took place selectively to give the product of arylation at the less‐hindered position α to sulfur (**3v**). Working with phenyl methyl sulfoxide, sulfonium salt **2w** proved difficult to purify so the crude product was taken into the rearrangement step to give **3w** in an overall 45% isolated yield. The 2‐step process was also compatible with cyclic sulfoxide partners, with tetrahydropthiophene (**3x**), tetrahydrothiopyran (**3y**), 1,4‐oxathiane (**3z**), and cyano‐substituted tetrahydrothiopyran (**3aa**) sulfoxides undergoing coupling in moderate overall yield (Scheme 2).

**Scheme 2:**
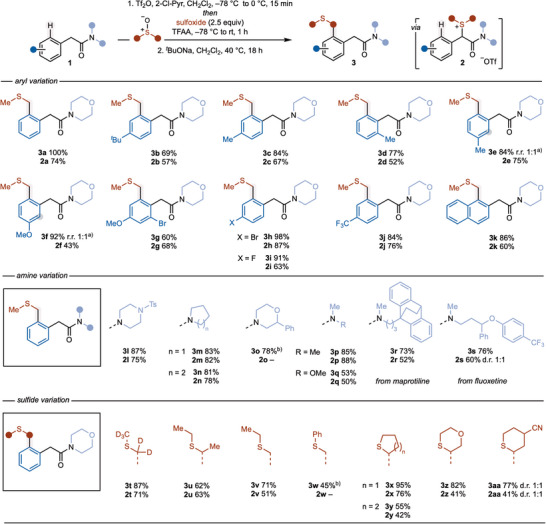
Scope of α‐amido sulfonium salt formation and rearrangement in arylacetamides. Reaction conditions for α‐amido sulfonium salt formation: 2‐chloropyridine (2.0 equiv), sulfoxide (2.5 equiv), Tf_2_O (1.1 equiv), TFAA (1.0 equiv), CH_2_Cl_2_ [0.25 M], −78 °C, 2 h. Reaction conditions for sulfonium salt rearrangement: *^t^* BuONa (2.0 equiv), CH_2_Cl_2_ [0.10 M], 40 °C, 18 h. ^a)^Shaded circles highlight the position of substitution in the other regioisomer. DMSO: dimethylsulfoxide; Pyr: pyridine; Tf: 1,1,1‐trifluoromethylsulfonyl; TFAA: trifluoroacetic anhydride. ^b)^Overall yield for 2 steps—**2o** and **2w—**were not isolated and were rearranged directly to **3o** and **3w**, respectively.

The process can be carried out on a 10 mmol scale—**2a** was formed in 70% yield and underwent smooth rearrangement to give **3a** in 93% yield (2.46 g)—and the products are rich in potential for further manipulation (Scheme 3a). For example, reduction or α‐alkylation of the directing amide group gave **4** and **5** in 74% and 93% yield, respectively. Selective oxidation of the alkylsulfanyl unit in **3a** gave sulfoxide **6** in 90%. Finally, re‐exposure of **3a** to the 2‐step procedure, using tetrahydropthiophene sulfoxide as the coupling partner, gave the unsymmetrical bis‐sulfide **7** in 51% overall yield.

**Scheme 3:**
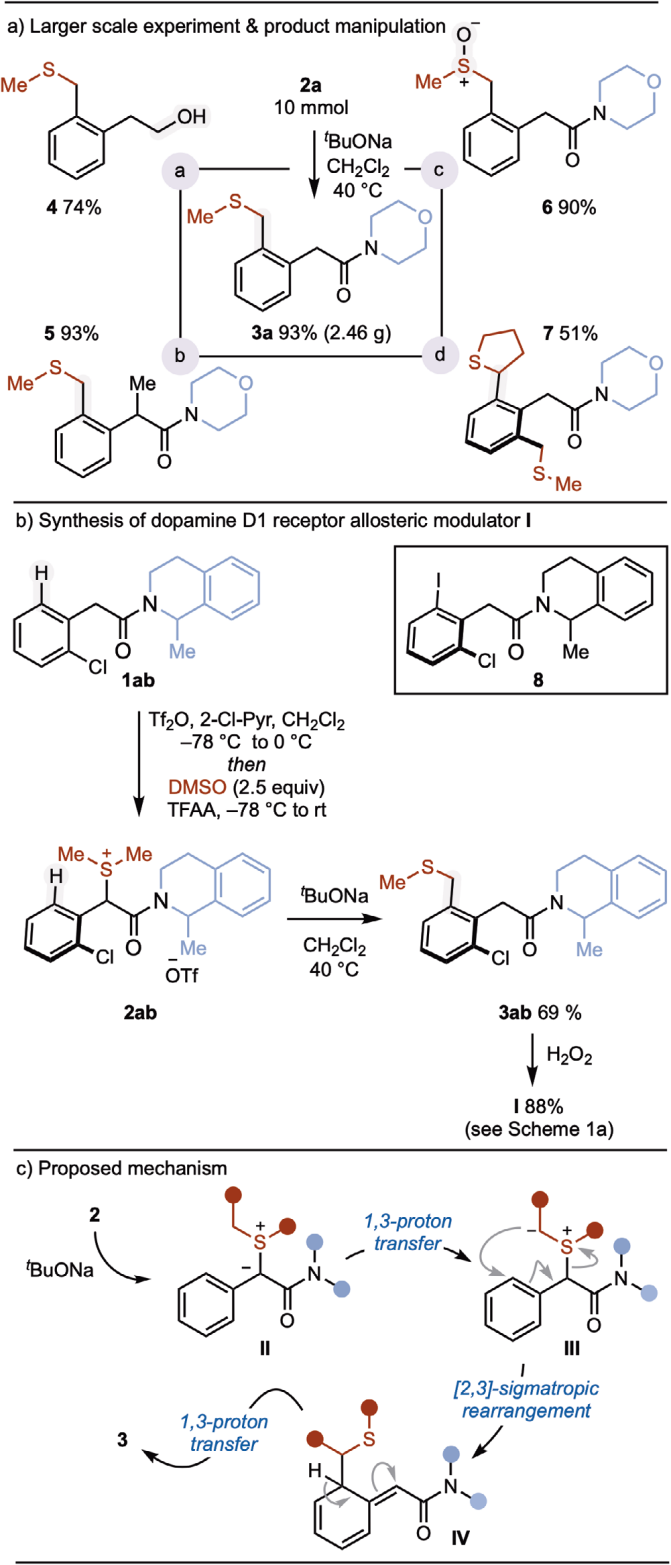
a) Larger‐scale sulfonium salt formation/rearrangement and product manipulation. Reaction conditions: (a) LiAlH_4_, THF, 0 °C to rt; (b) *^n^* BuLi, MeI, THF; (c) AcOH, H_2_O_2_, 0 °C; (d) see Scheme 2. b) Synthesis of bioactive aryl acetamide **I**. DMSO: Dimethylsulfoxide; Tf: 1,1,1‐trifluoromethylsulfonyl; TFAA: trifluoroacetic anhydride. c) Proposed reaction mechanism for the base‐mediated [2,3]‐sigmatropic rearrangement of α‐amido sulfonium salts.

Finally, the 2‐step formal C─H/C─H coupling of arylacetamides and sulfoxides has been used to prepare *rac*‐**I** (Scheme 1a)—a dopamine D1 receptor allosteric modulator,^[^ ^4^ ^]^ originally prepared in five steps and low yield from aryl iodide **8**, with the first step being an inefficient Pd‐catalyzed methoxycarbonylation.^[^ ^4^ ^]^ Readily prepared arylacetamide **1ab** underwent sulfonium salt formation to give **2ab**, which was not isolated and directly engaged in the rearrangement to give **3ab** (69% over two steps). Straightforward oxidation of the alkylsulfanyl unit in **3ab** to the alkylsulfonyl motif gave **I** (Scheme 3b).

Based on previous reports of related [2,3]‐sigmatropic rearrangements,^[^ ^66^, ^67^, ^68^, ^69^, ^70^, ^71^, ^72^, ^73^, ^74^, ^75^, ^76^, ^77^, ^78^, ^79^, ^80^, ^81^ ^]^ we propose a mechanism involving deprotonation of the α‐amido sulfonium salt **2** by *^t^* BuONa to generate sulfur‐ylide intermediate **II**. After 1,3‐proton transfer to afford sulfur‐ylide **III**, [2,3]‐sigmatropic rearrangement involving the aromatic ring of the arylacetamide moiety delivers **IV**. A second 1,3‐proton transfer—likely mediated by the base—delivers the rearomatized product **3**.

In conclusion, a two‐step transition metal‐free formal C─H/C─H coupling of arylacetamides with sulfoxides proceeds via the formation and rearrangement of little‐known α‐amido sulfonium salts; the amide motif “catches” the sulfoxide partner in an interrupted Pummerer process prior to its delivery to the *ortho*‐position of the aromatic ring by [2,3]‐sigmatropic rearrangement of a sulfur ylide intermediate. The approach delivers *ortho*‐alkylated arylacetamides bearing benzylic sulfide functional handles that can be exploited in downstream manipulations. The short synthesis of a dopamine D1 receptor allosteric modulator has been used to illustrate the utility of the approach.

## Conflict of Interests

The authors declare no conflict of interest.

## Supporting information



Supporting Information

## Data Availability

The data that support the findings of this study are available in the Supporting Information of this article.

## References

[1] F. Goetze , E. Kehrli , J. Marcus Eugene , P. Ricketts , C. Taube , (Pfizer Products Inc., New York City, US), WO2005115369 A2, 2005.

[2] T. J. Schubert , E. Oboh , H. Peek , E. Philo , J. E. Teixeira , E. E. Stebbins , P. Miller , J. Oliva , F. M. Sverdrup , D. W. Griggs , C. D. Huston , M. J. Meyers , J. Med. Chem. 2023, 66, 7834–7848. 37267631 10.1021/acs.jmedchem.3c00110 PMC11103792

[3] A. Y. Kocaman , B. Güven , Cytotechnology 2016, 68, 947–956. 25690333 10.1007/s10616-015-9847-z PMC4960144

[4] A. Valade , E. Jnoff , A. Ates , P. Burssens , D. Skolc , (UCB Biopharma SPRL, Brussels, BE), WO2016055479 A1, 2016.

[5] K. Bhattacharya , O. Cameron , P. Fernando , W. Kung , T. Londregan , F. McClure , M. Simila , (Pfizer Inc., New York City, US), WO2011114271 A1, 2011.

[6] P. R. Halfpenny , D. C. Horwell , J. Hughes , J. C. Hunter , D. C. Rees , J. Med. Chem. 1990, 33, 286–291. 2153208 10.1021/jm00163a047

[7] Q. Zheng , C.‐F. Liu , J. Chen , G.‐W. Rao , Adv. Synth. Catal. 2020, 362, 1406–1446.

[8] C. S. Yeung , X. Zhao , N. Borduas , V. M. Dong , Chem. Sci. 2010, 1, 331–336.

[9] Y. Jaiswal , Y. Kumar , R. Thakur , J. Pal , R. Subramanian , A. Kumar , J. Org. Chem. 2016, 81, 12499–12505. 27978736 10.1021/acs.joc.6b02353

[10] Y. Liu , B. Huang , X. Cao , J.‐P. Wan , ChemCatChem 2016, 8, 1470–1473.

[11] Z. Tu , Y. Du , X. Cao , Y. Liu , Adv. Synth. Catal. 2019, 361, 4989–4997.

[12] A. Dutta , M. Jeganmohan , J. Org. Chem. 2022, 87, 13154–13167. 36094897 10.1021/acs.joc.2c01625

[13] A. Deb , S. Bag , R. Kancherla , D. Maiti , J. Am. Chem. Soc. 2014, 136, 13602–13605. 25188679 10.1021/ja5082734

[14] Q. Bu , T. Rogge , V. Kotek , L. Ackermann , Angew. Chem. Int. Ed. 2018, 57, 765–768. 10.1002/anie.201711108 29141119

[15] Y. Jaiswal , Y. Kumar , A. Kumar , J. Org. Chem. 2018, 83, 1223–1231. 29276827 10.1021/acs.joc.7b02618

[16] S. Mishra , A. Kumar , Eur. J. Org. Chem. 2022, e202201129.

[17] X. Ye , C. Xu , L. Wojtas , N. G. Akhmedov , H. Chen , X. Shi , Org. Lett. 2016, 18, 2970–2973. 27267908 10.1021/acs.orglett.6b01319

[18] J. Park , M. Kim , S. Sharma , E. Park , A. Kim , S. H. Lee , J. H. Kwak , Y. H. Jung , I. S. Kim , Chem. Commun. 2013, 49, 1654–1656. 10.1039/c3cc38764j 23334232

[19] R. Sivasakthikumaran , S. Jambu , M. Jeganmohan , J. Org. Chem. 2019, 84, 3977–3989. 30821149 10.1021/acs.joc.8b03257

[20] Y. Jaiswal , Y. Kumar , A. Kumar , Org. Biomol. Chem. 2019, 17, 6809–6820. 31246220 10.1039/c9ob01082c

[21] M. V. Barysevich , M. V. Laktsevich‐Iskryk , A. V. Krech , V. N. Zhabinskii , V. A. Khripach , A. L. Hurski , Eur. J. Org. Chem. 2020, 937–943.

[22] X.‐C Wang , W. Gong , L.‐Z. Fang , R.‐Y. Zhu , S. Li , K. M. Engle , J.‐Q. Yu , Nature 2015, 519, 334–338. 25754328 10.1038/nature14214 PMC4368492

[23] P.‐X. Shen , X.‐C. Wang , P. Wang , R.‐Y. Zhu , J.‐Q. Yu , J. Am. Chem. Soc. 2015, 137, 11574–11577. 26313012 10.1021/jacs.5b08914 PMC4573956

[24] Z. Jin , L. Chu , Y.‐Q. Chen , J.‐Q. Yu , Org. Lett. 2018, 20, 425–428. 29303593 10.1021/acs.orglett.7b03336

[25] J. D. Lasso , D. J. Castillo‐Pazos , C.‐J. Li , Chem. Soc. Rev. 2021, 50, 10955–10982. 34382989 10.1039/d1cs00380a

[26] S. Roy , S. Panja , S. R. Sahoo , S. Chatterjee , D. Maiti , Chem. Soc. Rev. 2023, 52, 2391–2479. 36924227 10.1039/d0cs01466d

[27] L. H. S. Smith , S. C. Coote , H. F. Sneddon , D. J. Procter , Angew. Chem. Int. Ed. 2010, 49, 5832–5844. 10.1002/anie.201000517 20583014

[28] A. P. Pulis , D. J. Procter , Angew. Chem. Int. Ed. 2016, 55, 9842–9860. 10.1002/anie.201601540 27409984

[29] H. Yorimitsu , Chem. Rec. 2017, 17, 1156–1167. 28488753 10.1002/tcr.201700017

[30] T. Yanagi , K. Nogi , H. Yorimitsu , Tetrahedron Lett. 2018, 59, 2951–2959.

[31] Z. He , A. P. Pulis , G. J. P. Perry , D. J. Procter , Phosphorus Sulfur Silicon Relat. Elem. 2019, 194, 669–677.

[32] D. Kaiser , I. Klose , R. Oost , J. Neuhaus , N. Maulide , Chem. Rev. 2019, 119, 8701–8780. 31243998 10.1021/acs.chemrev.9b00111 PMC6661881

[33] V. Trudel , C.‐H. Tien , A. Trofimova , A. K. Yudin , Nat. Rev. Chem. 2021, 5, 604–623. 37118420 10.1038/s41570-021-00304-2

[34] G. J. P. Perry , H. Yorimitsu , ACS Sustainable Chem. Eng. 2022, 10, 2569–2586.

[35] H. Yorimitsu , G. J. P. Perry , Proc. Jpn. Acad. Ser. B 2022, 98, 190–205. 35400695 10.2183/pjab.98.012 PMC9071926

[36] S. Akai , N. Morita , K. Iio , Y. Nakamura , Y. Kita , Org. Lett. 2000, 2, 2279–2282. 10930263 10.1021/ol0001261

[37] S. Akai , N. Kawashita , H. Satoh , Y. Wada , K. Kakiguchi , I. Kuriwaki , Y. Kita , Org. Lett. 2004, 6, 3793–3796. 15469351 10.1021/ol0484310

[38] S. Akai , N. Kawashita , Y. Wada , H. Satoh , A. H. Alinejad , K. Kakiguchi , I. Kuriwaki , Y. Kita , Tetrahedron Lett. 2006, 47, 1881–1884.

[39] T. Kobatake , S. Yoshida , H. Yorimitsu , K. Oshima , Angew. Chem. Int. Ed. 2010, 49, 2340–2343. 10.1002/anie.200906774 20186899

[40] X. Huang , N. Maulide , J. Am. Chem. Soc. 2011, 133, 8510–8513. 21574566 10.1021/ja2031882

[41] L. Shang , Y. Chang , F. Luo , J.‐N. He , X. Huang , L. Zhang , L. Kong , K. Li , B. Peng , J. Am. Chem. Soc. 2017, 139, 4211–4217. 28245112 10.1021/jacs.7b00969

[42] L. Zhang , J.‐N. He , Y. Liang , M. Hu , L. Shang , X. Huang , L. Kong , Z.‐X. Wang , B. Peng , Angew. Chem. Int. Ed. 2019, 58, 5316–5320. 10.1002/anie.201900434 30810251

[43] M. Chen , Y. Liang , T. Dong , W. Liang , Y. Liu , Y. Zhang , X. Huang , L. Kong , Z.‐X. Wang , B. Peng , Angew. Chem. Int. Ed. 2021, 60, 2339–2345. 10.1002/anie.202010740 33017503

[44] S. Yoshida , H. Yorimitsu , K. Oshima , Org. Lett. 2009, 11, 2185–2188. 19371075 10.1021/ol9004883

[45] A. J. Eberhart , J. E. Imbriglio , D. J. Procter , Org. Lett. 2011, 13, 5882–5885. 21999481 10.1021/ol2025197

[46] A. J. Eberhart , C. Cicoira , D. J. Procter , Org. Lett. 2013, 15, 3994–3997. 23855635 10.1021/ol401786d PMC3819180

[47] M. Šiaučiulis , S. Sapmaz , A. P. Pulis , D. J. Procter , Chem. Sci. 2018, 9, 754–759. 29629145 10.1039/c7sc04723a PMC5870476

[48] J. Yan , A. P. Pulis , G. J. P. Perry , D. J. Procter , Angew. Chem. Int. Ed. 2019, 58, 15675–15679. 10.1002/anie.201908319 31479175

[49] A. J. Eberhart , D. J. Procter , Angew. Chem. Int. Ed. 2013, 52, 4008–4011. 10.1002/anie.201300223 23447131

[50] A. J. Eberhart , H. J. Shrives , E. Álvarez , A. Carrër , Y. Zhang , D. J. Procter , Chem. Eur. J. 2015, 21, 7428–7434. 25752800 10.1002/chem.201406424 PMC4524421

[51] A. J. Eberhart , H. Shrives , Y. Zhang , A. Carrër , A. V. S. Parry , D. J. Tate , M. L. Turner , D. J. Procter , Chem. Sci. 2016, 7, 1281–1285. 29910885 10.1039/c5sc03823e PMC5975836

[52] J. A. Fernández‐Salas , A. J. Eberhart , D. J. Procter , J. Am. Chem. Soc. 2016, 138, 790–793. 26745643 10.1021/jacs.5b12579

[53] T. Kobatake , D. Fujino , S. Yoshida , H. Yorimitsu , K. Oshima , J. Am. Chem. Soc. 2010, 132, 11838–11840. 20687603 10.1021/ja1030134

[54] B. Peng , D. Geerdink , C. Farès , N. Maulide , Angew. Chem. Int. Ed. 2014, 53, 5462–5466. 10.1002/anie.201402229 24740762

[55] B. Peng , X. Huang , L.‐G. Xie , N. Maulide , Angew. Chem. Int. Ed. 2014, 53, 8718–8721. 10.1002/anie.201310865 24590501

[56] T. Yanagi , S. Otsuka , Y. Kasuga , K. Fujimoto , K. Murakami , K. Nogi , H. Yorimitsu , A. Osuka , J. Am. Chem. Soc. 2016, 138, 14582–14585. 27794615 10.1021/jacs.6b10278

[57] H. J. Shrives , J. A. Fernández‐Salas , C. Hedtke , A. P. Pulis , D. J. Procter , Nat. Commun. 2017, 8, 14801. 28317882 10.1038/ncomms14801 PMC5364387

[58] D. Kaldre , B. Maryasin , D. Kaiser , O. Gajsek , L. González , N. Maulide , Angew. Chem. Int. Ed. 2017, 56, 2212–2215. 10.1002/anie.201610105 28097797

[59] Z. He , H. J. Shrives , J. A. Fernández‐Salas , A. Abengózar , J. Neufeld , K. Yang , A. P. Pulis , D. J. Procter , Angew. Chem. Int. Ed. 2018, 57, 5759–5764. 10.1002/anie.201801982 29528177

[60] T. Yanagi , H. Yorimitsu , Chem. Eur. J. 2021, 27, 13450–13456. 34322930 10.1002/chem.202101735

[61] T. Yanagi , K. Nogi , H. Yorimitsu , Chem. Eur. J. 2020, 26, 783–787. 31489707 10.1002/chem.201903570

[62] M. Tayu , A. Rahmanudin , G. J. P. Perry , R. U. Khan , D. J. Tate , R. Marcial‐Hernandez , Y. Shen , I. Dierking , Y. Janpatompong , S. Aphichatpanichakul , A. Zamhuri , I. Victoria‐Yrezabal , M. L. Turner , D. J. Procter , Chem. Sci. 2022, 13, 421–429. 35126974 10.1039/d1sc05070b PMC8730195

[63] R. Bisht , M. V. Popescu , Z. He , A. M. Ibrahim , G. E. M. Crisenza , R. S. Paton , D. J. Procter , Angew. Chem. Int. Ed. 2023, 62, e202302418. 10.1002/anie.202302418 PMC10953450 37000422

[64] M. Leypold , K. A. D'Angelo , M. Movassaghi , Org. Lett. 2020, 22, 8802–8807. 33048547 10.1021/acs.orglett.0c03160 PMC7680396

[65] L. van Dalsen , S. Zhang , W. Tian , B. W. Joynson , C. Romano , D. J. Procter , ACS Catal. 2025, 15, 8345–8352. 40401097 10.1021/acscatal.5c02029 PMC12090209

[66] C. R. Hauser , S. W. Kantor , W. R. Brasen , J. Am. Chem. Soc. 1953, 75, 2660–2663.

[67] M. Kumar , I. Sadaf , J. Pamidighantam , Anamika , S. Kumar , J. Heterocycl. Chem. 2024, 61, 29–70.

[68] S. Jana , Y. Guo , R. M. Koenigs , Chem. Eur. J. 2021, 27, 1270–1281. 32754993 10.1002/chem.202002556 PMC7894496

[69] R. Fan , C. Tan , Y. Liu , Y. Wei , X. Zhao , X. Liu , J. Tan , H. Yoshida , Chin. Chem. Lett. 2021, 32, 299–312.

[70] L.‐Q. Lu , T.‐R. Li , Q. Wang , W.‐J. Xiao , Chem. Soc. Rev. 2017, 46, 4135–4149. 28604845 10.1039/c6cs00276e

[71] Z. Sheng , Z. Zhang , C. Chu , Y. Zhang , J. Wang , Tetrahedron 2017, 73, 4011–4022.

[72] For a review of related rearrangements of sulfonium salts, see: Y. Liang , B. Peng , Acc. Chem. Res. 2022, 55, 2103–2122. 35861672 10.1021/acs.accounts.2c00263

[73] X.‐S. Liu , Z. Tang , Z. Li , M. Li , L. Xu , L. Liu , Nat. Commun. 2021, 12, 7298. 34911935 10.1038/s41467-021-27167-x PMC8674301

[74] T.‐J. Lee , W. J. Holtz , Tetrahedron Lett. 1983, 24, 2071–2072.

[75] M. Liao , L. Peng , J. Wang , Org. Lett. 2008, 10, 693–696. 18237182 10.1021/ol703058p

[76] Y. Li , Y. Shi , Z. Huang , X. Wu , P. Xu , J. Wang , Y. Zhang , Org. Lett. 2011, 13, 1210–1213. 21306152 10.1021/ol200091k

[77] Z. Yang , Y. Guo , R. M. Koenigs , Chem. Commun. 2019, 55, 8410–8413. 10.1039/c9cc03809d 31257375

[78] S.‐S. Li , J. Wang , J. Org. Chem. 2020, 85, 12343–12358. 32881498 10.1021/acs.joc.0c01590

[79] Z. Yang , Y. Guo , R. M. Koenigs , Chem. Eur. J. 2019, 25, 6703–6706. 30920053 10.1002/chem.201900597

[80] Y. Wang , P. Jia , Y. Hao , J. Li , R. Lai , L. Guo , Y. Wu , Tetrahedron Lett. 2022, 107, 154098.

[81] For a related rearangement of sulfur‐ylides, prepared from epoxides, see: A. Robert , M. T. Thomas , A. Foucaud , J. Chem. Soc., Chem. Commun. 1979, 1048–1050.

