# Supplementary material for: Transition Metal‐Free Formal C─H/C─H Coupling of Arylacetamides and Sulfoxides: An Interrupted Pummerer/[2,3]‐sigmatropic Rearrangement Sequence
Source: Angew Chem Int Ed Engl. 2025 Jul 29;64(37):e202511703. doi: 10.1002/anie.202511703 (PMC12416468; doi:10.1002/anie.202511703)

Transition metal-free formal C–H/C–H coupling of arylacetamides and sulfoxides: an interrupted Pummerer/[2,3]-sigmatropic rearrangement sequence

*Shibo Zhang, Ying Xin Lou, Allya Larroza, Ben W. Joynson, Ciro Romano and David J. Procter**

Department of Chemistry, University of Manchester, Oxford Road, Manchester, M13 9PL (UK)

Supporting Information

1 General Information **.......... 3**

2 Synthesis of Starting Materials **.......... 4**

2.1 General Procedure 1 (GP-1): Amide coupling using acid chlorides 4

2.2 General Procedure 2 (GP-2): Amide coupling using carboxylic acids 4

2.3 General Procedure 3 (GP-3): Amide coupling with medicinally-relevant amines 5

2.4 Synthesis of Sulfoxides 26

3 Synthesis of Amido Sulfonium Salts and their Rearrangement **.......... 31**

3.1 General Procedure 6 (GP-6): Synthesis of Amido Sulfonium Salts 31

3.2 General Procedure 7 (GP-7): Rearrangement of Amido Sulfonium Salts (sodium *tert*-butoxide) 34

3.3 General Procedure 8 (GP-8): Rearrangement of Amido Sulfonium Salts (potassium hydroxide)34

3.4 General Procedure 9 (GP-9): Rearrangement of Amido Sulfonium Salts (sodium *tert*-butoxide telescoped from amide) 34

4 Manipulation of Rearrangement Products **.......... 94**

5 References **.......... 98**

6 NMR Spectra **.......... 100**

1 General Information

Reagents were purchased from commercial suppliers and used as received. Procedures requiring inert conditions were conducted in flame- or oven-dried glassware under an atmosphere of anhydrous dinitrogen using standard Schlenk techniques. All solvents were purchased at 99.8% purity. Anhydrous solvents were purchased from Sigma-Aldrich and used as supplied. Deuterated chloroform was stored over 4 Å molecular sieves.

NMR spectra were recorded at 298 K (^{1}H, 500 / 400 MHz; ^{13}C, 125 / 101 MHz; ^{19}F, 471 / 376 MHz). Chemical shifts (δ) are reported in ppm; coupling constants, *J*, are reported in Hz. Signals are reported as singlet (s), doublet (d), triplet (t), quartet (q), septet (sept), multiplet (m), broad (br), apparent (app), and combinations thereof. Chemical shifts are reported relative to tetramethylsilane (TMS) and referenced to the appropriate residual solvent peaks for ^{1}H and ^{13}C{^{1}H} NMR respectively:

$CDCl_3$: 7.26 ppm, 77.16 ppm.

CD_3CN: 1.94 ppm, 1.32 ppm.

High-resolution mass spectra were obtained using electron impact ionization (EI) and chemical ionization (CI), electrospray ionization (ESI), or atmospheric pressure chemical ionization (APCI). Column chromatography was accomplished using silica gel 60 Å (40-60 *μ*m particle size) used as purchased from Sigma-Aldrich. Analytical thin-layer chromatography was carried out on aluminium-backed silica gel plates (Merck/EMD Millipore, 60 Å pore size, precoated with a 254 nm responsive fluorescent dye). Compounds were visualized by exposure to UV radiation (254 nm) or by staining with potassium permanganate followed by heating.

2 *Synthesis of Starting Materials*

2.1 General Procedure 1 (GP-1): Amide coupling using acid chlorides

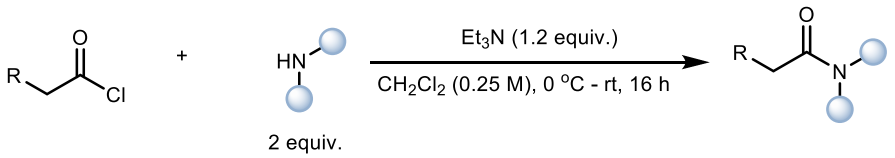

To a solution of secondary amine (10.0 mmol) and Et_3N (0.84 mL, 6.0 mmol) in CH_2Cl_2 (20 mL, 0.25 M) cooled to 0 °C (ice/water bath) was added the acid chloride (5.0 mmol) drop-wise. After complete addition, the reaction was allowed to warm to room temperature and stirred overnight. The reaction was quenched by the addition of 1 M HCl (10 mL) and the CH_2Cl_2 layer was separated. The aqueous layer was extracted with CH_2Cl_2 (3 × 10 mL) and the combined organic layers were washed with saturated aqueous $NaHCO_3$ (50 mL), dried over Na_2SO_4, and concentrated *in vacuo*. If required, purification by column chromatography with the eluents given afforded the pure product.

2.2 General Procedure 2 (GP-2): Amide coupling using carboxylic acids

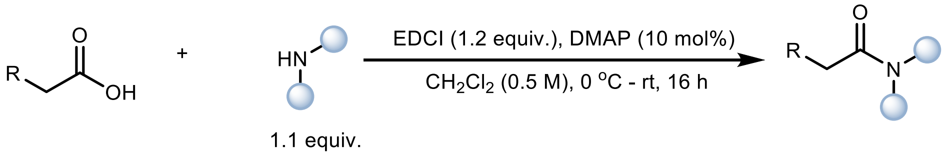

To a solution of carboxylic acid (5.0 mmol) in CH_2Cl_2 (10 mL, 0.5 M) cooled to 0 °C (ice/water bath) was added secondary amine (5.5 mmol), EDC•HCl (1.15 g, 6.0 mmol), and DMAP (61 mg, 0.5 mmol), sequentially. The reaction was allowed to warm to room temperature and stirred overnight. The reaction was quenched by the addition of 1 M HCl (10 mL) and the CH_2Cl_2 layer was separated. The aqueous layer was extracted with CH_2Cl_2 (3 × 10 mL) and the combined organic layers were washed with saturated aqueous $NaHCO_3$ (50 mL), dried over Na_2SO_4, and concentrated *in vacuo*. If required, purification by column chromatography with the eluents given afforded the pure product.

2.2.1 General Procedure 3 (GP-3): Amide coupling with medicinally-relevant amines

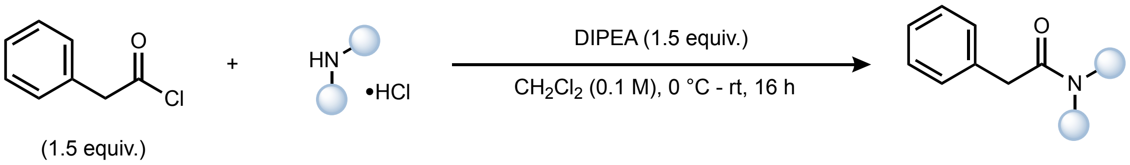

According to the modified literature procedure,[1] to a solution of amine hydrochloride (2.0 mmol) in CH_2Cl_2 (10 mL, 0.1 M) cooled to 0 °C (ice/water bath) was added DIPEA (0.52 mL, 3.0 mmol) followed by phenylacetyl chloride (0.40 mL, 3.0 mmol) drop-wise. The reaction was allowed to warm to room temperature and stirred overnight. The reaction was quenched by the addition of either 1 M HCl (10 mL) or saturated aqueous $NaHCO_3$ (10 mL) for amides with basic functional groups, and the CH_2Cl_2 layer was separated. The aqueous layer was extracted with CH_2Cl_2 (3 × 10 mL) and the combined organic layers were washed with saturated aqueous $NaHCO_3$ (50 mL), dried over Na_2SO_4, and concentrated *in vacuo*. Purification by column chromatography with the eluents given afforded the pure product.

2-Phenyl-1-morpholinoethan-1-one (1a)

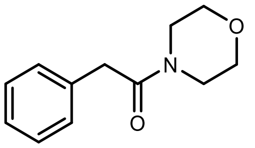

$C_{12}H_{15}NO_2$
MW: 205.26

Synthesised according to **GP-1** from 2-phenylacetyl chloride (6.6 mL, 50.0 mmol), morpholine (8.6 mL, 100.0 mmol), and Et_3N (8.4 mL, 60.0 mmol) in CH_2Cl_2 (150 mL). Purification by column chromatography (silica gel; EtOAc) afforded the product as a white solid (9.22 g, 44.9 mmol, 90%).

^{1}H NMR (400 MHz, $CDCl_3$): δ 7.35 – 7.30 (m, 2H, Ar *H*), 7.28 – 7.22 (m, 3H, Ar *H*), 3.74 (s, 2H, C(O)CH_2), 3.65 (s, 4H, 2 x CH_2), 3.50 – 3.41 (m, 4H, 2 x CH_2).

^{13}C{^{1}H} NMR (101 MHz, $CDCl_3$): δ 169.8 (*C*=O), 134.9 (Ar *C*), 129.0 (Ar *C*H), 128.7 (Ar *C*H), 127.1 (Ar *C*H), 67.0 (*C*H_2), 66.6 (*C*H_2), 46.7 (*C*H_2), 42.3 (*C*H_2), 41.0 (C(O)*C*H_2).

HRMS: calcd. for $C_{12}H_{15}NO_2Na$ $[M+Na]^+$: 228.0995; found (ESI$^+$): 228.0996.

Characterization data are consistent with literature values.[2]

2-(4-(*tert*-Butyl)phenyl)-1-morpholinoethan-1-one (1b)

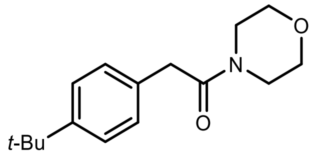

$C_{16}H_{23}NO_2$
MW: 261.37

Synthesised according to **GP-2** from 2-(4-(*tert*-butyl)phenyl)acetic acid (0.96 g, 5.0 mmol), morpholine (0.44 mL, 5.5 mmol), EDCI (1.15 g, 6.0 mmol), DMAP (0.06 g, 0.5 mmol) in CH_2Cl_2 (10 mL). Purification by column chromatography (silica gel; 20-50% EtOAc in hexanes) afforded the product as an off white solid (1.18 g, 4.5 mmol, 90%).

^{1}H NMR (400 MHz, $CDCl_3$): δ 7.37 – 7.31 (m, 2H, Ar *H*), 7.19 – 7.14 (m, 2H, Ar H), 3.69 (2H, s, C(O)C*H*$_2$), 3.65 (br., 4H, C*H*$_2$), 3.52 – 3.48 (m, 2H, C*H*$_2$), 3.47 – 3.42 (m, 2H, C*H*$_2$), 1.30 (s, 9H, C(C*H*$_3$)$_3$).

^{13}C{^{1}H} NMR (101 MHz, $CDCl_3$): δ 170.0 (*C*=O), 149.9 (Ar *C*), 131.8 (Ar *C*), 128.3 (Ar *C*H), 125.8 (Ar *C*H), 67.0 (*C*H$_2$), 66.6 (*C*H$_2$), 46.7 (*C*H$_2$), 42.3 (*C*H$_2$), 40.4 (C(O)*C*H$_2$), 34.6 (*C*(CH$_3$)$_3$), 31.5 (C(*C*H$_3$)$_3$).

HRMS: calcd. for $C_{16}H_{23}NO_2Na$ $[M+Na]^+$: 284.1621; found (ESI$^+$): 284.1635.

Characterization data are consistent with literature values.[3]

2-(4-Methylphenyl)-1-morpholinoethan-1-one (1c)

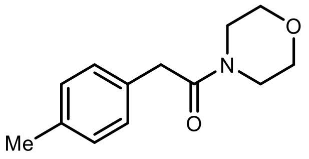

$C_{13}H_{17}NO_2$
MW: 219.28

Synthesised according to **GP-2** from 2-(4-methylphenyl)acetic acid (751 mg, 5.0 mmol), morpholine (0.47 mL, 5.5 mmol), EDC•HCl (1.15 g, 6.0 mmol), and DMAP (61 mg, 0.5 mmol) in CH_2Cl_2 (10 mL). The pure product was afforded without further purification as a white solid (1.08 g, 4.94 mmol, 98%).

^{1}H NMR (400 MHz, $CDCl_3$): δ 7.15 – 7.09 (m, 4H, Ar *H*), 3.68 (s, 2H C(O)CH_2), 3.67 – 3.63 (m, 4H, 2 × CH_2), 3.50 – 3.44 (m, 2H, CH_2), 3.44 – 3.37 (m, 2H, CH_2), 2.32 (s, 3H, CH_3).

^{13}C{^{1}H} NMR (101 MHz, $CDCl_3$): δ 169.9 (*C*=O), 136.6 (Ar *C*), 131.8 (Ar *C*), 129.6 (Ar *C*H), 128.5 (Ar *C*H), 66.9 (*C*H_2), 66.6 (*C*H_2), 46.6 (*C*H_2), 42.2 (*C*H_2), 40.6 (C(O)*C*H_2), 21.1 (*C*H_3).

HRMS: calcd. for $C_{13}H_{17}NO_2Na$ $[M+Na]^+$: 242.1151; found (ESI$^+$): 242.1159.

Characterization data are consistent with literature values.[4]

1-Morpholino-2-(*o*-tolyl)ethan-1-one (1d)

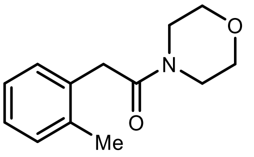

$C_{13}H_{17}NO_2$
MW: 219.28

Synthesised according to **GP-2** from 2-(*o*-tolyl)acetic acid (0.75 g, 5.0 mmol), morpholine (0.44 mL, 5.5 mmol), EDCI (1.15 g, 6.0 mmol), DMAP (0.06 g, 0.5 mmol) in CH_2Cl_2 (10 mL). Purification by column chromatography (silica gel; 20-50% EtOAc in hexanes) afforded the product as a white solid (0.89 g, 4.0 mmol, 80%).

^{1}H NMR (500 MHz, $CDCl_3$): δ 7.19 – 7.14 (m, 3H, Ar C*H*), 7.13 – 7.09 (m, 1H, Ar C*H*), 3.67 (br., 4H, CH_2), 3.66 (s, 2H, C(O)CH_2), 3.56 – 3.52 (m, 2H, CH_2), 3.41 – 3.37 (m, 2H, CH_2), 2.27 (s, 3H, CH_3).

^{13}C{^{1}H} NMR (126 MHz, $CDCl_3$): δ 169.7 (*C*=O), 136.3 (Ar *C*), 133.5 (Ar *C*), 130.4 (Ar *C*H), 128.6 (Ar *C*H), 127.1 (Ar *C*H), 126.3 (Ar *C*H), 66.9 (*C*H_2), 66.6 (*C*H_2), 46.4 (*C*H_2), 42.1 (*C*H_2), 38.3 (C(O)*C*H_2), 19.7 (*C*H_3).

HRMS: calcd. for $C_{13}H_{17}NO_2Na$ $[M+Na]^+$: 242.1151; found (ESI^+): 242.1161.

Characterization data are consistent with literature values.[3]

1-Morpholino-2-(*m*-tolyl)ethan-1-one (1e)

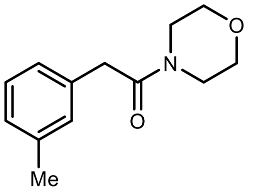

$C_{13}H_{17}NO_2$
MW: 219.28

Synthesised according to **GP-2** from 2-(*m*-tolyl)acetic acid (0.75 g, 5.0 mmol), morpholine (0.44 mL, 5.5 mmol), EDCI (1.15 g, 6.0 mmol), DMAP (0.06 g, 0.5 mmol) in CH_2Cl_2 (10 mL). Purification by column chromatography (silica gel; 20-50% EtOAc in hexanes) afforded the product as an off white solid (1.03 g, 4.7 mmol, 94%).

^{1}H NMR (400 MHz, $CDCl_3$): δ 7.21 (t, *J* = 7.8 Hz, 1H, Ar C*H*), 7.08 – 7.04 (m, 2H, Ar C*H*), 7.02 (d, *J* = 7.6 Hz, 1H, Ar C*H*), 3.69 (s, 2H, C(O)CH_2), 3.64 (br., 4H, CH_2), 3.50 – 3.46 (m, 2H, CH_2), 3.45 – 3.41 (m, 2H, CH_2), 2.33 (s, 3H, CH_3).

^{13}C{^{1}H} NMR (101 MHz, $CDCl_3$): δ 169.9 (*C*=O), 138.6 (Ar *C*), 134.8 (Ar *C*), 129.3 (Ar *C*H), 128.8 (Ar *C*H), 127.8 (Ar *C*H), 125.6 (Ar *C*H), 66.9 (*C*H_2), 66.6 (*C*H_2), 46.6 (*C*H_2), 42.2 (*C*H_2), 40.9 (C(O)CH_2), 21.5 (CH_3).

HRMS: calcd. for $C_{13}H_{17}NO_2Na$ $[M+Na]^+$: 242.1151; found (ESI^+): 242.1162.

Characterization data are consistent with literature values.[3]

2-(3-Methoxyphenyl)-1-morpholinoethan-1-one (1f)

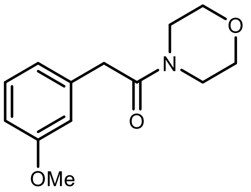

$C_{13}H_{17}NO_3$
MW: 235.28

Synthesised according to **GP-2** from 2-(3-methoxyphenyl)acetic acid (0.83 g, 5.0 mmol), morpholine (0.44 mL, 5.5 mmol), EDCI (1.15 g, 6.0 mmol), DMAP (0.06 g, 0.5 mmol) in CH_2Cl_2 (10 mL). Purification by column chromatography (silica gel; 20-50% EtOAc in hexanes) afforded the product as an off white solid (0.80 g, 3.4 mmol, 68%).

^{1}H NMR (400 MHz, $CDCl_3$): δ 7.23 – 7.15 (m, 1H, Ar C*H*), 6.80 – 6.71 (m, 3H, Ar *H*), 3.74 (s, 3H, OCH_3), 3.66 (s, 2H, C(O)CH_2), 3.59 (s, 4H, CH_2), 3.47 – 3.34 (m, 4H, CH_2).

^{13}C{^{1}H} NMR (101 MHz, $CDCl_3$): δ 169.4 (*C*=O), 159.9 (Ar *C*), 136.3 (Ar *C*), 129.7 (Ar *C*H), 120.8 (Ar *C*H), 114.1 (Ar *C*H), 112.3 (Ar *C*H), 66.7 (CH_2), 66.4 (CH_2), 55.1 (OCH_3), 46.5 (CH_2), 42.1 (CH_2), 40.8 (C(O)CH_2).

HRMS: calcd. for $C_{13}H_{17}NO_3Na$ $[M+Na]^+$: 258.1101; found (ESI$^+$): 258.1111.

Characterization data are consistent with literature values.[3]

2-(2-Bromo-4-methoxyphenyl)-1-morpholinoethan-1-one (1g)

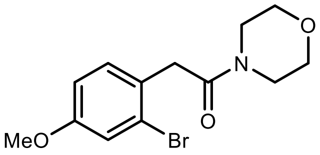

$C_{13}H_{16}BrNO_3$
MW: 314.18

Synthesised according to **GP-2** from 2-(2-bromo-4-methoxyphenyl)acetic acid (1.23 g, 5.0 mmol), morpholine (0.44 mL, 5.5 mmol), EDCI (1.15 g, 6.0 mmol), DMAP (0.06 g, 0.5 mmol) in CH_2Cl_2 (10 mL). Purification by column chromatography (silica gel; 20-50% EtOAc in hexanes) afforded the product as a white solid (1.42 g, 4.4 mmol, 90%).

^{1}H NMR (500 MHz, CDCl$_3$): δ 7.21 (d, *J* = 8.5 Hz, 1H, Ar C*H*), 7.12 (d, *J* = 2.6 Hz,1H, Ar C*H*), 6.84 (dd, *J* = 8.6, 2.7 Hz, 1H, Ar C*H*), 3.79 (s, 3H, OC*H*$_3$), 3.75 (s, 2H, C(O)C*H*$_2$), 3.70 – 3.64 (m, 4H, C*H*$_2$), 3.61 – 3.57 (m, 2H, C*H*$_2$), 3.49 – 3.44 (m, 2H, C*H*$_2$).

^{13}C{^{1}H} NMR (126 MHz, CDCl$_3$): δ 169.3 (*C*=O), 159.3 (Ar *C*), 131.1 (Ar *C*H), 126.9 (Ar *C*), 124.7 (Ar *C*), 118.1 (Ar *C*H), 114.0 (Ar *C*H), 67.0 (*C*H$_2$), 66.7 (*C*H$_2$), 55.7 (O*C*H$_3$), 46.5 (*C*H$_2$), 42.4 (*C*H$_2$), 39.7 (C(O)*C*H$_2$).

HRMS: calcd. for $C_{13}H_{17}BrNO_3$ $[M+H]^+$: 314.0386; found (ESI$^+$): 314.0395.

ν (neat/cm^{-1}): 1030, 1114, 1235, 1436, 1494, 1605, 1646, 2854, 2962.

m.p.: 98-100 °C.

2-(4-Bromophenyl)-1-morpholinoethan-1-one (1h)

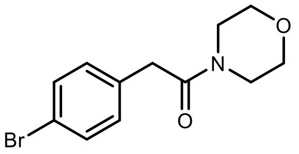

$C_{12}H_{14}BrNO_2$
MW: 284.15

Synthesised according to **GP-2** from 2-(4-bromophenyl)acetic acid (1.08 g, 5.0 mmol), morpholine (0.48 mL, 5.5 mmol), EDC•HCl (1.15 g, 6.0 mmol), and DMAP (61 mg, 0.5 mmol) in CH_2Cl_2 (20 mL). Purification by column chromatography (silica gel; 20-50% EtOAc in hexanes) afforded the product as a white solid (1.27 g, 4.5 mmol, 90%).

^{1}H NMR (500 MHz, $CDCl_3$): δ 7.48 – 7.42 (m, 2H, Ar *H*), 7.15 – 7.08 (m, 2H, Ar *H*), 3.68 – 3.60 (m, 6H, CH_2), 3.55 – 3.50 (m, 2H, CH_2), 3.45 – 3.40 (m, 2H, CH_2).

^{13}C{^{1}H} NMR (126 MHz, $CDCl_3$): δ 169.2 (*C*=O), 133.9 (Ar *C*), 132.0 (Ar *C*H), 130.5 (Ar *C*H), 121.0 (Ar *C*), 66.9 (*C*H_2), 66.6 (*C*H_2), 46.6 (*C*H_2), 42.3 (*C*H_2), 40.1 (C(O)*C*H_2).

HRMS: calcd. for $C_{12}H_{15}BrNO_2$ $[M+H]^+$: 284.0281; found (ESI^+): 284.0288.

Characterization data are consistent with literature values.[3]

2-(4-Fluorophenyl)-1-morpholinoethan-1-one (1i)

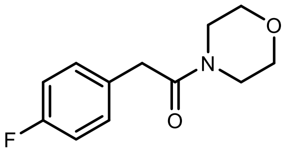

$C_{12}H_{14}FNO_2$
MW: 223.25

Synthesised according to **GP-1** from 2-(4-fluorophenyl)acetyl chloride (0.69 mL, 5.0 mmol), morpholine (0.86 mL, 10.0 mmol), and Et_3N (0.84 mL, 6.0 mmol) in CH_2Cl_2 (20 mL). The product was afforded without further purification as a white solid (1.04 g, 4.67 mmol, 93%).

^{1}H NMR (400 MHz, $CDCl_3$): δ 7.23 – 7.17 (m, 2H, Ar *H*), 7.06 – 6.97 (m, 2H, Ar *H*), 3.69 (s, 2H, C(O)CH_2), 3.69 – 3.63 (m, 4H, 2 × CH_2), 3.52 (dd, *J* = 5.6, 3.7 Hz, 2H, CH_2), 3.44 (dd, *J* = 5.6, 3.7 Hz, 2H, CH_2).

^{13}C{^{1}H} NMR (101 MHz, $CDCl_3$): δ 169.6 (*C*=O), 162.0 (d, *J* = 245.5 Hz, Ar *C*F), 130.6 (d, *J* = 3.4 Hz, Ar *C*), 130.3 (d, *J* = 8.1 Hz, Ar *C*H), 115.8 (d, *J* = 21.4 Hz, Ar *C*H), 66.9 (*C*H_2), 66.6 (*C*H_2), 46.6 (*C*H_2), 42.3 (*C*H_2), 39.9 (C(O)*C*H_2).

^{19}F NMR (376 MHz, $CDCl_3$): δ -115.84 (tt, *J* = 13.9, 5.3 Hz).

HRMS: calcd. for $C_{12}H_{14}FNO_2Na$ $[M+Na]^+$: 246.0901; found (ESI$^+$): 246.0905.

Characterization data are consistent with literature values.[3]

2-(4-Trifluoromethylphenyl)-1-morpholinoethan-1-one (1j)

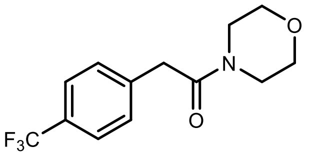

$C_{13}H_{14}F_3NO_2$
MW: 273.26

Synthesised according to **GP-1** from 2-(4-trifluoromethylphenyl)acetyl chloride (1.32 mL, 10.0 mmol), morpholine (1.73 mL, 10.0 mmol), and Et_3N (1.67 mL, 12.0 mmol) in CH_2Cl_2 (80 mL). Purification by column chromatography (silica gel; EtOAc) afforded the product as a white solid (1.77 g, 8.02 mmol, 80%).

^{1}H NMR (400 MHz, CDCl$_3$): δ 7.59 (d, *J* = 8.0 Hz, 2H, Ar *H*), 7.36 (d, *J* = 8.0 Hz, 2H, Ar *H*), 3.77 (s, 2H, C(O)CH_2), 3.69 – 3.61 (m, 4H, 2 x CH_2), 3.59 – 3.41 (m, 4H, 2 x CH_2).

^{13}C{^{1}H} NMR (101 MHz, CDCl$_3$): δ 168.9 (*C*=O), 139.0 (Ar *C*), 129.3 (Ar *C*H), 129.5 (q, *J* = 32.8 Hz, Ar *C*), 125.8 (q, *J* = 3.8 Hz, Ar *C*H), 124.2 (q, *J* = 272.1 Hz, *C*F$_3$), 66.9 (*C*H$_2$), 66.6 (*C*H$_2$), 46.6 (*C*H$_2$), 42.4 (*C*H$_2$), 40.4 (C(O)*C*H$_2$).

^{19}F NMR (376 MHz, CDCl$_3$): δ -62.53 (s, C*F$_3$*).

HRMS: calcd. for $C_{13}H_{14}NO_2F_3Na$ $[M+Na]^+$: 296.0869; found (ESI$^+$): 296.0864.

Characterization data are consistent with literature values.[3]

1-Morpholino-2-(naphthalen-2-yl)ethan-1-one (1k)

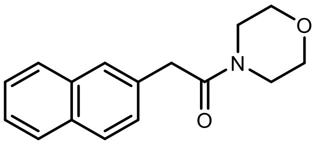

$C_{16}H_{17}NO_2$
MW: 255.32

Synthesised according to **GP-2** from 2-(2-naphthyl)acetic acid (931 mg, 5.0 mmol), morpholine (0.47 mL, 5.5 mmol), EDC•HCl (1.15 g, 6.0 mmol), and DMAP (61 mg, 0.5 mmol) in CH_2Cl_2 (10 mL). Purification by column chromatography (silica gel; 50% EtOAc in hexanes) afforded the product as a white solid (888 mg, 3.48 mmol, 70%).

^{1}H NMR (400 MHz, $CDCl_3$): δ 7.84 – 7.76 (m, 3H, Ar *H*), 7.68 (d, *J* = 1.8 Hz, 1H, Ar *H*), 7.50 – 7.43 (m, 2H, Ar *H*), 7.38 (dd, *J* = 8.5, 1.9 Hz, 1H, Ar *H*), 3.90 (s, 2H, C(O)CH_2), 3.70 – 3.63 (m, 4H, 2 x CH_2), 3.46 (br., 4H, 2 x CH_2).

^{13}C{^{1}H} NMR (101 MHz, $CDCl_3$): δ 169.7 (*C*=O), 133.7 (Ar *C*), 132.5 (Ar *C*), 132.4 (Ar *C*), 128.7 (Ar *C*H), 127.8 (Ar *C*H), 127.7 (Ar *C*H), 127.1 (Ar *C*H), 126.8 (Ar *C*H), 126.4 (Ar *C*H), 126.0 (Ar *C*H), 66.9 (*C*H_2), 66.6 (*C*H_2), 46.7 (*C*H_2), 42.3 (*C*H_2), 41.2 (C(O)*C*H_2).

HRMS: calcd. for $C_{16}H_{17}NO_2Na$ $[M+Na]^+$: 278.1152; found (ESI+): 278.1143.

Characterization data are consistent with literature values.[5]

2-Phenyl-1-(4-tosylpiperazin-1-yl)ethan-1-one (1l)

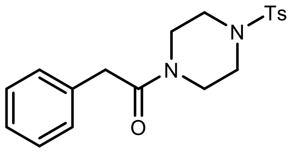

$C_{19}H_{22}N_2O_3S$
MW: 358.46

Synthesised according to **GP-1** from 2-phenylacetyl chloride (0.68 mL, 5.0 mmol), *N*-tosylpiperazine (2.40 g, 10.0 mmol), and Et_3N (0.84 mL, 6.0 mmol) in CH_2Cl_2 (30 mL). Purification by column chromatography (silica gel; 0-5% MeOH in CH_2Cl_2) afforded the product as a white solid (840 mg, 2.34 mmol, 47%).

^{1}H NMR (400 MHz, $CDCl_3$): δ 7.57 – 7.53 (m, 2H, Ar *H*), 7.32 (d, *J* = 7.9 Hz, 2H, Ar *H*), 7.25 – 7.18 (m, 3H, Ar *H*), 7.13 (dd, *J* = 7.9, 1.7 Hz, 2H, Ar *H*), 3.70 (t, *J* = 5.2 Hz, 2H, NC*H*$_2$), 3.66 (s, 2H, C(O)C*H*$_2$), 3.49 (t, *J* = 5.1 Hz, 2H, NC*H*$_2$), 2.93 (t, *J* = 5.2 Hz, 2H, NC*H*$_2$), 2.72 (t, *J* = 5.1 Hz, 2H, NC*H*$_2$), 2.45 (s, 3H, C*H*$_3$).

^{13}C{^{1}H} NMR (101 MHz, $CDCl_3$): δ 169.6 (*C*=O), 144.2 (Ar *C*), 134.5 (Ar *C*), 132.2 (Ar *C*), 129.9 (Ar *C*H), 128.9 (Ar *C*H), 128.6 (Ar *C*H), 127.9 (Ar *C*H), 127.1 (Ar *C*H), 45.9 (*C*H$_2$), 45.8 (*C*H$_2$), 45.6 (*C*H$_2$), 41.2 (*C*H$_2$), 41.1 (*C*H$_2$), 21.7 (*C*H$_3$).

HRMS: calcd. for $C_{19}H_{22}N_2O_3SNa$ $[M+H]^+$: 381.1243; found (ESI$^+$): 381.1260.

v_{max} (neat/cm^{-1}): 696, 730, 947, 1111, 1159, 1233, 1342, 1412, 1655, 2847.

m.p.: 173-175 °C.

2-Phenyl-1-(pyrrolidin-1-yl)ethan-1-one (1m)

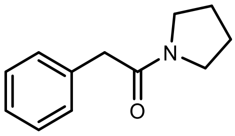

$C_{12}H_{15}NO$
MW: 189.26

Synthesised according to **GP-1** from 2-phenylacetyl chloride (0.68 mL, 5.0 mmol), pyrrolidine (0.82 mL, 10.0 mmol), and Et_3N (0.84 mL, 6.0 mmol) in CH_2Cl_2 (20 mL). Purification by column chromatography (silica gel; 50-70% EtOAc in hexanes) afforded the product as a white solid (902 mg, 4.77 mmol, 95%).

^{1}H NMR (400 MHz, $CDCl_3$): δ 7.34 – 7.21 (m, 5H, Ar *H*), 3.66 (s, 2H, PhC*H*$_2$), 3.49 (t, *J* = 6.7 Hz, 2H, C*H*$_2$), 3.42 (t, *J* = 6.7 Hz, 2H, C*H*$_2$), 1.95 – 1.79 (m, 4H, 2 x C*H*$_2$).

^{13}C{^{1}H} NMR (101 MHz, $CDCl_3$): δ 169.7 (*C*=O), 135.1 (Ar *C*), 129.1 (Ar *C*H), 128.7 (Ar *C*H), 126.8 (Ar *C*H), 47.0 (*C*H$_2$), 46.1 (*C*H$_2$), 42.5 (C(O)*C*H$_2$), 26.3 (*C*H$_2$), 24.5 (*C*H$_2$).

HRMS: calcd. for $C_{12}H_{16}NO$ $[M+H]^+$: 190.1232; found (ESI$^+$): 190.1229.

Characterization data are consistent with literature values.[7]

2-Phenyl-1-(piperidn-1-yl)ethan-1-one (1n)

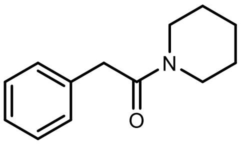

$C_{13}H_{17}NO$
MW: 203.29

Synthesised according to **GP-1** from 2-phenylacetyl chloride (0.68 mL, 5.0 mmol), piperidine (0.99 mL, 10.0 mmol), and Et_3N (0.84 mL, 6.0 mmol) in CH_2Cl_2 (20 mL). Purification by column chromatography (silica gel; 50-70% EtOAc in hexanes) afforded the product as a white solid (1.00 g, 4.94 mmol, 99%).

^{1}H NMR (400 MHz, $CDCl_3$): δ 7.35 – 7.29 (m, 2H, Ar *H*), 7.28 – 7.21 (m, 3H, Ar *H*), 3.74 (s, 2H, PhCH_2), 3.61 – 3.56 (m, 2H, CH_2), 3.40 – 3.35 (m, 2H, CH_2), 1.62 – 1.49 (m, 4H, 2 x CH_2), 1.39 – 1.31 (m, 2H, CH_2).

^{13}C{^{1}H} NMR (101 MHz, $CDCl_3$): δ 169.3 (*C*=O), 135.5 (Ar *C*), 128.7 (Ar *C*H), 128.7 (Ar *C*H), 126.7 (Ar *C*H), 47.3 (*C*H_2), 42.9 (*C*H_2), 41.3 (C(O)*C*H_2), 26.3 (*C*H_2), 25.6 (*C*H_2), 24.5 (*C*H_2).

HRMS: calcd. for $C_{13}H_{18}NO$ $[M+H]^+$: 204.1388; found (ESI$^+$): 204.1382.

Characterization data are consistent with literature values.[7]

2-Phenyl-1-(2-phenylmorpholino)ethan-1-one (1o)

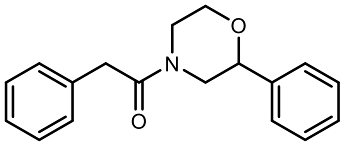

$C_{18}H_{19}NO_2$
MW: 281.36

Synthesised according to **GP-2** from 2-phenylacetic acid (680 mg, 5.0 mmol), 2-phenylmorpholine (897 mg, 5.5 mmol), EDC•HCl (1.15 g, 6.0 mmol), and DMAP (61 mg, 0.5 mmol) in CH_2Cl_2 (10 mL). Purification by column chromatography (silica gel; 50% EtOAc in hexanes) afforded the product as a light yellow oil (1.18 g, 4.2 mmol, 84%).

A 1.5:1 mixture of rotamers was observed at 298 K in $CDCl_3$.

^{1}H NMR (400 MHz, $CDCl_3$): 7.42 – 7.23 (m, 9H, Ar *H*), 7.16 – 7.07 (m, 1H, Ar *H*), 4.69 (m, 0.4H, C*H₂*), 4.62 – 4.52 (m, 0.4H, C*H₂*), 4.39 (m, 0.6H, C*H*), 4.05 (m, 0.4H, C*H*), 3.95 (m, 1H, C*H₂*), 3.85 – 3.68 (m, 3H, C*H₂* + C(O)C*H₂*), 3.61 (m, 0.6H, C*H₂*), 3.43 (m, 0.4H, C*H₂*), 3.28 (m, 0.4H, C*H₂*), 3.05 (m, 0.6H, C*H₂*), 2.96 – 2.84 (m, 0.6H, C*H₂*), 2.73 (m, 0.4H, C*H₂*).

^{13}C{^{1}H} NMR (101 MHz, $CDCl_3$): δ 169.7 (*C*=O), 169.5 (*C*=O), 139.0 (Ar *C*), 138.7 (Ar *C*), 135.1 (Ar *C*), 134.9 (Ar *C*), 129.0 (Ar *C*H), 128.9 (Ar *C*H), 128.6 (Ar *C*H), 128.6 (Ar *C*H), 128.6 (Ar *C*H), 128.6 (Ar *C*H), 128.3 (Ar *C*H), 128.2 (Ar *C*H), 127.1 (Ar *C*H), 127.0 (Ar *C*H), 126.1 (Ar *C*H), 126.0 (Ar *C*H), 78.1 (*C*H), 77.9 (*C*H), 66.9 (*C*H₂), 66.5 (*C*H₂), 53.0 (*C*H₂), 48.0 (*C*H₂), 46.1 (*C*H₂), 41.7 (*C*H₂), 41.4 (C(O)*C*H₂), 41.2 (C(O)*C*H₂).

HRMS: calcd. for $C_{16}H_{17}NO_2Na$ $[M+H]^+$: 282.1489; found (ESI$^+$): 282.1497.

ν_{max} (neat/cm^{-1}): 753, 769, 789, 1131, 1409, 1462, 1680, 2966.

***N,N*-Dimethyl-2-phenylacetamide (1p)**

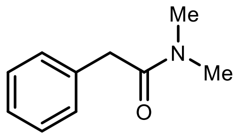

$C_{10}H_{13}NO$
MW: 163.22

Synthesised according to **GP-1** from 2-phenylacetyl chloride (0.68 mL, 5.0 mmol), dimethylamine hydrochloride (820 mg, 10.0 mmol), and Et_3N (0.84 mL, 6.0 mmol) in CH_2Cl_2 (20 mL). Purification by column chromatography (silica gel; 20-50% EtOAc in hexanes) afforded the product as a white solid (500 mg, 3.08 mmol, 62%).

^{1}H NMR (400 MHz, $CDCl_3$): δ 7.35 – 7.30 (m, 2H, Ar *H*), 7.28 – 7.22 (m, 3H, Ar *H*), 3.73 (s, 2H, PhCH_2), 3.00 (s, 3H, CH_3), 2.97 (s, 3H, CH_3).

^{13}C{^{1}H} NMR (101 MHz, $CDCl_3$): δ 171.2 (*C*=O), 135.1 (Ar *C*), 128.8 (Ar *C*H), 128.7 (Ar *C*H), 126.8 (Ar *C*H), 41.1 (Ph*C*H_2), 37.9 (*C*H_3), 35.7 (*C*H_3).

HRMS: calcd. for $C_{10}H_{14}NO$ $[M+H]^+$: 164.1070; found (ESI$^+$): 164.1071.

Characterization data are consistent with literature values.[6]

N-Methoxy-N-methyl-2-phenylacetamide (1q)

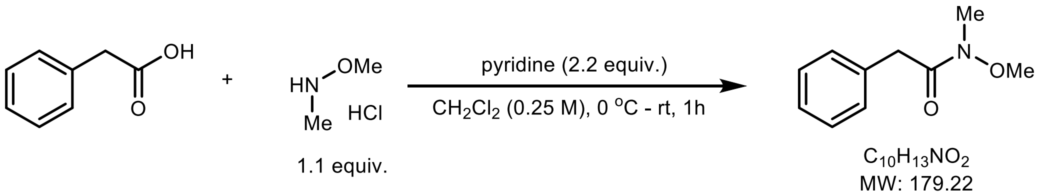

According to the literature procedure,[11] phenylacetic acid (0.68 g, 5.0 mmol) was added to a suspension of *N*,*O*-dimethylhydroxylamine hydrochloride (585 mg, 6.0 mmol) in CH_2Cl_2 (20 mL) cooled to 0 °C (ice/water bath). Pyridine (0.89 mL, 11.0 mmol) was then added drop-wise, and the resulting solution was stirred at 0 °C for 5 min then warmed to room temperature and stirred for 1 h. The reaction was quenched by the addition of 1 M HCl (10 mL) and the CH_2Cl_2 layer was separated. The aqueous layer was extracted with CH_2Cl_2 (3 × 10 mL) and the combined organic layers were washed with saturated aqueous $NaHCO_3$ (50 mL), dried over Na_2SO_4, and concentrated *in vacuo*. The product was afforded without further purification as a colourless oil (650 mg, 3.63 mmol, 73%).

^{1}H NMR (400 MHz, $CDCl_3$): δ 7.34 – 7.27 (m, 4H, Ar*H*), 7.27 – 7.21 (m, 1H, Ar*H*), 3.78 (s, 2H, C(O)C*H₂*), 3.60 (s, 3H, OC*H₃*), 3.19 (s, 3H, NC*H₃*).

^{13}C{^{1}H} NMR (101 MHz, $CDCl_3$): δ 172.5 (*C*=O), 135.0 (Ar *C*), 129.4 (Ar *C*H), 128.6 (Ar *C*H), 126.8 (Ar *C*H), 61.3 (O*C*H_3), 39.5 (C(O)*C*H_2), 32.3 (N*C*H_3).

HRMS: calcd. for $C_{10}H_{13}NO_2Na$ $[M+Na]^+$: 202.0838; found (ESI^+): 202.0845.

Characterization data are consistent with literature values.[8]

N-(3-(9,10-Ethanoanthracen-9(10H)-yl)propyl)-N-methyl-2-phenylacetamide (1r)

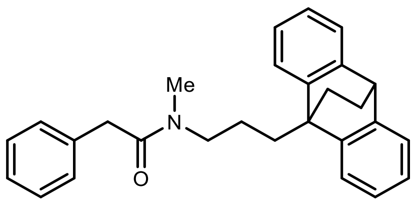

$C_{28}H_{29}NO$
MW: 395.55

Synthesised according to **GP-3** from maprotiline hydrochloride (629 mg, 2.0 mmol), DIPEA (0.52 mL, 3.0 mmol), and phenylacetyl chloride (0.40 mL, 3.0 mmol) in CH_2Cl_2 (20 mL). Purification by column chromatography (silica gel; 30% EtOAc) in hexanes) afforded the product as a white solid (683 mg, 1.72 mmol, 86%).

A 1.5:1 mixture of rotamers was observed at 298 K in $CDCl_3$.

^{1}H NMR (400 MHz, $CDCl_3$): δ 7.38 – 7.17 (m, 8H, Ar *H*), 7.14 – 7.04 (m, 5H, Ar *H*), 4.35 – 4.26 (m, 1H, C*H*), 3.87 (s, 0.8H, CH_2), 3.78 (s, 1.2H, CH_2), 3.69 (t, J = 7.4 Hz, 1.2H, NCH_2), 3.58 (t, J = 7.8 Hz, 0.8H, NCH_2), 3.08 (s, 1.7H, NCH_3), 3.06 (s, 1.3H, NCH_3), 2.48 – 2.39 (m, 1.2H, CH_2), 2.35 – 2.25 (m, 0.8H, CH_2), 2.07 – 1.96 (m, 1.2H, CH_2), 1.96 – 1.86 (m, 0.8H, CH_2), 1.82 (ddd, J = 10.4, 4.3, 2.8 Hz, 2H, HC-CH_2), 1.60 – 1.51 (m, 1.2H, HC-CH_2CH_2), 1.51 – 1.44 (m, 0.8H, HC-CH_2CH_2).

^{13}C NMR (101 MHz, $CDCl_3$): δ 171.2 (*C*=O), 171.0 (*C*=O), 145.4 (Ar *C*), 145.1 (Ar *C*), 145.0 (Ar *C*), 135.5 (Ar *C*), 135.2 (Ar *C*), 128.94 (Ar *C*H), 128.87 (Ar *C*H), 128.85 (Ar *C*H), 128.8 (Ar *C*H), 127.0 (Ar *C*H), 126.9 (Ar *C*H), 125.5 (Ar *C*H), 125.44 (Ar *C*H), 125.42 (Ar *C*H), 125.35 (Ar *C*H), 123.6 (Ar *C*H), 123.5 (Ar *C*H), 121.3 (Ar *C*H), 121.0 (Ar *C*H), 51.3 (N*C*H_2), 48.9 (N*C*H_2), 44.8 (*C*H), 44.62 (*C*H), 44.55 (*C*H), 41.6 (*C*H_3), 41.3 (*C*H_3), 36.1 (*C*H_3), 33.7 (*C*H_3), 29.7 (*C*H_2), 28.14 (*C*H_2), 28.08 (*C*H_2), 27.8 (*C*H_2), 27.6 (*C*H_2), 24.0 (*C*H_2), 22.9 (*C*H_2).

HRMS: $C_{25}H_{24}F_3NO_2Na$ $[M+Na]^+$: 418.2141; found (ESI$^+$): 418.2155.

ν_{max} (neat/cm^{-1}): 725, 749, 762, 1115, 1401, 1450, 1628, 2936.

m.p.: 100-102 °C

N-Methyl-2-phenyl-*N*-(3-phenyl-3-(4-(trifluoromethyl)phenoxy)propyl)acetamide (1s)

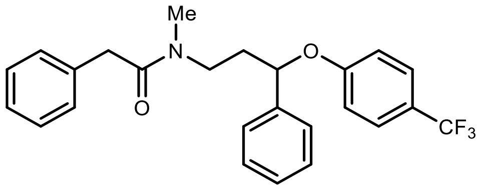

$C_{25}H_{24}F_3NO_2$
MW: 427.47

Synthesised according to **GP-3** from fluoxetine hydrochloride (1.73 g, 5.0 mmol), DIPEA (1.31 mL, 7.5 mmol), and phenylacetyl chloride (0.99 mL, 7.5 mmol) in CH_2Cl_2 (50 mL). Purification by column chromatography (silica gel; 20-40% EtOAc) in hexanes) afforded the product as a colourless viscous oil (1.94 g, 4.53 mmol, 91%).

A 1.2:1 mixture of rotamers was observed at 298 K in $CDCl_3$.

^{1}H NMR (400 MHz, $CDCl_3$): δ 7.45 (d, J = 8.8 Hz, 0.8H, Ar *H*), 7.41 (d, J = 8.8 Hz, 1.2H, Ar *H*), 7.39 – 7.19 (m, 9H, Ar *H*), 7.11 (dd, J = 6.9, 1.8 Hz, 0.8H, Ar *H*), 6.87 (d, J = 8.5 Hz, 0.8H, Ar *H*), 6.83 (d, J = 8.7 Hz, 1.2H, Ar *H*), 5.13 (dd, J = 8.7, 4.3 Hz, 0.6H, OC*H*Ph), 5.10 (dd, J = 8.7, 3.8 Hz, 0.4H, OC*H*Ph), 3.70 – 3.51 (m, 3.5H, CH_2, C$H_{2A,B}$), 3.44 (ddd, J = 14.4, 9.3, 4.4 Hz, 0.5H, CH_{2B}), 2.96 (s, 1.6H, NCH_3), 2.95 (s, 1.4H, NCH_3), 2.24 – 2.07 (m, 1.6H, CH_2), 2.04 – 1.98 (m, 0.4H, CH_2).

^{13}C{^{1}H} NMR (101 MHz, $CDCl_3$): δ 171.23 (*C*=O), 171.15 (*C*=O), 160.4 (Ar *C*), 160.1 (Ar *C*), 140.8 (Ar *C*), 140.0 (Ar *C*), 135.2 (Ar *C*), 135.0 (Ar *C*), 129.2 (Ar *C*H), 128.93 (Ar *C*H), 128.88 (Ar *C*H), 128.82 (Ar *C*H), 128.79 (Ar *C*H), 128.4 (Ar *C*H), 128.1 (Ar *C*H), 127.1 (q, J = 3.8 Hz, Ar *C*H), 126.9 (q, J = 4.0 Hz, Ar *C*H), 125.8 (Ar *C*H), 125.7 (Ar *C*H), 123.4 (q, J = 32.8 Hz, Ar *C*), 123.0 (q, J = 32.5 Hz, Ar *C*), 115.8 (Ar *C*H), 115.8 (Ar *C*H), 78.3 (O*C*HPh), 77.4 (O*C*HPh), 46.7 (*C*H_2), 45.6 (*C*H_2), 41.5 (*C*H_2), 40.9 (*C*H_2), 37.3 (*C*H_2), 36.6 (N*C*H_3), 36.3 (*C*H_2), 33.6 (N*C*H_3). (*C*F_3 not observed but detected by ^{19}F NMR.)

^{19}F NMR (471 MHz, $CDCl_3$): δ -61.58 (s, 1.7F), -61.65 (s, 1.3F).

HRMS: calcd. for $C_{25}H_{24}F_3NO_2Na$ $[M+Na]^+$: 450.1651; found (ESI$^+$): 450.1671.

ν_{max} (neat/cm^{-1}): 698, 835, 1066, 1107, 1158, 1244, 1322, 1453, 1516, 1613, 1639, 2930.

2-(2-Chlorophenyl)-1-(1-methyl-3,4-dihydroisoquinolin-2(1H)-yl)ethan-1-one (1ab)

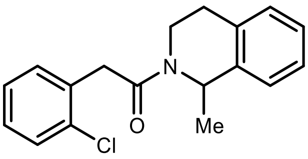

$C_{18}H_{18}NClO$
MW: 299.80

Synthesised according to **GP-2** from 2-(2-chlorophenyl)acetic acid (850 mg, 5.0 mmol), 1-methyl-1,2,3,4-tetrahydroisoquinoline (809 mg, 5.5 mmol), EDC•HCl (1.15 g, 6.0 mmol), and DMAP (61 mg, 0.5 mmol) in CH_2Cl_2 (10 mL). Purification by column chromatography (silica gel; 50% EtOAc in hexanes) afforded the product as a white solid (1.27 mg, 3.48 mmol, 85%).

A 2:1 mixture of rotamers was observed at 298 K in $CDCl_3$.

^{1}H NMR (400 MHz, $CDCl_3$): δ 7.44 – 7.04 (m, 8H, Ar *H*), 5.69 (q, *J* = 6.8 Hz, 1H, CH_3C*H*), 5.06 (q, *J* = 6.8 Hz, 1H, CH_3C*H*), 4.77 – 4.67 (m, 1H, C*H₂*), 3.49 (ddd, *J* = 13.5, 8.6, 6.6 Hz, 1H, CH_2), 3.16 – 3.04 (m, 1H, CH_2), 2.95 (ddd, *J* = 17.1, 11.7, 5.6 Hz, 1H, CH_2), 2.82 – 2.71 (m, 2H, CH_2), 1.49 (m, 3H, CH_3).

^{13}C{^{1}H} NMR (101 MHz, $CDCl_3$): δ 168.6 (*C*=O), 168.6 (*C*=O), 138.7 (Ar *C*), 137.7 (Ar *C*), 134.3 (Ar *C*), 134.1 (Ar *C*), 133.9 (Ar *C*), 133.6 (Ar *C*), 133.5 (Ar *C*), 133.3 (Ar *C*), 131.1 (Ar *C*H), 130.5 (Ar *C*H), 129.6 (Ar *C*H), 129.5 (Ar *C*H), 129.3 (Ar *C*H), 128.7 (Ar *C*H), 128.5 (Ar *C*H), 127.3 (Ar *C*H), 127.2 (Ar *C*H), 127.1 (Ar *C*H), 127.1 (Ar *C*H), 126.9 (Ar *C*H), 126.8 (Ar *C*H), 126.7 (Ar *C*H), 126.5 (Ar *C*H), 126.4 (Ar *C*H), 52.5 (*C*H), 49.0 (*C*H), 40.2 (*C*H_2), 38.7 (*C*H_2), 38.4 (*C*H_2), 35.5 (*C*H_2), 29.4 (*C*H_2), 28.7 (*C*H_2), 23.0 (*C*H_3), 21.7 (*C*H_3).

HRMS: calcd. for $C_{18}H_{19}ClNO$ $[M+H]^+$: 300.1150; found (ESI$^+$): 300.1143.

$ν_{max}$ (neat/cm^{-1}): 686, 750, 953, 1117, 1164, 1241, 1366, 1431, 1640, 2928.

m.p.: (recrystallized from EtOAc) 168-169 °C.

2.3 Synthesis of Sulfoxides

General Procedure 4 for the Preparation of Sulfoxides

For the synthesis of sulfoxides, *m*-CPBA (1 equiv., 5 mmol) was added in portions over 10 min to a cooled solution of the corresponding sulfide (1.1 equiv., 5.5 mmol) in CH_2Cl_2 (15 mL). After stirring at 0 °C for 1 h, a saturated aqueous solution of $NaHCO_3$ (15 mL) was added. The mixture was stirred at room temperature for 30 min before extraction with CH_2Cl_2 (3 x 30 mL). The combined organic layers were dried with $MgSO_4$, filtered and concentred under reduced pressure. The crude product mixture was purified by column chromatography on silica gel using ethyl acetate and methanol to give the products.

General Procedure 5 for the Preparation of Sulfoxides

To a solution of sulfide (26.8 mmol, 1 equiv.) in glacial acetic acid (7.2 mL, 5.4 equiv.) at 0 °C, H_2O_2 (30 wt%) (34.8 mmol, 1.2 mL, 1.3 equiv.) was added dropwise. After 3 h, the reaction mixture was diluted with CH_2Cl_2 (10 mL) and was filtered over Na_2SO_4. The acetic acid was evaporated under reduced pressure. The crude product mixture was purified by column chromatography on silica gel using ethyl acetate as eluent to give the products.

Tetrahydro-2*H*-thiopyran 1-oxide

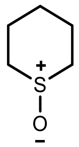

$C_5H_{10}OS$
MW: 118.19

Synthesised according to **GP-4** from tetrahydro-2*H*-thiopyran (0.57 mL, 5.5 mmol), *m*-CPBA (1.23 g, 5.0 mmol) and CH_2Cl_2 (15 mL). Purification by column chromatography (silica gel; 5-20% MeOH in EtOAc) afforded the product as white solid (307 mg, 2.6 mmol, 52%).

^{1}H NMR (400 MHz, $CDCl_3$): δ 2.87 – 2.81 (m, 2H, C*H₂*), 2.75 – 2.69 (m, 2H, C*H₂*), 2.23 – 2.14 (m, 2H, C*H₂*), 1.67 – 1.58 (m, 2H, C*H₂*), 1.57 – 1.51 (m, 2H, C*H₂*).

^{13}C{^{1}H} NMR (101 MHz, $CDCl_3$): δ 48.9 (*C*H₂), 24.6 (*C*H₂), 19.1 (*C*H₂).

HRMS: calcd. for $C_5H_{10}OS$ $[M+H]^+$: 119.0452; found ($APCI^+$): 119.0523.

Characterization data are consistent with literature values.[9]

1,4-Oxathiane 4-oxide

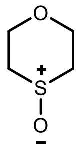

$C_4H_8O_2S$
MW: 120.17

Synthesised according to **GP-4** from 1,4-oxathiane (0.52 mL, 5.5 mmol), *m*-CPBA (1.23 g, 5.0 mmol) and CH_2Cl_2 (15 mL). Purification by column chromatography (silica gel; 5-15% MeOH in EtOAc) afforded the product as a colorless solid (312 mg, 2.6 mmol, 52%).

^{1}H NMR (400 MHz, $CDCl_3$): δ 4.42 – 4.36 (m, 2H, C*H₂*), 3.86 – 3.81 (m, 2H, C*H₂*), 2.97 – 2.90 (m, 2H, C*H₂*), 2.78 – 2.73 (m, 2H, C*H₂*).

^{13}C{^{1}H} NMR (101 MHz, $CDCl_3$): δ 59.4 ($\mathit{C}H_2$), 46.5 ($\mathit{C}H_2$).

HRMS: calcd. for $C_4H_8O_2S$ $[M+H]^+$: 121.0245; found (APCI$^+$): 121.0315.

Characterization data are consistent with literature values.[10]

Tetrahydro-2*H*-thiopyran-4-carbonitrile 1-oxide

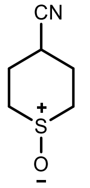

C_6H_9NOS
MW: 143.20

Synthesised according to **GP-4** from tetrahydro-2*H*-thiopyran-4-carbonitrile (0.65 mL, 5.5 mmol), *m*-CPBA (1.23 g, 5.0 mmol) and CH_2Cl_2 (15 mL). Purification by column chromatography (silica gel; 5-10% MeOH in EtOAc) afforded the product as a white solid (601 mg, 4.2 mmol, 84%).

^{1}H NMR (400 MHz, $CDCl_3$): δ 3.04 – 2.96 (m, 1H, C*H*), 2.91 – 2.78 (m, 3H, CH_2), 2.66 – 2.50 (m, 3H, CH_2), 2.02 – 1.90 (m, 2H, CH_2).

^{13}C{^{1}H} NMR (101 MHz, $CDCl_3$): δ 120.2 (*C*N), 45.1 (*C*H), 42.3 (CH_2), 26.8 (CH_2), 26.0 (CH_2), 18.4 (CH_2).

HRMS: calcd. for C_6H_9OS $[M+H]^+$: 144.0405; found (APCI$^+$): 144.0472.

(Methylsulfinyl)ethane

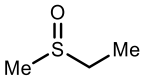

C_3H_8OS
MW: 92.16

Synthesised according to **GP-5** from ethyl(methyl)sulfide, acetic acid, and H_2O_2. Purification by column chromatography (silica gel; 50%-100% EtOAc in hexane) afforded the product as a colourless oil.

^{1}H NMR (400 MHz, CDCl$_3$): δ 2.60 – 2.41 (m, 2H, C*H*$_2$), 2.32 (s, 3H, SC*H*$_3$), 1.09 (t, *J* = 7.5 Hz, 3H, C*H*$_3$).

^{13}C{^{1}H} NMR (101 MHz, CDCl$_3$): δ 47.2 (C*H*$_2$), 37.3 (SC*H*$_3$), 6.2 (C*H*$_3$).

HRMS: calcd. for C_6H_8OSNa $[M+Na]^+$: 115.0188; found (ESI$^+$): 115.0193.

Characterization data are consistent with literature values.[11]

3.1 General Procedure 6 (GP-6): Synthesis of Amido Sulfonium Salts

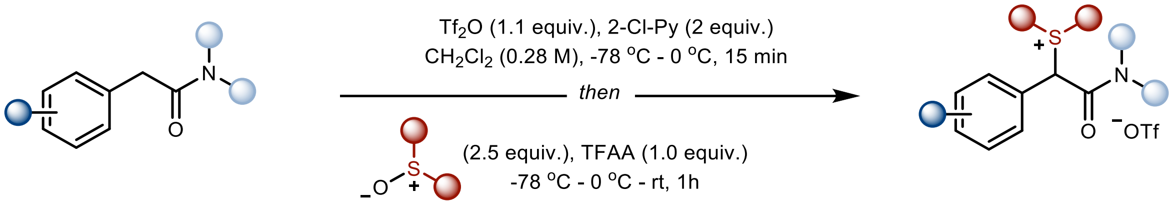

According to the modified literature procedure,[12] an oven- or flame-dried flask under an atmosphere of dinitrogen was charged with amide (1.0 mmol), 2-chloropyridine (0.19 mL, 2.0 mmol) and anhydrous CH_2Cl_2 (0.28 M, 3.5 mL). The solution was cooled to -78 °C (dry ice/acetone bath) and Tf_2O (0.18 mL, 1.1 mmol) was added drop-wise. After complete addition, the reaction was warmed to 0 °C (ice/water bath) and stirred for 15 min. After cooling once more to -78 °C, the sulfoxide (2.5 mmol) in anhydrous CH_2Cl_2 (0.5 mL) was added drop-wise, followed by drop-wise addition of TFAA (0.14 mL, 1.0 mmol). The reaction mixture was warmed to 0 °C and stirred for 15 min and at room temperature for 45 min. The reaction was quenched by addition of saturated aqueous $NaHCO_3$ (5 mL/mmol) followed by vigorous stirring for 5 min. The organic layer was separated and the aqueous layer extracted with CH_2Cl_2 (3 × 10 mL). The combined organic layers were dried over $MgSO_4$, filtered, and concentrated *in vacuo*. Purification by column chromatography using the eluents given afforded the pure product. In general, the products could be purified further by recrystallization from EtOAc if required.

3.2 Purification and robustness of sulfonium salts

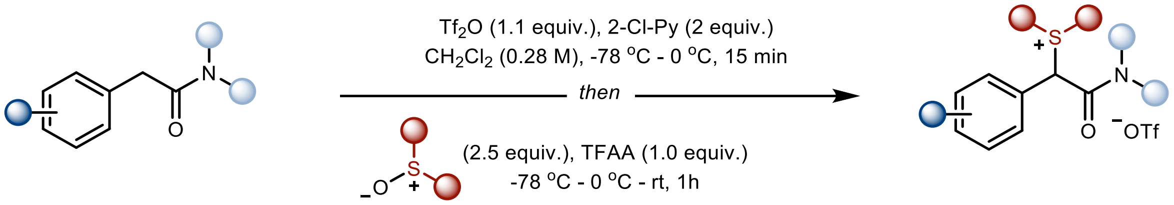

Entry	Work-up	Purification	Isolated Yield / %
1	None	Column Chromatography	52
2	K_2CO_3 plug, then hexane trituration	Column Chromatography	46
3	NaOAc plug, then hexane trituration	Column Chromatography	45
4	K_2CO_3 plug, then hexane trituration	Silica plug, then recrystallization (EtOAc)	38
5	K_2CO_3 plug, then hexane trituration	Recrystallization (EtOAc)	54
6	Aq. $NaHCO_3$, then hexane trituration	Recrystallization (EtOAc)	59
7	Aq. $NaHCO_3$	Column Chromatography	74

Supplementary Table S1: *Optimization of the work-up and purification in the formation of amido sulfonium salts.*

Discussion: In general, column chromatography was utilized to achieve the highest yield and purity, though trituration from hexanes followed by recrystallization also achieved high purity at the expense of yield. Sulfonium salts bearing more electron-withdrawing substituents were in general more crystalline and could be isolated by trituration/recrystallization with higher isolated yields. In general, sulfonium salts made *via* this method were found to be stable under ambient conditions for >1 year.

Reaction Robustness

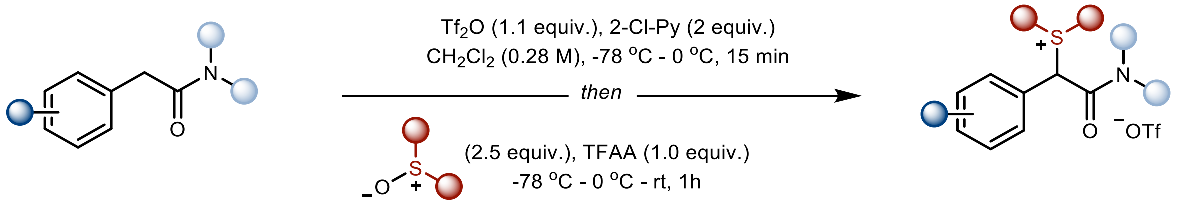

Entry	Conditions	Yield / %[a]
1	Anhydrous CH_2Cl_2, inert atmosphere	88
2	Winchester-grade CH_2Cl_2, inert atmosphere	80
3	Winchester-grade CH_2Cl_2, air	60

Supplementary Table S2: *Solvent grade tests for α-amido sulfonium salt synthesis. [a]Determined by ^1H NMR spectroscopy using CH_2Br_2 as an internal standard.*

3.3 General Procedure 7 (GP-7): Rearrangement of Amido Sulfonium Salts (Sodium *tert*-Butoxide)

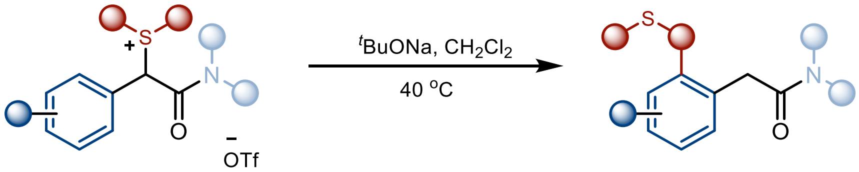

An oven-dried microwave vial was charged with sulfonium salt (0.1 mmol) and sodium *tert*-butoxide (19.2 mg, 0.2 mmol) and then sealed and evacuated and back-filled with dinitrogen 3 times. Anhydrous CH_2Cl_2 (1.0 mL, 0.1 M) was then added. The sample was stirred at 40 °C for 18 h. The crude material was filtered through a syringe filter, then purified by column chromatography using the eluents given to afford the pure product.

3.4 General Procedure 8 (GP-8): Rearrangement of Amido Sulfonium Salts (Potassium Hydroxide)

An oven-dried microwave vial was charged with sulfonium salt (0.1 mmol) and potassium hydroxide (11.2 mg, 0.2 mmol) and then sealed and evacuated and back-filled with dinitrogen 3 times. Anhydrous acetonitrile (1.0 mL, 0.1 M) was then added. The sample was stirred at 40 °C for 18 h. The crude material was filtered through a syringe filter, then purified by column chromatography using the eluents given to afford the pure product.

3.5 General Procedure 9 (GP-9): Rearrangement of Crude Amido Sulfonium Salts

An oven-dried flask under an atmosphere of dinitrogen was charged with amide (0.5 mmol), 2-chloropyridine (0.095 mL, 1.0 mmol) and anhydrous CH_2Cl_2 (0.28 M, 1.8 mL). The solution was cooled to -78 °C (dry ice/acetone bath) and Tf_2O (0.09 mL, 0.55 mmol) was added drop-wise. After complete addition, the reaction was warmed to 0 °C (ice/water bath) and stirred for 15 min. After cooling once more to -78 °C, the sulfoxide (1.25 mmol) in anhydrous CH_2Cl_2 (0.3 mL) was added drop-wise, followed by drop-wise addition of TFAA (0.07 mL, 0.5 mmol). The reaction mixture was warmed to 0 °C and stirred for 15 min and at room temperature for 45 min. The reaction was quenched by addition of saturated aqueous $NaHCO_3$ (5 mL/mmol) followed by vigorous stirring for 5 min. The organic layer was separated and the aqueous layer extracted with CH_2Cl_2 (3 × 10 mL). The combined organic layers were dried over $MgSO_4$, filtered, concentrated *in vacuo*. The residue was filtered through a silica plug, first eluting with 4:1 EtOAc/hexane to remove non-polar impurities and then 10% MeOH in EtOAc. The polar

filtrate was concentrated to afford the crude sulfonium salt. The mixture was then transferred to an oven-dried microwave vial. Potassium hydroxide (11.2 mg, 0.2 mmol) was then added to the vial before it was sealed, evacuated and back-filled with dinitrogen 3 times. Anhydrous CH_2Cl_2 (1.0 mL, 0.1 M) was then added. The sample was stirred at 40 °C for 18 h. The crude material was filtered through a syringe filter, then purified by column chromatography using the eluents given to afford the product.

Dimethyl(2-morpholino-2-oxo-1-phenylethyl)sulfonium trifluoromethanesulfonate (2a)

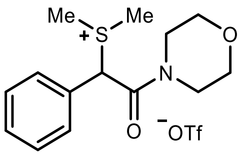

$C_{19}H_{28}F_3NO_5S_2$
MW: 471.55

Synthesised according to **GP-6** from 2-phenyl-1-morpholinoethan-1-one (205 mg, 1.0 mmol), Tf_2O (0.18 mL, 1.1 mmol), and 2-chloropyridine (0.19 mL, 2.0 mmol) in CH_2Cl_2 (3.5 mL) *then* DMSO (0.18 mL in 0.5 mL CH_2Cl_2, 2.5 mmol) and TFAA (0.14 mL, 1.0 mmol). Purification by column chromatography (silica gel; 0-10% MeOH in EtOAc) afforded the product as an off-white solid (309 mg, 0.74 mmol, 74%).

^{1}H NMR (400 MHz, $CDCl_3$): δ 7.64 – 7.58 (m, 2H, Ar *H*), 7.57 – 7.51 (m, 3H, Ar *H*), 6.76 (s, 1H, C*H*), 3.78 – 3.46 (m, 6H, 2 x CH_2, 2 x CH_{2A}), 3.22 – 3.08 (m, 5H, 2 x CH_{2B}, SCH_3), 2.70 (s, 3H, SCH_3).

^{13}C{^{1}H} NMR (101 MHz, $CDCl_3$): δ 163.5 (*C*=O), 131.9 (Ar *C*H), 130.8 (Ar *C*H), 129.7 (Ar *C*H), 127.0 (Ar *C*), 67.2 (*C*H), 66.4 (*C*H_2), 66.2 (*C*H_2), 46.6 (*C*H_2), 43.3 (*C*H_2), 26.1 (*C*H_3), 22.1 (*C*H_3). (*C*F_3 not observed but detected by ^{19}F NMR.)

^{19}F NMR (376 MHz, $CDCl_3$): δ -78.37 (s, SO_2CF_3).

HRMS: calcd. for $C_{14}H_{20}NO_2S$ [M-OTf]$^+$: 266.1209; found (ESI$^+$): 266.1207.

ν_{max} (neat/cm^{-1}): 639, 1029, 1164, 1255, 1276, 1449, 1642, 2862, 2935, 3024, 3482.

m.p.: (recrystallized from EtOAc) 138-140 °C.

2-(2-((Methylthio)methyl)phenyl)-1-morpholinoethan-1-one (3a)

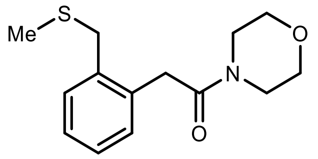

$C_{14}H_{19}NO_2S$
MW: 265.37

Synthesised according to **GP-7** from **2a** (41.5 mg, 0.1 mmol), using sodium *tert*-butoxide (19.2 mg, 0.2 mmol) in CH_2Cl_2 (1.0 mL). Purification by column chromatography (silica gel; 0-50% EtOAc in hexanes) afforded the product as an off yellow oil (26.0 mg, 0.1 mmol, 100 %).

^{1}H NMR (500 MHz, CD_3CN): δ 7.28 – 7.19 (m, 3H, Ar C*H*), 7.17 – 7.11 (m, 1H, Ar C*H*), 3.83 (s, 2H, C(O)CH_2), 3.68 (s, 2H, SCH_2), 3.63 – 3.45 (m, 8H, CH_2), 1.99 (s, 3H, SCH_3).

^{13}C{^{1}H} NMR (126 MHz, CD_3CN): δ 170.3 (*C*=O), 137.6 (Ar *C*), 135.8 (Ar *C*), 131.2 (Ar *C*H), 131.1 (Ar *C*H), 128.2 (Ar *C*H), 127.6 (Ar *C*H), 67.4 (*C*H_2), 67.2 (*C*H_2), 47.1 (*C*H_2), 42.9 (*C*H_2), 37.7 (C(O)*C*H_2), 36.5 (S*C*H_2), 15.3 (S*C*H_3).

HRMS: calcd. for $C_{14}H_{19}NO_2S$ $[M+H]^+$: 266.1209; found (ESI^+): 266.1202.

ν_{max} (neat/cm^{-1}): 698, 1078, 1299, 1542, 1699, 1704, 1755, 2893, 2911, 3028.

*The synthesis of **2a** was also carried out according to a **modified GP-4** on a 10 mmol scale as follows:*

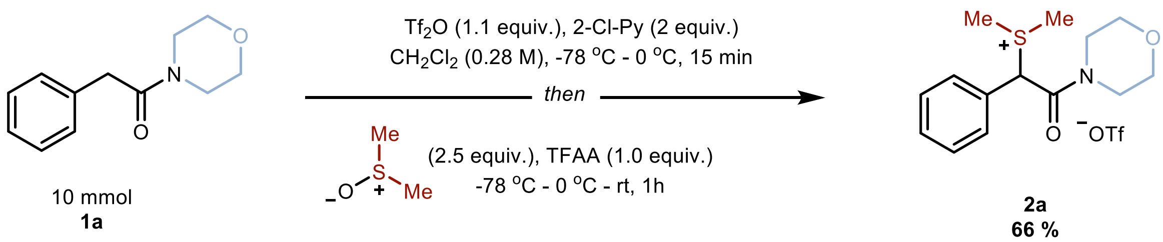

According to the modified literature procedure,[12] an oven-dried flask under an atmosphere of dinitrogen was charged with 2-phenyl-1-morpholinoethan-1-one (2.05 g, 10.0 mmol), 2-chloropyridine (1.89 mL, 20.0 mmol) and anhydrous CH_2Cl_2 (0.28 M, 35 mL). The solution was cooled to -78 °C (dry ice/acetone bath) and Tf_2O (1.85 mL, 11.0 mmol) was added drop-wise over 10 min. After complete addition, the reaction was warmed to 0 °C (ice/water bath) and stirred for 15 min. After cooling once more to -78 °C, DMSO (1.78 mL, 25.0 mmol) in anhydrous CH_2Cl_2 (5 mL) was added drop-wise, followed by drop-wise addition of TFAA (1.39 mL, 10.0 mmol). The reaction mixture was warmed to 0 °C and stirred for 15 min and at room temperature for 45 min. The reaction was poured onto a vigorously stirred saturated aqueous $NaHCO_3$ solution (50 mL) followed by vigorous stirring for 10 min. The organic layer was separated and the aqueous layer extracted with CH_2Cl_2 (3 × 50 mL). The combined organic layers were dried over $MgSO_4$, filtered, and concentrated *in vacuo*. Purification by column chromatography (silica gel; 0-5% MeOH in EtOAc) afforded the product as an off-white solid (2.76 g, 6.64 mmol, 66%).

Characterization data were identical to those listed above.

(1-(4-(*tert*-Butyl)phenyl)-2-morpholino-2-oxoethyl)dimethylsulfonium trifluoromethanesulfonate (2b)

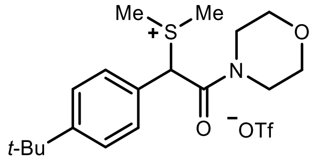

$C_{19}H_{28}F_3NO_5S_2$
MW: 471.55

Synthesised according to **GP-6** from 2-(4-(*tert*-butyl)phenyl)-1-morpholinoethan-1-one (261 mg, 1.0 mmol), Tf_2O (0.18 mL, 1.1 mmol), and 2-chloropyridine (0.19 mL, 2.0 mmol) in CH_2Cl_2 (3.5 mL) *then* DMSO (0.18 mL in 0.5 mL CH_2Cl_2, 2.5 mmol) and TFAA (0.14 mL, 1.0 mmol). Purification by column chromatography (silica gel; 0-10% MeOH in EtOAc) afforded the product as a pale yellow oil (270 mg, 0.57 mmol, 57%).

^{1}H NMR (500 MHz, CD_3CN): δ 7.63 – 7.57 (m, 2H, Ar C*H*), 7.41 – 7.36 (m, 2H, Ar C*H*), 5.99 (s, 1H, C*H*SMe$_2$), 3.65 – 3.45 (m, 5H, C*H$_2$*, C*H*H), 3.40 – 3.34 (m, 1H, CH*H*), 3.07 – 3.00 (m, 2H, C*H$_2$*), 2.81 (s, 3H, SC*H$_3$*), 2.49 (s, 3H, SC*H$_3$*), 1.32 (s, 9H, C(C*H$_3$*)$_3$).

^{13}C{^{1}H} NMR (126 MHz, CD_3CN): δ 164.6 (*C*=O), 155.9 (Ar *C*), 130.5 (Ar *C*H), 128.1 (Ar *C*H), 124.1 (Ar *C*), 121.9 (q, J = 320.6 Hz, SO$_2$*C*F$_3$), 66.8 (*C*H$_2$), 66.7 (*C*HSMe$_2$), 66.4 (*C*H$_2$), 47.2 (*C*H$_2$), 43.8 (*C*H$_2$), 35.6 (*C*(CH$_3$)$_3$), 31.3 (C(*C*H$_3$)$_3$), 25.7 (S*C*H$_3$), 23.1 (S*C*H$_3$).

^{19}F NMR (376 MHz, CD_3CN): δ -79.3 (s, SO$_2$C*F$_3$*).

HRMS: calcd. for $C_{18}H_{28}NO_2S$ [M-OTf]$^+$: 322.1835; found (ESI$^+$): 322.1849.

ν_{max} (neat/cm^{-1}): 636, 1028, 1112, 1157, 1251, 1464, 1639, 2964, 3523.

2-(4-(*tert*-Butyl)-2-((methylthio)methyl)phenyl)-1-morpholinoethan-1-one (3b)

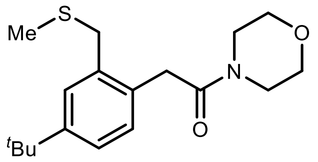

$C_{18}H_{27}NO_2S$
MW: 321.4790

Synthesised according to **GP-7** from **2b** (47.0 mg, 0.1 mmol), with sodium *tert*-butoxide (19.2 mg, 0.2 mmol) in CH_2Cl_2 (1.0 mL). Purification by column chromatography (silica gel; 0-50% EtOAc in hexanes) afforded the product as a colourless oil (22.0 mg, 0.069 mmol, 69 %).

^{1}H NMR (500 MHz, CDCl$_3$): δ 7.24 (dd, *J* = 8.0, 2.2 Hz, 1H, Ar C*H*), 7.17 (d, *J* = 2.1 Hz,1H, Ar C*H*), 7.07 (d, *J* = 8.0 Hz,1H, Ar C*H*), 3.84 (s, 2H, C(O)CH_2), 3.71 – 3.64 (m, 6H, SCH_2 + CH_2), 3.60 – 3.54 (m, 2H, CH_2), 3.48 – 3.42 (m, 2H, CH_2), 2.01 (s, 3H, SCH_3), 1.30 (s, 9H, C(CH_3)$_3$).

^{13}C{^{1}H} NMR (126 MHz, CDCl$_3$): δ 170.0 (*C*=O), 149.8 (Ar *C*), 134.9 (Ar *C*), 130.7 (Ar *C*), 129.3 (Ar *C*H), 127.8 (Ar *C*H), 124.7 (Ar *C*H), 67.0 (*C*H$_2$), 66.7 (*C*H$_2$), 46.6 (*C*H$_2$), 42.3 (*C*H$_2$), 37.1 (C(O)*C*H$_2$), 37.0 (S*C*H$_2$), 34.4 (*C*(CH$_3$)$_3$), 31.4 (C(*C*H$_3$)$_3$), 15.4 (S*C*H$_3$).

HRMS: calcd. for $C_{18}H_{28}NO_2S$ [M+H]$^+$: 322.1835; found (ESI$^+$): 322.1827.

ν_{max} (neat/cm^{-1}): 1035, 1113, 1232, 1271, 1429, 1639, 2857, 2913, 2960.

(1-(4-Methylphenyl)-2-morpholino-2-oxoethyl)dimethylsulfonium trifluoromethanesulfonate (2c)

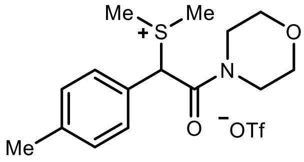

$C_{16}H_{22}F_3NO_5S_2$
MW: 429.47

Synthesised according to **GP-6** from 2-(4-methylphenyl)-1-morpholinoethan-1-one (235 mg, 1.0 mmol), Tf_2O (0.18 mL, 1.1 mmol), and 2-chloropyridine (0.19 mL, 2.0 mmol) in CH_2Cl_2 (3.5 mL) *then* DMSO (0.18 mL in 0.5 mL CH_2Cl_2, 2.5 mmol) and TFAA (0.14 mL, 1.0 mmol). Purification by column chromatography (silica gel; 0-10% MeOH in EtOAc) afforded the product as a tan solid (285 mg, 0.663 mmol, 67%).

^{1}H NMR (400 MHz, $CDCl_3$): δ 7.47 (d, *J* = 8.0 Hz, 2H, Ar *H*), 7.32 (d, *J* = 8.0 Hz, 2H, Ar *H*), 6.72 (s, 1H, C*H*SMe$_2$), 3.78 – 3.53 (m, 5H, 2 × CH_2, CH_{2A}), 3.49 (ddd, *J* = 12.8, 6.9, 3.3 Hz, 1H, CH_{2B}), 3.21 – 3.06 (m, 2H, CH_2), 3.12 (s, 3H, SCH_3), 2.68 (s, 3H, SCH_3), 2.41 (s, 3H, CH_3).

^{13}C{^{1}H} NMR (101 MHz, $CDCl_3$): δ 163.7 (*C*=O), 142.5 (Ar *C*), 131.4 (Ar *C*), 129.6 (Ar *C*H), 123.8 (Ar *C*H), 120.7 (q, *J* = 319.4 Hz, SO$_2$*C*F$_3$), 67.1 (*C*H), 66.4 (*C*H$_2$), 66.2 (*C*H$_2$), 46.6 (*C*H$_2$), 43.3 (*C*H$_2$), 25.9 (S*C*H$_3$), 22.0 (S*C*H$_3$), 21.6 (*C*H$_3$).

^{19}F NMR (376 MHz, CD_3CN): δ -78.41 (s, SO$_2$CF_3).

HRMS: calcd. for $C_{15}H_{22}NO_2S$ [M-OTf]$^+$: 280.1371; found (ESI$^+$): 280.1384.

ν_{max} (neat/cm^{-1}): 847, 1027, 1113, 1152, 1219, 1256, 1448, 1464, 1635, 2862, 2934.

m.p.: (recrystallized from EtOAc) 149 -150 °C.

2-(4-Methyl-2-((methylthio)methyl)phenyl)-1-morpholinoethan-1-one (3c)

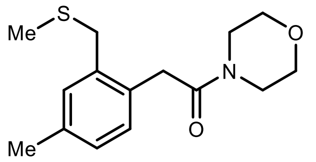

$C_{15}H_{21}NO_2S$
MW: 279.40

Synthesised according to **GP-7** from **2c** (43.0 mg, 0.1 mmol), with sodium *tert*-butoxide (19.2 mg, 0.2 mmol) in CH_2Cl_2 (1.0 mL). Purification by column chromatography (silica gel; 0-50% EtOAc in hexanes) afforded the product as a colourless oil (23.1 mg, 0.084 mmol, 84 %).

^{1}H NMR (400 MHz, $CDCl_3$): δ 7.05 – 7.02 (m, 2H, Ar C*H*), 7.00 (s, 1H, Ar C*H*), 3.83 (s, 2H, C(O)CH_2), 3.68 (s, 4H, CH_2), 3.64 (s, 2H, SCH_2), 3.59 – 3.52 (m, 2H, CH_2), 3.47 – 3.40 (m, 2H, CH_2), 2.31 (s, 3H, CH_3), 2.03 (s, 3H, SCH_3).

^{13}C{^{1}H} NMR (101 MHz, $CDCl_3$): δ 170.0 (*C*=O), 136.6 (Ar *C*), 135.3 (Ar *C*), 131.5 (Ar *C*H), 130.7 (Ar *C*), 129.4 (Ar *C*H), 128.5 (Ar *C*H), 67.0 (*C*H_2), 66.7 (*C*H_2), 46.6 (*C*H_2), 42.3 (*C*H_2), 37.2 (C(O)*C*H_2), 36.6 (S*C*H_2), 21.1 (*C*H_3), 15.4 (S*C*H_3).

HRMS: calcd. for $C_{15}H_{21}NO_2SNa$ $[M+Na]^+$: 302.1185; found (ESI^+): 302.1172.

ν_{max} (neat/cm^{-1}): 1036, 1114, 1231, 1272, 1432, 1642, 2854, 2915, 2962.

Dimethyl(2-morpholino-2-oxo-1-(*o*-tolyl)ethyl)sulfonium trifluoromethanesulfonate (2d)

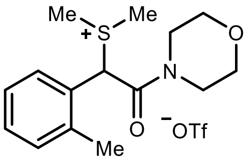

$C_{16}H_{22}F_3NO_5S_2$
MW: 429.47

Synthesised according to **GP-6** from 1-morpholino-2-(o-tolyl)ethan-1-one (219 mg, 1.0 mmol), Tf_2O (0.18 mL, 1.1 mmol), and 2-chloropyridine (0.19 mL, 2.0 mmol) in CH_2Cl_2 (3.5 mL) *then* DMSO (0.18 mL in 0.5 mL CH_2Cl_2, 2.5 mmol) and TFAA (0.14 mL, 1.0 mmol). Purification by column chromatography (silica gel; 0-10% MeOH in EtOAc) afforded the product as a pale yellow oil (223 mg, 0.520 mmol, 52%).

^{1}H NMR (500 MHz, CD_3CN): δ 7.51 – 7.44 (m, 1H, Ar C*H*), 7.43 – 7.40 (m, 1H, Ar C*H*), 7.40 – 7.36 (m, 2H, Ar C*H*), 6.11 (s, 1H, C*H*SMe_2), 3.66 – 3.44 (m, 5H, CH_2 + C*H*H), 3.25 (ddd, *J* = 13.5, 6.8, 3.1 Hz, 1H, CH*H*), 2.99 – 2.88 (m, 5H, CH_2 + SCH_3), 2.52 (s, 3H, SCH_3), 2.45 (s, 3H, CH_3).

^{13}C{^{1}H} NMR (101 MHz, CD_3CN): δ 164.9 (*C*=O), 139.3 (Ar *C*), 133.6 (Ar *C*), 132.3 (Ar *C*H), 131.0 (Ar *C*H), 128.8 (Ar *C*H), 126.3 (Ar *C*H), 122.0 (q, *J* = 320.5 Hz, SO_2CF_3), 66.8 (CH_2), 66.3 (CH_2), 65.4 (*C*HSMe_2), 47.0 (CH_2), 44.0 (CH_2), 26.9 (S*C*H_3), 22.9 (S*C*H_3), 19.9 (CH_3).

^{19}F NMR (376 MHz, CD_3CN): δ -79.30 (s, SO_2CF_3).

HRMS: calcd. for $C_{15}H_{22}NO_2S$ [M-OTf]$^+$: 280.1366; found (ESI$^+$): 280.1379.

ν_{max} (neat/cm^{-1}): 636, 1028, 1155, 1249, 1442, 1637, 2932, 3020, 3513.

2-(2-Methyl-6-((methylthio)methyl)phenyl)-1-morpholinoethan-1-one (3d)

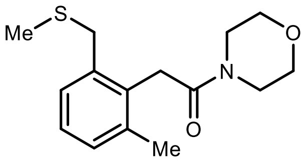

$C_{15}H_{21}NO_2S$
MW: 279.40

Synthesised according to **GP-7** from **2d** (43.0 mg, 0.1 mmol), with sodium *tert*-butoxide (19.2 mg, 0.2 mmol) in CH_2Cl_2 (1.0 mL). Purification by column chromatography (silica gel; 0-50% EtOAc in hexanes) afforded the product as a white solid (22.0 mg, 0.077 mmol, 77%).

^{1}H NMR (400 MHz, $CDCl_3$): δ 7.13 – 7.07 (m, 2H, Ar C*H*), 7.06 – 6.99 (m, 1H, Ar C*H*), 3.86 (s, 2H, C(O)CH_2), 3.78 – 3.68 (m, 4H, CH_2), 3.68 – 3.63 (m, 6H, SCH_2 + CH_2), 2.27 (s, 3H, CH_3), 2.01 (s, 3H, SCH_3).

^{13}C{^{1}H} NMR (101 MHz, $CDCl_3$): δ 169.0 (*C*=O), 137.9 (Ar *C*), 136.0 (Ar *C*), 133.1 (Ar *C*), 129.6 (Ar *C*H), 128.5 (Ar *C*H), 126.6 (Ar *C*H), 67.1 (*C*H$_2$), 66.7 (*C*H$_2$), 46.2 (*C*H$_2$), 42.4 (*C*H$_2$), 37.1 (S*C*H$_2$), 32.7 (C(O)*C*H$_2$), 20.3 (*C*H$_3$), 15.2 (S*C*H$_3$).

HRMS: calcd. for $C_{15}H_{22}NO_2S$ [M+H]$^+$: 280.1366; found (ESI$^+$): 280.1377.

ν_{max} (neat/cm^{-1}): 1033, 1113, 1229, 1269, 1427, 1640, 2853, 2918, 2961.

m.p.: 82-85 °C.

Dimethyl(2-morpholino-2-oxo-1-(*m*-tolyl)ethyl)sulfonium trifluoromethanesulfonate (2e)

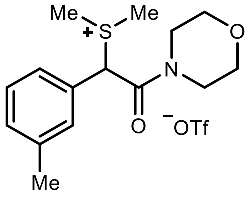

$C_{16}H_{22}F_3NO_5S_2$
MW: 429.47

Synthesised according to **GP-6** from 1-morpholino-2-(m-tolyl)ethan-1-one (219 mg, 1.0 mmol), Tf_2O (0.18 mL, 1.1 mmol), and 2-chloropyridine (0.19 mL, 2.0 mmol) in CH_2Cl_2 (3.5 mL) *then* DMSO (0.18 mL in 0.5 mL CH_2Cl_2, 2.5 mmol) and TFAA (0.14 mL, 1.0 mmol). Purification by column chromatography (silica gel; 0-10% MeOH in EtOAc) afforded the product as a pale yellow oil (320 mg, 0.750 mmol, 75%).

^{1}H NMR (500 MHz, CD_3CN): δ 7.47 – 7.39 (m, 2H, Ar C*H*), 7.29 – 7.23 (m, 2H, Ar C*H*), 5.95 (s, 1H, C*H*SMe$_2$), 3.65 – 3.46 (m, 5H, C*H$_2$* + C*H*H), 3.40 – 3.33 (m, 1H, CH*H*), 3.08 – 3.01 (m, 2H, C*H$_2$*), 2.82 (s, 3H, SC*H$_3$*), 2.49 (s, 3H, SC*H$_3$*), 2.39 (s, 3H, C*H$_3$*).

^{13}C{^{1}H} NMR (101 MHz, CD_3CN): δ 164.5 (*C*=O), 141.6 (Ar *C*), 133.1 (Ar *C*H), 131.3 (Ar *C*H), 131.0 (Ar *C*H), 127.9 (Ar *C*H), 127.0 (Ar *C*), 122.1 (q, *J* = 320.5 Hz, SO$_2$*C*F$_3$), 67.1 (*C*HSMe$_2$), 66.8 (*C*H$_2$), 66.4 (*C*H$_2$), 47.2 (*C*H$_2$), 43.8 (*C*H$_2$), 25.9 (S*C*H$_3$), 23.2 (S*C*H$_3$), 21.4 (*C*H$_3$).

^{19}F NMR (376 MHz, CD_3CN): δ -79.30 (s, SO$_2$C*F$_3$*).

HRMS: calcd. for $C_{15}H_{22}NO_2S$ [M-OTf]$^+$: 280.1366; found (ESI$^+$): 280.1379.

$ν_{max}$ (neat/cm^{-1}): 635, 1027, 1155, 1248, 1446, 1638, 2932, 3020, 3517.

2-(3-Methyl-2-((methylthio)methyl)phenyl)-1-morpholinoethan-1-one (3e)

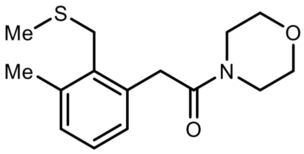

$C_{15}H_{21}NO_2S$
MW: 279.40

Synthesised according to **GP-7** from **2e** (43.0 mg, 0.1 mmol), with sodium *tert*-butoxide (19.2 mg, 0.2 mmol) in CH_2Cl_2 (1.0 mL). Purification by column chromatography (silica gel; 0-50% EtOAc in hexanes) afforded the combined products (**3e** & **3e'**) as a colourless oil (23.3 mg, 0.084 mmol, 84 %) (in a 1:1 ratio). The mixture was repurified by column chromatography (silica gel; 0-30% EtOAc in hexanes) afforded the isolated products **3e** and **3e'**.

^{1}H NMR (500 MHz, $CDCl_3$): δ 7.15 – 7.05 (m, 2H, Ar C*H*), 7.01 – 6.96 (m, 1H, Ar C*H*), 3.91 (s, 2H, C(O)CH_2), 3.74 (s, 2H, SCH_2), 3.72 – 3.66 (m, 4H, CH_2), 3.59 – 3.53 (m, 2H, CH_2), 3.47 – 3.42 (m, 2H, CH_2), 2.42 (s, 3H, CH_3), 2.15 (s, 3H, SCH_3).

^{13}C{^{1}H} NMR (126 MHz, $CDCl_3$): δ 170.2 (*C*=O), 137.8 (Ar *C*), 134.3 (Ar *C*), 134.0 (Ar *C*), 129.6 (Ar *C*H), 127.6 (Ar *C*H), 127.0 (Ar *C*H), 67.0 (*C*H_2), 66.8 (*C*H_2), 46.7 (*C*H_2), 42.3 (*C*H_2), 38.2 (C(O)*C*H_2), 33.0 (S*C*H_2), 20.1 (*C*H_3), 16.4 (S*C*H_3).

HRMS: calcd. for $C_{15}H_{21}NO_2SNa$ $[M+Na]^+$: 302.1185; found (ESI$^+$): 302.1172.

ν_{max} (neat/cm^{-1}): 1031, 1113, 1226, 1271, 1431, 1638, 2854, 2916, 2963.

2-(5-Methyl-2-((methylthio)methyl)phenyl)-1-morpholinoethan-1-one (3e')

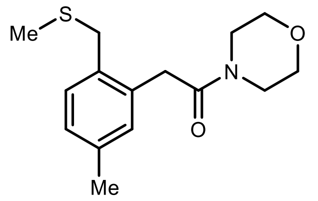

$C_{15}H_{21}NO_2S$
MW: 279.40

^{1}H NMR (500 MHz, $CDCl_3$): δ 7.07 (d, *J* = 7.6 Hz, 1H, Ar C*H*), 7.00 (dd, *J* = 7.7, 1.9 Hz, 1H, Ar C*H*), 6.97 (d, *J* = 1.8 Hz, 1H, Ar C*H*), 3.85 (s, 2H, C(O)C*H₂*), 3.69 (br., 4H, C*H₂*), 3.64 (s, 2H, SC*H₂*), 3.59 – 3.54 (m, 2H, C*H₂*), 3.49 – 3.43 (m, 2H, C*H₂*), 2.31 (s, 3H, C*H₃*), 2.01 (s, 3H, SC*H₃*).

^{13}C{^{1}H} NMR (126 MHz, $CDCl_3$): δ 170.0 (*C*=O), 137.6 (Ar *C*), 133.6 (Ar *C*), 132.4 (Ar *C*), 130.7 (Ar *C*H), 130.4 (Ar *C*H), 127.7 (Ar *C*H), 67.1 (*C*H₂), 66.8 (*C*H₂), 46.6 (*C*H₂), 42.3 (*C*H₂), 37.5 (C(O)*C*H₂), 36.3 (S*C*H₂), 21.2 (*C*H₃), 15.3 (S*C*H₃).

HRMS: calcd. for $C_{15}H_{21}NO_2SNa$ $[M+Na]^+$: 302.1185; found (ESI$^+$): 302.1172.

ν_{max} (neat/cm^{-1}): 1031, 1113, 1226, 1271, 1431, 1638, 2854, 2916, 2963.

(1-(3-Methoxyphenyl)-2-morpholino-2-oxoethyl)dimethylsulfonium trifluoromethanesulfonate (2f)

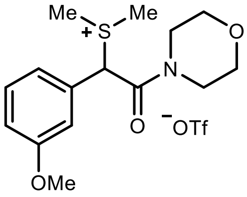

$C_{16}H_{22}F_3NO_6S_2$
MW: 445.47

Synthesised according to **GP-6** from 2-(3-methoxyphenyl)-1-morpholinoethan-1-one (235 mg, 1.0 mmol), Tf_2O (0.18 mL, 1.1 mmol), and 2-chloropyridine (0.19 mL, 2.0 mmol) in CH_2Cl_2 (3.5 mL) *then* DMSO (0.18 mL in 0.5 mL CH_2Cl_2, 2.5 mmol) and TFAA (0.14 mL, 1.0 mmol). Purification by column chromatography (silica gel; 0-10% MeOH in EtOAc) afforded the product as a pale yellow oil (191 mg, 0.430 mmol, 43%).

^{1}H NMR (400 MHz, CD_3CN): δ 7.48 (dd, *J* = 8.4, 7.6 Hz, 1H, Ar C*H*), 7.14 (ddd, *J* = 8.4, 2.6, 0.9 Hz, 1H, Ar C*H*), 7.01 (ddd, *J* = 7.6, 1.8, 0.9 Hz, 1H, Ar C*H*), 6.98 (t, *J* = 2.2 Hz, 1H, Ar C*H*), 5.90 (s, 1H, C*H*SMe_2), 3.83 (s, 3H, OCH_3), 3.67 – 3.59 (m, 1H, C*H*H), 3.59 – 3.47 (m, 4H, CH_2), 3.41 – 3.33 (m, 1H, CH*H*), 3.12 – 3.04 (m, 2H, CH_2), 2.82 (s, 3H, SCH_3), 2.51 (s, 3H, SCH_3).

^{13}C{^{1}H} NMR (101 MHz, CD_3CN): δ 164.4 (*C*=O), 161.8 (Ar *C*), 132.4 (Ar *C*H), 128.6 (Ar *C*), 122.7 (Ar *C*H), 122.0 (q, *J* = 321.2 Hz, SO_2CF_3), 118.1 (Ar *C*H), 116.0 (Ar *C*H), 67.0 (*C*HSMe_2), 66.8 (*C*H_2), 66.5 (*C*H_2), 56.3 (O*C*H_3), 47.2 (*C*H_2), 43.8 (*C*H_2), 26.0 (S*C*H_3), 23.3 (S*C*H_3).

^{19}F NMR (376 MHz, CD_3CN): δ -79.30 (s, SO_2CF_3).

HRMS: calcd. for $C_{15}H_{22}NO_3S$ [M-OTf]$^+$: 296.1315; found (ESI$^+$): 296.1330.

ν_{max} (neat/cm^{-1}): 637, 1028, 1157, 1249, 1439, 1639, 2934, 3018, 3497.

2-(3-Methoxy-2-((methylthio)methyl)phenyl)-1-morpholinoethan-1-one (3f)

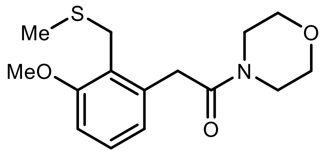

$C_{15}H_{21}NO_3S$
MW: 295.40

Synthesised according to **GP-7** from **2f** (44.5 mg, 0.1 mmol), with sodium *tert*-butoxide (19.2 mg, 0.2 mmol) in CH_2Cl_2 (1.0 mL). Purification by column chromatography (silica gel; 0-50% EtOAc in hexanes) afforded the combined product (**3f** & **3f'**) as a colourless oil (27.3 mg, 0.092 mmol, 92 %) (in 1:1 ratio). The mixture was repurified by column chromatography (silica gel; 0-30% EtOAc in hexanes) afforded the isolated products **3f** and **3f'**.

^{1}H NMR (400 MHz, $CDCl_3$): δ 7.19 (t, *J* = 8.0 Hz, 1H, Ar C*H*), 6.83 – 6.75 (m, 2H, Ar C*H*), 3.88 (s, 2H, C(O)CH_2), 3.82 (s, 3H, OCH_3), 3.79 (s, 2H, SCH_2), 3.68 (br., 4H, CH_2), 3.58 – 3.52 (m, 2H, CH_2), 3.47 – 3.40 (m, 2H, CH_2), 2.07 (s, 3H, SCH_3).

^{13}C{^{1}H} NMR (101 MHz, $CDCl_3$): δ 169.9 (*C*=O), 157.9 (Ar *C*), 135.5 (Ar *C*), 128.3 (Ar *C*H), 124.6 (Ar *C*), 121.4 (Ar *C*H), 109.5 (Ar *C*H), 67.0 (*C*H_2), 66.7 (*C*H_2), 55.8 (O*C*H_3), 46.6 (*C*H_2), 42.3 (*C*H_2), 37.8 (C(O)*C*H_2), 28.3 (S*C*H_2), 15.6 (S*C*H_3).

HRMS: calcd. for $C_{15}H_{21}NO_3SNa$ $[M+Na]^+$: 318.1134; found (ESI^+): 318.1123.

ν_{max} (neat/cm^{-1}): 1038, 1114, 1257, 1433, 1608, 1644, 2854, 2915, 2961.

2-(5-Methoxy-2-((methylthio)methyl)phenyl)-1-morpholinoethan-1-one (3f’)

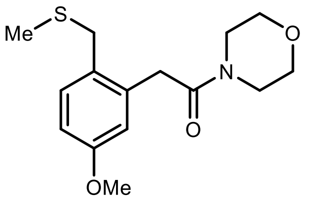

$C_{15}H_{21}NO_3S$
MW: 295.40

^{1}H NMR (400 MHz, CDCl$_3$): δ 7.13 – 7.07 (m, 1H, Ar C*H*), 6.76 – 6.70 (m, 2H, Ar C*H*), 3.86 (s, 2H, C(O)C*H$_2$*), 3.78 (s, 3H, OC*H$_3$*), 3.69 (br., 4H, C*H$_2$*), 3.63 (s, 2H, SC*H$_2$*), 3.61 – 3.54 (m, 2H, C*H$_2$*), 3.50 – 3.43 (m, 2H, C*H$_2$*), 2.01 (s, 3H, SC*H$_3$*).

^{13}C{^{1}H} NMR (101 MHz, CDCl$_3$): δ 169.7 (*C*=O), 159.2 (Ar *C*), 135.2 (Ar *C*), 131.8 (Ar *C*H), 127.5 (Ar *C*), 115.6 (Ar *C*H), 111.9 (Ar *C*H), 67.1 (*C*H$_2$), 66.8 (*C*H$_2$), 55.4 (O*C*H$_3$), 46.6 (*C*H$_2$), 42.4 (*C*H$_2$), 37.7 (C(O)*C*H$_2$), 36.1 (S*C*H$_2$), 15.3 (S*C*H$_3$).

HRMS: calcd. for $C_{15}H_{21}NO_3SNa$ $[M+Na]^+$: 318.1134; found (ESI$^+$): 318.1123.

ν_{max} (neat/cm^{-1}): 1038, 1114, 1257, 1433, 1608, 1644, 2854, 2915, 2961.

(1-(2-Bromo-4-methoxyphenyl)-2-morpholino-2-oxoethyl)dimethylsulfonium trifluoromethanesulfonate (2g)

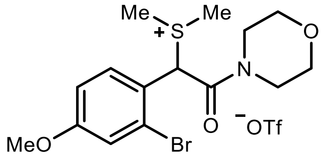

$C_{16}H_{21}BrF_3NO_6S_2$
MW: 524.36

Synthesised according to **GP-6** 2-(2-bromo-4-methoxyphenyl)-1-morpholinoethan-1-one (314 mg, 1.0 mmol), Tf_2O (0.18 mL, 1.1 mmol), and 2-chloropyridine (0.19 mL, 2.0 mmol) in CH_2Cl_2 (3.5 mL) *then* DMSO (0.18 mL in 0.5 mL CH_2Cl_2, 2.5 mmol) and TFAA (0.14 mL, 1.0 mmol). Purification by column chromatography (silica gel; 0-10% MeOH in EtOAc) afforded the product as a pale yellow oil (356 mg, 0.680 mmol, 68%).

^{1}H NMR (500 MHz, CD_3CN): δ 7.42 – 7.38 (m, 2H, Ar C*H*), 7.11 (dd, *J* = 8.8, 2.6 Hz, 1 H, Ar C*H*), 6.26 (s, 1H, C*H*SMe$_2$), 3.85 (s, 3H, OC*H$_3$*), 3.66 – 3.47 (m, 5H, C*H$_2$* + C*H*H), 3.36 – 3.28 (m, 1 H, CH*H*), 3.02 (dddd, *J* = 11.1, 9.1, 6.5, 3.1 Hz, 2H, C*H$_2$*), 2.90 (s, 3 H, SC*H$_3$*), 2.62 (s, 3H, C*H$_3$*).

^{13}C{^{1}H} NMR (126 MHz, CD_3CN): δ 164.5 (*C*=O), 163.4 (Ar *C*), 133.1 (Ar *C*H), 126.9 (Ar *C*), 120.8 (Ar *C*H), 118.7 (Ar *C*), 116.7 (Ar *C*H), 66.8 (*C*H$_2$), 66.4 (*C*H$_2$), 65.9 (*C*HSMe$_2$), 57.0 (O*C*H$_3$), 47.1 (*C*H$_2$), 44.1 (*C*H$_2$), 26.3 (S*C*H$_3$), 23.0 (S*C*H$_3$). (*C*F$_3$ not observed but detected by ^{19}F NMR.)

^{19}F NMR (376 MHz, CD_3CN): δ -79.40 (s, SO$_2$C*F$_3$*).

HRMS: calcd. for $C_{15}H_{21}BrNO_3S$ [M-OTf]$^+$: 374.0420; found (ESI$^+$): 374.0424.

ν_{max} (neat/cm^{-1}): 638, 1029, 1274, 1494, 1601, 1643, 2859, 2929, 3364.

2-(2-Bromo-4-methoxy-6-((methylthio)methyl)phenyl)-1-morpholinoethan-1-one (3g)

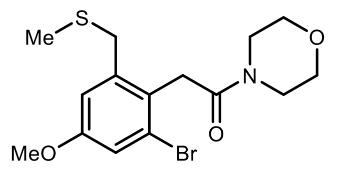

$C_{15}H_{20}BrNO_3S$
MW: 374.29

Synthesised according to **GP-7** from **2g** (52.4 mg, 0.1 mmol), with sodium *tert*-butoxide (19.2 mg, 0.2 mmol) in CH_2Cl_2 (1.0 mL). Purification by column chromatography (silica gel; 0-50% EtOAc in hexanes) afforded the product as a colourless oil (22.4 mg, 0.060 mmol, 60%).

^{1}H NMR (500 MHz, CDCl$_3$): δ 7.04 (d, J = 2.7 Hz, 1H, Ar C*H*), 6.75 (d, J = 2.7 Hz, 1H, Ar C*H*), 3.97 (s, 2H, C(O)C*H$_2$*), 3.79 – 3.73 (m, 5H, OC*H$_3$* + C*H$_2$*), 3.72 – 3.68 (m, 2H, C*H$_2$*), 3.68 – 3.62 (m, 6H, SC*H$_2$* + C*H$_2$*), 2.02 (s, 3H, SC*H$_3$*).

^{13}C{^{1}H} NMR (126 MHz, CDCl$_3$): δ 168.5 (*C*=O), 158.5 (Ar *C*), 139.4 (Ar *C*), 126.9 (Ar *C*), 126.6 (Ar *C*), 116.6 (Ar *C*H), 116.4 (Ar CH), 67.1 (*C*H$_2$), 66.8 (*C*H$_2$), 55.6 (O*C*H$_3$), 46.4 (*C*H$_2$), 42.5 (*C*H$_2$), 37.8 (S*C*H$_2$), 35.4 (C(O)*C*H$_2$), 15.3 (S*C*H$_3$).

HRMS: calcd. for $C_{15}H_{21}BrNO_3S$ $[M+H]^+$: 374.0420; found (ESI$^+$): 374.0412.

ν_{max} (neat/cm^{-1}): 1037, 1114, 1230, 1271, 1435, 1648, 2853, 2917, 2961.

(1-(4-Bromophenyl)-2-morpholino-2-oxoethyl)dimethylsulfonium trifluoromethanesulfonate (2h)

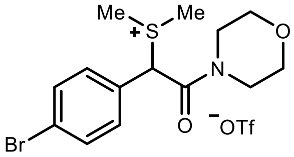

$C_{15}H_{19}BrF_3NO_5S_2$
MW: 494.34

Synthesised according to **GP-6** from 2-(4-bromophenyl)-1-morpholinoethan-1-one (284 mg, 1.0 mmol), Tf_2O (0.18 mL, 1.1 mmol), and 2-chloropyridine (0.19 mL, 2.0 mmol) in CH_2Cl_2 (3.5 mL) *then* DMSO (0.18 mL in 0.5 mL CH_2Cl_2, 2.5 mmol) and TFAA (0.14 mL, 1.0 mmol). Purification by column chromatography (silica gel; 0-10% MeOH in EtOAc) afforded the product as a yellow solid (430 mg, 0.87 mmol, 87%)

^{1}H NMR (400 MHz, $CDCl_3$): δ 7.78 – 7.70 (m, 2H, Ar *H*), 7.42 – 7.33 (m, 2H, Ar *H*), 6.02 – 5.83 (m, 1H, C*H*SMe$_2$), 3.67 – 3.46 (m, 5H, 2 × CH_2, CH_{2A}), 3.38 – 3.28 (m, 1H, CH_{2B}), 3.16 – 2.98 (m, 2H, CH_2), 2.80 (s, 3H, SCH_3), 2.52 (s, 3H, SCH_3).

^{13}C{^{1}H} NMR (101 MHz, $CDCl_3$): δ 164.1 (*C*=O), 134.4 (Ar *C*), 132.7 (Ar *C*H), 126.5 (Ar *C*H), 126.4 (Ar *C*), 66.8 (*C*HSMe$_2$), 66.4 (*C*H$_2$), 66.1 (*C*H$_2$), 47.2 (*C*H$_2$), 43.8 (*C*H$_2$), 25.8 (S*C*H$_3$), 23.2 (S*C*H$_3$). (*C*F$_3$ not observed but detected by ^{19}F NMR.)

^{19}F NMR (376 MHz, $CDCl_3$): δ -79.30 (s, SO$_2$C*F$_3$*).

HRMS: calcd. for $C_{14}H_{19}BrNO_2S$ [M-OTf]$^+$: 344.0314; found (ESI$^+$): 344.0309.

ν_{max} (neat/cm^{-1}): 677, 1051, 1239, 1476, 1688, 1723, 2891, 2951, 3577.

m.p.: (recrystallized from EtOAc) 135-138 °C.

2-(4-Bromo-2-((methylthio)methyl)phenyl)-1-morpholinoethan-1-one (3h)

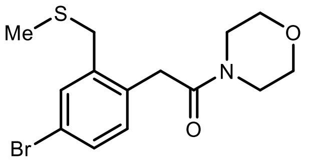

$C_{14}H_{18}BrNO_2S$
MW: 344.27

Synthesised according to **GP-7** from **2h** (49.4 mg, 0.1 mmol), with sodium *tert*-butoxide (19.2 mg, 0.2 mmol) in CH_2Cl_2 (1.0 mL). Purification by column chromatography (silica gel; 0-50% EtOAc in hexanes) afforded the product as a yellow solid (33.7 mg, 0.098 mmol, 98%).

^{1}H NMR (500 MHz, CDCl$_3$): δ 7.39 – 7.32 (m, 2H, Ar C*H*), 7.04 (d, *J* = 8.1 Hz, 1 H, Ar C*H*), 3.81 (s, 2H, C(O)CH_2), 3.71 – 3.64 (m, 4H, CH_2), 3.63 – 3.59 (m, 4H, SCH_2 + CH_2), 3.49 – 3.45 (m, 2H, CH_2), 2.03 (s, 3H, SCH_3).

^{13}C{^{1}H} NMR (126 MHz, CDCl$_3$): δ 169.2 (*C*=O), 138.1 (Ar *C*), 133.3 (Ar *C*H), 133.0 (Ar *C*), 131.6 (Ar *C*H), 130.8 (Ar *C*H), 120.8 (Ar *C*), 67.0 (*C*H$_2$), 66.7 (*C*H$_2$), 46.5 (*C*H$_2$), 42.4 (*C*H$_2$), 37.0 (C(O)*C*H$_2$), 36.2 (S*C*H$_2$), 15.4 (S*C*H$_3$).

HRMS: calcd. for $C_{14}H_{19}BrNO_2S$ $[M+H]^+$: 344.0314; found (ESI$^+$): 344.0307.

ν_{max} (neat/cm^{-1}): 1035, 1114, 1231, 1272, 1432, 1643, 2854, 2916, 2963.

m.p.: 110-113 °C.

(1-(4-Fluorophenyl)-2-morpholino-2-oxoethyl)dimethylsulfonium trifluoromethanesulfonate (2i)

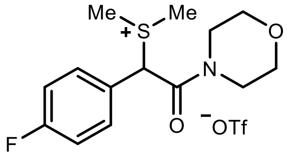

$C_{15}H_{19}F_4NO_5S_2$
MW: 433.43

Synthesised according to **GP-6** from 2-(4-fluorophenyl)-1-morpholinoethan-1-one (223 mg, 1.0 mmol), Tf_2O (0.18 mL, 1.1 mmol), and 2-chloropyridine (0.19 mL, 2.0 mmol) in CH_2Cl_2 (3.5 mL) *then* DMSO (0.18 mL in 0.5 mL CH_2Cl_2, 2.5 mmol) and TFAA (0.14 mL, 1.0 mmol). Purification by column chromatography (silica gel; 0-10% MeOH in EtOAc) afforded the product as a white solid (271 mg, 0.63 mmol, 63%).

^{1}H NMR (400 MHz, $CDCl_3$): δ 7.69 – 7.63 (m, 2H, Ar *H*), 7.23 (dd, *J* = 9.0, 8.1 Hz, 2H, Ar *H*), 6.84 (s, 1H, C*H*SMe$_2$), 3.77 (ddd, *J* = 13.3, 5.8, 3.2 Hz, 1H, C*H$_2$*), 3.70 – 3.57 (m, 4H, C*H$_2$*), 3.47 (ddd, *J* = 13.3, 7.3, 3.4 Hz, 1H, C*H$_2$*), 3.25 (ddd, *J* = 12.5, 6.0, 3.2 Hz, 1H, C*H$_2$*), 3.16 (s, 3H, SC*H$_3$*), 3.11 (ddd, *J* = 13.3, 7.6, 3.2 Hz, 1H, C*H$_2$*), 2.72 (s, 3H, SC*H$_3$*).

^{13}C{^{1}H} NMR (101 MHz, $CDCl_3$): δ 164.5 (d, *J* = 254.3 Hz, Ar *C*F), 164.3 (*C*=O), 131.9 (d, *J* = 8.7 Hz, Ar *C*H), 122.9 (d, *J* = 3.4 Hz, Ar *C*), 120.6 (q, *J* = 319.4 Hz, SO$_2$*C*F$_3$), 118.1 (d, *J* = 22.1 Hz, Ar *C*H), 66.4 (*C*H$_2$), 66.2 (*C*H$_2$), 66.1 (*C*HSMe$_2$), 46.5 (*C*H$_2$), 43.3 (*C*H$_2$), 25.9 (S*C*H$_3$), 21.9 (S*C*H$_3$).

^{19}F NMR (376 MHz, $CDCl_3$): δ -78.41 (s, 3F, O$_3$SC*F$_3$*), -106.22 (tt, *J* = 8.4, 4.4 Hz, 1F, Ar*F*).

HRMS: calcd. for $C_{14}H_{19}FNO_2S$ [M-OTf]$^+$: 284.1121; found (ESI$^+$): 284.1134.

ν_{max} (neat/cm^{-1}): 854, 1028, 1111, 1151, 1230, 1256, 1446, 1465, 1508, 1632, 3025.

m.p.: (recrystallized from EtOAc) 158-160 °C.

2-(4-Fluoro-2-((methylthio)methyl)phenyl)-1-morpholinoethan-1-one (3i)

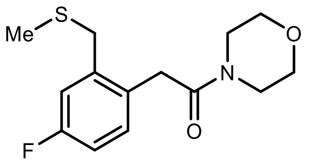

$C_{14}H_{18}FNO_2S$
MW: 283.36

Synthesised according to **GP-7** from **2i** (43.3 mg, 0.1 mmol), with sodium *tert*-butoxide (19.2 mg, 0.2 mmol) in CH_2Cl_2 (1.0 mL). Purification by column chromatography (silica gel; 0-50% EtOAc in hexanes) afforded the product as an off yellow oil (25.7 mg, 0.091 mmol, 91%).

^{1}H NMR (400 MHz, CDCl$_3$): δ 7.15 – 7.07 (m, 1H, Ar *H*), 6.97 – 6.88 (m, 2H, Ar *H*), 3.81 (s, 2H, C*H*$_2$C(O)), 3.74 – 3.30 (m, 10H, SC*H*$_2$ + C*H*$_2$), 2.02 (s, 3H, C*H*$_3$).

^{13}C{^{1}H} NMR (101 MHz, CDCl$_3$): δ 169.5 (*C*=O), 161.6 (d, *J* = 245.9 Hz, Ar *C*F), 138.1 (d, *J* = 7.0 Hz, Ar *C*), 131.4 (d, *J* = 8.1 Hz, Ar *C*H), 129.6 (d, *J* = 3.2 Hz, Ar *C*), 117.3 (d, *J* = 21.7 Hz, Ar *C*H), 114.4 (d, *J* = 21.0 Hz, Ar *C*H), 67.0 (*C*H$_2$), 66.7 (*C*H$_2$), 46.5 (*C*H$_2$), 42.3 (*C*H$_2$), 36.7 (*C*H$_2$C(O)), 36.3 (S*C*H$_2$), 15.3 (*C*H$_3$).

^{19}F NMR (376 MHz, CDCl$_3$): δ -115.93 (td, *J* = 8.8, 5.7 Hz, F, C*F*).

HRMS: calcd. for $C_{14}H_{19}FNO_2S$ [M+H]$^+$: 284.1115; found (ESI$^+$): 284.1108.

ν_{max} (neat/cm^{-1}): 678, 1036, 1248, 1433, 1498, 1640, 2852, 2919.

(1-(4-Trifluoromethylphenyl)-2-morpholino-2-oxoethyl)dimethylsulfonium trifluoromethanesulfonate (2j)

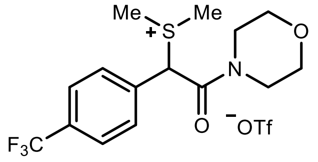

$C_{16}H_{19}F_6NO_5S_2$
MW: 483.44

Synthesised according to **GP-6** from 2-(4-trifluoromethylphenyl)-1-morpholinoethan-1-one (273 mg, 1.0 mmol), Tf_2O (0.18 mL, 1.1 mmol), and 2-chloropyridine (0.19 mL, 2.0 mmol) in CH_2Cl_2 (3.5 mL) *then* DMSO (0.18 mL in 0.5 mL CH_2Cl_2, 2.5 mmol) and TFAA (0.14 mL, 1.0 mmol). Purification by column chromatography (silica gel; 10% MeOH in EtOAc) afforded the product as an off-white solid (367 mg, 0.76 mmol, 76%).

^{1}H NMR (400 MHz, CD_3CN): δ 7.88 (d, *J* = 8.1 Hz, 2H, Ar *H*), 7.66 (d, *J* = 8.1 Hz, 2H, Ar *H*), 6.03 (s, 1H, C*H*SMe$_2$), 3.67 – 3.47 (m, 5H, 2 x C*H$_2$*, 1 x C*H*$_{2A}$), 3.39 – 3.31 (m, 1H, C*H*$_{2A}$), 3.12 – 3.06 (m, 1H, C*H*$_{2B}$), 3.05 – 2.98 (m, 1H, C*H*$_{2B}$) 2.84 (s, 3H, C*H*$_3$), 2.53 (s, 3H, C*H*$_3$).

^{13}C{^{1}H} NMR (101 MHz, CD_3CN): δ 164.0 (*C*=O), 133.4 (q, *J* = 32.8 Hz, Ar *C*CF$_3$), 131.8 (Ar *C*H), 131.5 (q, J = 1.3 Hz, Ar *C*), 128.2 (q, *J* = 3.8 Hz, Ar *C*H), 124.8 (q, *J* = 271.9 Hz, *C*F$_3$), 122.1 (q, *J* = 320.7 Hz, SO$_2$*C*F$_3$), 66.8 (*C*H$_2$), 66.4 (*C*H$_2$), 65.8 (*C*HSMe$_2$), 47.2 (*C*H$_2$), 43.9 (*C*H$_2$), 25.9 (S*C*H$_3$), 23.3 (S*C*H$_3$).

^{19}F NMR (376 MHz, CD_3CN): δ -63.57 (s, 3F, C*F$_3$*), -79.32 (s, 3F, SO$_2$C*F$_3$*).

HRMS: calcd. for $C_{15}H_{19}F_3NO_2S$ [M-OTf]$^+$: 334.1083; found (ESI$^+$): 334.1092.

ν_{max} (neat/cm^{-1}): 639, 857, 1030, 1164, 1253, 1447, 1643, 1721, 2861, 2935, 3515.

m.p.: (recrystallized from EtOAc) 186–190 °C.

2-(2-((Methylthio)methyl)-4-(trifluoromethyl)phenyl)-1-morpholinoethan-1-one (3j)

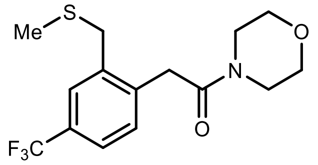

$C_{15}H_{18}F_3NO_2S$
MW: 333.37

Synthesised according to **GP-7** from **2j** (48.3 mg, 0.1 mmol), with sodium *tert*-butoxide (19.2 mg, 0.2 mmol) in CH_2Cl_2 (1.0 mL). Purification by column chromatography (silica gel; 0-50% EtOAc in hexanes) afforded the product as a white solid (28.0 mg, 0.084 mmol, 84%).

^{1}H NMR (400 MHz, $CDCl_3$): δ 7.50 (dd, *J* = 8.0, 1.9 Hz, 1H, Ar C*H*), 7.45 (d, *J* = 2.0 Hz, 1H, Ar C*H*), 7.29 (d, *J* = 8.0 Hz, 1H, Ar C*H*), 3.93 (s, 2H, C(O)CH_2), 3.73 – 3.66 (m, 6H, SCH_2 + CH_2), 3.66 – 3.61 (m, 2H, CH_2), 3.52 – 3.47 (m, 2H, CH_2), 2.03 (s, 3H, SCH_3).

^{13}C{^{1}H} NMR (101 MHz, $CDCl_3$): δ 168.9 (*C*=O), 138.2 (Ar *C*), 136.9 (Ar *C*), 131.3 (Ar *C*H), 129.4 (q, *J* = 32.6 Hz, Ar *C*), 127.1 (q, *J* = 3.7 Hz, Ar *C*H), 124.6 (q, *J* = 3.8 Hz, Ar *C*H), 124.1 (q, *J* = 272.0 Hz, *C*F_3), 67.0 (*C*H_2), 66.7 (*C*H_2), 46.5 (*C*H_2), 42.4 (*C*H_2), 37.2 (C(O)*C*H_2), 36.4 (S*C*H_2), 15.4 (S*C*H_3).

^{19}F NMR (376 MHz, $CDCl_3$): δ -62.5 (s).

HRMS: calcd. for $C_{15}H_{19}F_3NO_2S$ $[M+H]^+$: 334.1083; found (ESI$^+$): 334.1073.

ν_{max} (neat/cm^{-1}): 1113, 1159, 1236, 1332, 1423. 1648, 2861, 2923, 2968.

m.p.: 115-118 °C.

(1-(Naphthalen-2-yl)-2-morpholino-2-oxoethyl)dimethylsulfonium trifluoromethanesulfonate (2k)

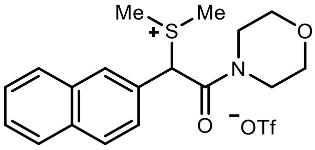

$C_{19}H_{22}F_3NO_5S_2$
MW: 465.50

Synthesised according to **GP-6** from 2-(naphthalen-2-yl)-1-morpholinoethan-1-one (95 mg, 0.37 mmol), Tf_2O (68 μL, 1.1 mmol), and 2-chloropyridine (70 μL, 2.0 mmol) in CH_2Cl_2 (1.3 mL) *then* DMSO (66 μL in 0.2 mL CH_2Cl_2, 0.93 mmol) and TFAA (0.14 mL, 1.0 mmol). Purification by column chromatography (silica gel; 0-10% MeOH in EtOAc) afforded the product as a pale yellow oil (102 mg, 0.22 mmol, 60%).

^{1}H NMR (400 MHz, CD_3CN): δ 8.09 (d, *J* = 8.5 Hz, 1H, Ar *H*), 8.05 – 7.98 (m, 3H, Ar *H*), 7.70 – 7.64 (m, 2H, Ar *H*), 7.48 (dd, *J* = 8.5, 2.0 Hz, 1H, Ar *H*), 6.05 (s, 1H, C*H*SMe$_2$), 3.67 – 3.49 (m, 4H, 2 x CH_2), 3.48 – 3.34 (m, 2H, 2 x CH_{2A}) 3.09 – 2.96 (m, 2H, 2 x CH_{2B}), 2.84 (s, 3H, SCH_3), 2.52 (s, 3H, SCH_3).

^{13}C{^{1}H} NMR (101 MHz, CD_3CN) δ 164.5 (*C*=O), 135.2 (Ar *C*), 134.2 (Ar *C*), 131.8 (Ar *C*H), 131.3 (Ar *C*H), 129.6 (Ar *C*H), 129.3 (Ar *C*H), 128.9 (Ar *C*H), 128.6 (Ar *C*H), 126.5 (Ar *C*H), 124.4 (Ar *C*), 122.0 (q, *J* = 320.3 Hz, *C*F$_3$), 67.4 (*C*HSMe$_2$), 66.9 (*C*H$_2$), 66.4 (*C*H$_2$), 47.3 (*C*H$_2$), 43.8 (*C*H$_2$), 25.9 (S*C*H$_3$), 23.4 (S*C*H$_3$).

^{19}F NMR (376 MHz, CD_3CN): -79.33 (s, SO$_2$CF_3).

HRMS: calcd. for $C_{18}H_{22}NO_2S$ [M-OTf]$^+$: 316.1362; found (ESI$^+$): 316.1366.

ν_{max} (neat/cm^{-1}): 639, 750, 1031, 1258, 1443, 1643, 2864, 2928, 3354.

2-(1-((Methylthio)methyl)naphthalen-2-yl)-1-morpholinoethan-1-one (3k)

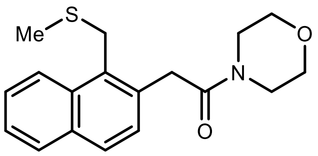

$C_{18}H_{21}NO_2S$
MW: 315.43

Synthesised according to **GP-7** from **2k** (46.5 mg, 0.1 mmol), with sodium *tert*-butoxide (19.2 mg, 0.2 mmol) in CH_2Cl_2 (1.0 mL). Purification by column chromatography (silica gel; 0-50% EtOAc in hexanes) afforded the product as a white solid (27.1 mg, 0.086 mmol, 86%).

^{1}H NMR (500 MHz, $CDCl_3$): δ 8.15 (d, *J* = 8.5 Hz, 1H, Ar C*H*), 7.82 (dd, *J* = 8.2, 1.4 Hz, 1H, Ar C*H*), 7.76 (d, *J* = 8.5 Hz, 1H, Ar C*H*), 7.55 (ddd, *J* = 8.5, 6.8, 1.4 Hz, 1H, Ar C*H*), 7.47 (ddd, *J* = 8.0, 6.8, 1.1 Hz, 1H, Ar C*H*), 7.30 (d, *J* = 8.4 Hz, 1H, Ar C*H*), 4.18 (s, 2H, SC*H₂*), 4.05 (s, 2H, C(O)C*H₂*), 3.70 (br., 4H, C*H₂*), 3.55 – 3.50 (m, 2H, C*H₂*), 3.48 – 3.44 (m, 2H, C*H₂*), 2.20 (s, 3H, SC*H₃*).

^{13}C{^{1}H} NMR (126 MHz, $CDCl_3$): δ 169.9 (*C*=O), 133.0 (Ar *C*), 132.5 (Ar *C*), 131.7 (Ar *C*), 131.1 (Ar *C*), 128.7 (Ar *C*H), 128.4 (Ar *C*H), 127.0 (Ar *C*H), 126.7 (Ar *C*H), 125.7 (Ar *C*H), 124.0 (Ar *C*H), 67.0 (*C*H$_2$), 66.7 (*C*H$_2$), 46.6 (*C*H$_2$), 42.4 (*C*H$_2$), 38.7 (C(O)*C*H$_2$), 31.8 (S*C*H$_2$), 16.6 (S*C*H$_3$).

HRMS: calcd. for $C_{18}H_{21}NO_2SNa$ [M+Na]$^+$: 338.1185; found (ESI$^+$): 338.1176.

ν_{max} (neat/cm^{-1}): 802, 1034, 1114, 1228, 1433, 1642, 2854, 2916, 3051.

m.p.: 150-151 °C.

(2-Oxo-1-phenyl-2-(4-tosylpiperazin-1-yl)ethyl)dimethylsulfonium trifluoromethanesulfonate (2l)

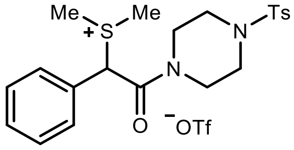

$C_{22}H_{27}F_3N_2O_6S_3$
MW: 568.64

Synthesised according to **GP-6** from 2-phenyl-1-(4-tosylpiperazin-1-yl)ethan-1-one (358 mg, 1.0 mmol), Tf_2O (0.18 mL, 1.1 mmol), and 2-chloropyridine (0.19 mL, 2.0 mmol) in CH_2Cl_2 (3.5 mL) *then* DMSO (0.18 mL in 0.5 mL CH_2Cl_2, 2.5 mmol) and TFAA (0.14 mL, 1.0 mmol). Purification by column chromatography (silica gel; 0-2% MeOH in EtOAc) afforded the product as a yellow solid (426 mg, 0.748 mmol, 75%).

^{1}H NMR (400 MHz, CD_3CN): δ 7.56 – 7.47 (m, 3H, Ar *H*), 7.46 – 7.37 (m, 4H, Ar *H*), 7.37 – 7.32 (m, 2H, Ar *H*), 5.91 (s, 1H, C*H*SMe_2), 3.74 (ddd, *J* = 13.4, 6.3, 3.2 Hz, 1H, NC*H*$_2$), 3.54 (ddd, *J* = 13.4, 7.8, 3.3 Hz, 1H, NC*H*$_2$), 3.39 (ddd, *J* = 14.0, 7.8, 3.3 Hz, 1H, NC*H*$_2$), 3.19 (dddd, *J* = 14.0, 6.3, 3.3, 1.0 Hz, 1H, NC*H*$_2$), 3.06 (dddd, *J* = 11.8, 6.3, 3.3, 1.0 Hz, 1H, NC*H*$_2$), 2.86 (dddd, *J* = 11.8, 6.3, 3.3, 1.0 Hz, 1H, NC*H*$_2$), 2.76 (s, 3H, C*H*$_3$), 2.70 (ddd, *J* = 11.8, 7.8, 3.3 Hz, 1H, NC*H*$_2$), 2.46 (s, 3H, C*H*$_3$), 2.45 (s, 3H, C*H*$_3$), 2.01 – 1.95 (m, 1H, NC*H*$_2$).

^{13}C{^{1}H} NMR (101 MHz, CD_3CN): δ 164.5 (C=O), 145.5 (Ar *C*), 132.8 (Ar *C*), 132.4 (Ar *C*), 131.2 (Ar *C*H), 130.9 (Ar *C*H), 130.8 (Ar *C*H), 128.6 (Ar *C*H), 127.0 (Ar *C*H), 122.1 (q, *J* = 320.8 Hz, SO_2CF_3), 66.8 (*C*HSMe_2), 46.3 (*C*H$_2$), 46.1 (*C*H$_2$), 45.9 (*C*H$_2$), 42.9 (*C*H$_2$), 25.9 (S*C*H$_3$), 23.1 (S*C*H$_3$), 21.5 (*C*H$_3$).

^{19}F NMR (376 MHz, $CDCl_3$): δ -78.40 (s, SO_2CF_3).

HRMS: calcd. for $C_{21}H_{27}N_2O_3S_2$ [M-OTf]$^+$: 419.1463; found (ESI$^+$): 419.1480.

ν_{max} (neat/cm^{-1}): 724, 913, 1028, 1163, 1223, 1254, 1348, 1452, 1639, 2938.

m.p.: (recrystallized from EtOAc) 149-150 °C.

2-(2-((Methylthio)methyl)phenyl)-1-(4-tosylpiperazin-1-yl)ethan-1-one (3l)

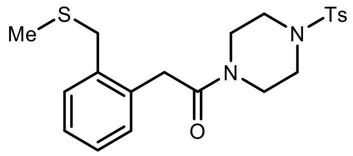

$C_{21}H_{26}N_2O_3S_2$
MW: 418.57

Synthesised according to **GP-7** from **2l** (56.8 mg, 0.1 mmol), with sodium *tert*-butoxide (19.2 mg, 0.2 mmol) in CH_2Cl_2 (1.0 mL). Purification by column chromatography (silica gel; 0-50% EtOAc in hexanes) afforded the product as a white solid (36.4 mg, 0.087 mmol, 87%).

^{1}H NMR (500 MHz, CDCl$_3$): δ 7.61 – 7.57 (m, 2H, Ar C*H*), 7.36 – 7.31 (m, 2H, Ar C*H*), 7.19 – 7.08 (m, 3H, Ar C*H*), 7.01 (dd, *J* = 7.6, 1.3 Hz, 1H, Ar C*H*), 3.81 (s, 2H, C(O)CH_2), 3.76 – 3.70 (m, 2H, CH_2), 3.60 (s, 2H, SCH_2), 3.55 – 3.48 (m, 2H, CH_2), 2.98 (t, *J* = 5.2 Hz, 2H, CH_2), 2.85 (t, *J* = 5.0 Hz, 2H, CH_2), 2.45 (s, 3H, CH_3), 1.97 (s, 3H, SCH_3).

^{13}C{^{1}H} NMR (126 MHz, CDCl$_3$): δ 169.6 (*C*=O), 144.2 (Ar *C*), 135.4 (Ar *C*), 133.4 (Ar *C*), 132.3 (Ar *C*), 130.8 (Ar *C*H), 129.9 (Ar *C*H), 129.5 (Ar *C*H), 127.9 (Ar *C*H), 127.8 (Ar *C*H), 127.0 (Ar *C*H), 46.0 (*C*H$_2$), 45.9 (*C*H$_2$), 45.5 (*C*H$_2$), 41.2 (*C*H$_2$), 37.6 (C(O)*C*H$_2$), 36.4 (S*C*H$_2$), 21.7 (*C*H$_3$), 15.2 (S*C*H$_3$).

HRMS: calcd. for $C_{21}H_{26}N_2O_3S_2Na$ [M+Na]$^+$: 441.1277; found (ESI$^+$): 441.1269.

ν_{max} (neat/cm^{-1}): 649, 724, 914, 1163, 1346, 1433, 1644, 2854, 2916.

m.p.: 108-111 °C.

(2-(Pyrrolidin-1-yl)-2-oxo-1-phenylethyl)dimethylsulfonium trifluoromethanesulfonate (2m)

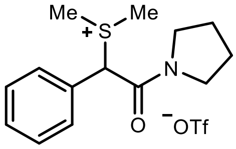

$C_{15}H_{20}F_3NO_4S_2$
MW: 399.44

Synthesised according to **GP-6** from 2-phenyl-1-(pyrrolidin-1-yl)ethan-1-one (189 mg, 1.0 mmol), Tf_2O (0.18 mL, 1.1 mmol), and 2-chloropyridine (0.19 mL, 2.0 mmol) in CH_2Cl_2 (3.5 mL) *then* DMSO (0.18 mL in 0.5 mL CH_2Cl_2, 2.5 mmol) and TFAA (0.14 mL, 1.0 mmol). Purification by column chromatography (silica gel; 0-10% MeOH in EtOAc) afforded the product as a brown oil (327 mg, 0.82 mmol, 82%).

^{1}H NMR (400 MHz, CD_3CN): δ 7.60 – 7.53 (m, 3H, Ar *H*), 7.49 – 7.44 (m, 2H, Ar *H*), 5.64 (s, 1H, C*H*SMe$_2$), 3.52 – 3.42 (m, 2H, 2 x CH_{2A}), 3.41 – 3.34 (m, 1H, CH_{2B}), 2.80 (s, 3H, SCH_3), 2.78 – 2.71 (m, 1H, CH_{2B}), 2.48 (s, 3H, SCH_3), 1.90 – 1.80 (m, 2H, C$H_{2A,}$ CH_{2B}), 1.79 – 1.67 (m, 2H, C$H_{2A,}$ CH_{2B}).

^{13}C{^{1}H} NMR (101 MHz, CD_3CN): δ 163.7 (*C*=O), 132.3 (Ar *C*H), 131.1 (Ar *C*H), 131.0 (Ar *C*H), 127.1 (Ar *C*), 67.6 (*C*HSMe$_2$), 47.7 (*C*H$_2$), 47.5 (*C*H$_2$), 26.5 (*C*H$_2$), 26.0 (S*C*H$_3$), 24.6 (*C*H$_2$), 23.1 (S*C*H$_3$). (*C*F$_3$ not observed but detected by ^{19}F NMR.)

^{19}F NMR (376 MHz, CD_3CN): -79.30 (s, SO$_2$C*F$_3$*).

HRMS: calcd. for $C_{14}H_{20}NOS$ [M-OTf]$^+$: 250.1260; found (ESI$^+$): 250.1265.

ν_{max} (neat/cm^{-1}): 639, 1032, 1163, 1260, 1449, 1640, 2849, 2917, 3024.

2-(2-((Methylthio)methyl)phenyl)-1-(pyrrolidin-1-yl)ethan-1-one (3m)

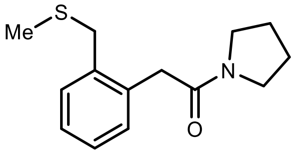

$C_{14}H_{19}NOS$
MW: 249.12

Synthesised according to **GP-7** from **2m** (39.9 mg, 0.1 mmol), with sodium *tert*-butoxide (19.2 mg, 0.2 mmol) in CH_2Cl_2 (1.0 mL). Purification by column chromatography (silica gel; 0-50% EtOAc in hexanes) afforded the product as an off yellow oil (20.6 mg, 0.083 mmol, 83%).

^{1}H NMR (400 MHz, $CDCl_3$): δ 7.25 – 7.13 (m, 4H, Ar *H*), 3.81 (s, 2H, C(O)CH_2), 3.72 (s, 2H, SCH_2), 3.53 – 3.45 (m, 4H, CH_2), 2.01 (s, 3H, SCH_3), 1.98 – 1.82 (m, 4H, CH_2).

^{13}C{^{1}H} NMR (101 MHz, $CDCl_3$): δ 169.4 (*C*=O), 136.2 (Ar *C*), 134.2 (Ar *C*), 130.5 (Ar *C*H), 130.2 (Ar *C*H), 127.6 (Ar *C*H), 126.8 (Ar *C*H), 47.0 (*C*H_2), 46.0 (*C*H_2), 39.1 (C(O)*C*H_2), 36.5 (S*C*H_2), 26.3 (*C*H_2), 24.5 (*C*H_2), 15.3 (S*C*H_3).

HRMS: calcd. for $C_{14}H_{20}NOS$ $[M+H]^+$: 250.1260; found (ESI$^+$): 250.1254.

ν_{max} (neat/cm^{-1}): 672, 1048, 1251, 1420, 1489, 1631, 2871, 2914.

(2-(Piperidin-1-yl)-2-oxo-1-phenylethyl)dimethylsulfonium trifluoromethanesulfonate (2n)

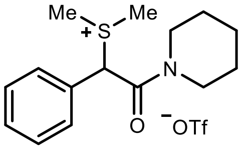

$C_{16}H_{22}F_3NO_4S_2$
MW: 413.47

Synthesised according to **GP-6** from 1-(piperidin-1-yl)-2-phenylethan-1-one (203 mg, 1.0 mmol), Tf_2O (0.18 mL, 1.1 mmol), and 2-chloropyridine (0.19 mL, 2.0 mmol) in CH_2Cl_2 (3.5 mL) *then* DMSO (0.18 mL in 0.5 mL CH_2Cl_2, 2.5 mmol) and TFAA (0.14 mL, 1.0 mmol). Purification by column chromatography (silica gel; 0-10% MeOH in EtOAc) afforded the product as a pale yellow solid (321 mg, 0.78 mmol, 78%).

^{1}H NMR (400 MHz, CD_3CN): 7.63 – 7.53 (m, 3H, Ar *H*), 7.48 – 7.43 (m, 2H, Ar *H*), 5.87 (s, 1H, C*H*SMe$_2$), 3.77 – 3.69 (m, 1H, CH_{2A}), 3.37 – 3.29 (m, 1H, CH_{2B}), 3.25 – 3.11 (m, 2H, CH_2), 2.77 (s, 3H, SCH_3), 2.47 (s, 3H, SCH_3), 1.60 – 1.47 (m, 3H, CH_2 + CH_{2A}), 1.44 – 1.31 (m, 2H, CH_2), 0.81 – 0.71 (m, 1H, CH_{2B}).

^{13}C{^{1}H} NMR (101 MHz, CD_3CN): 163.8 (*C*=O), 132.3 (Ar *C*H), 131.2 (Ar *C*H), 130.8 (Ar *C*H), 127.6 (Ar *C*), 122.0 (q, J = 320.3 Hz, SO$_2$*C*F$_3$), 67.7 (*C*H), 47.9 (*C*H$_2$), 44.6 (*C*H$_2$), 26.0 (*C*H$_2$), 25.9 (S*C*H$_3$ + *C*H$_2$), 24.4 (*C*H$_2$), 23.3 (S*C*H$_3$).

^{19}F NMR (376 MHz, CD_3CN): -79.35 (s, SO$_2$CF_3).

HRMS: calcd. for $C_{15}H_{22}NOS$ [M-OTf]$^+$: 264.1417; found (ESI$^+$): 264.1416.

v_{max} (neat/cm^{-1}): 639, 893, 1032, 1256, 1455, 1637, 2861, 2941, 3025, 3337.

m.p.: (recrystallized from EtOAc) 135-137 °C.

2-(2-((Methylthio)methyl)phenyl)-1-(piperidin-1-yl)ethan-1-one (3n)

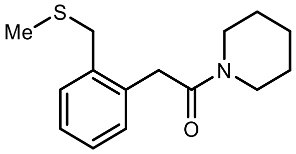

$C_{15}H_{21}NOS$
MW: 263.40

Synthesised according to **GP-7** from **2n** (41.3 mg, 0.1 mmol), with sodium *tert*-butoxide (19.2 mg, 0.2 mmol) in CH_2Cl_2 (1.0 mL). Purification by column chromatography (silica gel; 0-50% EtOAc in hexanes) afforded the product as an off yellow oil (21.3 mg, 0.081 mmol, 81%).

^{1}H NMR (400 MHz, $CDCl_3$): δ 7.24 – 7.11 (m, 4H, Ar *H*), 3.86 (s, 2H, C(O)CH_2), 3.67 (s, 2H, SCH_2), 3.59 (t, J = 5.4 Hz, 2H, CH_2), 3.41 – 3.33 (m, 2H, CH_2), 1.99 (s, 3H, SCH_3), 1.64 – 1.36 (m, 6H, CH_2).

^{13}C{^{1}H} NMR (101 MHz, $CDCl_3$): δ 169.2 (*C*=O), 135.5 (Ar *C*), 134.4 (Ar *C*), 130.4 (Ar *C*H), 129.6 (Ar *C*H), 127.6 (Ar *C*H), 126.6 (Ar *C*H), 47.2 (*C*H_2), 42.9 (*C*H_2), 37.7 (C(O)*C*H_2), 36.4 (S*C*H_2), 26.4 (*C*H_2), 25.6 (*C*H_2), 24.5 (*C*H_2), 15.2 (S*C*H_3).

HRMS: calcd. for $C_{15}H_{22}NOS$ $[M+H]^+$: 264.1417; found (ESI$^+$): 264.1410.

ν_{max} (neat/cm^{-1}): 688, 1049, 1235, 1422, 1490, 1632, 2853, 2932.

2-(2-((Methylthio)methyl)phenyl)-1-(2-phenylmorpholino)ethan-1-one (3o)

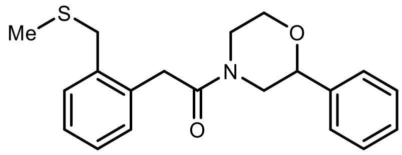

$C_{20}H_{23}NO_2S$
MW: 341.47

Synthesised according to **GP-9**, with potassium hydroxide (11.2 mg, 0.2 mmol) in CH_2Cl_2 (1.0 mL). Purification by column chromatography (silica gel; 0-50% EtOAc in hexanes) afforded the product as an off yellow oil (133 mg, 0.39 mmol, 78%).

A 1.3:1 mixture of rotamers was observed at 298 K in $CDCl_3$.

^{1}H NMR (400 MHz, $CDCl_3$): δ 7.49 – 7.02 (m, 9H, Ar *H*), 4.73 – 4.41 (m, 1.5H, C*H*$_2$ + C*H*), 4.28 – 4.02 (m, 1.2H, C*H*$_2$ + C*H*), 4.02 – 3.82 (m, 3H, C(O)C*H*$_2$ + C*H*$_2$), 3.80 – 3.54 (m, 3.2H, SC*H*$_2$ + C*H*$_2$), 3.33 – 3.25 (m, 0.5H, C*H*$_2$), 2.97 (m, 1.2H, C*H*$_2$), 2.82 – 2.46 (m, 0.8H, C*H*$_2$), 2.03 (s, 1.4H, SC*H*$_3$), 1.86 (s, 1.6H, SC*H*$_3$).

^{13}C{^{1}H} NMR (101 MHz, $CDCl_3$): δ 169.8 (*C*=O), 169.8 (*C*=O), 138.8 (Ar *C*), 138.7 (Ar *C*), 138.6 (Ar *C*), 135.6 (Ar *C*), 135.3 (Ar *C*), 135.2 (Ar *C*), 133.9 (Ar *C*H), 133.8 (Ar *C*H), 130.8 (Ar *C*H), 130.8 (Ar *C*H), 129.6 (Ar *C*H), 129.4 (Ar *C*H), 128.6 (Ar *C*H), 128.6 (Ar *C*H), 128.3 (Ar *C*H), 128.0 (Ar *C*H), 127.1 (Ar *C*H), 127.0 (Ar *C*H), 126.2 (Ar *C*H), 126.1 (Ar *C*H), 78.3 (*C*H), 78.1 (*C*H), 67.1 (*C*H$_2$), 66.7 (*C*H$_2$), 52.9 (*C*H$_2$), 48.1 (*C*H$_2$), 46.1 (*C*H$_2$), 41.8 (*C*H$_2$), 37.9 (C(O)*C*H$_2$), 37.8 (C(O)*C*H$_2$), 36.6 (S*C*H$_2$), 36.4 (S*C*H$_2$), 15.4 (S*C*H$_3$), 14.9 (S*C*H$_3$).

HRMS: calcd. for $C_{20}H_{24}NO_2S$ $[M+H]^+$: 342.1522; found (ESI$^+$): 342.1529.

ν_{max} (neat/cm^{-1}): 681, 1033, 1257, 1467, 1633, 2851, 2934.

(2-(Dimethylamino)-2-oxo-1-phenylethyl)dimethylsulfonium trifluoromethanesulfonate (2p)

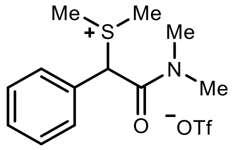

$C_{13}H_{18}F_3NO_4S_2$
MW: 373.41

Synthesised according to **GP-6** from *N,N*-dimethyl-2-phenylacetamide (163 mg, 1.0 mmol), Tf_2O (0.18 mL, 1.1 mmol), and 2-chloropyridine (0.19 mL, 2.0 mmol) in CH_2Cl_2 (3.5 mL) *then* DMSO (0.18 mL in 0.5 mL CH_2Cl_2, 2.5 mmol) and TFAA (0.14 mL, 1.0 mmol). Purification by column chromatography (silica gel; 0-10% MeOH in EtOAc) afforded the product as an off brown oil (330 mg, 0.88 mmol, 88%).

^{1}H NMR (400 MHz, CD_3CN): δ 7.61 – 7.54 (m, 3H, Ar *H*), 7.48 – 7.43 (m, 2H, Ar *H*), 5.87 (s, 1H, C*H*SMe$_2$), 2.93 (s, 3H, C*H*$_3$), 2.80 (s, 3H, C*H*$_3$), 2.76 (s, 3H, SC*H*$_3$), 2.48 (s, 3H, SC*H*$_3$).

^{13}C{^{1}H} NMR (101 MHz, CD_3CN): δ 165.7 (*C*=O), 132.4 (Ar *C*H), 131.2 (Ar *C*H), 130.8 (Ar *C*H), 127.2 (Ar *C*), 121.9 (q, *J* = 320.3 Hz, *C*F$_3$), 67.5 (*C*H), 37.7 (*C*H$_3$), 36.5 (*C*H$_3$), 26.0 (S*C*H$_3$), 23.2 (S*C*H$_3$).

^{19}F NMR (376 MHz, $CDCl_3$): δ -79.37 (s, SO$_2$C*F*$_3$).

HRMS: calcd. for $C_{18}H_{28}NO_2S$ [M-OTf]$^+$: 224.1104; found (ESI$^+$): 224.1111.

v_{max} (neat/cm^{-1}): 639, 1030, 1157, 1257, 1648, 2858, 2935, 3031.

***N,N*-Dimethyl-2-(2-((methylthio)methyl)phenyl)acetamide (3p)**

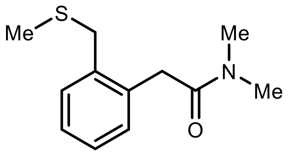

$C_{12}H_{17}NOS$
MW: 223.33

Synthesised according to **GP-7** from **2p** (37.3 mg, 0.1 mmol), with sodium *tert*-butoxide (19.2 mg, 0.2 mmol) in CH_2Cl_2 (1.0 mL). Purification by column chromatography (silica gel; 0-50% EtOAc in hexanes) afforded the product as an off yellow oil (19.0 mg, 0.085 mmol, 85%).

^{1}H NMR (400 MHz, $CDCl_3$): δ 7.37 – 7.17 (m, 4H, Ar *H*), 3.96 (s, 2H, C(O)CH_2), 3.76 (s, 2H, SCH_2), 3.08 (s, 6H, NCH_3), 2.08 (s, 3H, SCH_3).

^{13}C{^{1}H} NMR (101 MHz, $CDCl_3$): δ 171.2 (*C*=O), 135.8 (Ar *C*), 134.2 (Ar *C*), 130.5 (Ar *C*H), 129.8 (Ar *C*H), 127.8 (Ar *C*H), 126.8 (Ar *C*H), 37.8 (C(O)*C*H_2), 37.8 (N*C*H_3), 36.5 (S*C*H_2), 15.3 (S*C*H_3).

HRMS: calcd. for $C_{12}H_{18}NOS$ $[M+H]^+$: 224.1104; found (ESI$^+$): 224.1097.

ν_{max} (neat/cm^{-1}): 671, 1051, 1263, 1435, 1490, 1639, 2915, 3019.

(2-(Methoxy(methyl)amino)-2-oxo-1-phenylethyl)dimethylsulfonium trifluoromethanesulfonate (2q)

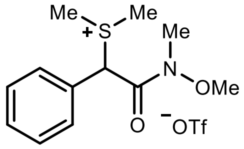

$C_{13}H_{18}F_3NO_5S_2$
MW: 389.40

Synthesised according to **GP-6** from *N*-methoxy-*N*-methyl-2-phenylacetamide (179 mg, 1.0 mmol), Tf_2O (0.18 mL, 1.1 mmol), and 2-chloropyridine (0.19 mL, 2.0 mmol) in CH_2Cl_2 (3.5 mL) *then* DMSO (0.18 mL in 0.5 mL CH_2Cl_2, 2.5 mmol) and TFAA (0.14 mL, 1.0 mmol). Purification by column chromatography (silica gel; 0-2% MeOH in EtOAc) afforded the product as a viscous yellow oil (196 mg, 0.504 mmol, 50%).

^{1}H NMR (400 MHz, $CDCl_3$): δ 7.63 – 7.58 (m, 2H, Ar *H*), 7.56 – 7.51 (m, 3H, Ar *H*), 6.64 (s, 1H, C*H*SMe$_2$), 3.61 (s, 3H, OC*H*$_3$), 3.25 (s, 3H, SC*H*$_3$), 3.20 (s, 3H, SC*H*$_3$), 2.76 (s, 3H, NC*H*$_3$).

^{13}C{^{1}H} NMR (101 MHz, $CDCl_3$): δ 165.3 (*C*=O), 131.4 (Ar *C*), 130.3 (Ar *C*H), 129.7 (Ar *C*H), 127.4 (Ar *C*H), 120.7 (q, *J* = 319.5 Hz, SO$_2$*C*F$_3$), 66.5 (*C*HSMe$_2$), 62.0 (O*C*H$_3$), 32.6 (N*C*H$_3$), 25.4 (S*C*H$_3$), 21.8 (S*C*H$_3$).

^{19}F NMR (376 MHz, $CDCl_3$): δ -78.41 (s, SO$_2$C*F*$_3$).

HRMS: calcd. for $C_{12}H_{18}NO_2S$ [M-OTf]$^+$: 240.1058; found (ESI$^+$): 240.1052.

ν_{max} (neat/cm^{-1}): 638, 1030, 1157, 1256, 1432, 1656, 2940, 3022.

_N_-Methoxy-_N_-methyl-2-(2-((methylthio)methyl)phenyl)acetamide (3q)

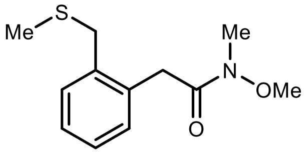

$C_{12}H_{17}NO_2S$
MW: 239.33

Synthesised according to **GP-7** from **2q** (38.9 mg, 0.1 mmol), with sodium *tert*-butoxide (19.2 mg, 0.2 mmol) in CH_2Cl_2 (1.0 mL). Purification by column chromatography (silica gel; 0-50% EtOAc in hexanes) afforded the product as an off yellow oil (12.7 mg, 0.053 mmol, 53%).

^{1}H NMR (500 MHz, $CDCl_3$): δ 7.25 – 7.18 (m, 4H, Ar C*H*), 3.95 (s, 2H, C(O)CH_2), 3.74 (s, 2H, SCH_2), 3.67 (s, 3H, OCH_3), 3.21 (s, 3H, NCH_3), 2.01 (s, 3H, SCH_3).

^{13}C{^{1}H} NMR (126 MHz, $CDCl_3$): δ 172.5 (*C*=O), 136.4 (Ar *C*), 134.0 (Ar *C*), 131.1 (Ar *C*H), 130.5 (Ar *C*H), 127.6 (Ar *C*H), 127.0 (Ar *C*H), 61.5 (O*C*H_3), 36.4 (S*C*H_2), 36.3 (C(O)*C*H_2), 32.5 (N*C*H_3), 15.3 (S*C*H_3).

HRMS: calcd. for $C_{12}H_{18}NO_2S$ $[M+H]^+$: 240.1053; found (ESI$^+$): 240.1048.

ν_{max} (neat/cm^{-1}): 733, 1002, 1116, 1171, 1379, 1415, 1660, 2855, 2917.

(2-((3-(9,10-Ethanoanthracen-9(10*H*)-yl)propyl)(methyl)amino)-2-oxo-1-phenylethyl)dimethylsulfonium trifluoromethanesulfonate (2r)

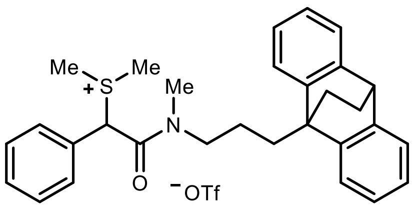

$C_{31}H_{34}F_3NO_4S_2$
MW: 605.73

Synthesised according to **GP-6** from *N*-(2-phenylacetyl)maprotiline (427 mg, 1.0 mmol), Tf_2O (0.18 mL, 1.1 mmol), and 2-chloropyridine (0.19 mL, 2.0 mmol) in CH_2Cl_2 (3.5 mL) *then* DMSO (0.18 mL in 0.5 mL CH_2Cl_2, 2.5 mmol) and TFAA (0.14 mL, 1.0 mmol). Purification by column chromatography (silica gel; 50-100% EtOAc in hexanes then 0-2% MeOH in EtOAc) afforded the product as a yellow foam (314 mg, 0.519 mmol, 52%).

A 2:1 mixture of rotamers was observed at 298 K in $CDCl_3$.

^{1}H NMR (400 MHz, $CDCl_3$): δ 7.81 – 7.65 (m, 2H, Ar *H*), 7.60 – 7.44 (m, 3H, Ar *H*), 7.32 – 7.03 (m, 8H, Ar *H*), 6.97 (s, 0.33H, C*H*SMe$_2$), 6.96 – 6.83 (m, 0.66H, Ar *H*), 6.79 (s, 0.66H, C*H*SMe$_2$), 4.33 – 4.20 (m, 1H, C*H*), 4.07 – 3.81 (m, 1H, NCH_{2A}), 3.52 – 3.35 (m, 1H, NCH_{2B}), 3.29 – 3.15 (m, 3H, SCH_3), 3.13 – 3.03 (m, 3H, NCH_3), 2.76 – 2.69 (m, 3H, SCH_3), 2.50 – 2.32 (m, 1.33H, CH_2), 2.12 – 1.93 (m, 1.66H, CH_2), 1.91 – 1.70 (m, 2H, CH_2), 1.62 – 1.44 (m, 1.33H, CH_2), 1.25 – 1.17 (m, 0.33H, CH_2), 1.12 – 0.95 (m, 0.33H, CH_2).

^{13}C NMR (101 MHz, $CDCl_3$): δ 165.1 (*C*=O), 164.6 (*C*=O), 145.1 (Ar *C*), 145.03 (Ar *C*), 145.02 (Ar *C*), 144.9 (Ar *C*), 144.8 (Ar *C*), 144.6 (Ar *C*), 131.84 (Ar *C*H), 131.75 (Ar *C*H), 130.8 (Ar *C*H), 130.7 (Ar *C*H), 129.8 (Ar *C*H), 127.9 (Ar *C*), 126.9 (Ar *C*H), 125.52 (Ar *C*H), 125.50 (Ar *C*H), 125.50 (Ar *C*H), 125.43 (Ar *C*H), 125.41 (Ar *C*H), 123.64 (Ar *C*H), 123.61 (Ar *C*H), 123.57 (Ar *C*H), 123.4 (Ar *C*H), 120.7 (q, J = 319.9 Hz, SO$_2$*C*F$_3$), 121.14 (Ar *C*H), 121.07 (Ar *C*H), 121.0 (Ar *C*H), 68.0 (*C*HSMe$_2$), 67.9 (*C*HSMe$_2$), 50.9 (N*C*H$_2$), 50.0 (N*C*H$_2$), 44.7 (*C*H), 44.53 (*C*H), 44.45 (*C*H), 44.4 (*C*H), 35.7 (N*C*H$_3$), 33.9 (N*C*H$_3$), 29.6 (*C*H$_2$), 28.0 (*C*H$_2$), 27.64 (*C*H$_2$), 27.55 (*C*H$_2$), 27.5 (*C*H$_2$), 26.4 (S*C*H$_3$), 26.2 (S*C*H$_3$), 23.0 (*C*H$_2$), 22.5 (*C*H$_2$), 22.0 (S*C*H$_3$), 21.8 (S*C*H$_3$).

^{19}F NMR (376 MHz, CD_3CN): δ -78.31 (s, SO$_2$CF_3).

HRMS: calcd. for $C_{30}H_{34}NOS$ [M-OTf]$^+$: 456.2361; found (ESI$^+$): 456.2377.

ν_{max} (neat/cm^{-1}): 1027, 1150, 1223, 1240, 1276, 1450, 1646, 2862, 2931.

N-(3-(9,10-Ethanoanthracen-9(10H)-yl)propyl)-N-methyl-2-(2-((methylthio)methyl)phenyl)acetamide (3r)

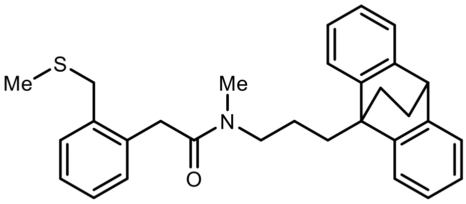

$C_{30}H_{33}NOS$
MW: 455.66

Synthesised according to **GP-8** from **2r** (60.5 mg, 0.1 mmol), with potassium hydroxide (11.2 mg, 0.2 mmol) in acetonitrile (1.0 mL). Purification by column chromatography (silica gel; 0-50% EtOAc in hexanes) afforded the product as a yellow oil (33.3 mg, 0.073 mmol, 73%).

A 1.2:1 mixture of rotamers was observed at 298 K in $CDCl_3$.

^{1}H NMR (400 MHz, $CDCl_3$): δ 7.28 – 7.19 (m, 7H, Ar *H*), 7.16 – 7.04 (m, 5H, Ar *H*), 4.28 (t, *J* = 2.7 Hz, 1H, C*H*), 4.06 (m, 1H, NCH_2), 3.93 (m, 1H, NCH_2), 3.77 – 3.69 (m, 3H, CH_2), 3.58 (t, *J* = 7.7 Hz, 1H, CH_2), 3.15 – 3.06 (m, 3H, NCH_3), 2.12 – 1.97 (m, 4H, CH_2 + SCH_3), 1.83 (ddt, *J* = 10.6, 4.3, 1.9 Hz, 2H, CH_2), 1.67 – 1.48 (m, 3H, CH_2).

^{13}C{^{1}H} NMR (101 MHz, $CDCl_3$): δ 171.0 (*C*=O), 171.0 (*C*=O), 145.4 (Ar *C*), 145.1 (Ar *C*), 145.1 (Ar *C*), 145.0 (Ar *C*), 135.9 (Ar *C*), 135.8 (Ar *C*), 134.4 (Ar *C*), 134.3 (Ar *C*), 130.7 (Ar *C*H), 130.6 (Ar *C*H), 130.0 (Ar *C*H), 129.7 (Ar *C*H), 127.9 (Ar *C*H), 127.8 (Ar *C*H), 127.0 (Ar *C*H), 126.9 (Ar *C*H), 125.5 (Ar *C*H), 125.5 (Ar *C*H), 125.4 (Ar *C*H), 123.6 (Ar *C*H), 123.5 (Ar *C*H), 121.3 (Ar *C*H), 121.0 (Ar *C*H), 51.4 (*C*H_2), 49.1 (*C*H_2), 44.9 (*C*H_2), 44.7 (*C*H), 44.6 (*C*H), 38.2 (*C*H_2), 37.9 (*C*H_2), 36.7 (*C*H_2), 36.6 (*C*H_2), 36.1 (N*C*H_3), 33.7 (N*C*H_3), 29.8 (*C*H_2), 28.3 (*C*H_2), 28.2 (*C*H_2), 27.8 (*C*H_2), 27.7 (*C*H_2), 24.2 (*C*H_2), 22.9 (*C*H_2), 15.5 (S*C*H_3), 15.3 (S*C*H_3).

HRMS: calcd. for $C_{30}H_{34}NOS$ $[M+H]^+$: 456.2356; found (ESI$^+$): 456.2354.

ν_{max} (neat/cm^{-1}): 672, 1050, 1266, 1455, 1485, 1641, 1696, 2866, 2946.

(2-(Methyl(3-phenyl-3-(4-(trifluoromethyl)phenoxy)propyl)amino)-2-oxo-1-phenylethyl) dimethylsulfonium trifluoromethanesulfonate (2s)

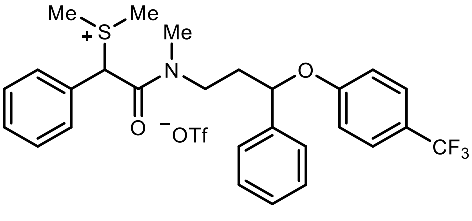

$C_{28}H_{29}F_6NO_5S_2$
MW: 637.65

Synthesised according to **GP-6** from *N*-(2-phenylacetyl)fluoxetine (855 mg, 2.0 mmol), Tf_2O (0.37 mL, 2.2 mmol), and 2-chloropyridine (0.38 mL, 4.0 mmol) in CH_2Cl_2 (7 mL) *then* DMSO (0.36 mL in 1 mL CH_2Cl_2, 5.0 mmol) and TFAA (0.28 mL, 2.0 mmol). Purification by column chromatography (silica gel; 50-100% EtOAc in hexanes then 0-1% MeOH in EtOAc) afforded the product as a yellow foam as an inseparable mixture of diastereomers and rotamers (771 mg, 1.21 mmol, 60%).

^{1}H NMR (400 MHz, CDCl$_3$): δ 7.69 – 7.20 (m, 12H, Ar *H*), 6.97 – 6.81 (m, 2H, Ar *H*), 6.79 – 6.73 (m, 1H, C*H*SMe$_2$), 5.30 – 5.06 (m, 1H, OC*H*), 4.27 – 4.06 (m, 0.5H, C*H$_2$*), 3.79 – 3.59 (m, 0.5H, C*H$_2$*), 3.57 – 3.21 (m, 1H, C*H$_2$*), 3.21 – 2.77 (m, 6H, SC*H$_3$*, NC*H$_3$*), 2.76 – 2.60 (m, 3H, SC*H$_3$*), 2.24 – 2.04 (m, 2H, C*H$_2$*).

^{13}C{^{1}H} NMR (101 MHz, CDCl$_3$): δ 165.1 (*C*=O), 164.9 (*C*=O), 164.7 (*C*=O), 140.42 (Ar *C*), 140.39 (Ar *C*), 140.1 (Ar *C*), 139.8 (Ar *C*), 131.7 (Ar *C*H), 131.64 (Ar *C*H), 131.60 (Ar *C*H), 130.64 (Ar *C*H), 130.60 (Ar *C*H), 129.1 (Ar *C*H), 129.98 (Ar *C*H), 128.95 (Ar *C*H), 128.2 (Ar *C*H), 128.10 (Ar *C*H), 127.4 (Ar *C*H), 127.3 (Ar *C*H), 127.1 – 126.8 (m, Ar *C*H), 126.7 (Ar *C*H), 125.8 (q, *J* = 4.3 Hz, Ar *C*H), 123.5 – 122.4 (m, Ar *C*), 120.7 (q, *J* = 319.7 Hz, *C*F$_3$), 116.0 (Ar *C*H), 115.9 (Ar *C*H), 77.3 (OC*H*), 76.5 (OC*H*), 67.9 (*C*HSMe$_2$), 67.6 (*C*HSMe$_2$), 67.51 (*C*HSMe$_2$), 67.48 (*C*HSMe$_2$), 47.3 (*C*H$_2$), 46.6 (*C*H$_2$), 45.4 (*C*H$_2$), 36.4 (N*C*H$_3$), 36.3 (N*C*H$_3$), 36.1 (N*C*H$_3$), 35.70 (CH$_2$), 35.65 (N*C*H$_3$), 35.4 (N*C*H$_3$), 34.2 (N*C*H$_3$), 34.1 (N*C*H$_3$), 26.0 (S*C*H$_3$), 25.79 (S*C*H$_3$), 25.75 (S*C*H$_3$), 25.7 (S*C*H$_3$), 22.0 (S*C*H$_3$), 21.9 (S*C*H$_3$).

^{19}F NMR (376 MHz, CD$_3$CN): δ -61.54 (C*F$_3$*), -61.59 (C*F$_3$*), -61.61 (C*F$_3$*), -78.32, (SO$_2$C*F$_3$*).

HRMS: calcd. for $C_{27}H_{29}F_3NO_2S$ [M-OTf]$^+$: 488.1871; found (ESI$^+$): 488.1891.

ν_{max} (neat/cm^{-1}): 1027, 1109, 1151, 1223, 1241, 1274, 1451, 1639, 2932.

***N*-Methyl-2-(2-((methylthio)methyl)phenyl)-N-(3-phenyl-3-(4-(trifluoromethyl)phenoxy)propyl)acetamide (3s)**

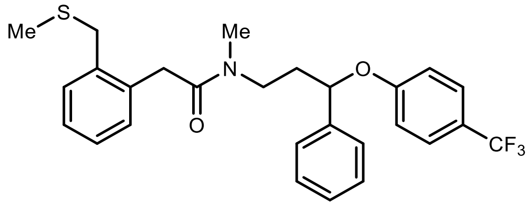

$C_{27}H_{28}F_3NO_2S$
MW: 487.58

Synthesised according to **GP-7** from **2s** (63.7 mg, 0.1 mmol), with sodium *tert*-butoxide (19.2 mg, 0.2 mmol) in CH_2Cl_2 (1.0 mL). Purification by column chromatography (silica gel; 0-50% EtOAc in hexanes) afforded the product as a colourless oil (37.1 mg, 0.076 mmol, 76%).

A 1.5:1 mixture of rotamers was observed at 298 K in $CDCl_3$.

^{1}H NMR (500 MHz, $CDCl_3$): δ 7.47 – 7.40 (m, 2H, Ar C*H*), 7.38 – 7.31 (m, 3H, Ar C*H*), 7.31 – 7.24 (m, 2H, Ar C*H*), 7.23 – 7.16 (m, 3H, Ar C*H*), 7.13 – 6.99 (m, 1H, Ar C*H*), 6.91 – 6.85 (m, 2H, Ar C*H*), 5.26 – 5.12 (m, 1H, OC*H*), 3.92 – 3.76 (m, 2H, C(O)CH_2), 3.72 – 3.46 (m, 4H, CH_2), 3.04 – 2.98 (m, 3H, NCH_3), 2.29 – 2.04 (m, 2H, CH_2), 2.03 – 1.96 (m, 3H, SCH_3).

^{13}C{^{1}H} NMR (126 MHz, $CDCl_3$): δ 171.1 (*C*=O), 171.0 (*C*=O), 160.4 (Ar *C*), 160.1 (Ar *C*), 140.8 (Ar *C*), 140.2 (Ar *C*), 135.9 (Ar *C*), 135.8 (Ar *C*), 134.2 (Ar *C*), 134.1 (Ar *C*), 130.6 (Ar *C*H), 130.5 (Ar *C*H), 129.9 (Ar *C*H), 129.1 (Ar *C*H), 128.9 (Ar *C*H), 128.3 (Ar *C*H), 128.1 (Ar *C*H), 127.7 (Ar *C*H), 127.0 – 126.8 (m, Ar *C*H), 127.00 (q, *J* = 4.2 Hz, Ar *C*H), 125.8 (Ar *C*H), 125.6 (Ar *C*H), 115.8 (Ar *C*H), 115.8 (Ar *C*H), 78.5 (O*C*H), 77.6 (O*C*H), 47.0 (N*C*H_2), 45.7 (N*C*H_2), 38.0 (C(O)*C*H_2), 37.6 (*C*H_2), 37.5 (C(O)*C*H_2), 36.5 (*C*H_2), 36.5 (N*C*H_3), 36.4 (S*C*H_2), 33.7 (N*C*H_3), 15.3 (S*C*H_3), 15.2 (S*C*H_3). (*C*F_3 not observed but detected by ^{19}F NMR.)

^{19}F NMR (376 MHz, $CDCl_3$): δ -61.6 (s, CF_3).

HRMS: calcd. for $C_{27}H_{28}F_3NO_2SNa$ $[M+Na]^+$: 510.1685; found (ESI^+): 510.1711.

ν_{max} (neat/cm^{-1}): 701, 836, 1110, 1160, 1247, 1325, 1643, 2854, 2920.

***Bis*(methyl-d3)(2-morpholino-2-oxo-1-phenylethyl)sulfonium trifluoromethanesulfonate (2t)**

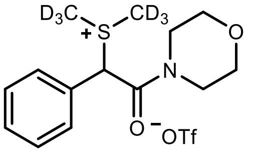

$C_{15}H_{14}D_6F_3NO_5S_2$
MW: 421.48

Synthesised according to **GP-6** from 2-phenyl-1-morpholinoethan-1-one (205 mg, 1.0 mmol), Tf_2O (0.18 mL, 1.1 mmol), and 2-chloropyridine (0.19 mL, 2.0 mmol) in CH_2Cl_2 (3.5 mL) *then* dimethyl sulfoxide-d6 (211 mg in 0.5 mL CH_2Cl_2, 2.5 mmol) and TFAA (0.14 mL, 1.0 mmol). Purification by column chromatography (silica gel; 0-10% MeOH in EtOAc) afforded the product as a yellow solid (299 mg, 0.71 mmol, 71%).

^{1}H NMR (400 MHz, $CDCl_3$): δ 7.59 – 7.44 (m, 5H, Ar *H*), 6.54 (s, 1H, C*H*S(CD_3)$_2$), 3.72 – 3.59 (m, 2H, C*H*$_2$), 3.59 – 3.45 (m, 4H, C*H*$_2$), 3.15 – 3.02 (m, 2H, C*H*$_2$).

^{13}C{^{1}H} NMR (101 MHz, $CDCl_3$): δ 163.6 (*C*=O), 131.7 (Ar *C*H), 130.6 (Ar *C*H), 129.6 (Ar *C*H), 126.9 (Ar *C*), 120.6 (q, *J* = 319.7 Hz, SO_2*C*F_3), 66.3 (*C*HS(CD_3)$_2$), 66.3 (*C*H$_2$), 66.0 (*C*H$_2$), 46.4 (*C*H$_2$), 43.2 (*C*H$_2$), 25.0 (Hept, *J* = 21.5 Hz, *C*D$_3$), 21.1 (Hept, *J* = 24.2 Hz, *C*D$_3$).

^{19}F NMR (376 MHz, $CDCl_3$): δ -78.40 (s, SO_2C*F*$_3$).

HRMS: calcd. for $C_{14}H_{14}D_6NO_2S$ [M-OTf]$^+$: 272.1586; found (ESI$^+$): 272.1585.

v_{max} (neat/cm^{-1}): 637, 1030, 1253, 1438, 1637, 1735, 2866, 2960, 3533.

m.p.: (recrystallized from EtOAc) 141-142 °C.

2-(2-(((Methyl-d3)thio)methyl-d2)phenyl)-1-morpholinoethan-1-one (3t)

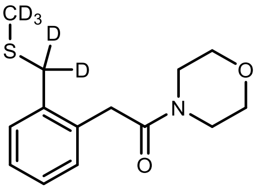

$C_{14}H_{14}D_5NO_2S$
MW: 270.40

Synthesised according to **GP-7** from **2t** (42.1 mg, 0.1 mmol), with sodium *tert*-butoxide (19.2 mg, 0.2 mmol) in CH_2Cl_2 (1.0 mL). Purification by column chromatography (silica gel; 0-50% EtOAc in hexanes) afforded the product as an off yellow oil (23.5 mg, 0.087 mmol, 87%).

^{1}H NMR (400 MHz, CDCl$_3$): δ 7.27 – 7.12 (m, 4H, Ar *H*), 3.88 (s, 2H, C(O)CH_2), 3.68 (s, 4H, CH_2), 3.59 – 3.52 (m, 2H, CH_2), 3.48 – 3.41 (m, 2H, CH_2).

^{13}C{^{1}H} NMR (101 MHz, CDCl$_3$): δ 169.8 (*C*=O), 135.5 (Ar *C*), 133.8 (Ar *C*), 130.6 (Ar *C*H), 129.6 (Ar *C*H), 127.8 (Ar *C*H), 127.0 (Ar *C*H), 67.0 (*C*H$_2$), 66.7 (*C*H$_2$), 46.6 (*C*H$_2$), 42.3 (*C*H$_2$), 37.5 (C(O)*C*H$_2$), 35.8 (p, J = 21.6 Hz, S*C*D$_2$), 14.5 (Hept, J = 21.2 Hz, S*C*D$_3$).

HRMS: calcd. for $C_{14}H_{15}D_5NO_2S$ $[M+H]^+$: 271.1523; found (ESI$^+$): 271.1513.

ν_{max} (neat/cm^{-1}): 645, 1035, 1228, 1429, 1489, 1637, 2853, 2962.

Diethyl(2-morpholino-2-oxo-1-phenylethyl)sulfonium trifluoromethanesulfonate (2u)

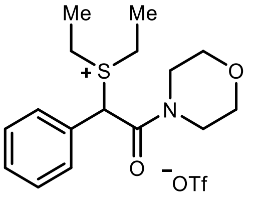

$C_{17}H_{24}F_3NO_5S_2$
MW: 443.50

Synthesised according to **GP-6** from 2-phenyl-1-morpholinoethan-1-one (205 mg, 1.0 mmol), Tf_2O (0.18 mL, 1.1 mmol), and 2-chloropyridine (0.19 mL, 2.0 mmol) in CH_2Cl_2 (3.5 mL) *then* diethyl sulfoxide (266 mg in 0.5 mL CH_2Cl_2, 2.5 mmol) and TFAA (0.14 mL, 1.0 mmol). Purification by column chromatography (silica gel; 0-10% MeOH in EtOAc) afforded the product as a light yellow solid (279 mg, 0.63 mmol, 63%).

^{1}H NMR (400 MHz, CD_3CN): δ 7.64 – 7.47 (m, 5H, Ar *H*), 5.97 (s, 1H, C*H*S(CH_2)$_2$), 3.69 – 3.20 (m, 8H, C*H*$_2$), 3.12 – 2.80 (m, 4H, C*H*$_2$), 1.49 (t, *J* = 7.4 Hz, 3H, C*H*$_3$), 1.14 (t, *J* = 7.5 Hz, 3H, C*H*$_3$).

^{13}C{^{1}H} NMR (101 MHz, CD_3CN): δ 164.2 (*C*=O), 132.2 (Ar *C*H), 131.3 (Ar *C*H), 130.6 (Ar *C*H), 127.9 (Ar *C*), 122.0 (q, *J* = 320.3 Hz, SO_2*C*F_3) 66.8 (*C*HS(CH_2)$_2$), 66.4 (*C*H_2), 64.4 (*C*H_2), 47.2 (*C*H_2), 43.9 (*C*H_2), 35.7 (*C*H_2), 32.9(*C*H_2), 9.9 (*C*H_3), 9.8 (*C*H_3).

^{19}F NMR (376 MHz, CD_3CN): δ -79.25 (s, SO_2C*F*$_3$).

HRMS: calcd. for $C_{16}H_{24}NO_2S$ [M-OTf]$^+$: 294.1522; found (ESI$^+$): 294.1529.

v_{max} (neat/cm^{-1}): 639, 1031, 1248, 1450, 1632, 1909, 2923, 3461.

m.p.: (recrystallized from EtOAc) 139-141 °C.

2-(2-(1-(Ethylthio)ethyl)phenyl)-1-morpholinoethan-1-one (3u)

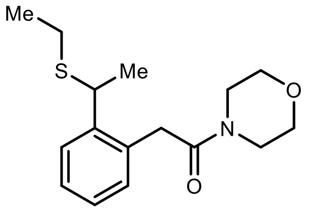

$C_{16}H_{23}NO_2S$
MW: 293.43

Synthesised according to **GP-7** from **2u** (44.3 mg, 0.1 mmol), with sodium *tert*-butoxide (19.2 mg, 0.2 mmol) in CH_2Cl_2 (1.0 mL). Purification by column chromatography (silica gel; 0-50% EtOAc in hexanes) afforded the product as an off yellow oil (18.2 mg, 0.062 mmol, 62%).

^{1}H NMR (400 MHz, $CDCl_3$): δ 7.47 (dd, *J* = 7.7, 1.5 Hz, 1H, Ar *H*), 7.38 – 7.27 (m, 1H, Ar *H*), 7.20 (td, *J* = 7.4, 1.5 Hz, 1H, Ar *H*), 7.11 (dd, *J* = 7.7, 1.5 Hz, 1H, Ar *H*), 4.14 (q, *J* = 6.9 Hz, 1H, C*H*CH_3), 3.88 (s, 2H, C(O)CH_2), 3.75 – 3.65 (m, 4H, CH_2), 3.61 – 3.35 (m, 4H, CH_2), 2.55 – 2.40 (m, 2H, SCH_2), 1.62 (d, *J* = 6.9 Hz, 3H, CH_3), 1.18 (t, *J* = 7.4 Hz, 3H, CH_3).

^{13}C{^{1}H} NMR (101 MHz, $CDCl_3$): δ 170.0 (*C*=O), 141.3 (Ar *C*), 132.8 (Ar *C*), 129.3 (Ar *C*H), 127.6 (Ar *C*H), 127.3 (Ar *C*H), 127.3 (Ar *C*H), 67.0 (*C*H_2), 66.7 (*C*H_2), 46.6 (*C*H_2), 42.3 (*C*H_2), 39.5 (*C*HCH_3), 38.0 (*C*H_2), 25.1 (S*C*H_2), 21.6 (*C*H_3), 14.8 (*C*H_3).

HRMS: calcd. for $C_{16}H_{24}NO_2S$ [M+H]$^+$: 294.1522; found (ESI$^+$): 294.1524.

ν_{max} (neat/cm^{-1}): 697, 1031, 1223, 1457, 1491, 1640, 2855, 2921.

Ethyl(methyl)(2-morpholino-2-oxo-1-phenylethyl)sulfonium trifluoromethanesulfonate (2v)

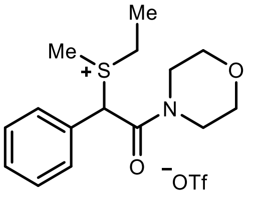

$C_{17}H_{24}F_3NO_5S_2$
MW: 429.47

Synthesised according to **GP-6** from 2-phenyl-1-morpholinoethan-1-one (205 mg, 1.0 mmol), Tf_2O (0.18 mL, 1.1 mmol), and 2-chloropyridine (0.19 mL, 2.0 mmol) in CH_2Cl_2 (3.5 mL) *then* ethyl methyl sulfoxide (230 mg in 0.5 mL CH_2Cl_2, 2.5 mmol) and TFAA (0.14 mL, 1.0 mmol). Purification by column chromatography (silica gel; 0-10% MeOH in EtOAc) afforded the product as an off white solid (219 mg, 0.51 mmol, 51%).

A 1:1 mixture of rotamers was observed at 298 K in $CDCl_3$.

^{1}H NMR (400 MHz, $CDCl_3$): δ 7.67 – 7.58 (m, 2H, Ar *H*), 7.55 – 7.47 (m, 3H, Ar *H*), 6.79 (s, 0.5H, C*H*S(CH_3)(CH_2CH_3)), 6.75 (s, 0.5H, C*H*S(CH_3)(CH_2CH_3)), 3.77 – 3.54 (m, 6H, CH_2 + SCH_2), 3.53 – 3.33 (m, 1.5H, CH_2 + SCH_2), 3.19 – 3.07 (m, 3.4H, CH_2 + SCH_3), 2.89 (dq, *J* = 13.0, 7.6 Hz, 0.5H, SCH_2), 2.62 (s, 1.5H, SCH_3), 1.54 (t, *J* = 7.5 Hz, 1.5H, SCH_2CH_3), 1.24 (t, *J* = 7.5 Hz, 1.5H, SCH_2CH_3).

^{13}C{^{1}H} NMR (101 MHz, $CDCl_3$): δ 163.5 (*C*=O), 163.5 (*C*=O), 131.8 (Ar *C*H), 131.7 (Ar *C*H), 130.7 (Ar *C*H), 130.7 (Ar *C*H), 129.7 (Ar *C*H), 129.6 (Ar *C*H), 127.4 (Ar *C*), 127.3 (Ar *C*), 120.6 (q, *J* = 319.8 Hz, SO_2CF_3), 66.6 (*C*HS(CH_3)(CH_2CH_3)), 66.6 (*C*HS(CH_3)(CH_2CH_3)), 66.3 (CH_2), 66.3 (CH_2), 66.2 (*C*H_2), 66.2 (*C*H_2), 46.6 (*C*H_2), 46.6 (*C*H_2), 43.3 (*C*H_2), 43.3 (*C*H_2), 38.0 (SCH_2), 33.4 (SCH_2), 23.3 (S*C*H_3), 18.9 (S*C*H_3), 10.0 (SCH_2*C*H_3), 9.6 (SCH_2*C*H_3).

^{19}F NMR (376 MHz, $CDCl_3$): δ - 78.39 (s, SO_2CF_3).

HRMS: calcd. for $C_{15}H_{22}NO_2S$ [M-OTf]$^+$: 280.1366; found (ESI$^+$): 280.1360.

ν_{max} (neat/cm^{-1}): 638, 1030, 1257, 1449, 1641, 2860, 2939, 3527.

m.p.: (recrystallized from EtOAc) 146-147 °C.

2-(2-((Ethylthio)methyl)phenyl)-1-morpholinoethan-1-one (3v)

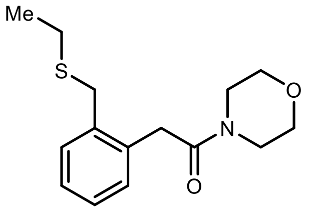

$C_{15}H_{21}NO_2S$
MW: 279.40

Synthesised according to **GP-7** from **2v** (42.9 mg, 0.1 mmol), with sodium *tert*-butoxide (19.2 mg, 0.1 mmol) in CH_2Cl_2 (1.0 mL). Purification by column chromatography (silica gel; 0-50% EtOAc in hexanes) afforded the product as an off yellow oil (19.8 mg, 0.071 mmol, 71%).

^{1}H NMR (400 MHz, CDCl$_3$): δ 7.35 – 7.16 (m, 4H, Ar *H*), 3.96 (s, 2H, C(O)CH_2), 3.77 (s, 2H, SCH_2), 3.75 (br., 4H, CH_2), 3.67 – 3.59 (m, 2H, CH_2), 3.55 – 3.50 (m, 2H, CH_2), 2.52 (q, *J* = 7.4 Hz, 2H, SCH_2CH$_3$), 1.30 (t, *J* = 7.4 Hz, 3H, SCH$_2$CH_3).

^{13}C{^{1}H} NMR (101 MHz, CDCl$_3$): δ 169.8 (*C*=O), 135.9 (Ar *C*), 133.9 (Ar *C*), 130.5 (Ar *C*H), 129.5 (Ar *C*H), 127.8 (Ar *C*H), 127.1 (Ar *C*H), 67.0 (*C*H$_2$), 66.7 (*C*H$_2$), 46.6 (*C*H$_2$), 42.3 (*C*H$_2$), 37.6 (C(O)*C*H$_2$), 34.3 (S*C*H$_2$), 25.8 (S*C*H$_2$CH$_3$), 14.6 (SCH$_2$*C*H$_3$).

HRMS: calcd. for $C_{15}H_{22}NO_2S$ [M+H]$^+$: 280.1366; found (ESI$^+$): 280.1357.

ν_{max} (neat/cm^{-1}): 698, 1035, 1229, 1427, 1490, 1639, 2854, 2921.

1-Morpholino-2-(2-((phenylthio)methyl)phenyl)ethan-1-one (3w)

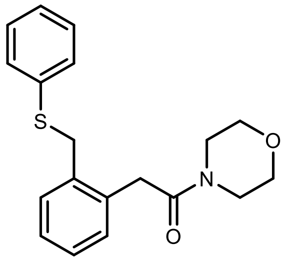

$C_{19}H_{21}NO_2S$
MW: 327.44

Synthesised according to **GP-9**, with potassium hydroxide (11.2 mg, 0.2 mmol) in CH_2Cl_2 (1.0 mL). Purification by column chromatography (silica gel; 0-50% EtOAc in hexanes) afforded the product as an off yellow oil (75.2 mg, 0.23 mmol, 45%).

A 11.2:1 mixture of rotamers was observed at 298 K in $CDCl_3$.

^{1}H NMR (400 MHz, $CDCl_3$): δ 7.51 – 7.45 (m, 0.4H, Ar *H*), 7.32 – 7.26 (m, 3.9H, Ar *H*), 7.24 – 7.21 (m, 1.7H, Ar *H*), 7.17 – 7.14 (m, 2.9H, Ar *H*), 4.13 (s, 0.2H, C(O)CH_2), 4.10 (s, 1.8H, C(O)CH_2), 3.87 (s, 1.8H, SCH_2), 3.75 (s, 0.2H, SCH_2), 3.69 – 3.43 (m, 8H, CH_2).

^{13}C{^{1}H} NMR (101 MHz, $CDCl_3$): δ 169.8 (*C*=O), 169.7 (*C*=O), 136.1 (Ar *C*), 136.1 (Ar *C*), 135.4 (Ar *C*), 135.1 (Ar *C*), 133.9 (Ar *C*), 133.9 (Ar *C*), 132.3 (Ar *C*H), 131.4 (Ar *C*H), 130.8 (Ar *C*H), 130.4 (Ar *C*H), 130.4 (Ar *C*H), 130.1 (Ar *C*H), 129.6 (Ar *C*H), 129.2 (Ar *C*H), 129.1 (Ar *C*H), 128.9 (Ar *C*H), 128.2 (Ar *C*H), 127.4 (Ar *C*H), 126.9 (Ar *C*H), 124.5 (Ar *C*H), 67.0 (*C*H_2), 66.8 (*C*H_2), 66.7 (*C*H_2), 61.4 (*C*H_2), 46.6 (*C*H_2), 46.5 (*C*H_2), 42.5 (*C*H_2), 42.3 (*C*H_2), 38.4 (C(O)*C*H_2), 38.2 (C(O)*C*H_2), 37.7 (S*C*H_2), 37.6 (S*C*H_2).

HRMS: calcd. for $C_{19}H_{22}NO_2S$ $[M+H]^+$: 328.1366; found (ESI^+): 328.1356.

ν_{max} (neat/cm^{-1}): 691, 1036, 1230, 1479, 1643, 2854, 2963.

1-(2-Morpholino-2-oxo-1-phenylethyl)tetrahydro-1*H*-thiophen-1-ium trifluoromethanesulfonate (2x)

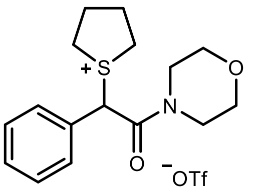

$C_{17}H_{22}F_3NO_5S_2$
MW: 441.48

Synthesised according to **GP-6** from 2-phenyl-1-morpholinoethan-1-one (205 mg, 1.0 mmol), Tf_2O (0.18 mL, 1.1 mmol), and 2-chloropyridine (0.19 mL, 2.0 mmol) in CH_2Cl_2 (3.5 mL) *then* tetrahydrothiophene *S*-oxide (260 mg in 0.5 mL CH_2Cl_2, 2.5 mmol) and TFAA (0.14 mL, 1.0 mmol). Purification by column chromatography (silica gel; 0-10% MeOH in EtOAc) afforded the product as an off white solid (337 mg, 0.76 mmol, 76%).

^{1}H NMR (400 MHz, CD_3CN): δ 7.63 – 7.57 (m, 2H, Ar *H*), 7.54 – 7.50 (m, 3H, Ar *H*), 6.65 (s, 1H, C*H*S(CH_2)$_2$), 3.79 – 3.47 (m, 8H, 3 x CH_2, 2 x CH_{2A}), 3.35 – 3.25 (m, 1H, 1 x CH_{2A}), 3.14 – 3.02 (m, 3H, 3 x CH_{2B}), 2.49 – 2.37 (m, 2H, CH_2), 2.24 – 2.07 (m, 2H, CH_2).

^{13}C{^{1}H} NMR (101 MHz, CD_3CN): δ 164.3 (*C*=O), 131.7 (Ar *C*H), 130.8 (Ar *C*H), 129.6 (Ar *C*H), 128.4 (Ar *C*), 120.5 (q, *J* = 319.4 Hz, *C*F_3), 67.0 (*C*HS(CH_2)$_2$), 66.3 (*C*H_2), 66.0 (*C*H_2), 46.6 (*C*H_2), 44.8 (*C*H_2), 43.4 (*C*H_2), 39.2 (*C*H_2), 28.57 (*C*H_2), 28.55 (*C*H_2).

^{19}F NMR (376 MHz, CD_3CN): δ -78.39 (s, SO_2C*F_3*).

HRMS: calcd. for $C_{16}H_{22}NO_2S$ [M-OTf]$^+$: 292.1366; found (ESI+): 292.1371.

ν_{max} (neat/cm^{-1}): 638, 1030, 1256, 1448, 1639, 1726, 2861, 2946, 3520.

m.p.: (recrystallized from EtOAc) 135-138 °C.

1-Morpholino-2-(2-(tetrahydrothiophen-2-yl)phenyl)ethan-1-one (3x)

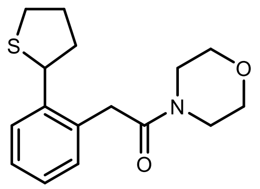

$C_{16}H_{21}NO_2S$
MW: 291.41

Synthesised according to **GP-7** from **2x** (44.1 mg, 0.1 mmol), with sodium *tert*-butoxide (19.2 mg, 0.2 mmol) in CH_2Cl_2 (1.0 mL). Purification by column chromatography (silica gel; 0-50% EtOAc in hexanes) afforded the product as a yellow oil (27.6 mg, 0.095 mmol, 95%).

^{1}H NMR (400 MHz, CDCl$_3$): δ 7.60 (dd, *J* = 7.9, 1.4 Hz, 1H, Ar *H*), 7.34 – 7.22 (m, 1H, Ar *H*), 7.18 (td, *J* = 7.5, 1.5 Hz, 1H, Ar *H*), 7.10 (dd, *J* = 7.7, 1.5 Hz, 1H, Ar *H*), 4.62 (dd, *J* = 8.3, 5.7 Hz, 1H, SC*H*), 3.82 (d, *J* = 7.6 Hz, 2H, CH_2), 3.71 – 3.39 (m, 8H, CH_2 + C(O)CH_2), 3.16 (ddd, *J* = 11.5, 8.9, 6.2 Hz, 1H, CH_2), 3.02 (ddd, *J* = 9.9, 6.4, 3.8 Hz, 1H, CH_2), 2.41 – 2.25 (m, 2H, CH_2), 2.10 – 1.93 (m, 2H, CH_2).

^{13}C{^{1}H} NMR (101 MHz, CDCl$_3$): δ 169.9 (*C*=O), 140.8 (Ar *C*), 133.2 (Ar *C*), 129.3 (Ar *C*H), 127.7 (Ar *C*H), 127.5 (Ar *C*H), 127.3 (Ar *C*H), 67.0 (*C*H$_2$), 66.7 (*C*H$_2$), 48.6 (*C*H$_2$), 46.6, (*C*H$_2$) 42.4 (*C*H$_2$), 38.6 (C(O)*C*H$_2$), 38.2 (S*C*H$_2$), 33.6 (S*C*H), 31.1 (*C*H$_2$).

HRMS: calcd. for $C_{16}H_{22}NO_2S$ [M+H]$^+$: 292.1366; found (ESI$^+$): 292.1358.

ν_{max} (neat/cm^{-1}): 695, 1035, 1230, 1437, 1488, 1641, 2856, 2923.

1-(2-Morpholino-2-oxo-1-phenylethyl)hexahydrothiopyrylium trifluoromethanesulfonate (2y)

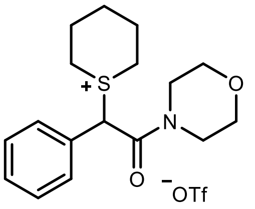

$C_{18}H_{24}F_3NO_5S_2$
MW: 455.51

Synthesised according to **GP-6** from 2-phenyl-1-morpholinoethan-1-one (205 mg, 1.0 mmol), Tf_2O (0.18 mL, 1.1 mmol), and 2-chloropyridine (0.19 mL, 2.0 mmol) in CH_2Cl_2 (3.5 mL) *then* tetrahydro-2*H*-thiopyran 1-oxide (295 mg in 0.5 mL CH_2Cl_2, 2.5 mmol) and TFAA (0.14 mL, 1.0 mmol). Purification by column chromatography (silica gel; 0-10% MeOH in EtOAc) afforded the product as a yellow solid (191 mg, 0.420 mmol, 42%).

^{1}H NMR (400 MHz, $CDCl_3$): δ 7.60 – 7.58 (m, 2H, Ar *H*), 7.49 – 7.48 (m, 3H, Ar *H*), 6.74 (s, 1H, SC*H*), 3.83 – 3.76 (m, 1H, C*H*$_2$), 3.71 – 3.52 (m, 7H, C*H*$_2$), 3.48 – 3.43 (m, 1H, C*H*$_2$), 3.16 – 3.05 (m, 2H, C*H*$_2$), 2.58 – 2.54 (m, 1H, C*H*$_2$), 2.34 – 2.24 (m, 2H, C*H*$_2$), 1.87 – 1.56 (m, 4H, C*H*$_2$)

^{13}C{^{1}H} NMR (101 MHz, $CDCl_3$): δ 163.2 (*C*=O), 131.5 (Ar *C*H), 130.5 (Ar *C*H), 129.39 (Ar *C*H), 127.1 (Ar *C*), 120.6 (q, J = 319.1 Hz, *C*F$_3$), 66.6 (*C*H), 66.3 (*C*H$_2$), 66.1 (*C*H$_2$), 46.4 (*C*H$_2$), 43.2 (*C*H$_2$), 39.0 (*C*H$_2$), 34.6 (*C*H$_2$), 23.6 (*C*H$_2$), 23.3 (*C*H$_2$), 22.8 (*C*H$_2$).

^{19}F NMR (376 MHz, $CDCl_3$): δ -78.38 (s, SO_2CF_3).

HRMS: calcd. for $C_{16}H_{22}NO_2S$ [M-OTf]$^+$: 306.1522; found (ESI$^+$): 306.1522.

ν_{max} (neat/cm^{-1}): 645, 1028, 1270, 1466, 1678, 1729, 2863, 2949, 3527.

m.p.: (recrystallized from EtOAc) 143-146 °C.

1-Morpholino-2-(2-(tetrahydro-2*H*-thiopyran-2-yl)phenyl)ethan-1-one (3y)

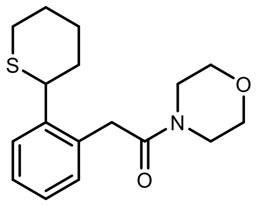

$C_{17}H_{23}NO_2S$
MW: 305.44

Synthesised according to **GP-8** from **2y** (91.0 mg, 0.2 mmol), with potassium hydroxide (22.4 mg, 0.4 mmol) in acetonitrile (2.0 mL). Purification by column chromatography (silica gel; 0-50% EtOAc in hexanes) afforded the product as a yellow oil (33.6 mg, 0.110 mmol, 55%).

^{1}H NMR (400 MHz, CDCl$_3$): δ 7.43 – 7.41 (m, 1H, Ar *H*), 7.27 – 7.24 (m, 1H, Ar *H*), 7.22 – 7.18 (m, 1H, Ar *H*), 7.13 – 7.10 (m, 1H, Ar *H*), 3.99 – 3.86 (m, 3H, C(O)CH_2 + C*H*), 3.72 – 3.64 (m, 4H, CH_2), 3.60 – 3.55 (m, 1H, CH_2), 3.52 – 3.39 (m, 3H, CH_2), 2.91 – 2.84 (m, 1H, CH_2), 2.68 – 2.62 (m, 1H, CH_2), 2.10 – 2.01 (m, 4H, CH_2), 1.75 – 1.65 (m, 2H, CH_2).

^{13}C{^{1}H} NMR (101 MHz, CDCl$_3$): δ 170.1 (*C*=O), 140.6 (Ar *C*), 132.9 (Ar *C*), 129.0 (Ar *C*H), 127.7 (Ar *C*H), 127.6 (Ar *C*H), 127.4 (Ar *C*H), 67.0 (*C*H$_2$), 66.7 (*C*H$_2$), 46.7 (*C*H$_2$), 43.4 (*C*H), 42.4 (*C*H$_2$), 37.9 (C(O)*C*H$_2$), 33.8 (*C*H$_2$), 31.3 (*C*H$_2$), 27.5 (*C*H$_2$), 27.0 (*C*H$_2$).

HRMS: calcd. for $C_{17}H_{24}NO_2S$ [M+H]$^+$: 306.1522; found (ESI$^+$): 306.1528.

ν_{max} (neat/cm^{-1}): 662, 1039, 1244, 1445, 1496, 1644, 2855, 2930.

4-(2-Morpholino-2-oxo-1-phenylethyl)-1,4-oxathian-4-ium trifluoromethanesulfonate (2z)

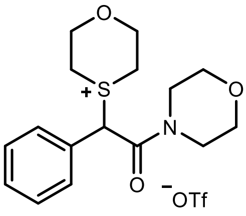

$C_{17}H_{22}F_3NO_6S_2$
MW: 457.48

Synthesised according to **GP-6** from 2-phenyl-1-morpholinoethan-1-one (205 mg, 1.0 mmol), Tf_2O (0.18 mL, 1.1 mmol), and 2-chloropyridine (0.19 mL, 2.0 mmol) in CH_2Cl_2 (3.5 mL) *then* 1,4-oxathiane 4-oxide (300 mg in 0.5 mL CH_2Cl_2, 2.5 mmol) and TFAA (0.14 mL, 1.0 mmol). Purification by column chromatography (silica gel; 0-5% MeOH in EtOAc) afforded the product as a white solid (188 mg, 0.41 mmol, 41%).

^{1}H NMR (400 MHz, $CDCl_3$): δ 7.63 – 7.60 (m, 2H, Ar *H*), 7.56 – 7.53 (m, 3H, Ar *H*), 7.09 (s, 1H, SC*H*), 3.86 – 3.80 (m, 2H, C*H*$_2$), 3.76 – 3.70 (m, 3H, C*H*$_2$), 3.67 – 3.60 (m, 5H, C*H*$_2$), 3.50 – 3.45 (m, 1H, C*H*$_2$), 3.21 – 3.17 (m, 1H, C*H*$_2$), 3.09 – 3.03 (m, 1H, C*H*$_2$), 2.96 – 2.89 (m, 1H, C*H*$_2$), 2.78 – 2.70 (m, 2H, C*H*$_2$).

^{13}C{^{1}H} NMR (101 MHz, $CDCl_3$): δ 163.4 (*C*=O), 132.1 (Ar *C*H), 130.8 (Ar *C*H), 129.9 (Ar *C*H), 125.9 (Ar *C*), 120.6 (q, *J* = 319.6 Hz), 67.5 (*C*H), 66.3 (*C*H$_2$), 66.2 (*C*H$_2$), 64.6 (*C*H$_2$), 64.4 (*C*H$_2$), 46.7 (*C*H$_2$), 43.3 (*C*H$_2$), 36.9 (*C*H$_2$), 33.5 (*C*H$_2$).

^{19}F NMR (376 MHz, CD_3CN): δ - 78.31 (s, SO_2CF_3).

HRMS: calcd. $C_{16}H_{22}NO_3S$ [M-OTf]$^+$: 309.1315; found (ESI$^+$): 309.1318.

v_{max} (neat/cm^{-1}): 639, 1032, 1258, 1448, 1643, 1729, 2861, 2949, 3522.

m.p.: (recrystallized from EtOAc) 132-136 °C.

2-(2-(1,4-Oxathian-3-yl)phenyl)-1-morpholinoethan-1-one (3z)

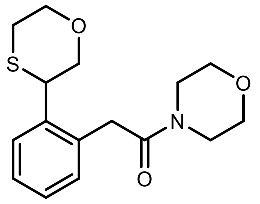

$C_{16}H_{21}NO_3S$
MW: 307.41

Synthesised according to **GP-8** from **2z** (91.4 mg, 0.2 mmol), with potassium hydroxide (22.4 mg, 0.4 mmol) in acetonitrile (2.0 mL). Purification by column chromatography (silica gel; 0-20% EtOAc in hexanes) afforded the product as an off yellow oil (50.2 mg, 0.164 mmol, 82%).

^{1}H NMR (400 MHz, $CDCl_3$): δ 7.45 – 7.43 (m, 1H, Ar *H*), 7.28 – 7.21 (m, 2H, Ar *H*), 7.16 – 7.13 (m, 1H, Ar *H*), 4.23 – 4.10 (m, 3H, C*H₂*), 3.92 – 3.49 (m, 12H, C(O)CH_2 + C*H* + CH_2), 3.11 – 3.04 (m, 1H, CH_2), 2.53 – 2.48 (m, 1H, CH_2).

^{13}C{^{1}H} NMR (101 MHz, $CDCl_3$): δ 169.5 (*C*=O), 137.0 (Ar *C*), 133.5 (Ar *C*), 129.8 (Ar *C*H), 128.0 (Ar *C*H), 127.9 (Ar *C*H), 127.7 (Ar *C*H), 73.5 (C(O)*C*H_2), 68.3 (*C*H_2), 66.9 (*C*H_2), 66.6 (*C*H_2), 46.5 (*C*H_2), 42.3 (*C*H_2), 40.1 (*C*H_2), 37.7 (*C*H), 28.6 (*C*H_2).

HRMS: calcd. for $C_{16}H_{21}NO_3S$ $[M+H]^+$: 308.1242; found (APCI$^+$): 308.1243.

ν_{max} (neat/cm^{-1}): 679, 1044, 1251, 1455, 1494, 1639, 2855, 2930.

4-Cyano-1-(2-morpholino-2-oxo-1-phenylethyl)hexahydrothiopyrylium trifluoromethanesulfonate (2aa)

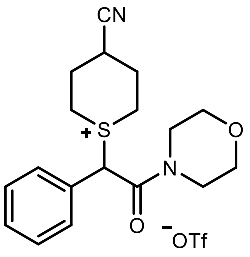

$C_{19}H_{23}F_3N_2O_5S_2$
MW: 480.52

Synthesised according to **GP-6** from 2-phenyl-1-morpholinoethan-1-one (205 mg, 1.0 mmol), Tf_2O (0.18 mL, 1.1 mmol), and 2-chloropyridine (0.19 mL, 2.0 mmol) in CH_2Cl_2 (3.5 mL) *then* tetrahydro-2*H*-thiopyran-4-carbonitrile 1-oxide (357 mg in 0.5 mL CH_2Cl_2, 2.5 mmol) and TFAA (0.14 mL, 1.0 mmol). Purification by column chromatography (silica gel; 5-10% MeOH in EtOAc) afforded the product as an oil (197 mg, 0.41 mmol, 41%).

^{1}H NMR (400 MHz, $CDCl_3$): δ 7.61 – 7.59 (m, 2H, Ar *H*), 7.55 – 7.53 (m, 3H, Ar *H*), 6.83 (s, 1H, SC*H*), 4.15 – 4.07 (m, 1H, CH_2), 3.97 – 3.90 (m, 1H, CH_2), 3.79 – 3.46 (m, 7H, CH_2), 3.29 – 3.21 (m, 1H, C*H*CN), 3.17 – 3.06 (m, 2H, CH_2), 2.78 – 2.60 (m, 3H, CH_2), 2.18 – 2.07 (m, 1H, CH_2), 2.01 – 1.90 (m, 1H, CH_2).

^{13}C{^{1}H} NMR (101 MHz, $CDCl_3$): δ 162.8 (*C*=O), 132.1 (Ar *C*H), 130.9 (Ar *C*H), 129.3 (Ar *C*H), 126.5 (Ar *C*), 120.5 (q, *J* = 319.7 Hz, *C*F_3), 119.15 (*C*N), 67.9 (*C*H), 66.3 (*C*H_2), 66.0 (*C*H_2), 46.5 (*C*H_2), 43.4 (*C*H_2), 37.2 (*C*H_2), 32.4 (*C*H_2), 26.8 (*C*HCN), 26.4 (*C*H_2), 25.3 (*C*H_2).

^{19}F NMR (376 MHz, $CDCl_3$): δ - 78.39 (s, SO_2CF_3).

HRMS: calcd. $C_{18}H_{23}N_2O_2S$ [M-OTf]$^+$: 331.1475; found (APCI$^+$): 331.1475.

ν_{max} (neat/cm^{-1}): 639, 1032, 1251, 1449, 1643, 1728, 2861, 2947, 3521.

2-(2-(2-Morpholino-2-oxoethyl)phenyl)tetrahydro-2H-thiopyran-4-carbonitrile (3aa)

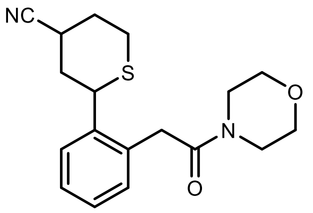

$C_{18}H_{22}N_2O_2S$
MW: 330.45

Synthesised according to **GP-8** from **2aa** (96.0 mg, 0.2 mmol), with potassium hydroxide (22.4 mg, 0.4 mmol) in acetonitrile (2.0 mL). Purification by column chromatography (silica gel; 0-20% EtOAc in hexanes) afforded the product as an off yellow oil (50.8 mg, 0.154 mmol, 77%).

^{1}H NMR (400 MHz, $CDCl_3$): δ 7.40 – 7.38 (m, 1H, Ar *H*), 7.30 – 7.21 (m, 2H, Ar *H*), 7.13 – 7.11 (m, 1H, Ar *H*), 4.07 – 4.04 (m, 1H, SC*H*), 3.84 (s, 2H, CH_2C(O)), 3.70 – 3.46 (m, 8H, CH_2), 2.92 – 2.84 (m, 1H, CH_2), 2.77 – 2.67 (m, 2H, C*H*CN + CH_2), 2.48 – 2.40 (m, 2H, CH_2), 2.33 – 2.24 (m, 1H, CH_2), 2.03 – 1.92 (m, 1H, CH_2).

^{13}C{^{1}H} NMR (101 MHz, $CDCl_3$): δ 169.6 (*C*=O), 139.1 (Ar *C*), 132.8 (Ar *C*), 130.0 (Ar *C*H), 128.2 (Ar *C*H), 128.1 (Ar *C*H), 127.4 (Ar *C*H), 121.4 (*C*N), 66.9 (*C*H_2), 66.6 (*C*H_2), 46.6 (*C*H_2), 42.4 (*C*H_2), 42.0 (S*C*H), 38.4 (*C*H_2C(O)), 36.9 (*C*H_2), 30.2 (*C*H_2), 29.7 (*C*H_2), 29.7 (*C*HCN).

HRMS: calcd. for $C_{18}H_{22}N_2O_2S$ $[M+H]^+$: 331.1402; found (APCI$^+$): 331.1403.

ν_{max} (neat/cm^{-1}): 630, 1033, 1241, 1455, 1493, 1638, 2830, 2928.

(1-(2-Chlorophenyl)-2-(1-methyl-3,4-dihydroisoquinolin-2(1H)-yl)-2-oxoethyl)dimethylsulfonium trifluoromethanesulfonate (2ab)

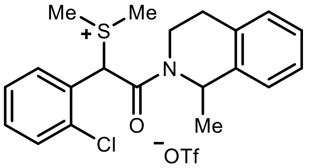

$C_{21}H_{23}ClF_3NO_4S_2$
MW: 509.98

Synthesised according to **GP-6** from 2-(2-chlorophenyl)-1-(1-methyl-3,4-dihydroisoquinolin-2(1H)-yl)ethan-1-one (899 mg, 3.0 mmol), Tf_2O (0.54 mL, 3.3 mmol), and 2-chloropyridine (0.57 mL, 6.0 mmol) in CH_2Cl_2 (10.5 mL) *then* DMSO (0.54 mL in 1.5 mL CH_2Cl_2, 7.5 mmol) and TFAA (0.52 mL, 3.0 mmol). Purification by column chromatography (silica gel; 0-10% MeOH in EtOAc) afforded the product as an off white solid (994 mg, 1.95 mmol, 65%).

A 2:1 mixture of rotamers was observed at 298 K in $CDCl_3$.

^{1}H NMR (400 MHz, $CDCl_3$): δ 7.66 – 7.36 (m, 4H, Ar *H*), 7.21 – 7.08 (m, 3H, Ar *H*), 6.99 – 6.78 (m, 2H, Ar *H* + C*H*S(CH_3)$_2$), 5.58 (q, *J* = 6.8 Hz, 0.3H, C*H*CH_3), 5.46 (q, *J* = 6.8 Hz, 0.7H, C*H*CH_3), 3.78 – 3.51 (m, 1.9H, CH_2), 3.29 – 3.20 (m, 3.5H, CH_2 + SCH_3), 2.80 – 2.74 (m, 3.7H, CH_2 + SCH_3), 2.59 – 2.49 (m, 0.8H, CH_2), 1.82 – 1.72 (m, 0.9H, CH_2), 1.71 – 1.63 (m, 0.5H, CH_2), 1.52 (d, *J* = 6.8 Hz, 2H, CHCH_3), 1.34 (d, *J* = 6.8 Hz, 1H, CHCH_3).

^{13}C{^{1}H} NMR (101 MHz, $CDCl_3$): δ 163.2 (*C*=O), 162.9 (*C*=O), 137.2 (Ar *C*), 136.4 (Ar *C*), 136.3 (Ar *C*), 135.4 (Ar *C*), 135.3 (Ar *C*), 133.2 (Ar *C*), 133.0 (Ar *C*), 133.0 (Ar *C*), 132.9 (Ar *C*H), 132.7 (Ar *C*H), 131.8 (Ar *C*H), 131.5 (Ar *C*H), 131.4 (Ar *C*H), 129.0 (Ar *C*H), 129.0 (Ar *C*H), 128.7 (Ar *C*H), 127.0 (Ar *C*H), 127.0 (Ar *C*H), 126.9 (Ar *C*H), 126.8 (Ar *C*H), 126.8 (Ar *C*H), 126.7 (Ar *C*H), 125.8 (Ar *C*H), 125.8 (Ar *C*H), 120.7 (d, *J* = 319.8 Hz, SO_2CF_3), 64.8 (*C*HS(CH_3)$_2$), 64.6 (*C*HS(CH_3)$_2$), 51.0 (*C*HCH_3), 49.9 (*C*HCH_3), 40.8 (*C*H_2), 40.0 (*C*H_2), 28.8 (*C*H_2), 28.2 (*C*H_2), 28.1 (S*C*H_3), 26.5 (S*C*H_3), 23.2 (S*C*H_3), 22.3 (S*C*H_3), 21.7 (CH*C*H_3), 21.3 (CH*C*H_3).

^{19}F NMR (376 MHz, $CDCl_3$): δ - 78.60 (s, SO_2CF_3).

HRMS: calcd. for $C_{20}H_{23}ClNOS$ [M-OTf]$^+$: 360.1183; found (ESI$^+$): 360.1174.

v_{max} (neat/cm^{-1}): 638, 1030, 1257, 1438, 1638, 1719, 2931, 3525.

m.p.: (recrystallized from EtOAc) 146-147 °C.

2-(2-Chloro-6-((methylthio)methyl)phenyl)-1-(1-methyl-3,4-dihydroisoquinolin-2(1H)-yl)ethan-1-one (3ab)

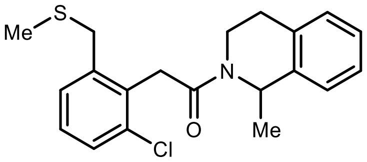

$C_{20}H_{23}NClOS$
MW: 359.91

Synthesised according to **GP-7** from **2ab** (50.9 mg, 0.1 mmol), with sodium *tert*-butoxide (19.2 mg, 0.2 mmol) in CH_2Cl_2 (1.0 mL). Purification by column chromatography (silica gel; 0-50% EtOAc in hexanes) afforded the product as an off yellow oil (24.8 mg, 0.069 mmol, 69%).

A 1.5:1 mixture of rotamers was observed at 298 K in $CDCl_3$.

^{1}H NMR (400 MHz, $CDCl_3$): δ 7.35 – 7.09 (m, 7H, Ar *H*), 5.66 (q, *J* = 6.8 Hz, 0.6H, CH_3CH), 5.26 (q, *J* = 6.8 Hz, 0.4H, CH_3CH), 4.76 – 4.63 (m, 0.4H, $CH_2C(O)$ + CH_2), 4.25 – 4.03 (m, 2.6H, SCH_2 + CH_2), 3.77 – 3.63 (m, 2.5H, CH_2), 3.18 – 2.88 (m, 2H, CH_2), 2.83 – 2.73 (m, 0.4H, CH_2), 2.03 – 1.98 (m, 3H, CH_3), 1.68 (d, *J* = 6.7 Hz, 1.2H, CH_3), 1.48 (d, *J* = 6.8 Hz, 1.8H, CH_3).

^{13}C{^{1}H} NMR (101 MHz, $CDCl_3$): δ 167.7 (*C*=O), 167.6 (*C*=O), 138.9 (Ar *C*), 137.7 (Ar *C*), 135.9 (Ar *C*), 135.8 (Ar *C*), 134.5 (Ar *C*), 133.4 (Ar *C*), 133.3 (Ar *C*), 133.2 (Ar *C*), 129.3 (Ar *C*H), 129.3 (Ar *C*H), 129.0 (Ar *C*H), 129.0 (Ar *C*H), 128.6 (Ar *C*H), 128.5 (Ar *C*H), 128.5 (Ar *C*H), 127.7 (Ar *C*H), 127.7 (Ar *C*H), 127.3 (Ar *C*H), 127.2 (Ar *C*), 126.9 (Ar *C*H), 126.7 (Ar *C*H), 126.6 (Ar *C*), 126.5 (Ar *C*H), 126.4 (Ar *C*H), 52.2 (CH_3CH), 49.2 (CH_3CH), 40.2 (CH_2), 37.1 (CH_2), 35.5 (CH_2), 35.5 (CH_2), 34.3 (CH_2), 34.2 (CH_2), 29.6 (CH_2), 28.7 (CH_2), 23.0 (CH_3), 21.8 (CH_3), 15.4 (CH_3), 15.3 (CH_3).

HRMS: calcd. for $C_{20}H_{23}ClNOS$ $[M+H]^+$: 360.1183; found (ESI$^+$): 360.1179.

ν_{max} (neat/cm^{-1}): 670, 1045, 1251, 1450, 1488, 1646, 1672, 2833, 2917.

2-(2-Chloro-6-((methylsulfonyl)methyl)phenyl)-1-(1-methyl-3,4-dihydroisoquinolin-2(1H)-yl)ethan-1-one (I)

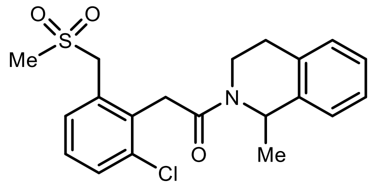

$C_{20}H_{23}NClO_3S$
MW: 391.91

To a solution of **3ab** (0.1 mmol, 1 equiv.) and H_2O_2 (30 wt %, 4 equiv.), $TiCl_4$ (1 equiv.) at 0 °C was added dropwise. The mixture was stirred at room temperature. After 0.5 h, the reaction was quenched by the addition of saturated NH_4Cl (3 mL) and the CH_2Cl_2 layer was separated. The aqueous layer was extracted with CH_2Cl_2 (3 × 10 mL) and the combined organic layers were washed with saturated aqueous $NaHCO_3$ (50 mL), dried over Na_2SO_4, and concentrated *in vacuo*. Purification by column chromatography (silica gel; 0-50% EtOAc in Hexane) afforded the product **(I)** as a colourless oil (34.5 mg, 0.088 mmol, 88%).

A 1.5:1 mixture of rotamers was observed at 298 K in $CDCl_3$.

^{1}H NMR (400 MHz, $CDCl_3$): δ 7.47 – 7.39 (m, 1H, Ar *H*), 7.34 – 7.26 (m, 1.4H, Ar *H*), 7.26 – 7.11 (m, 4.6H, Ar *H*), 5.65 – 5.56 (m, 0.5H, CH_3CH), 5.31 – 5.20 (m, 0.5H, CH_3CH), 4.64 (ddd, *J* = 13.0, 5.8, 2.7 Hz, 0.4H, CH_2), 4.52 – 4.37 (m, 1.8H, CH_2C(O) + CH_2), 4.35 – 4.03 (m, 2.7H, CH_2C(O) + CH_2), 3.74 – 3.63 (m, 0.7H, CH_2), 3.18 – 3.01 (m, 1.2H, CH_2), 2.99 – 2.86 (m, 3.7H, SCH_3 + CH_2), 2.79 (dt, *J* = 16.4, 3.5 Hz, 0.5H, CH_2), 2.62 – 2.53 (m, 0.3H, CH_2), 1.68 (d, *J* = 6.8 Hz, 1.2H, CH_3), 1.47 (d, *J* = 6.8 Hz, 1.8H, CH_3).

^{13}C{^{1}H} NMR (101 MHz, $CDCl_3$): δ 167.6 (*C*=O), 167.6 (*C*=O), 140.0 (Ar *C*), 138.6 (Ar *C*), 137.6 (Ar *C*), 136.2 (Ar *C*), 135.4 (Ar *C*), 135.3 (Ar *C*), 134.2 (Ar *C*), 133.4 (Ar *C*), 130.8 (Ar *C*H), 130.7 (Ar *C*H), 130.6 (Ar *C*H), 130.5 (Ar *C*H), 130.2 (Ar *C*H), 130.0 (Ar *C*H), 129.3 (Ar *C*H), 128.8 (Ar *C*H), 128.4 (Ar *C*), 128.4 (Ar *C*), 127.3 (Ar *C*H), 127.1 (Ar *C*H), 127.0 (Ar *C*H), 126.8 (Ar *C*H), 126.7 (Ar *C*H), 126.6 (Ar *C*H), 59.4 (CH_3*C*H), 59.2 (CH_3*C*H), 52.4 (*C*H_2), 52.4 (*C*H_2), 49.4 (*C*H_2), 48.8 (*C*H_2), 40.4 (*C*H_3), 40.3 (*C*H_3), 34.8 (*C*H_2), 34.8 (*C*H_2), 29.6 (*C*H_2), 28.7 (*C*H_2), 23.2 (*C*H_3), 21.9 (*C*H_3).

HRMS: calcd. for $C_{20}H_{24}ClNO_3S$ $[M+H]^+$: 392.1082; found (ESI^+): 392.1078.

ν_{max} (neat/cm^{-1}): 669, 1041, 1241, 1446, 1492, 1636, 1687, 2930, 2975.

2-(2-((Methylthio)methyl)phenyl)ethan-1-ol (4)

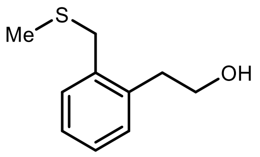

$C_{10}H_{14}OS$
MW: 182.28

An oven-dried microwave vial was charged with sulfonium salt **3a** (0.1 mmol) and lithium aluminium hydride (0.2 mmol) and then sealed and evacuated and back-filled with nitrogen 3 times. Anhydrous THF (1.0 mL, 0.1 M) was then added and the reaction was stirred at 0 °C for 1 h, before warming and stirring for 2 h at room temperature. The crude product mixture was filtered through a syringe filter. Purification by column chromatography (silica gel; 0-50% EtOAc in Hexane) afforded the product **4** as a colourless oil (13.4 mg, 0.074 mmol, 74%).

^{1}H NMR (400 MHz, CDCl$_3$): δ 7.33 – 7.15 (m, 4H, Ar *H*), 3.92 (t, *J* = 6.7 Hz, 2H, ArC*H*$_2$), 3.75 (s, 2H, SC*H*$_2$), 3.00 (t, *J* = 6.6 Hz, 2H, OC*H*$_2$), 2.05 (s, 3H, C*H*$_3$).

^{13}C{^{1}H} NMR (101 MHz, CDCl$_3$): δ 137.2 (Ar *C*), 136.3 (Ar *C*), 130.6 (Ar *C*H), 130.5 (Ar *C*H), 127.7 (Ar *C*H), 126.6 (Ar *C*H), 63.6 (ArC*H*$_2$), 36.1 (SC*H*$_2$), 35.6 (OC*H*$_2$), 15.4 (C*H*$_3$).

HRMS: calcd. for $C_{10}H_{14}OSNa$ [M+Na]$^+$: 205.0658; found (ESI$^+$): 205.0656.

ν_{max} (neat/cm^{-1}): 672, 1043, 1241, 1450, 1489, 1638, 1685, 2875, 2915.

2-(2-((Methylthio)methyl)phenyl)-1-morpholinopropan-1-one (5)

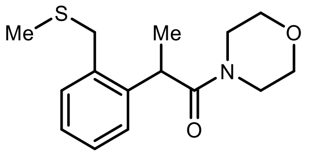

$C_{15}H_{21}NO_2S$
MW: 279.40

To a solution of **3a** (0.1 mmol, 1 equiv.) in anhydrous THF (1.0 mL, 0.1 M) at -78 °C was added *n*-butyllithium (1.2 equiv.) dropwise. After 1 h, iodomethane (1.5 equiv.) was added dropwise and the reaction was stirred at room temperature for 2 h. The reaction was quenched by the addition of saturated NH_4Cl (3 mL) and the CH_2Cl_2 layer was separated. The aqueous layer was extracted with CH_2Cl_2 (3 × 10 mL) and the combined organic layers were washed with saturated aqueous $NaHCO_3$ (50 mL), dried over Na_2SO_4, and concentrated *in vacuo*. Purification by column chromatography (silica gel; 0-50% EtOAc in Hexane) afforded the product **5** as a colourless oil (25.9 mg, 0.093 mmol, 93%).

^{1}H NMR (400 MHz, $CDCl_3$): δ 7.25 – 7.13 (m, 4H, Ar *H*), 4.20 (q, *J* = 6.8 Hz, 1H, C*H*CH_3), 3.84 – 3.06 (m, 10H, CH_2 + SCH_2), 2.04 (s, 3H, SCH_3), 1.43 (d, *J* = 6.8 Hz, 3H, CH_3).

^{13}C{^{1}H} NMR (101 MHz, $CDCl_3$): δ 172.6 (*C*=O), 140.6 (Ar *C*), 133.4 (Ar *C*), 131.3 (Ar *C*H), 128.4 (Ar *C*H), 127.4 (Ar *C*H), 126.9 (Ar *C*H), 66.9 (*C*H_2), 66.4 (*C*H_2), 46.1 (*C*H_2), 42.6 (*C*H_2), 39.4 (C(O)*C*H), 36.0 (S*C*H_2), 19.8 (S*C*H_3), 15.5 (*C*H_3).

HRMS: calcd. for $C_{15}H_{21}NO_2SNa$ $[M+H]^+$: 302.1185; found (ESI$^+$): 302.1177.

ν_{max} (neat/cm^{-1}): 671, 1027, 1230, 1426, 1488, 1639, 1744, 2853, 2915.

2-(2-((Methylsulfinyl)methyl)phenyl)-1-morpholinoethan-1-one (6)

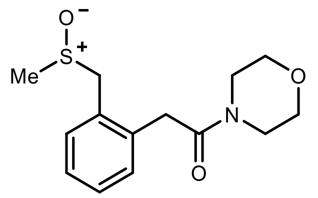

$C_{14}H_{19}NO_3S$
MW: 281.37

To a solution of **3a** (0.1 mmol, 1 equiv.) in glacial acetic acid (5.4 equiv.) at 0 °C, H_2O_2 (30 wt%1.3 equiv.) was added dropwise. After 3 h, the reaction mixture was diluted with CH_2Cl_2 (5 mL) and filtered through Na_2SO_4. The acetic acid was then removed under reduced pressure. Purification by column chromatography (silica gel; 0-10% MeOH in EtOAc) afforded the product **6** as a white solid (25 mg, 0.090 mmol, 90%).

^{1}H NMR (400 MHz, CD_3CN): δ 7.33 – 7.26 (m, 3H, Ar *H*), 7.22 – 7.15 (m, 1H, Ar *H*), 4.05 (d, *J* = 13.3 Hz, 1H, SCH_2), 3.94 (d, *J* = 13.3 Hz, 1H, SCH_2), 3.91 – 3.78 (m, 2H, CH_2), 3.64 – 3.46 (m, 8H, CH_2), 2.51 (s, 3H, SCH_3).

^{13}C{^{1}H} NMR (101 MHz, CD_3CN): δ 170.2 (*C*=O), 137.2 (Ar *C*), 132.5 (Ar *C*H), 132.2 (Ar *C*), 131.4 (Ar *C*H), 129.1 (Ar *C*H), 128.0 (Ar *C*H), 67.4 (*C*H_2), 67.3 (*C*H_2), 58.3 (S*C*H_2), 47.1 (*C*H_2), 42.9 (*C*H_2), 38.9 (*C*H_2C(O)), 38.5 (S*C*H_3).

HRMS: calcd. for $C_{14}H_{20}NO_3S$ $[M+H]^+$: 282.1158; found (ESI^+): 282.1151.

ν_{max} (neat/cm^{-1}): 687, 1035, 1232, 1431, 1492, 1636, 2856, 2917.

m.p.: 120-123 °C.

2-(2-((Methylthio)methyl)-6-(tetrahydrothiophen-2-yl)phenyl)-1-morpholinoethan-1-one (7)

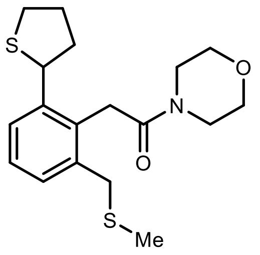

$C_{18}H_{25}NO_2S_2$
MW: 351.53

Synthesised according to **GP-9** from **3a** (0.2 mmol), 2-chloropyridine, (0.4 mmol) tetrahydrothiophene 1-oxide (0.50 mmol) in anhydrous CH_2Cl_2 (0.15 mL), and TFAA (0.2 mmol). Purification by column chromatography (silica gel; 0-50% EtOAc in hexane) afforded the product **7** as a colourless oil (17.9 mg, 0.051 mmol, 51%).

^{1}H NMR (400 MHz, CDCl$_3$, rotamers): δ 7.54 (dd, *J* = 8.0, 1.4 Hz, 1H, Ar *H*), 7.20 (t, *J* = 7.7 Hz, 1H, Ar *H*), 7.06 (dd, *J* = 7.5, 1.4 Hz, 1H, Ar *H*), 4.56 (dd, *J* = 8.8, 5.6 Hz, 1H, SC*H*), 4.12 – 4.02 (m, 1H, SC*H$_2$*), 3.99 – 3.88 (m, 1H, SC*H$_2$*), 3.75 – 3.63 (m, 12H, C*H$_2$* + C(O)CH$_2$), 3.21 – 3.10 (m, 1H, C*H$_2$*), 3.06 – 2.96 (m, 1H, C*H$_2$*), 2.45 – 2.26 (m, 2H, C*H$_2$*), 2.00 (s, 3H, SC*H$_3$*).

^{13}C{^{1}H} NMR (101 MHz, CDCl$_3$, rotamers): δ 169.3 (C=O), 142.5 (Ar *C*), 136.2 (Ar *C*), 133.1 (Ar *C*), 130.7 (Ar *C*H), 129.6 (Ar *C*H), 129.5 (Ar *C*H), 127.9 (Ar *C*H), 127.2 (Ar *C*H), 127.1 (Ar *C*H), 126.4 (Ar *C*H), 67.2 (*C*H$_2$), 66.9 (*C*H$_2$), 49.0 (*C*H$_2$), 42.6 (*C*H$_2$), 42.3 (*C*H$_2$), 38.9 (C(O)*C*H$_2$), 37.6 (S*C*H$_2$), 37.4 (S*C*H$_2$), 33.6 (S*C*H), 32.2 (*C*H$_2$), 31.1 (S*C*H$_2$), 29.8 (*C*H$_2$), 15.3 (S*C*H$_3$).

HRMS: calcd. for $C_{18}H_{25}NO_2S_2Na$ [M+H]$^+$: 374.1219; found (ESI$^+$): 374.1213.

ν_{max} (neat/cm^{-1}): 669, 1035, 1228, 1428, 1642, 2854, 2917.

5 References

(1) Shennan, B. D. A.; Sánchez-Alonso, S.; Rossini, G.; Dixon, D. J. 1,2-Redox Transpositions of Tertiary Amides. *J. Am. Chem. Soc.* **2023**, *145*, 21745–21751.

(2) Heyde, C.; Zug, I.; Hartmann, H. A Simple Route to N,N-Dialkyl Derivatives of 2-Amino-5-Thiophenecarboxylates. *Eur. J. Org. Chem.* **2000**, 3273–3278.

(3) Zhang, W.; Ready, J. M. The Ketene-Surrogate Coupling: Catalytic Conversion of Aryl Iodides into Aryl Ketenes through Ynol Ethers. *Angew. Chem. Int. Ed.* **2014**, *53*, 8980–8984.

(4) Oku, N.; Murakami, M.; Miura, T. Photoassisted Cross-Coupling Reaction of α-Chlorocarbonyl Compounds with Arylboronic Acids. *Org. Lett.* **2022**, *24*, 1616–1619.

(5) Djukanovic, D.; Ganiek, M. A.; Nishi, K.; Karaghiosoff, K.; Mashima, K.; Knochel, P. Preparation of Functionalized Amides Using Dicarbamoylzincs. *Angew. Chem. Int. Ed.* **2022**, *61*, e202205440.

(6) Wu, Z.; Laffoon, J. D.; Nguyen, T. T.; McAlpin, J. D.; Hull, K. L. Rhodium-Catalyzed Asymmetric Synthesis of β-Branched Amides. *Angew. Chem. Int. Ed.* **2017**, *129*, 1391–1395.

(7) Rodrigalvarez, J.; Wang, H.; Martin, R. Native Amides as Enabling Vehicles for Forging Sp3–Sp3 Architectures via Interrupted Deaminative Ni-Catalyzed Chain-Walking. *J. Am. Chem. Soc.* **2023**, *145*, 3869–3874.

(8) Bigi, M. A.; White, M. C. Terminal Olefins to Linear α,β-Unsaturated Ketones: Pd(II)/Hypervalent Iodine Co-Catalyzed Wacker Oxidation–Dehydrogenation. *J. Am. Chem. Soc.* **2013**, *135*, 7831–7834.

(9) Triandafillidi, Ierasia; Athanasiou, Modestos; Itskos, Grigorios; Koutentis, Panayiotis A.; Kokotos, Christoforos G. 3H-Phenothiazin-3-one: A Photocatalyst for the Aerobic Photochemical Oxidation of Sulfides to Sulfoxides. *Adv. Synth. Catal.* **2023**, *365*, 2643-2650.

(10) Amri, Nasser; Wirth, Thomas. Flow Electrosynthesis of Sulfoxides, Sulfones, and Sulfoximines without Supporting Electrolytes. *J. Org. Chem.* **2021***, 86,* 15961–15972.

(11) Maitra, Pranay Kumar; Bhattacharyya, Soumalya; Hickey, Neal; Mukherjee, Partha Sarathi. Self-Assembly of a Water-Soluble Pd16 Square Bicupola Architecture and Its Use in Aerobic Oxidation in Aqueous Medium. *J. Am. Chem. Soc.* **2024***, 146,* 15301–15308.

(12) Leypold, M.; D'Angelo, K. A.; Movassaghi, M. Chemoselective α-Sulfidation of Amides Using Sulfoxide Reagents. *Org. Lett.* **2020**, *22*, 8802–8807.

6 NMR Spectra

1a – ^{1}H NMR (400 MHz, $CDCl_3$)

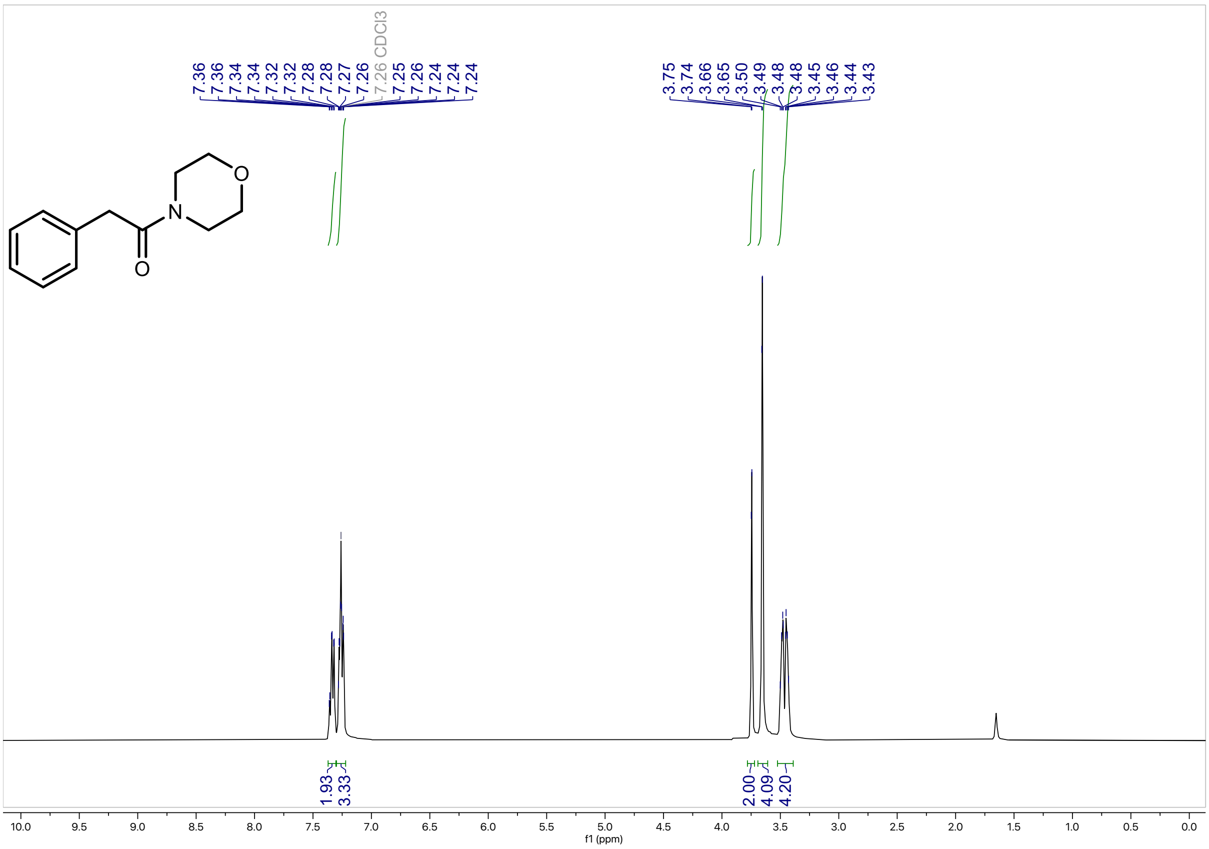

1a – ^{13}C NMR (101 MHz, $CDCl_3$)

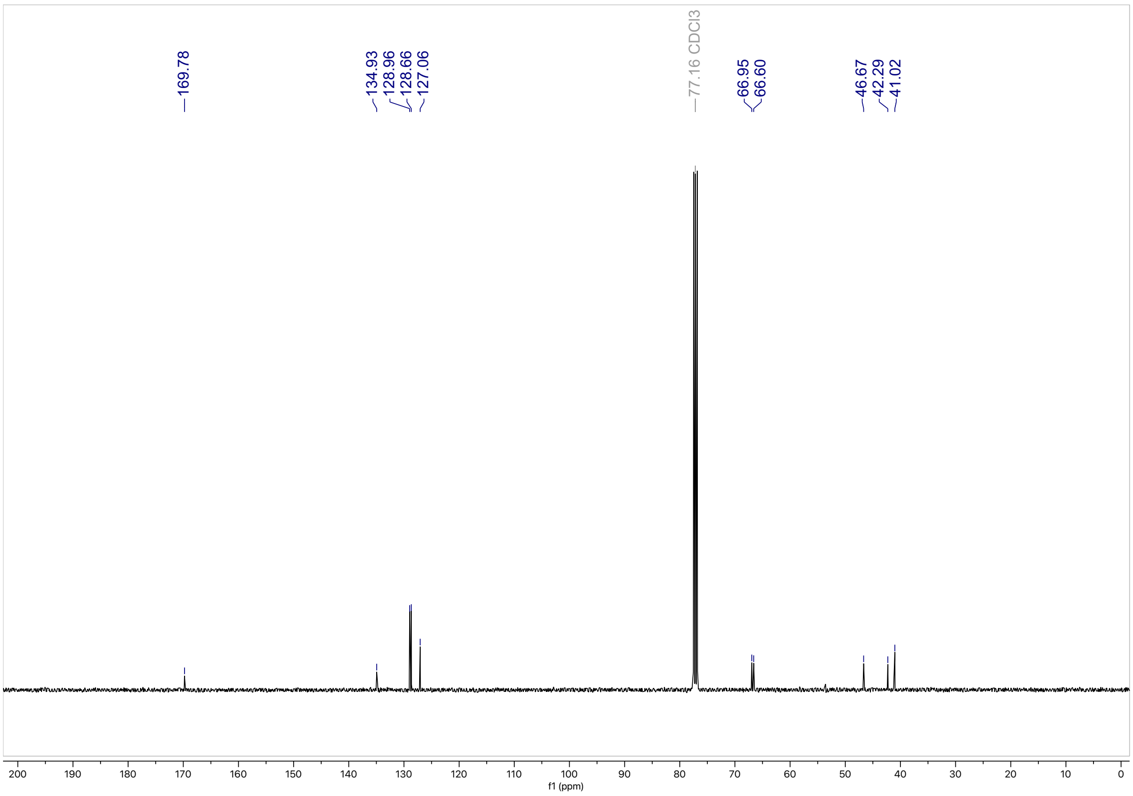

1b – ^{1}H NMR (400 MHz, $CDCl_3$)

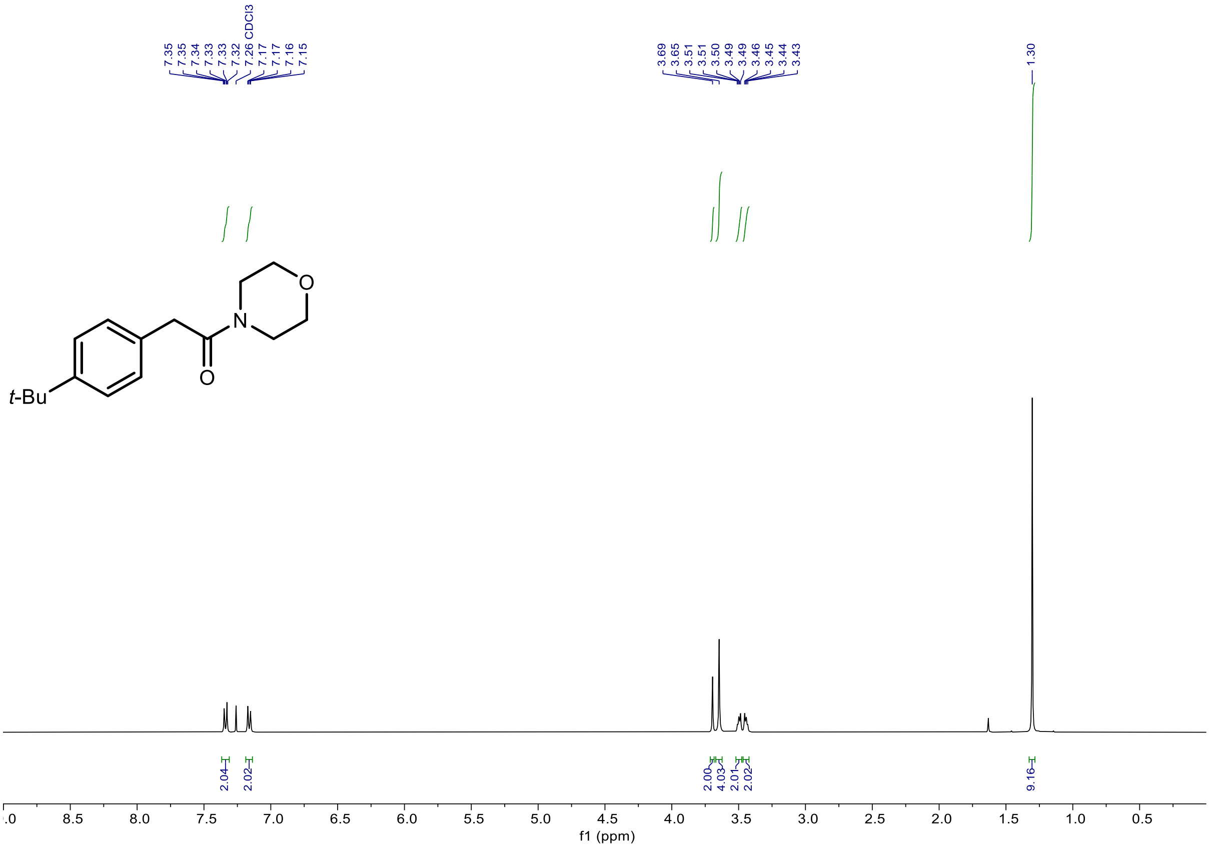

1b – ^{13}C NMR (101 MHz, $CDCl_3$)

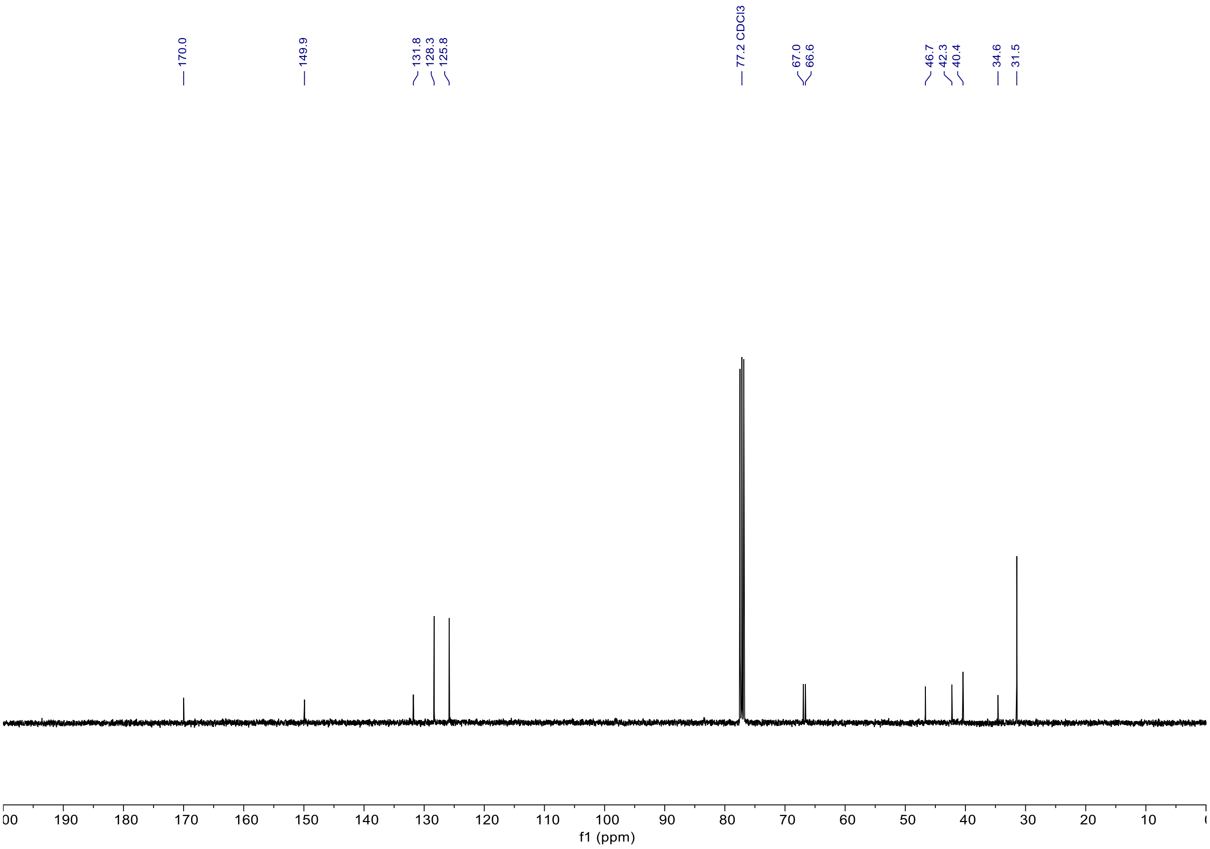

1c – ^{1}H NMR (400 MHz, $CDCl_3$)

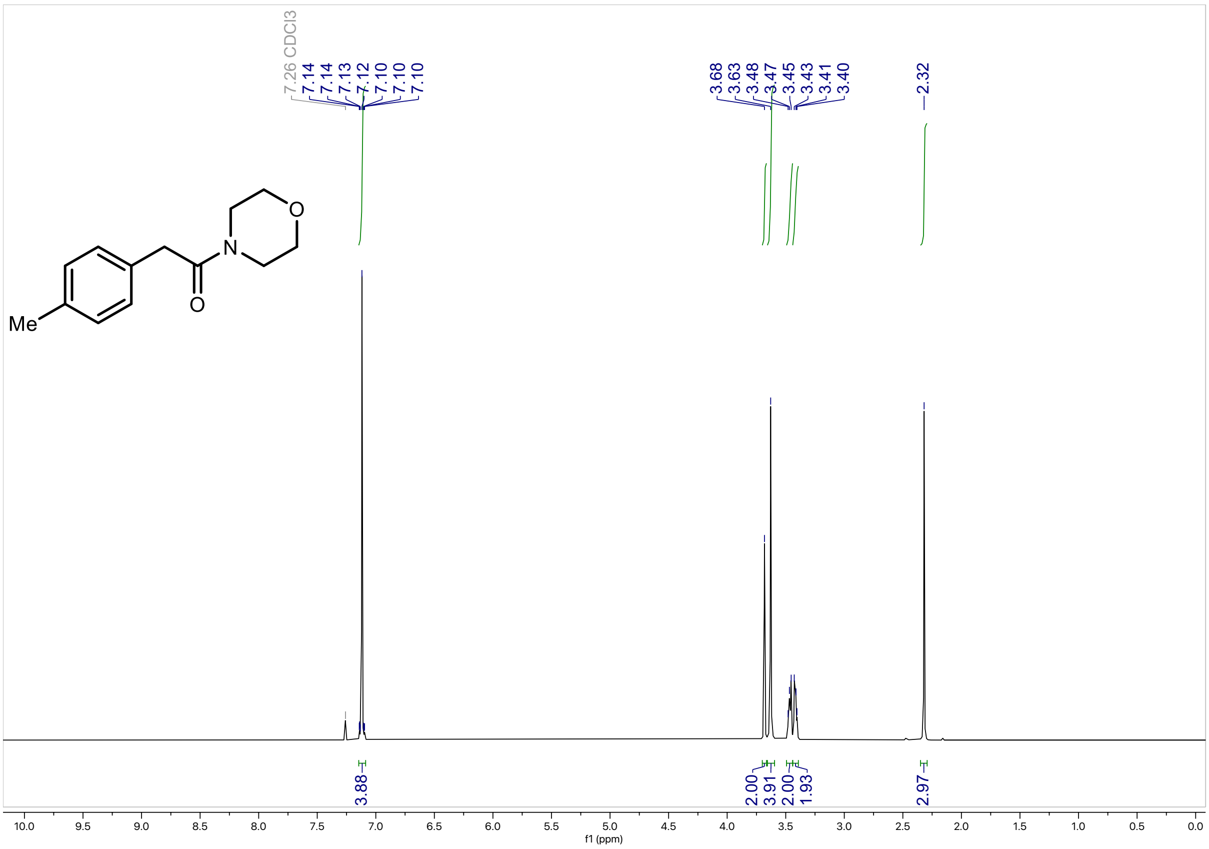

1c – ^{13}C NMR (101 MHz, $CDCl_3$)

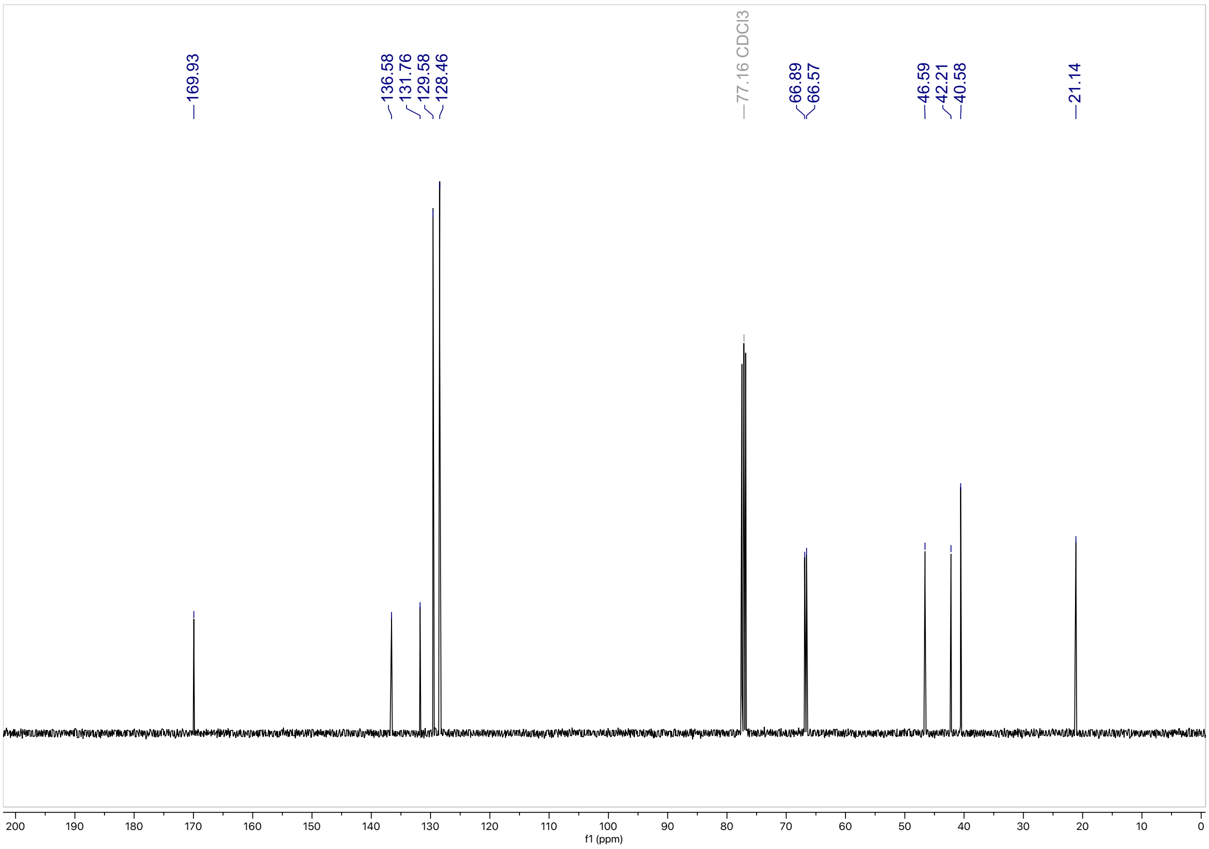

1d – ^{1}H NMR (500 MHz, $CDCl_3$)

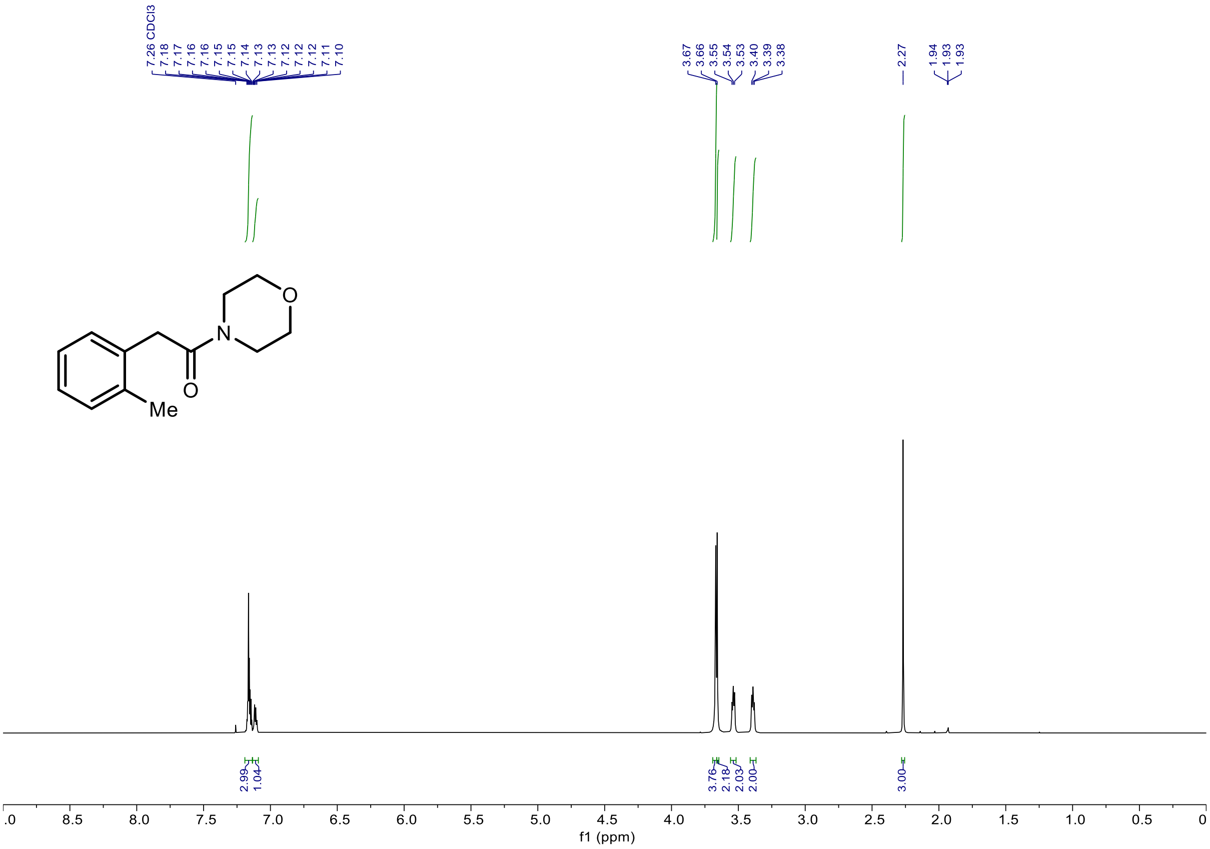

1d – ^{13}C NMR (126 MHz, $CDCl_3$)

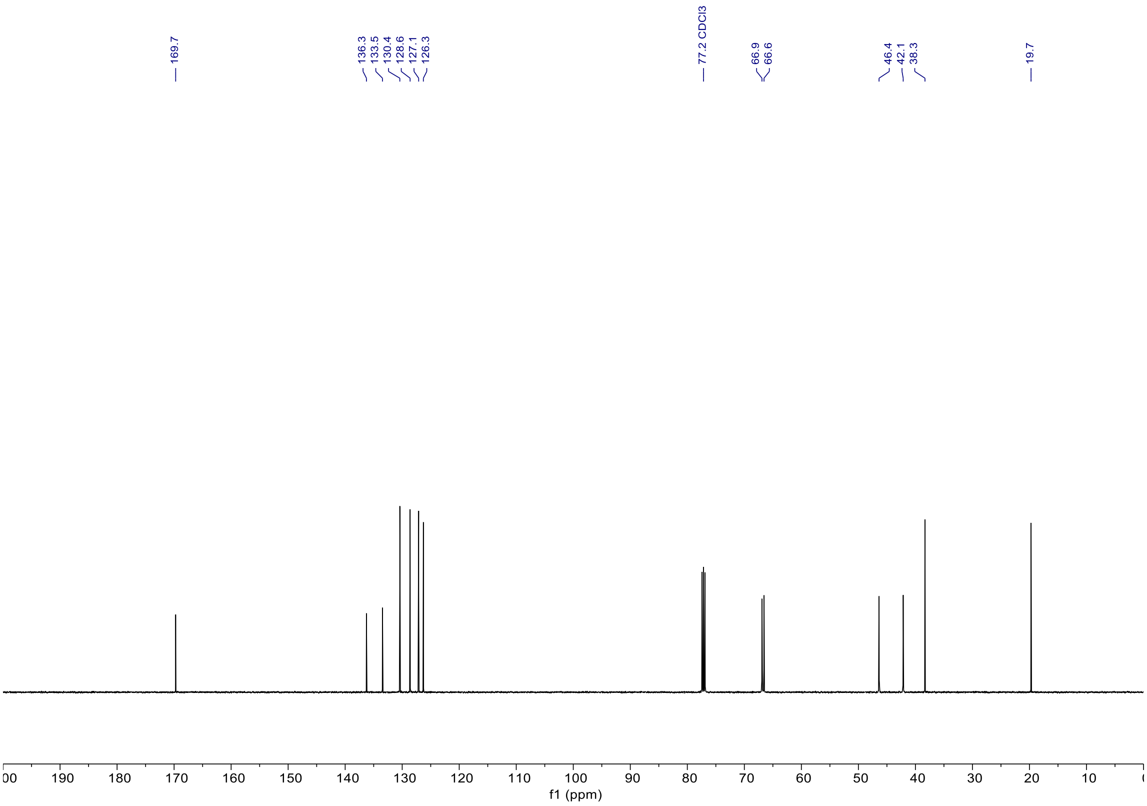

1e – ^{1}H NMR (400 MHz, CDCl$_3$)

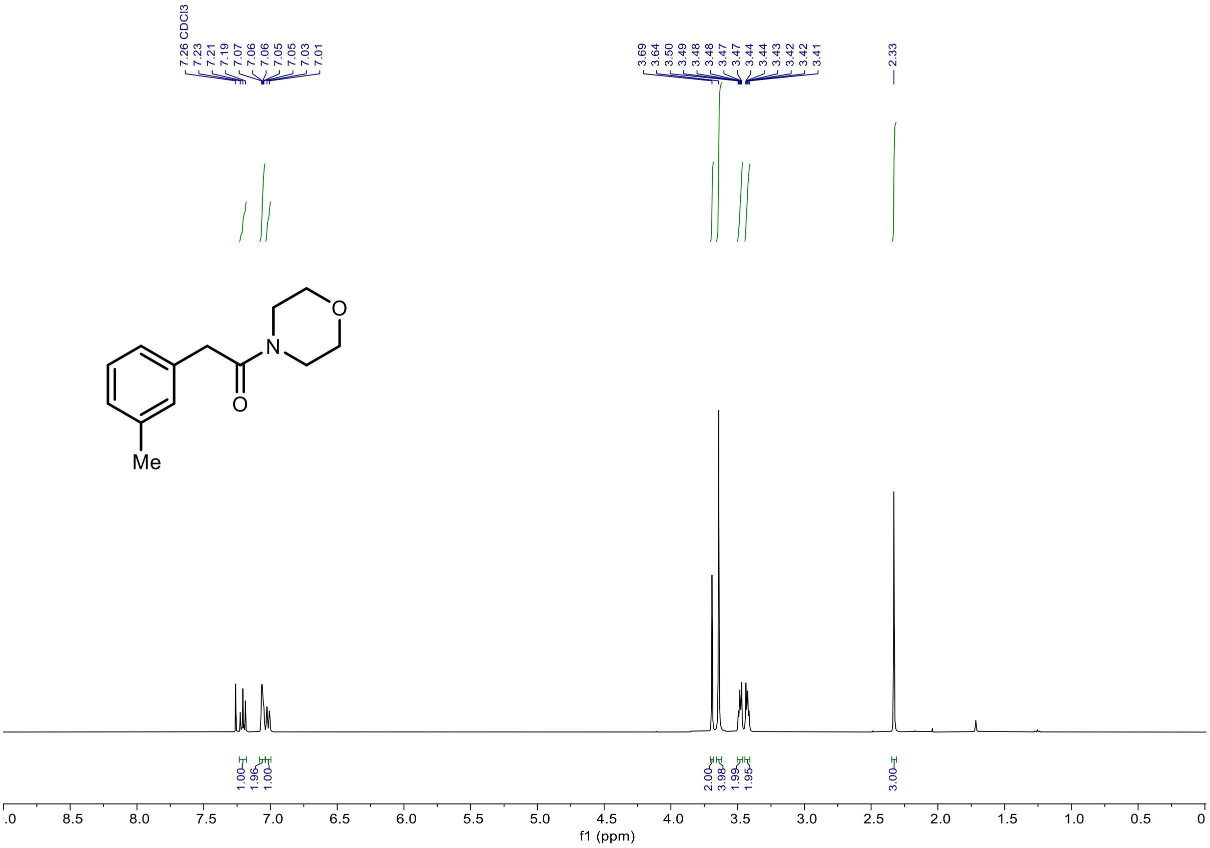

1e – ^{13}C NMR (101 MHz, CDCl$_3$)

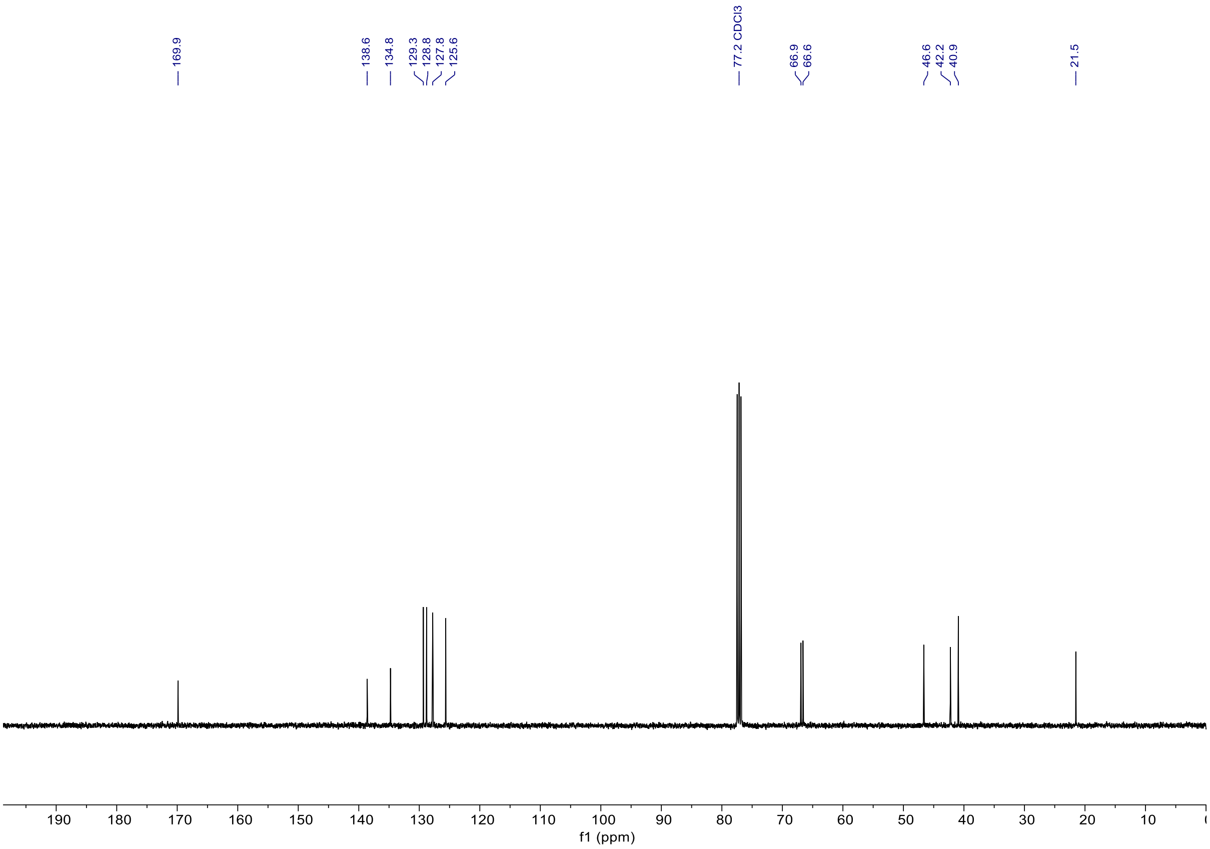

1f – ¹H NMR (400 MHz, $CDCl_3$)

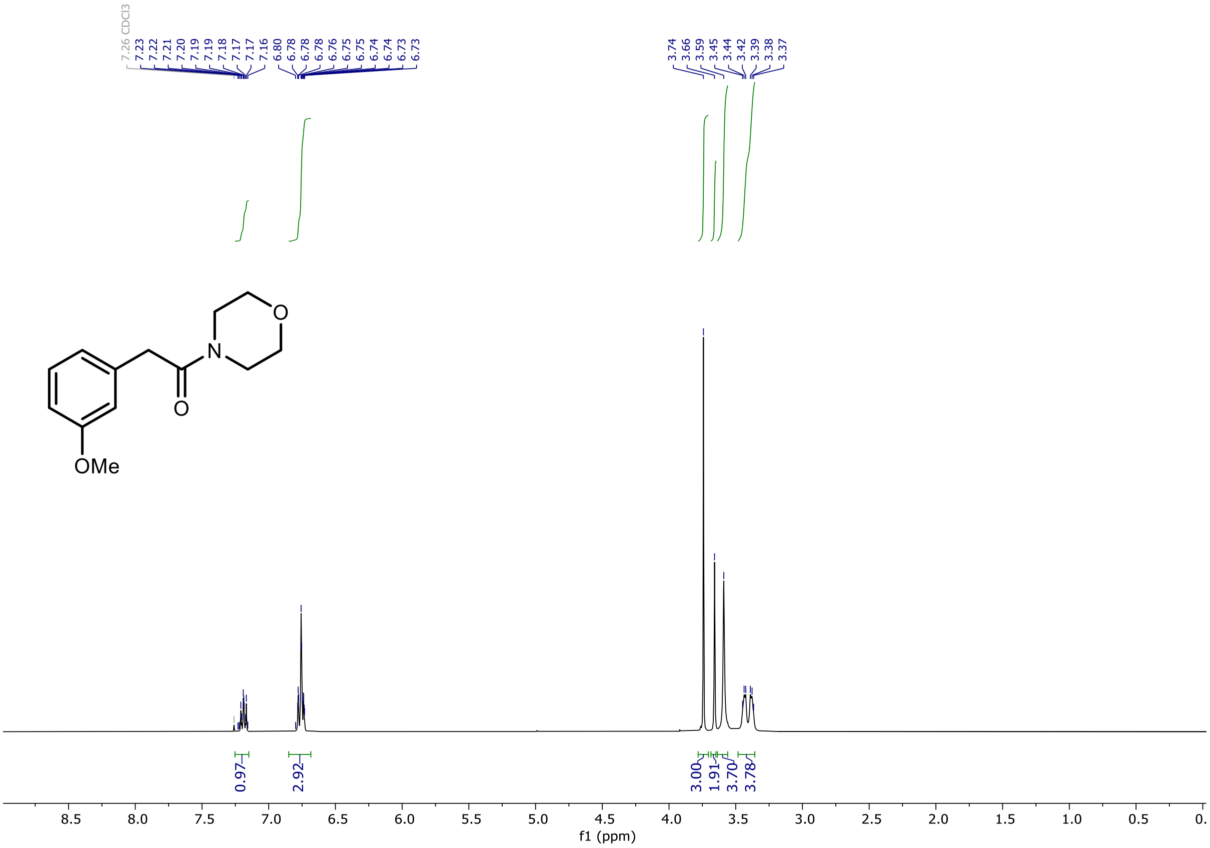

1f – ¹³C NMR (101 MHz, $CDCl_3$)

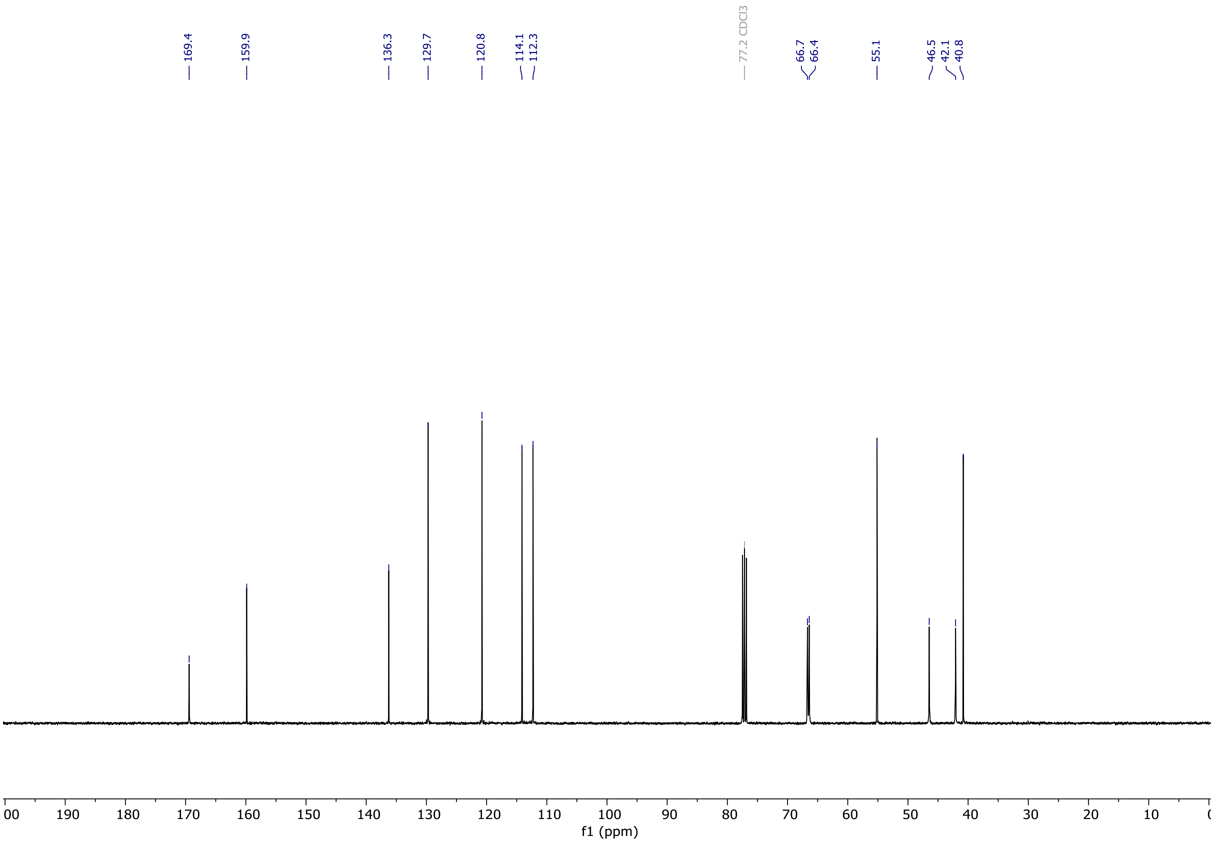

1g – ^{1}H NMR (500 MHz, $CDCl_3$)

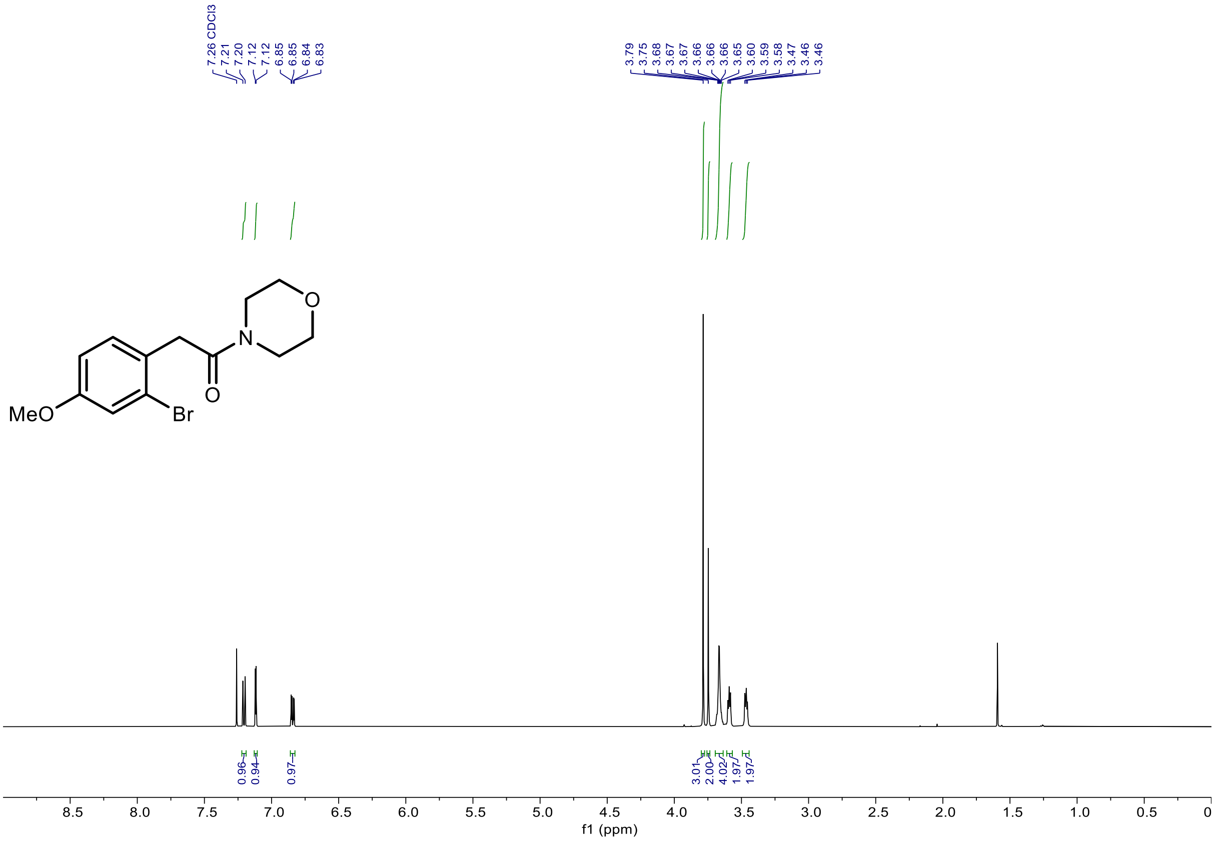

1g – ^{13}C NMR (126 MHz, $CDCl_3$)

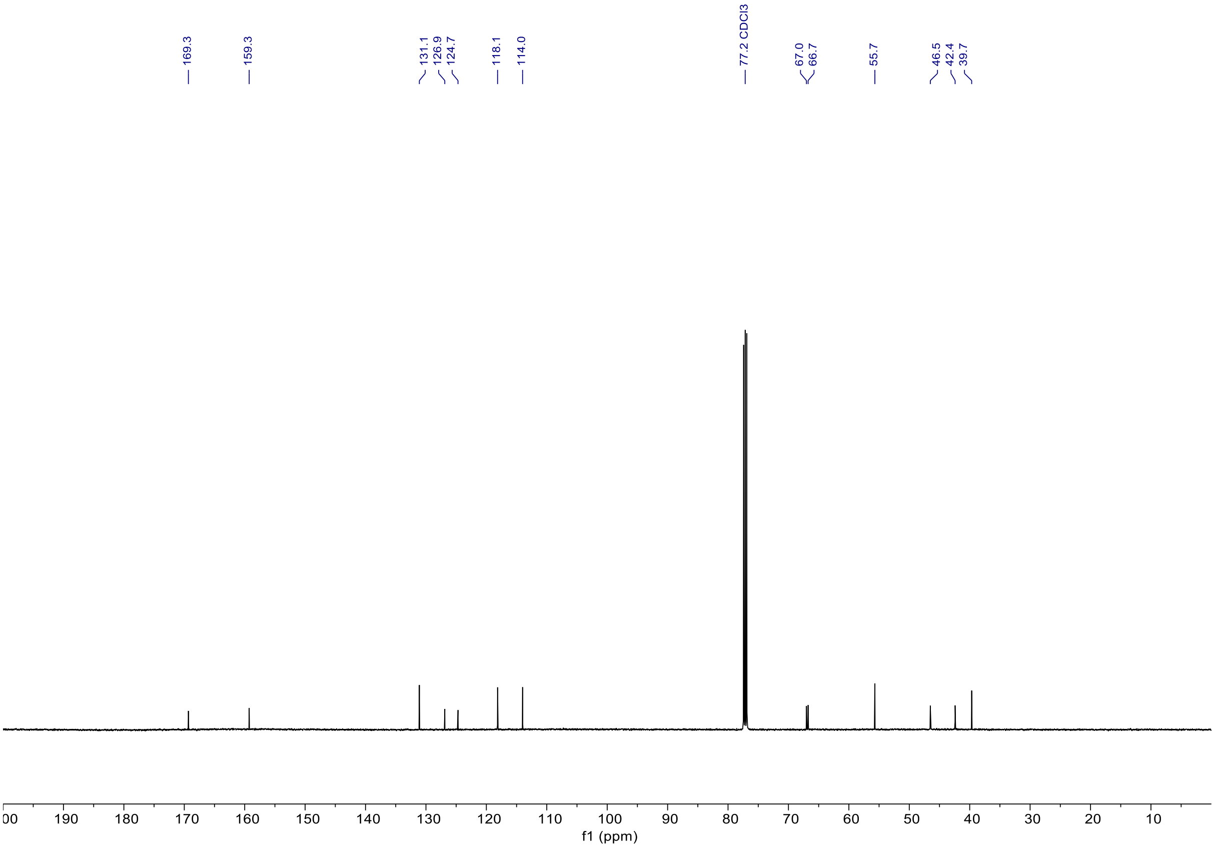

1h – ^{1}H NMR (500 MHz, $CDCl_3$)

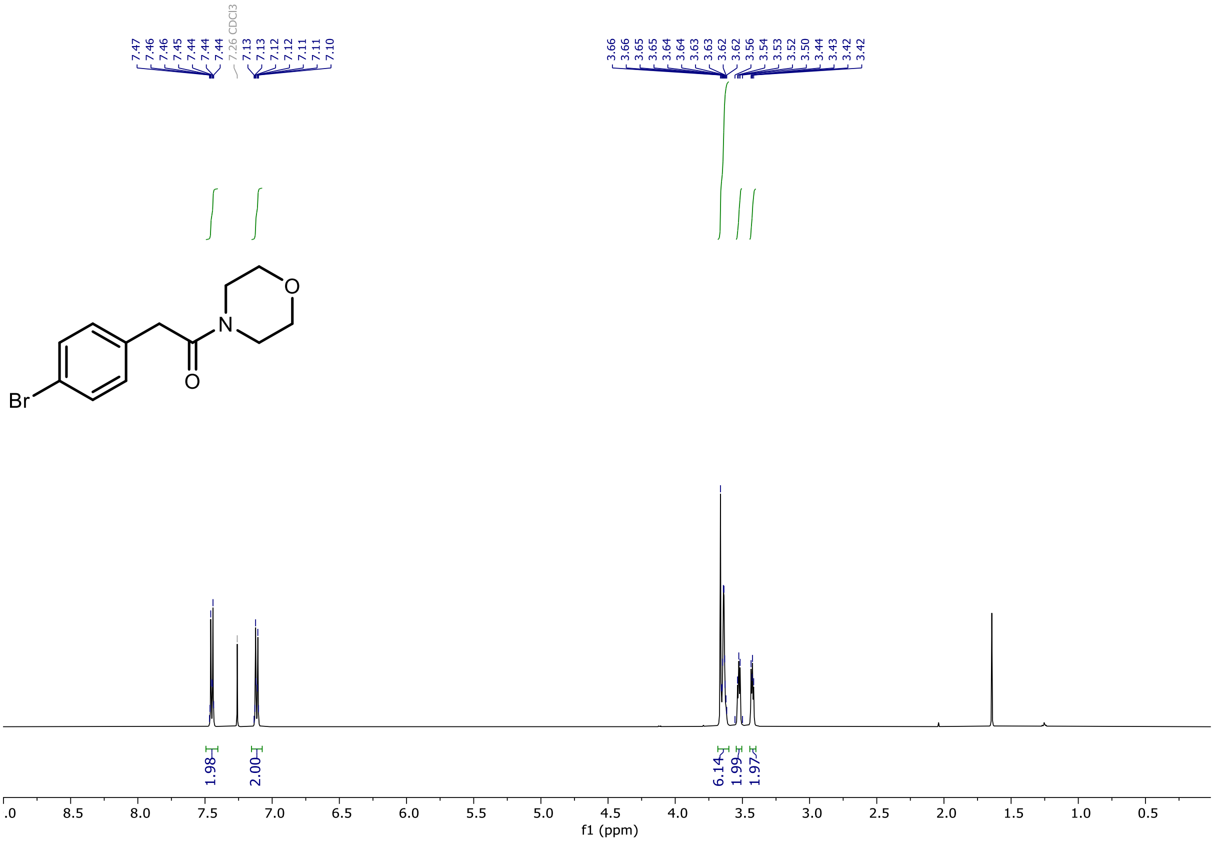

1h – ^{13}C NMR (126 MHz, $CDCl_3$)

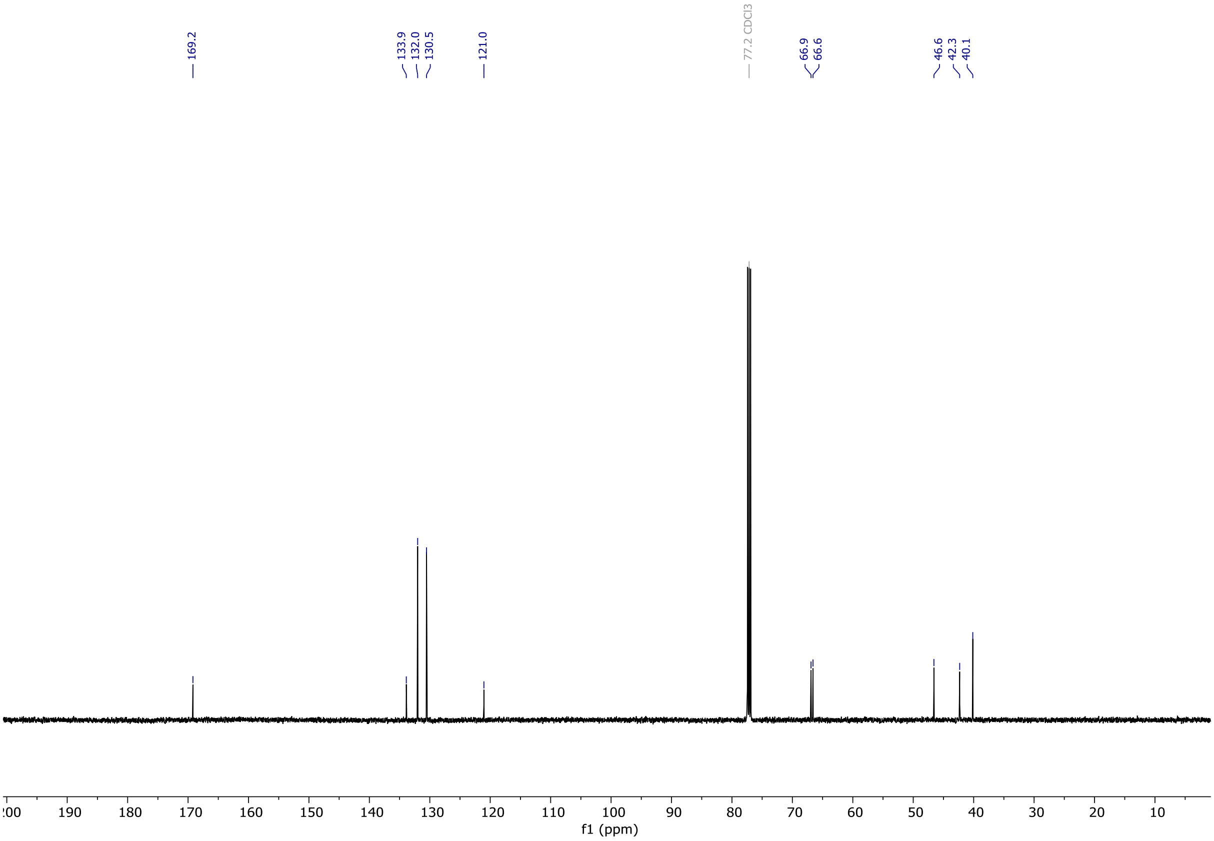

1i – ^{1}H NMR (400 MHz, $CDCl_3$)

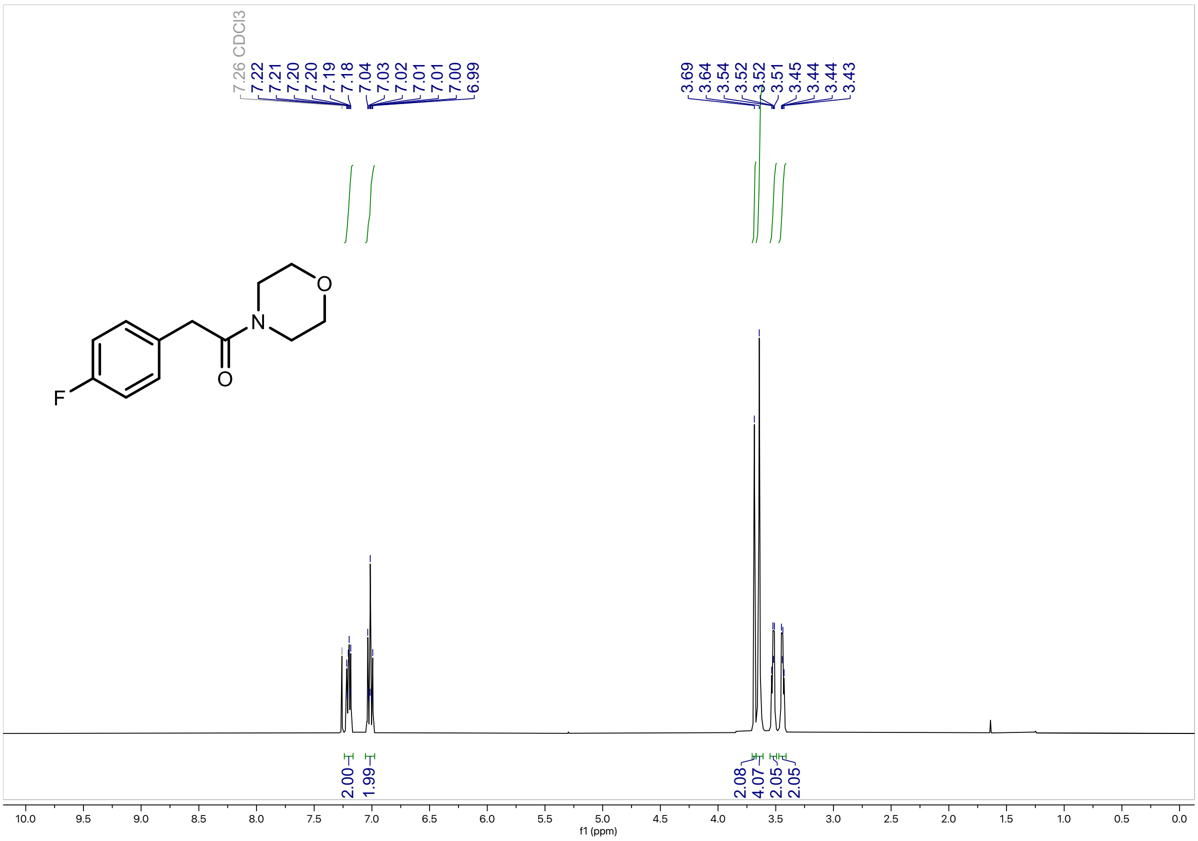

1i – ^{13}C NMR (101 MHz, $CDCl_3$)

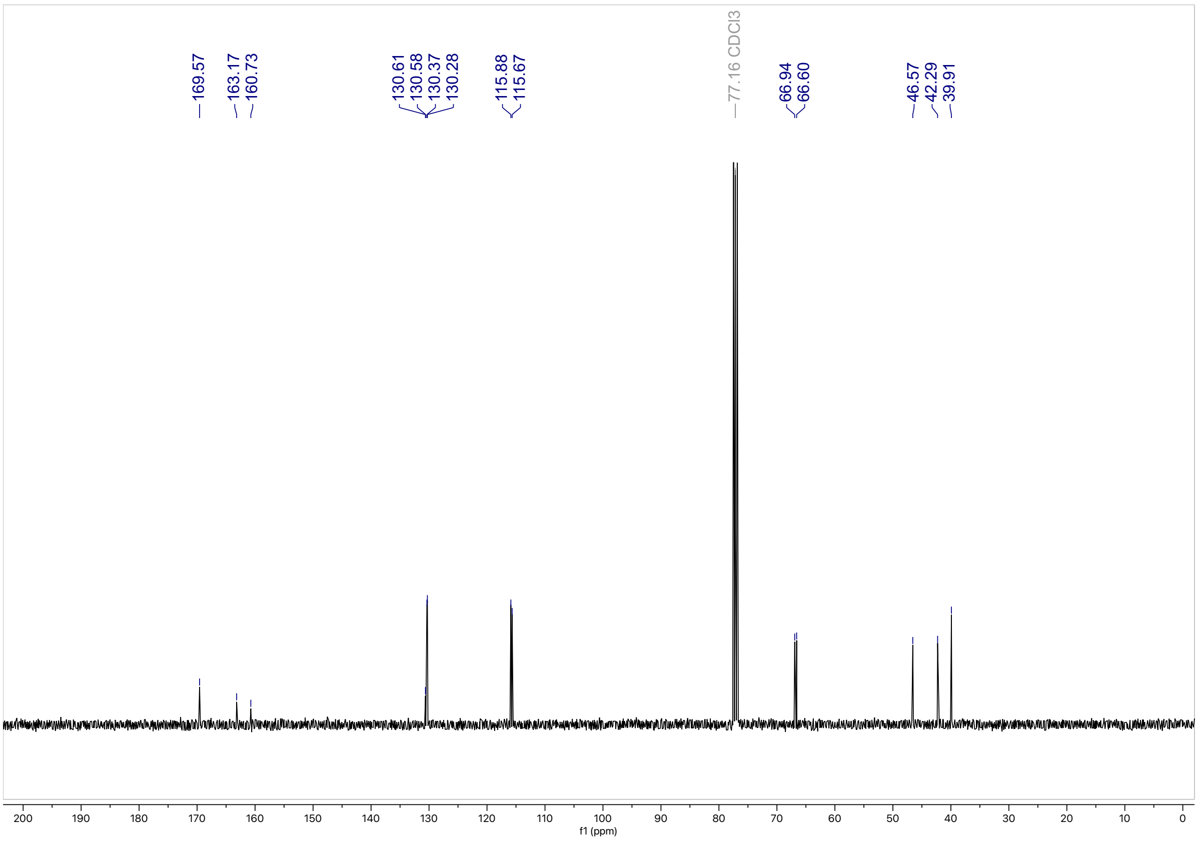

1i – ^{19}F NMR (376 MHz, $CDCl_3$)

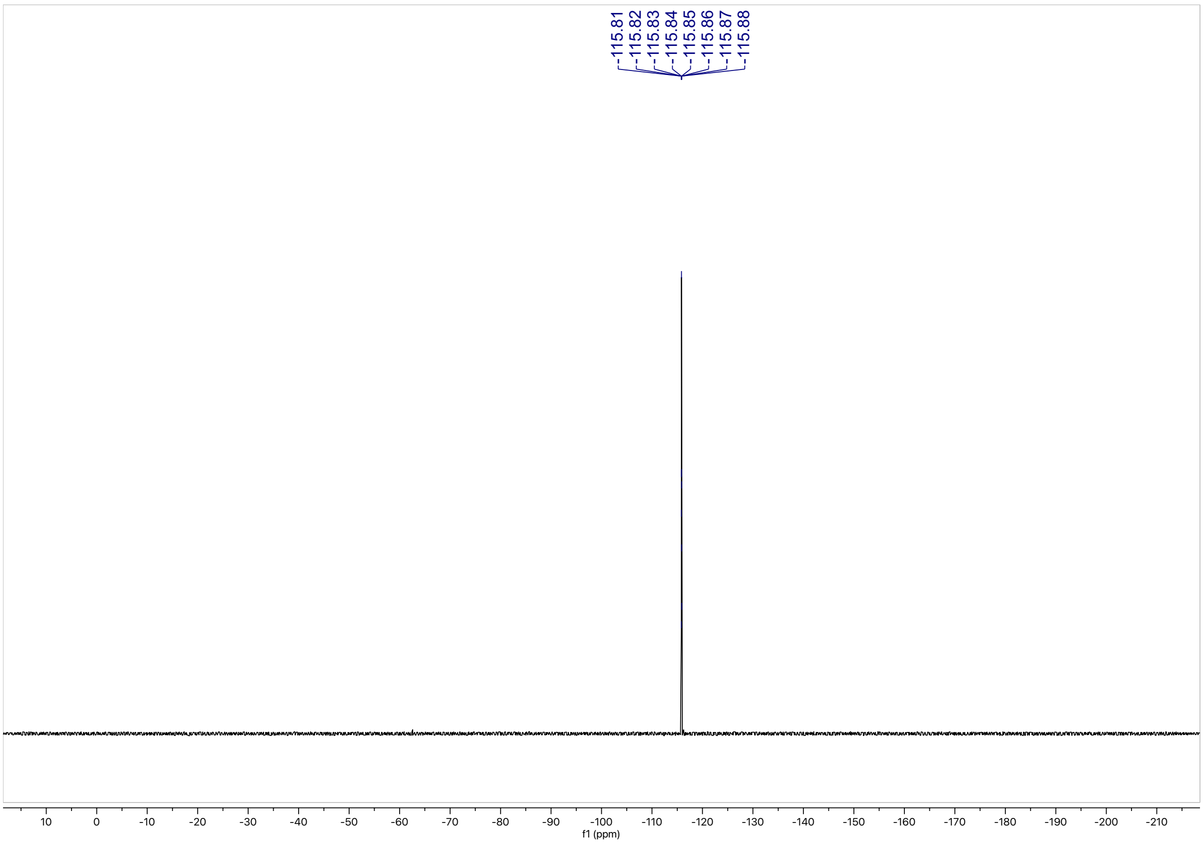

1j – ^{1}H NMR (400 MHz, $CDCl_3$)

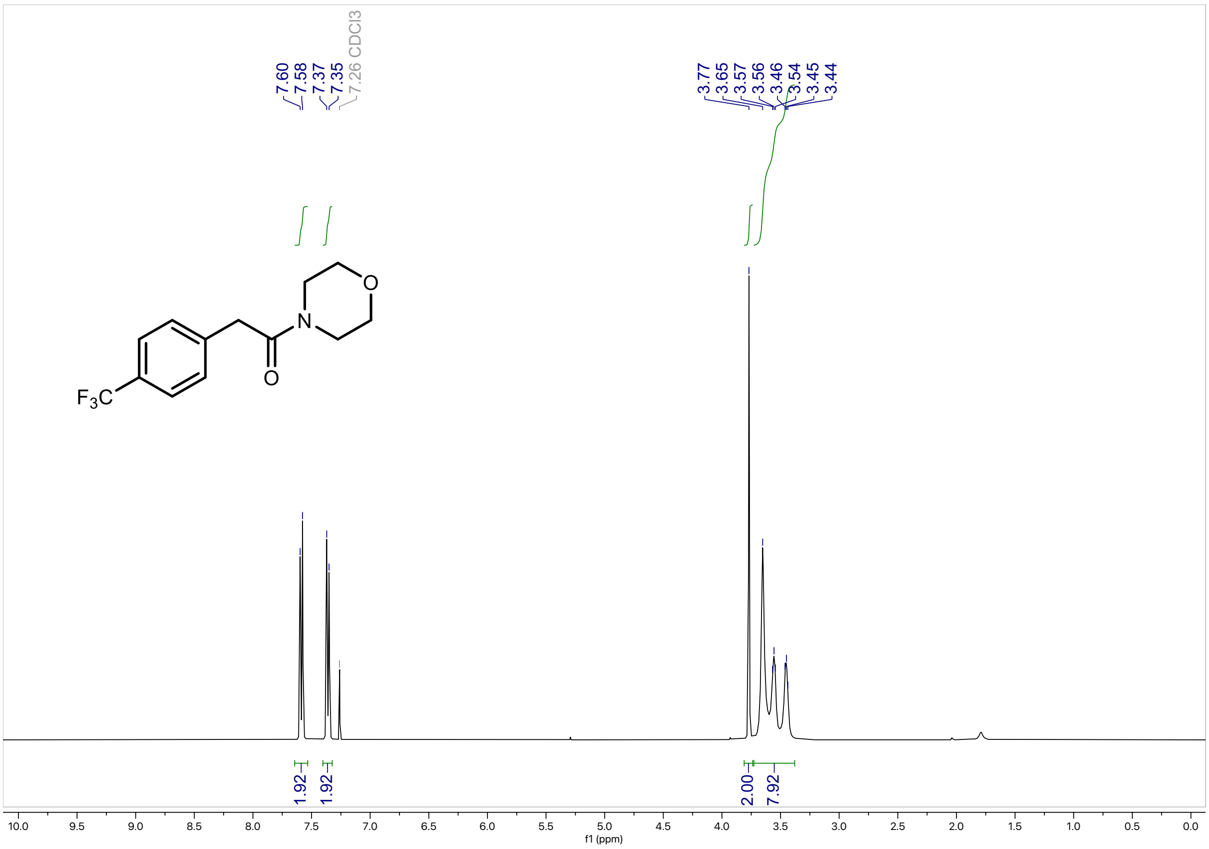

1j – ^{13}C NMR (101 MHz, $CDCl_3$)

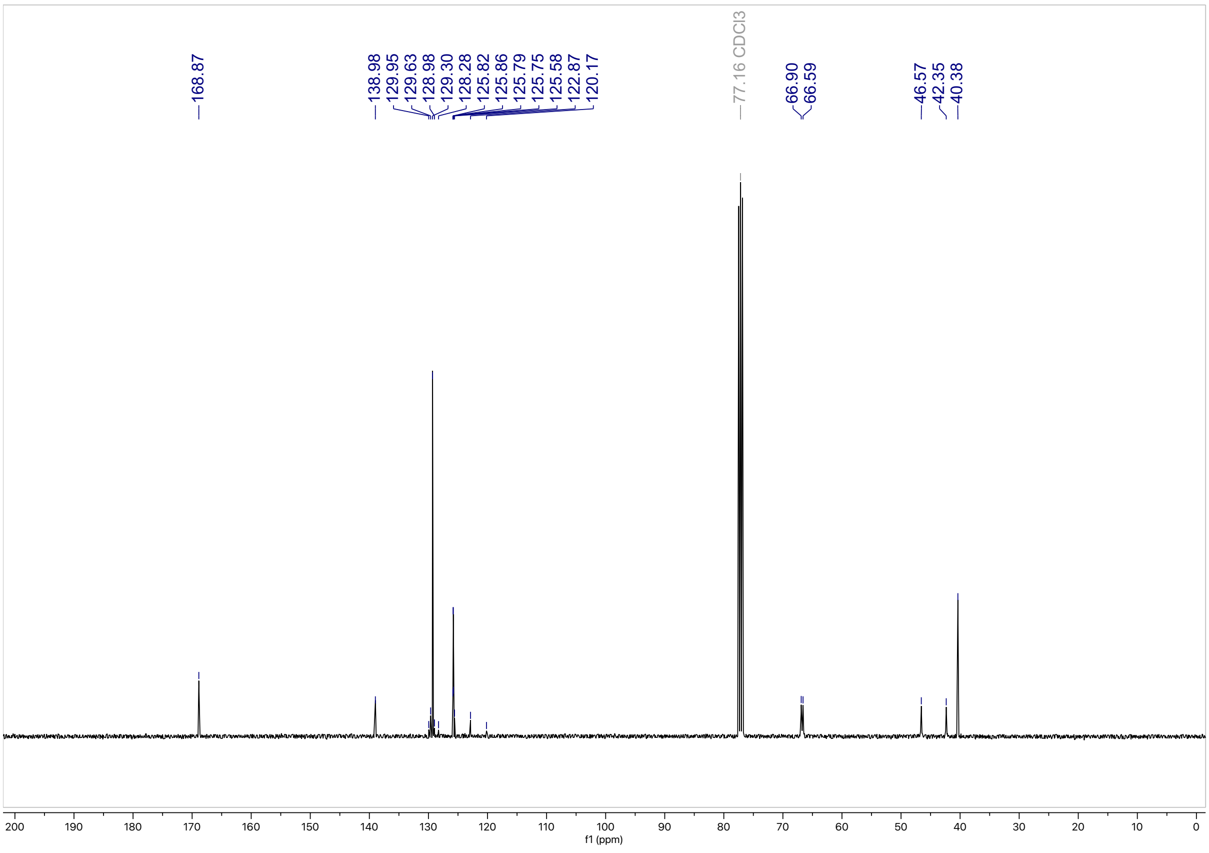

1j – ^{19}F NMR (376 MHz, $CDCl_3$)

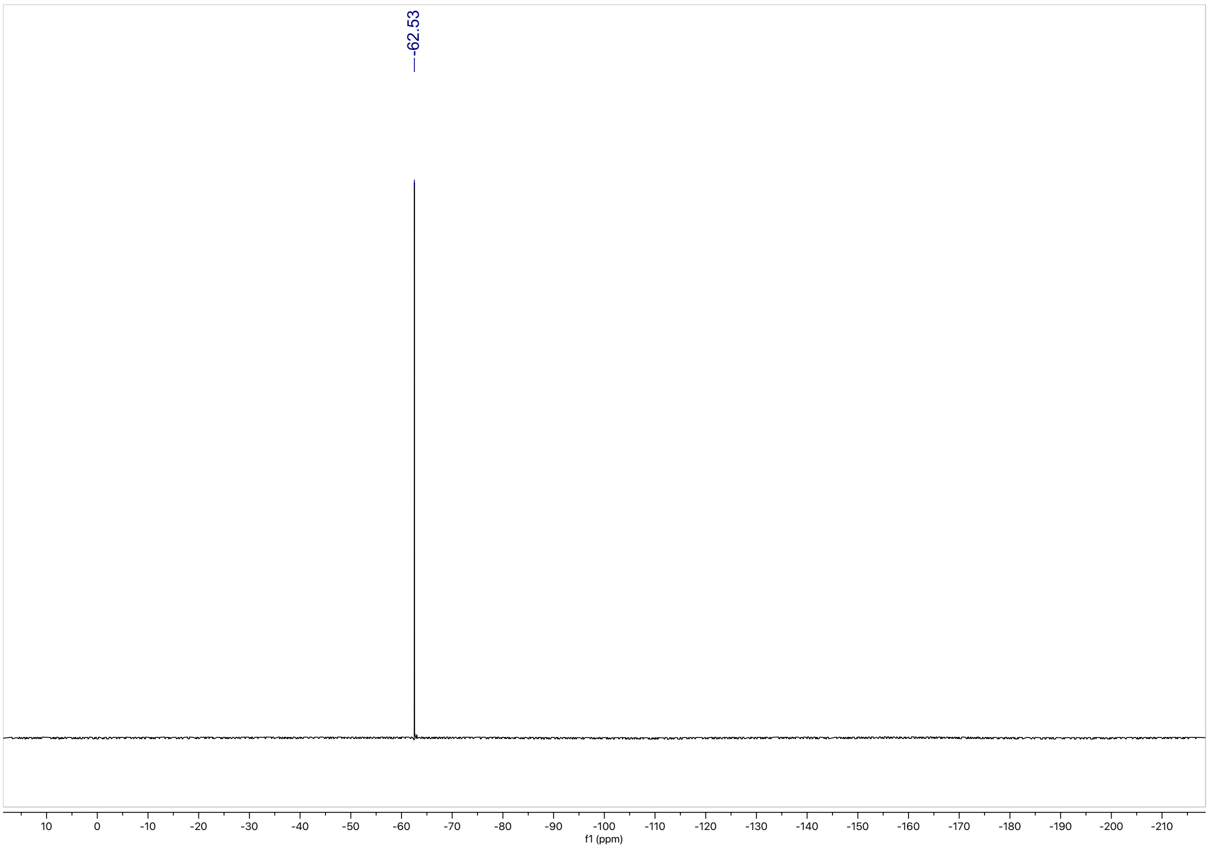

1k – ^{1}H NMR (400 MHz, $CDCl_3$)

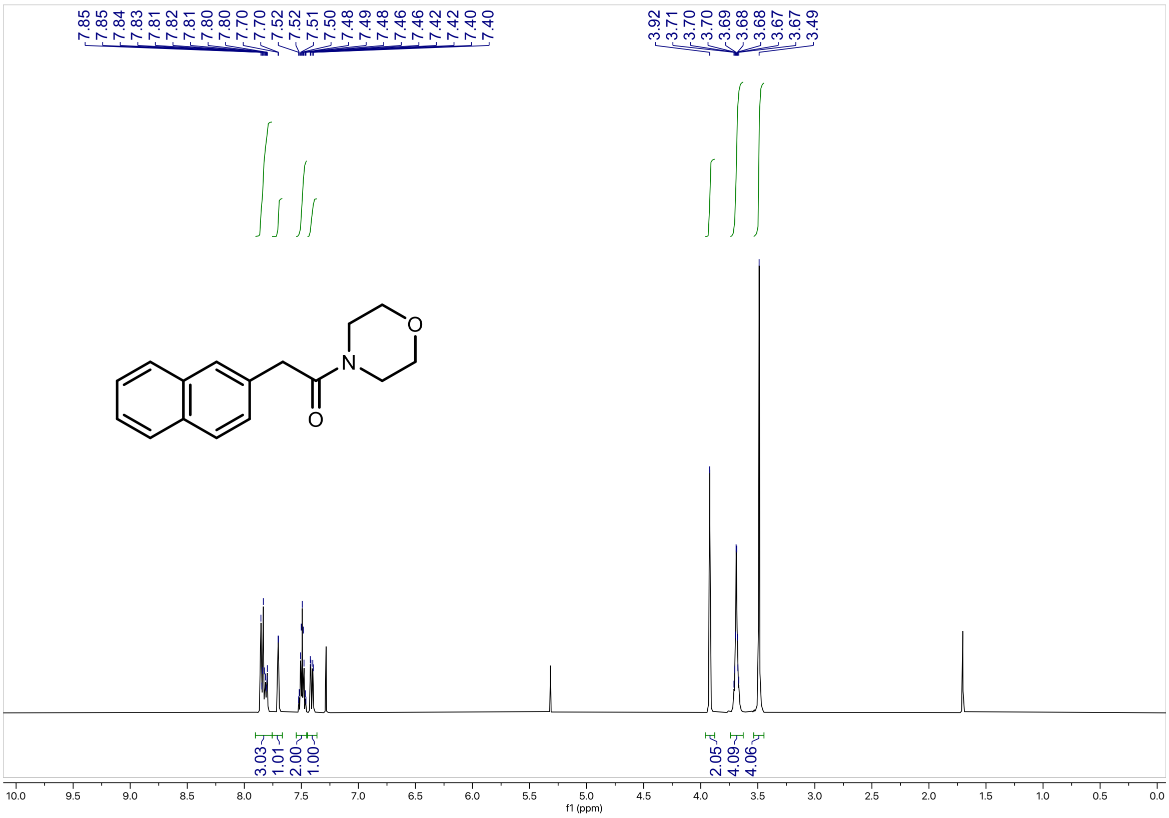

1k – ^{13}C NMR (101 MHz, $CDCl_3$)

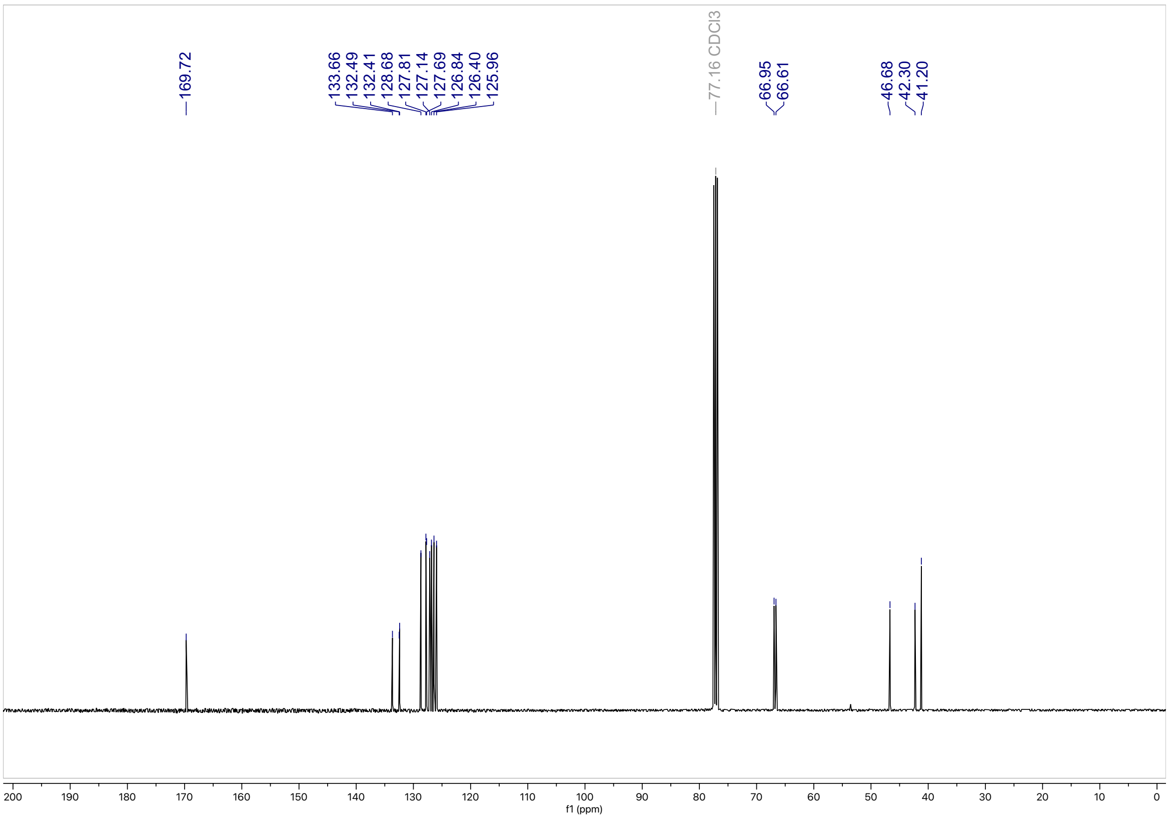

1l – ^{1}H NMR (400 MHz, $CDCl_3$)

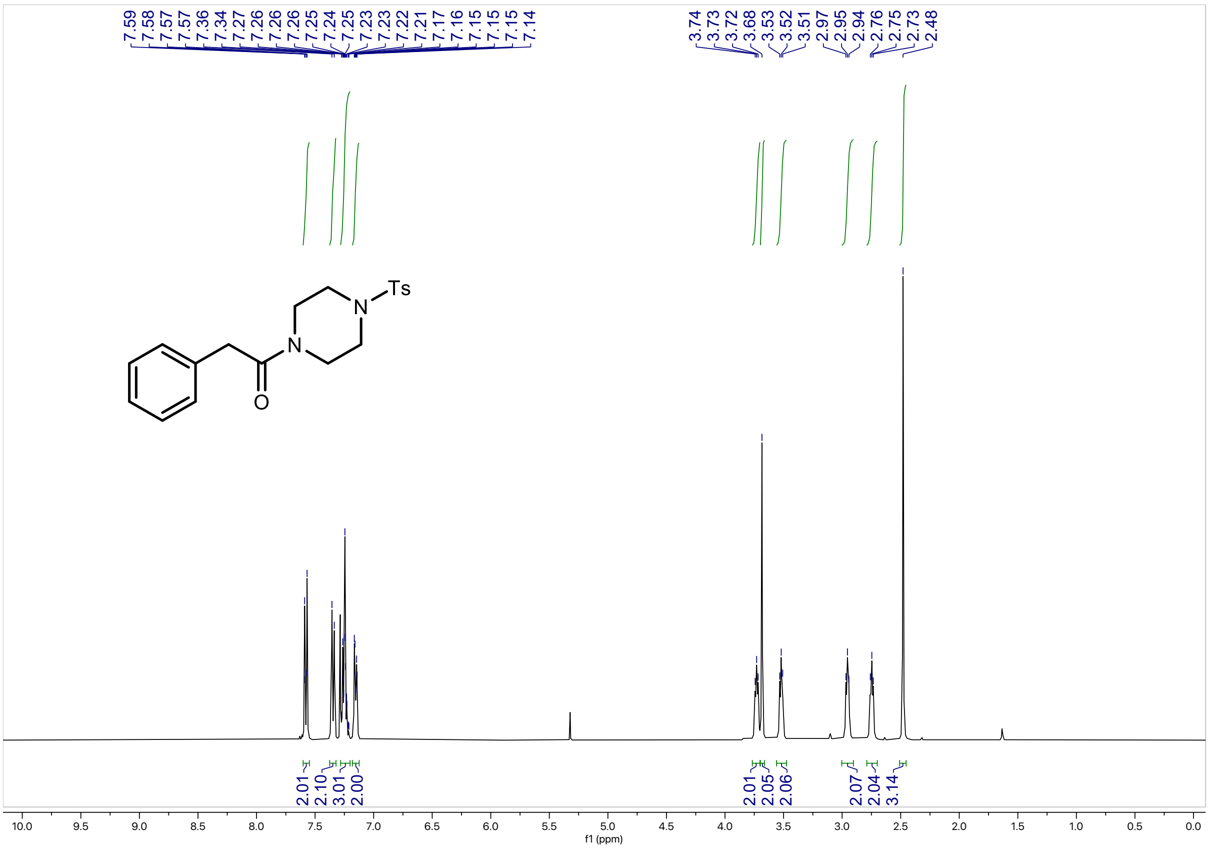

1l – ^{13}C NMR (101 MHz, $CDCl_3$)

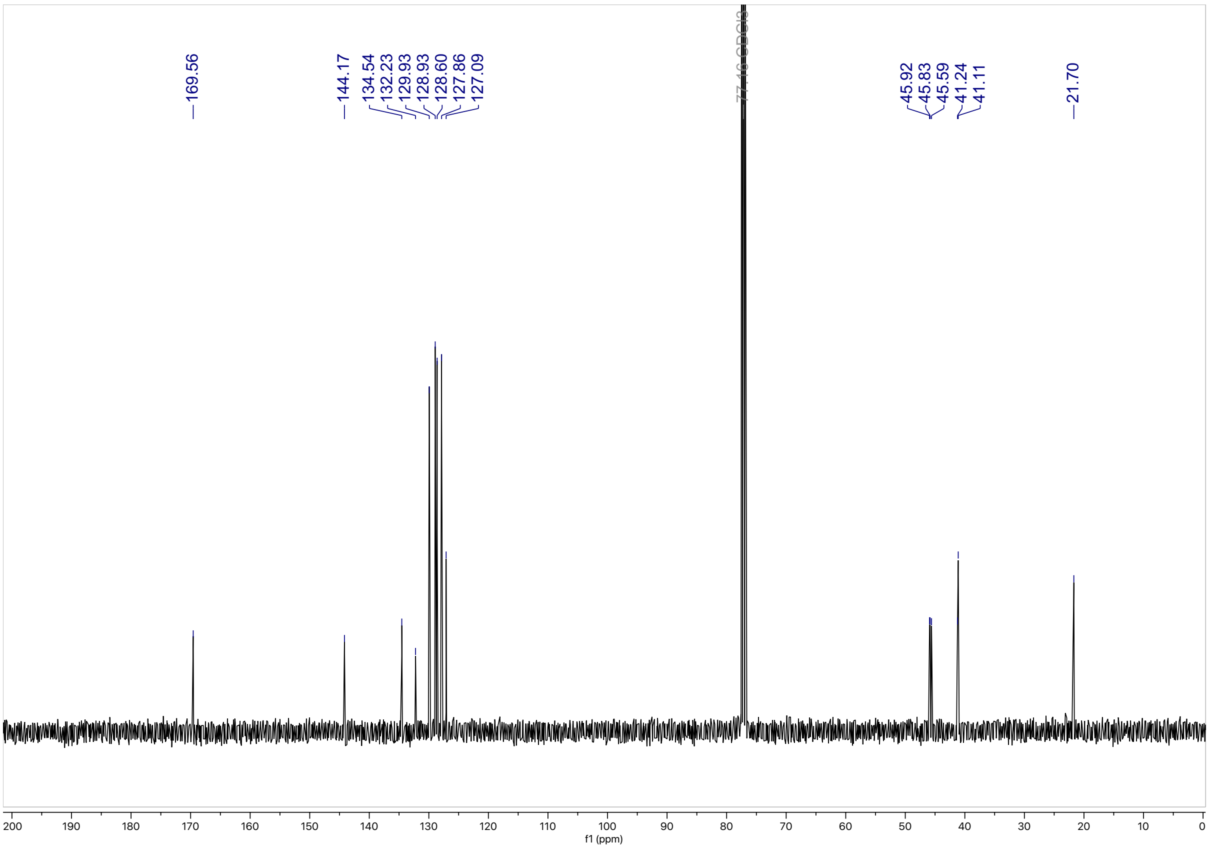

1m – ^{1}H NMR (400 MHz, CDCl$_3$)

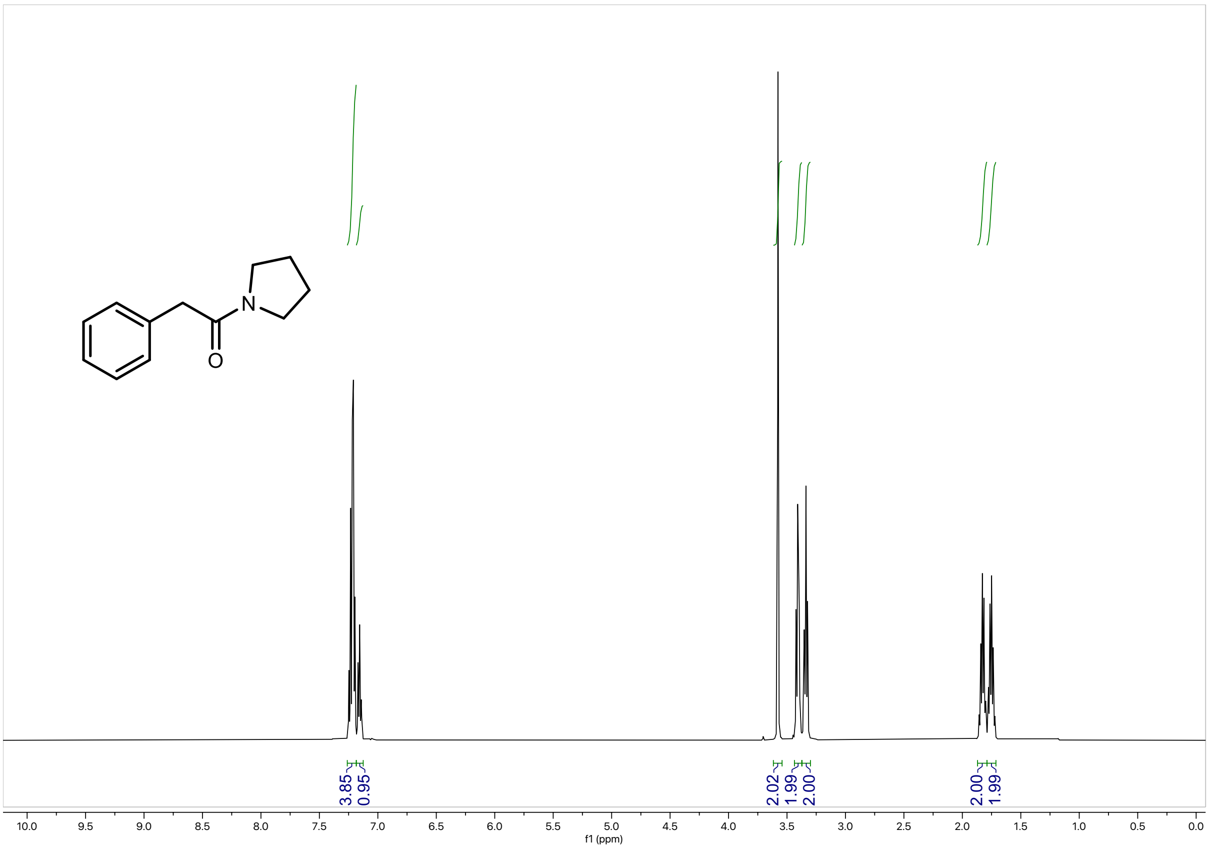

1m – ^{13}C NMR (101 MHz, CDCl$_3$)

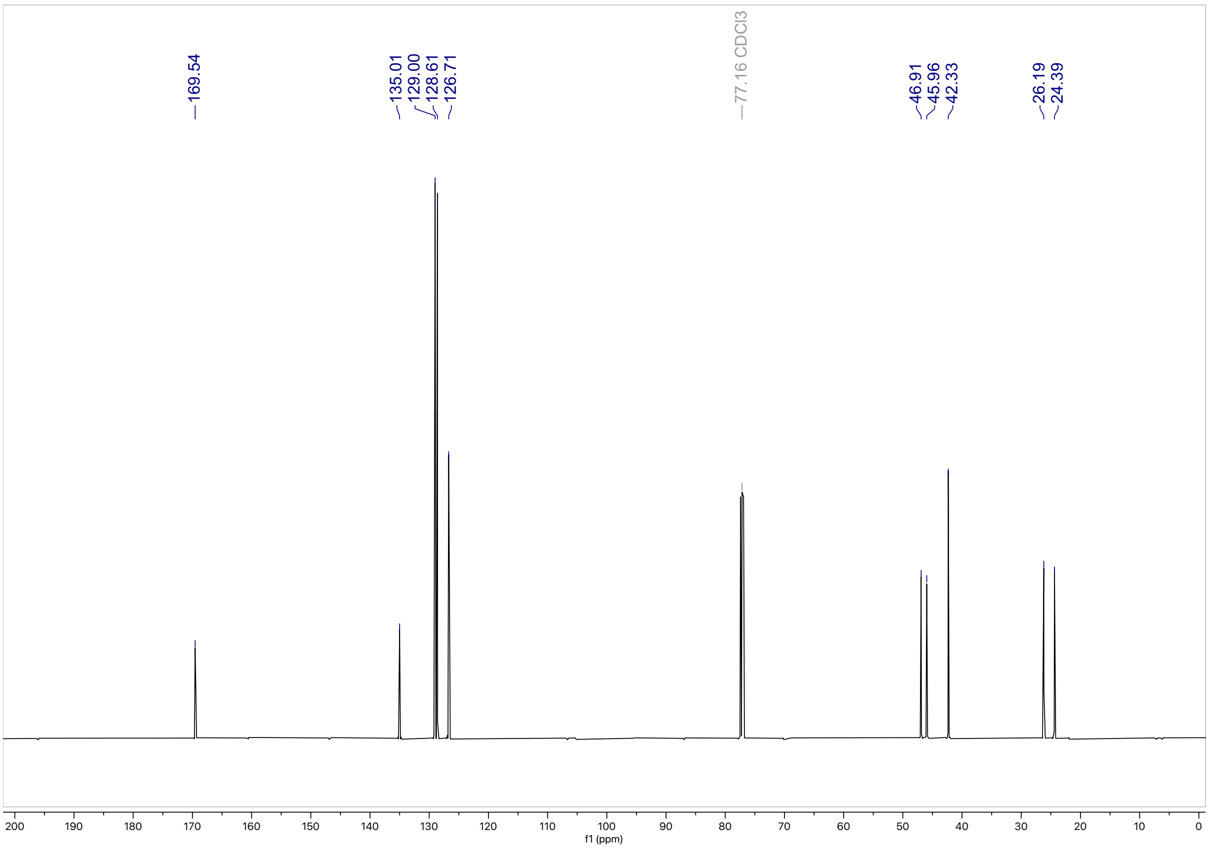

1n – ^{1}H NMR (400 MHz, $CDCl_3$)

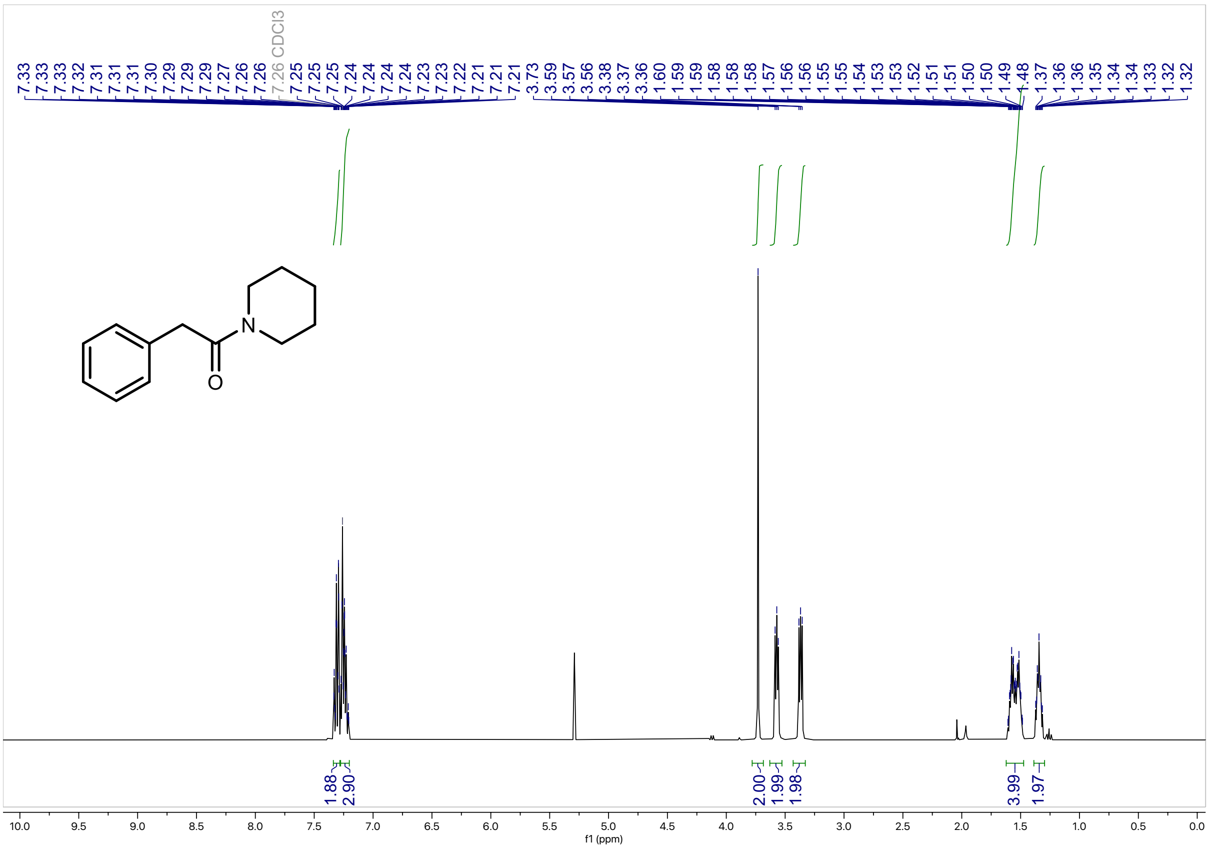

1n – ^{13}C NMR (101 MHz, $CDCl_3$)

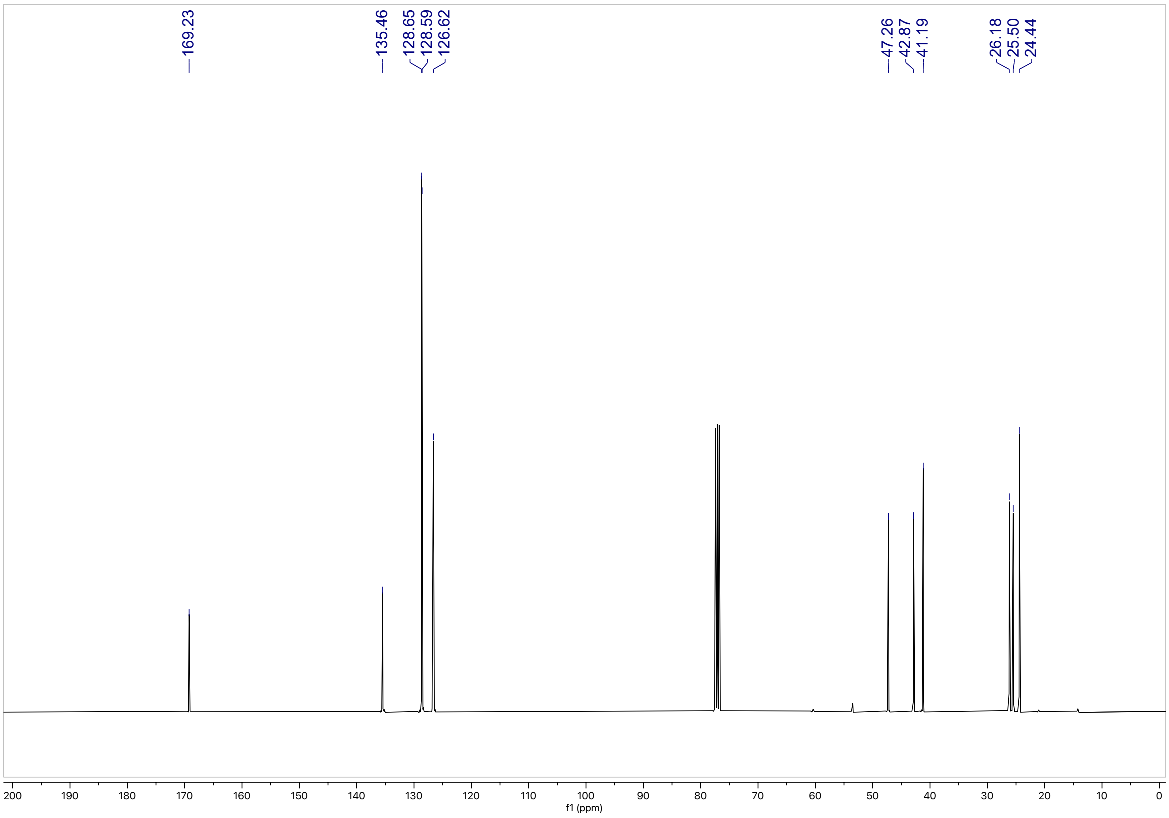

1o – ^{1}H NMR (400 MHz, $CDCl_3$)

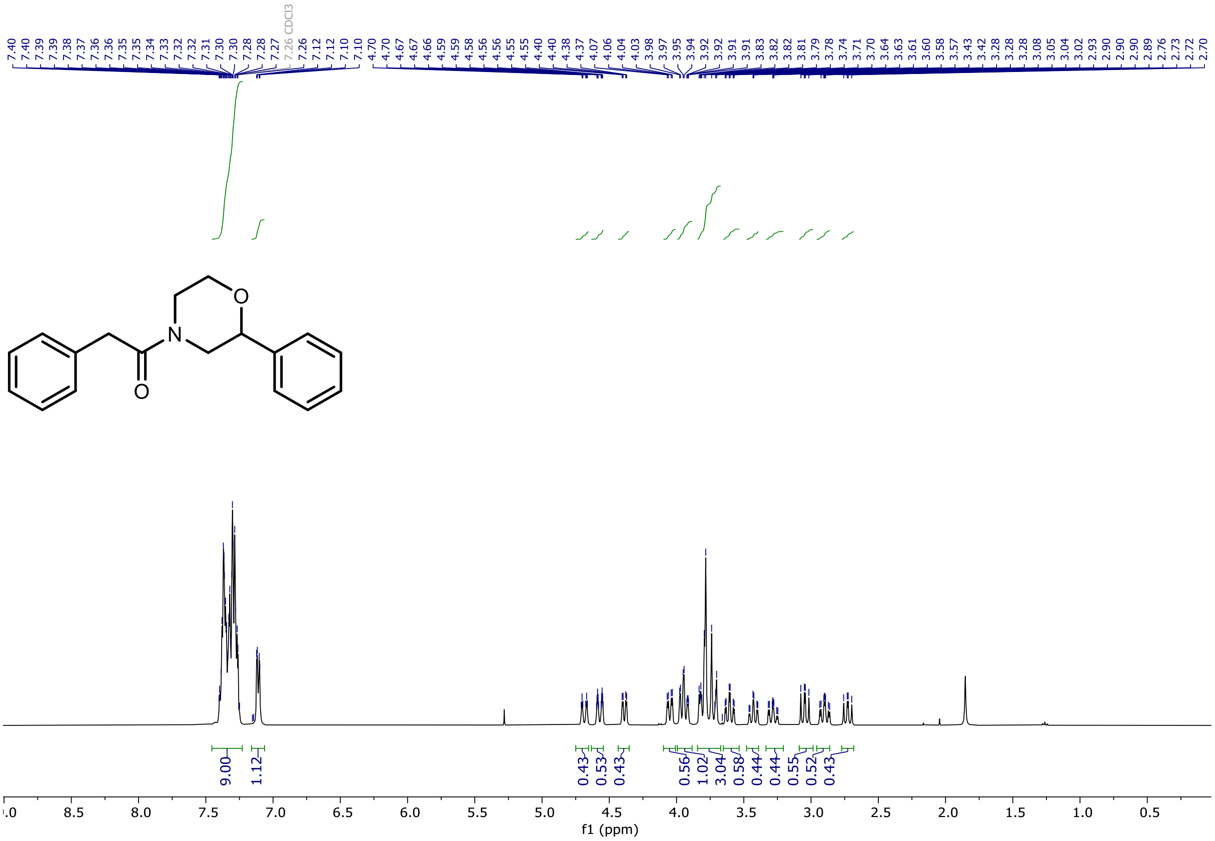

1o – ^{13}C NMR (101 MHz, $CDCl_3$)

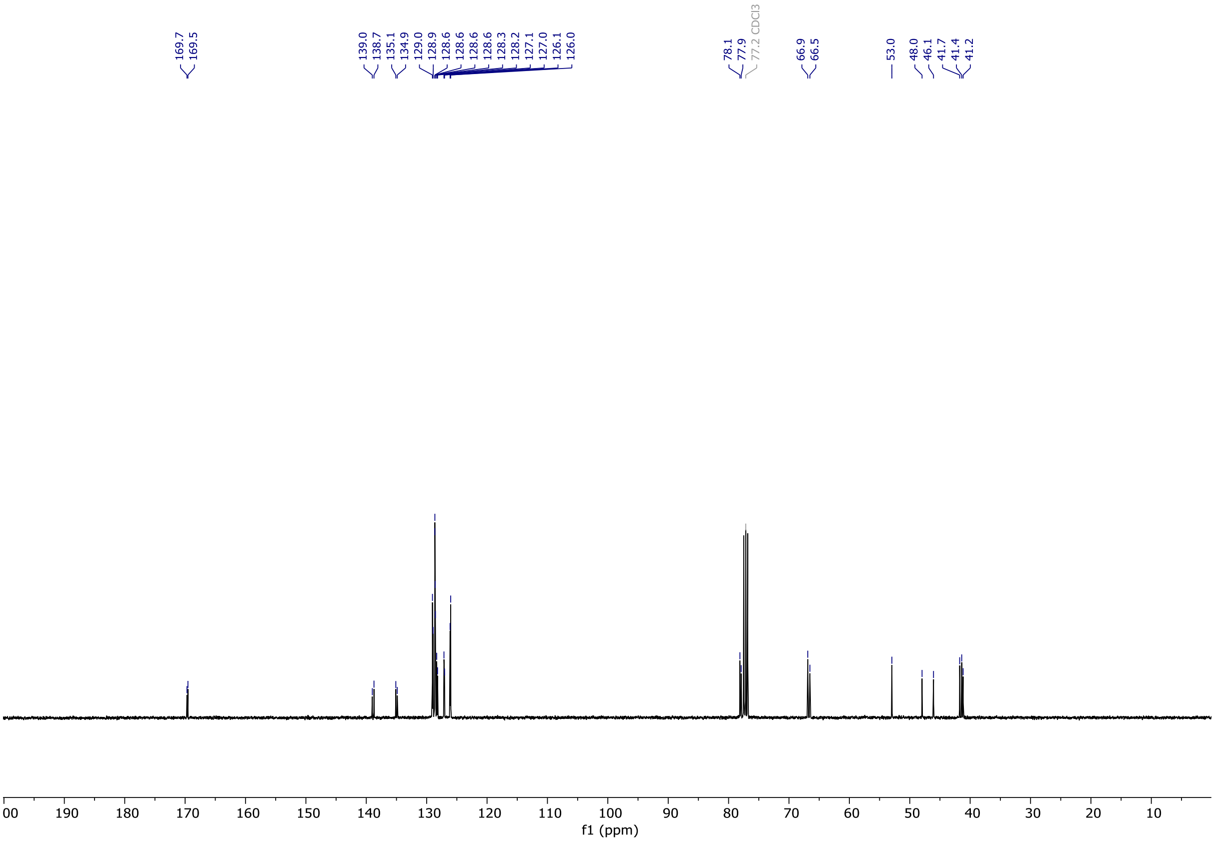

1p – ^{1}H NMR (400 MHz, CDCl$_3$)

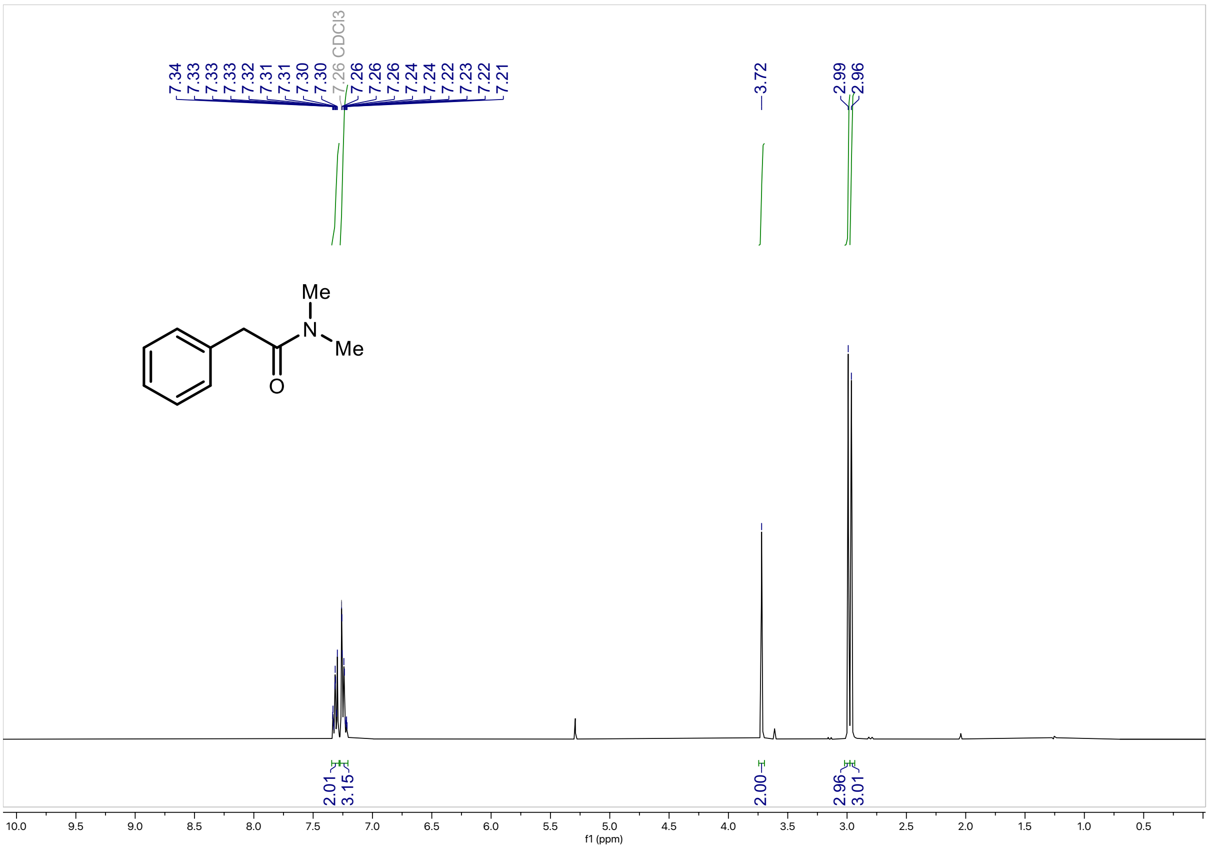

1p – ^{13}C NMR (101 MHz, CDCl$_3$)

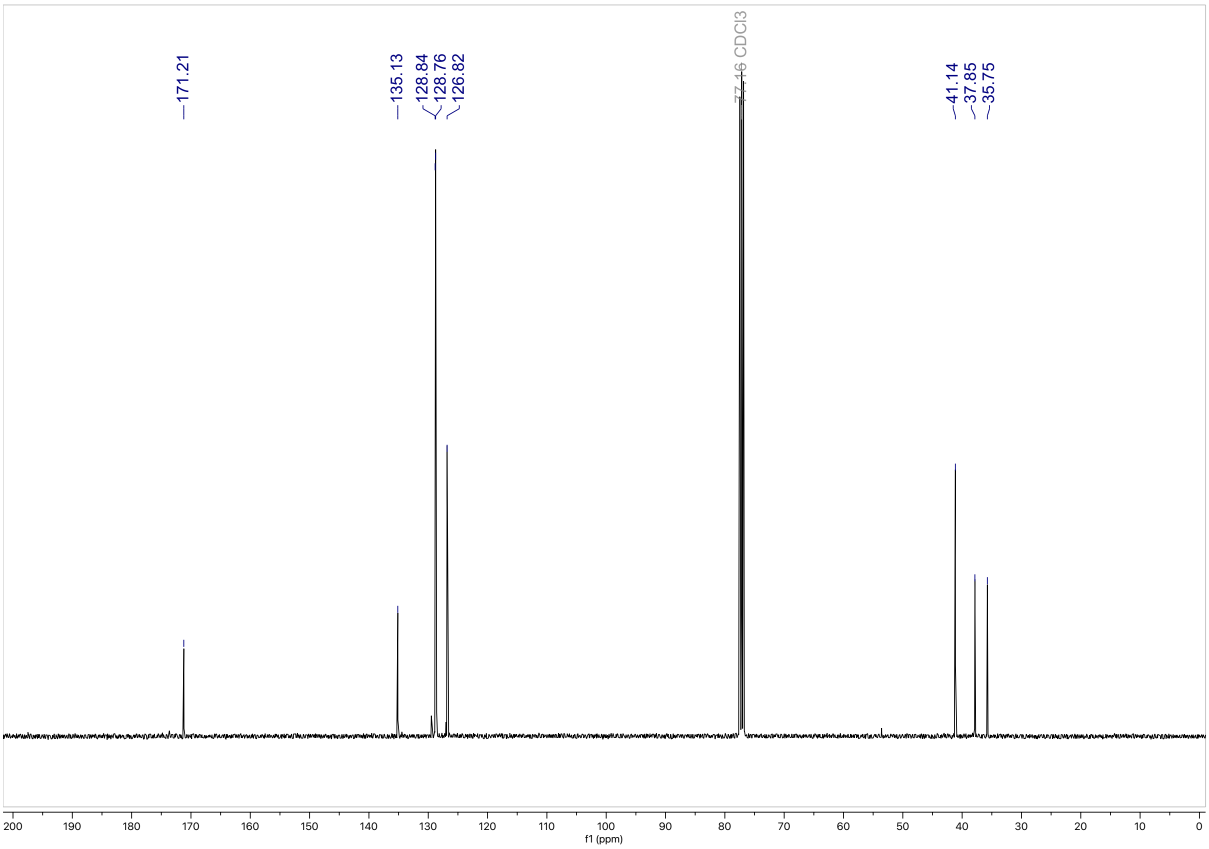

1q – ^{1}H NMR (400 MHz, $CDCl_3$)

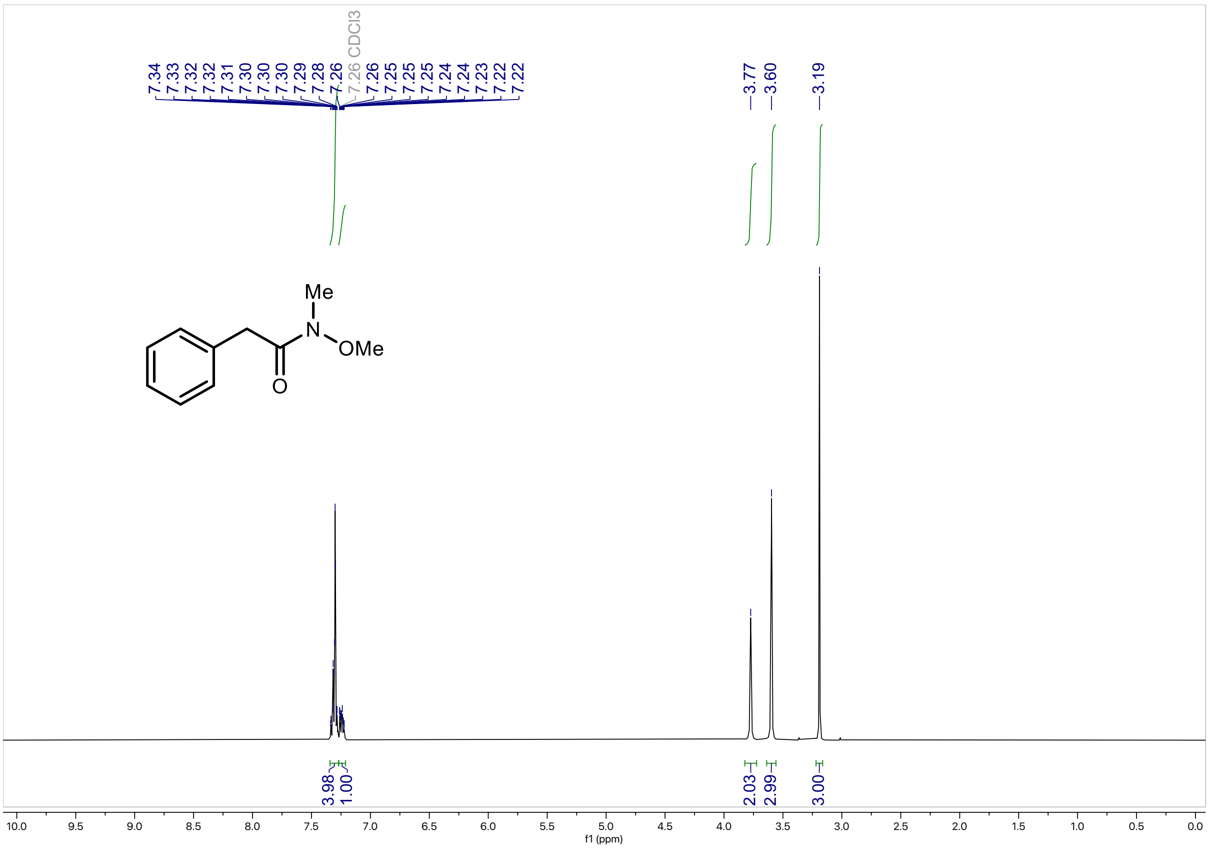

1q – ^{13}C NMR (101 MHz, $CDCl_3$)

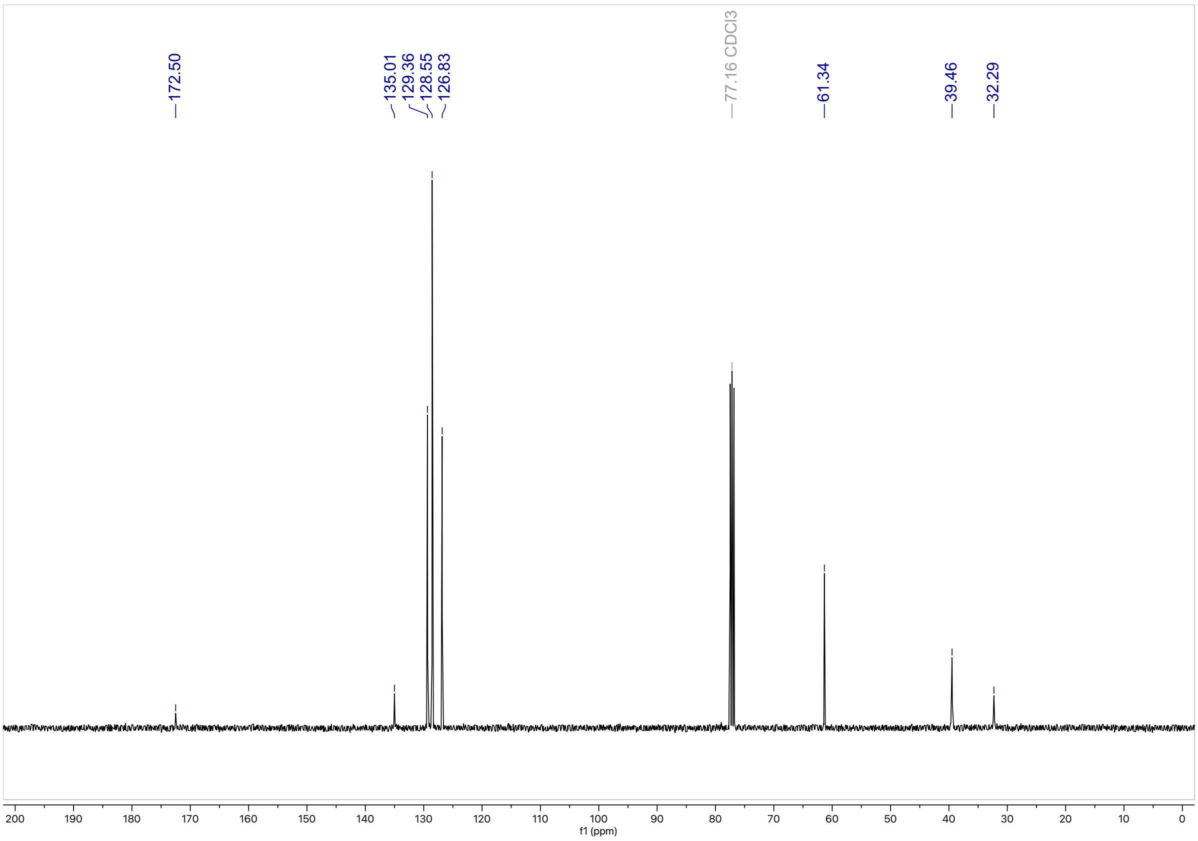

1r – ^{1}H NMR (400 MHz, CDCl$_3$)

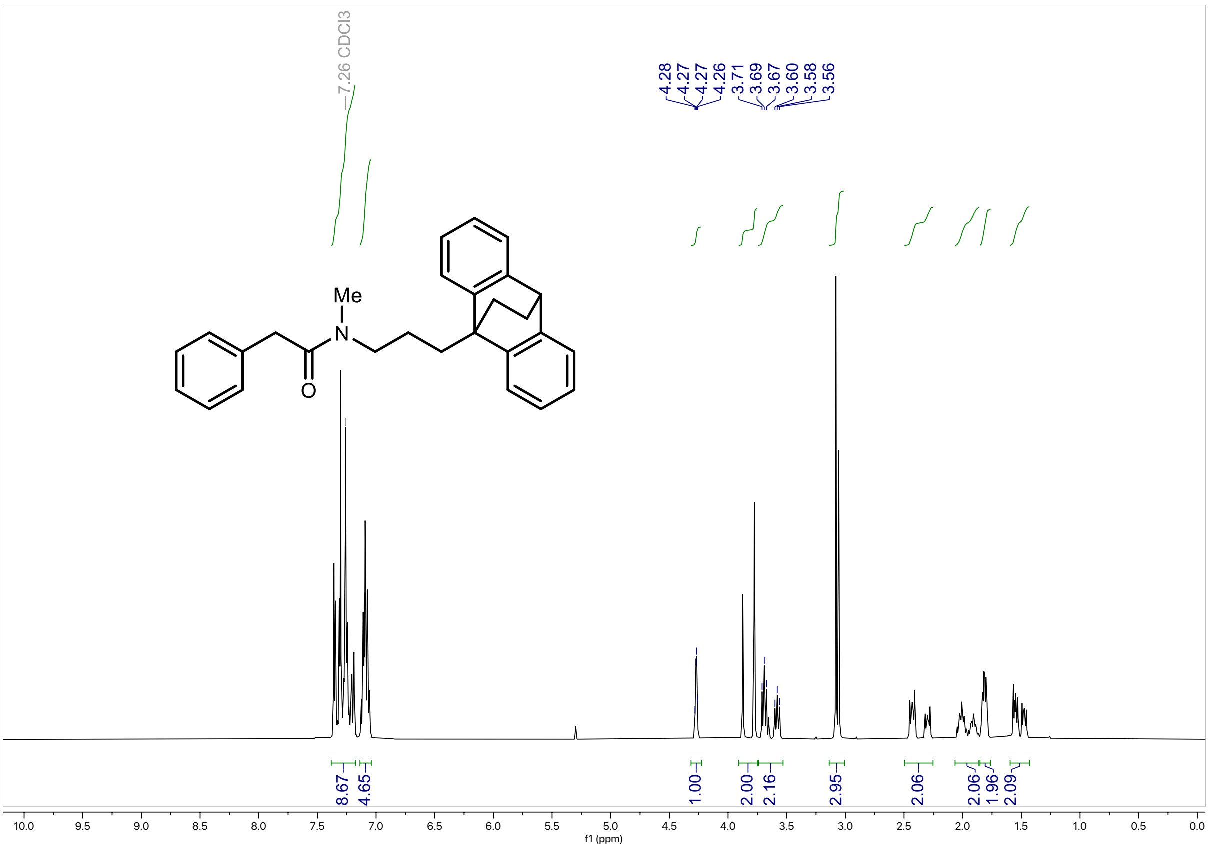

1r – ^{13}C NMR (101 MHz, CDCl$_3$)

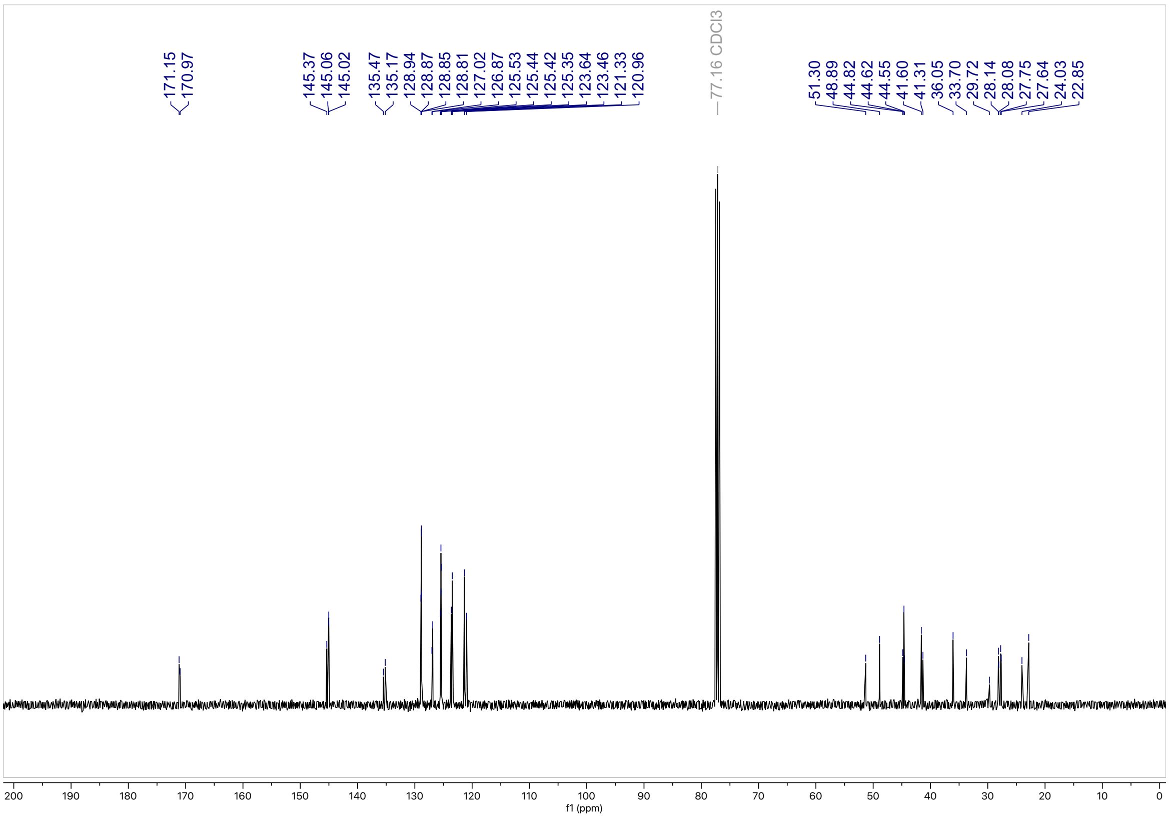

1s – ^{1}H NMR (400 MHz, CDCl$_3$)

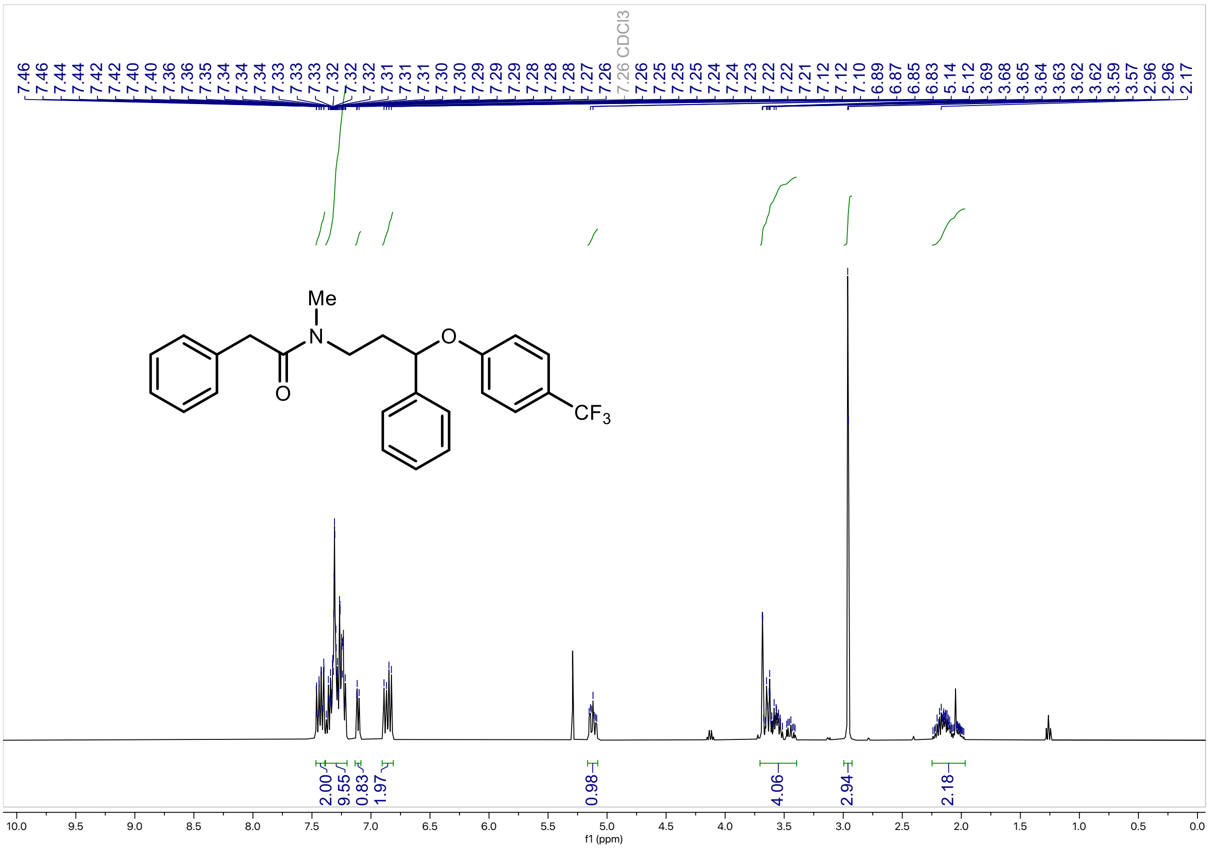

1s – ^{13}C NMR (101 MHz, CDCl$_3$)

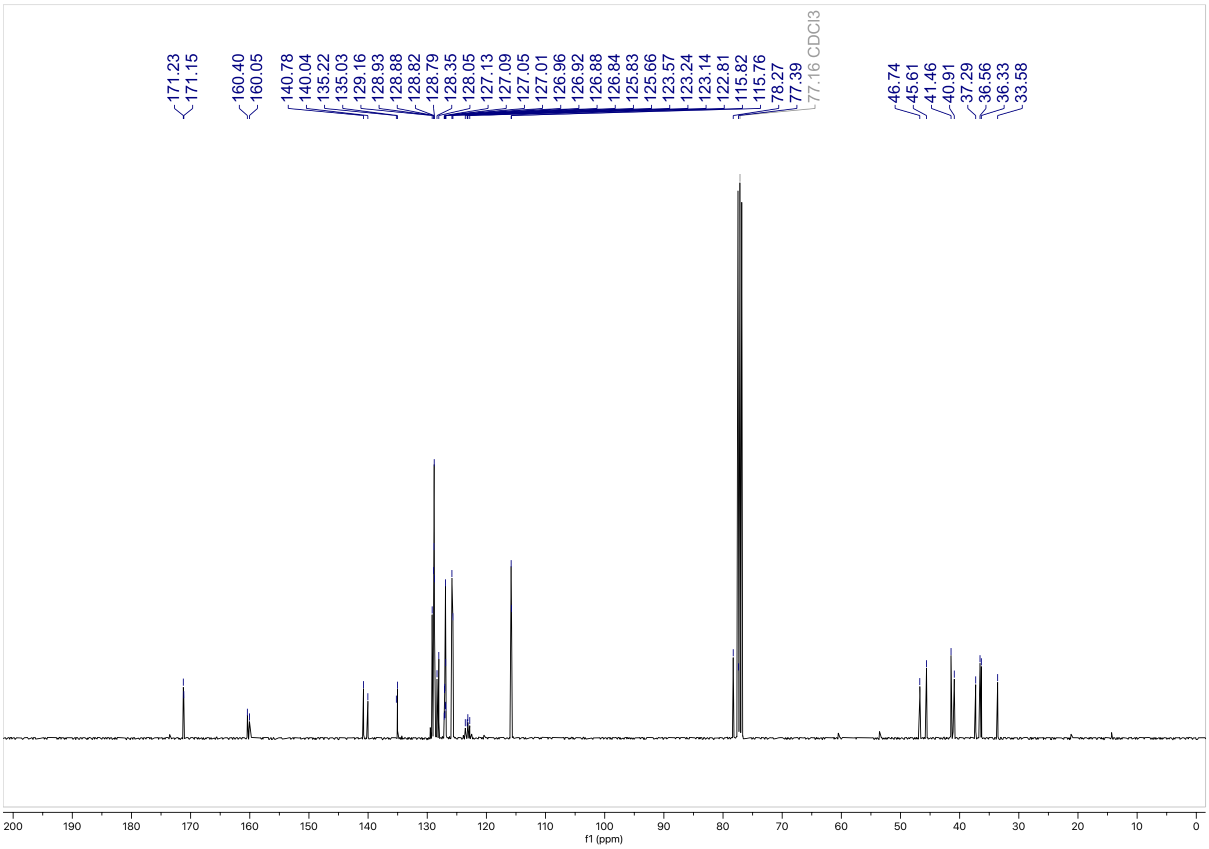

1ab – ¹H NMR (400 MHz, $CDCl_3$)

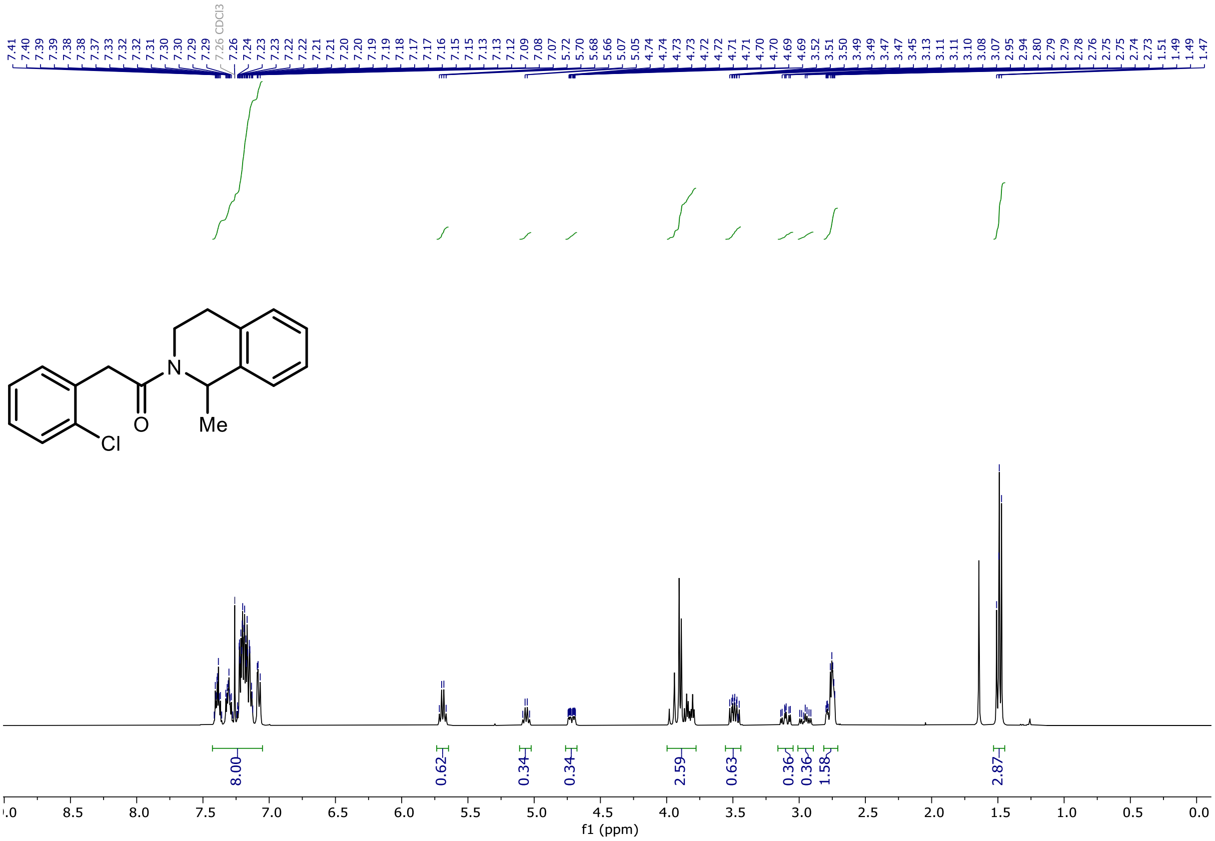

1ab – ¹³C NMR (101 MHz, $CDCl_3$)

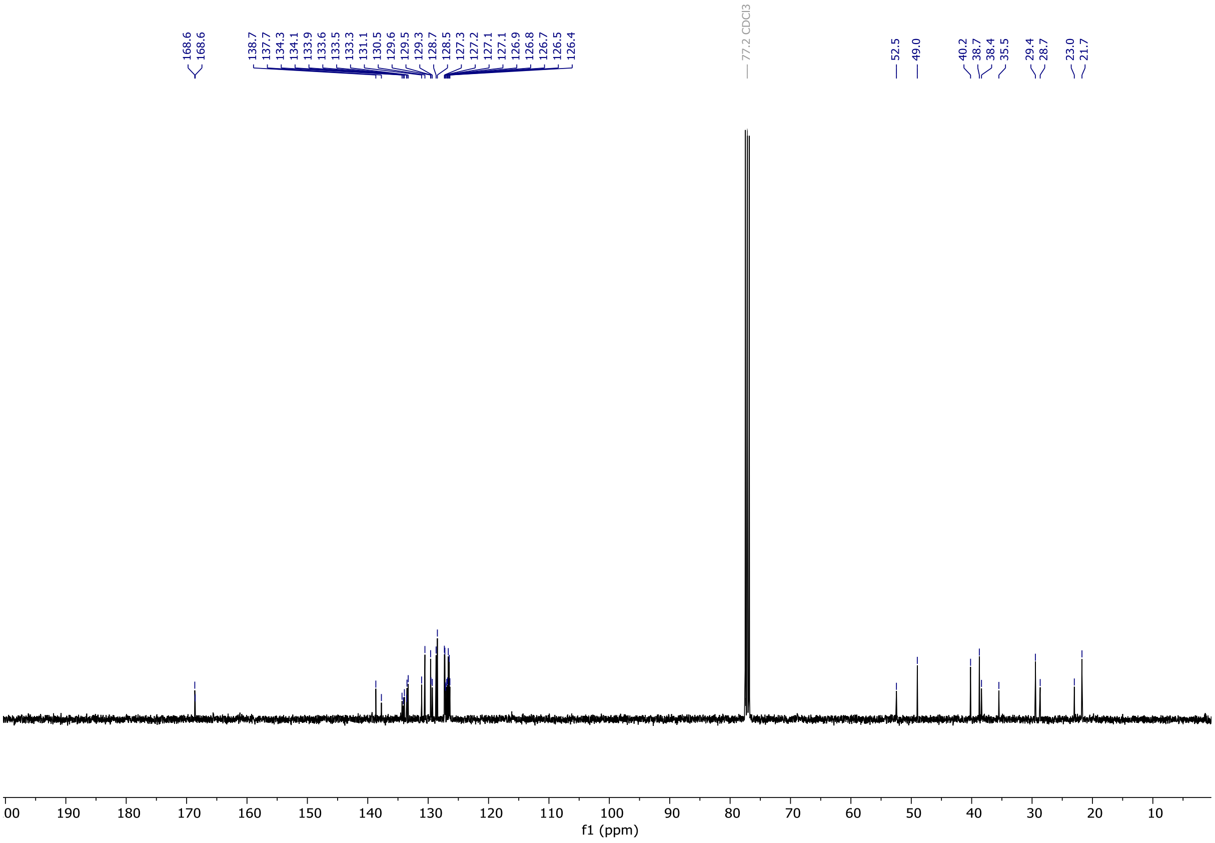

Tetrahydro-2H-thiopyran 1-oxide– ^{1}H NMR (400 MHz, $CDCl_3$)

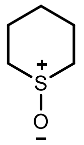

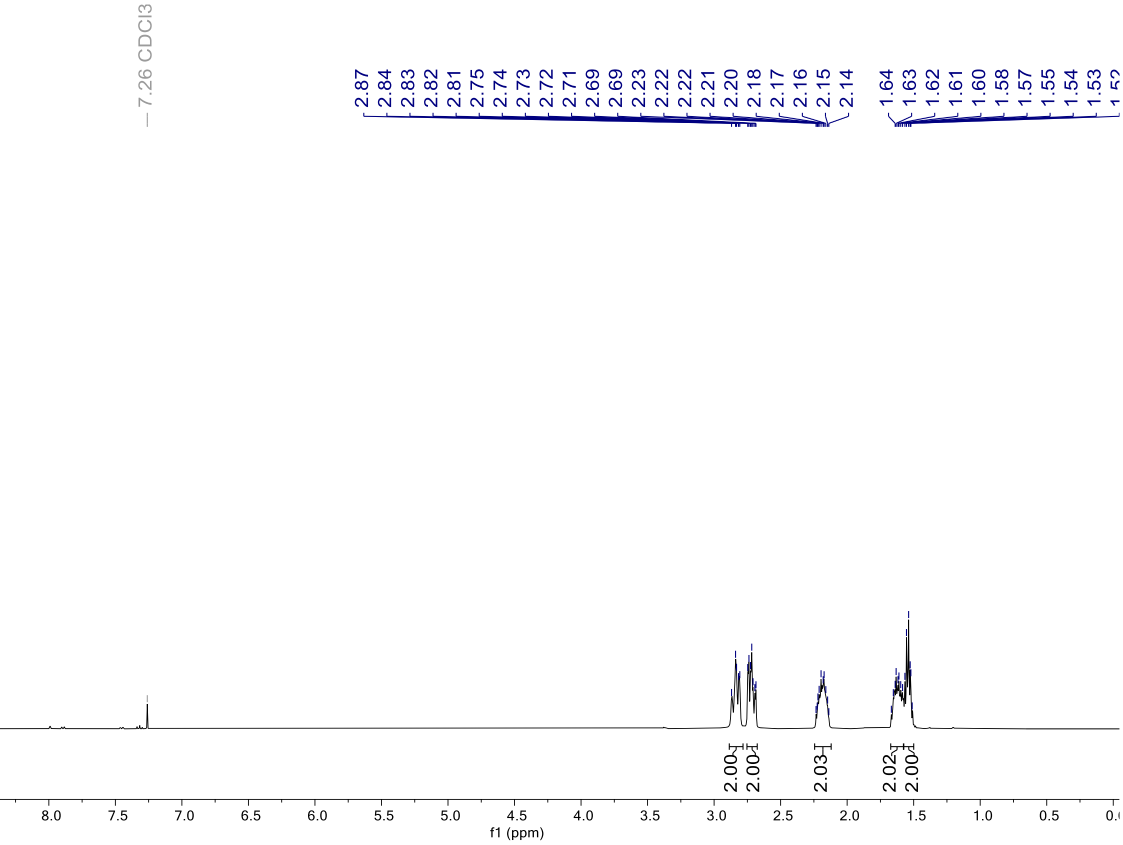

Tetrahydro-2H-thiopyran 1-oxide– ^{13}C NMR (101 MHz, $CDCl_3$)

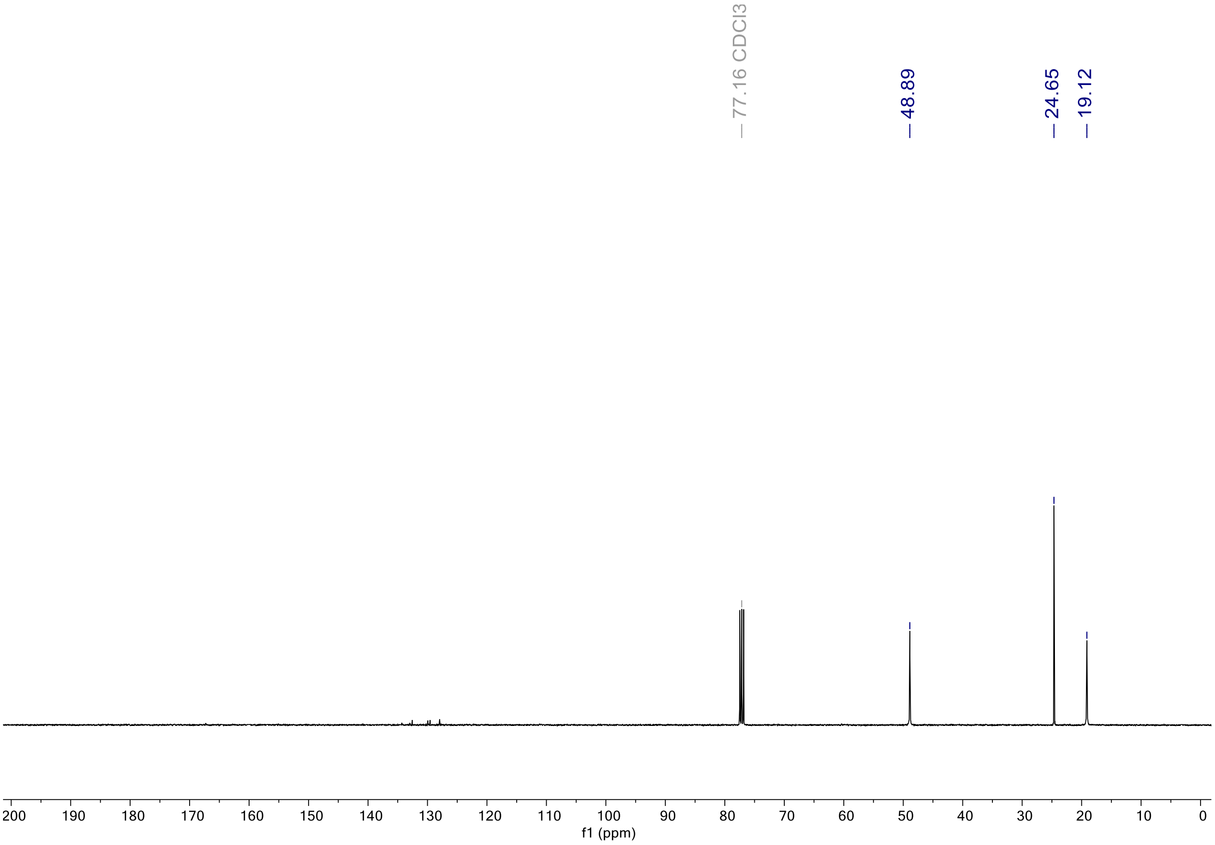

1,4-Oxathiane 4-oxide – ^{1}H NMR (400 MHz, $CDCl_3$)

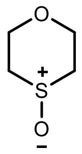

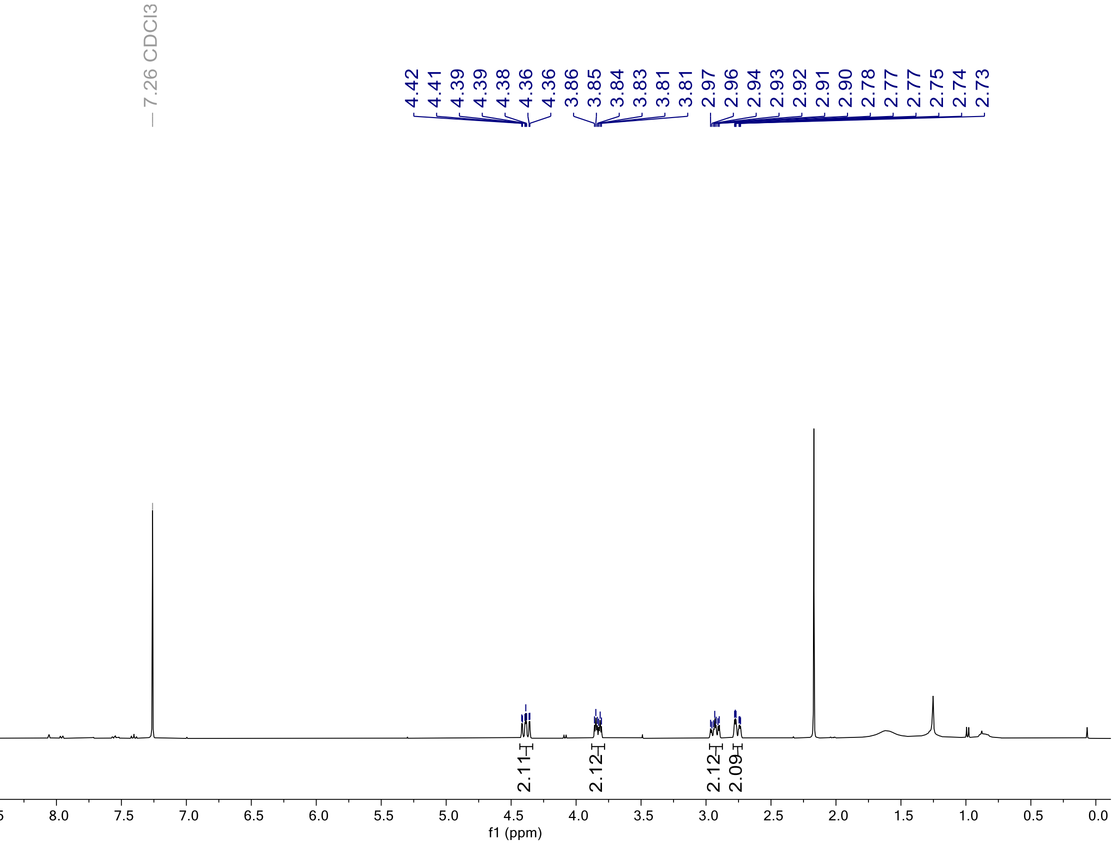

1,4-Oxathiane 4-oxide – ^{13}C NMR (101 MHz, $CDCl_3$)

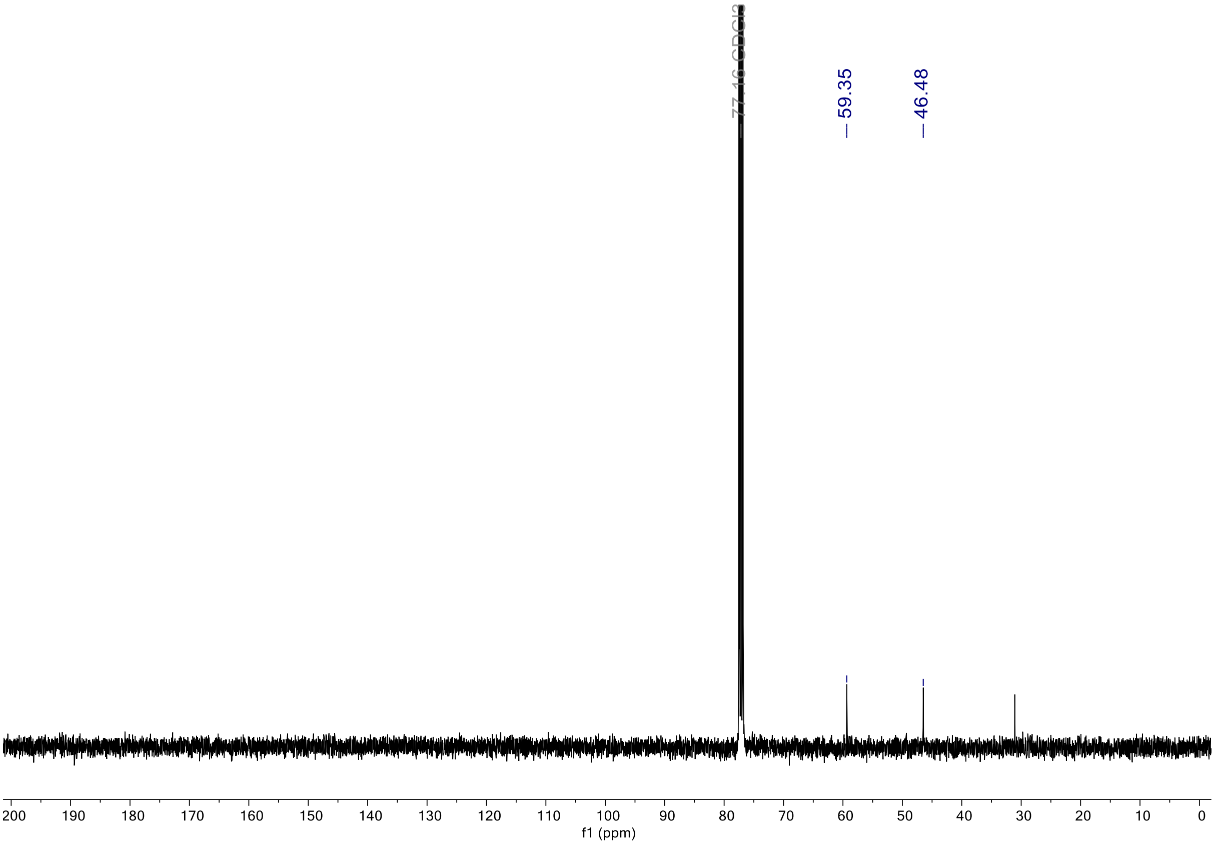

Tetrahydro-2H-thiopyran-4-carbonitrile 1-oxide – ^{1}H NMR (400 MHz, $CDCl_3$)

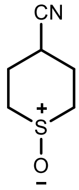

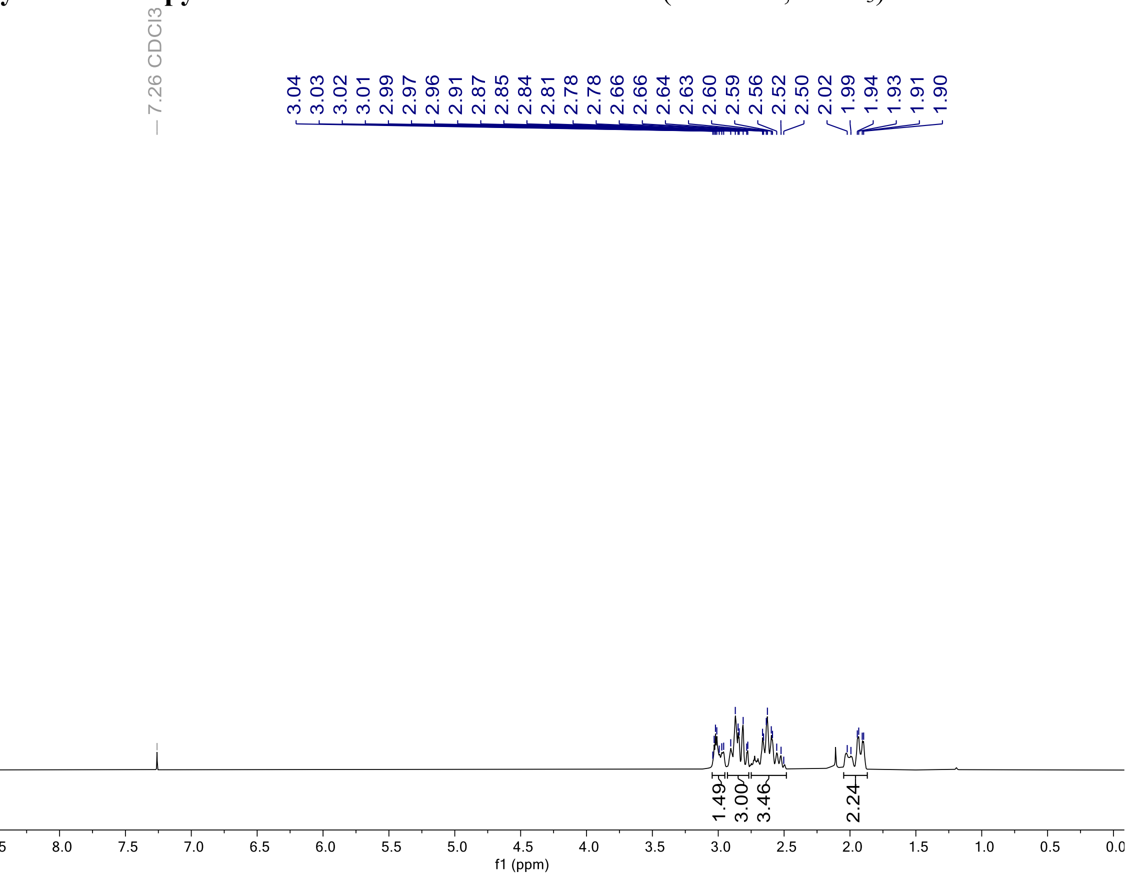

Tetrahydro-2H-thiopyran-4-carbonitrile 1-oxide – ^{13}C NMR (101 MHz, $CDCl_3$)

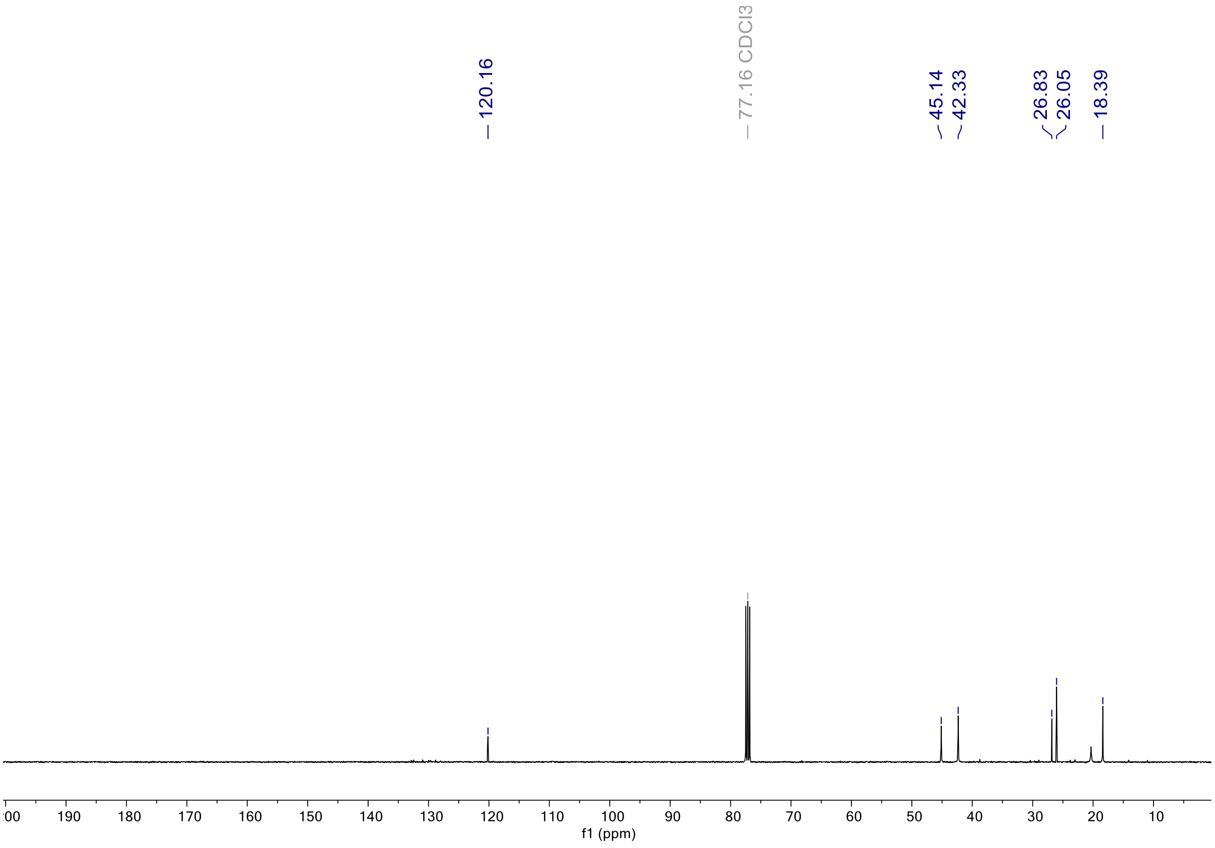

(Methylsulfinyl)ethane – ^{1}H NMR (400 MHz, $CDCl_3$)

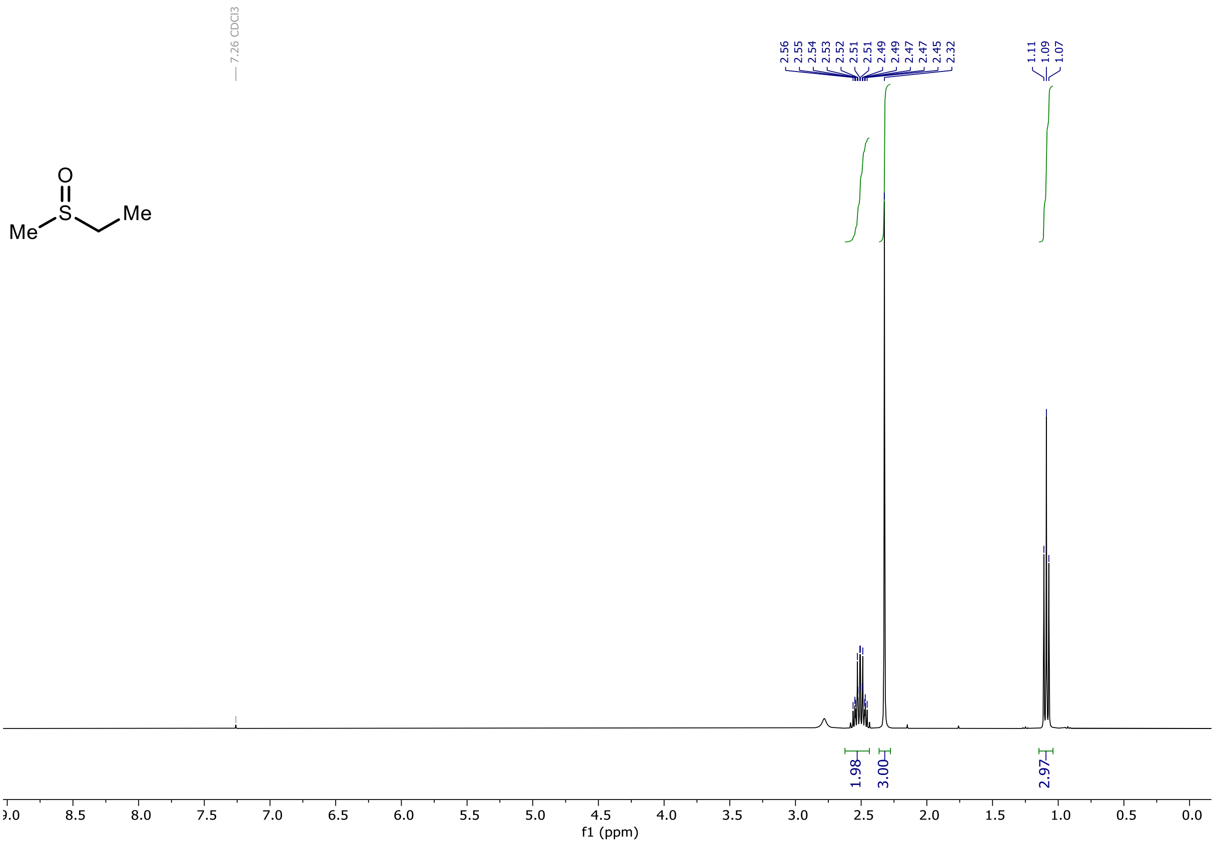

(Methylsulfinyl)ethane – ^{13}C NMR (101 MHz, $CDCl_3$)

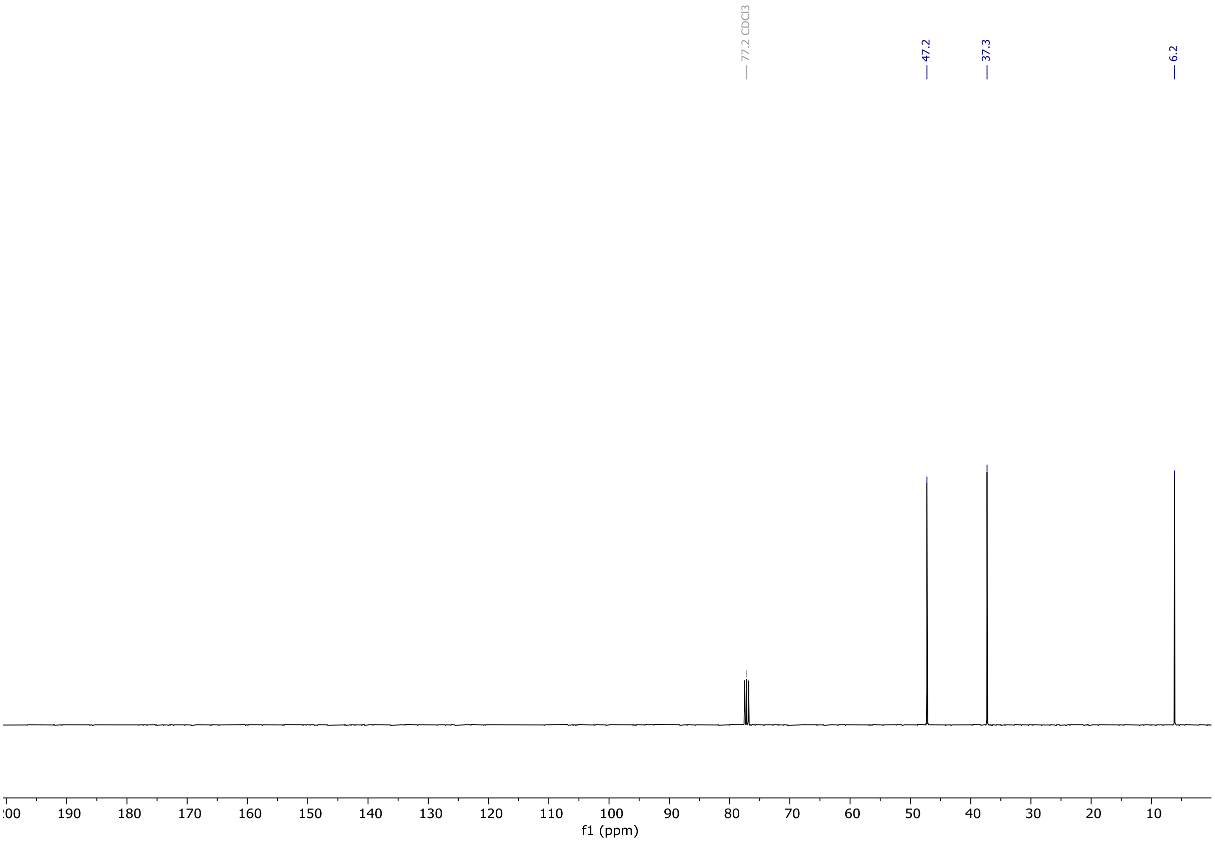

2a – ^{1}H NMR (400 MHz, $CDCl_3$)

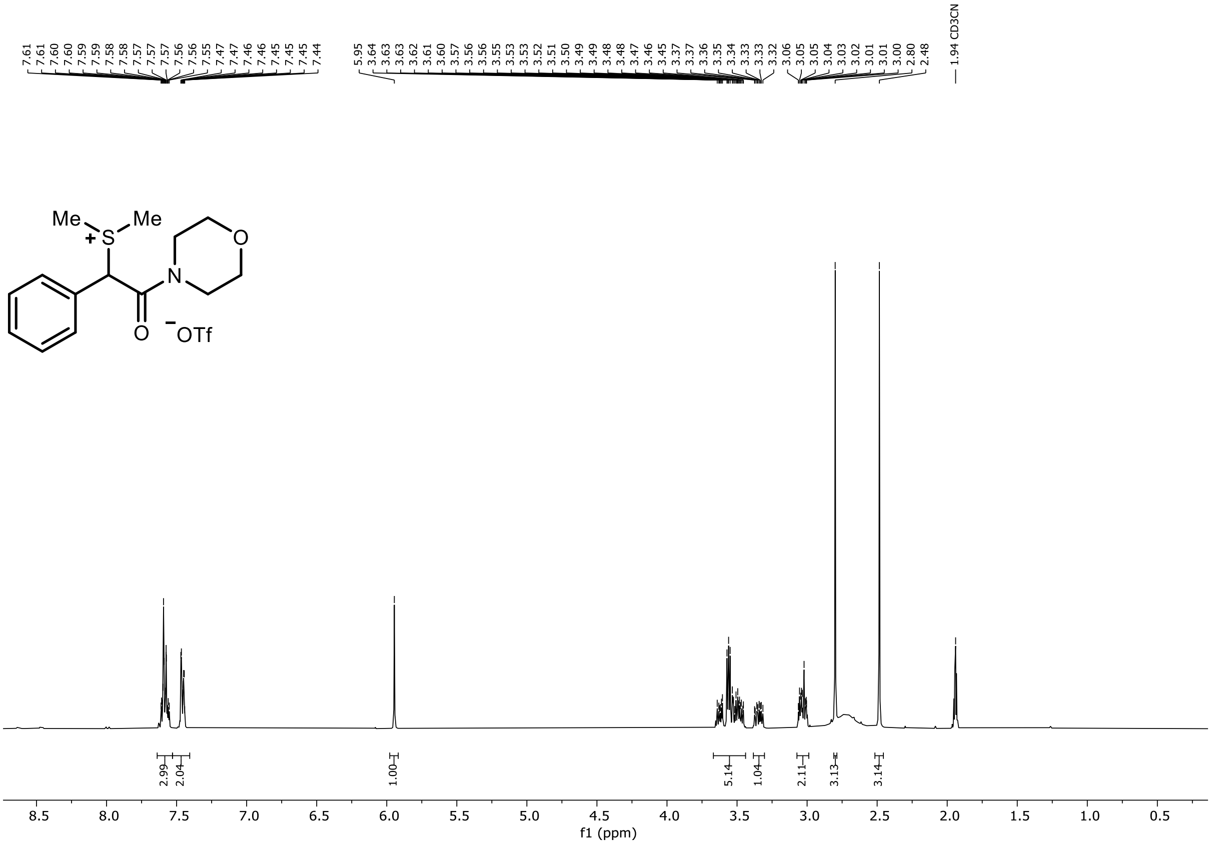

2a – ^{13}C NMR (101 MHz, $CDCl_3$)

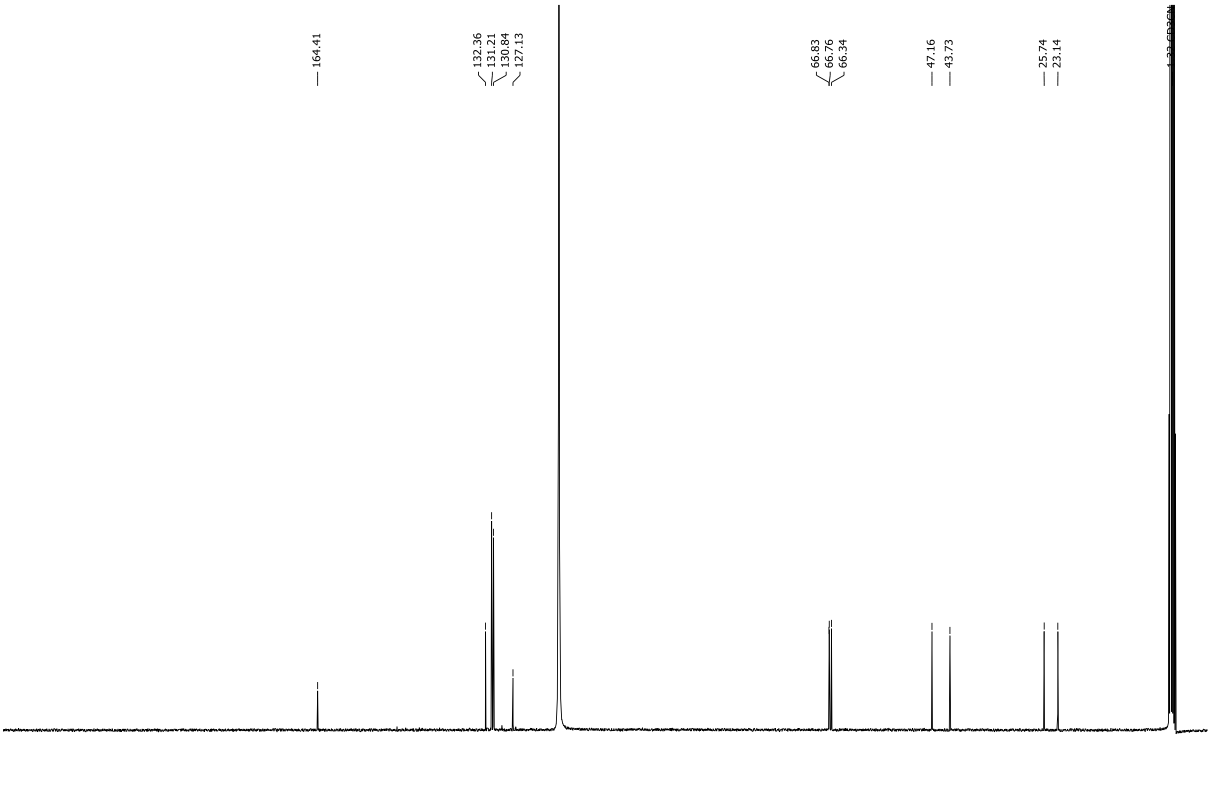

2i – ^{19}F NMR (376 MHz, $CDCl_3$)

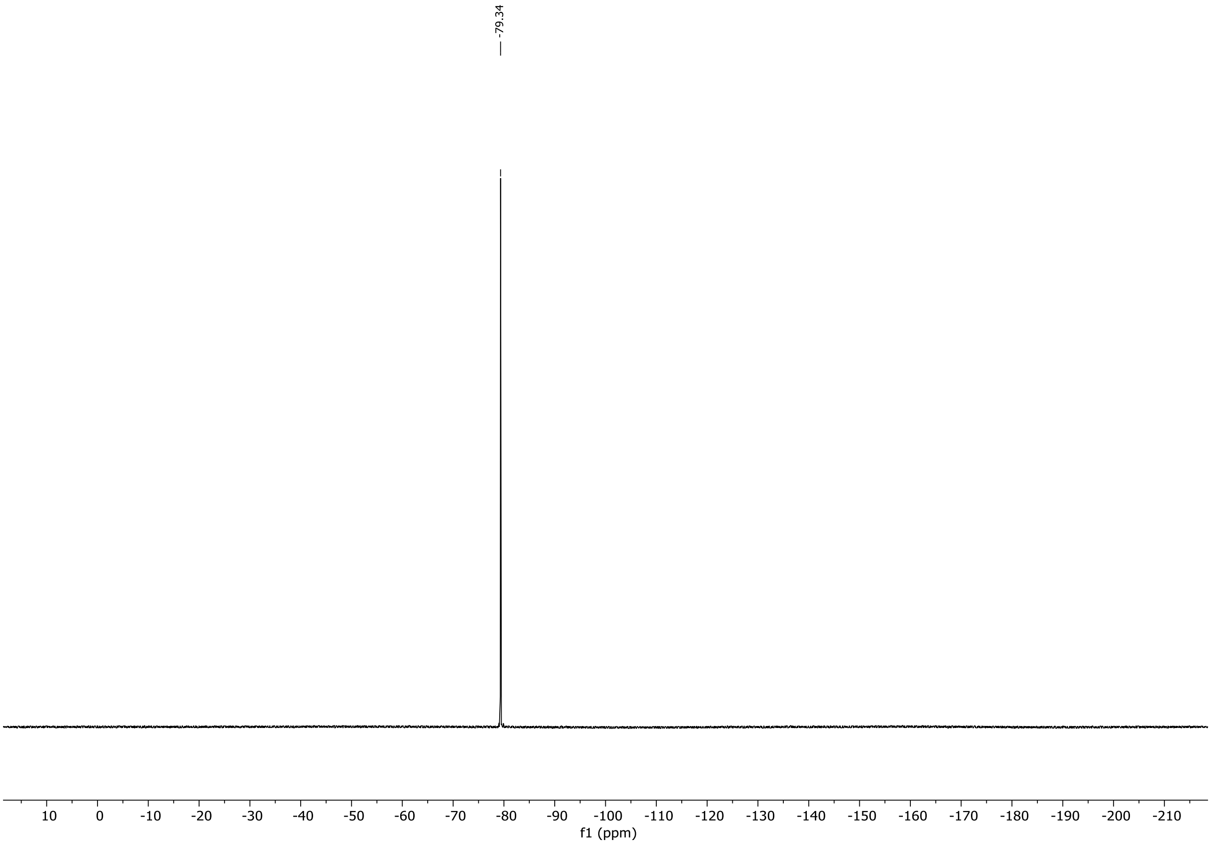

3a – ^{1}H NMR (500 MHz, CD_3CN)

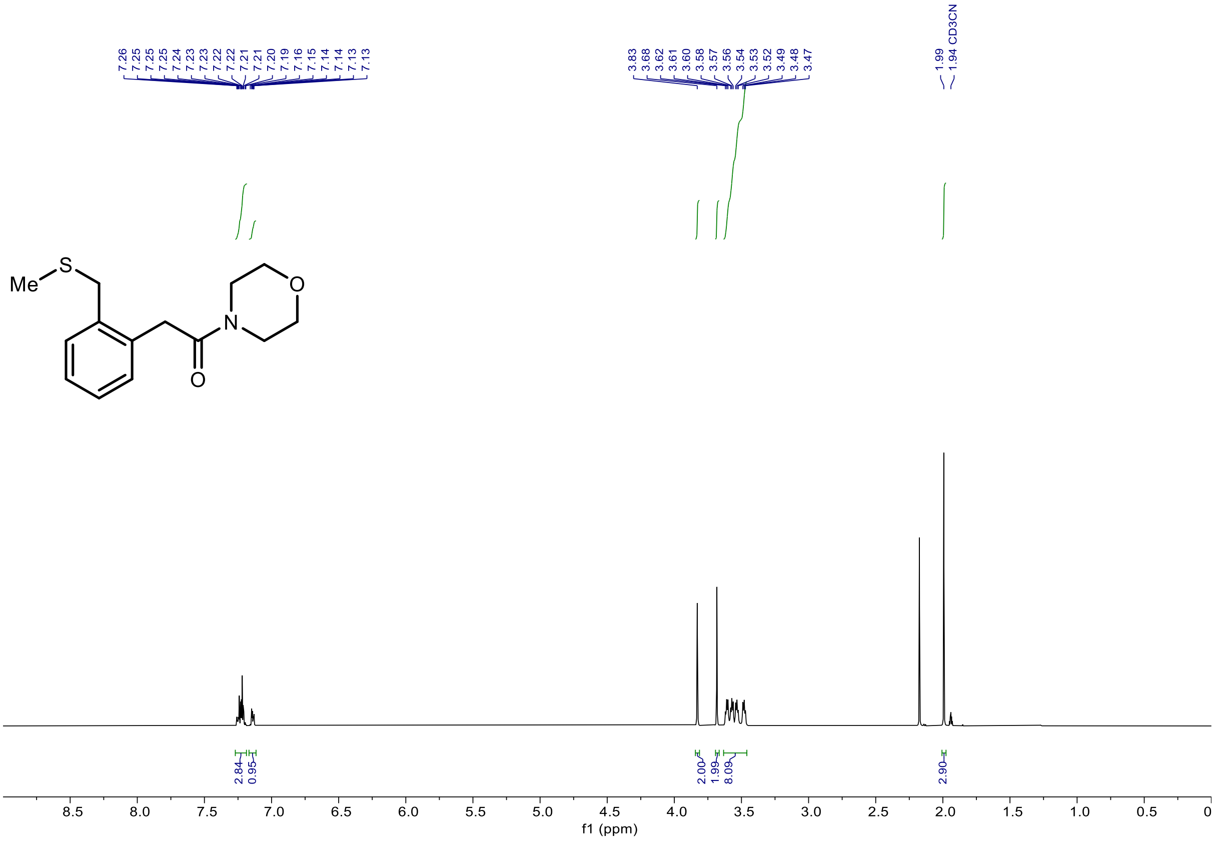

3a – ^{13}C NMR (126 MHz, CD_3CN)

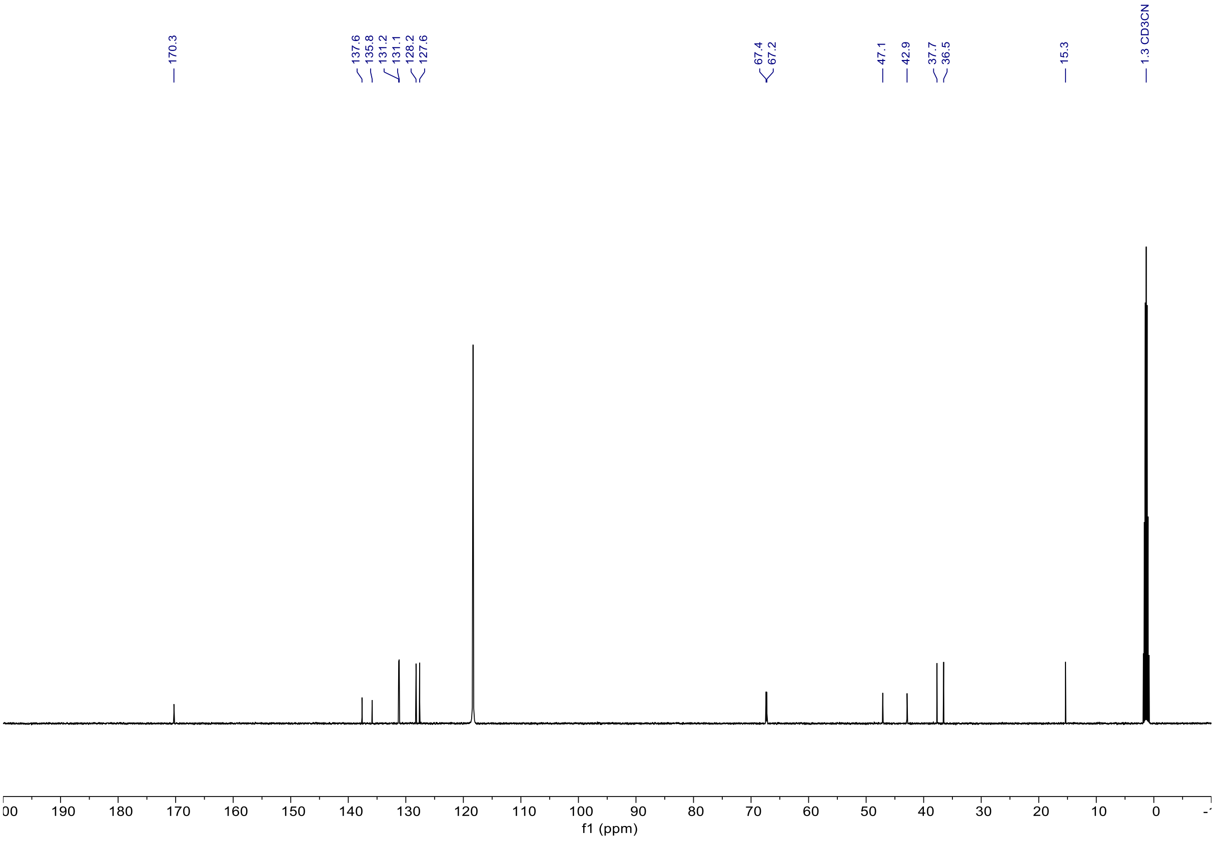

2b – ^{1}H NMR (500 MHz, CD_3CN)

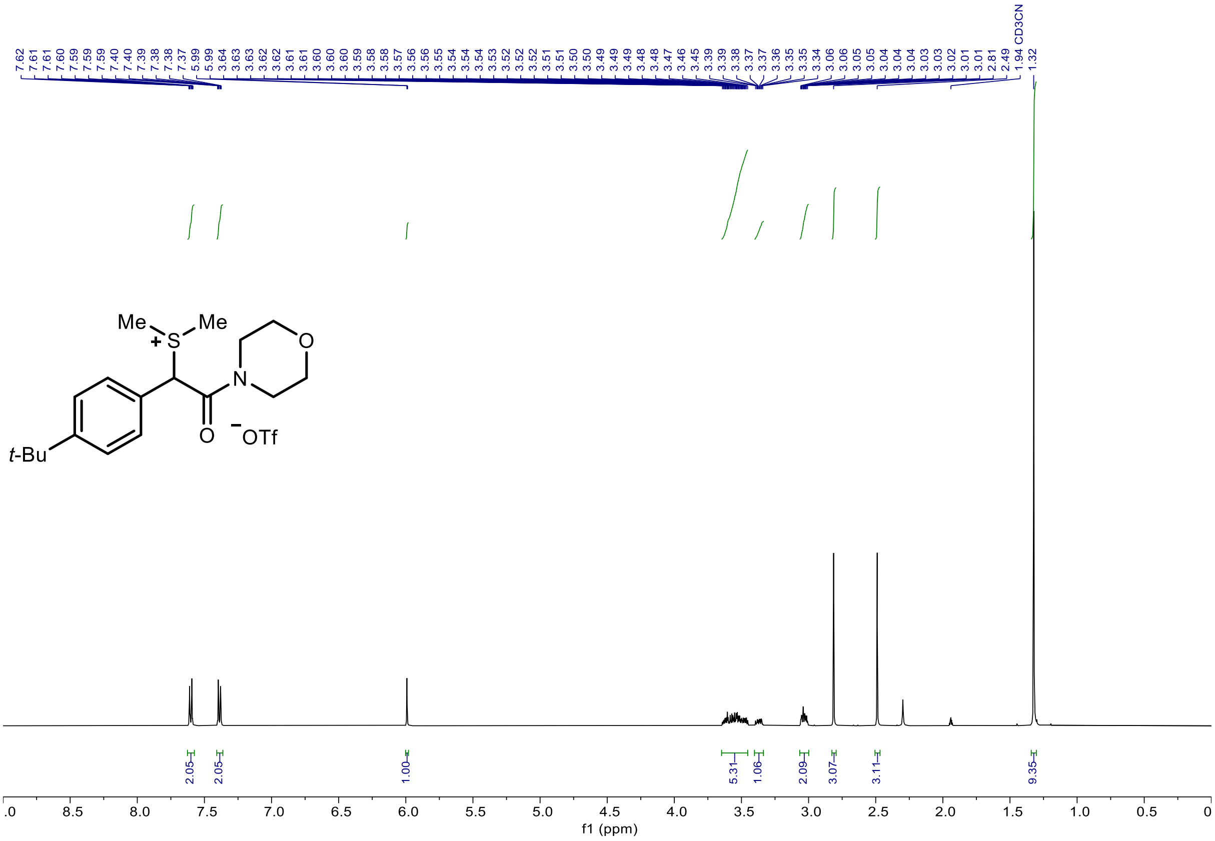

2b – ^{13}C NMR (126 MHz, CD_3CN)

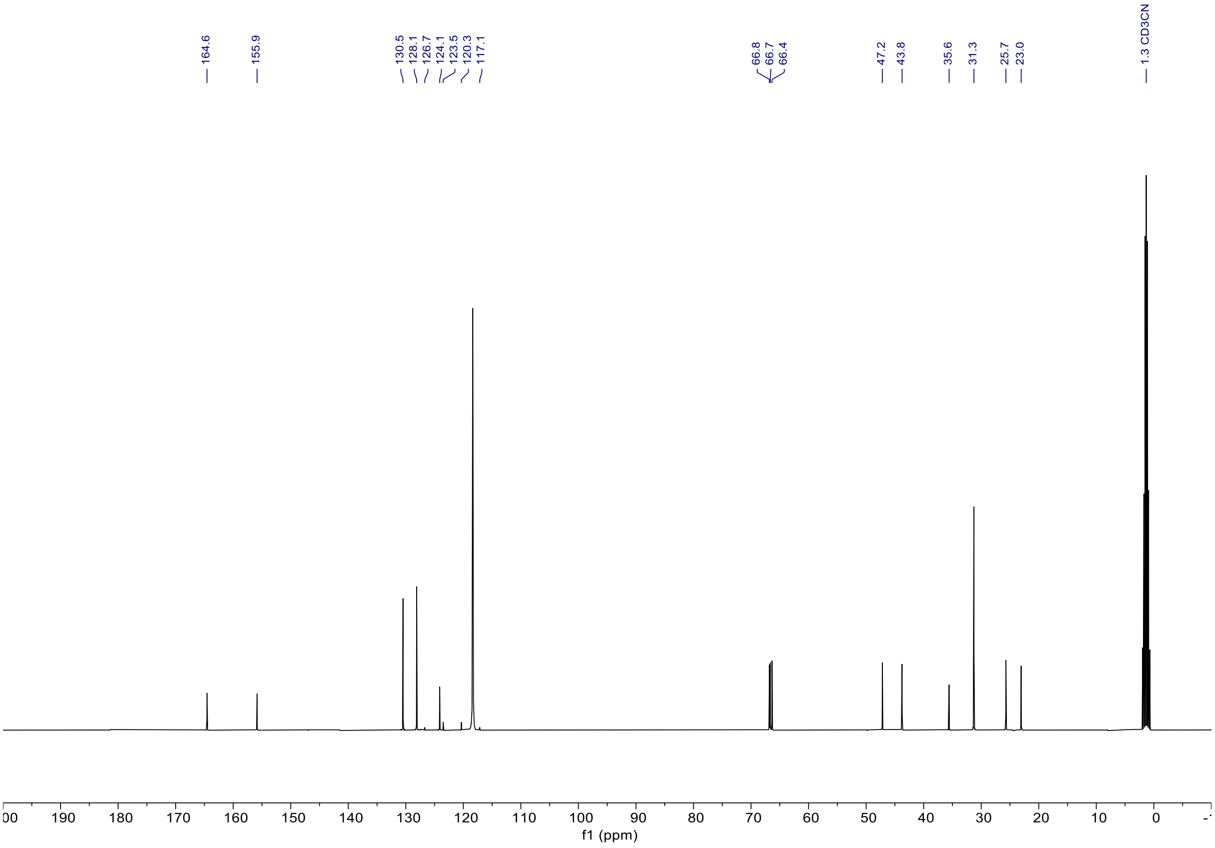

2b – ^{19}F NMR (376 MHz, CD_3CN)

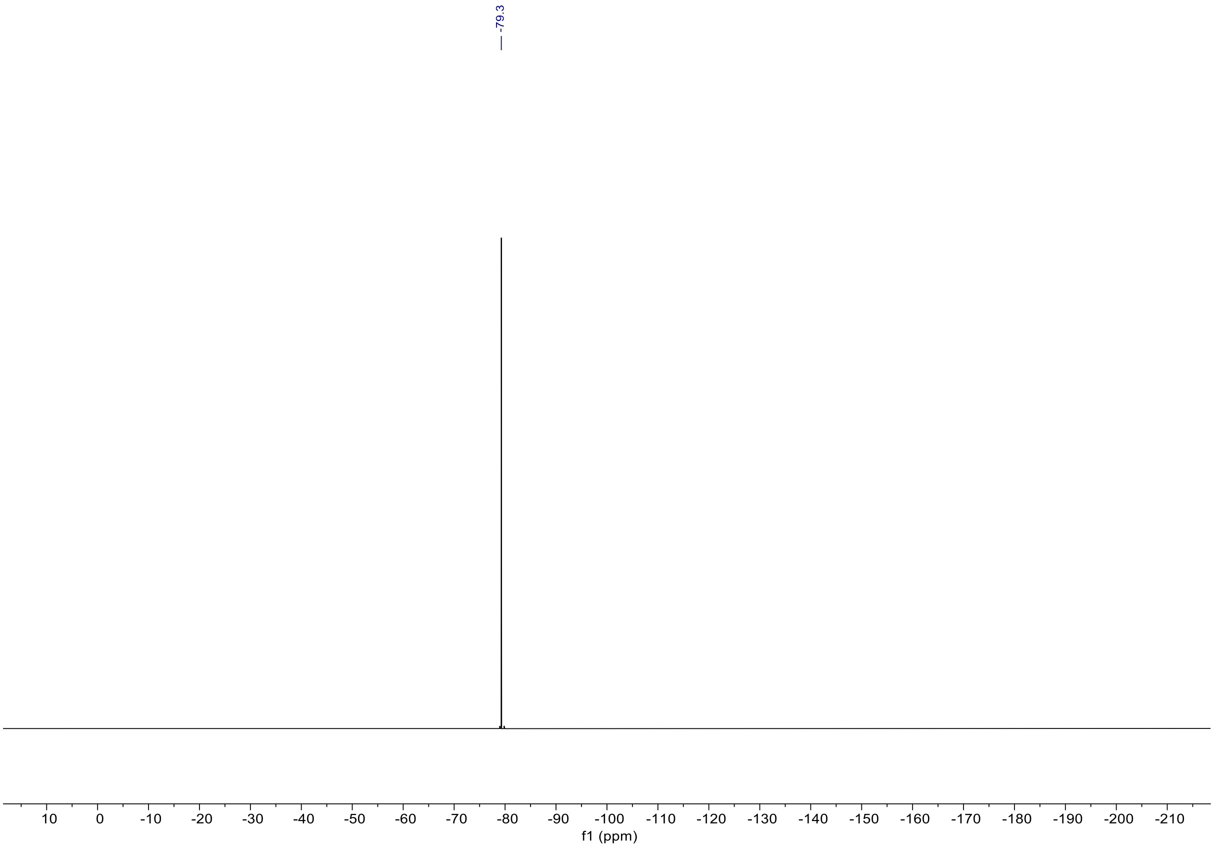

3b – ^{1}H NMR (500 MHz, CDCl$_3$)

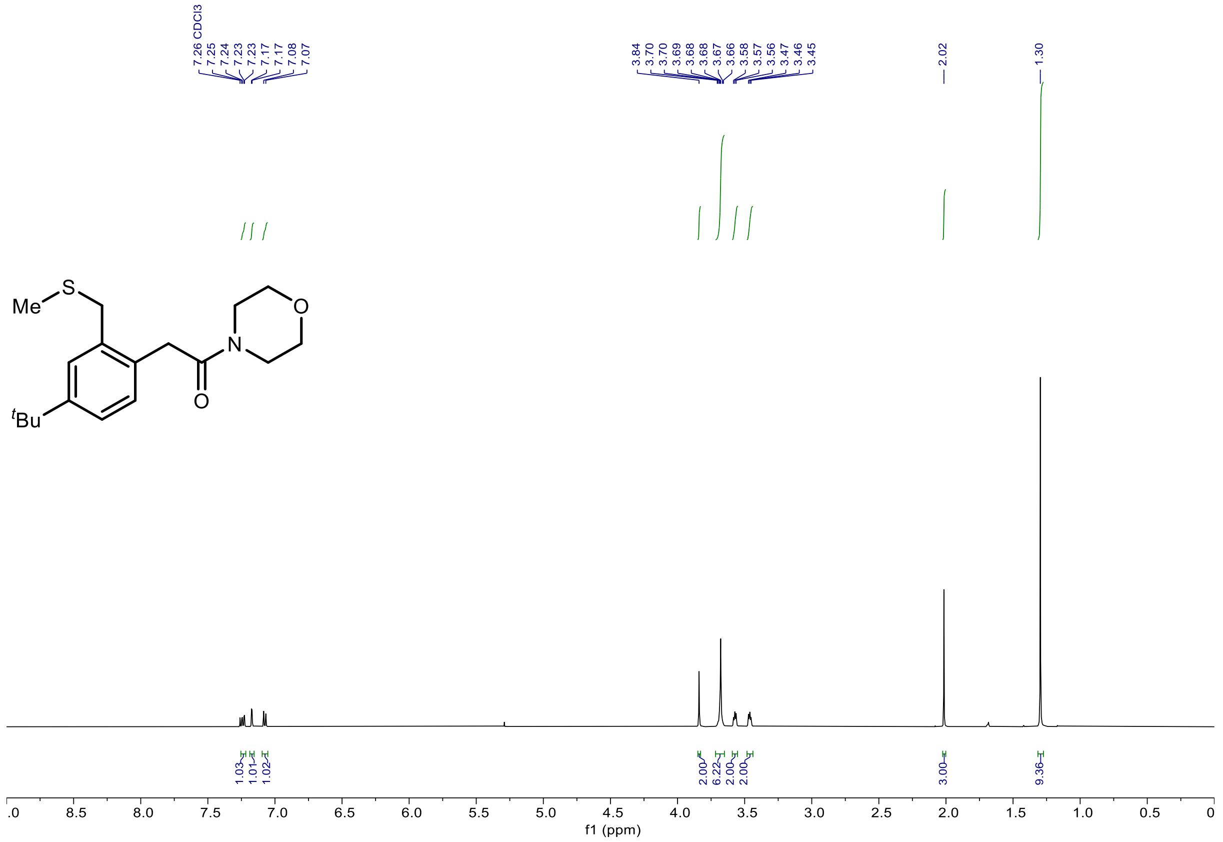

3b – ^{13}C NMR (126 MHz, CDCl$_3$)

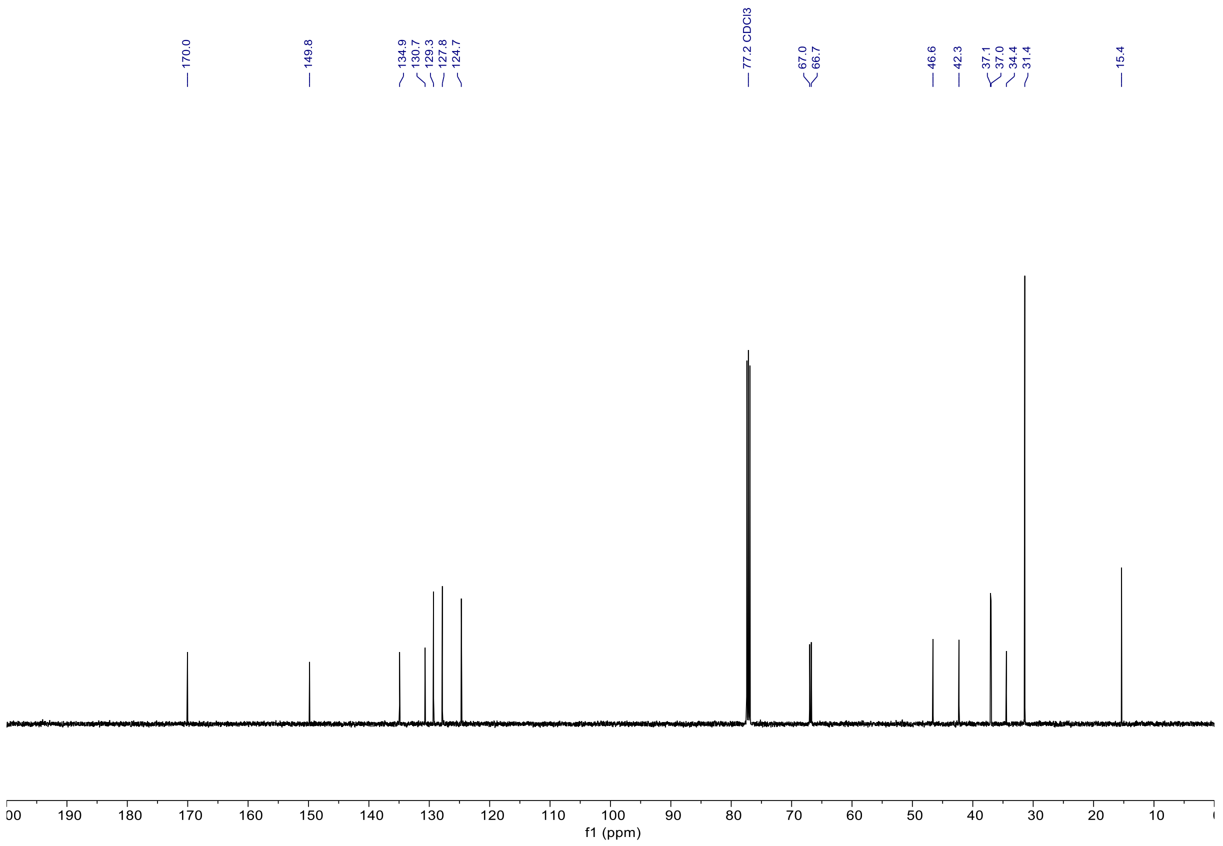

2c – ^{1}H NMR (400 MHz, $CDCl_3$)

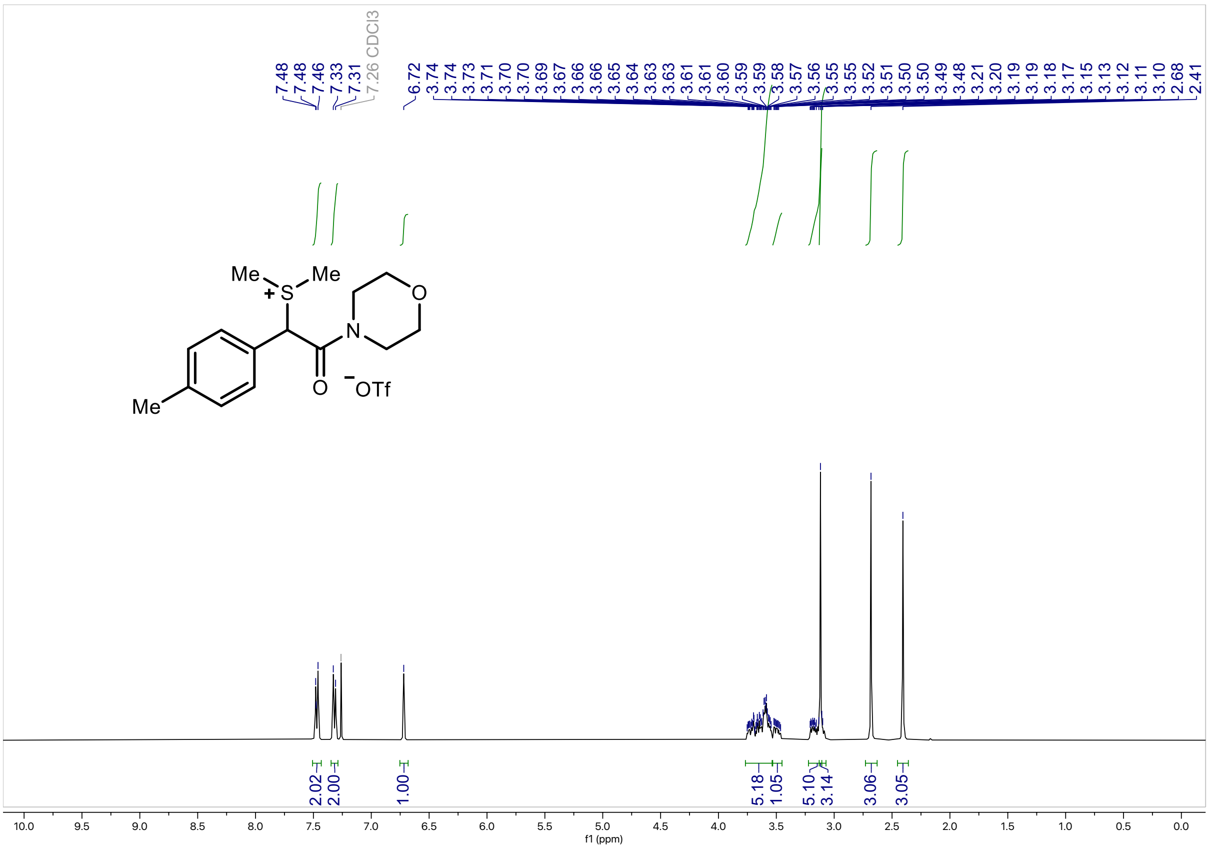

2c – ^{13}C NMR (101 MHz, $CDCl_3$)

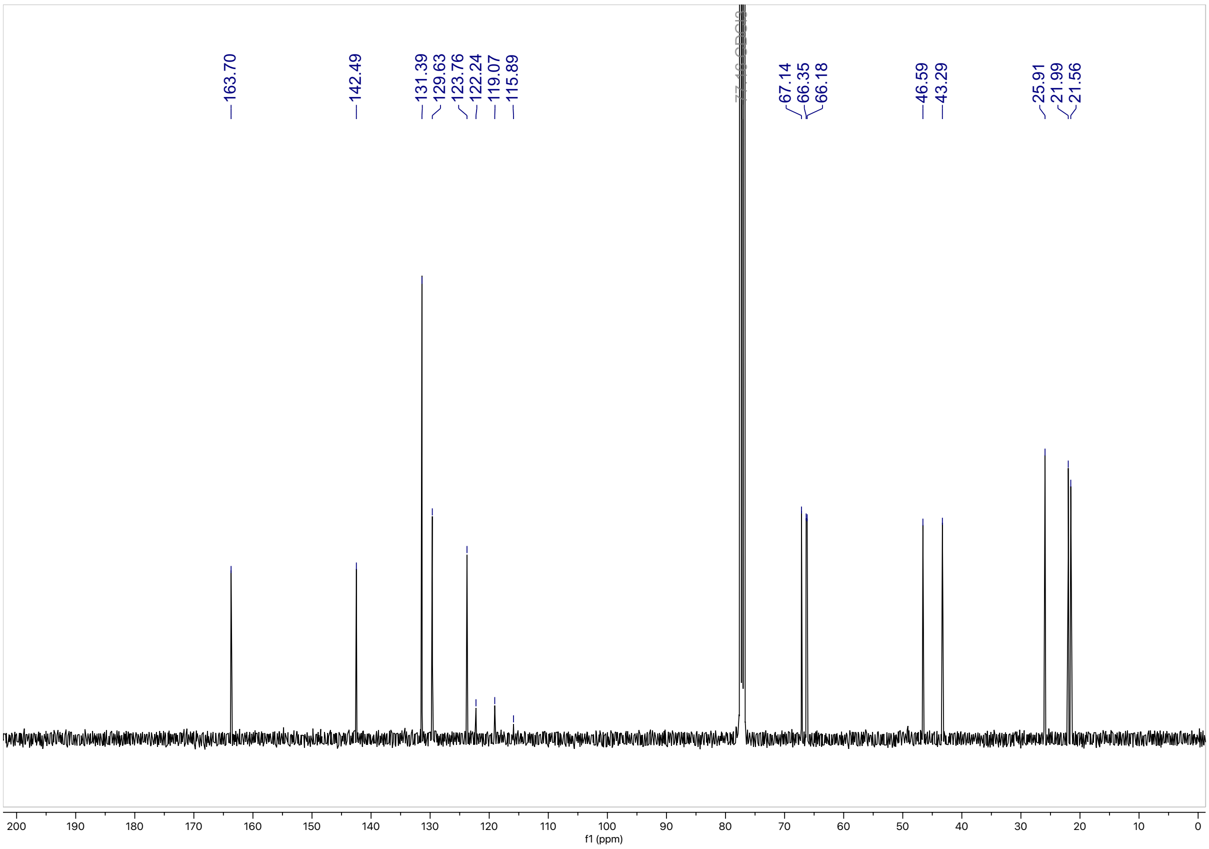

2c – ^{19}F NMR (376 MHz, $CDCl_3$)

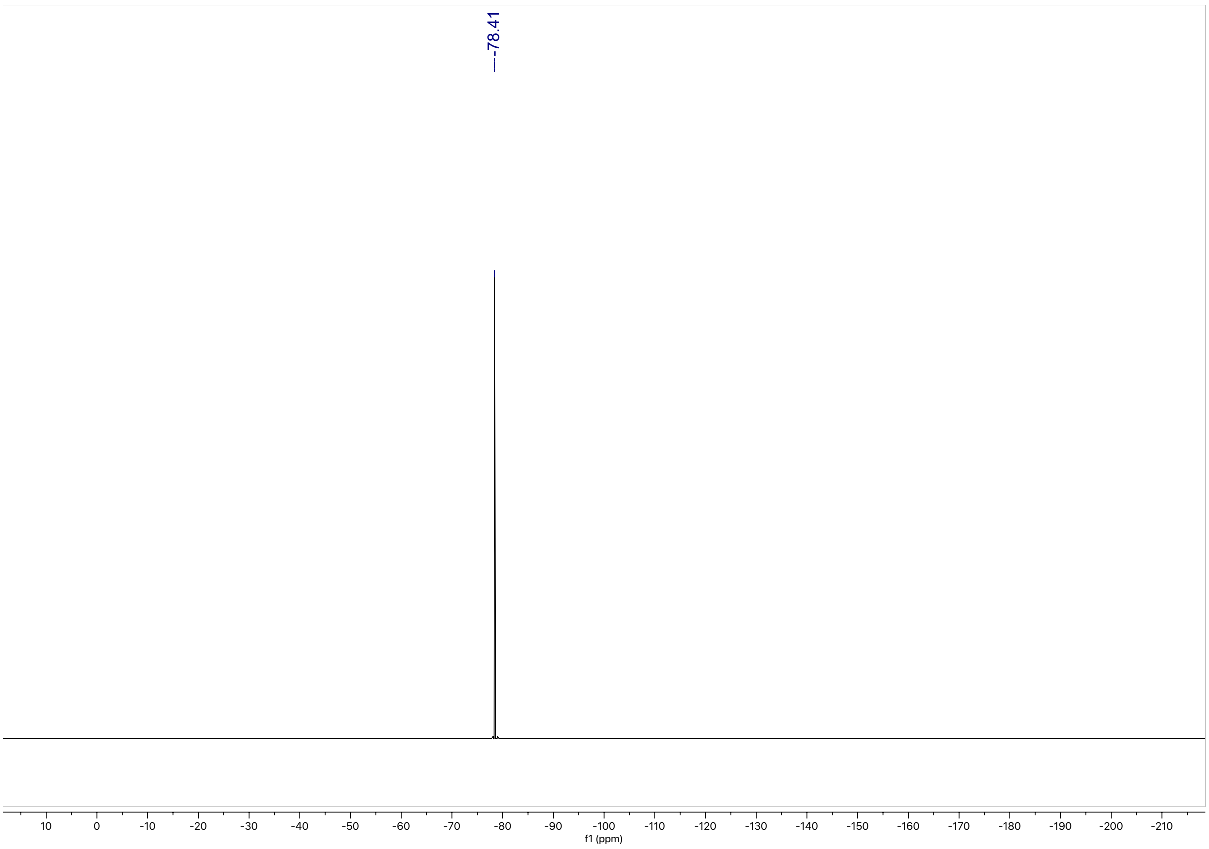

3c – ^{1}H NMR (400 MHz, $CDCl_3$)

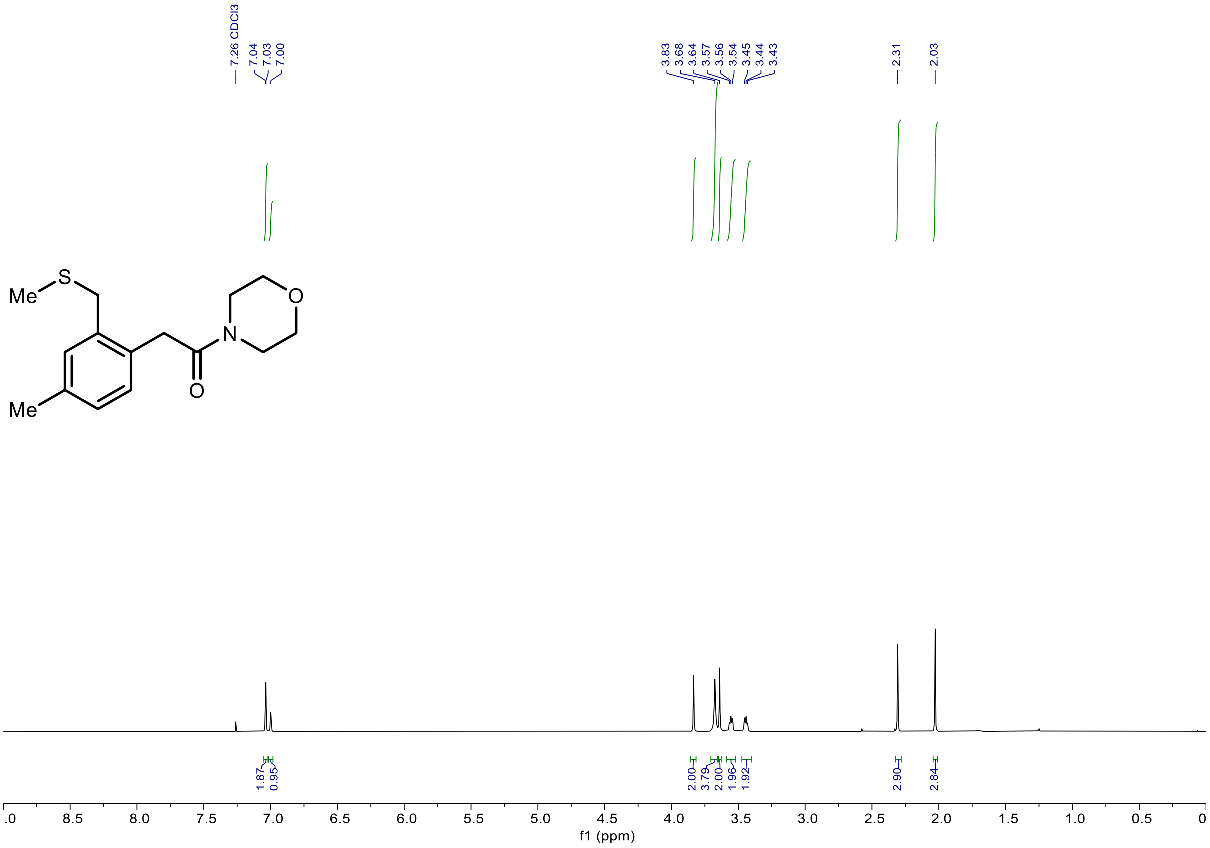

3c – ^{13}C NMR (101 MHz, $CDCl_3$)

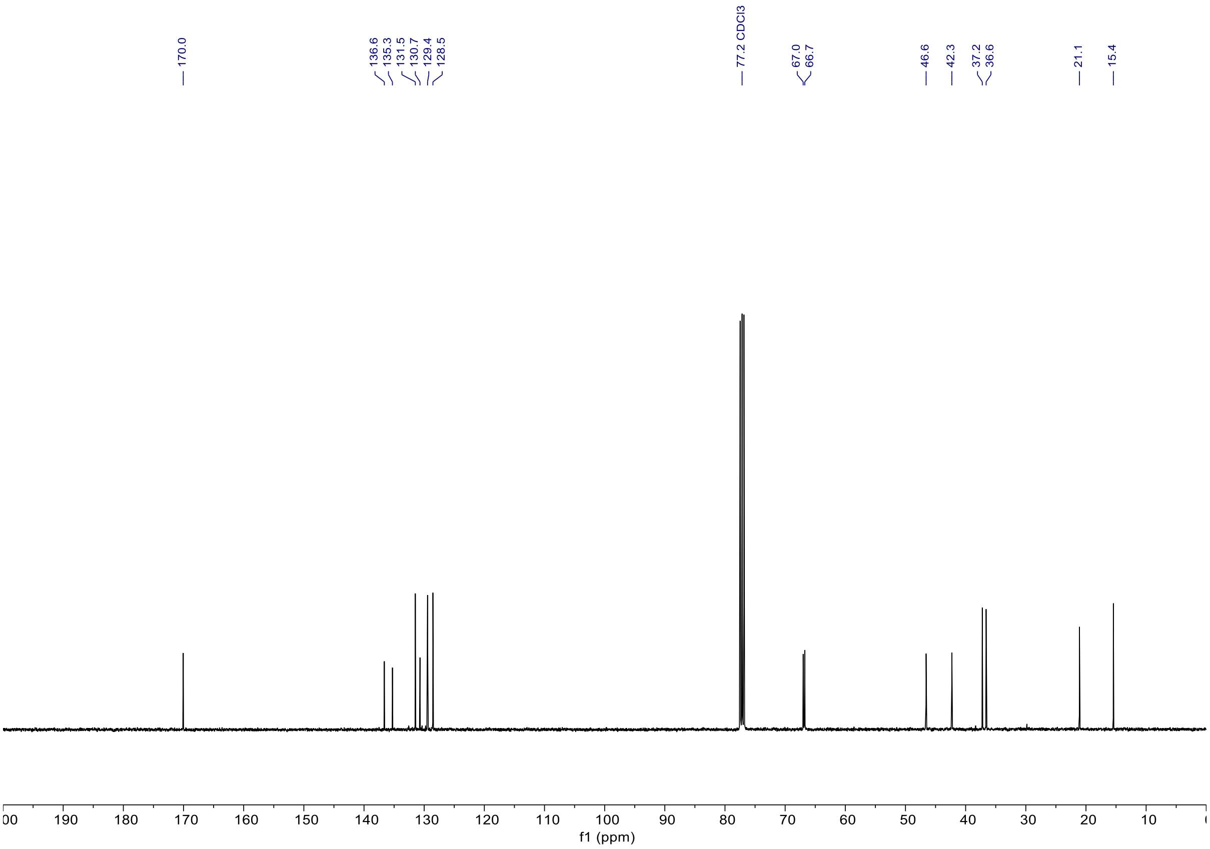

2d – ^{1}H NMR (500 MHz, CD_3CN)

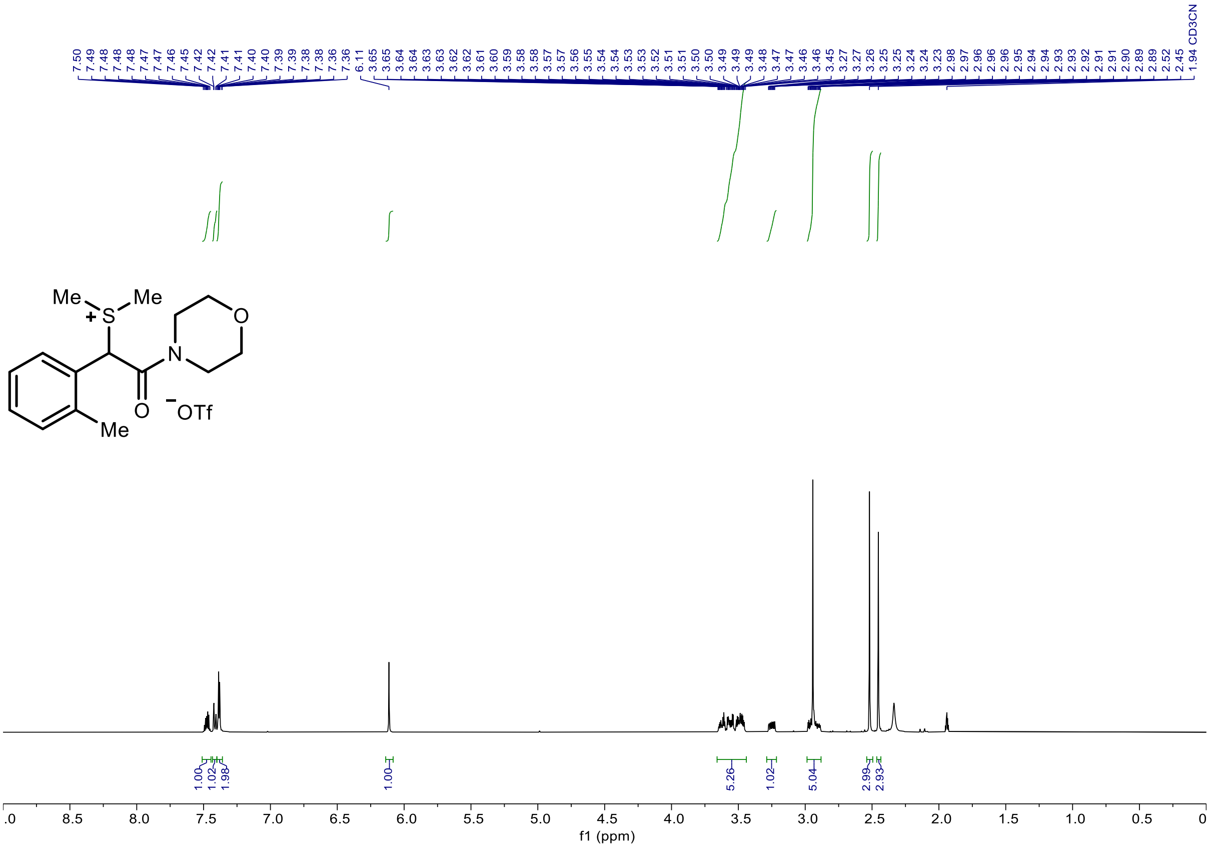

2d – ^{13}C NMR (126 MHz, CD_3CN)

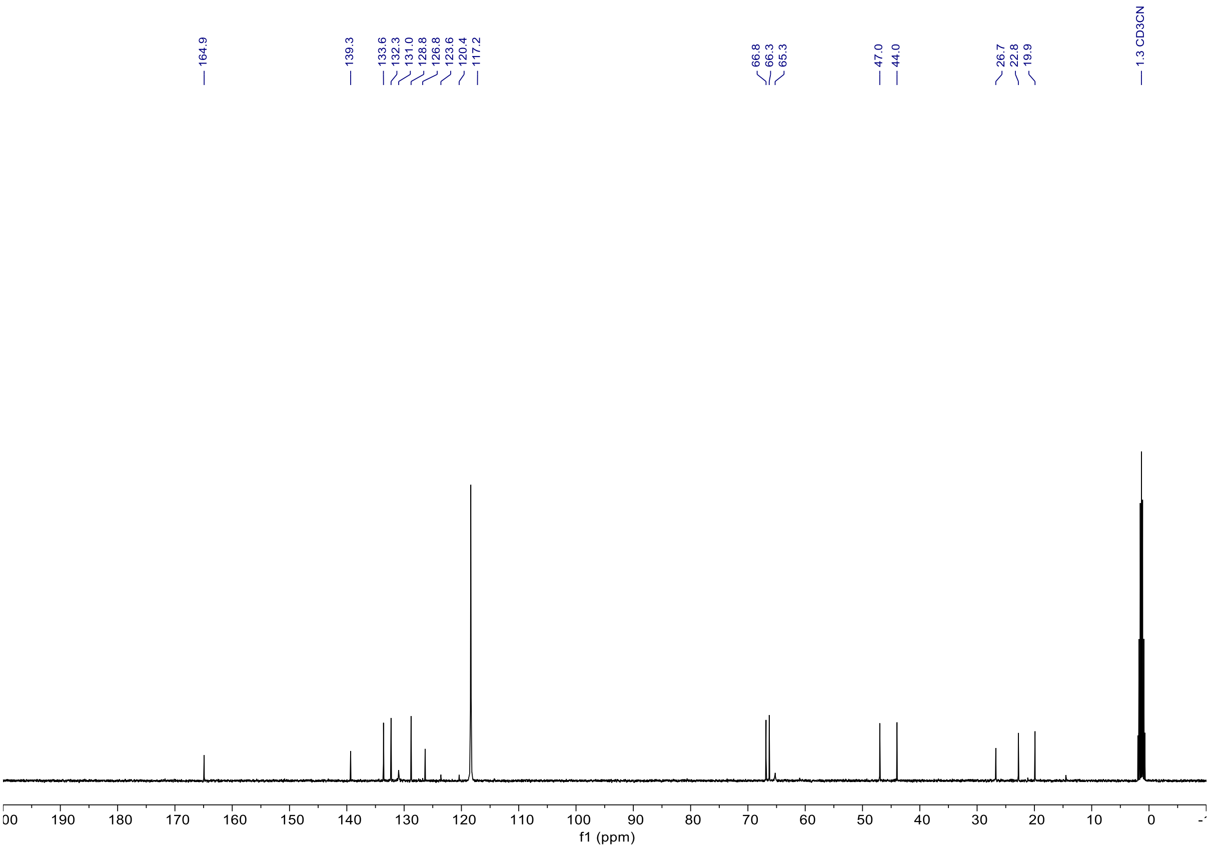

2d – ^{19}F NMR (376 MHz, CD_3CN)

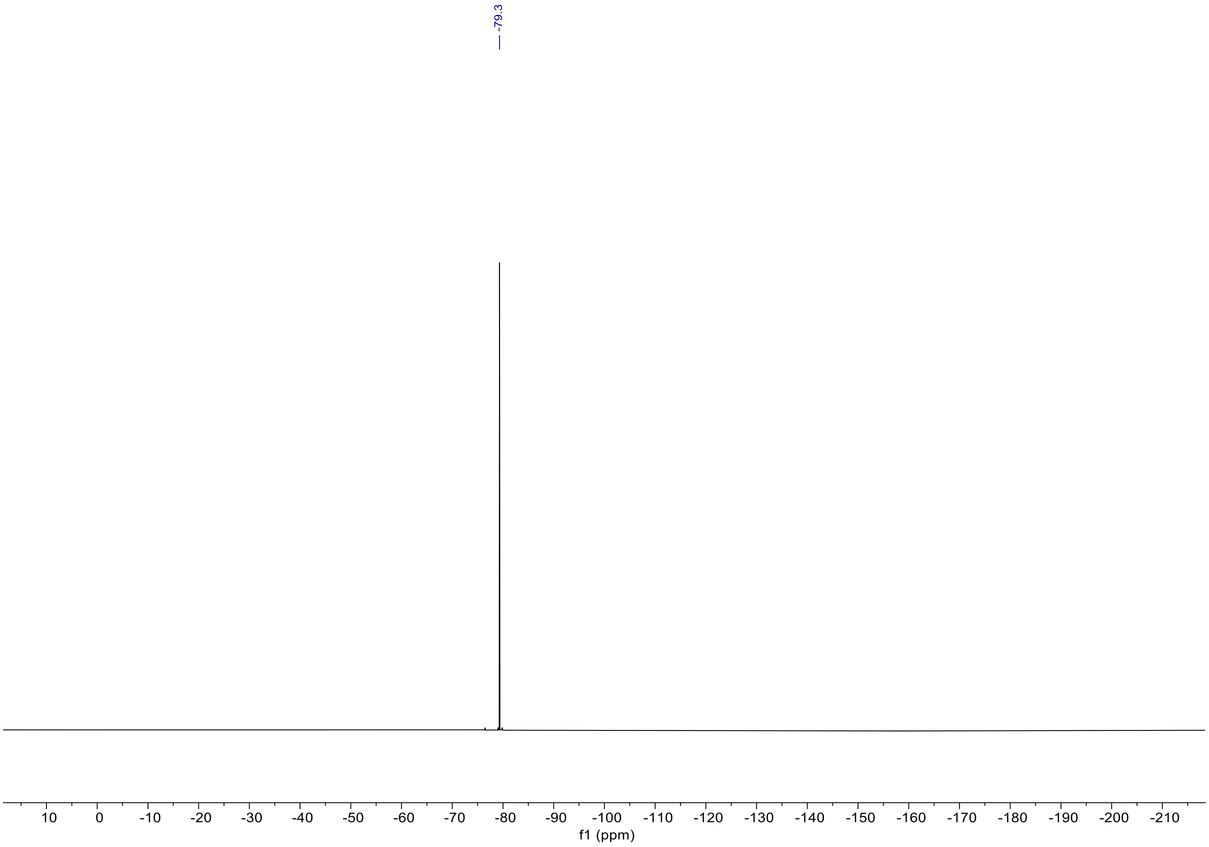

3d – ^{1}H NMR (400 MHz, $CDCl_3$)

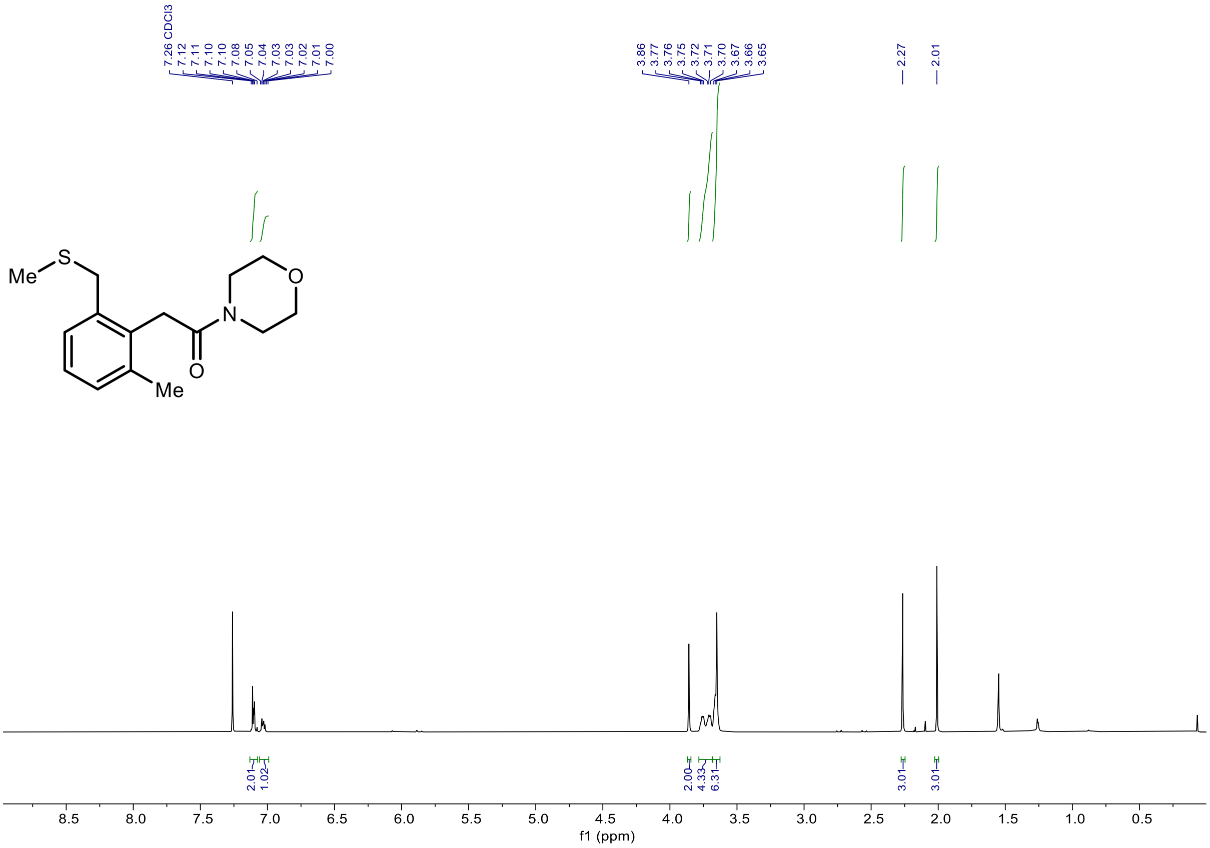

3d – ^{13}C NMR (101 MHz, $CDCl_3$)

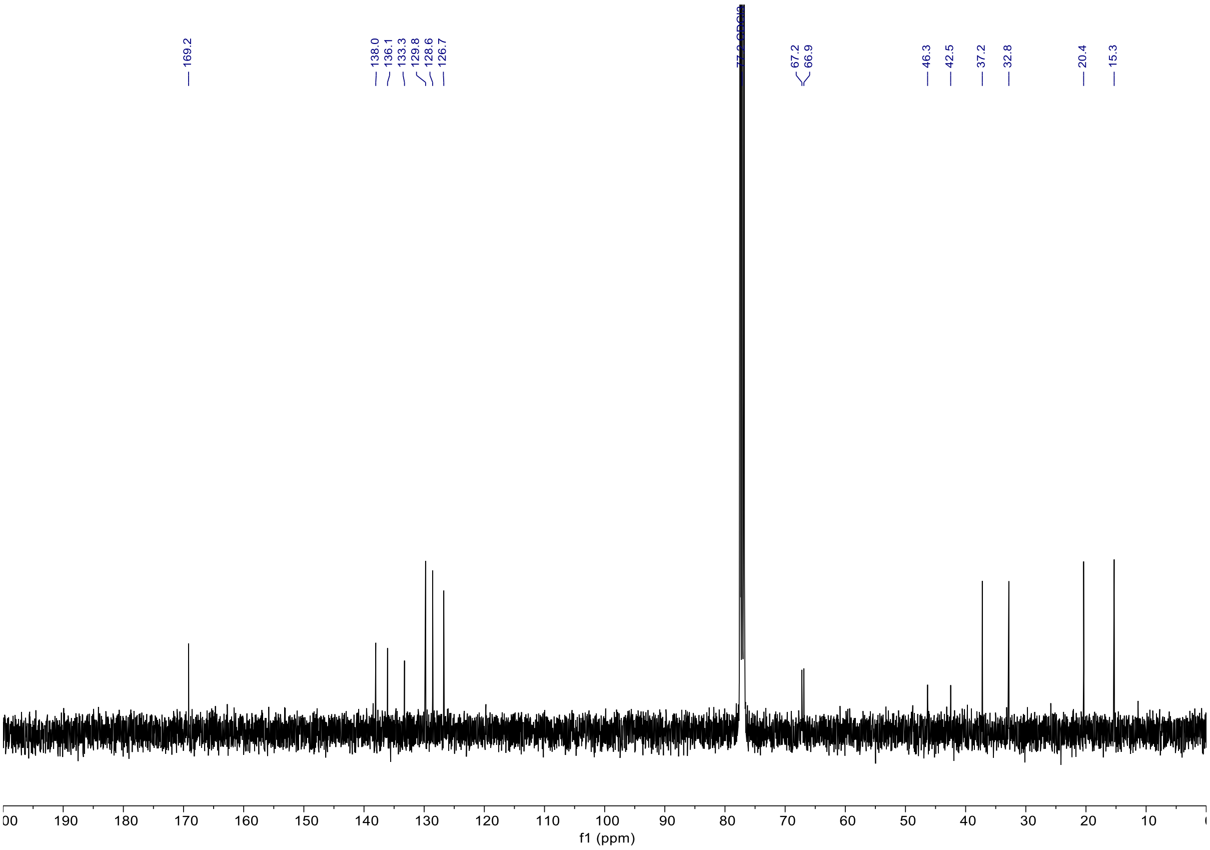

2e – ^{1}H NMR (500 MHz, CD_3CN)

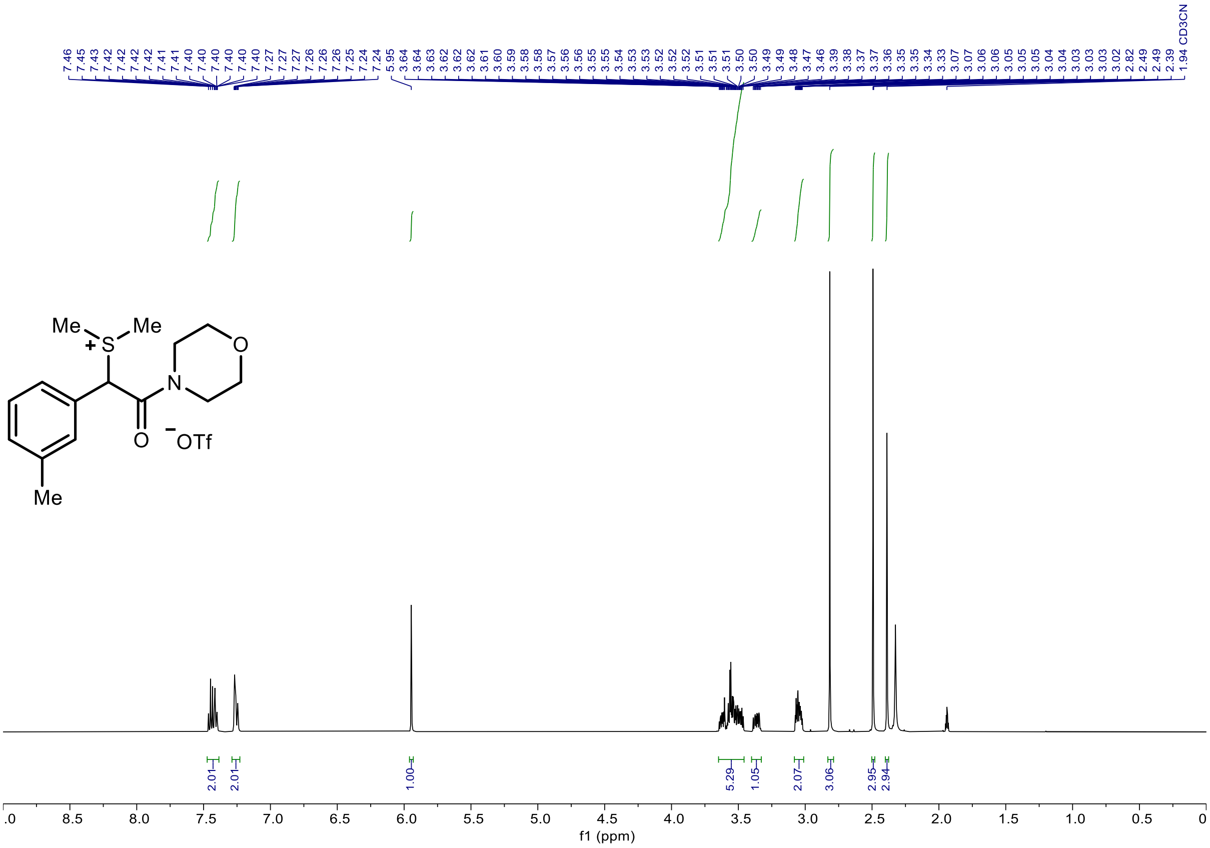

2e – ^{13}C NMR (101 MHz, CD_3CN)

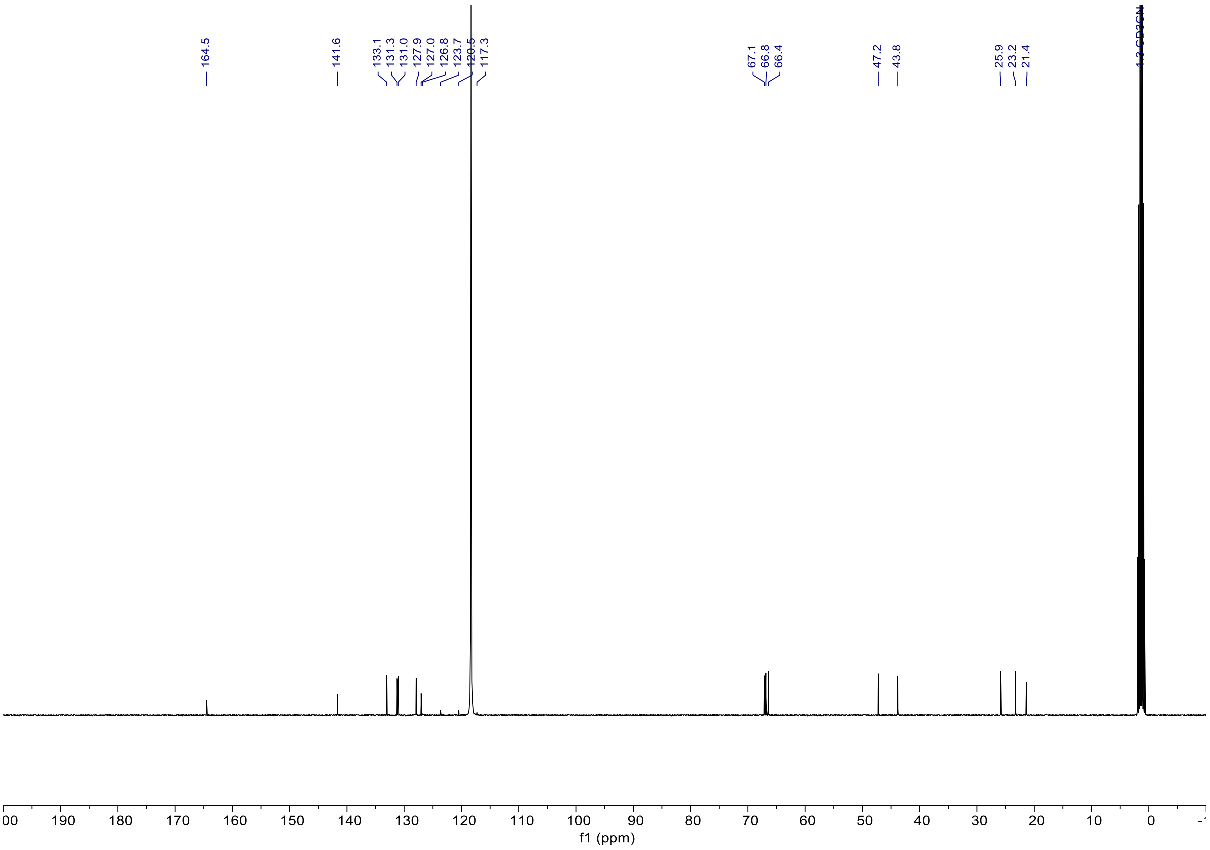

2e – ^{19}F NMR (376 MHz, CD_3CN)

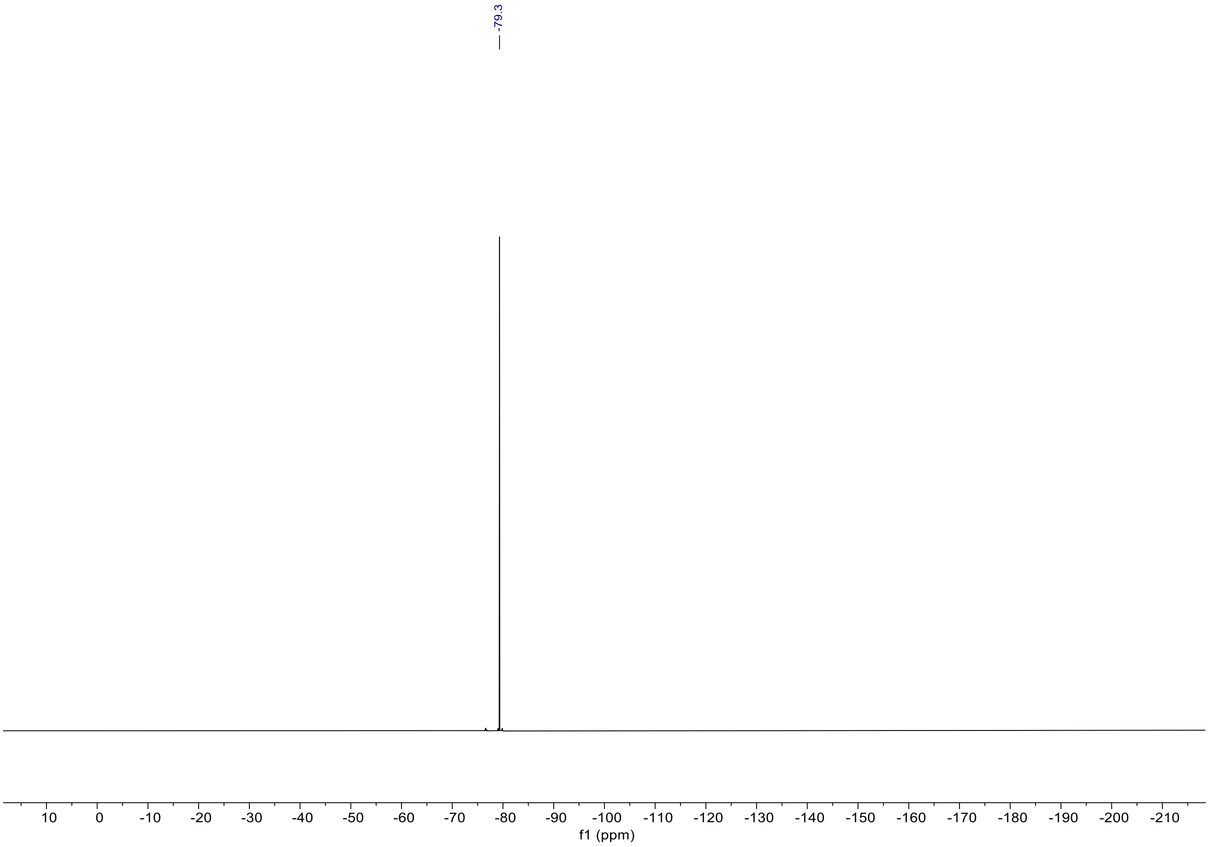

3e – ^{1}H NMR (500 MHz, $CDCl_3$)

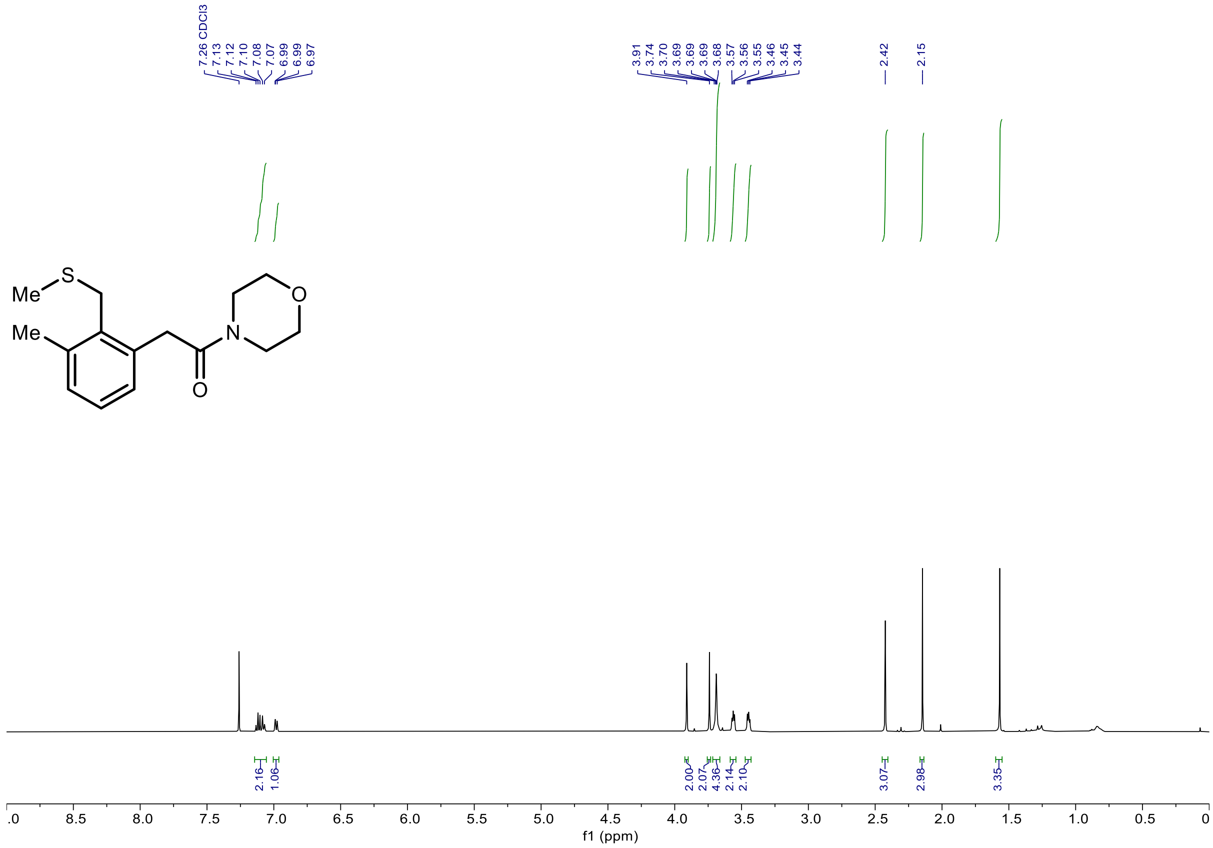

3e – ^{13}C NMR (126 MHz, $CDCl_3$)

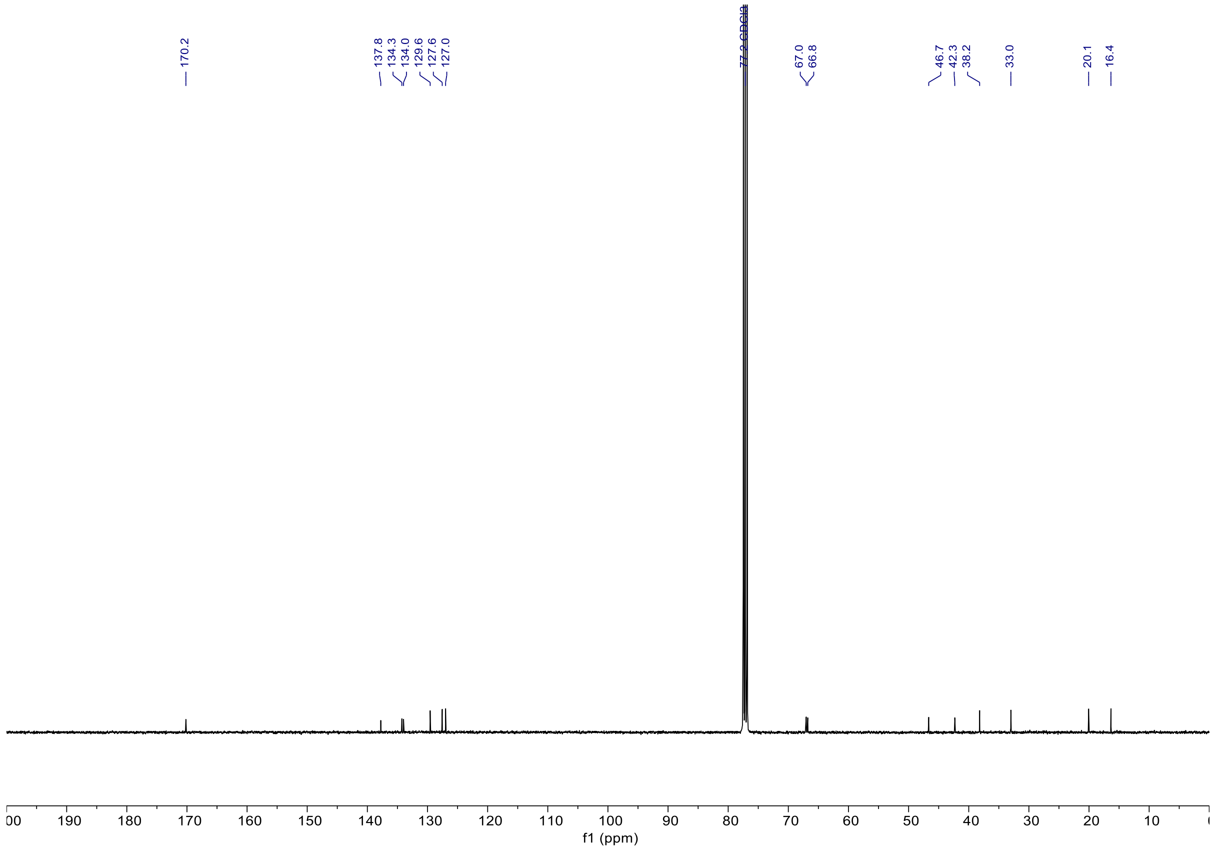

3e' – ^{1}H NMR (500 MHz, $CDCl_3$)

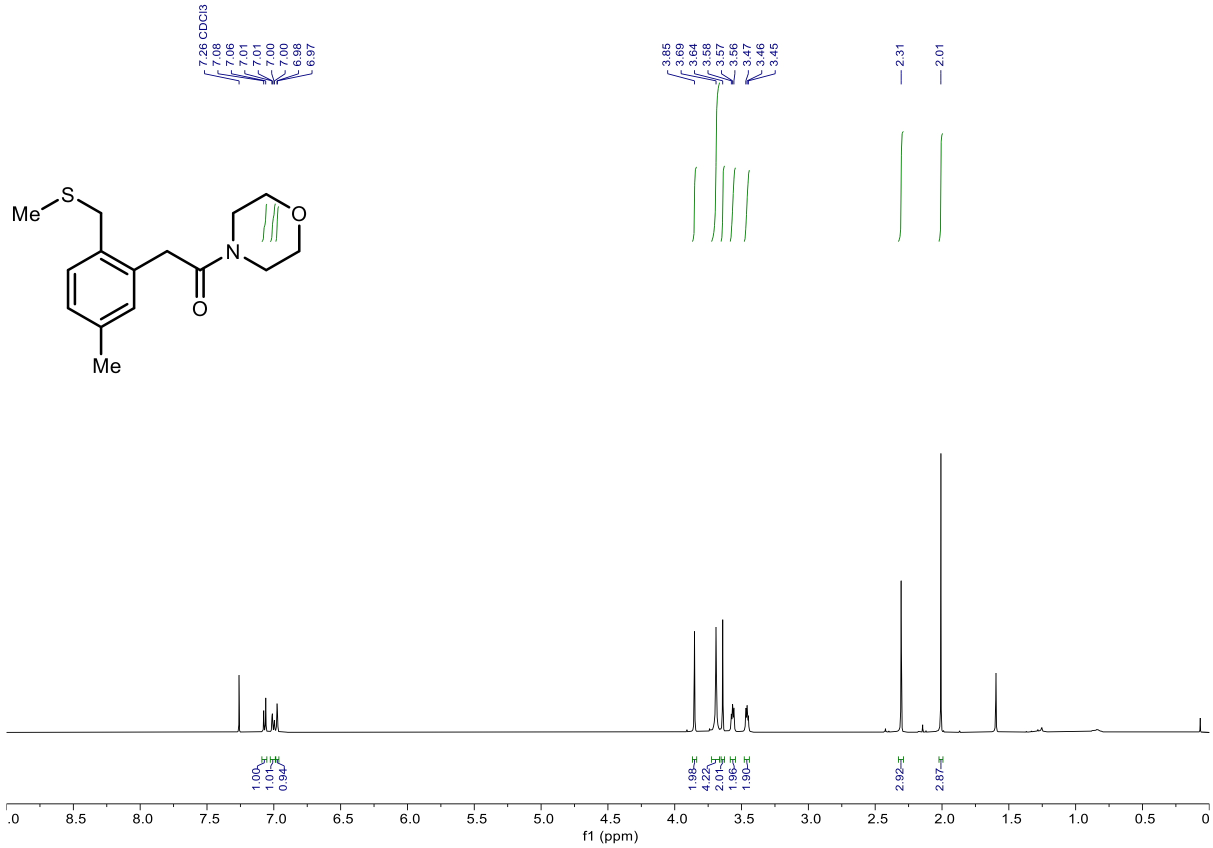

3e' – ^{13}C NMR (126 MHz, $CDCl_3$)

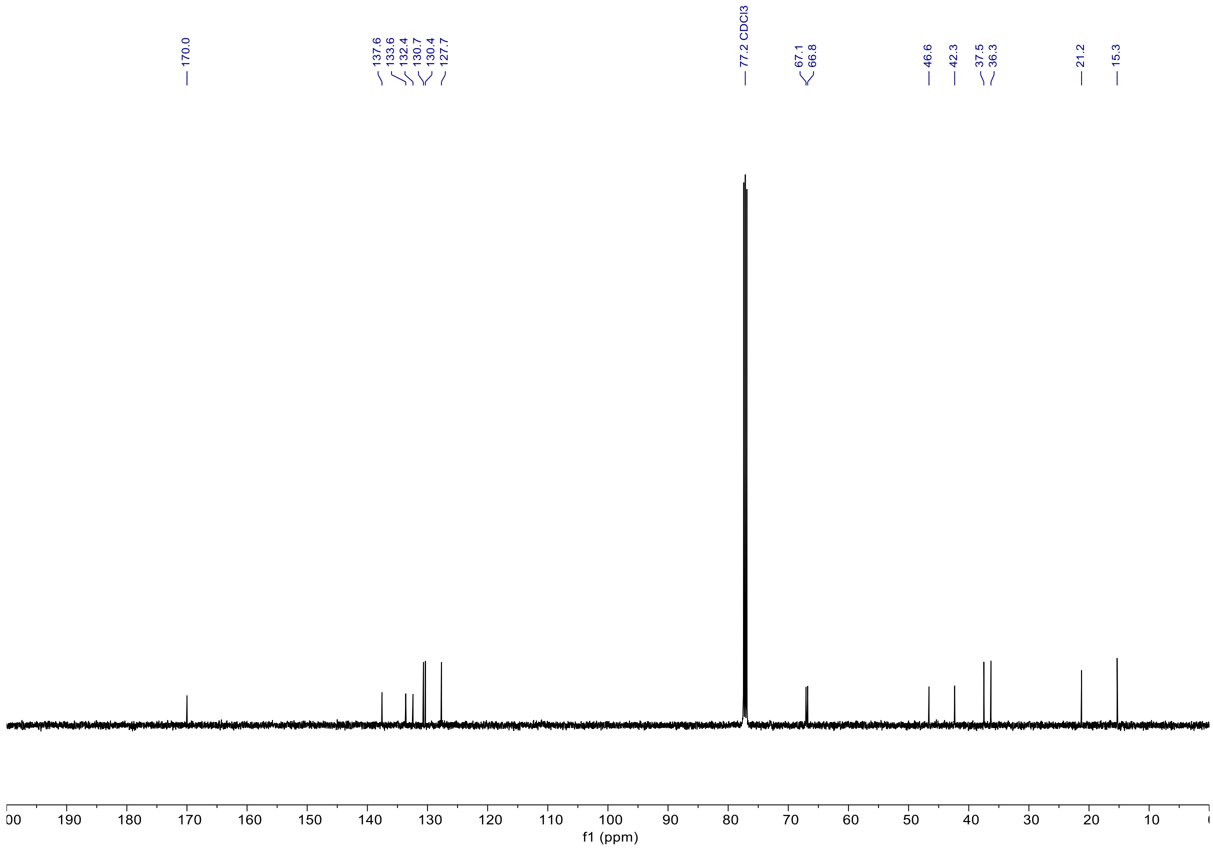

2f – ^{1}H NMR (400 MHz, CD_3CN)

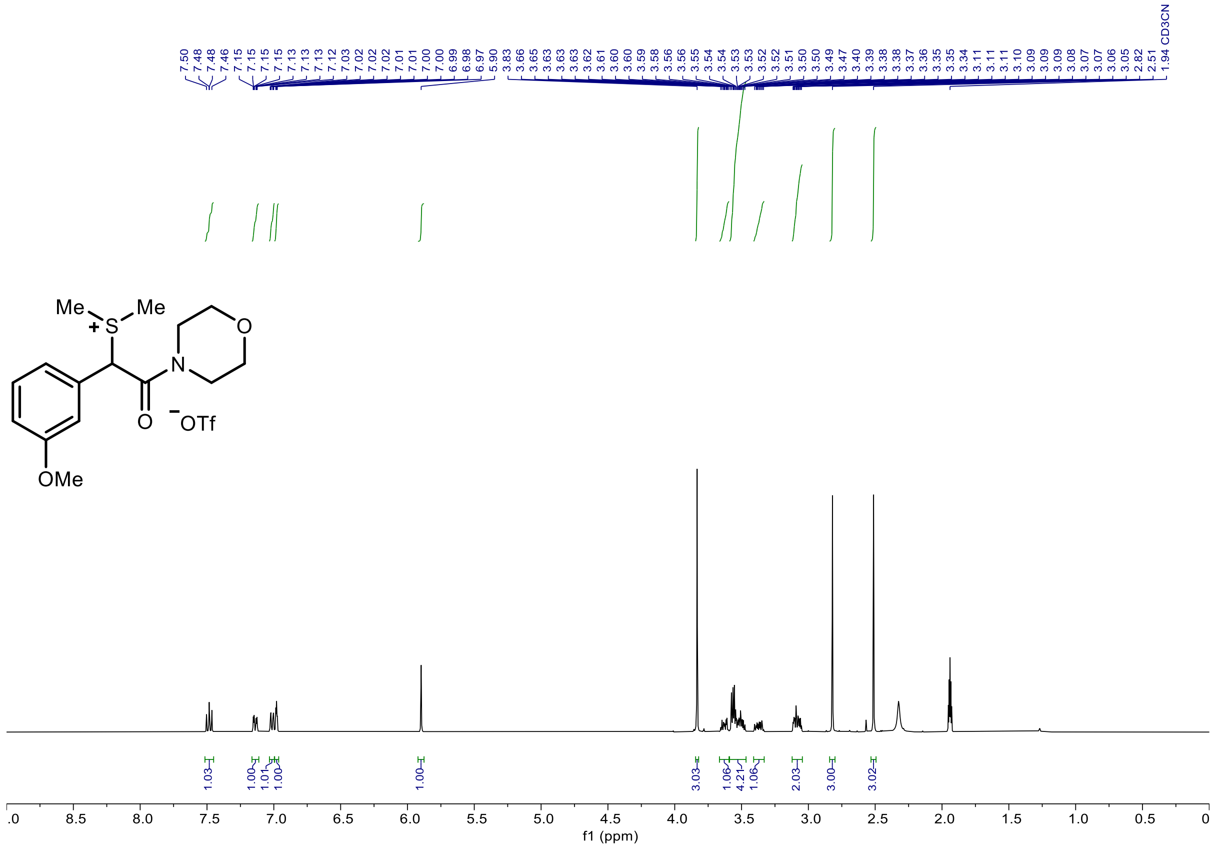

2f – ^{13}C NMR (101 MHz, CD_3CN)

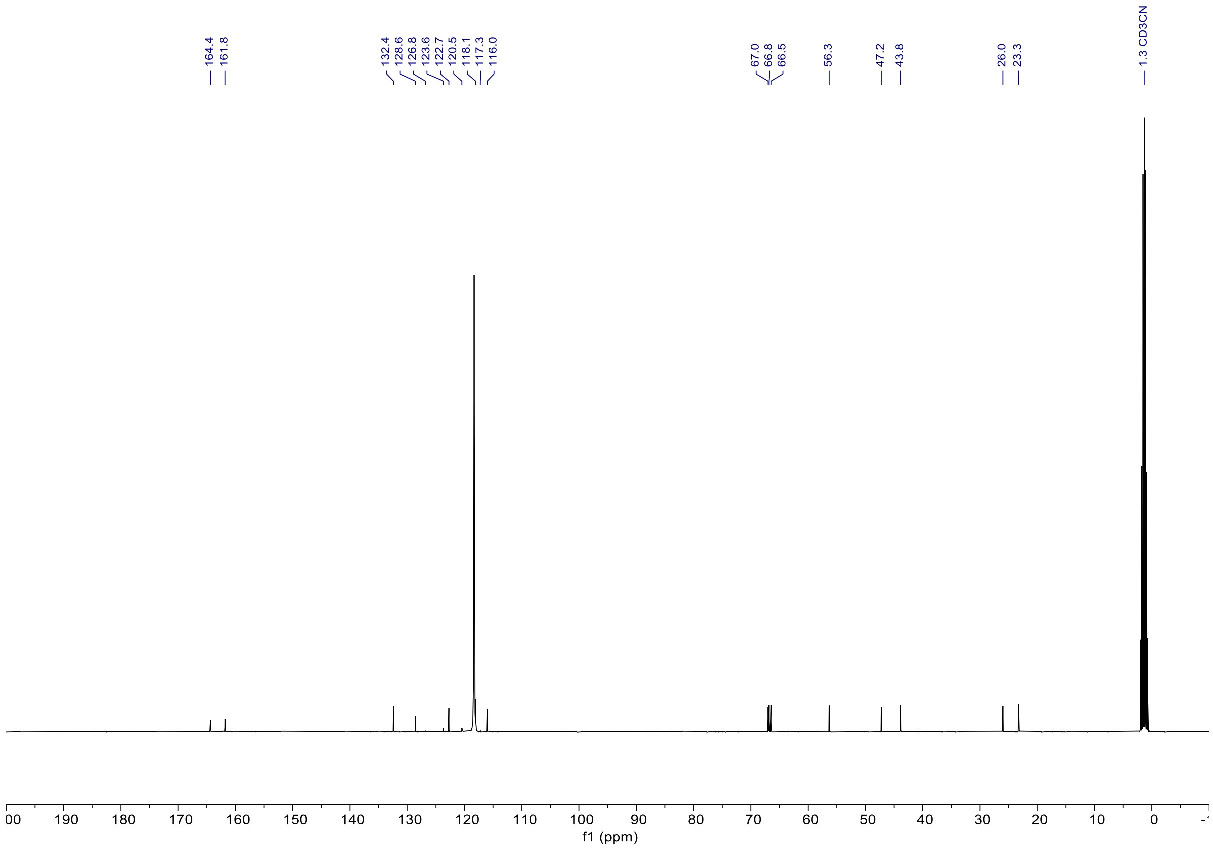

2f – ^{19}F NMR (376 MHz, CD_3CN)

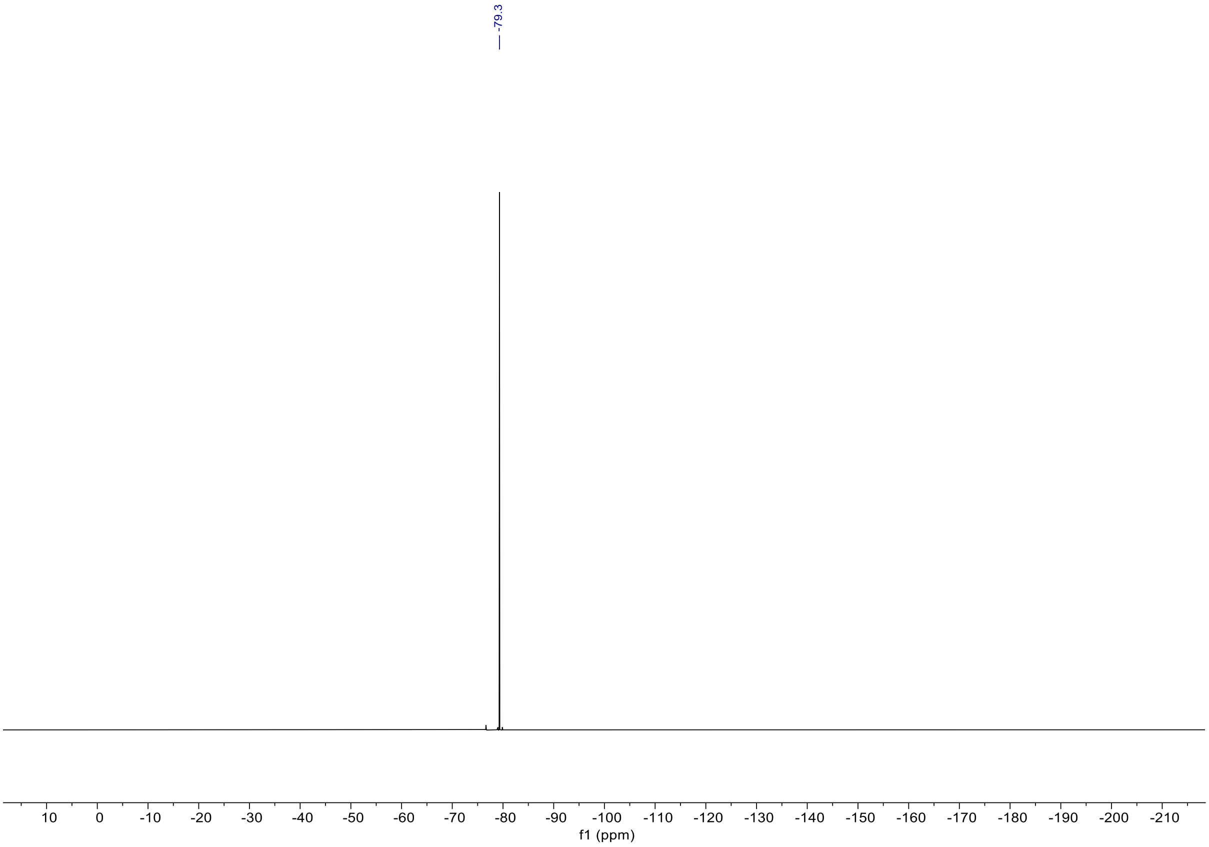

3f – ^{1}H NMR (400 MHz, $CDCl_3$)

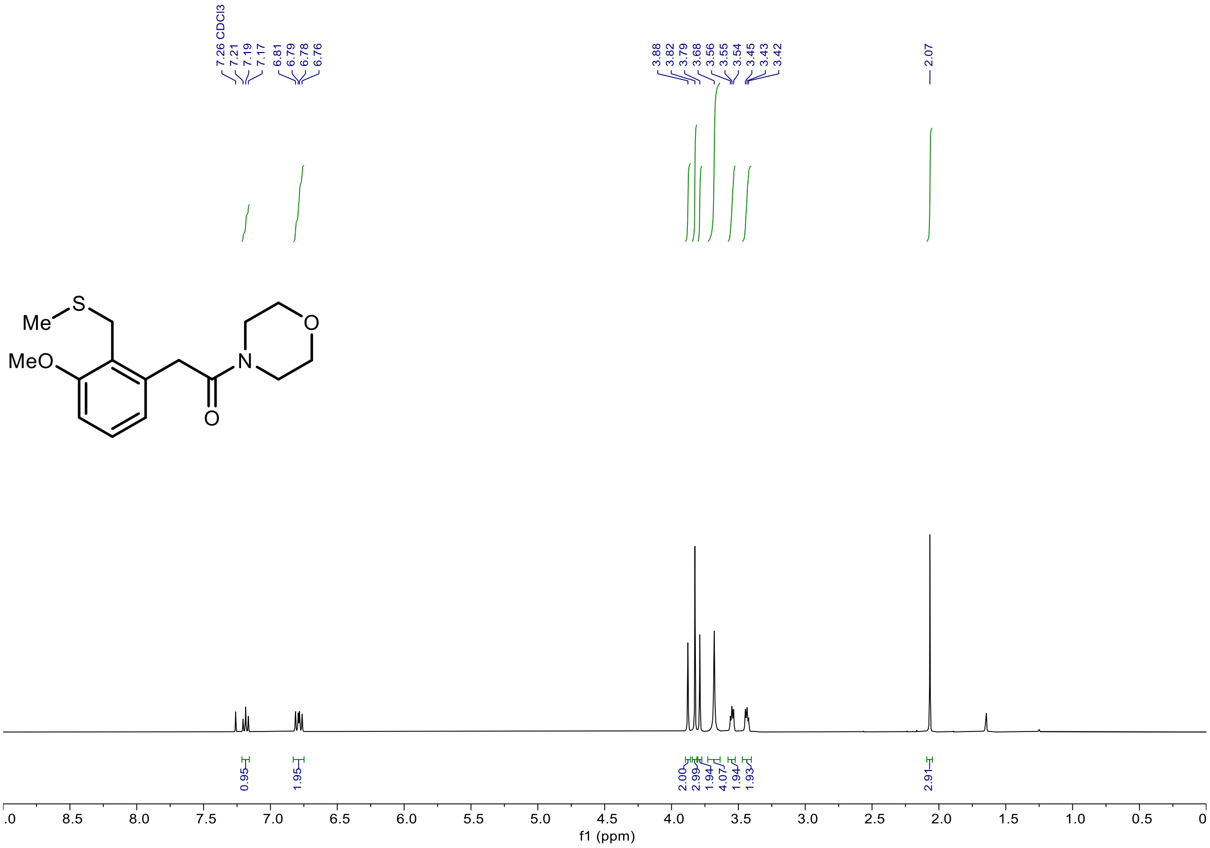

3f – ^{13}C NMR (101 MHz, $CDCl_3$)

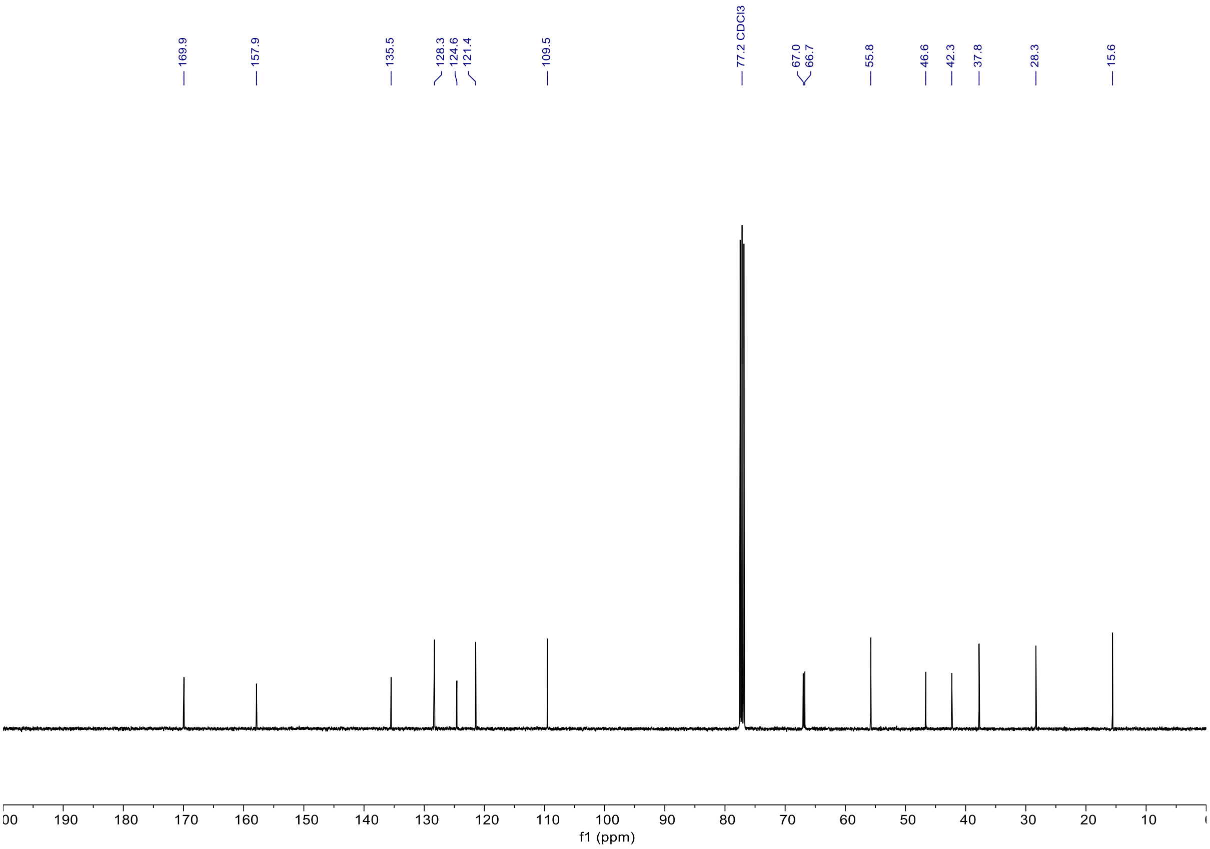

3f' – ^{1}H NMR (400 MHz, $CDCl_3$)

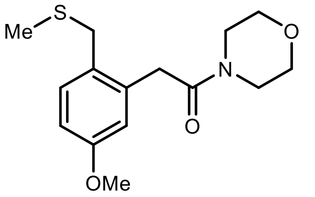

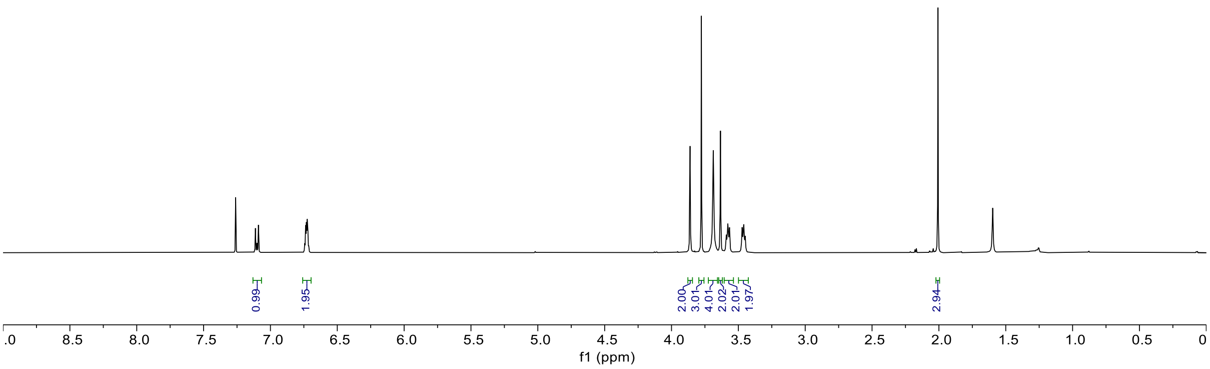

3f' – ^{13}C NMR (101 MHz, $CDCl_3$)

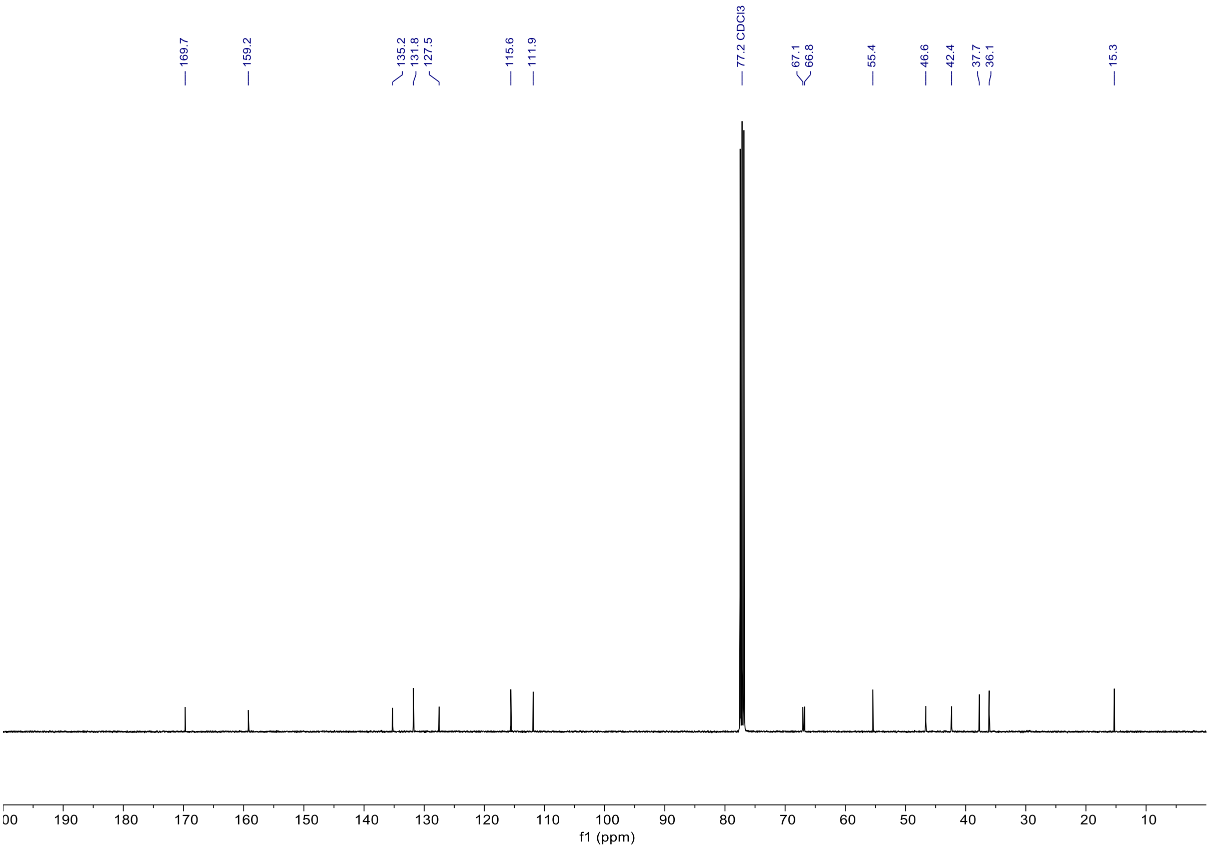

2g – ^{1}H NMR (500 MHz, CD_3CN)

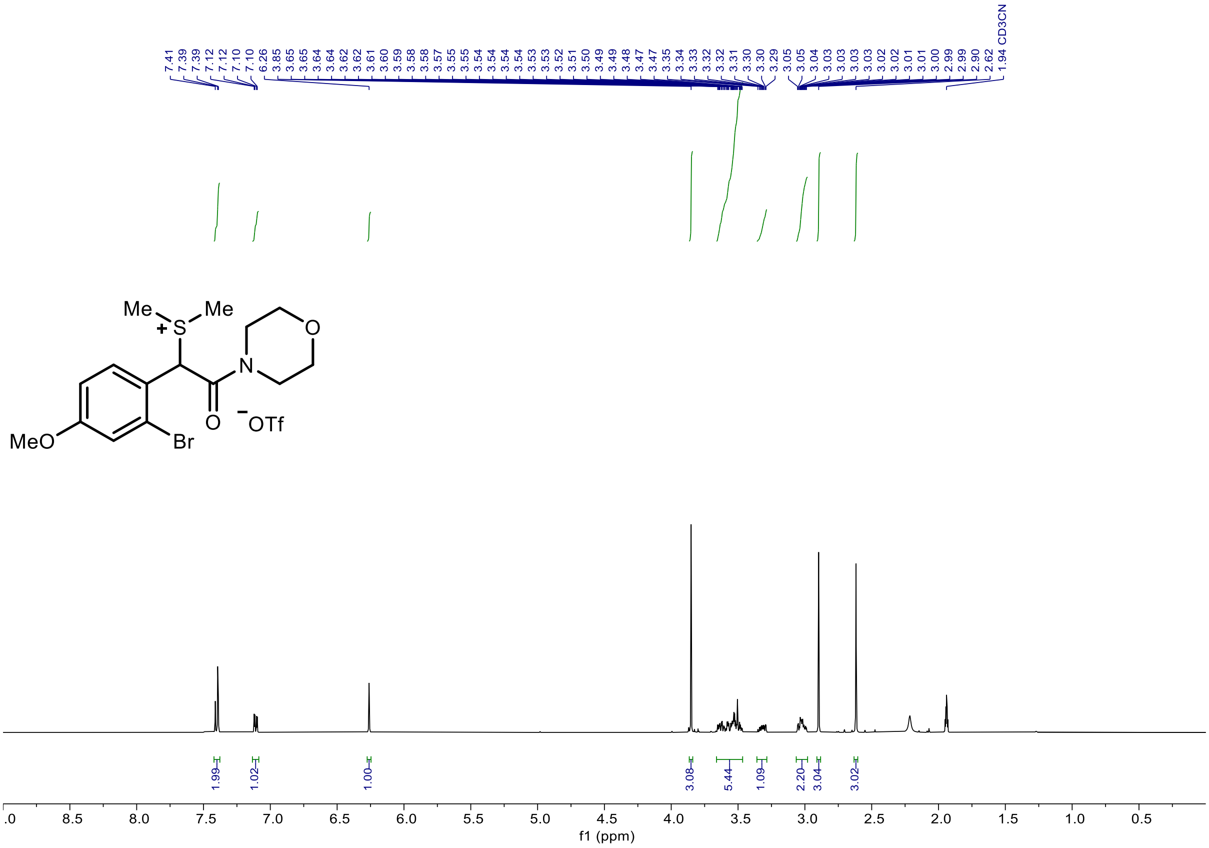

2g – ^{13}C NMR (126 MHz, CD_3CN)

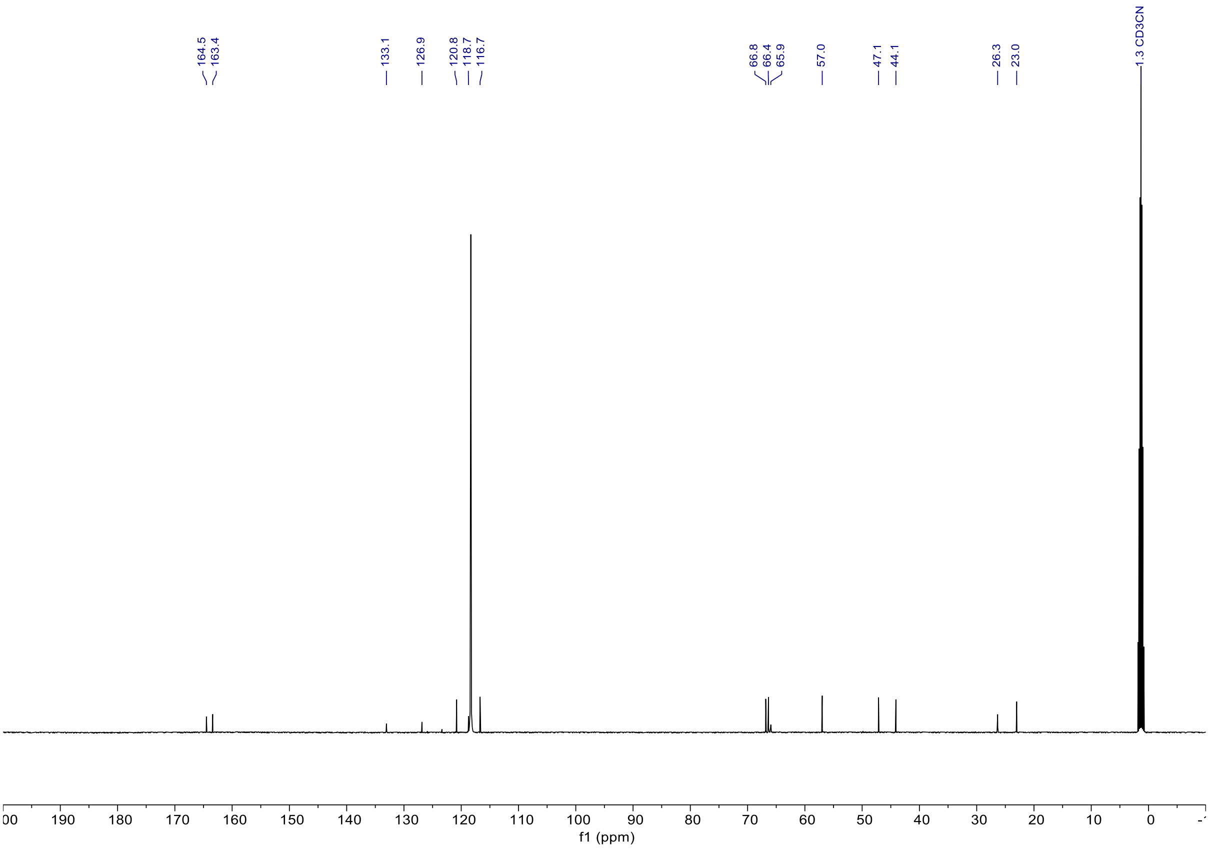

2g – ^{19}F NMR (376 MHz, CD_3CN)

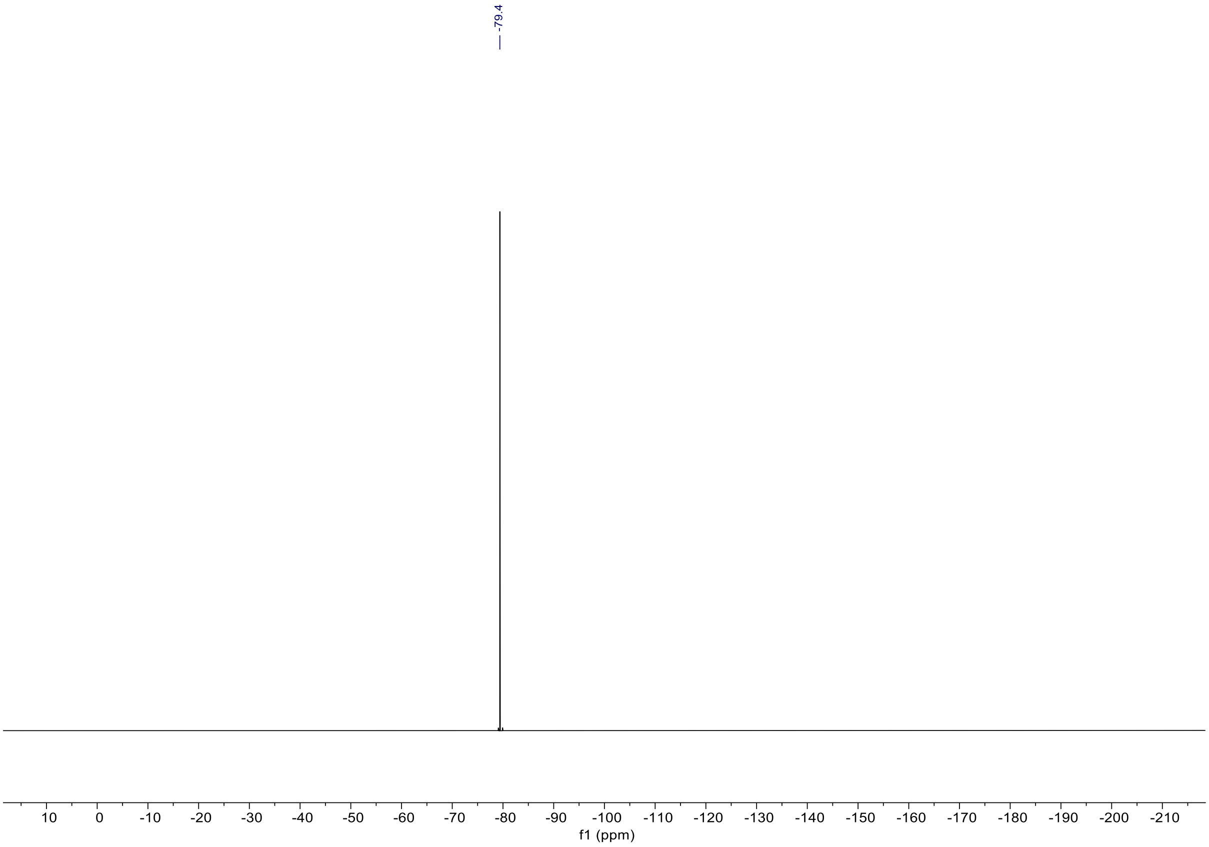

3g – ^{1}H NMR (500 MHz, $CDCl_3$)

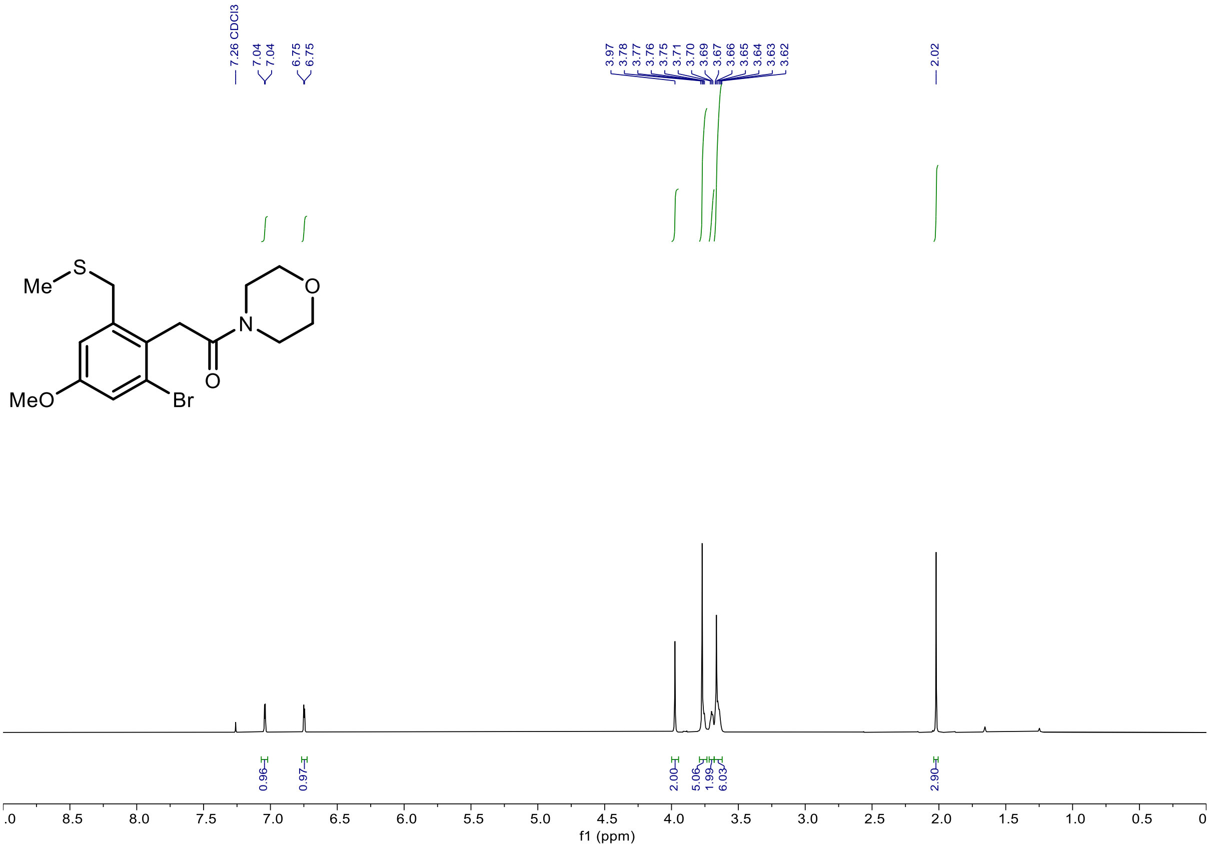

3g – ^{13}C NMR (126 MHz, $CDCl_3$)

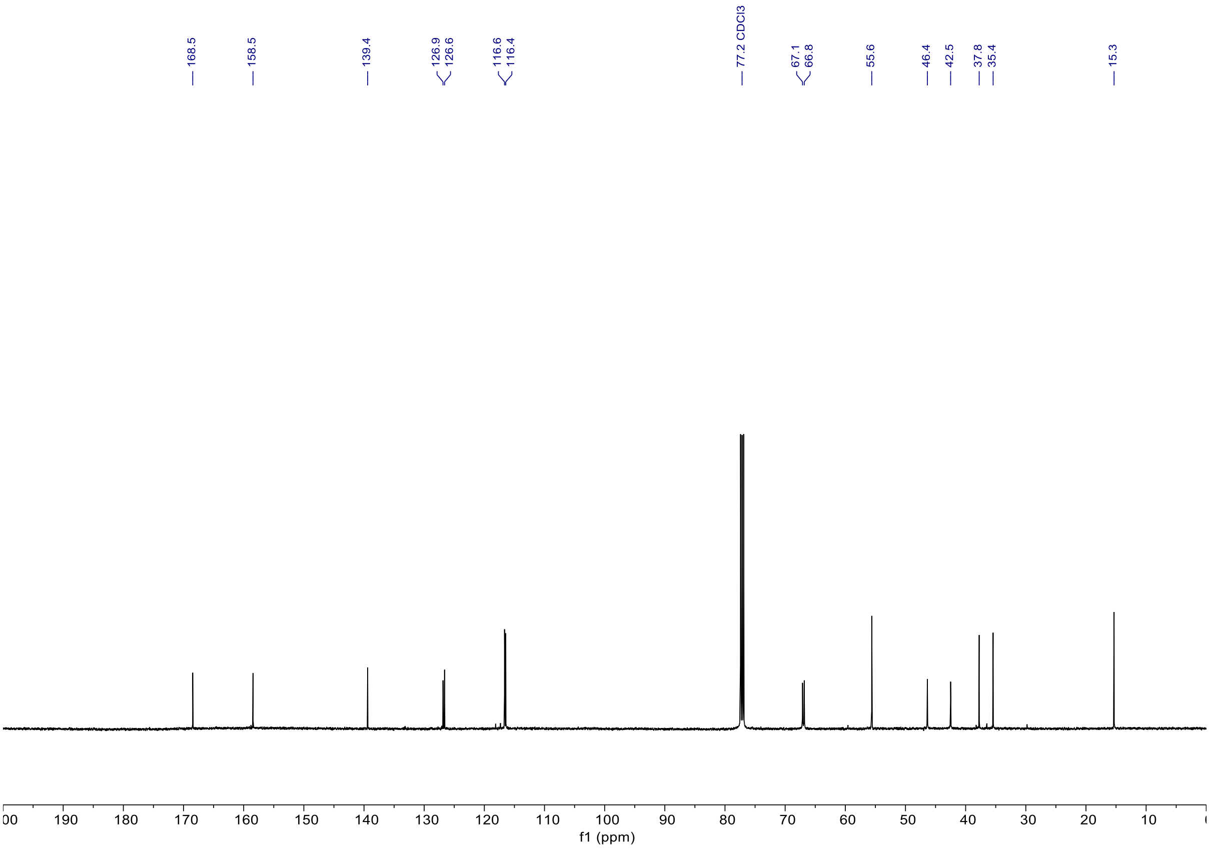

2h – ^{1}H NMR (400 MHz, $CDCl_3$)

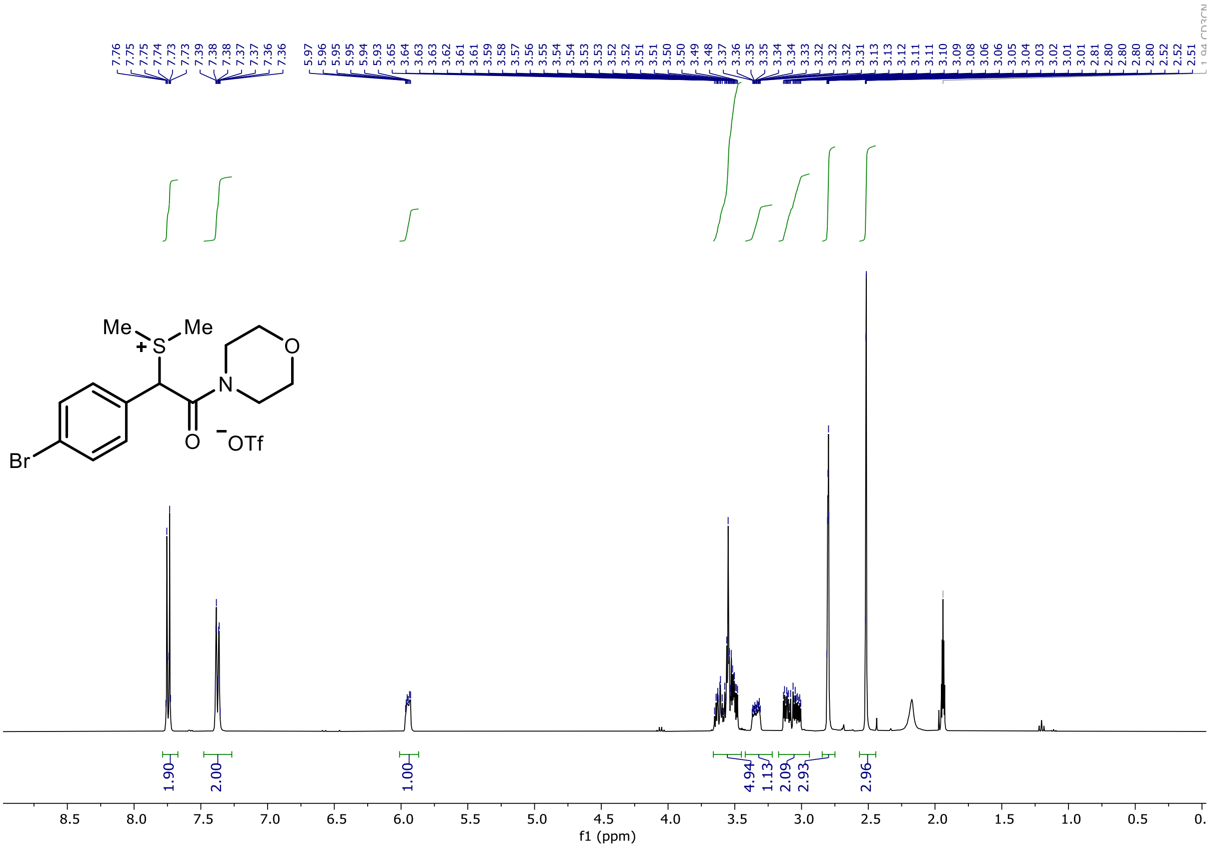

2h – ^{13}C NMR (101 MHz, $CDCl_3$)

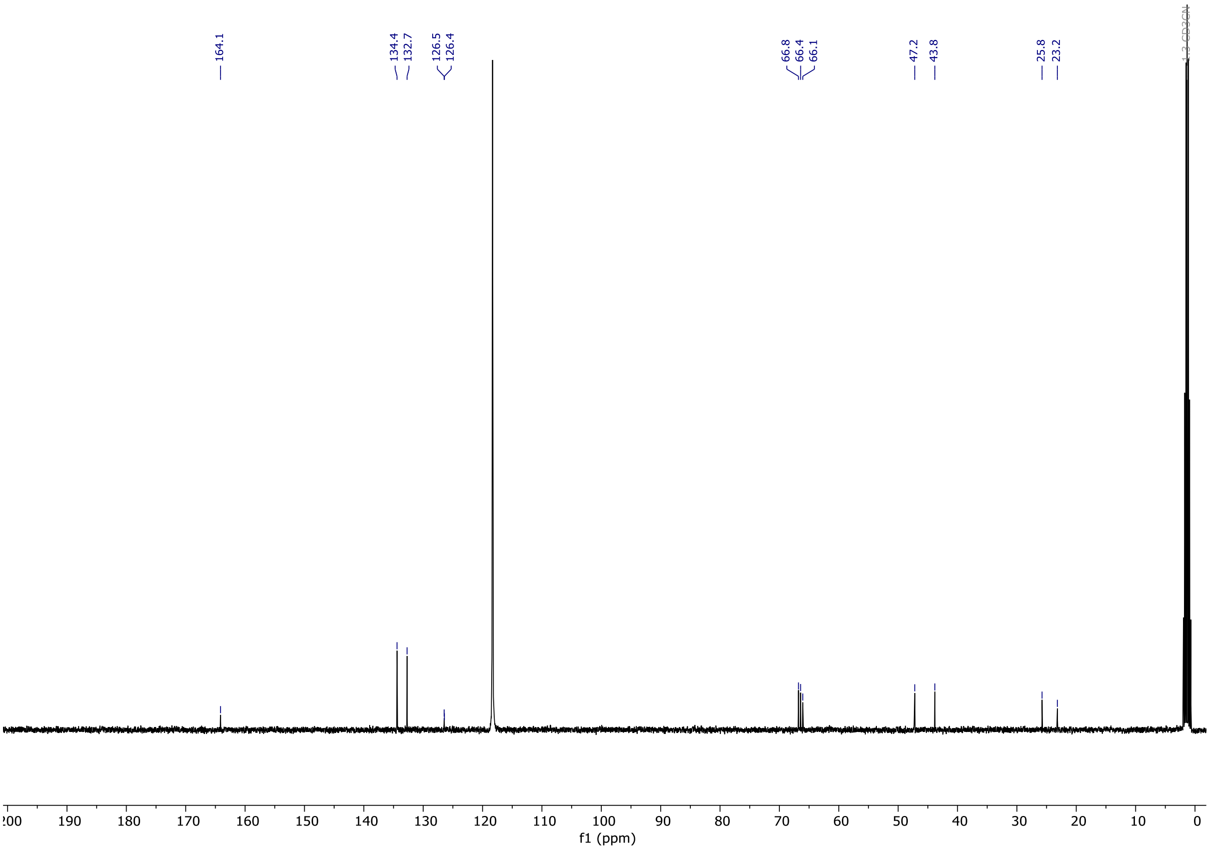

2h – ^{19}F NMR (376 MHz, $CDCl_3$)

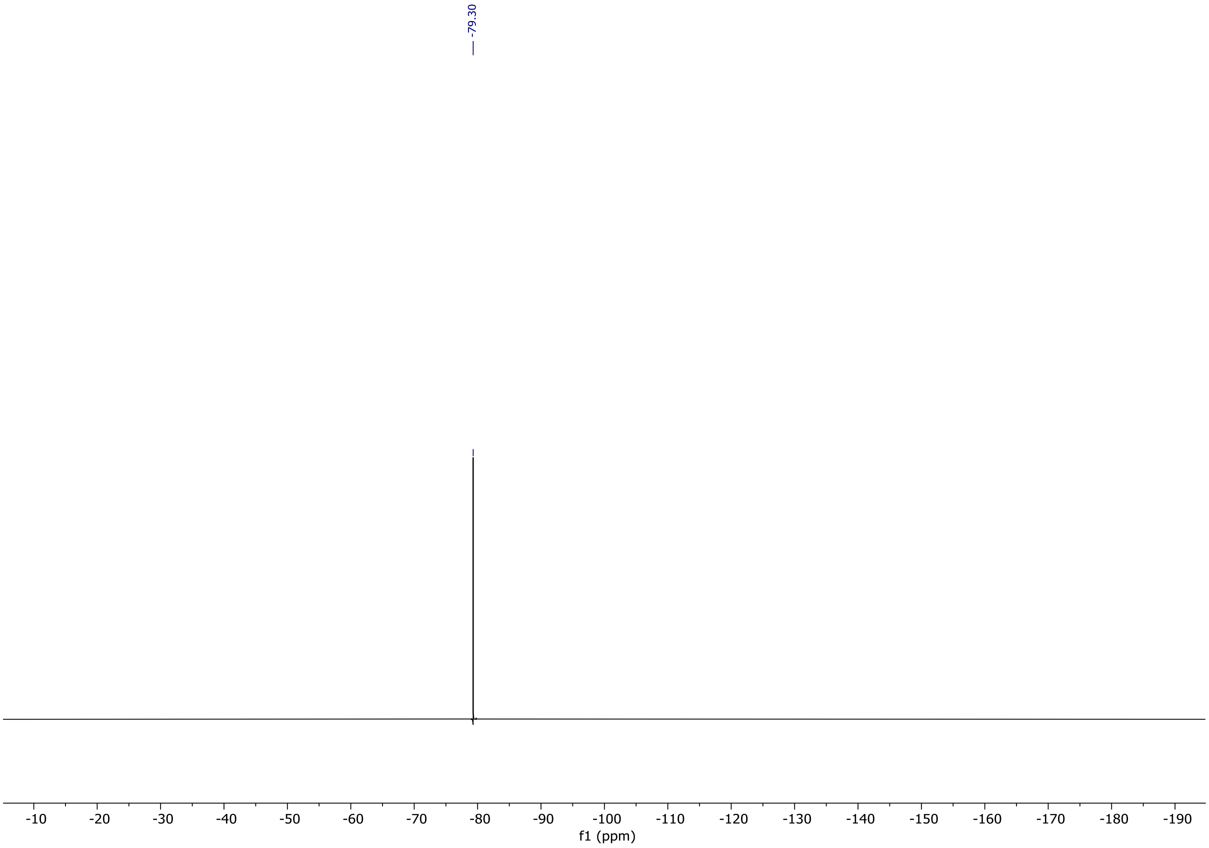

3h – ^{1}H NMR (500 MHz, $CDCl_3$)

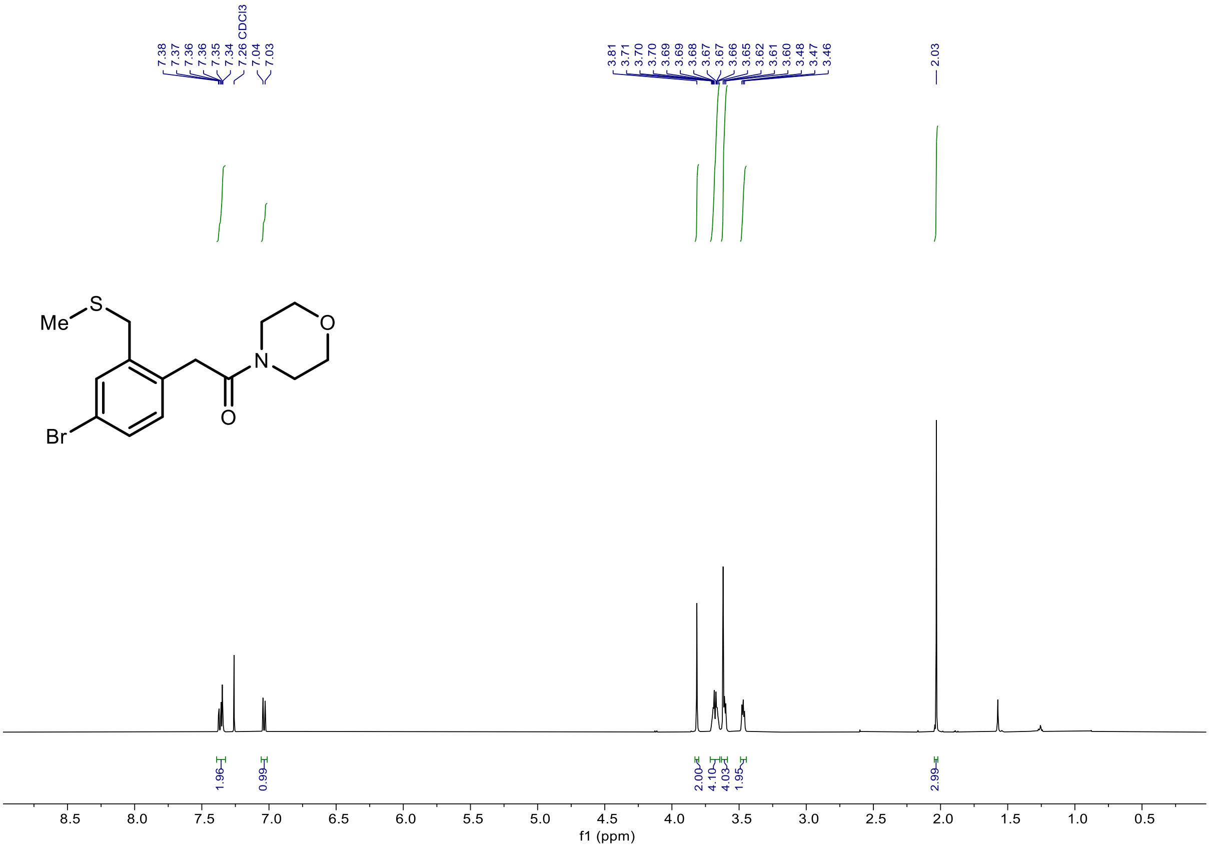

3h – ^{13}C NMR (126 MHz, $CDCl_3$)

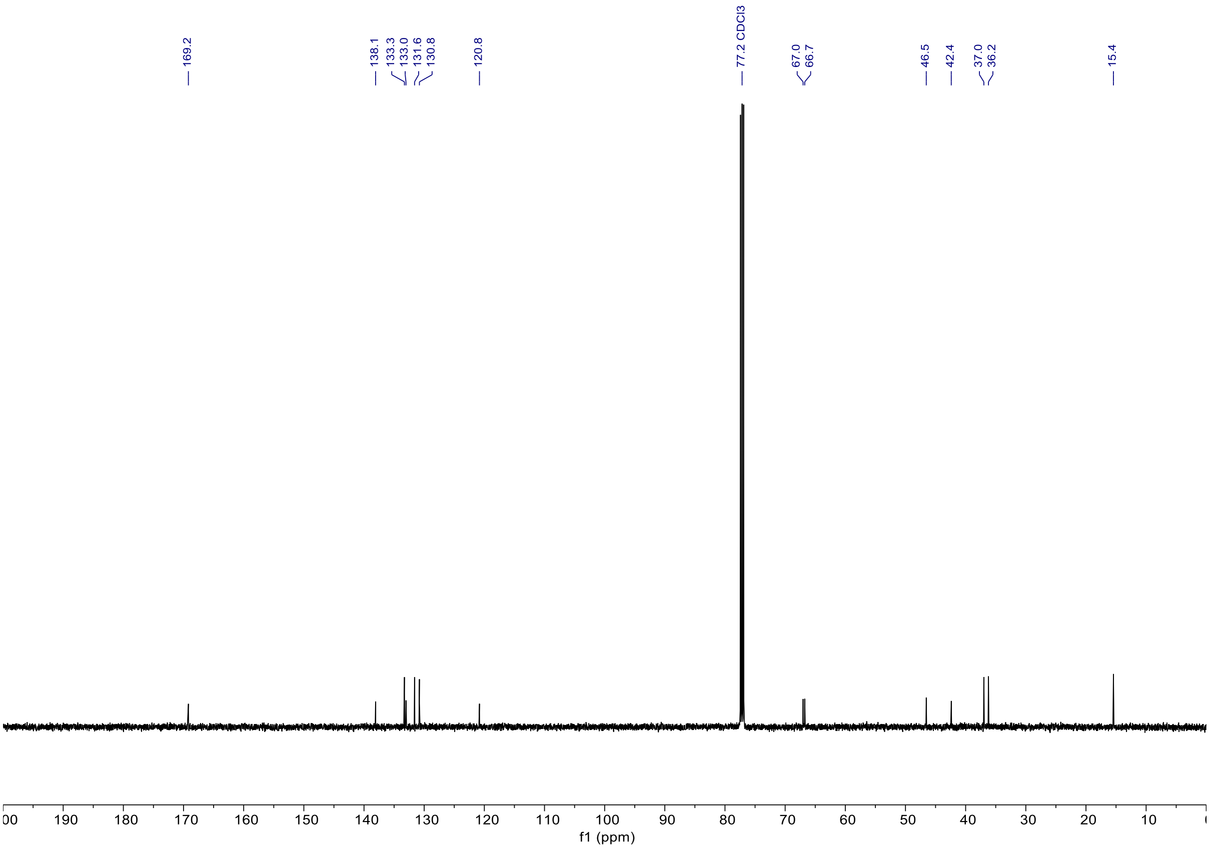

2i – ^{1}H NMR (400 MHz, $CDCl_3$)

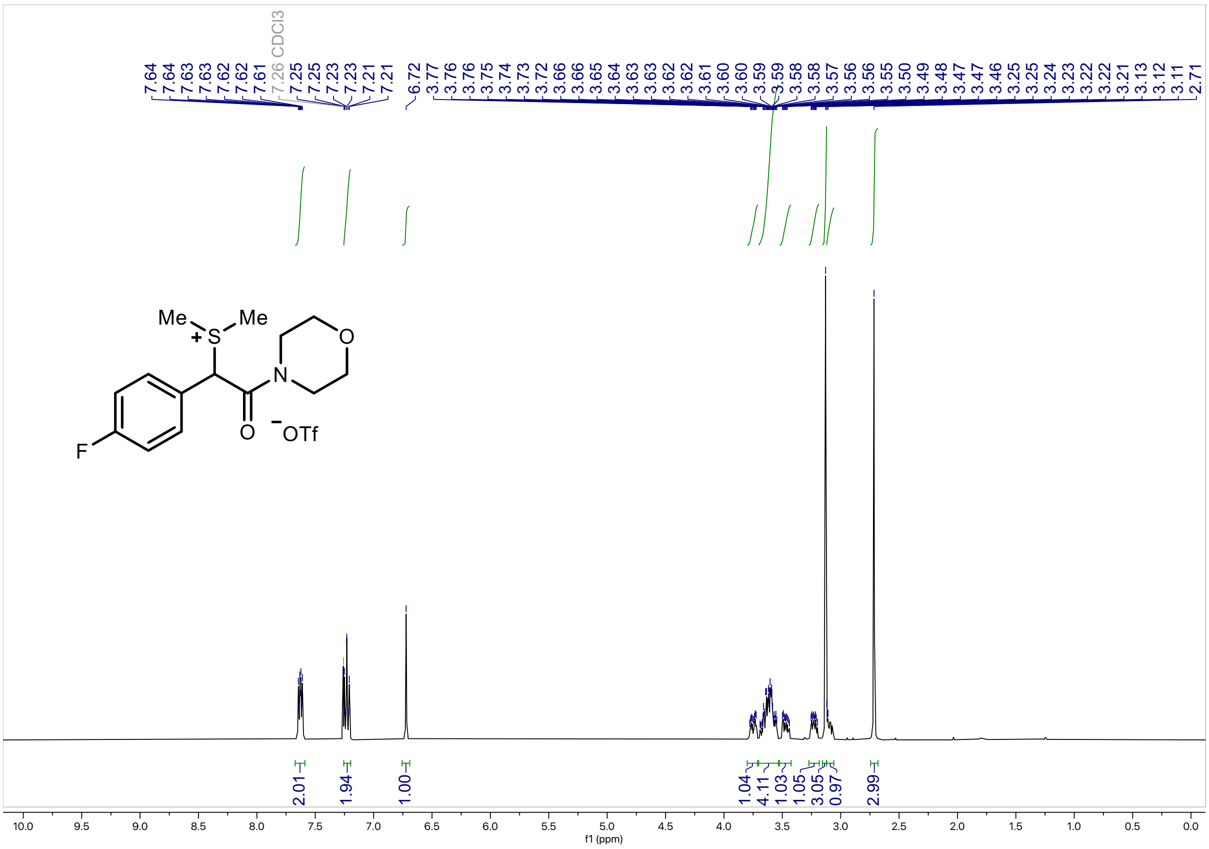

2i – ^{13}C NMR (101 MHz, $CDCl_3$)

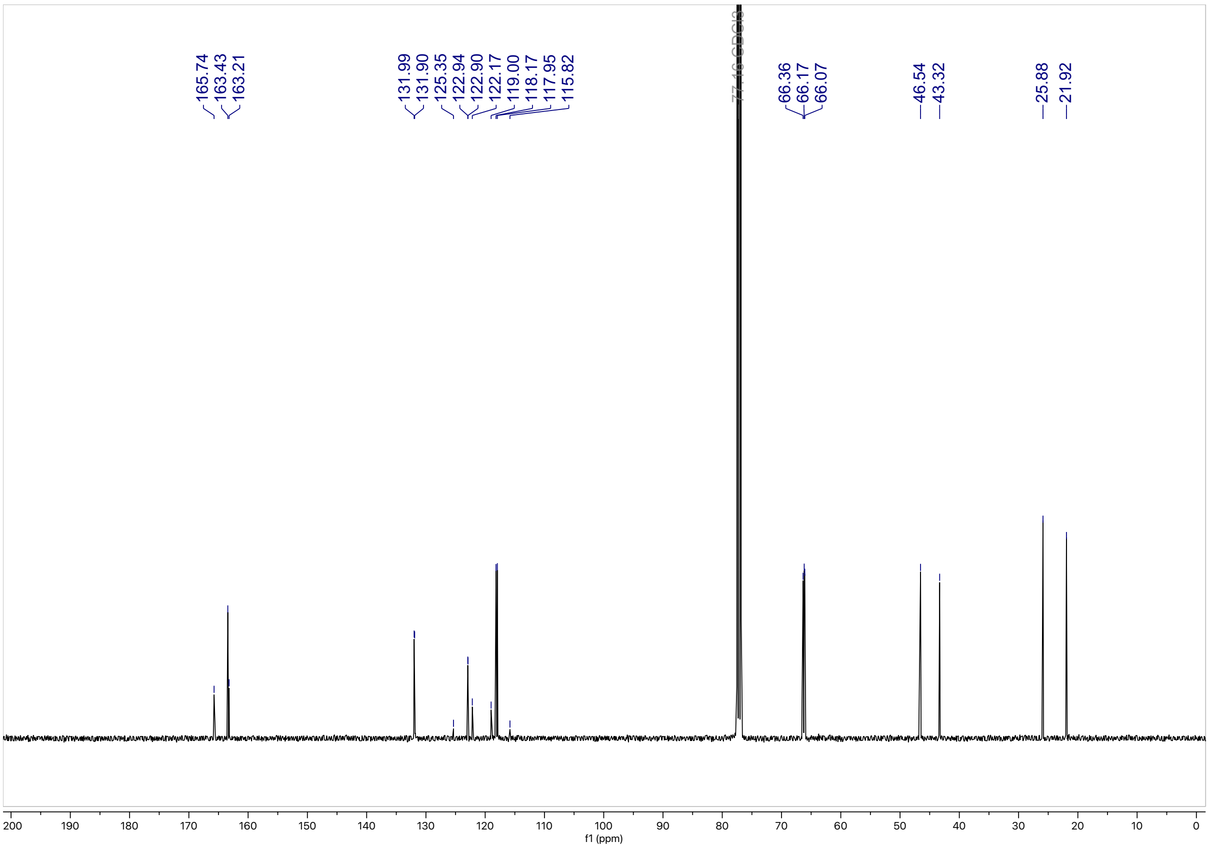

2i – ^{19}F NMR (376 MHz, $CDCl_3$)

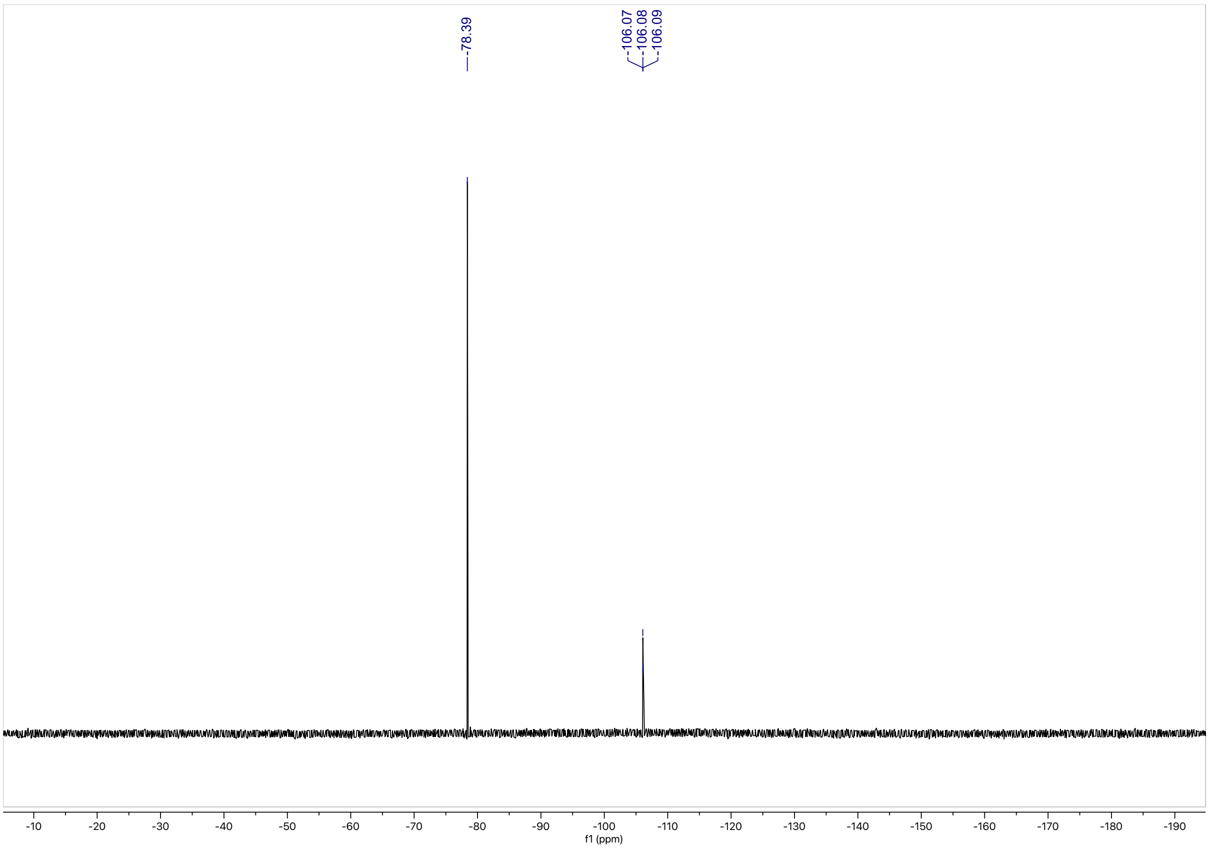

3i – ^{1}H NMR (400 MHz, $CDCl_3$)

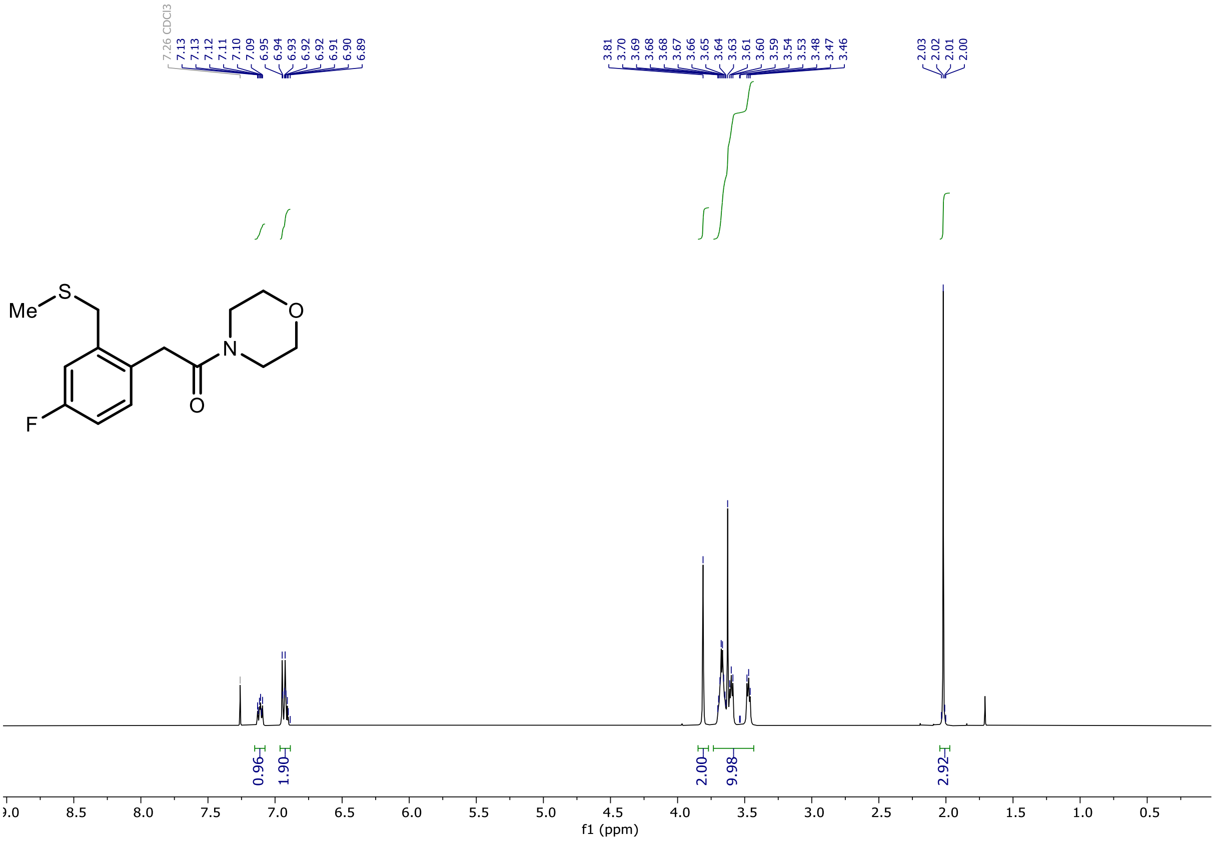

3i – ^{13}C NMR (101 MHz, $CDCl_3$)

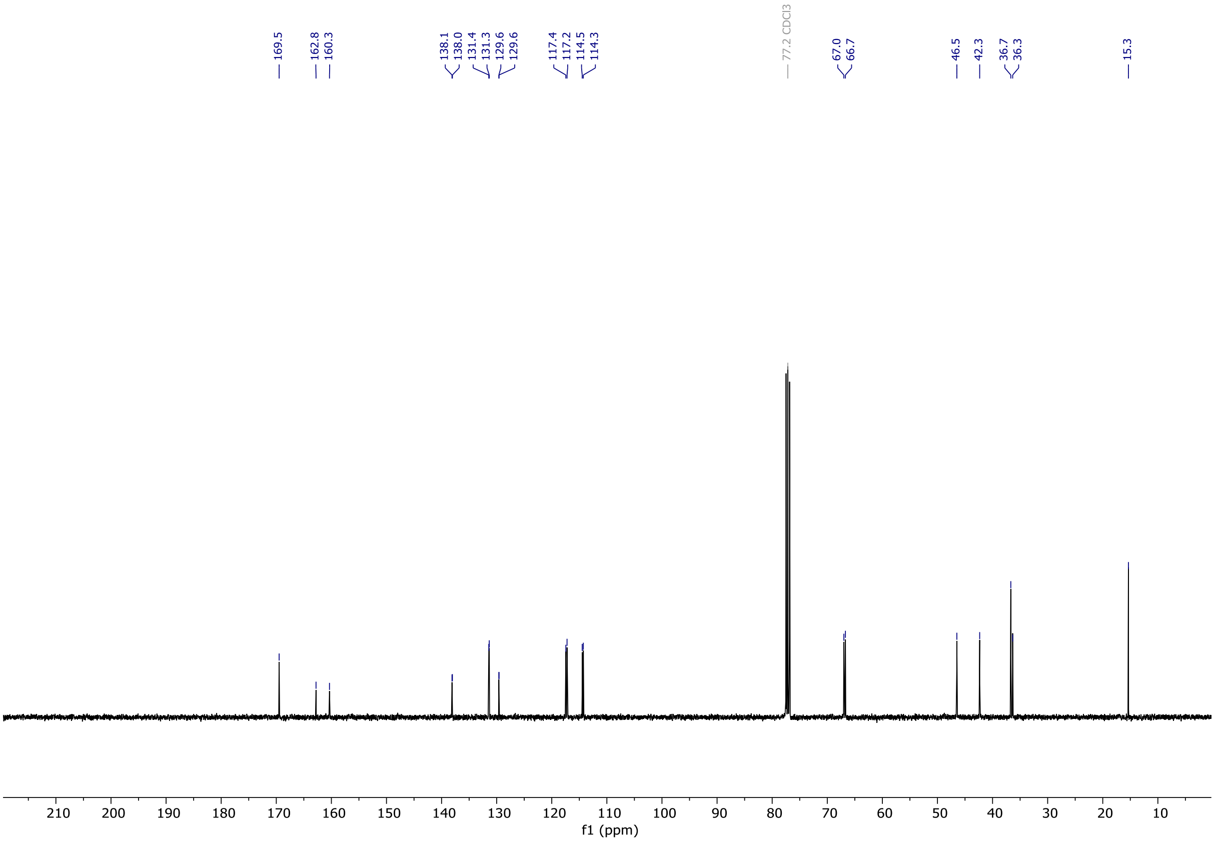

3i – ^{19}F NMR (376 MHz, $CDCl_3$)

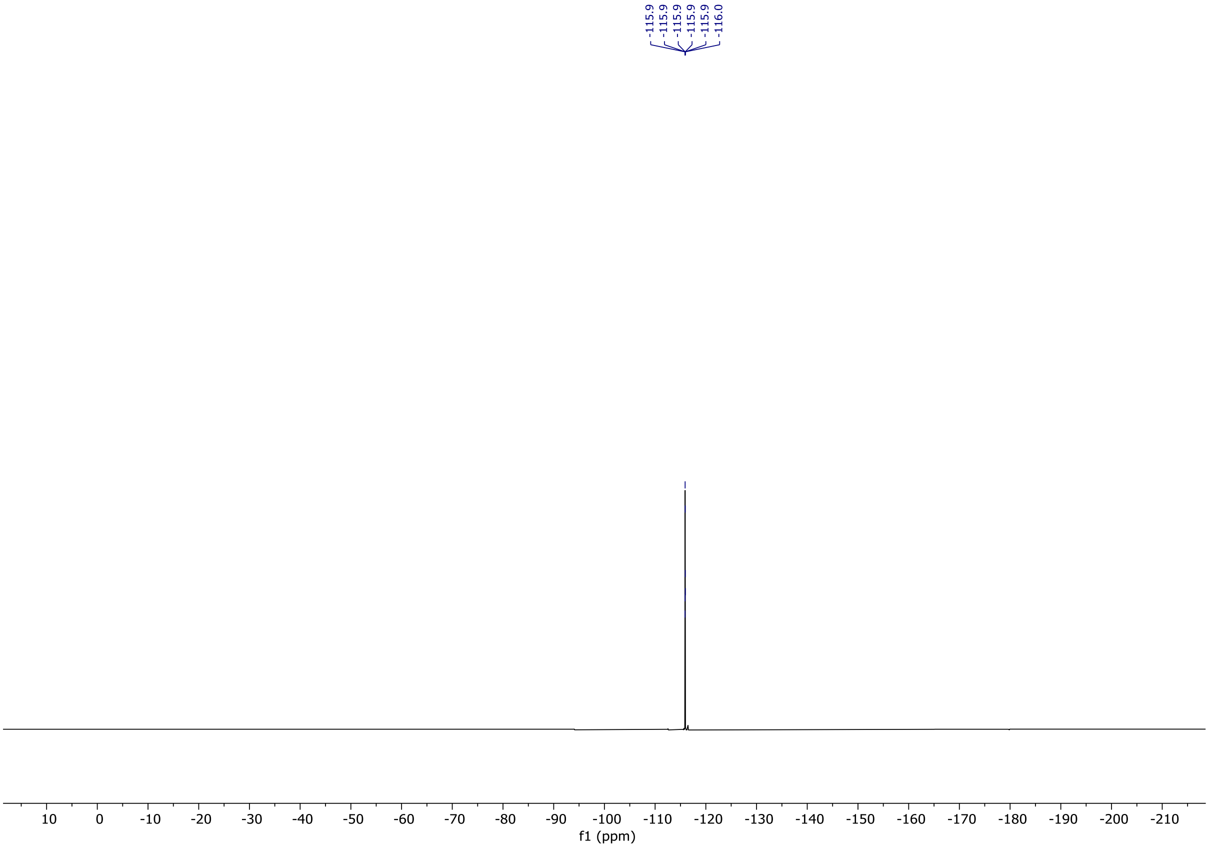

2j – ^{1}H NMR (400 MHz, CD_3CN)

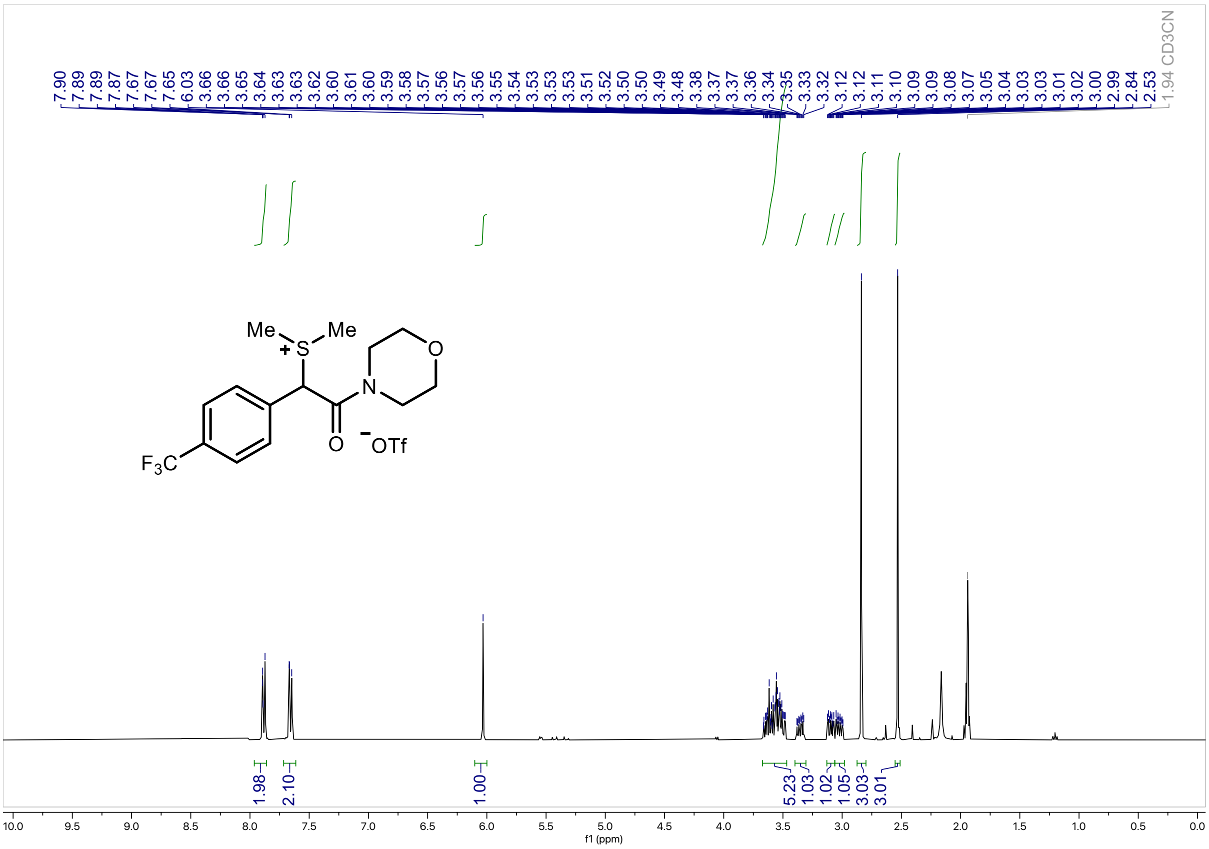

2j – ^{13}C NMR (101 MHz, CD_3CN)

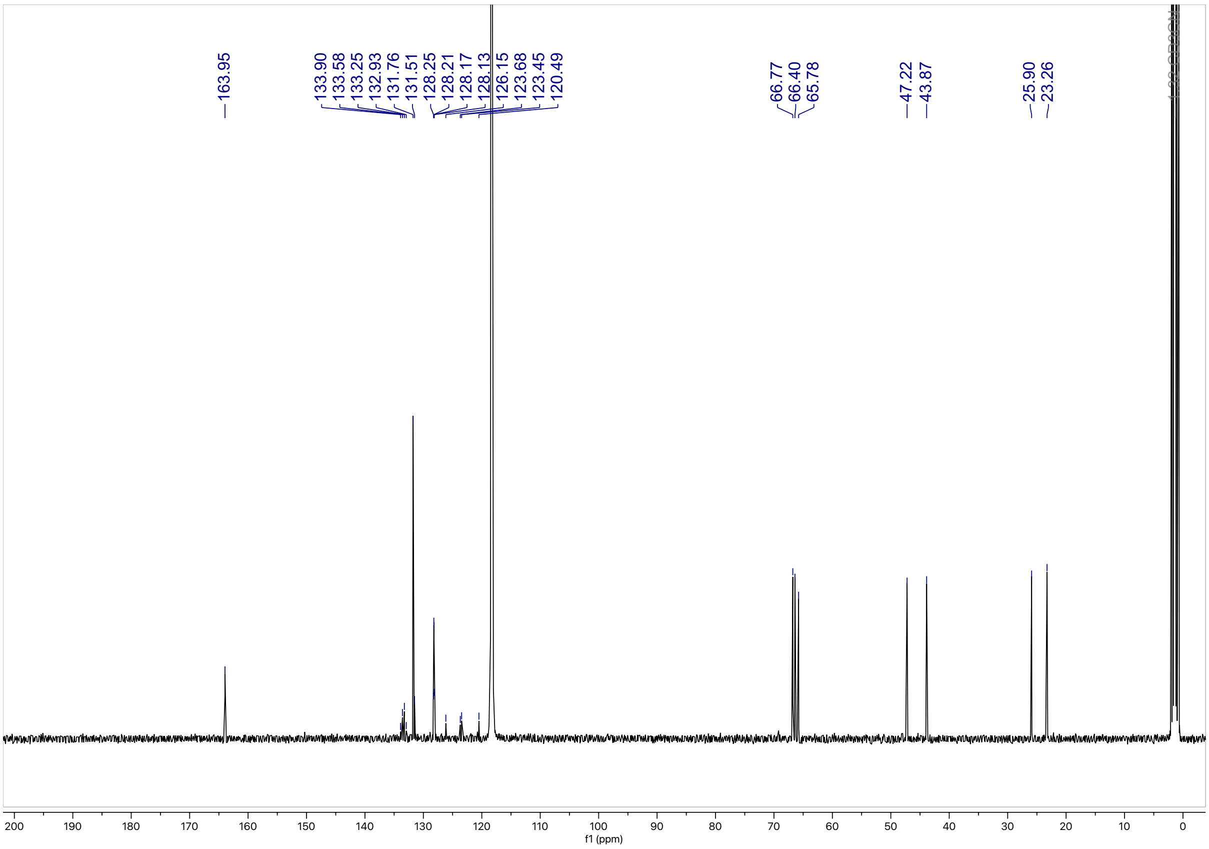

2j – ^{19}F NMR (376 MHz, CD_3CN)

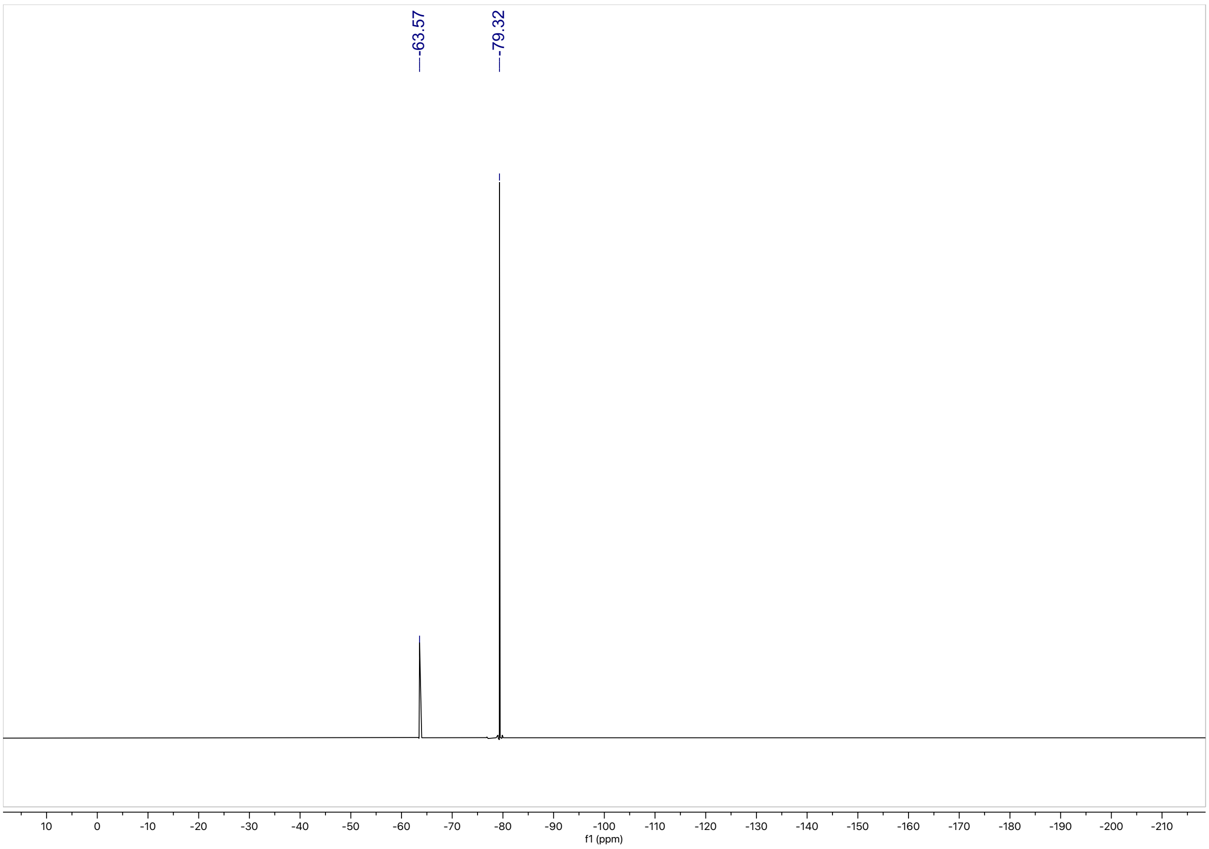

3j – ^{1}H NMR (400 MHz, $CDCl_3$)

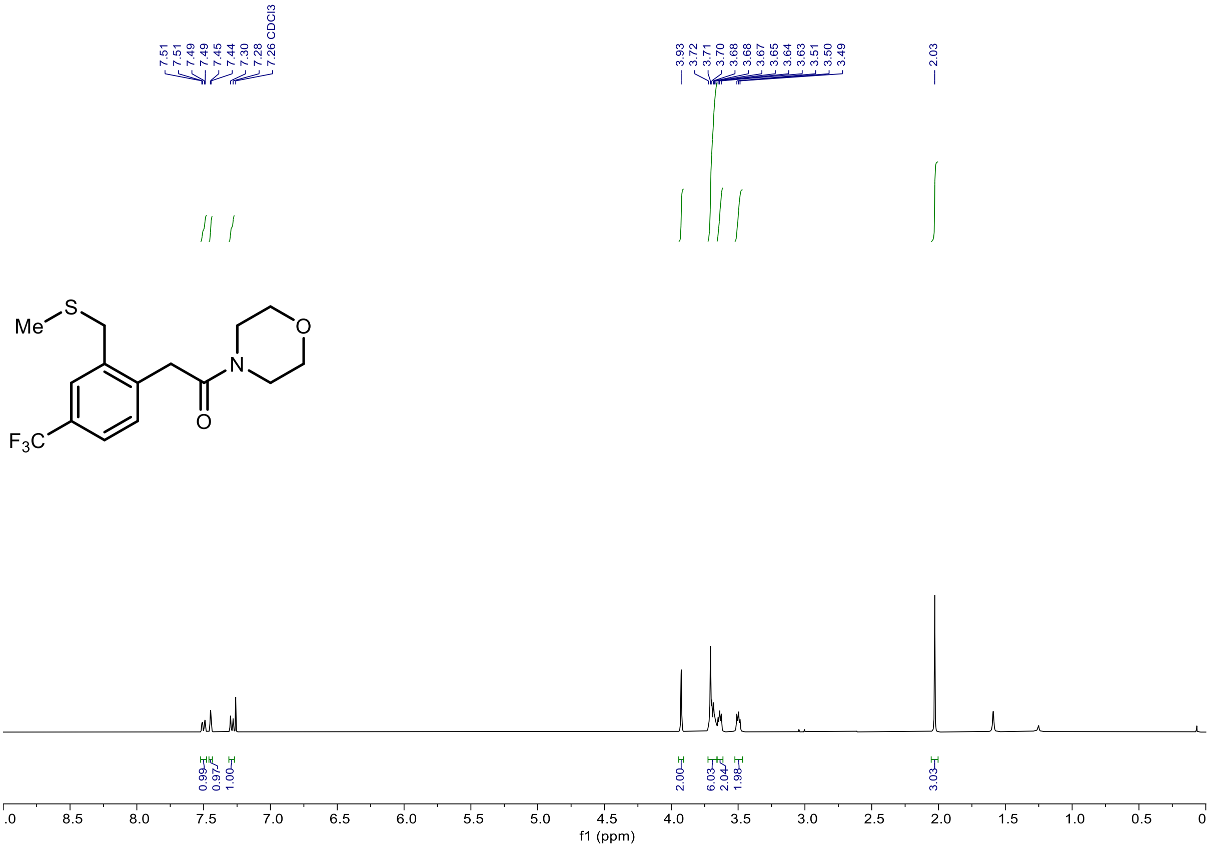

3j – ^{13}C NMR (101 MHz, $CDCl_3$)

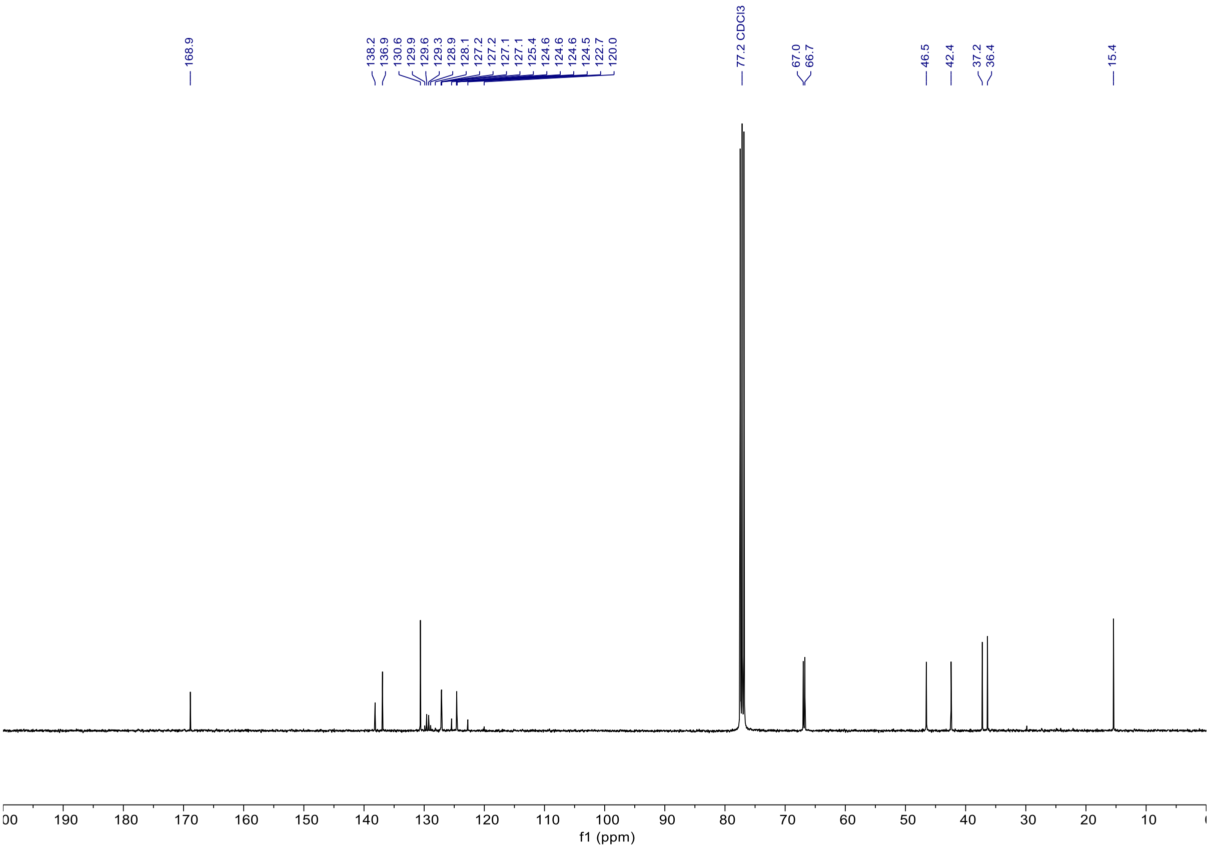

3j – ^{19}F NMR (376 MHz, $CDCl_3$)

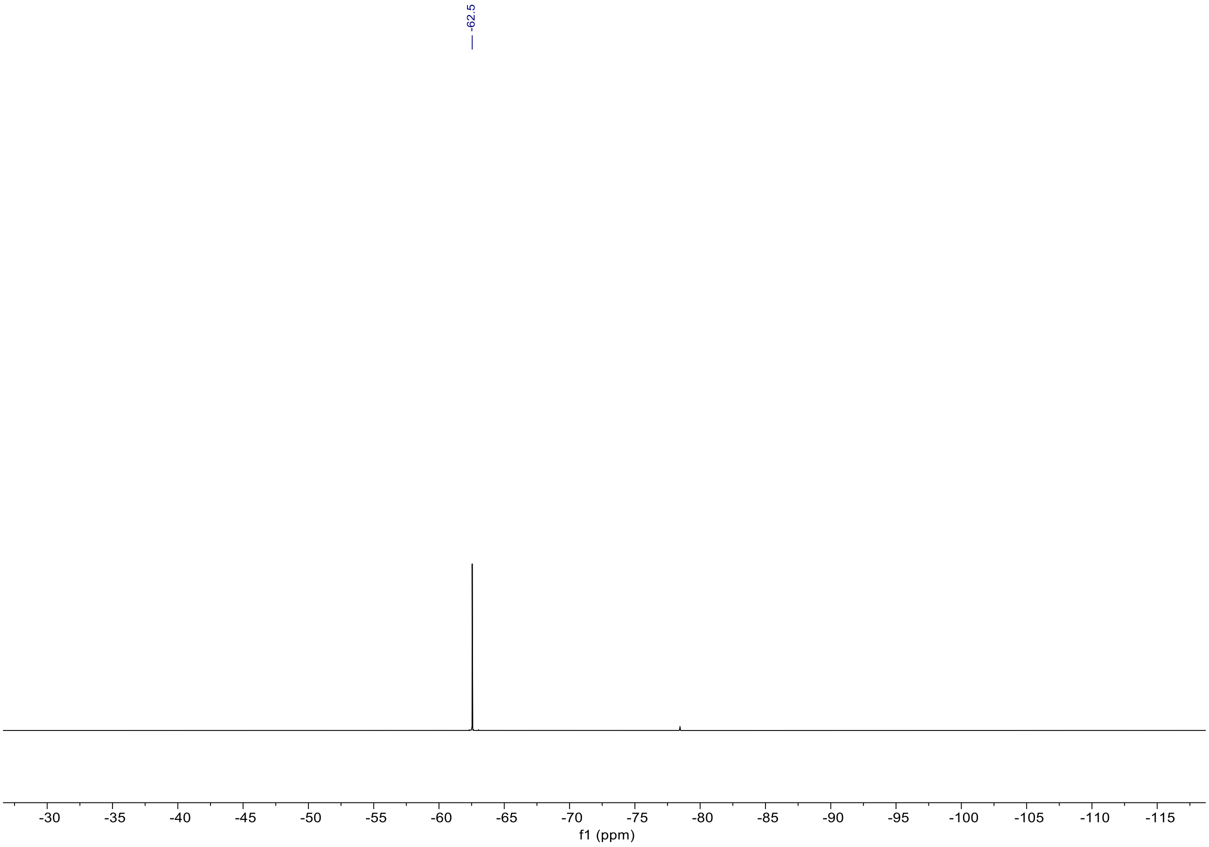

3k – ^{1}H NMR (500 MHz, $CDCl_3$)

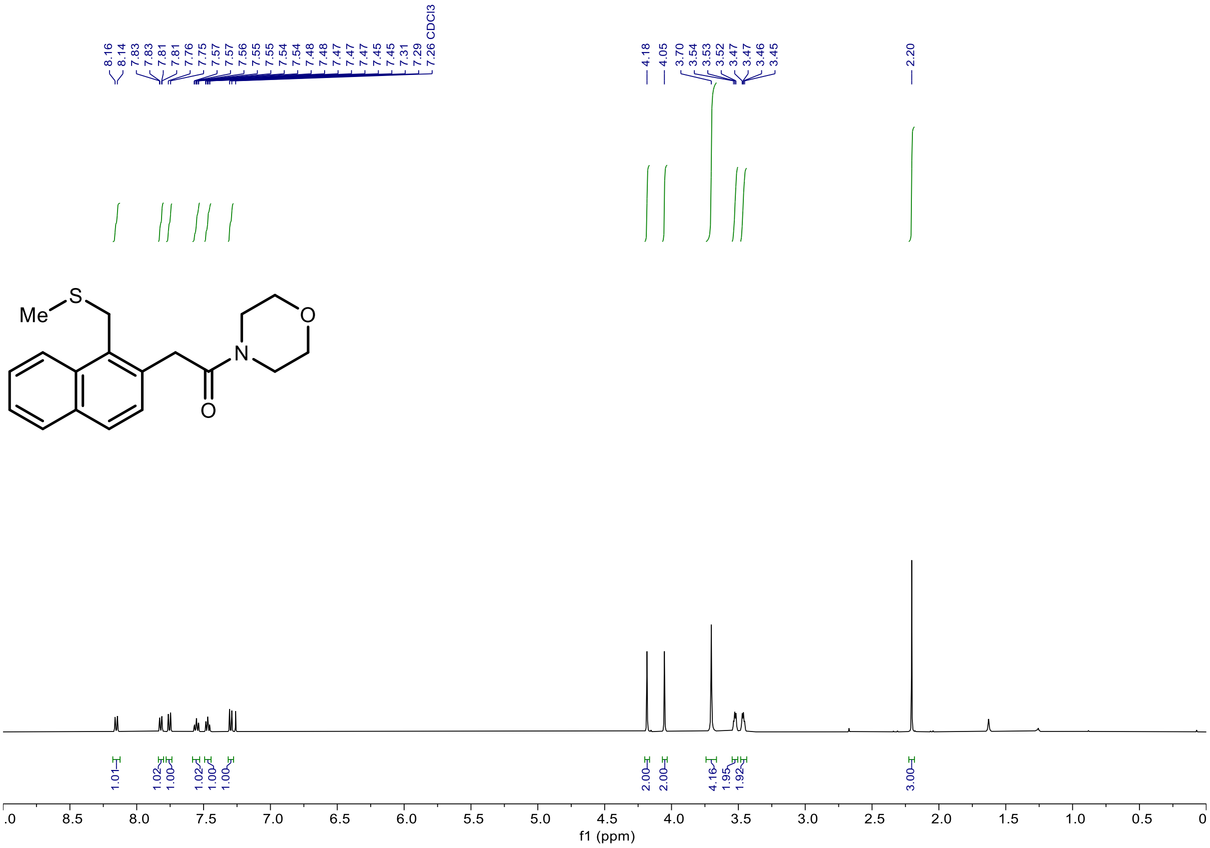

3k – ^{13}C NMR (126 MHz, $CDCl_3$)

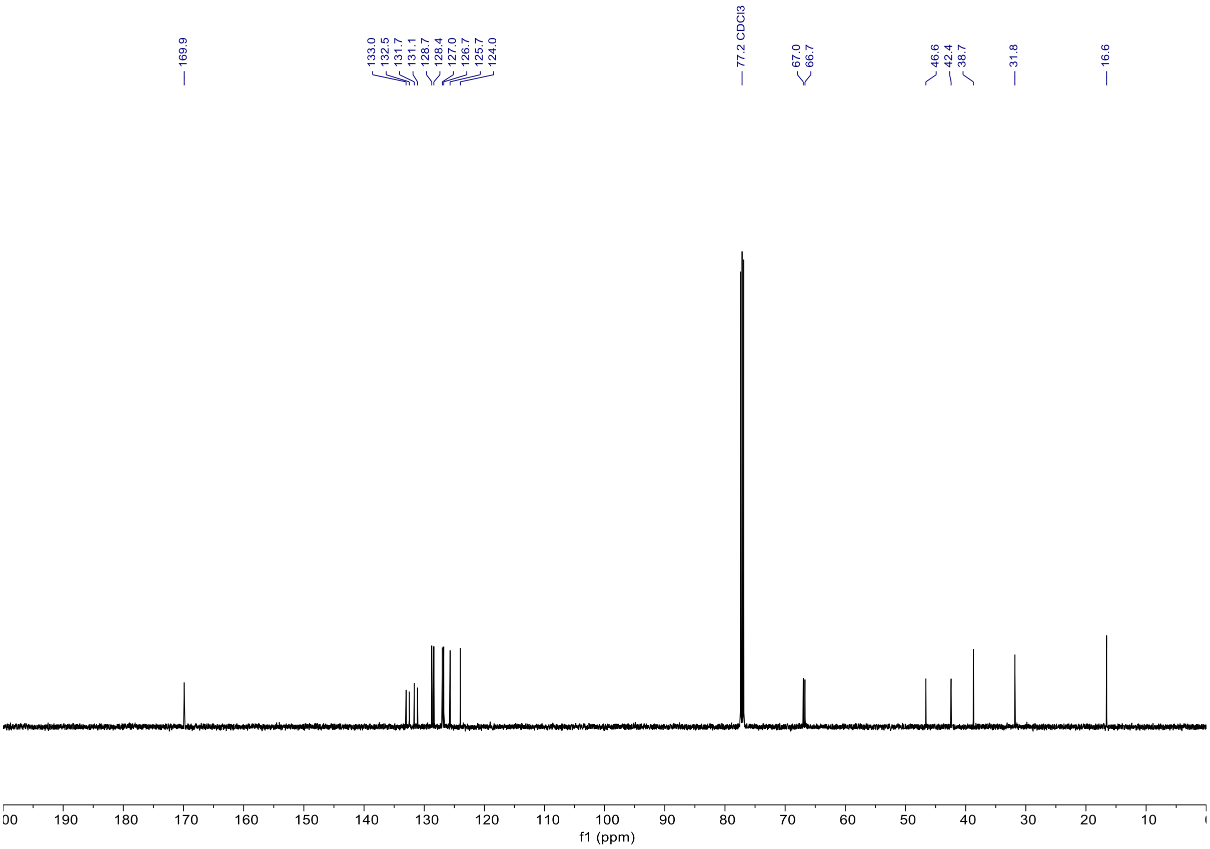

2l – ^{1}H NMR (400 MHz, CD_3CN)

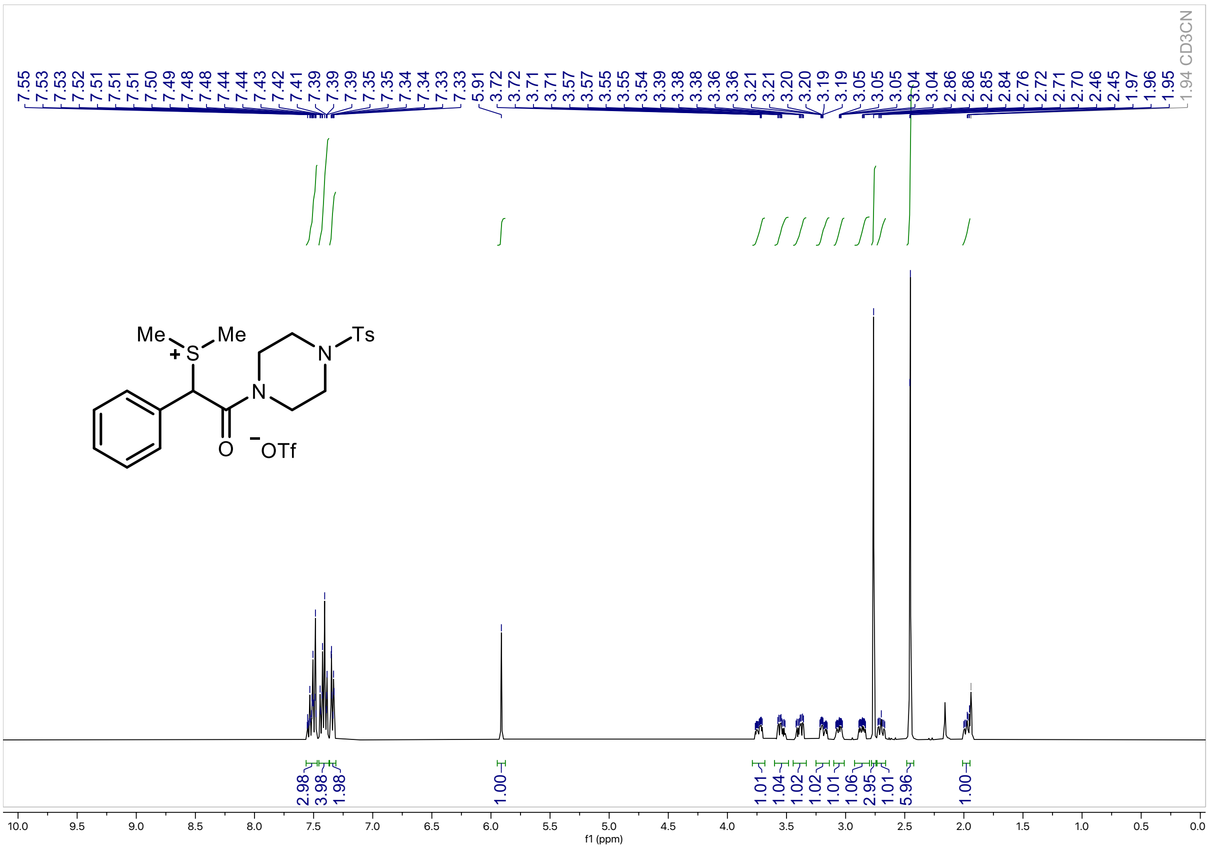

2l – ^{13}C NMR (101 MHz, CD_3CN)

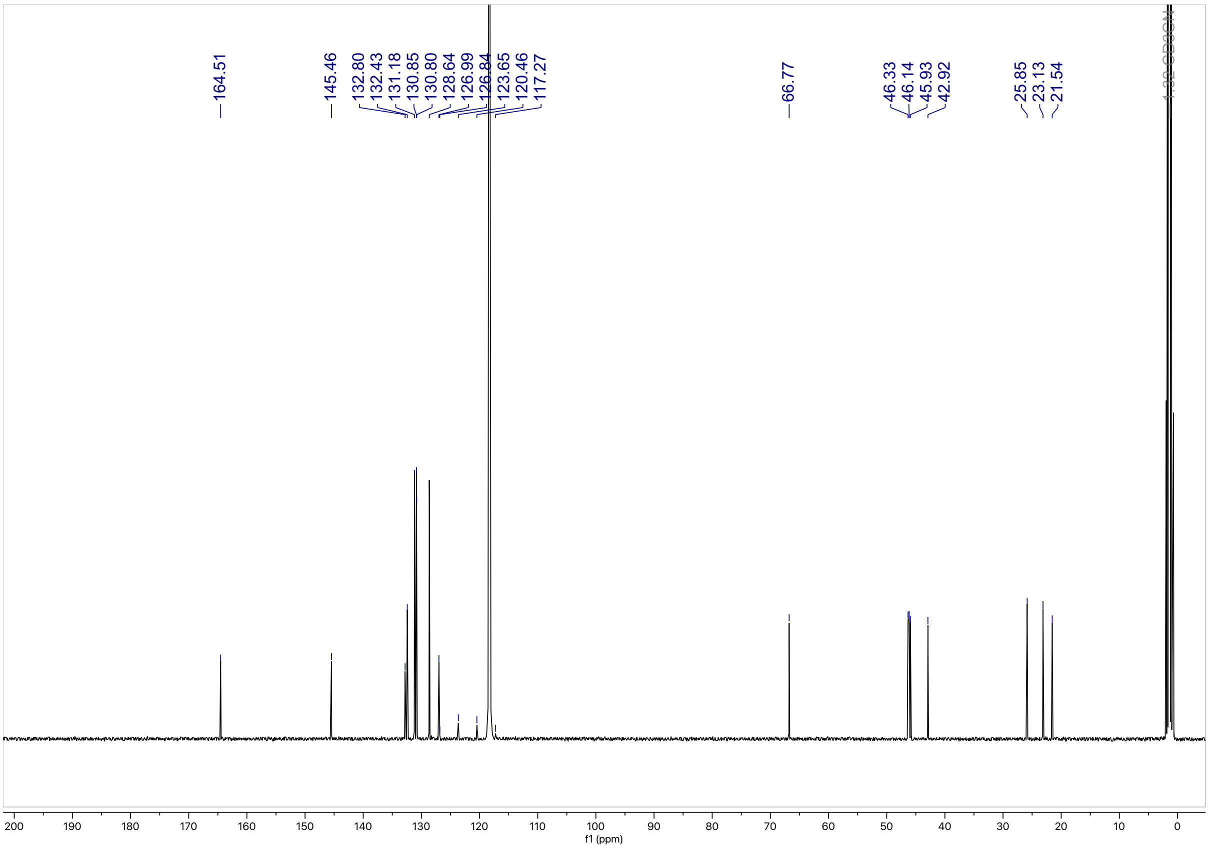

2l – ^{19}F NMR (376 MHz, CD_3CN)

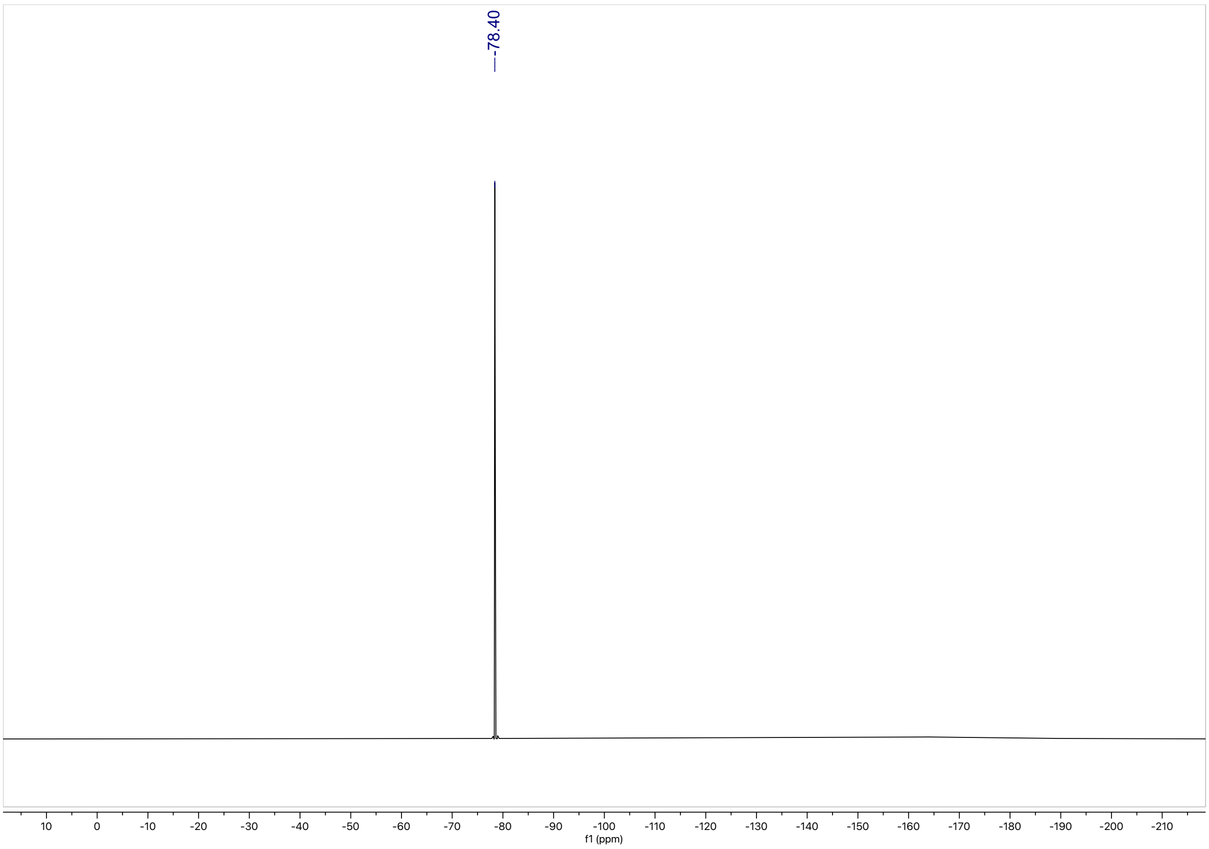

3l – ^{1}H NMR (500 MHz, CDCl$_3$)

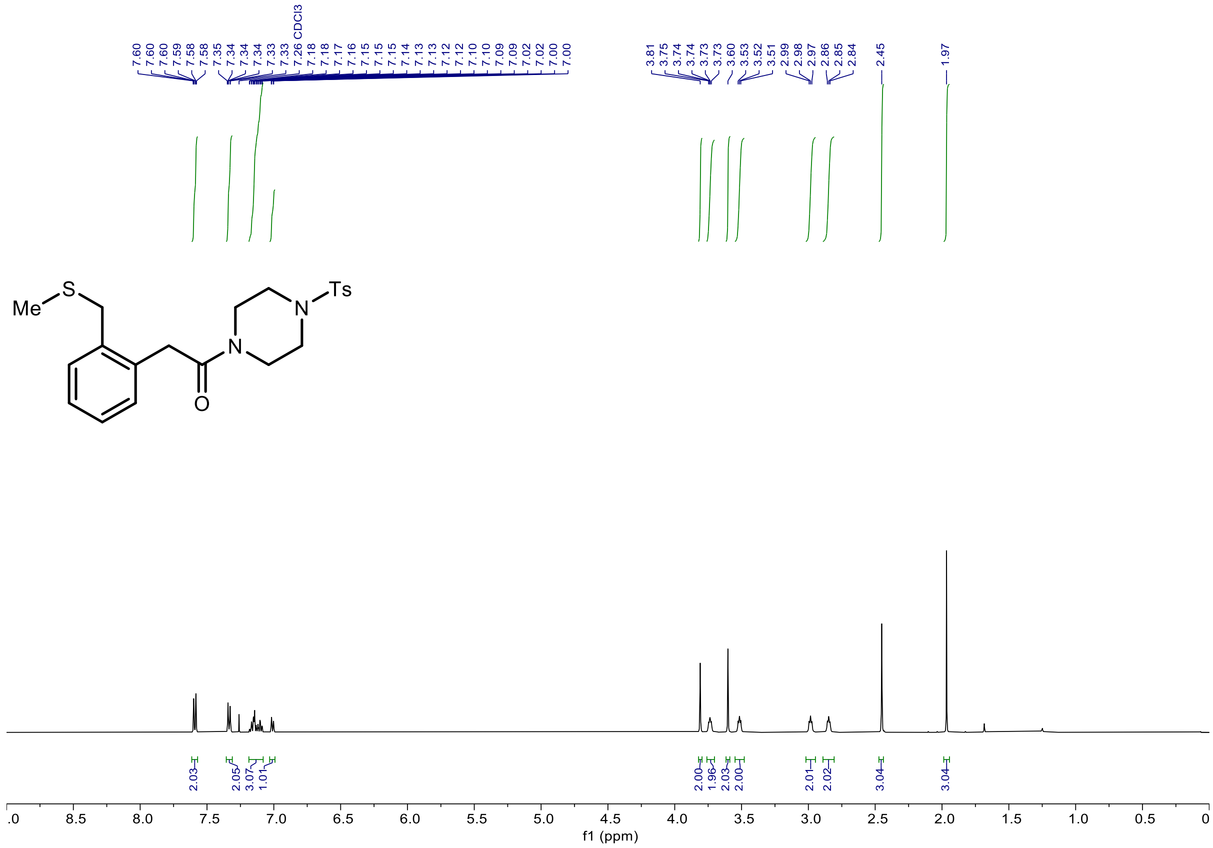

3l – ^{13}C NMR (126 MHz, CDCl$_3$)

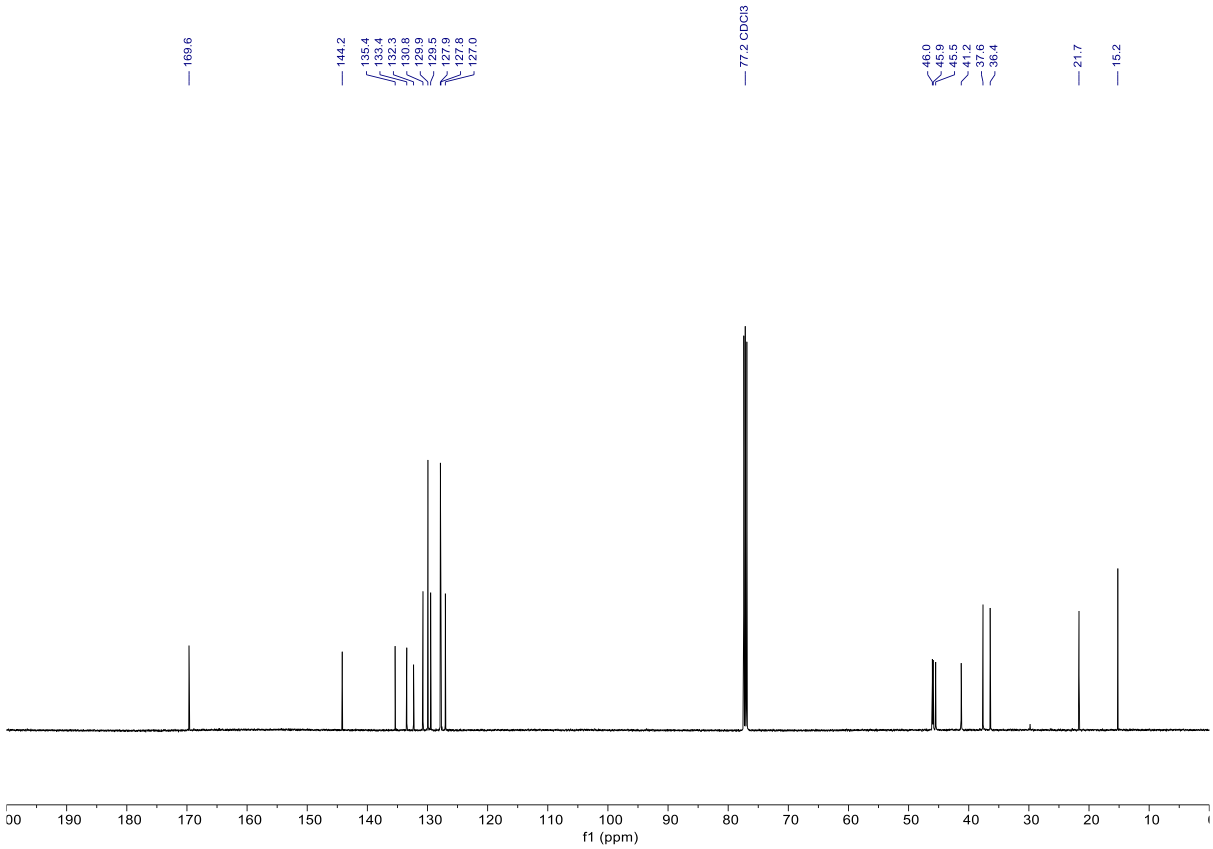

2m – ^{1}H NMR (400 MHz, CD_3CN)

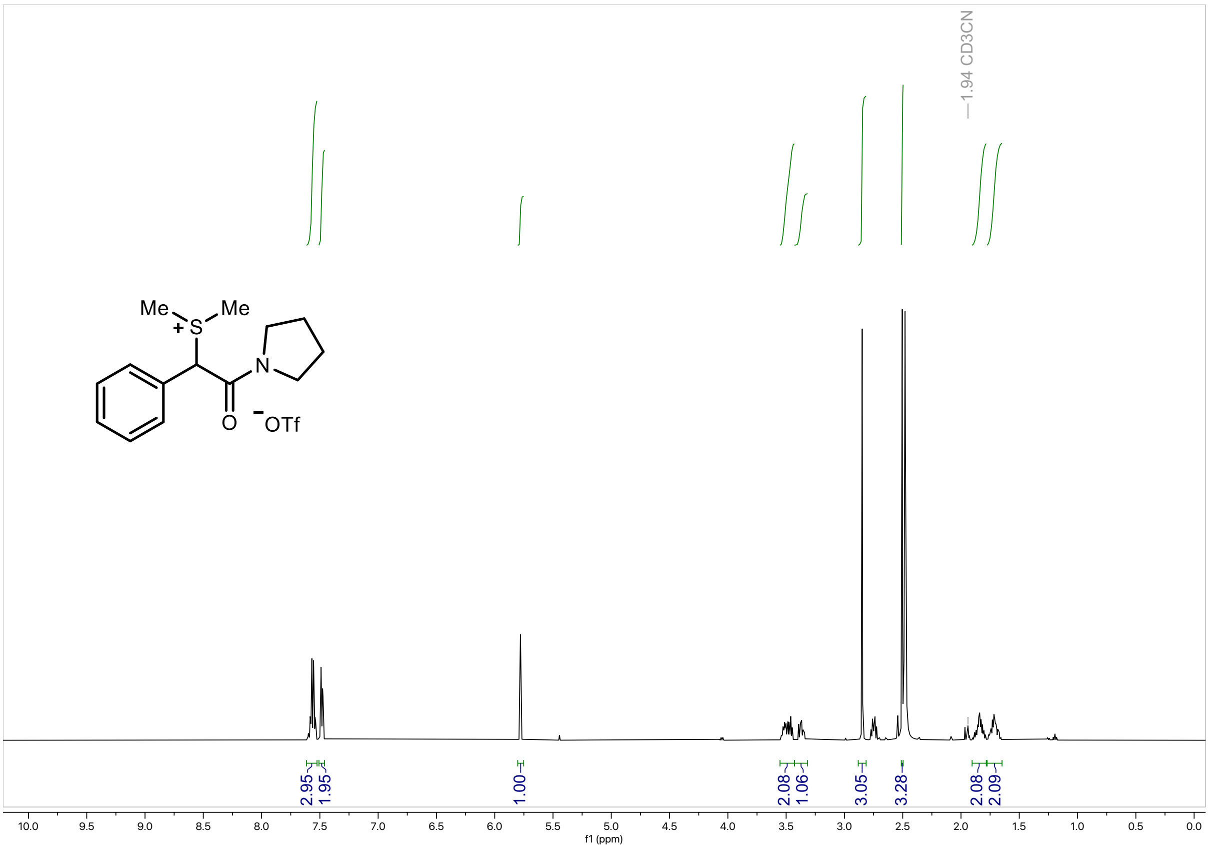

2m – ^{13}C NMR (101 MHz, CD_3CN)

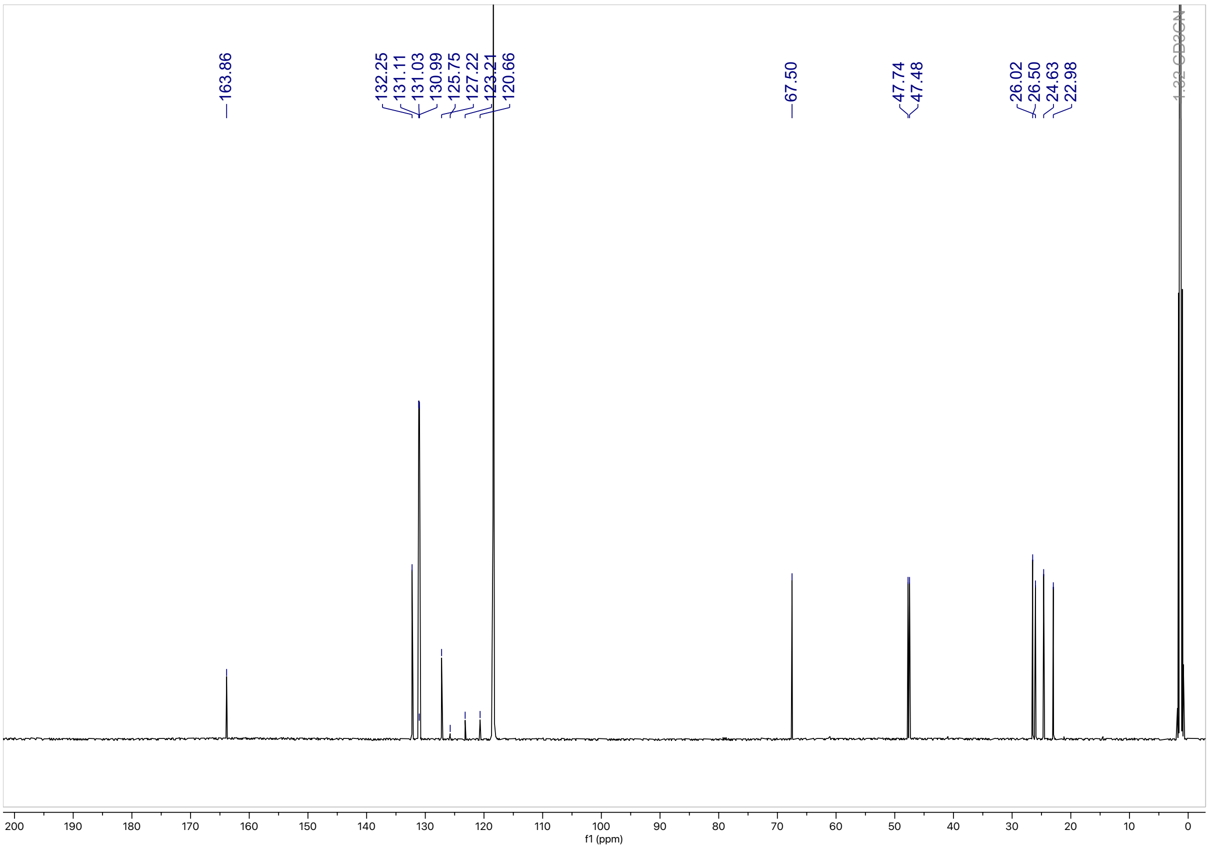

2m – ^{19}F NMR (376 MHz, CD_3CN)

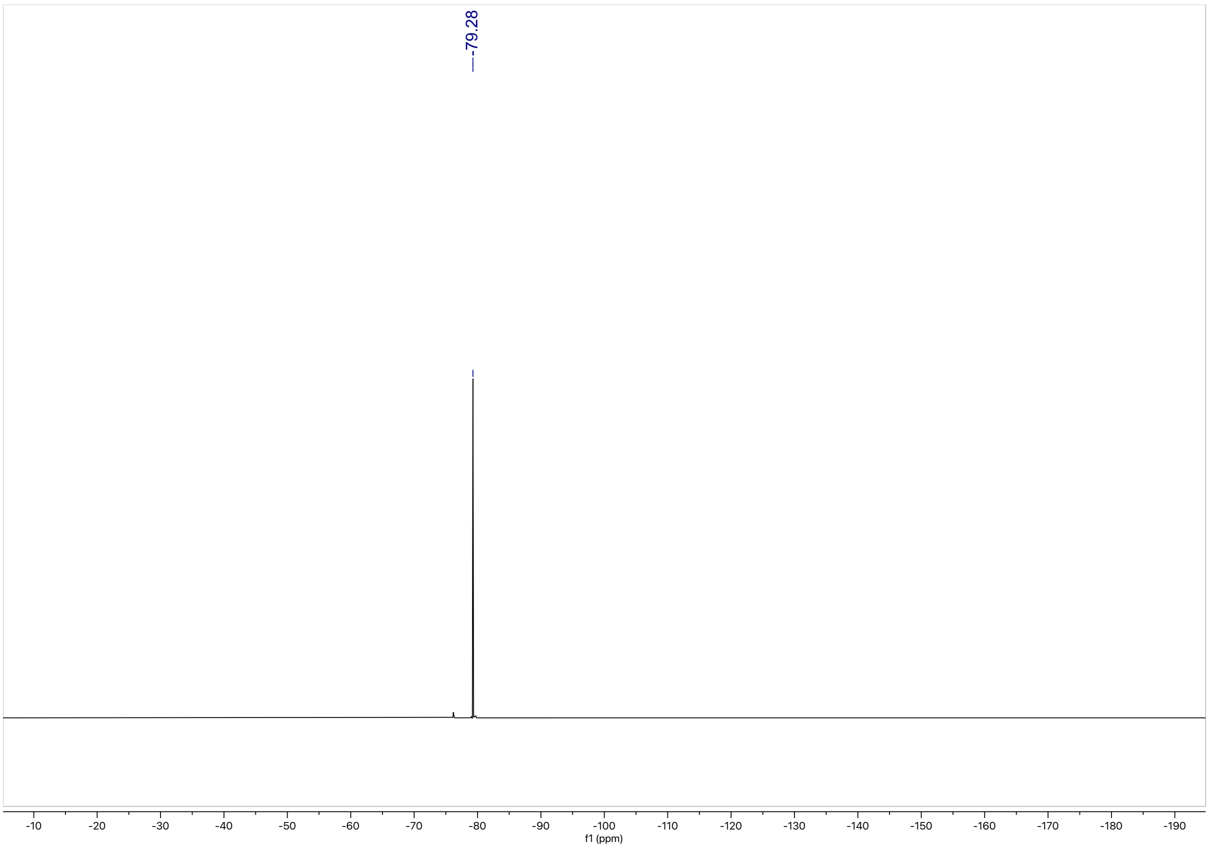

3m – ^{1}H NMR (400 MHz, $CDCl_3$)

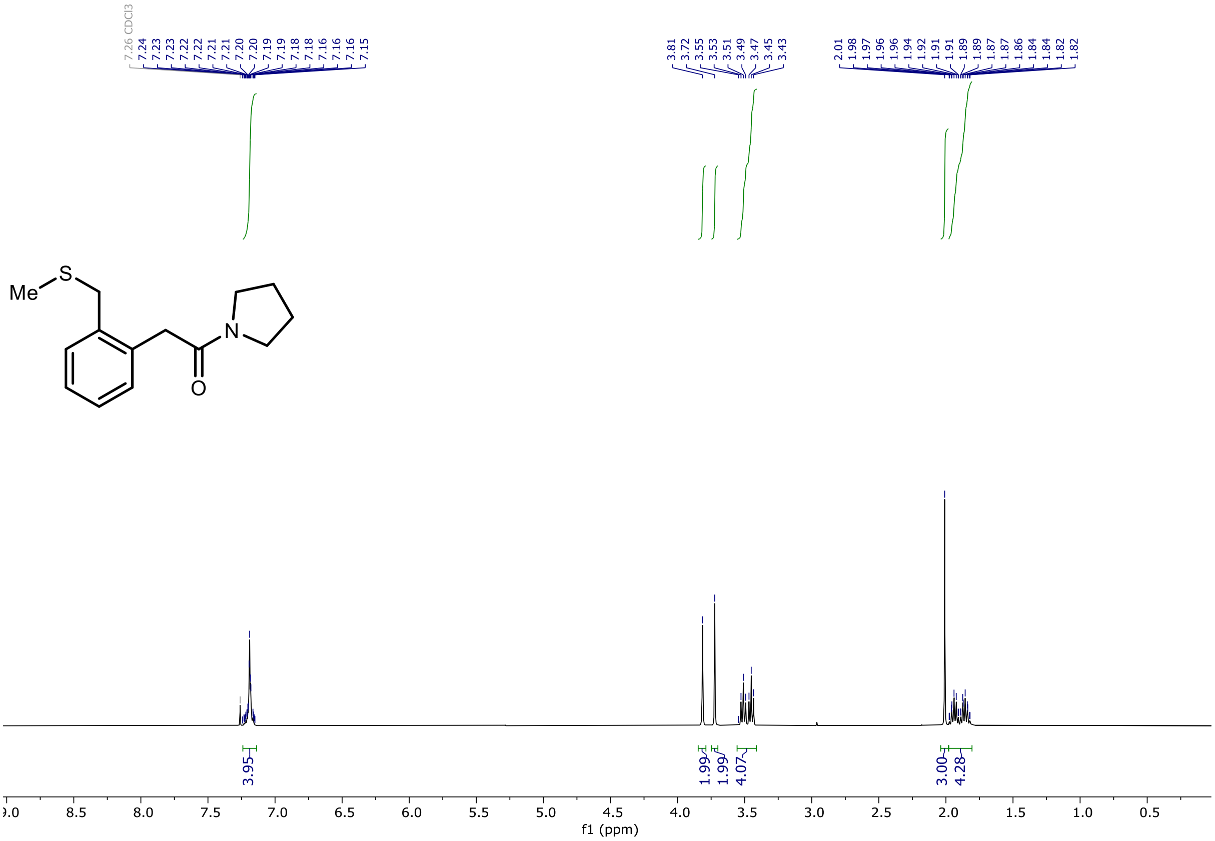

3m – ^{13}C NMR (101 MHz, $CDCl_3$)

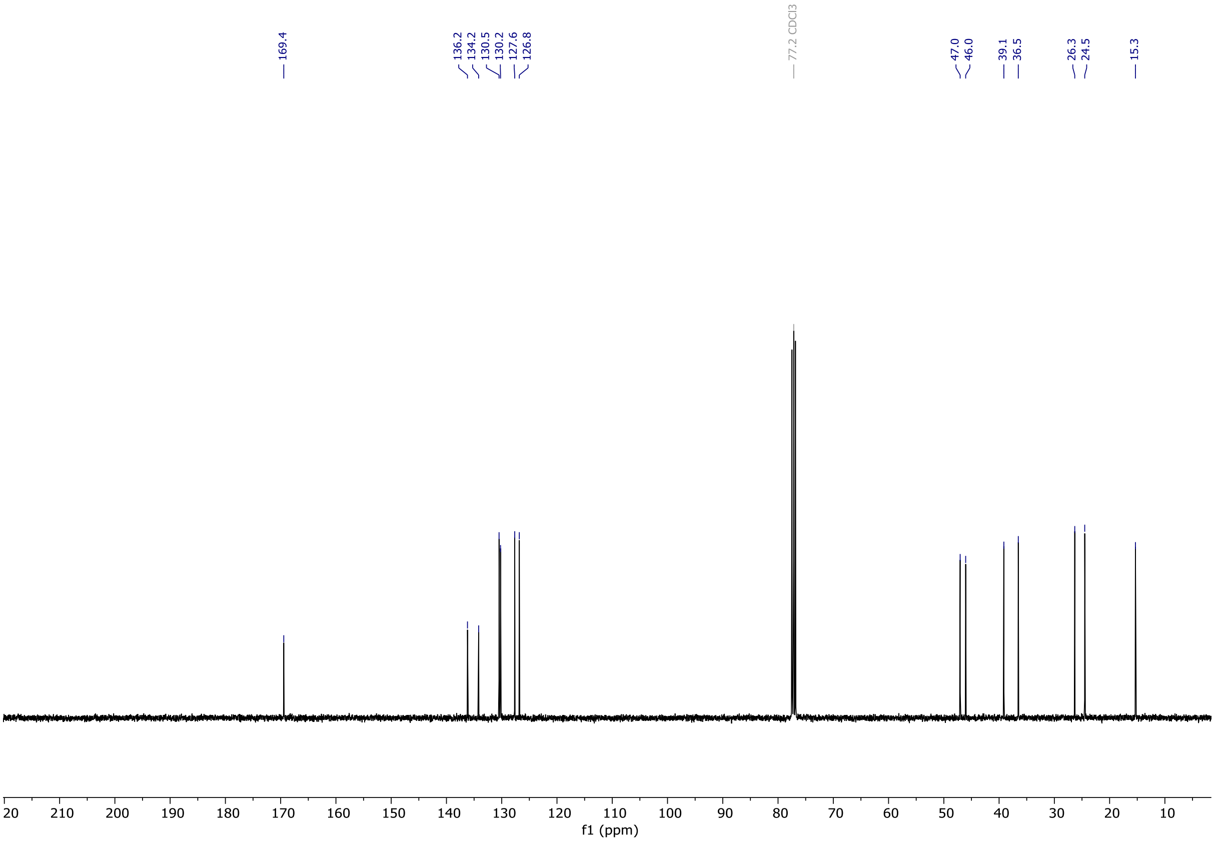

2n – ^{1}H NMR (400 MHz, CD_3CN)

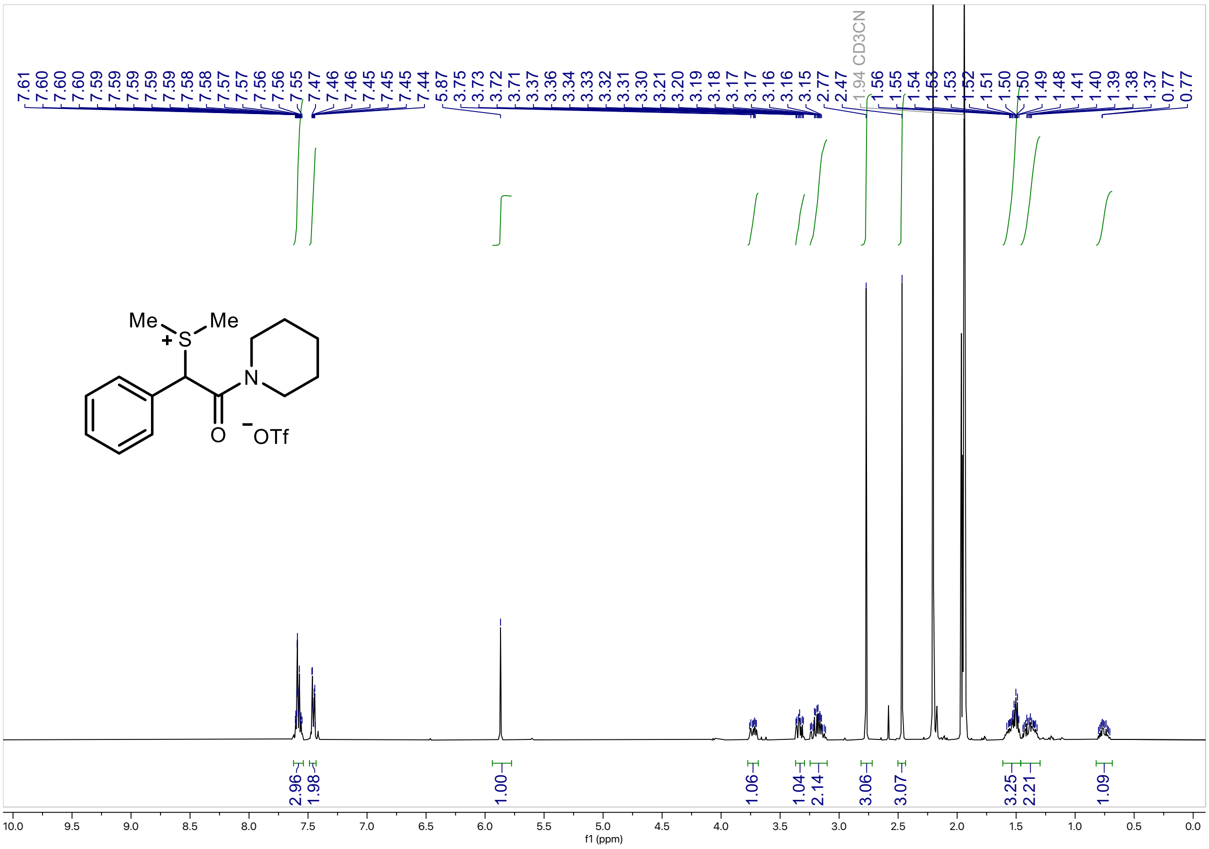

2n – ^{13}C NMR (101 MHz, CD_3CN)

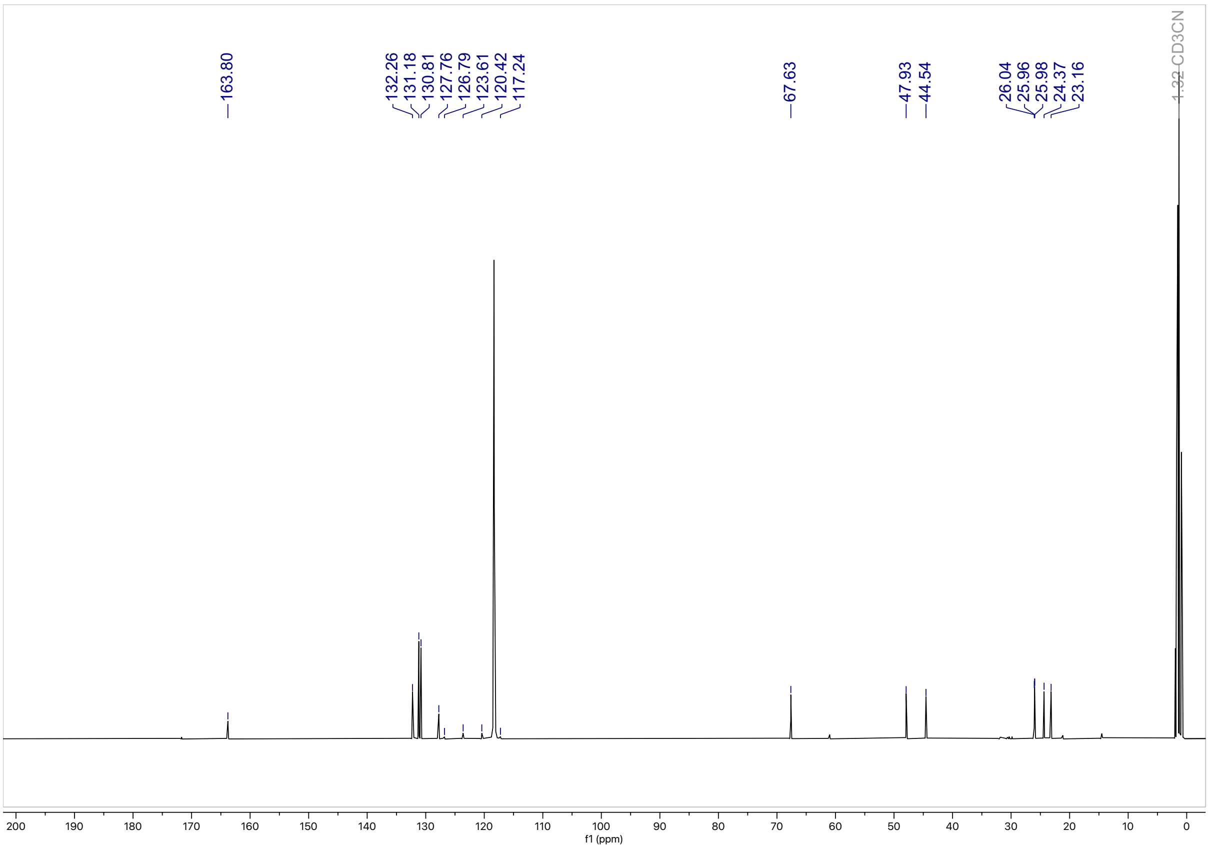

2n – ^{19}F NMR (376 MHz, CD_3CN)

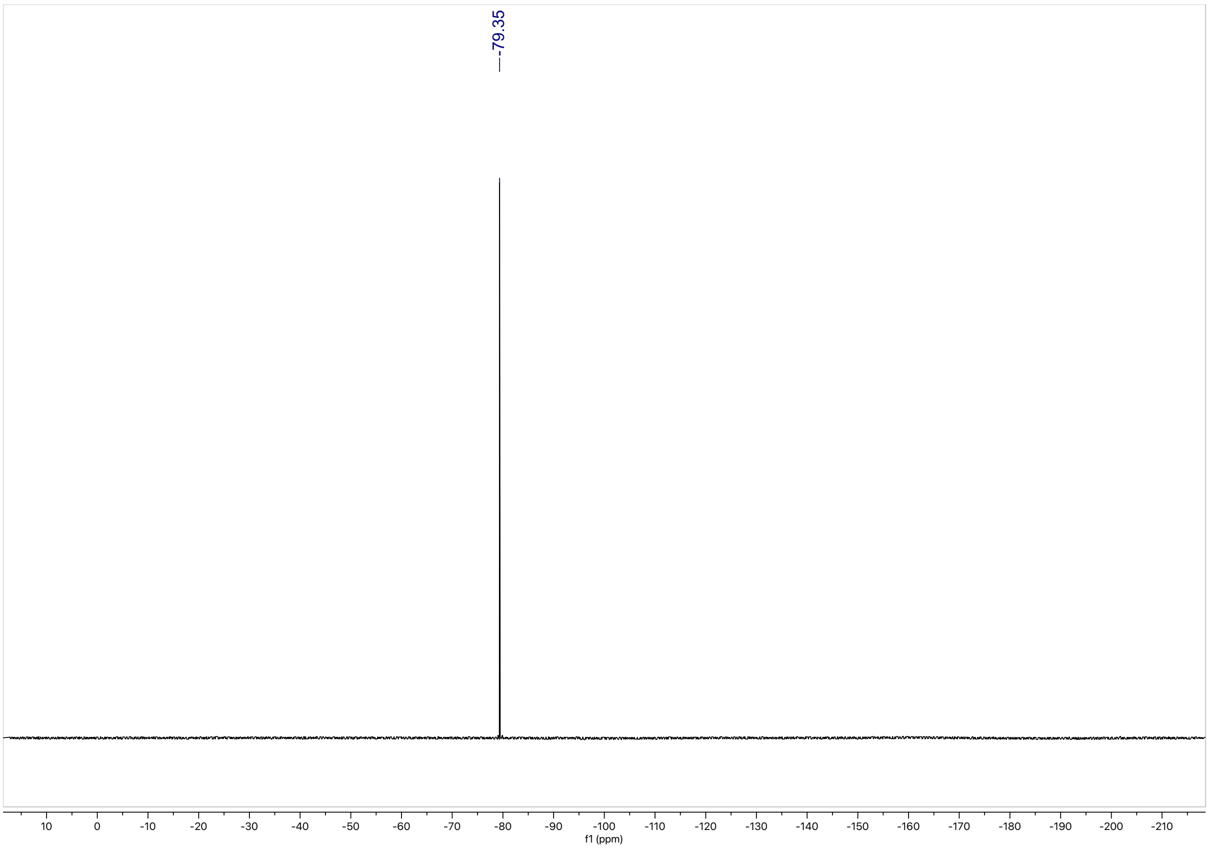

3n – ^{1}H NMR (400 MHz, $CDCl_3$)

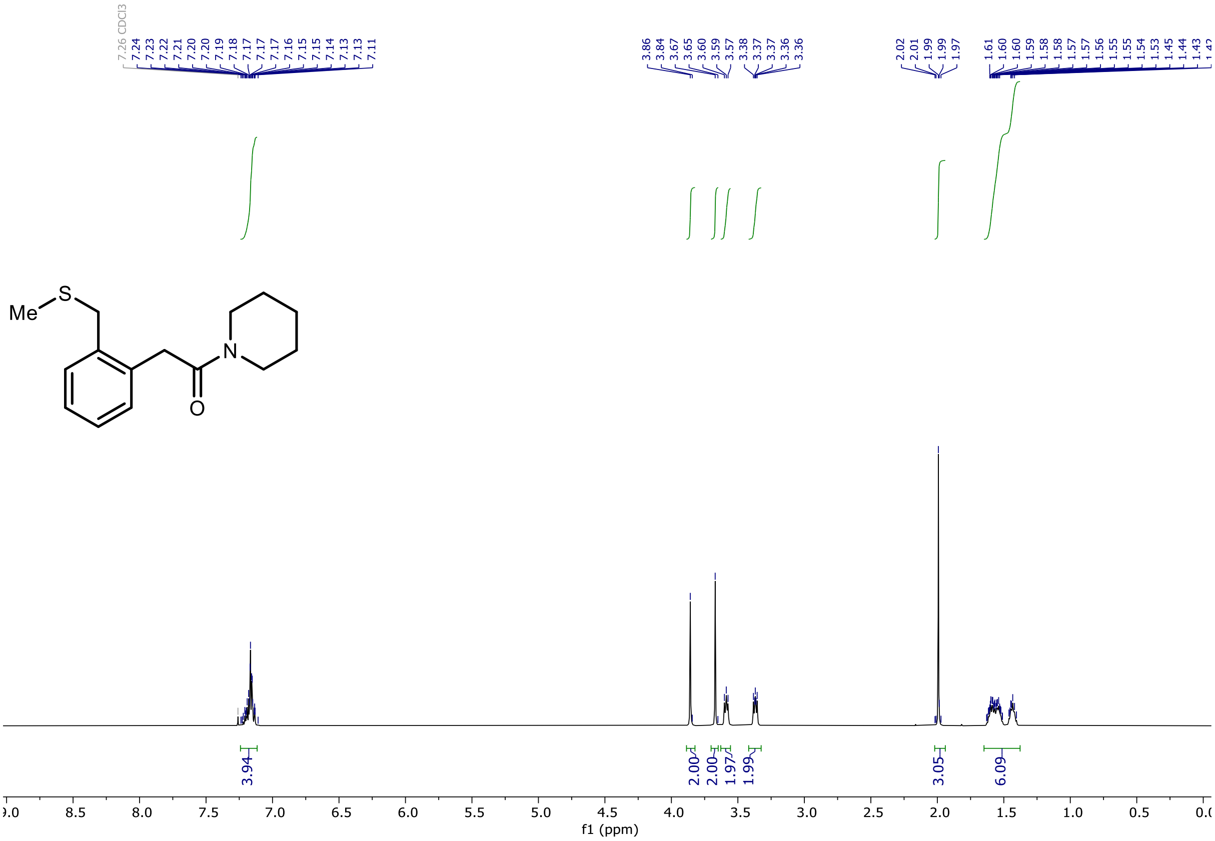

3n – ^{13}C NMR (101 MHz, $CDCl_3$)

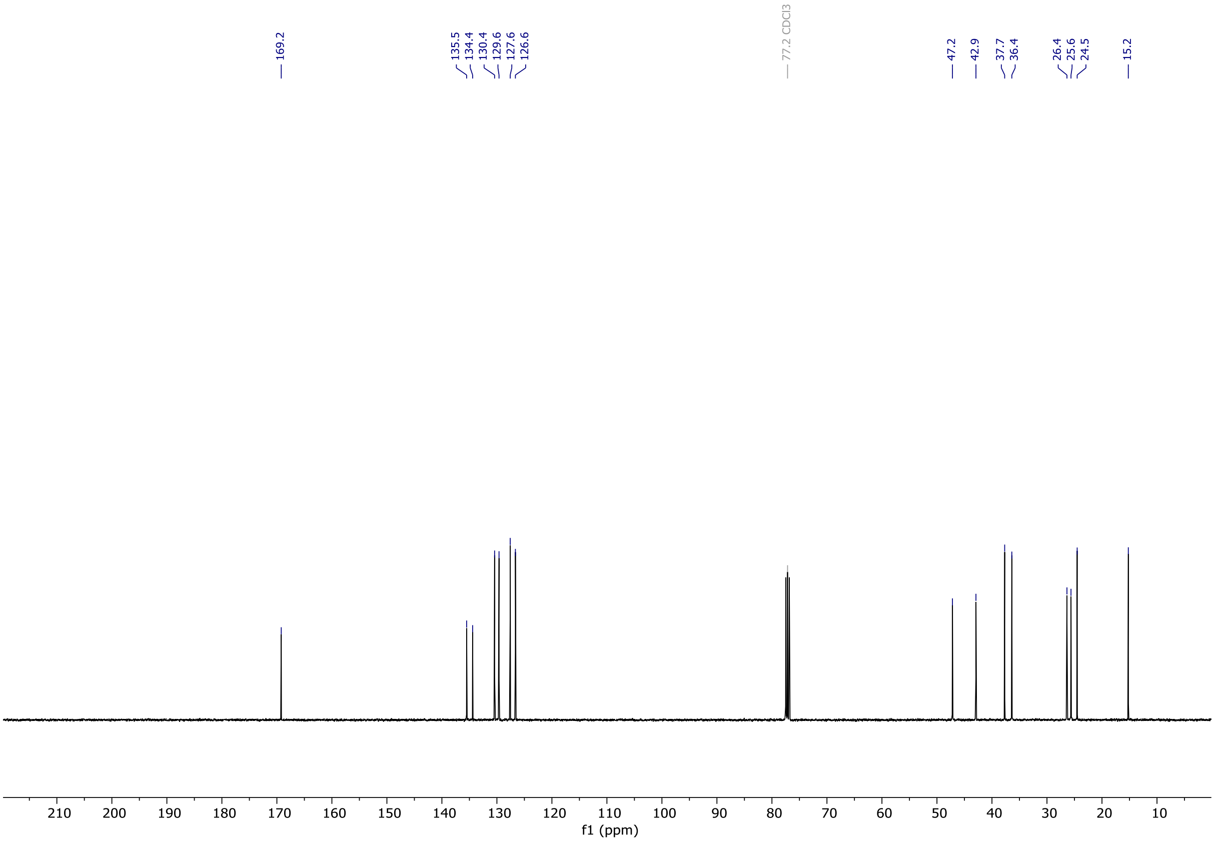

3o – ^{1}H NMR (400 MHz, $CDCl_3$, mixture of rotamers)

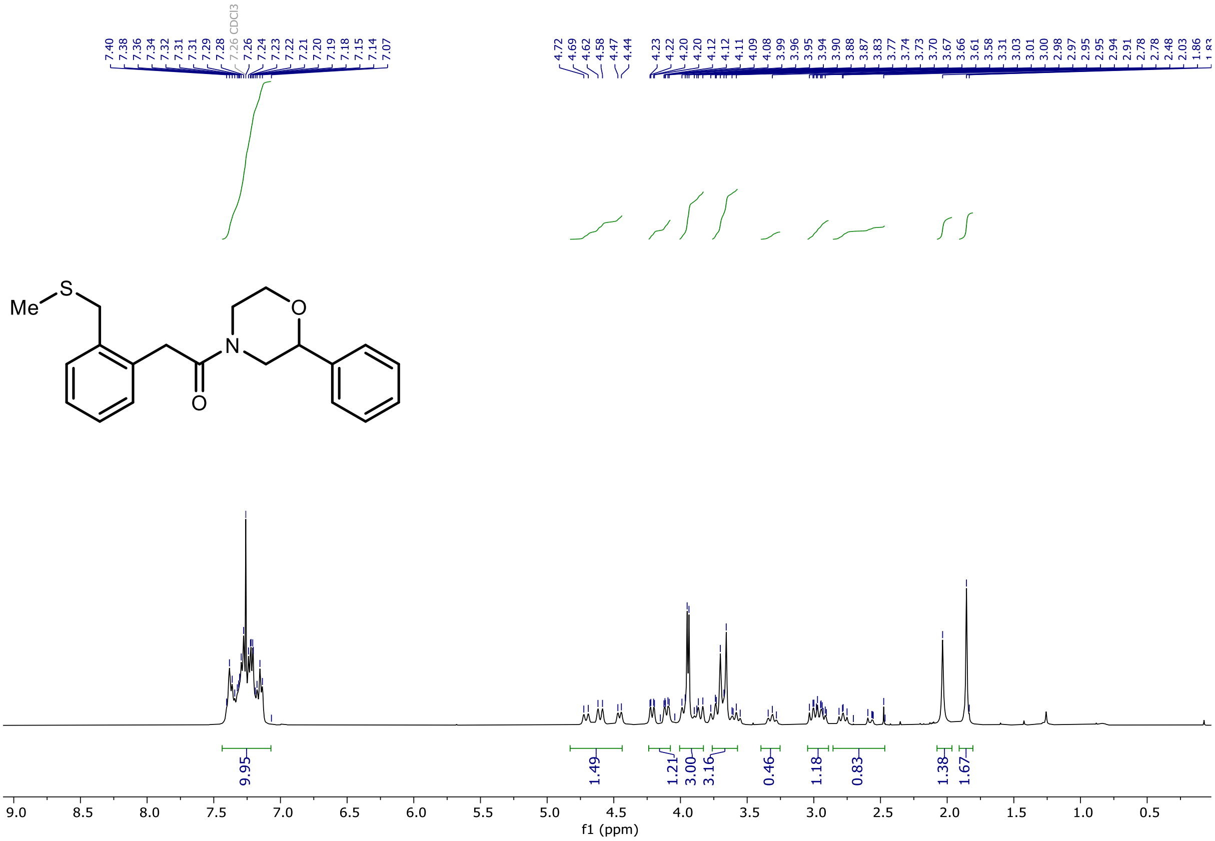

3o – ^{13}C NMR (101 MHz, $CDCl_3$, mixture of rotamers)

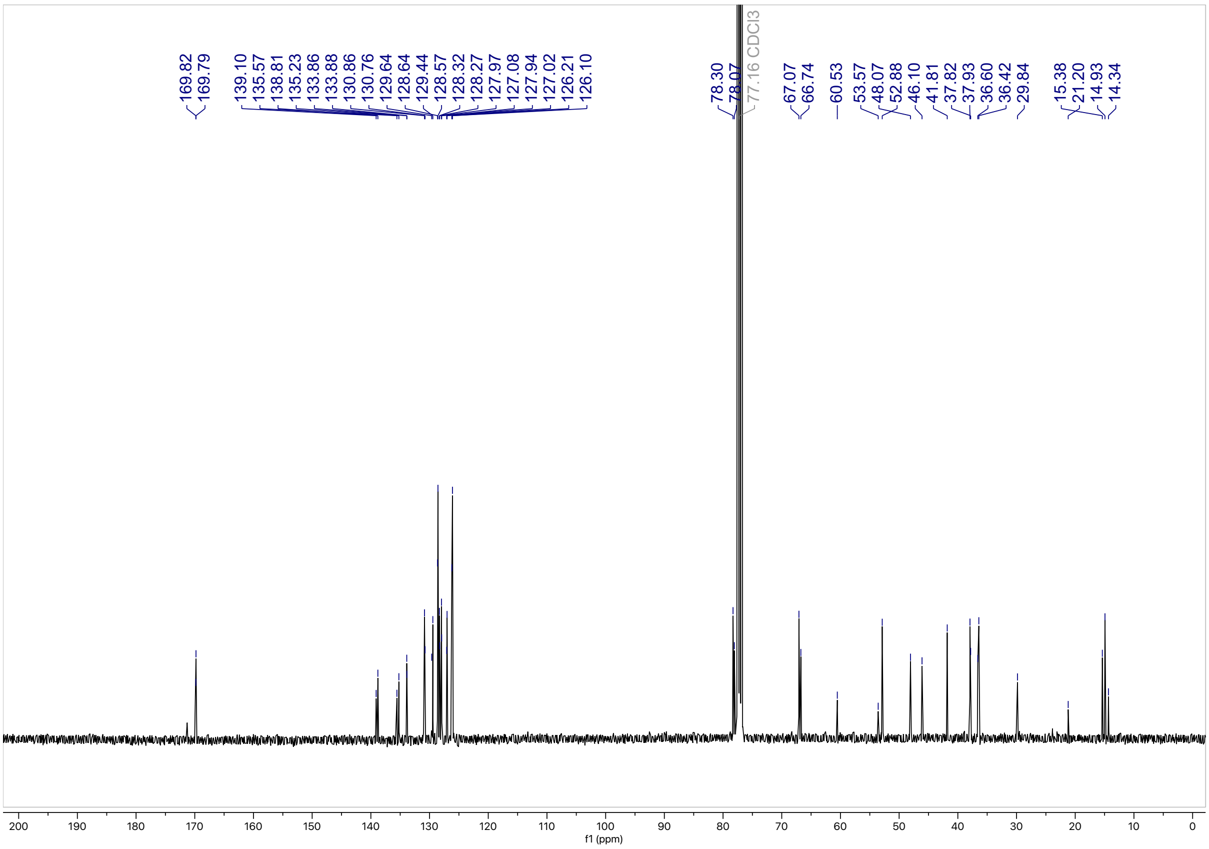

2p – ^{1}H NMR (400 MHz, CD_3CN)

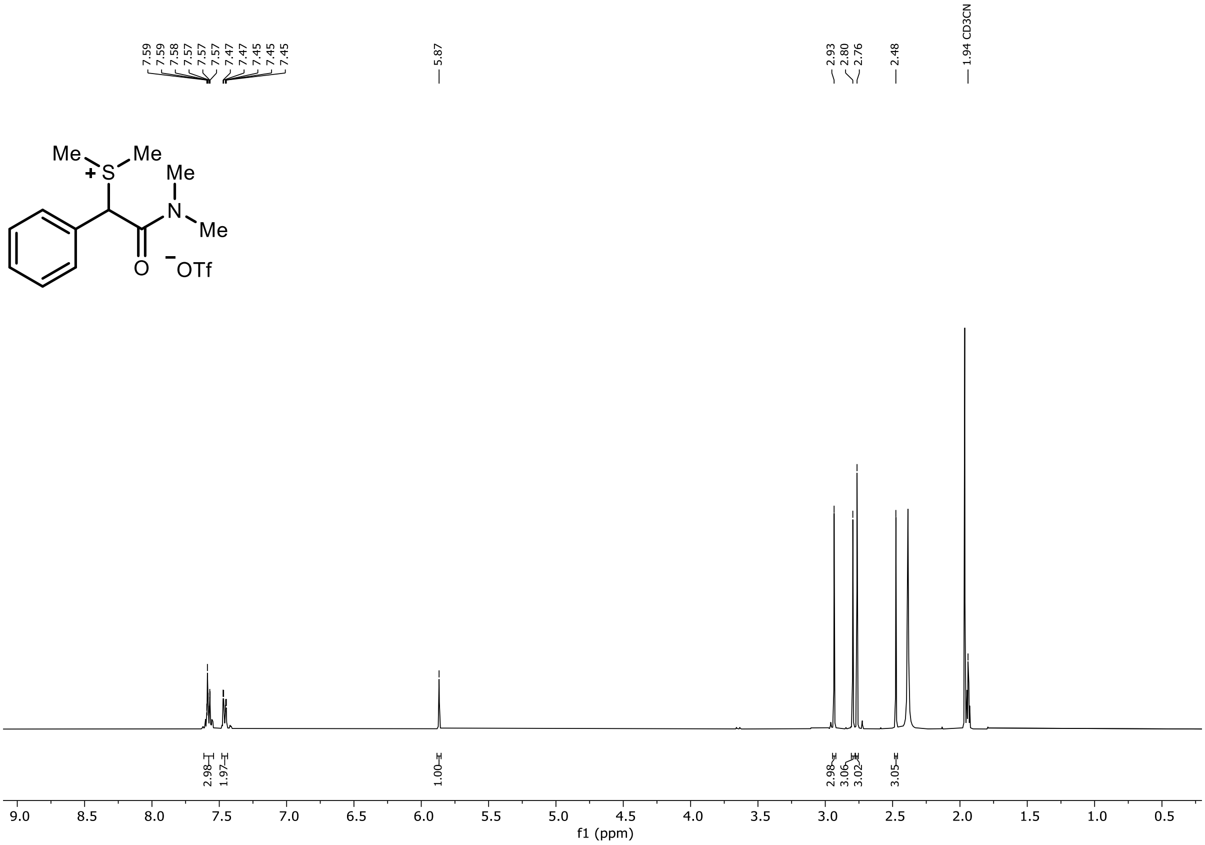

2p – ^{13}C NMR (101 MHz, CD_3CN)

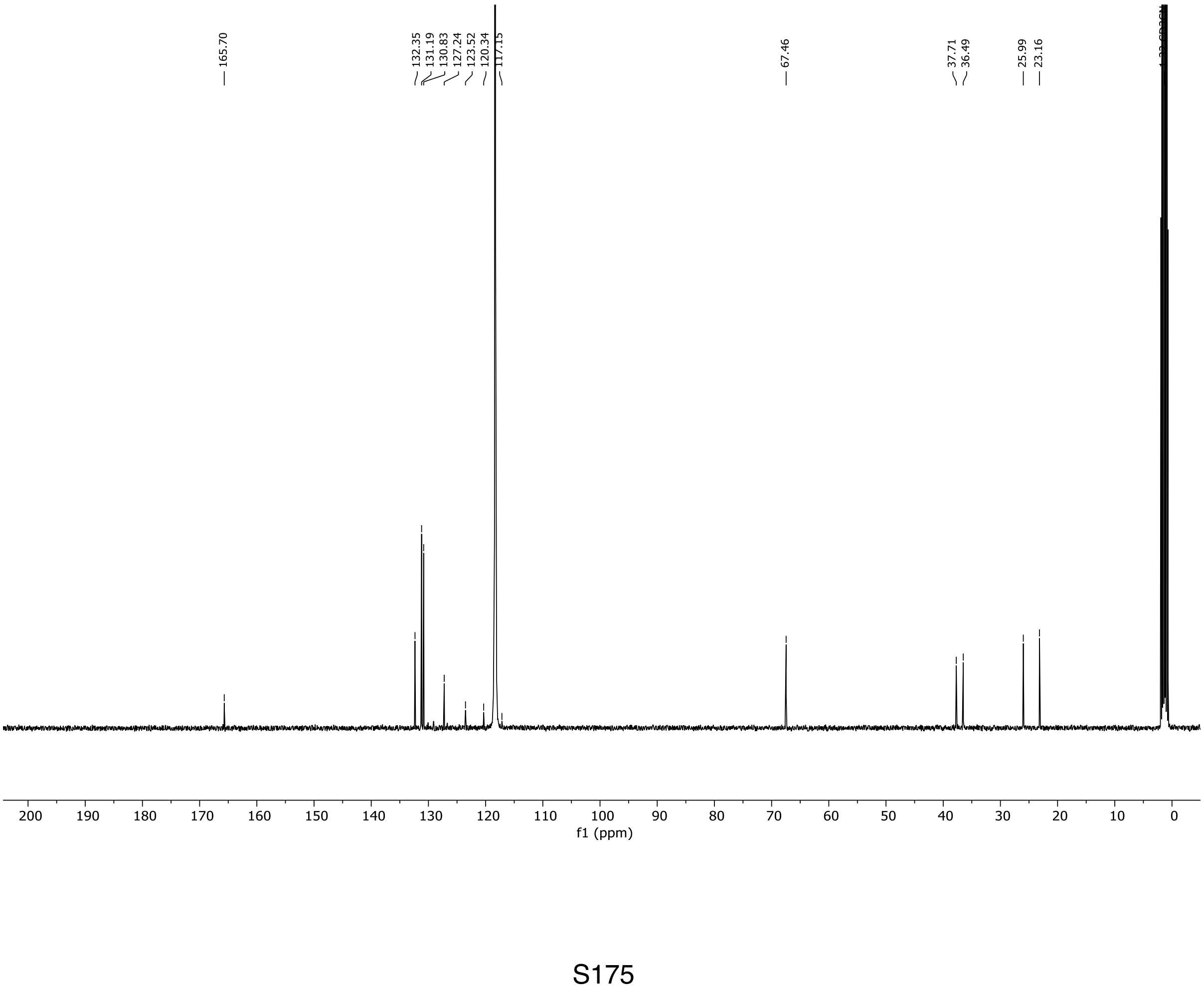

2p – ^{19}F NMR (376 MHz, $CDCl_3$)

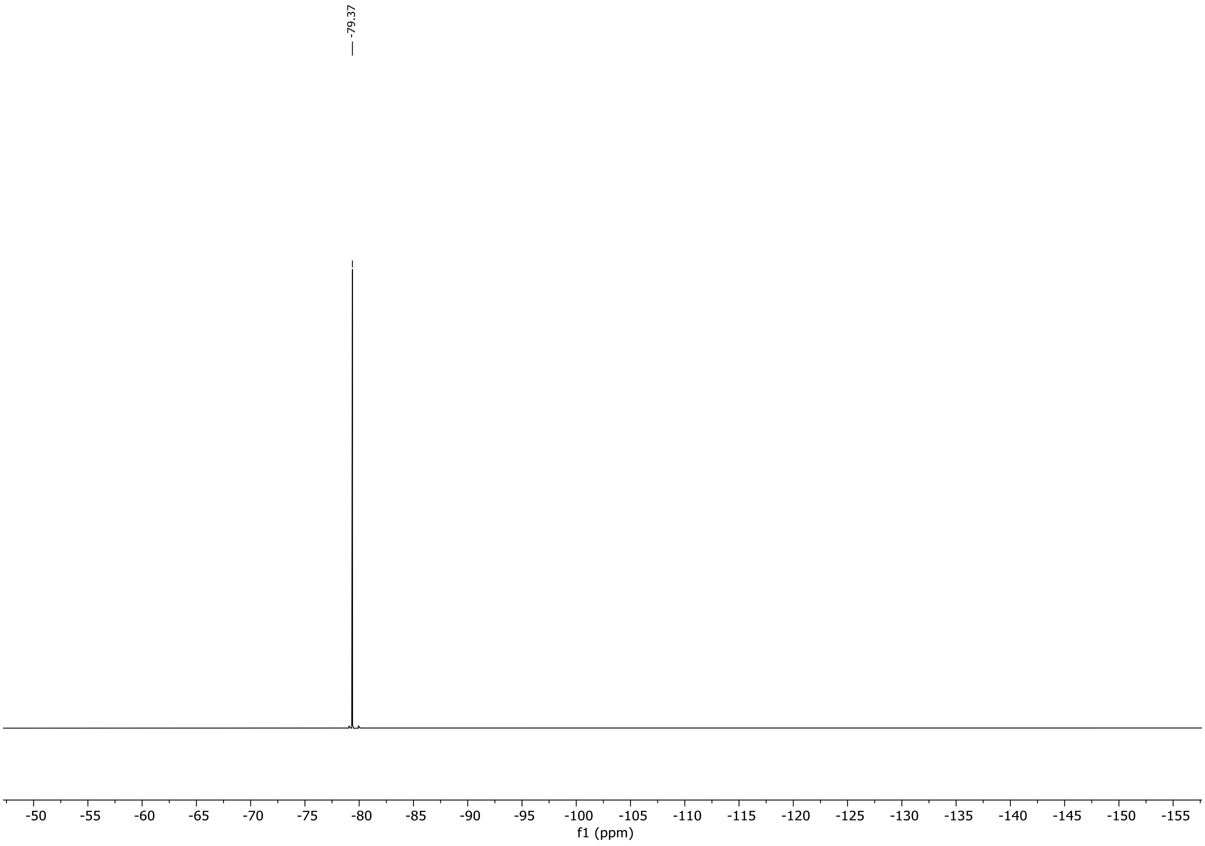

3p – ^{1}H NMR (400 MHz, $CDCl_3$)

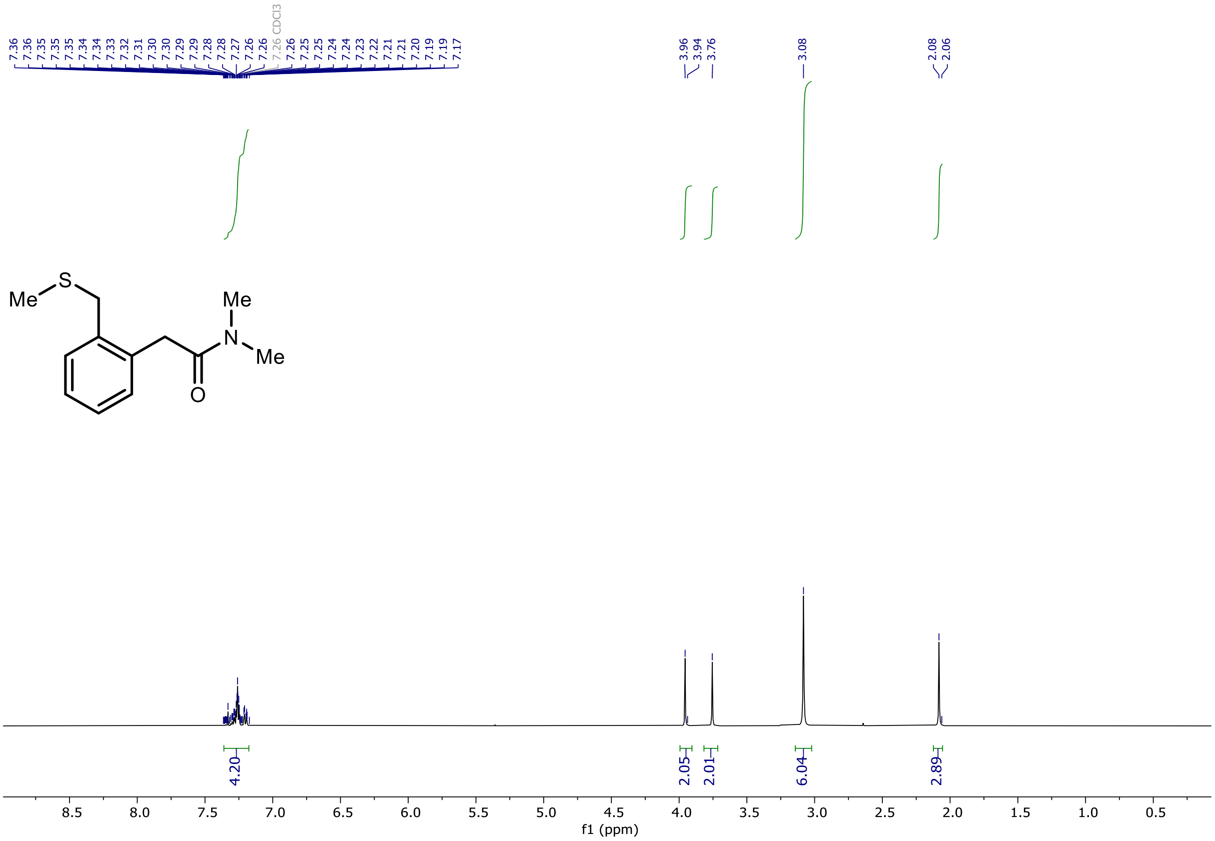

3p – ^{13}C NMR (101 MHz, $CDCl_3$)

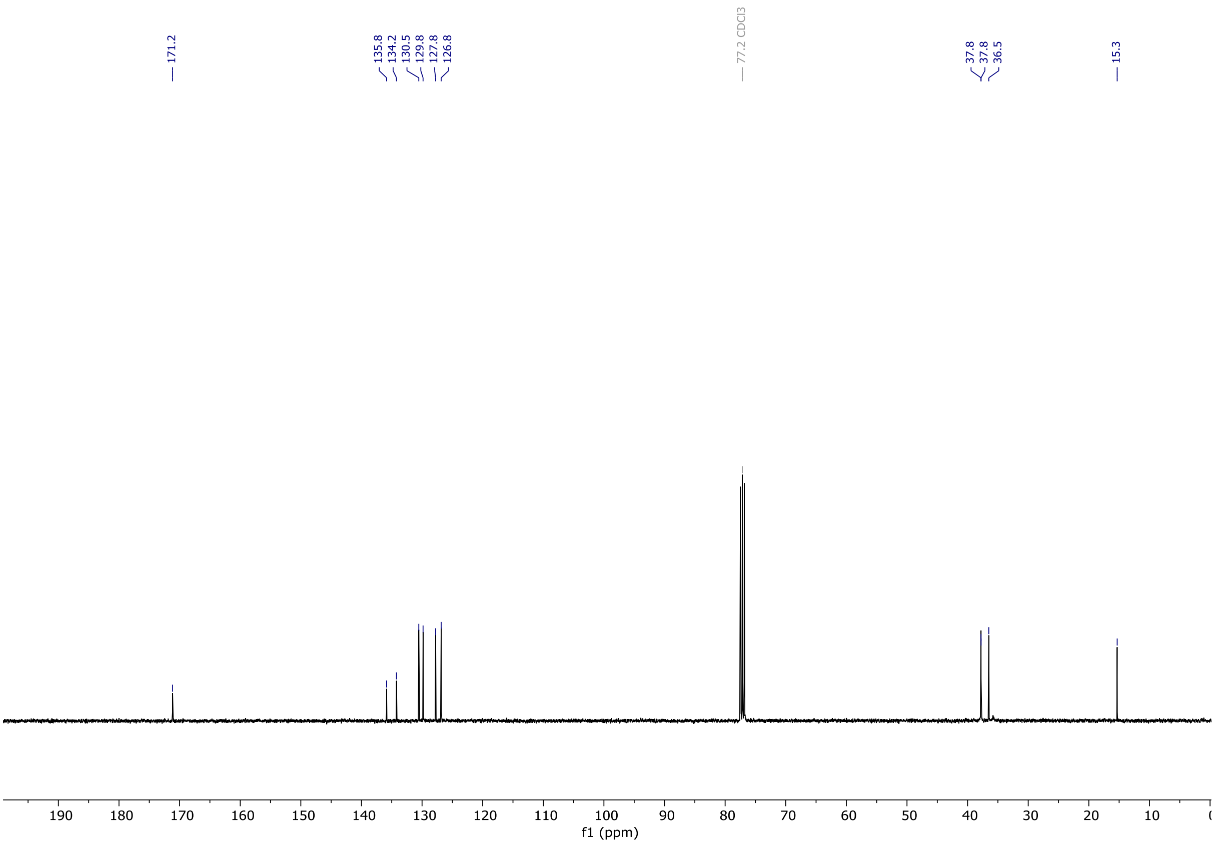

2q – ^{1}H NMR (400 MHz, $CDCl_3$)

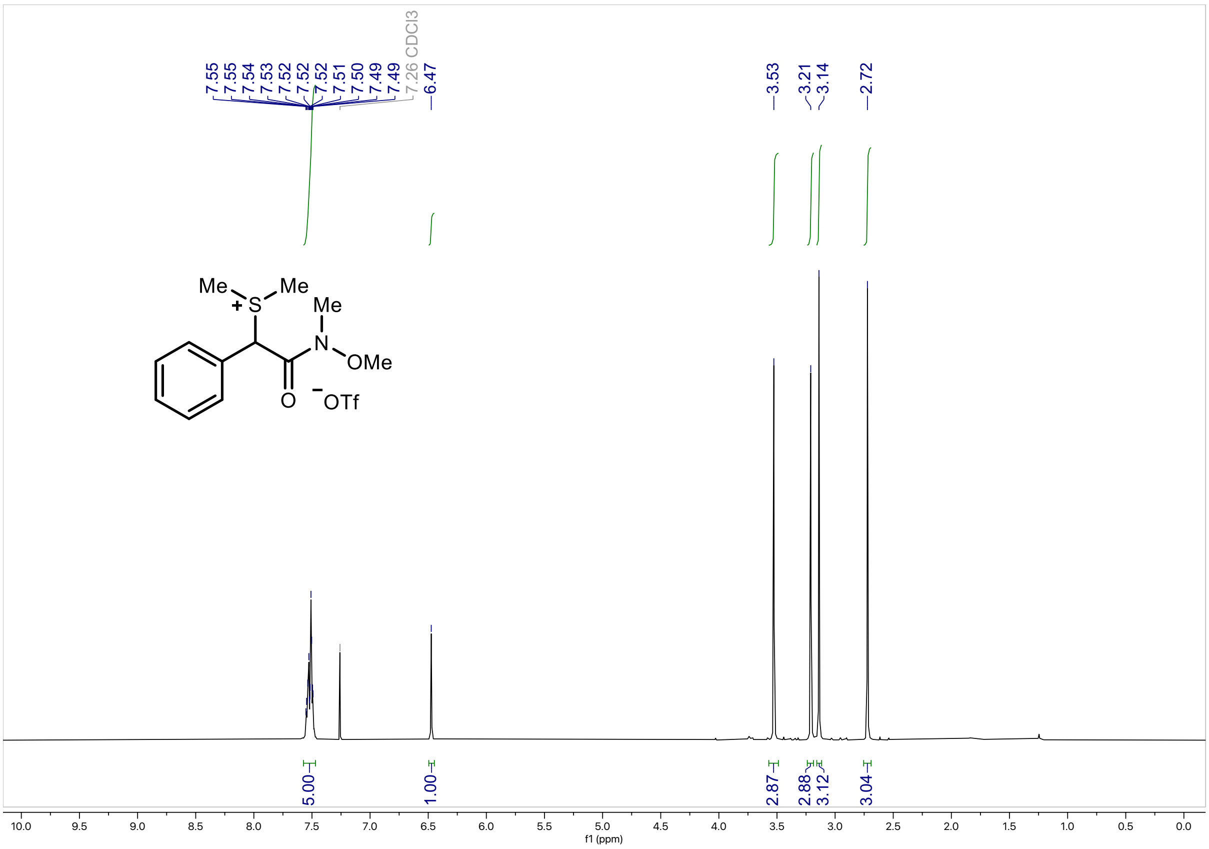

2q – ^{13}C NMR (101 MHz, $CDCl_3$)

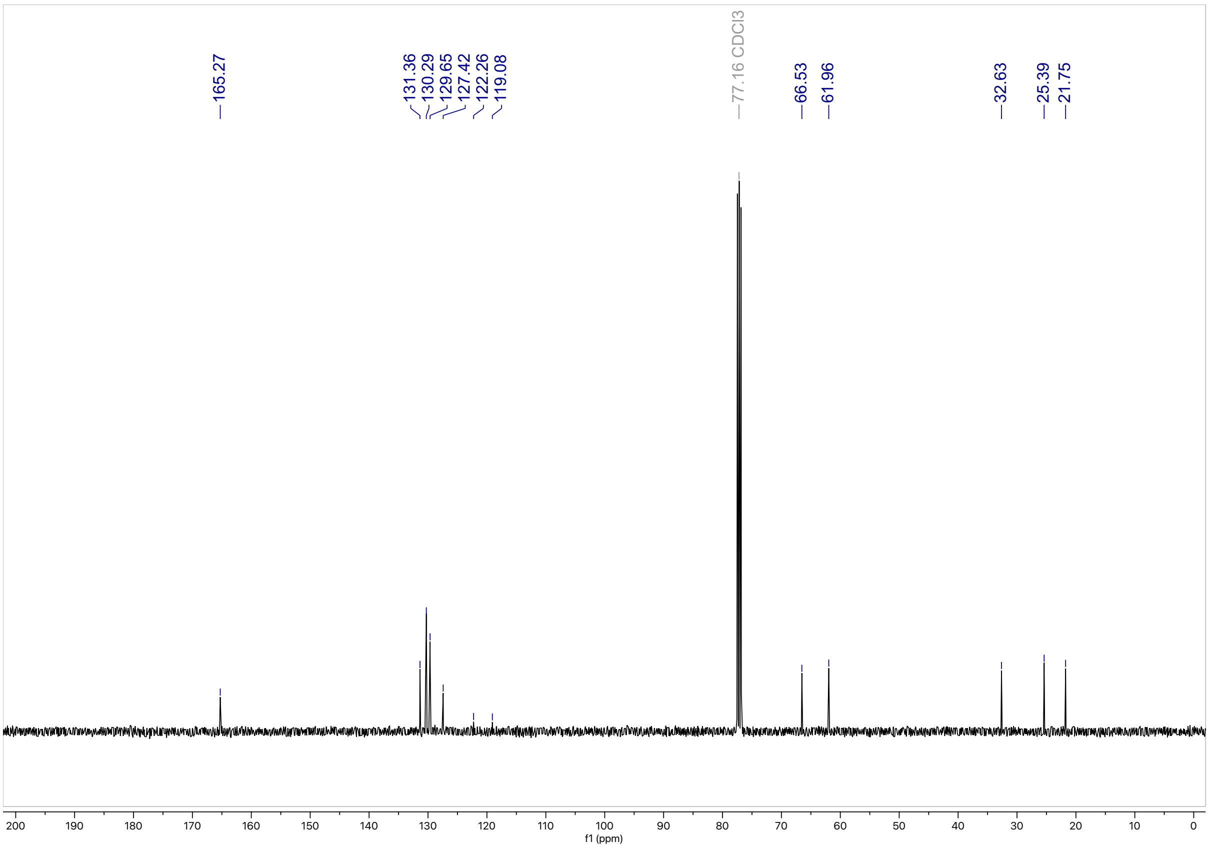

2q – ^{19}F NMR (376 MHz, $CDCl_3$)

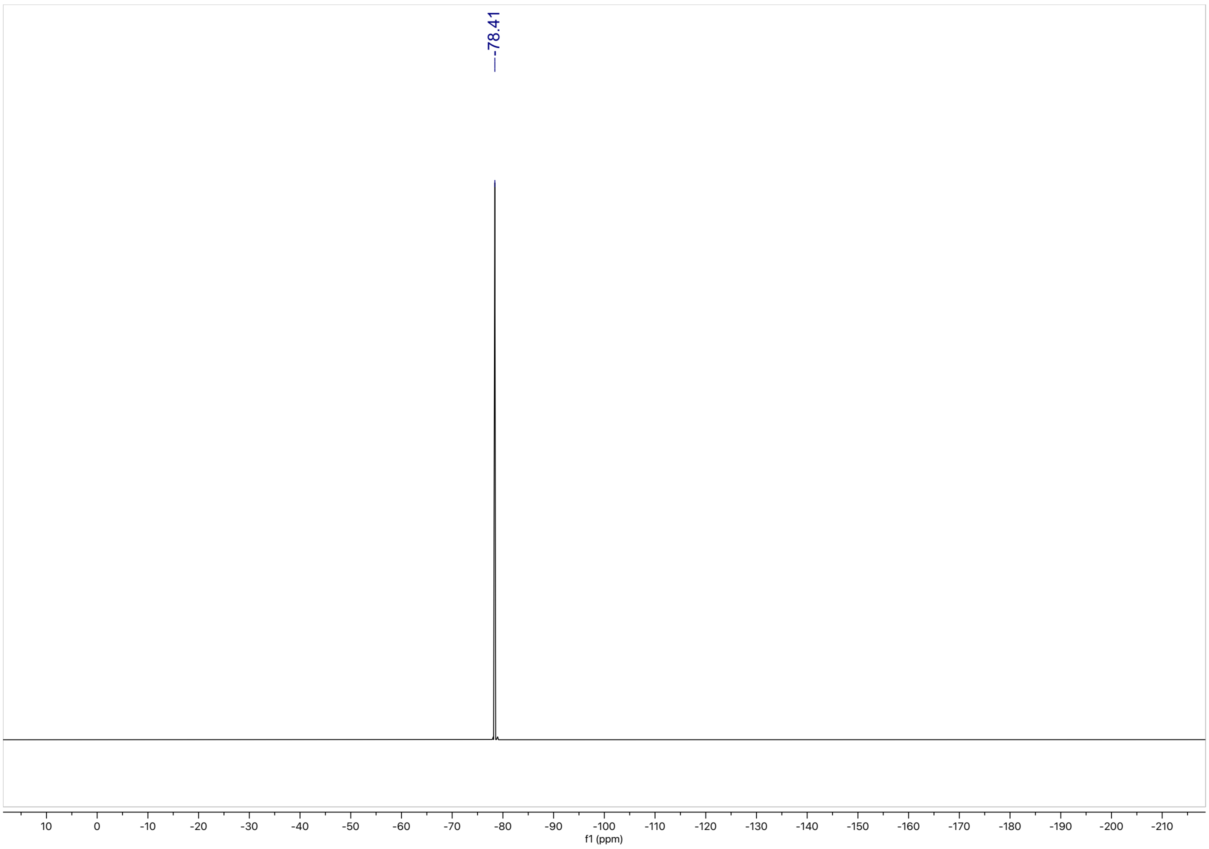

3q – ^{1}H NMR (500 MHz, CDCl$_3$)

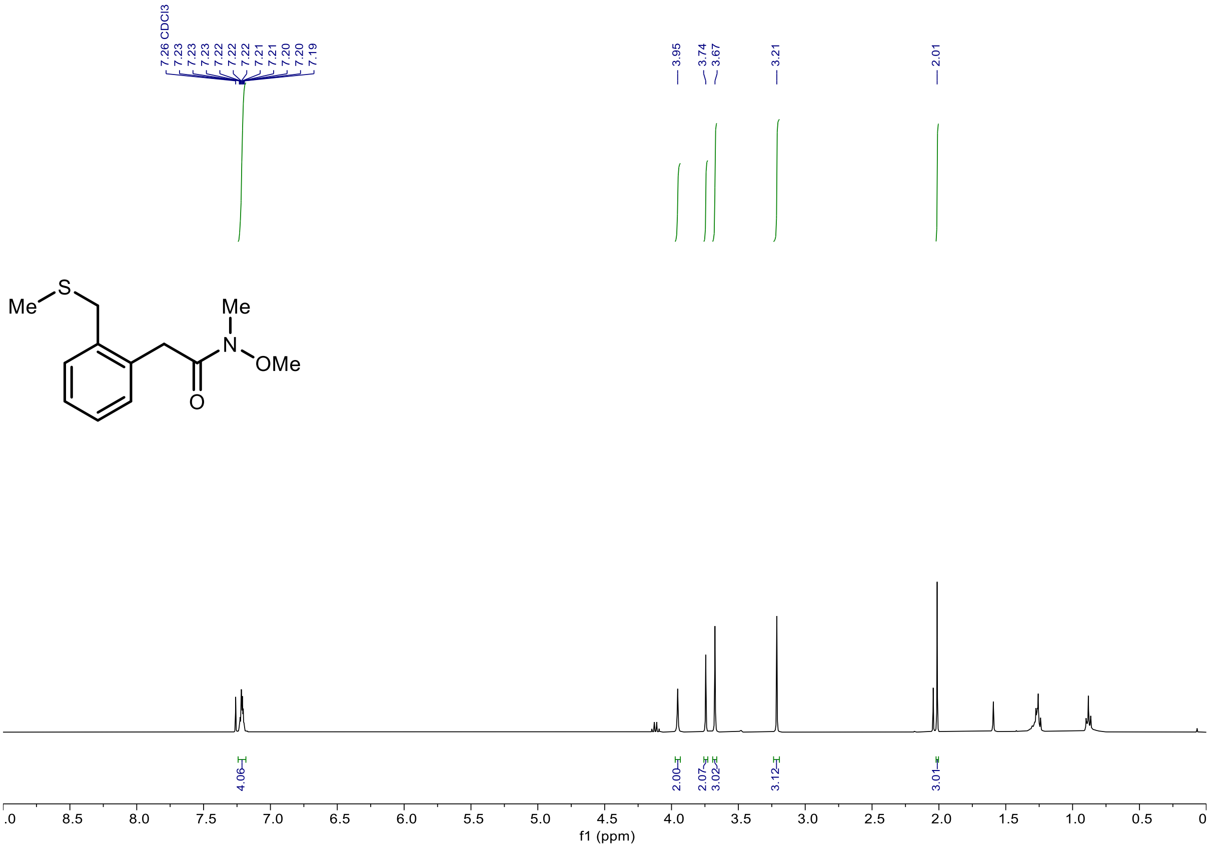

3q – ^{13}C NMR (126 MHz, CDCl$_3$)

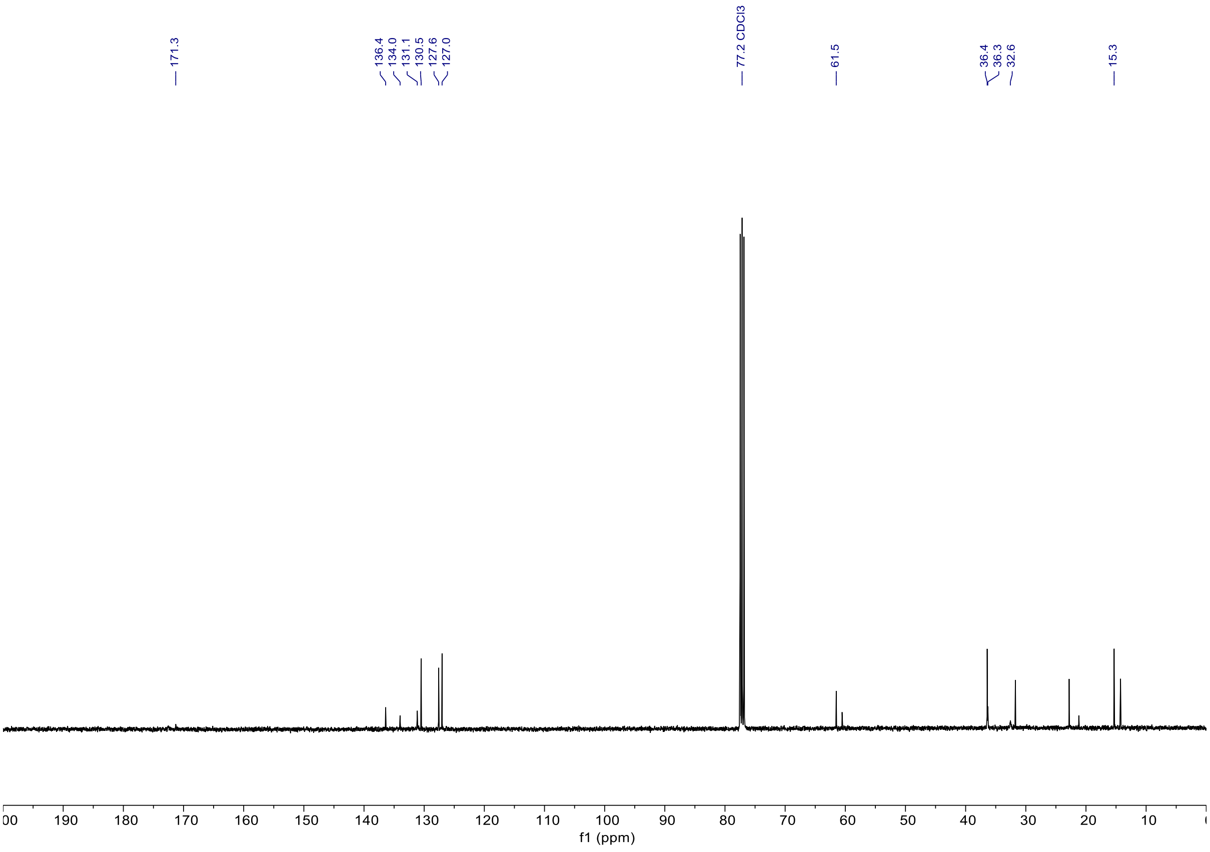

2r – ^{1}H NMR (400 MHz, $CDCl_3$, mixture of rotamers)

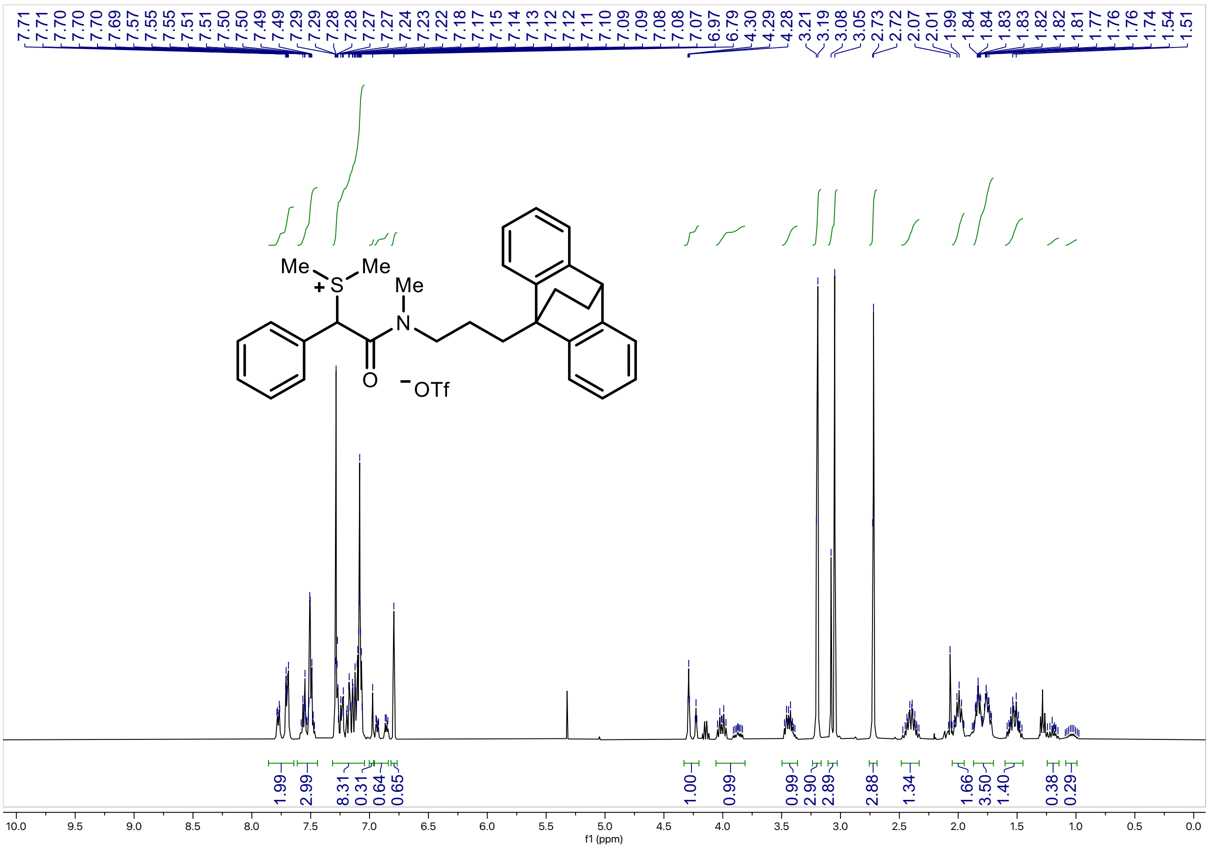

2r – ^{13}C NMR (101 MHz, $CDCl_3$, mixture of rotamers)

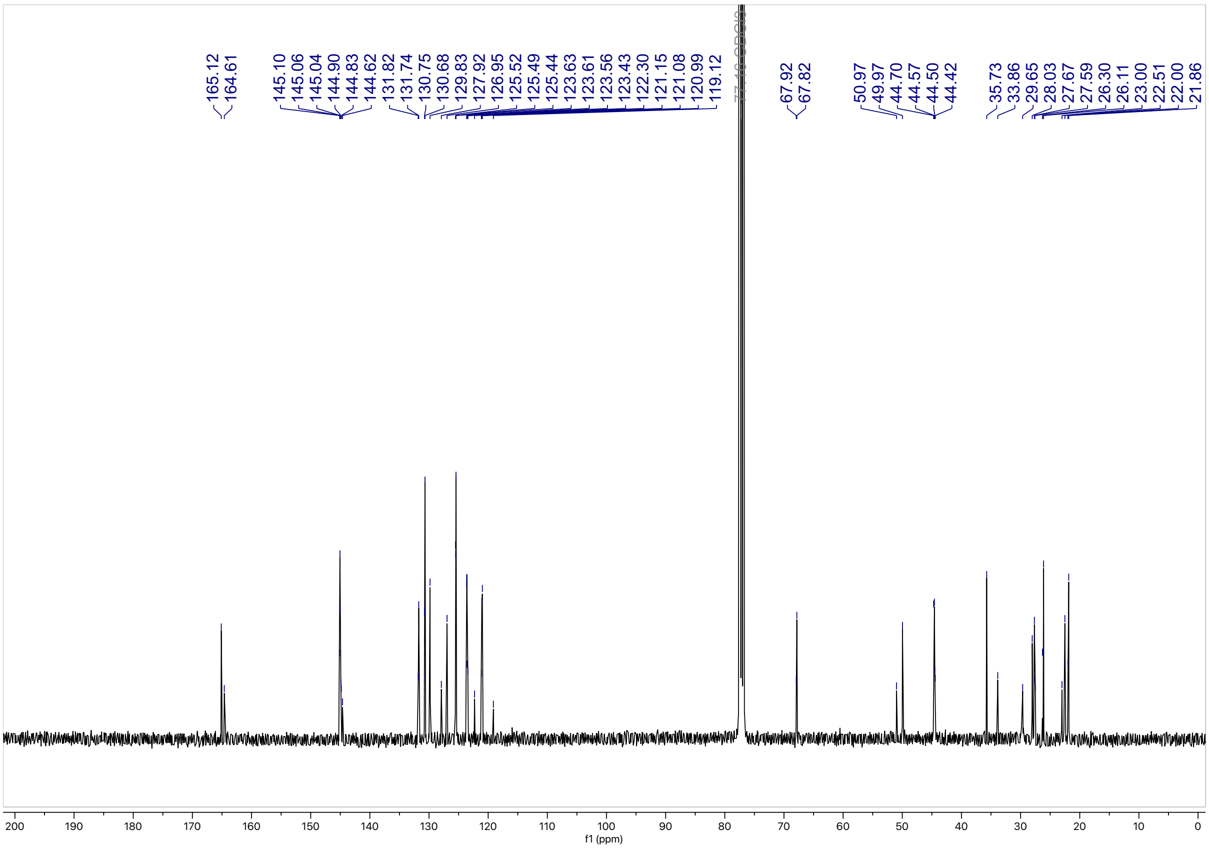

2r – ^{19}F NMR (376 MHz, CD_3CN)

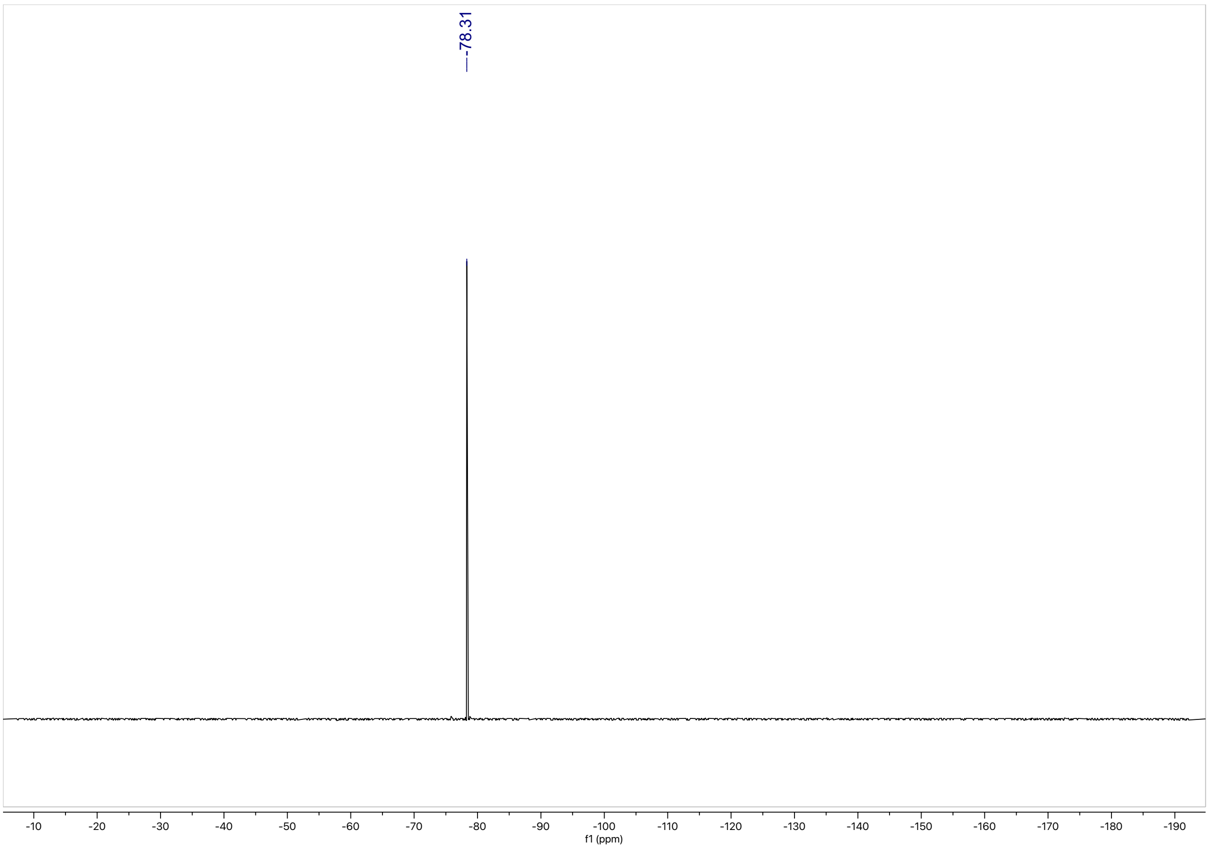

3r – ¹H NMR (400 MHz, $CDCl_3$, mixture of rotamers)

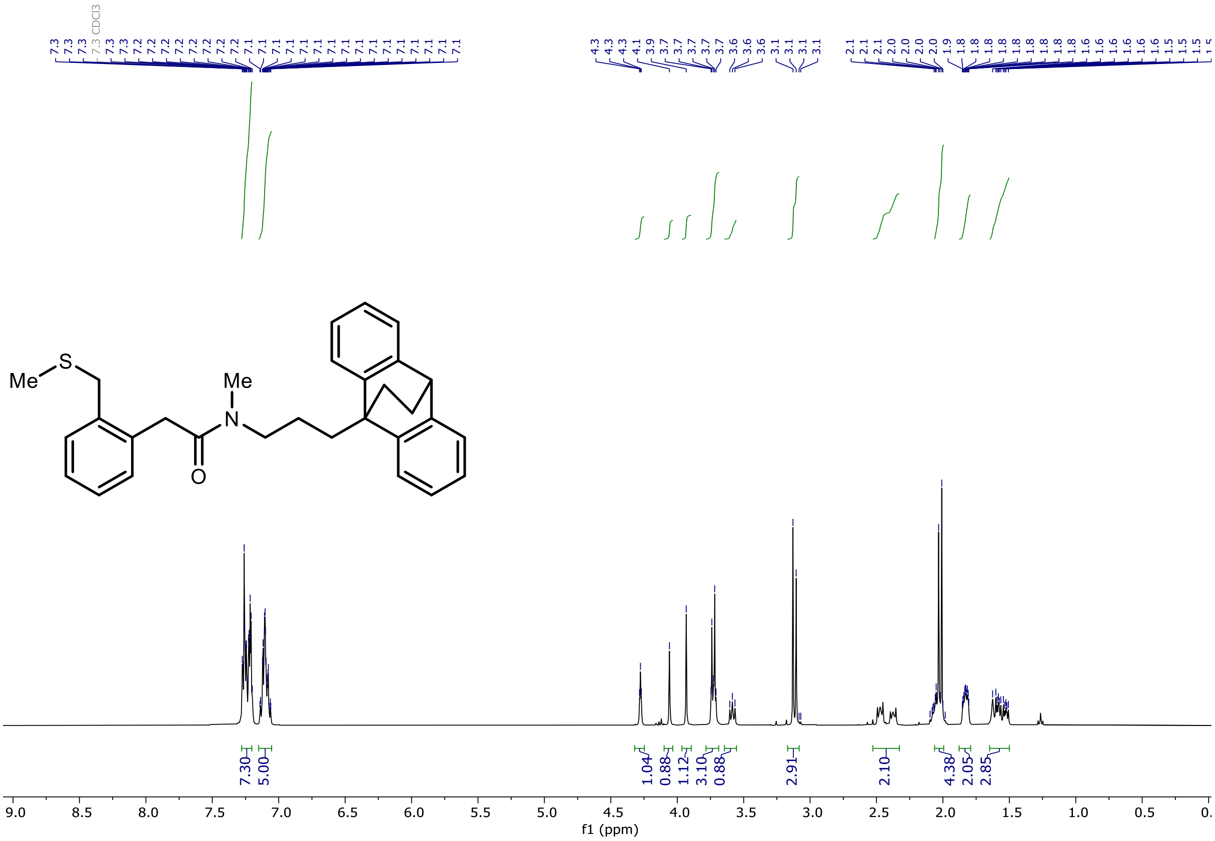

3r – ¹³C NMR (101 MHz, $CDCl_3$, mixture of rotamers)

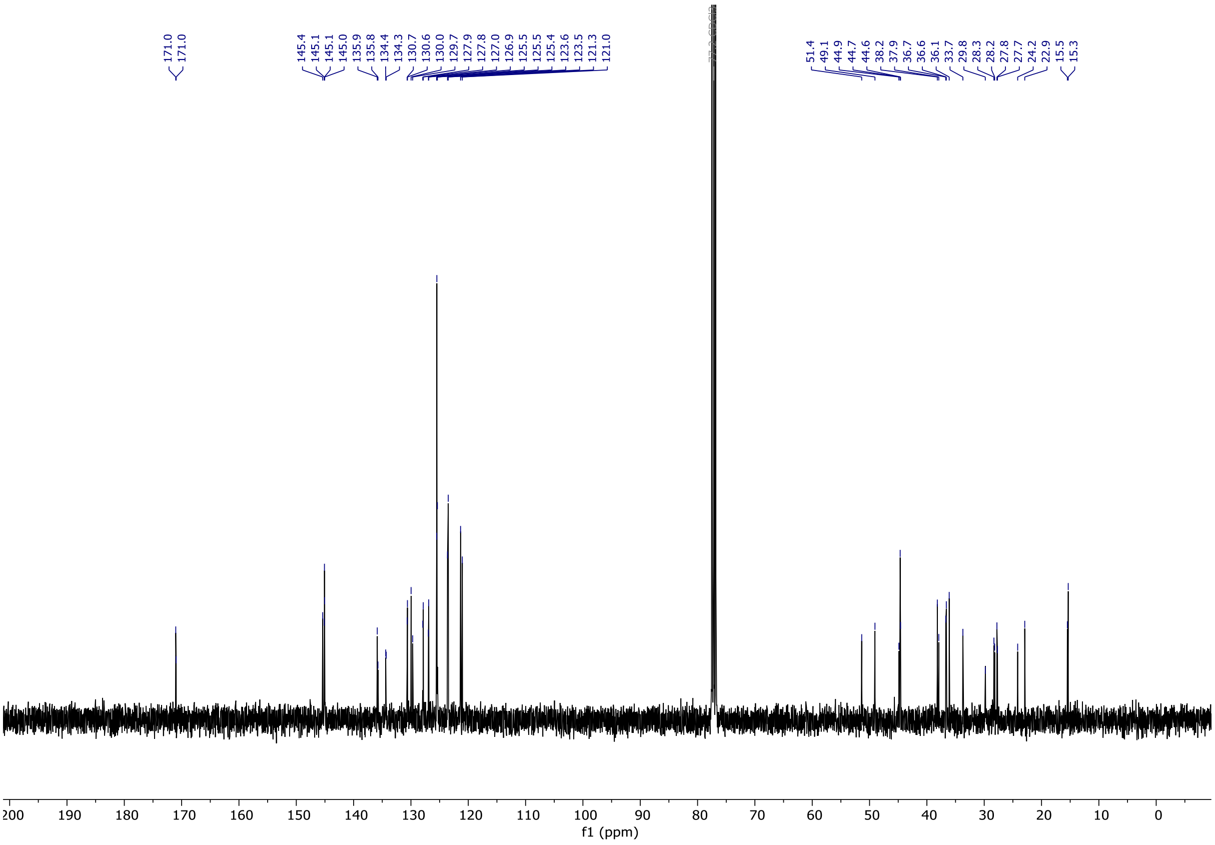

2s – ^{1}H NMR (400 MHz, $CDCl_3$)

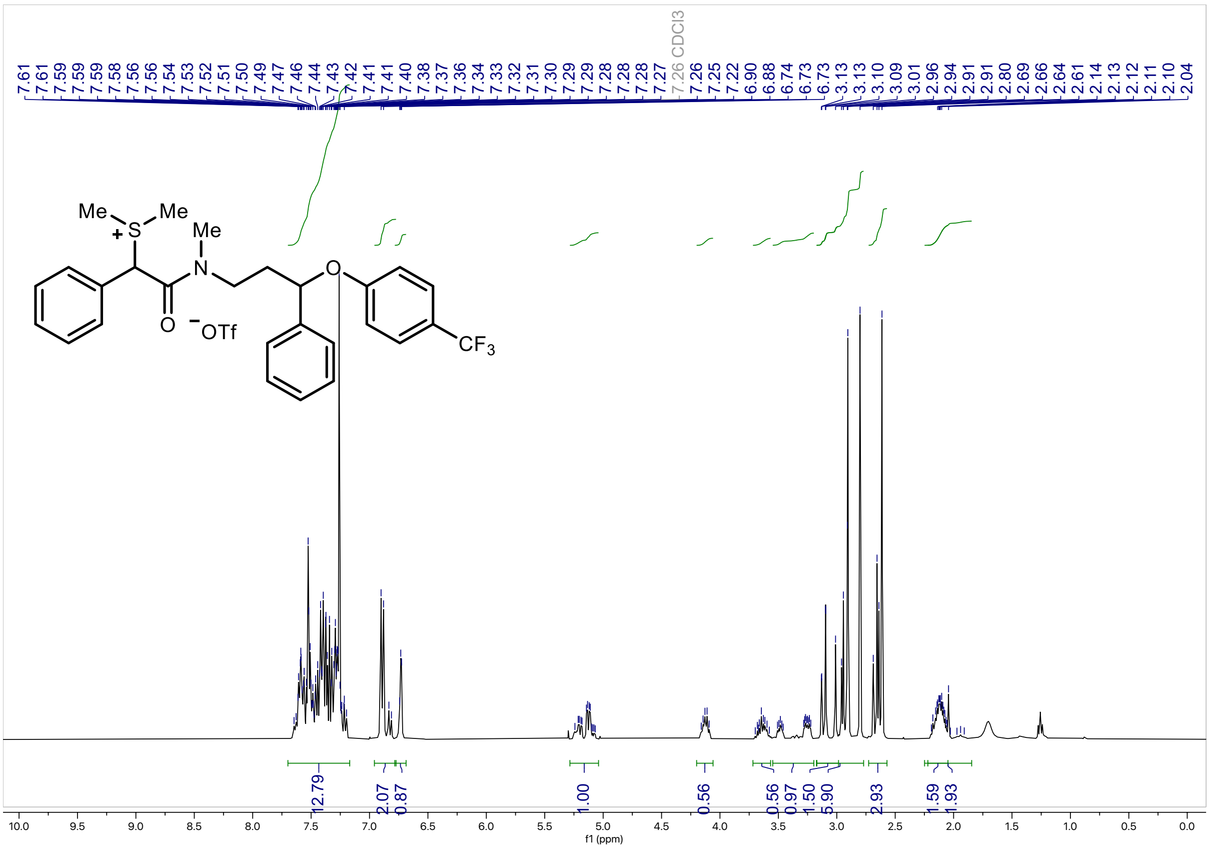

2s – ^{13}C NMR (101 MHz, $CDCl_3$)

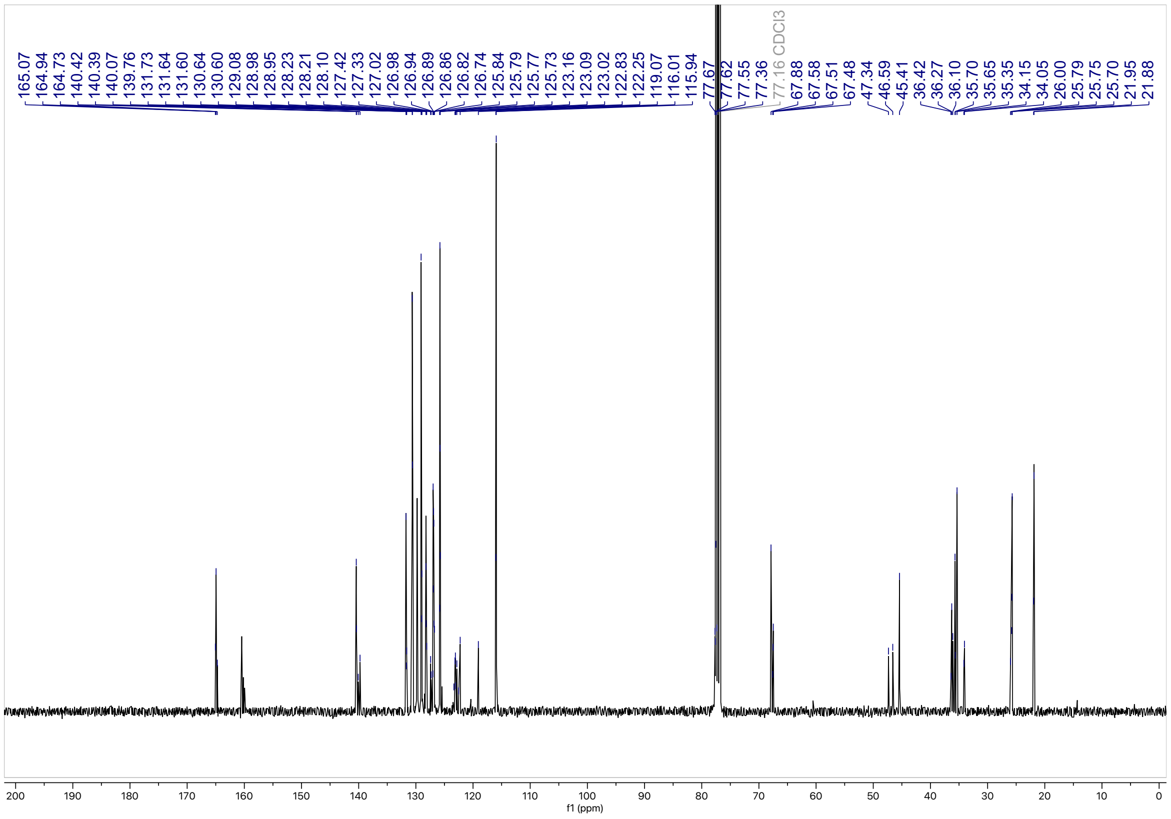

2s – ^{19}F NMR (376 MHz, CD_3CN)

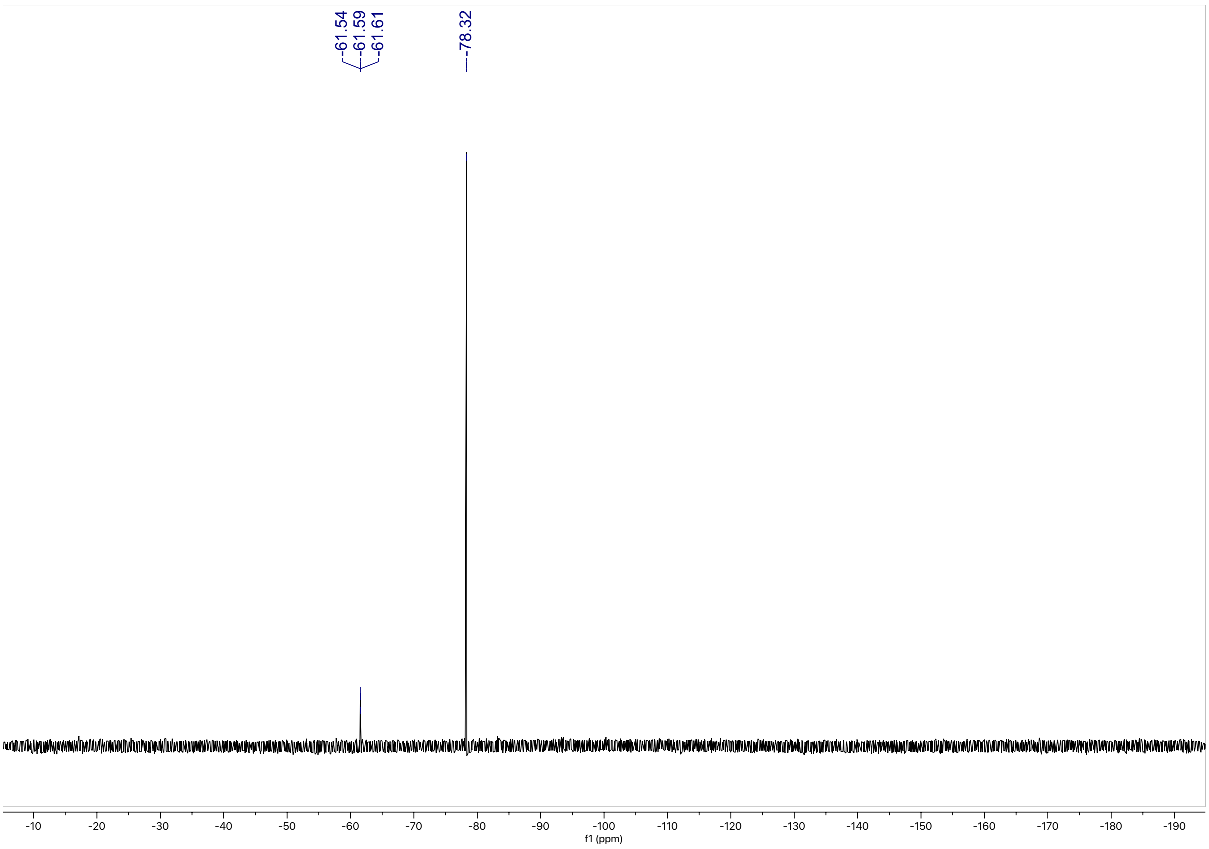

3s – ^{1}H NMR (500 MHz, $CDCl_3$)

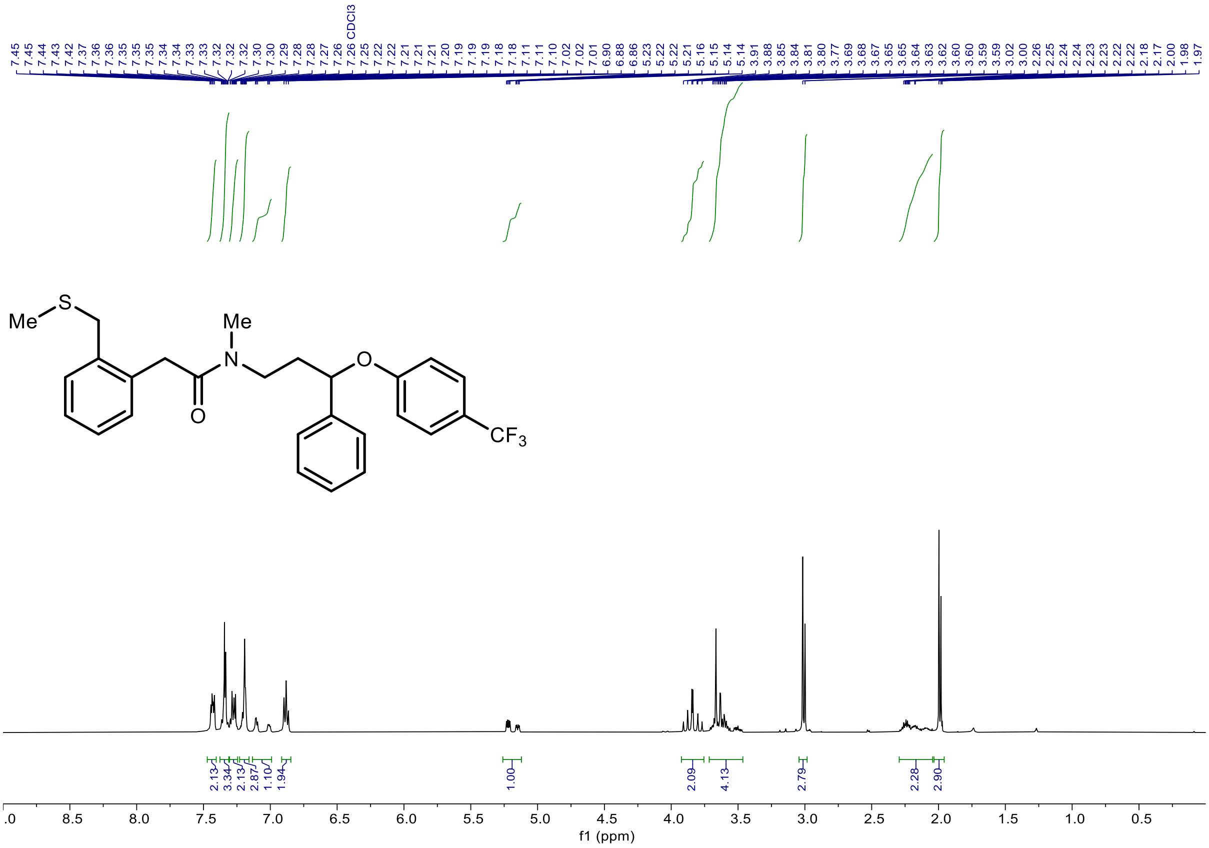

3s – ^{13}C NMR (126 MHz, $CDCl_3$)

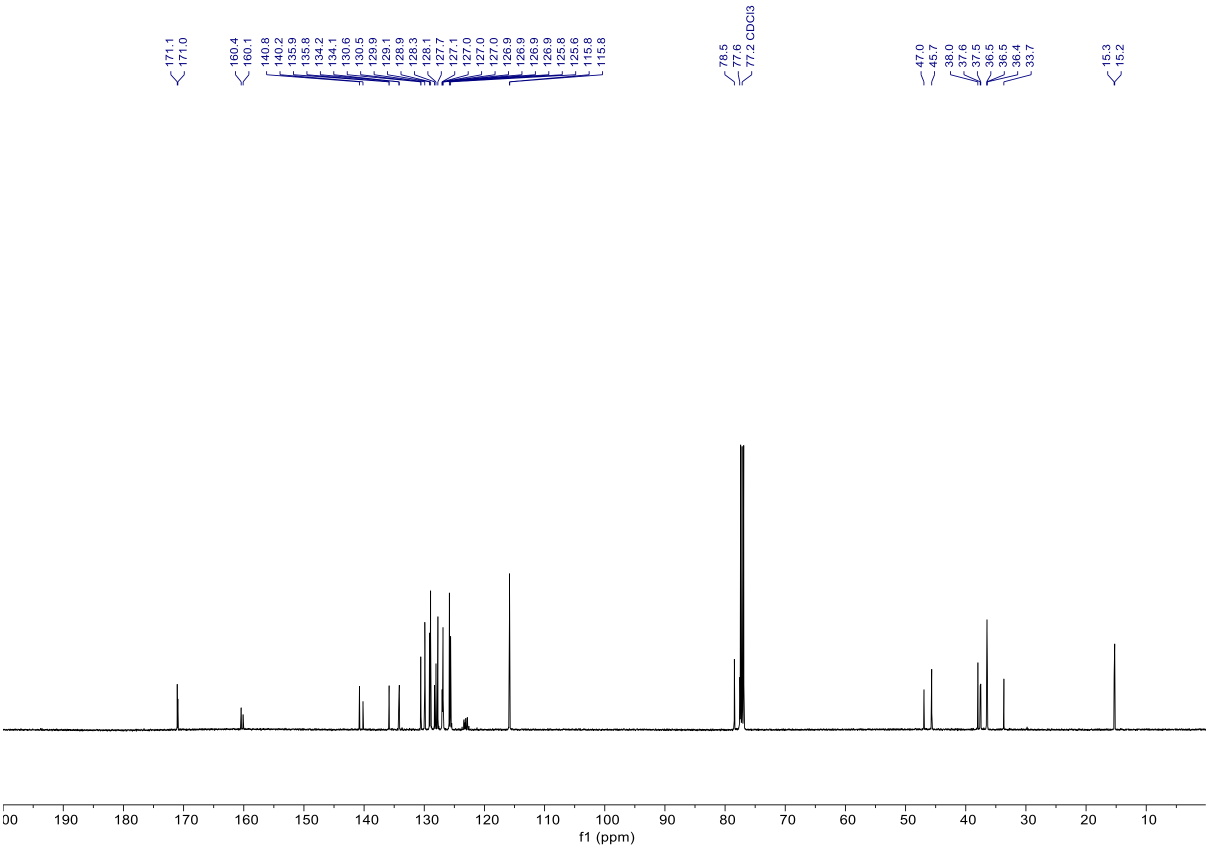

2t – ^{1}H NMR (400 MHz, $CDCl_3$)

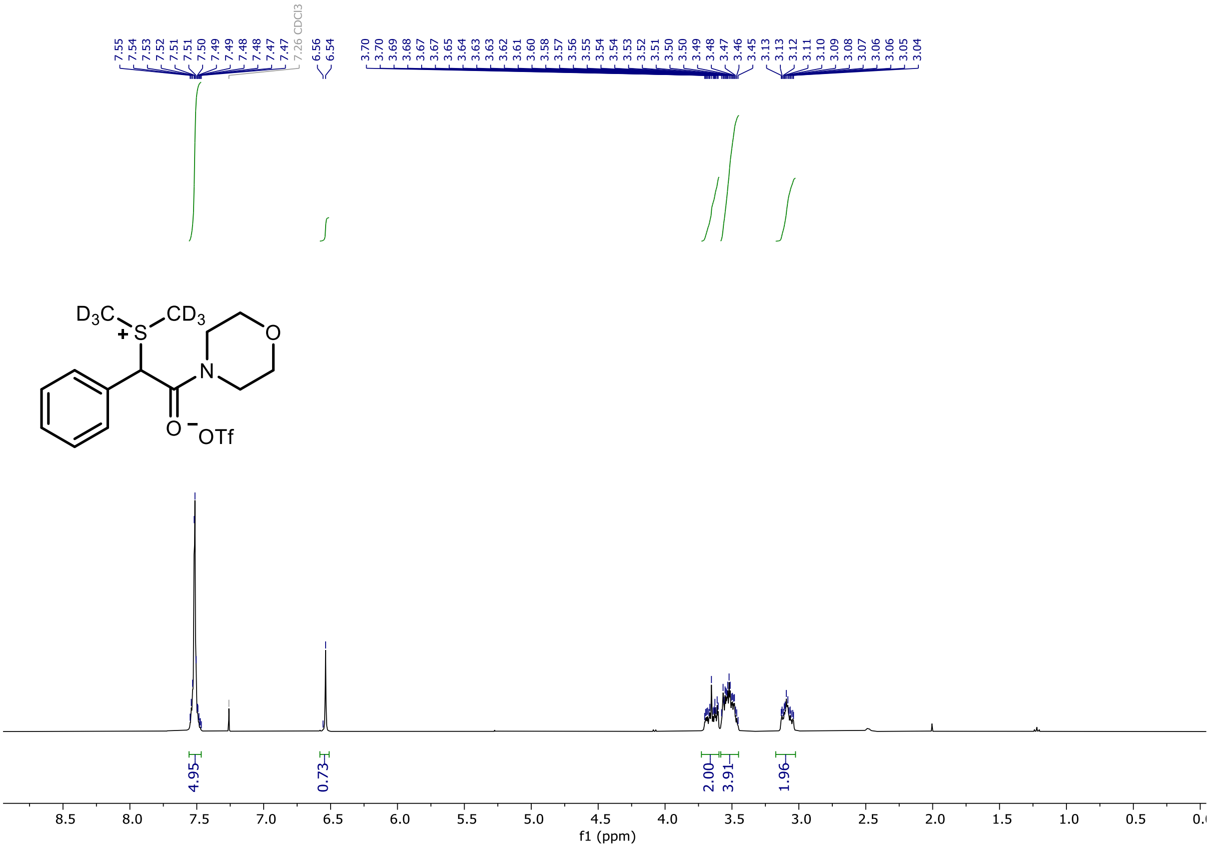

2t – ^{13}C NMR (101 MHz, $CDCl_3$)

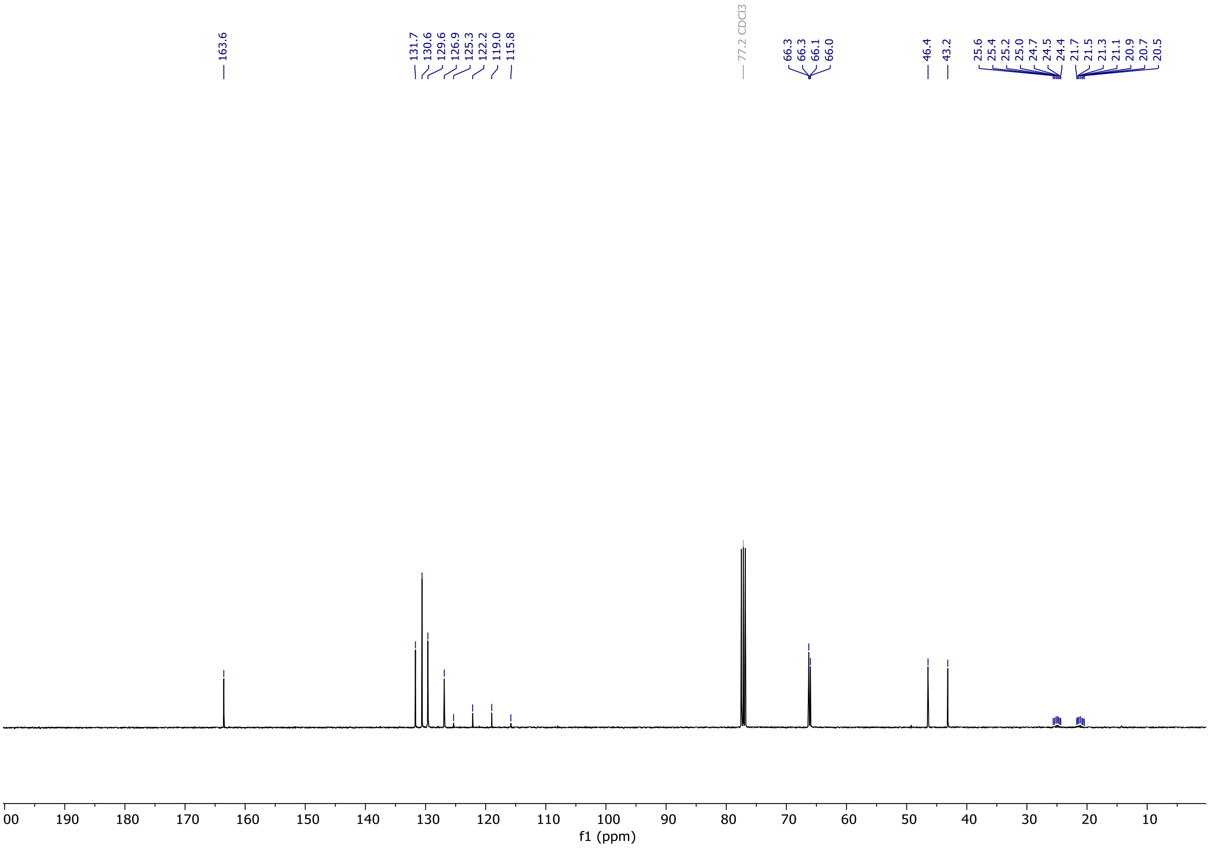

2t – ^{19}F NMR (376 MHz, $CDCl_3$)

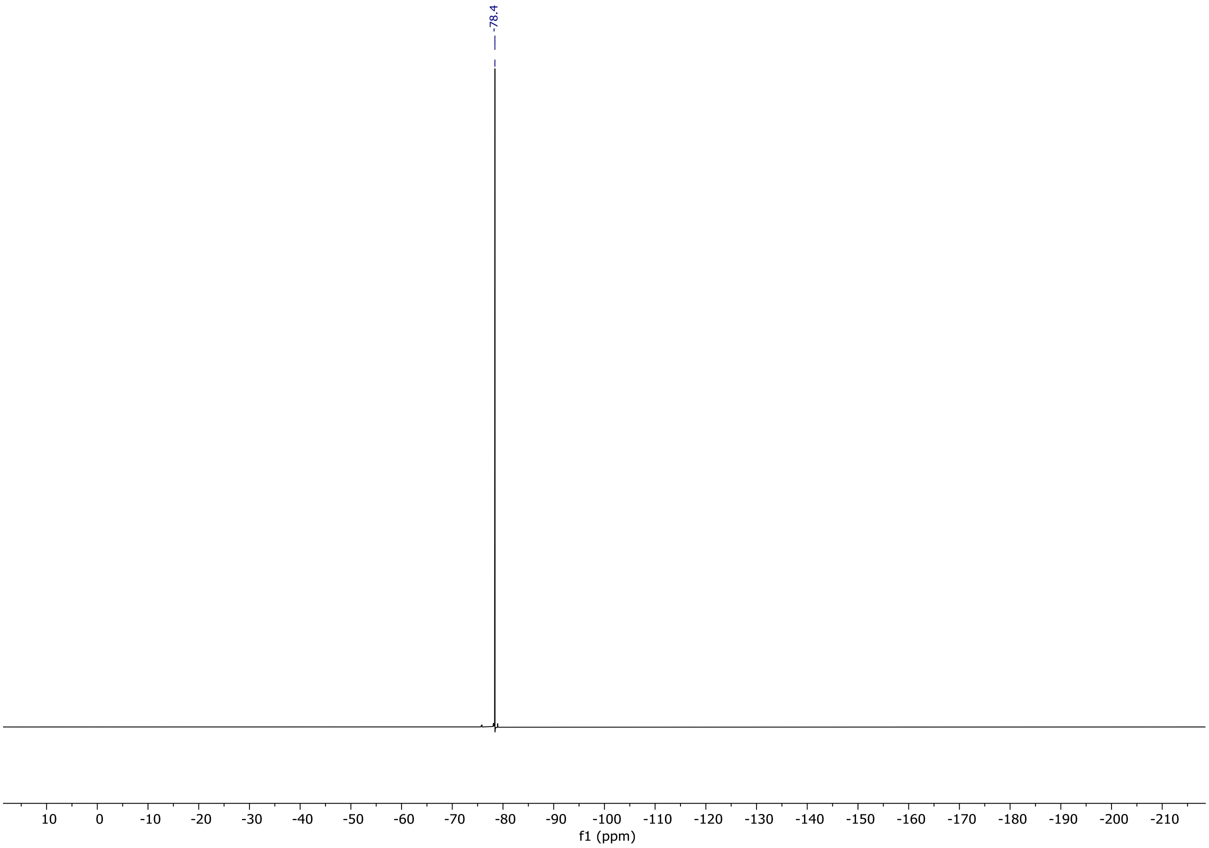

3t – ¹H NMR (400 MHz, $CDCl_3$)

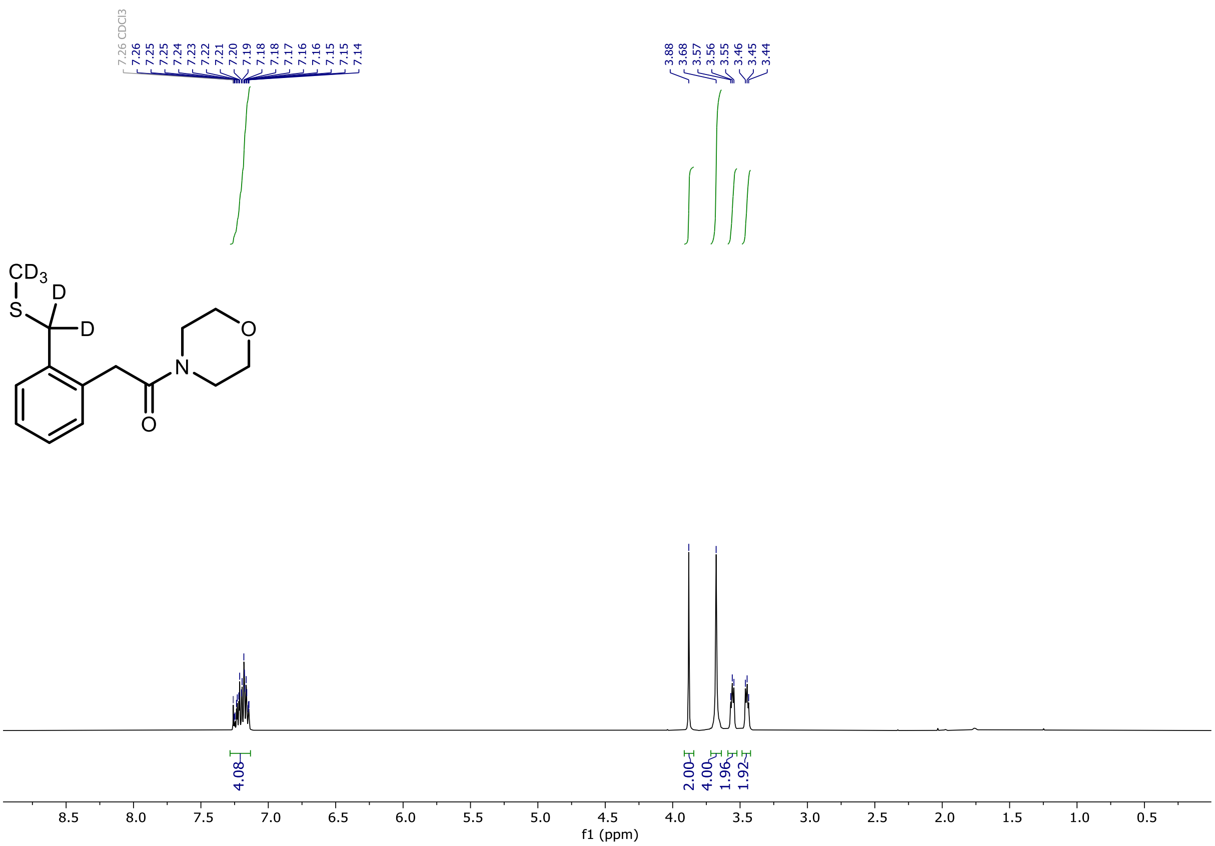

3t – ¹³C NMR (101 MHz, $CDCl_3$)

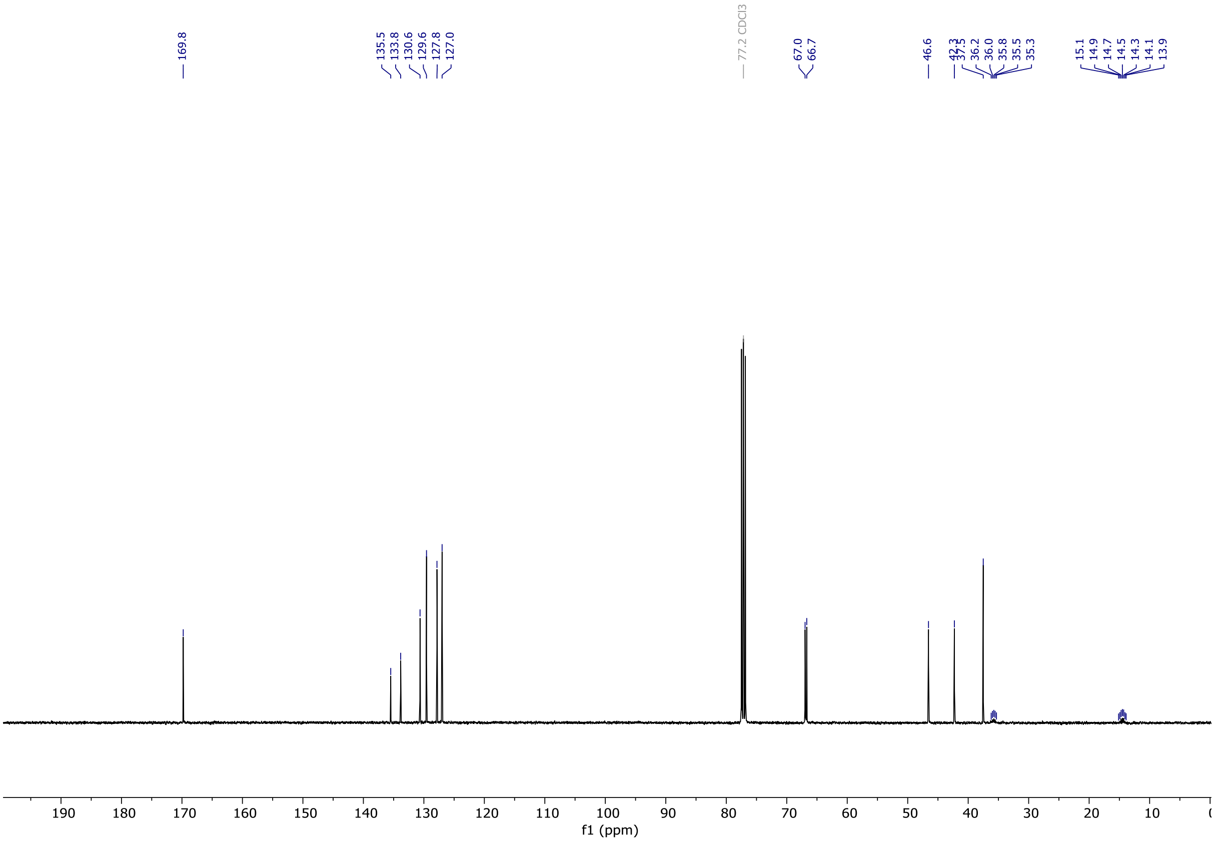

2u – ^{1}H NMR (400 MHz, CD_3CN)

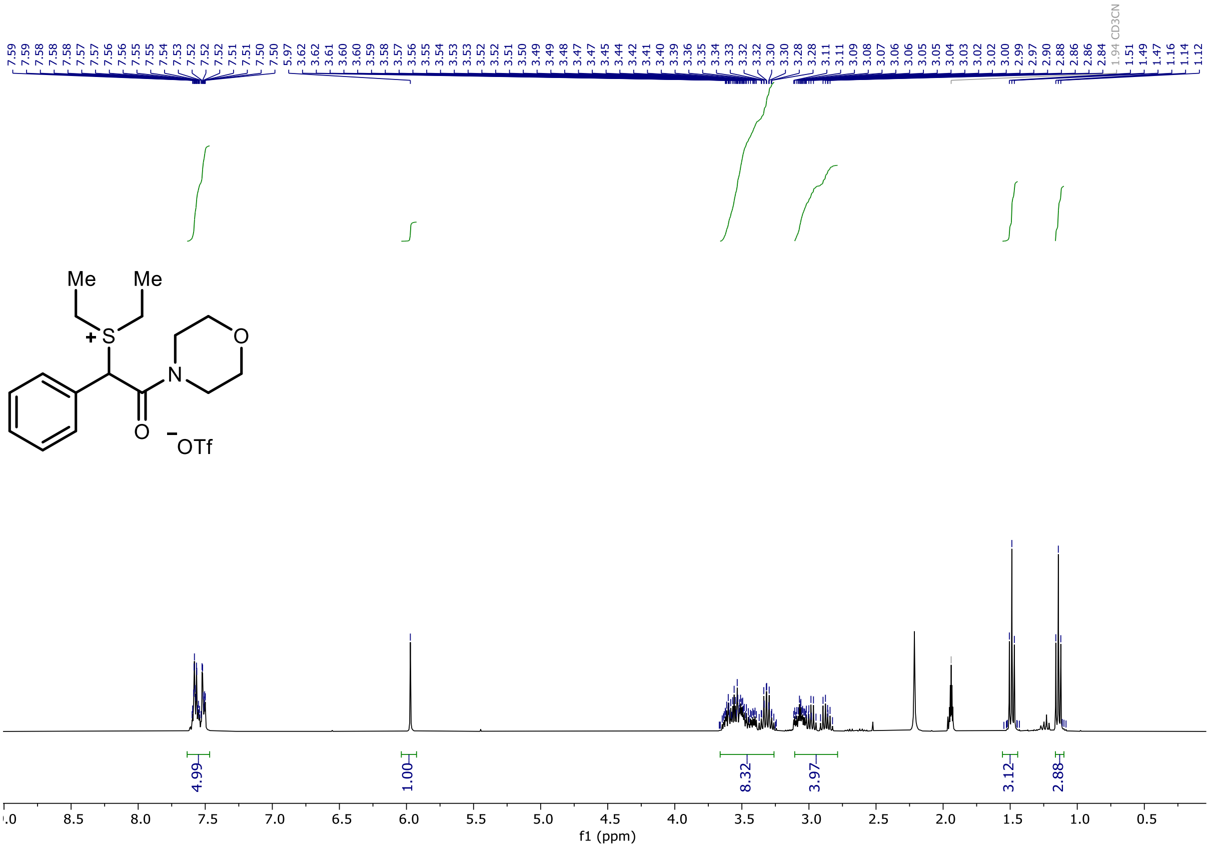

2u – ^{13}C NMR (101 MHz, CD_3CN)

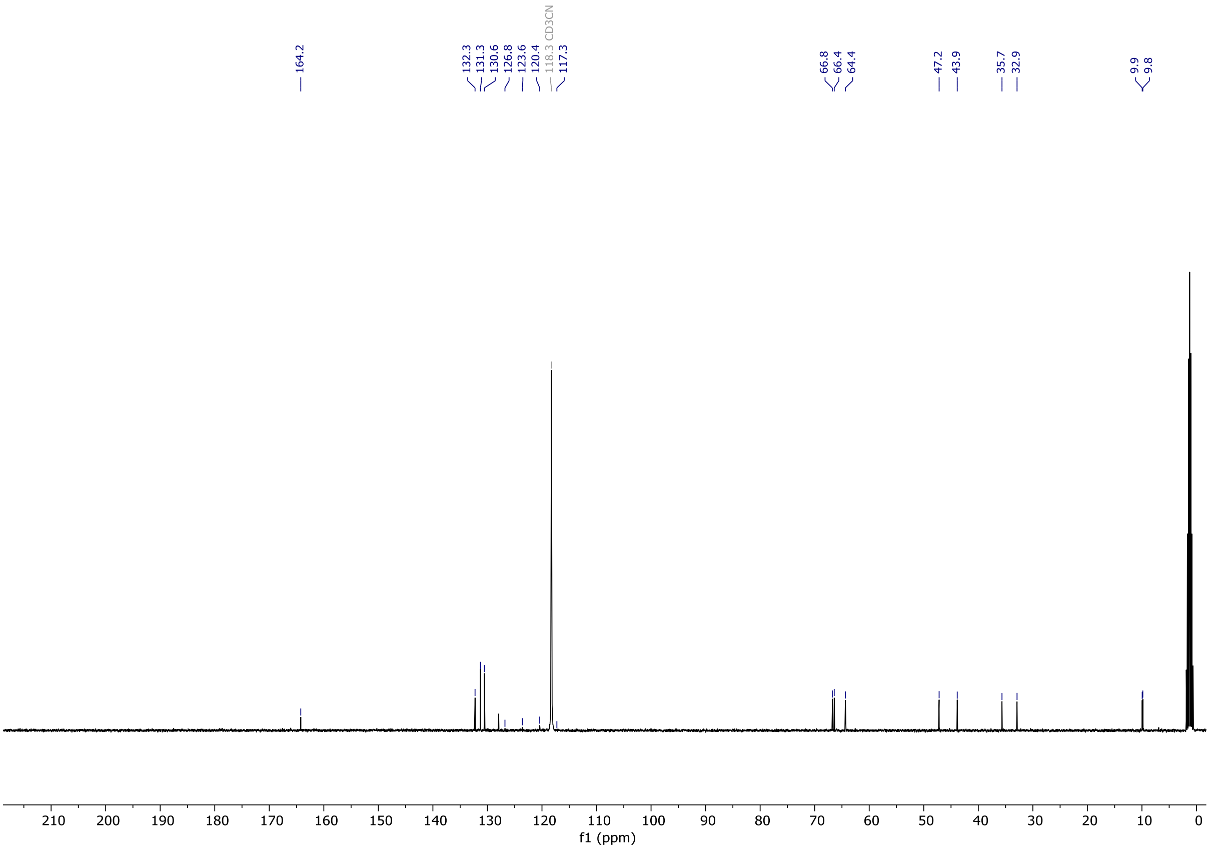

2u – ^{19}F NMR (376 MHz, CD_3CN)

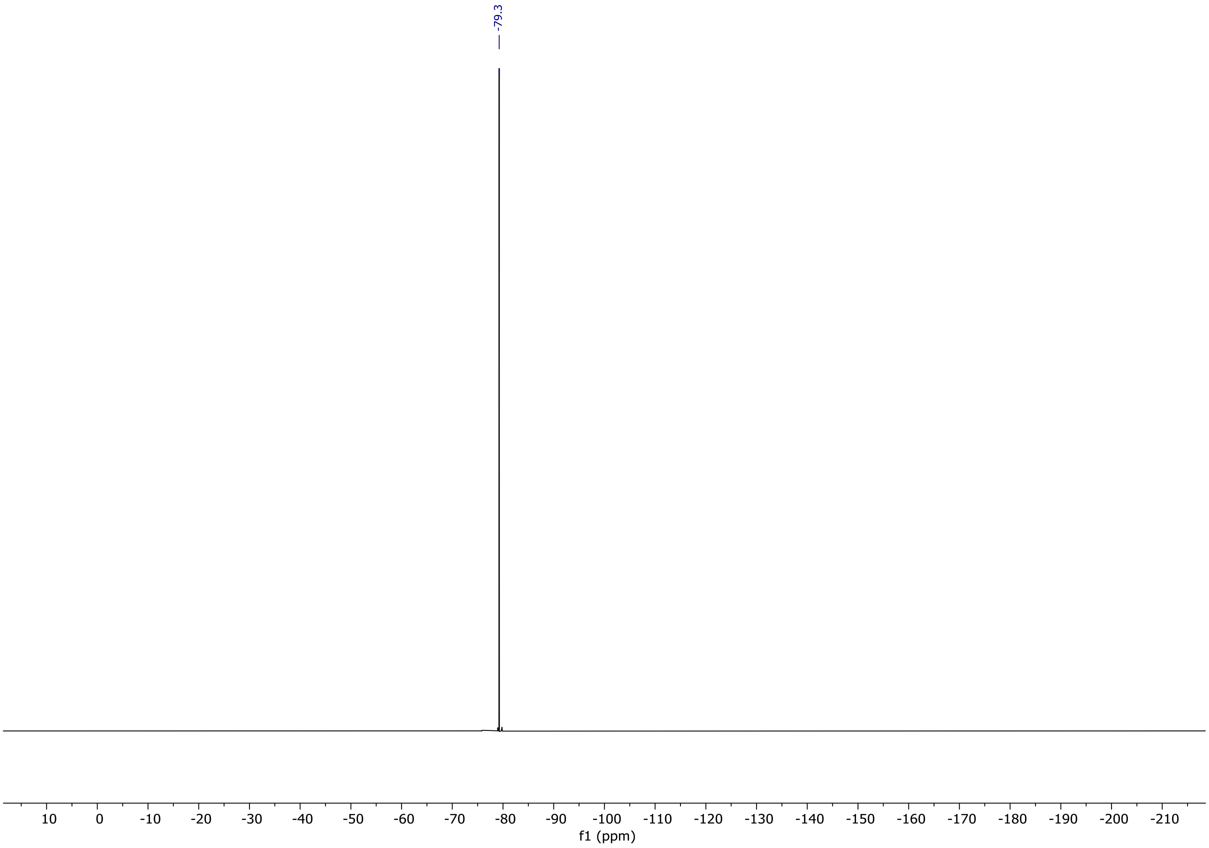

3u – ^{1}H NMR (400 MHz, $CDCl_3$)

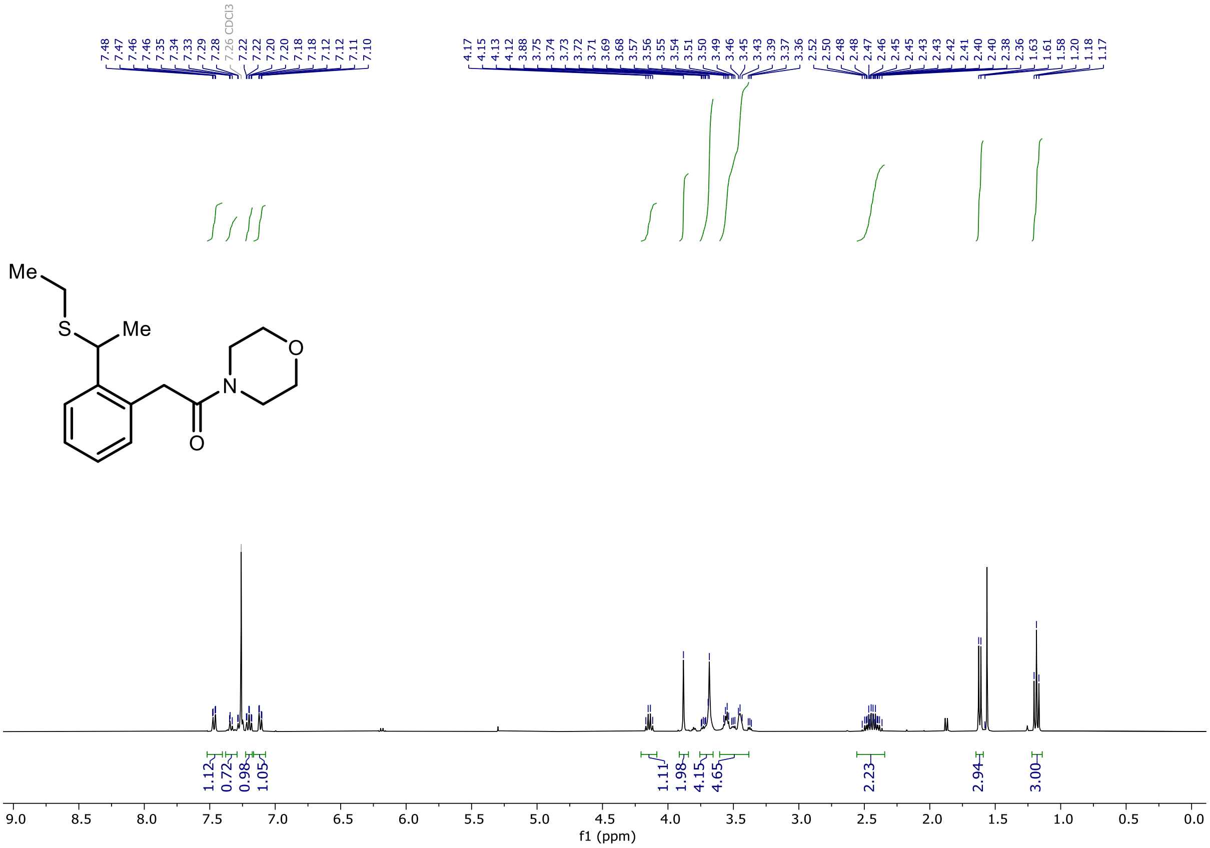

3u – ^{13}C NMR (101 MHz, $CDCl_3$)

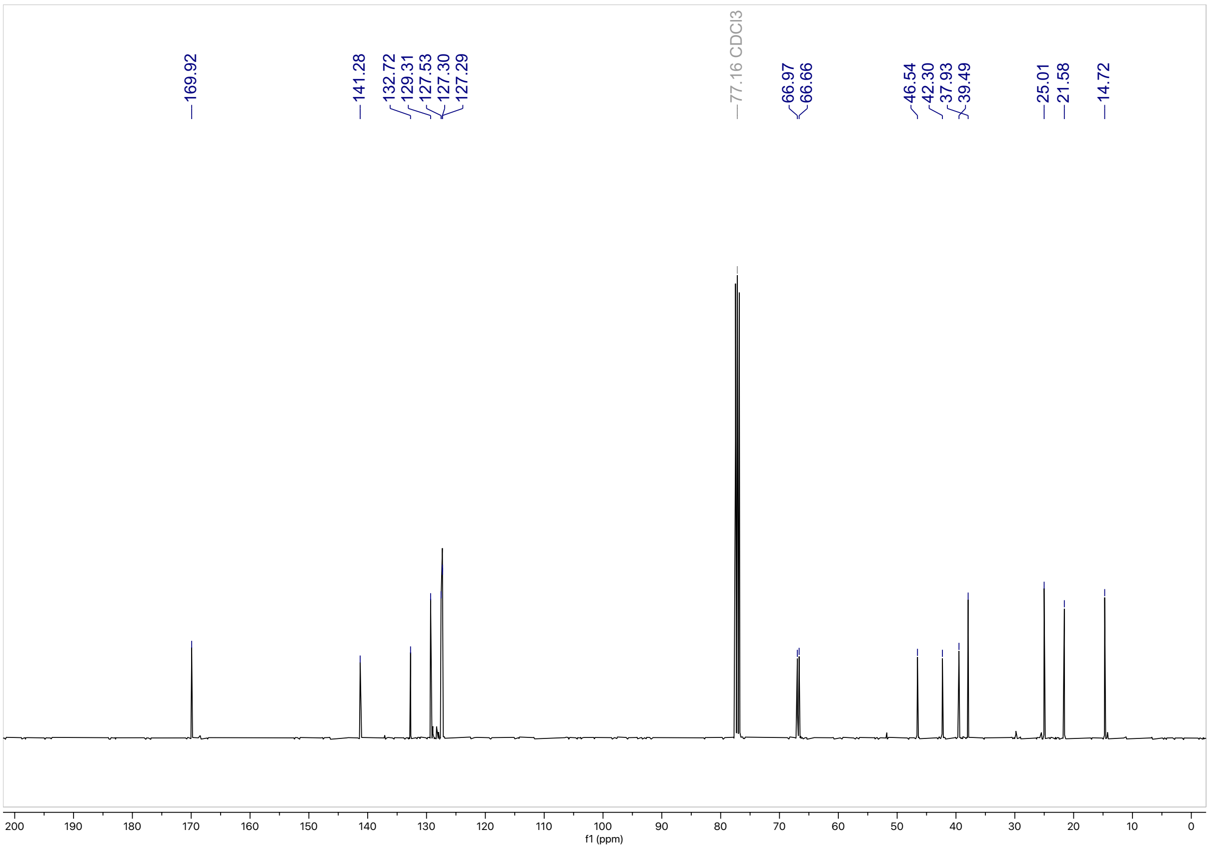

2v – ^{1}H NMR (400 MHz, CDCl$_3$)

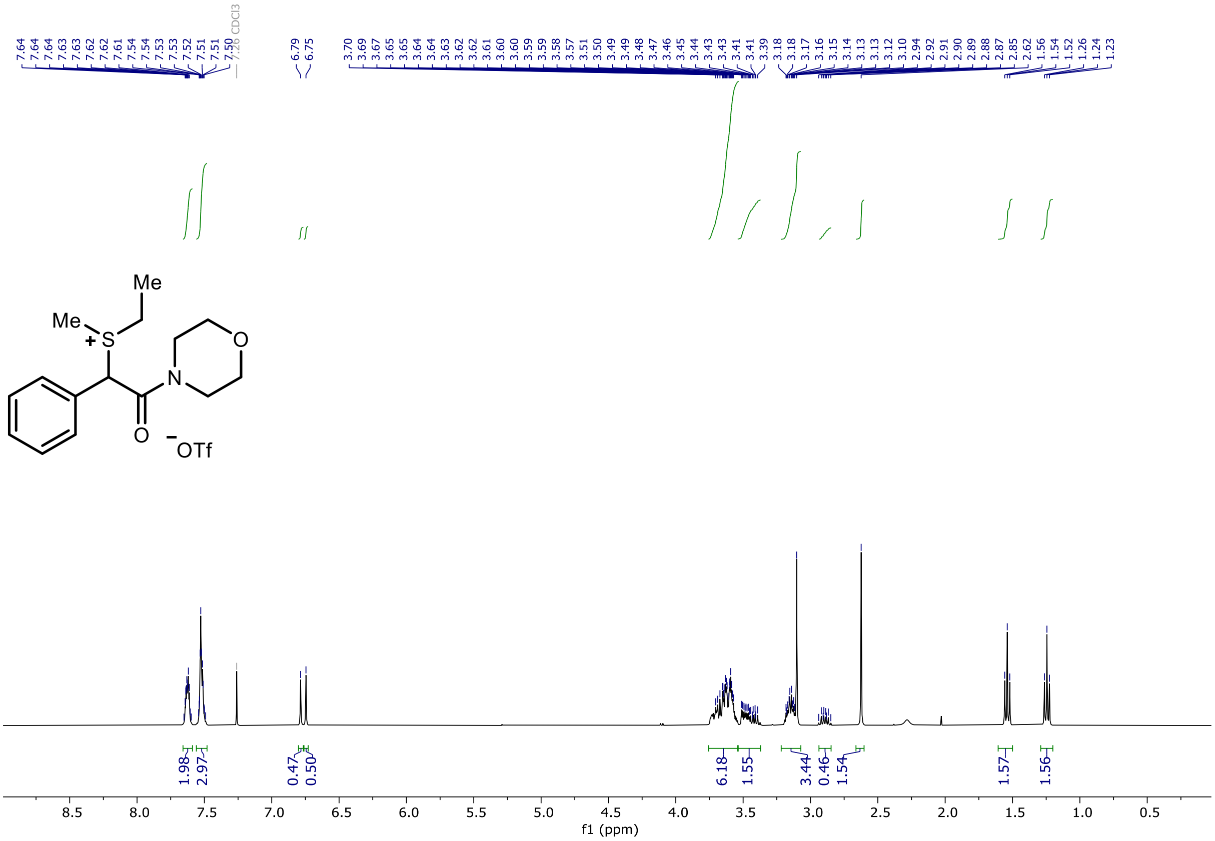

2v – ^{13}C NMR (101 MHz, CDCl$_3$)

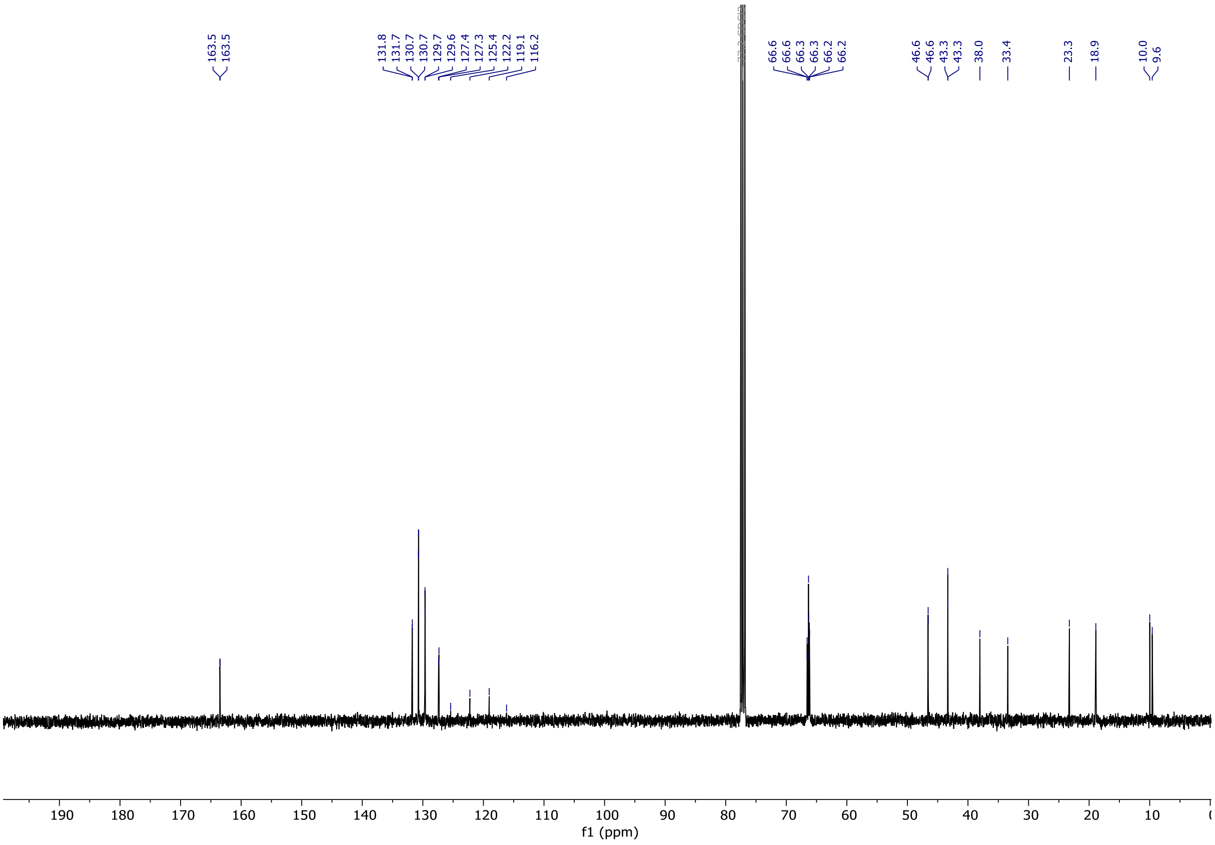

2v – ^{19}F NMR (376 MHz, $CDCl_3$)

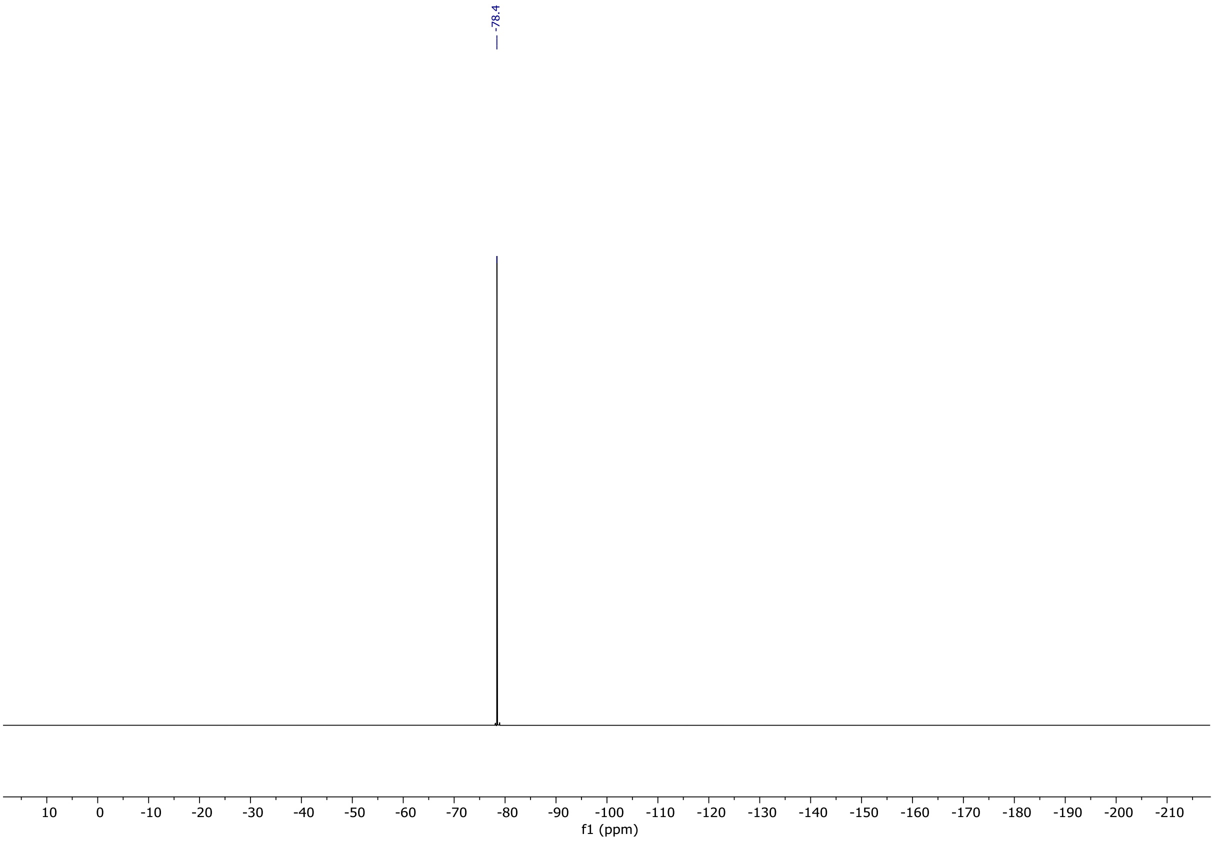

3v – ¹H NMR (400 MHz, $CDCl_3$)

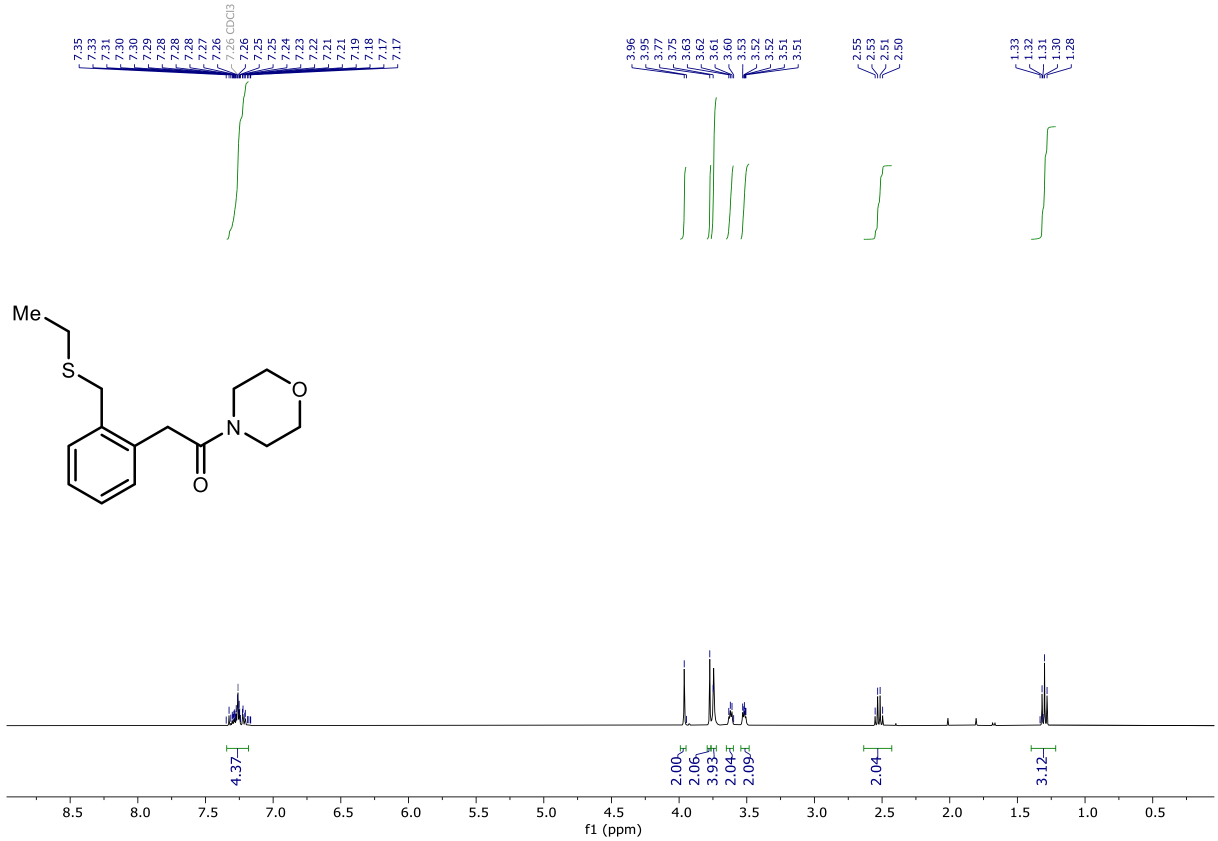

3v – ¹³C NMR (101 MHz, $CDCl_3$)

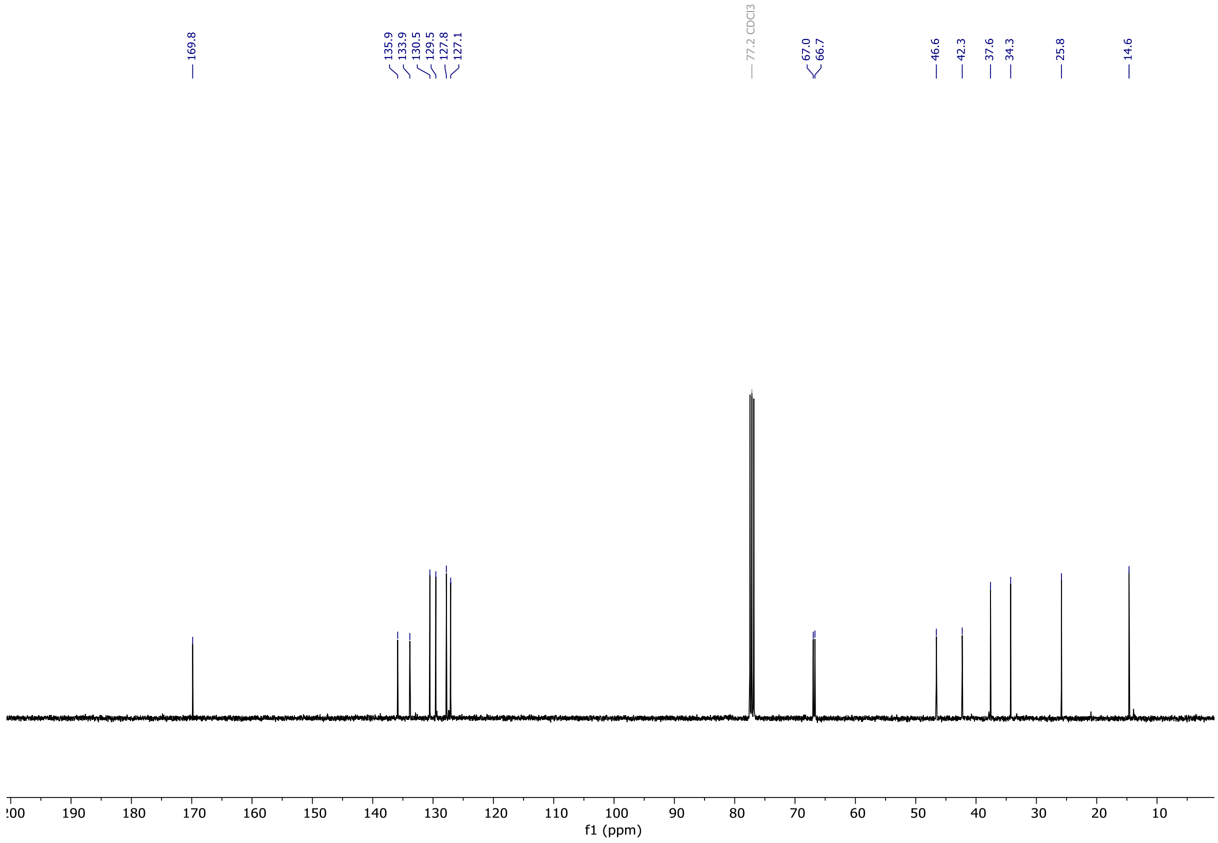

3w – ^{1}H NMR (400 MHz, $CDCl_3$)

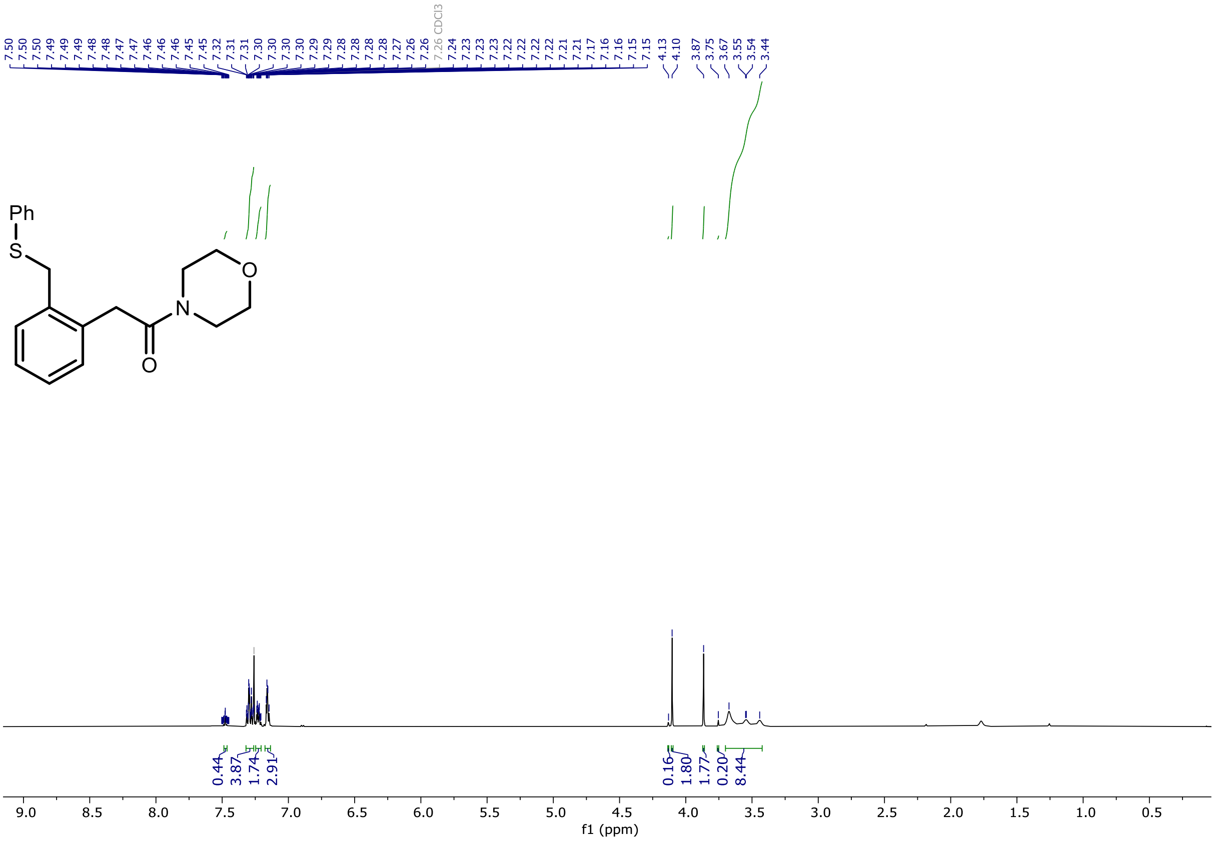

3w – ^{13}C NMR (101 MHz, $CDCl_3$)

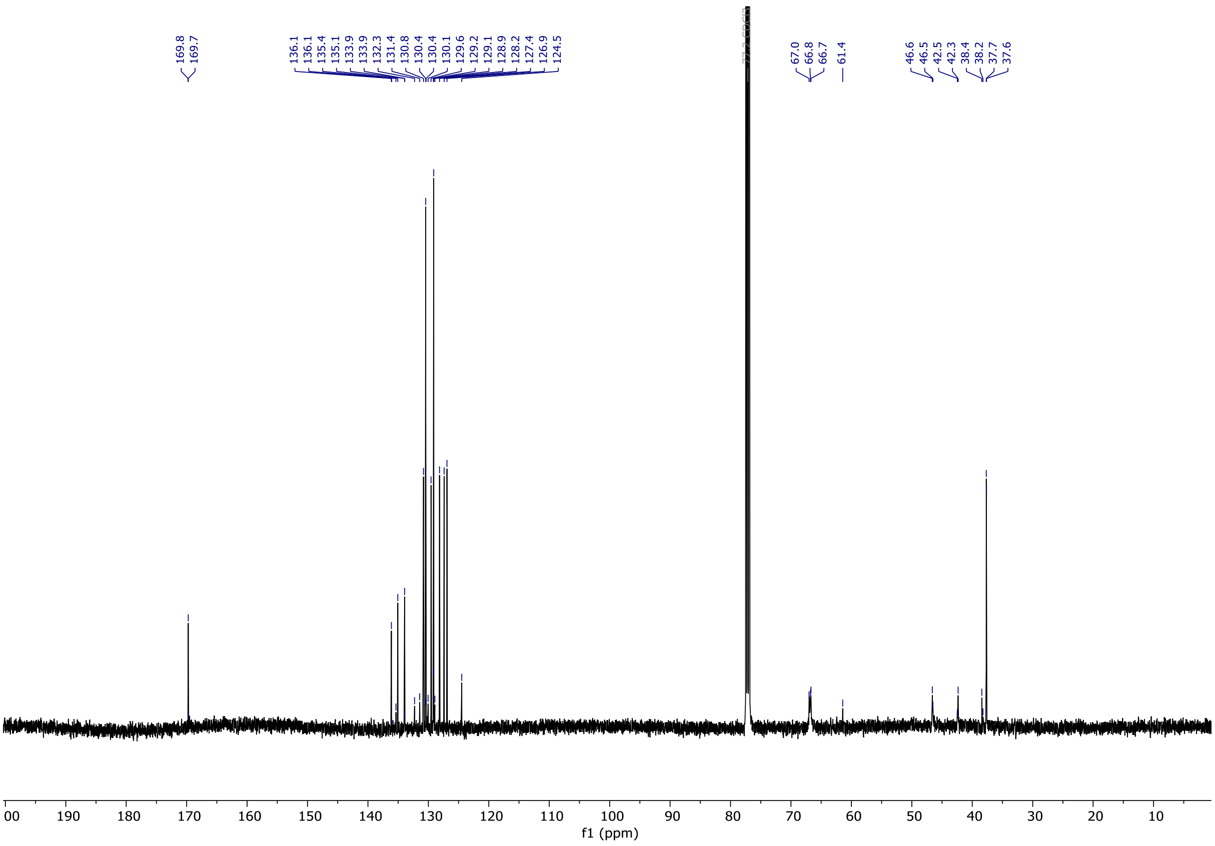

2x – ^{1}H NMR (400 MHz, CD_3CN)

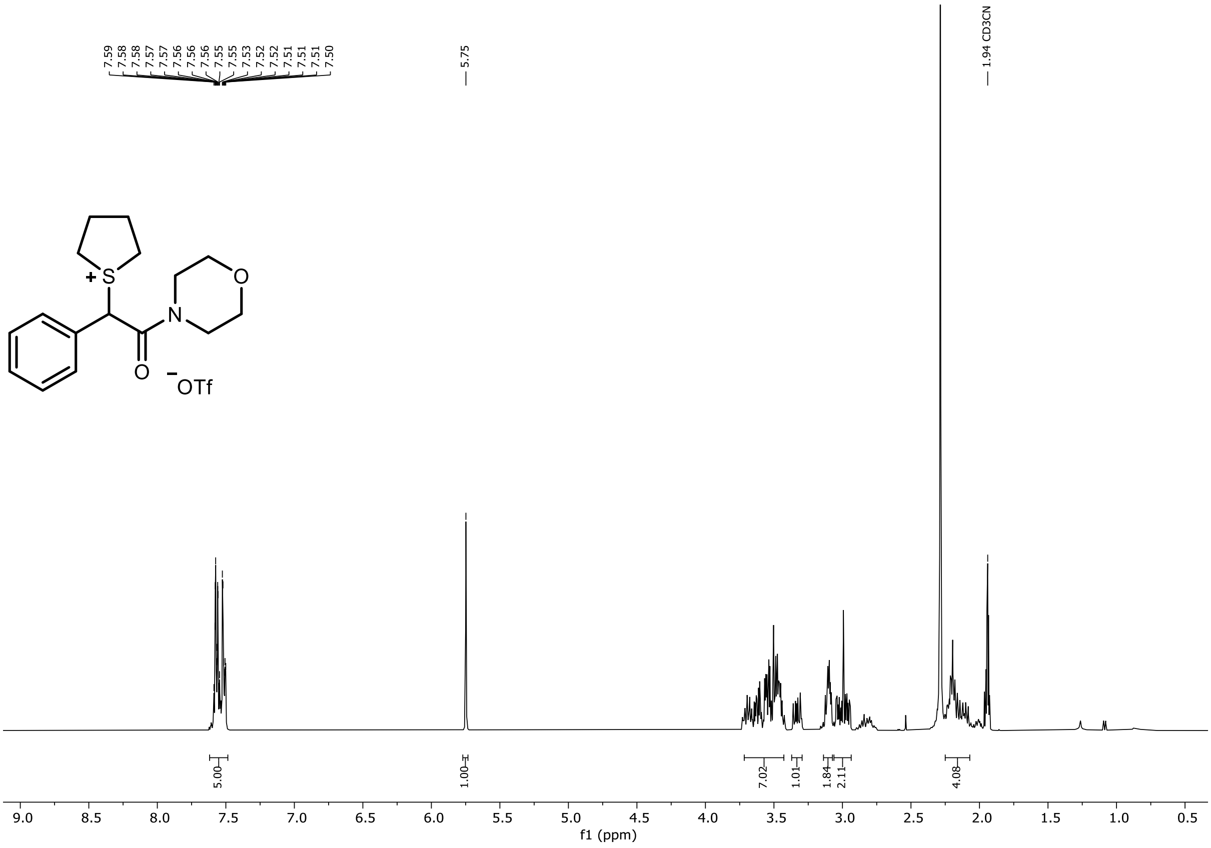

2x – ^{13}C NMR (101 MHz, CD_3CN)

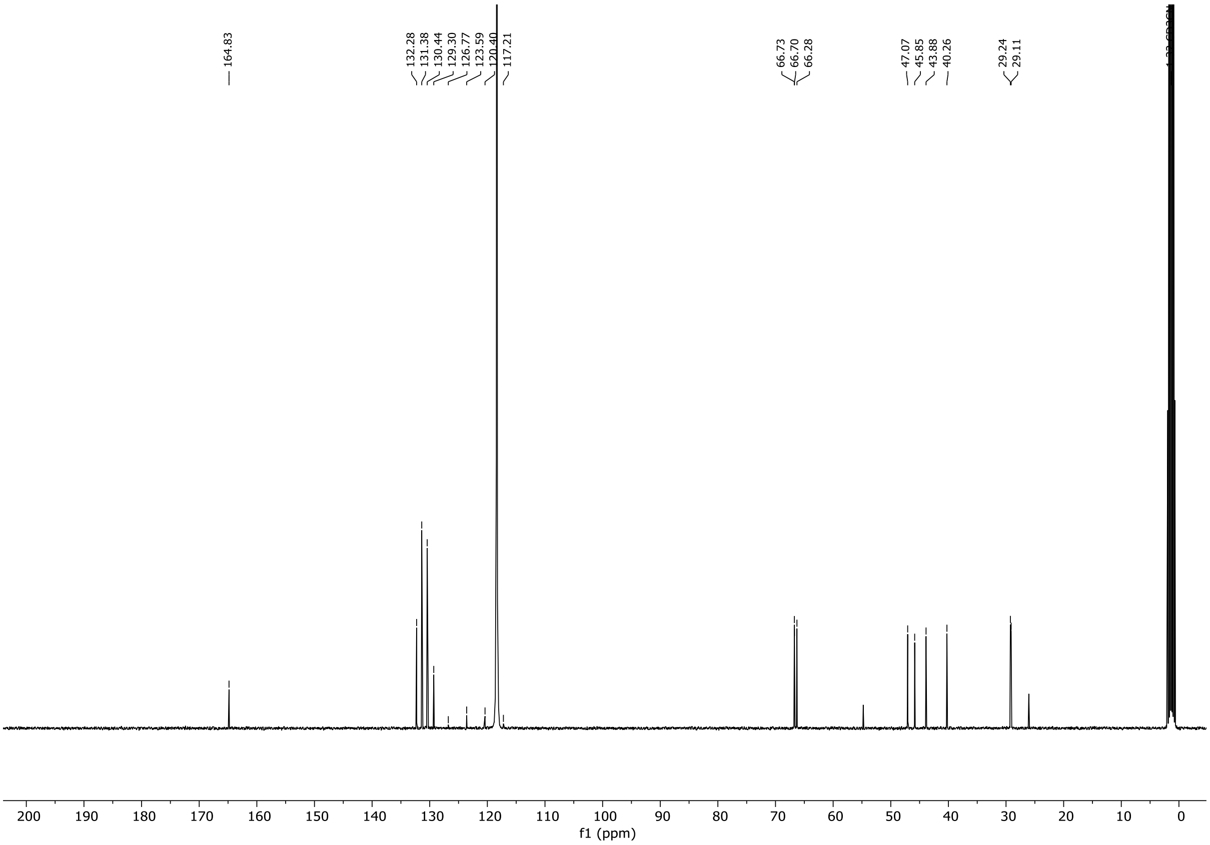

2x – ^{19}F NMR (376 MHz, CD_3CN)

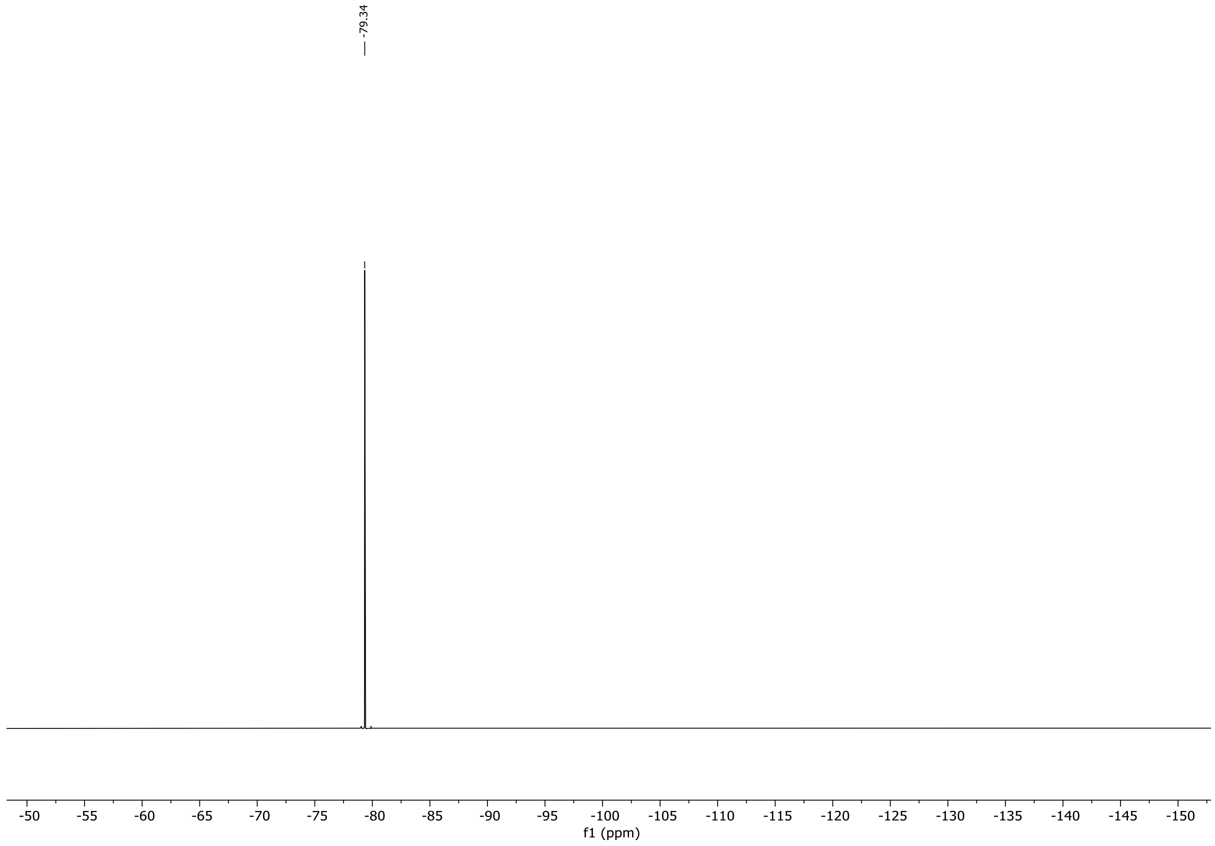

3x – ^{1}H NMR (400 MHz, $CDCl_3$)

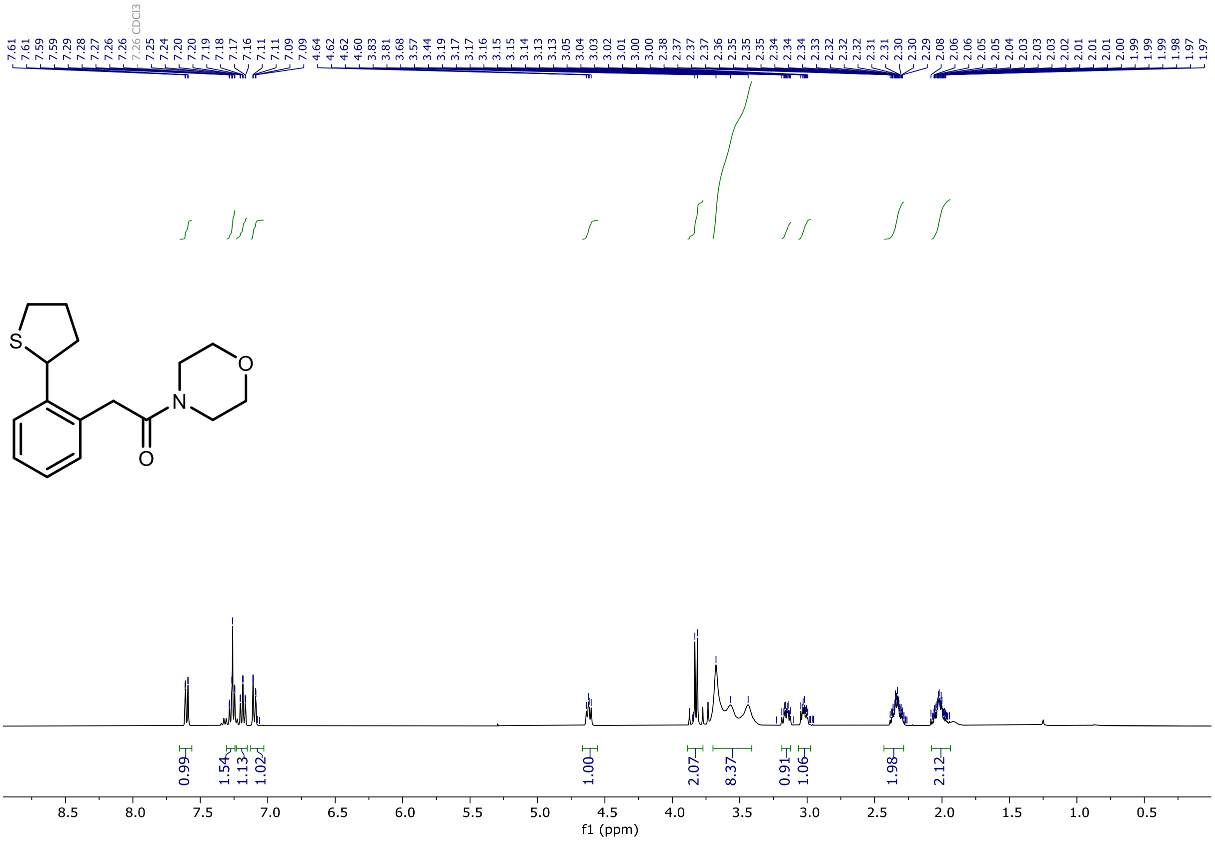

3x – ^{13}C NMR (101 MHz, $CDCl_3$)

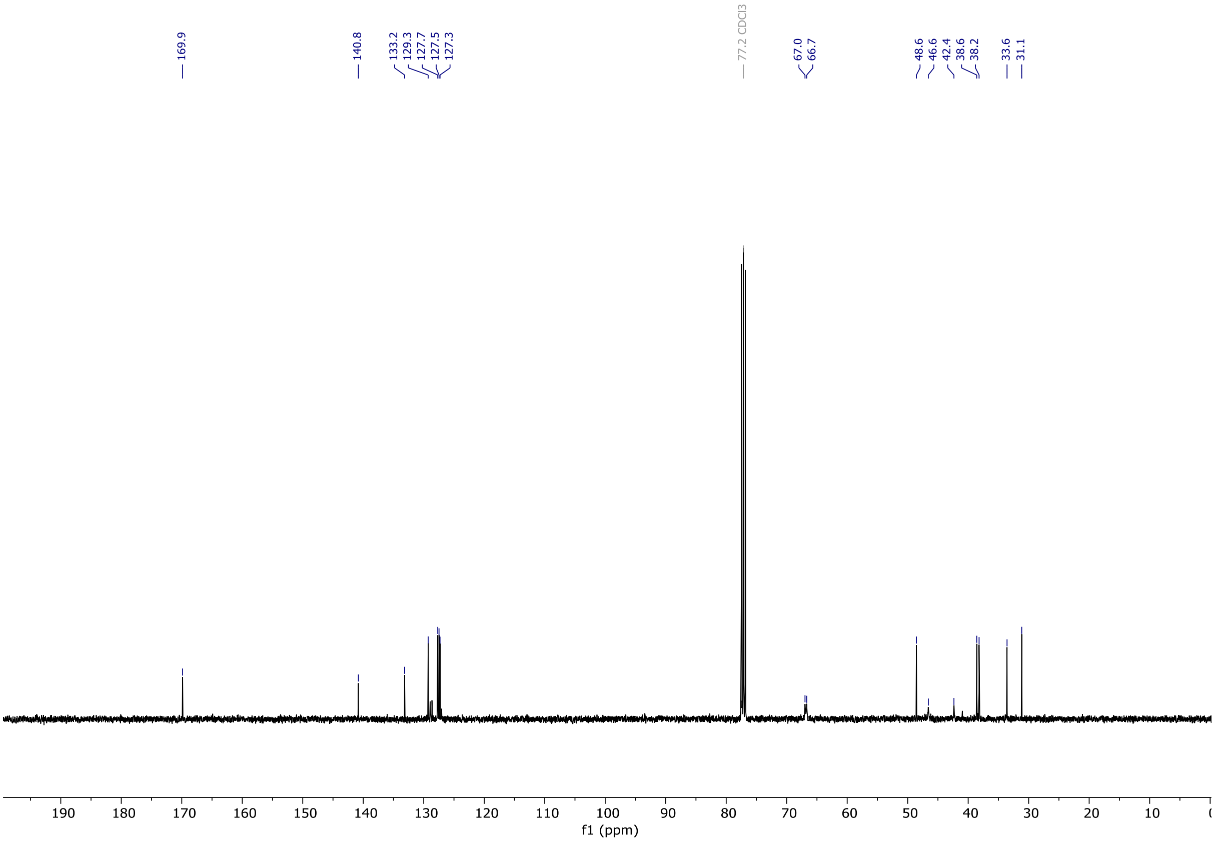

2y – ^{1}H NMR (400 MHz, $CDCl_3$)

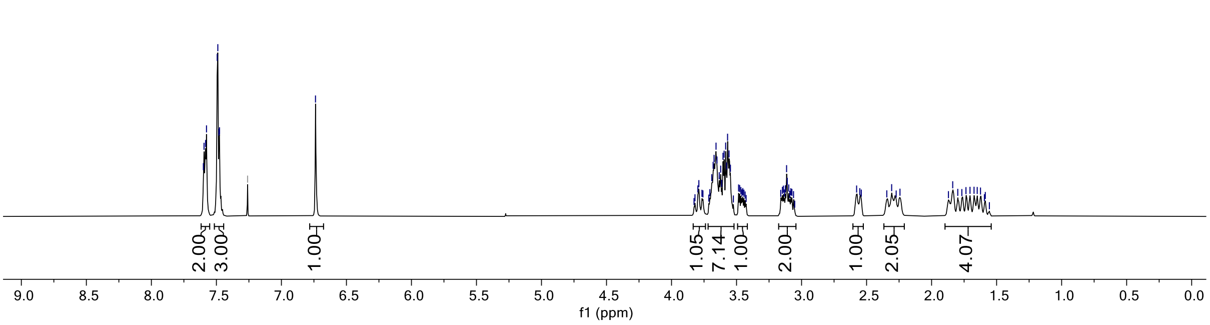

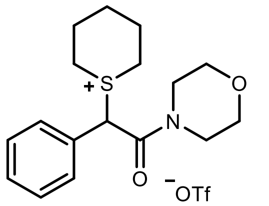

2y – ^{13}C NMR (101 MHz, $CDCl_3$)

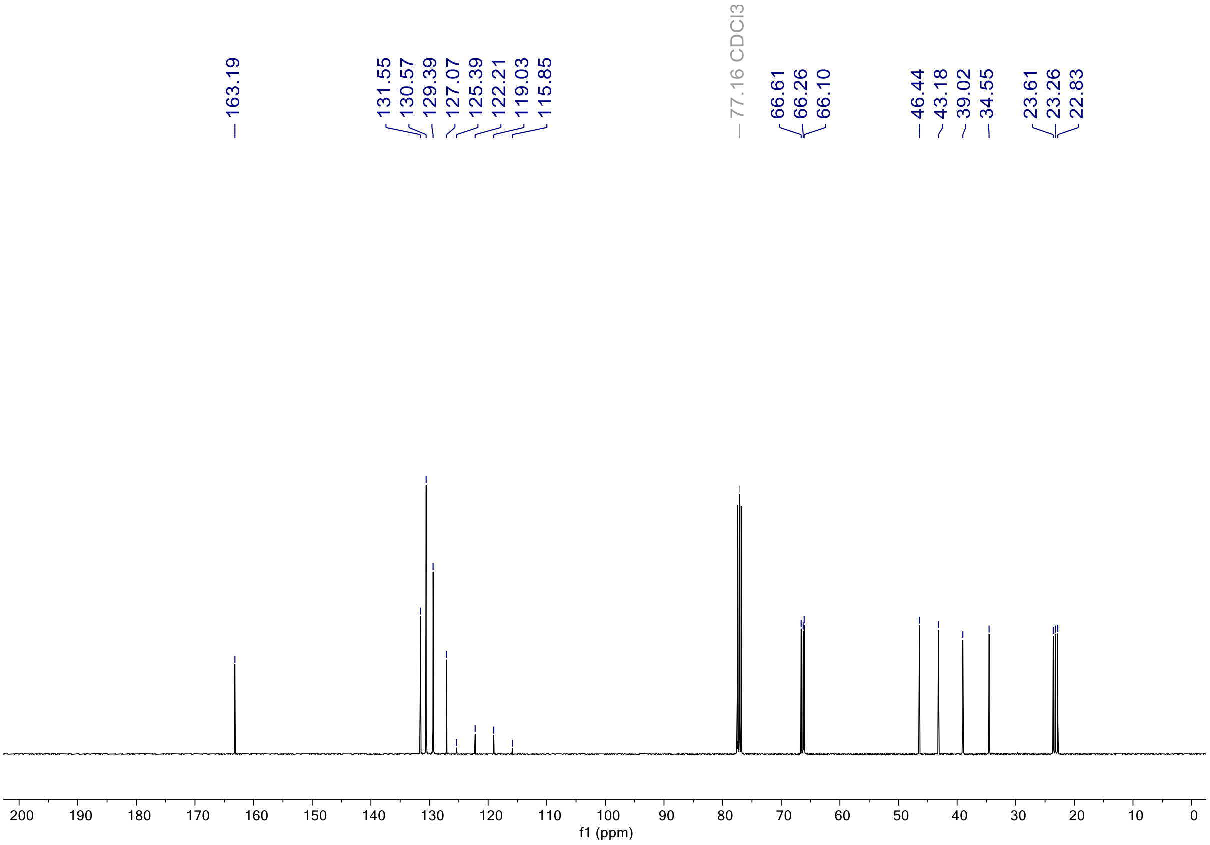

2y – ^{19}F NMR (376 MHz, $CDCl_3$)

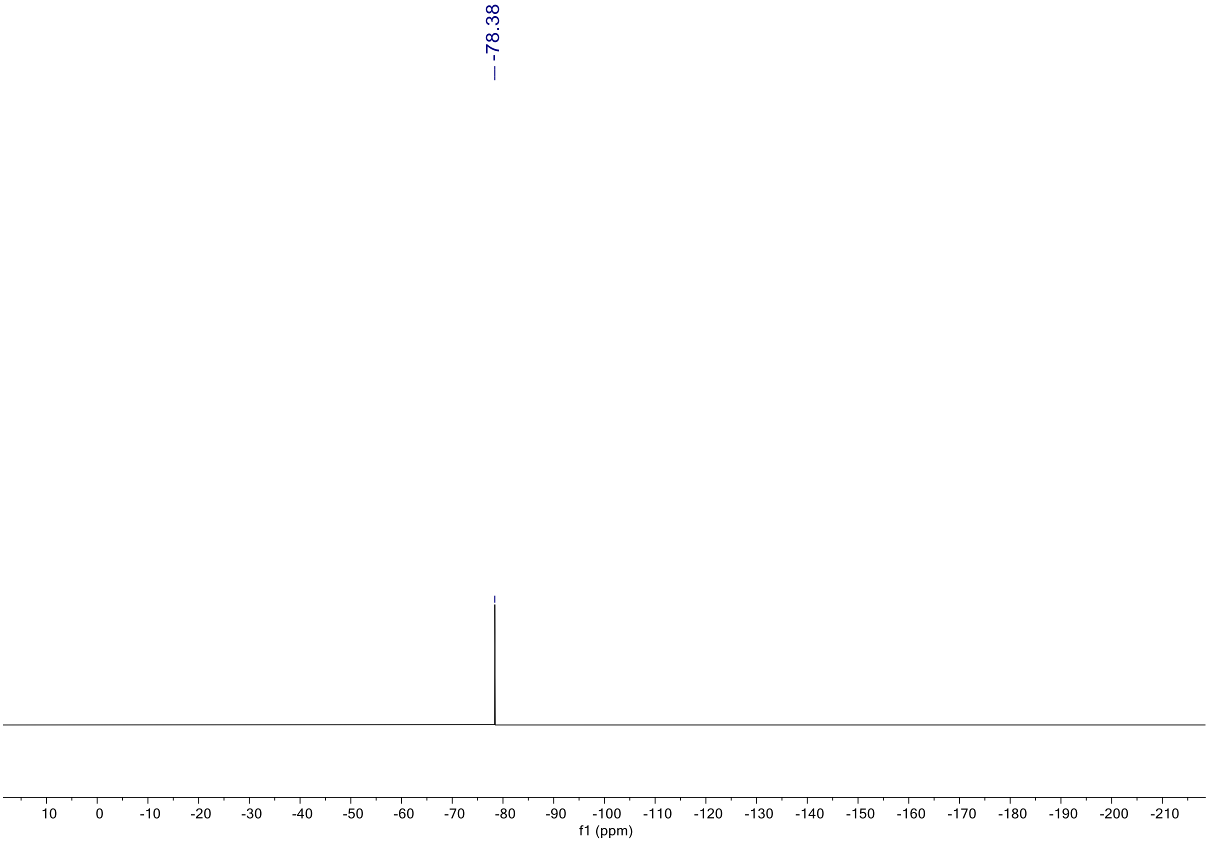

3y – ^{1}H NMR (400 MHz, $CDCl_3$)

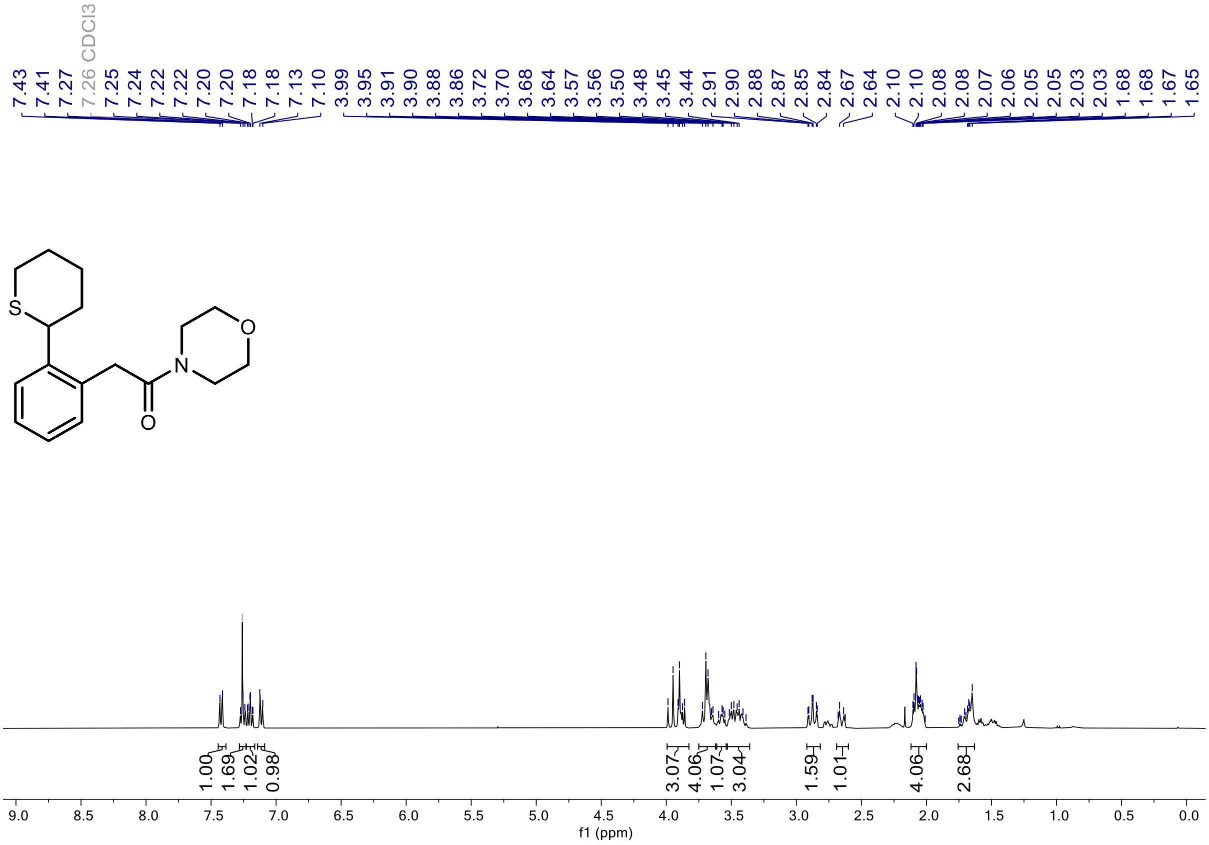

3y – ^{13}C NMR (101 MHz, $CDCl_3$)

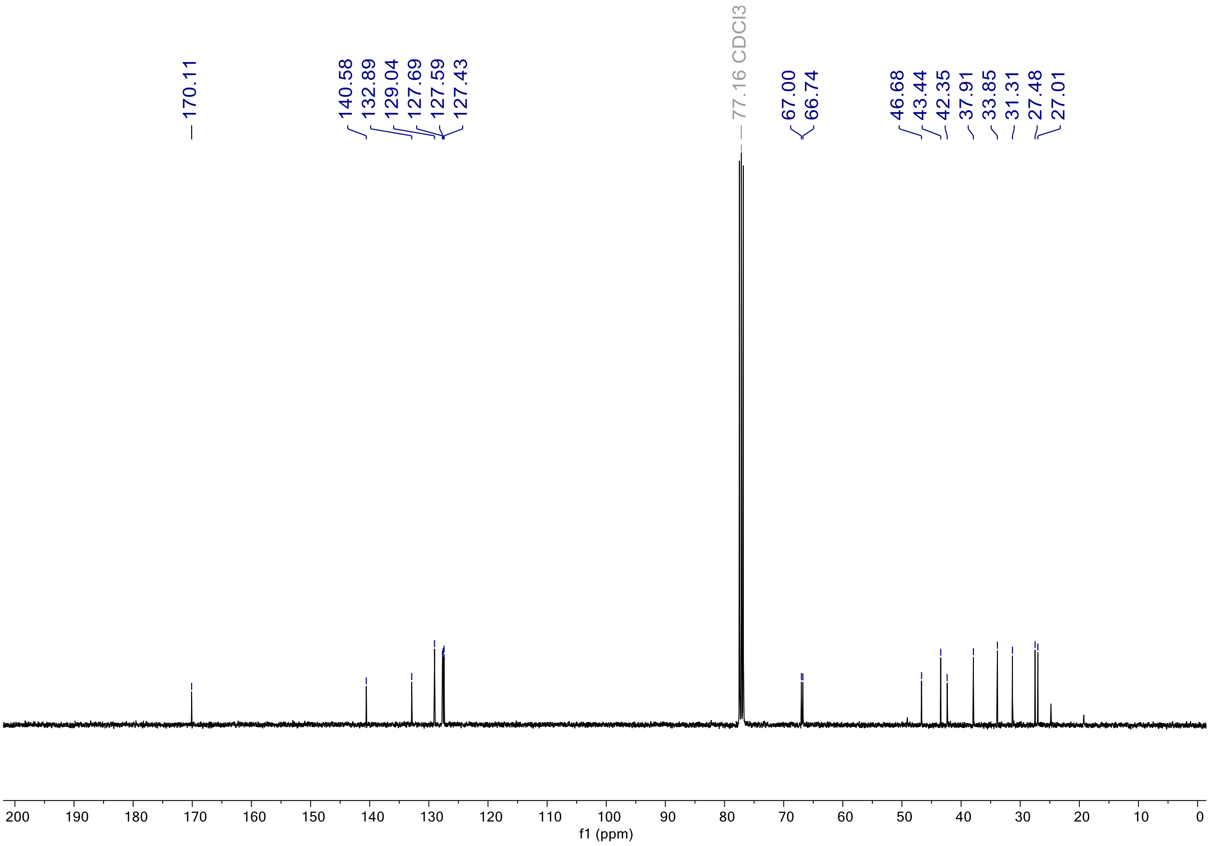

2z – ^{1}H NMR (400 MHz, $CDCl_3$)

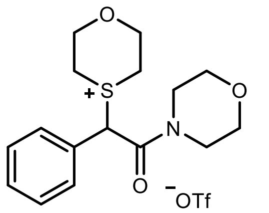

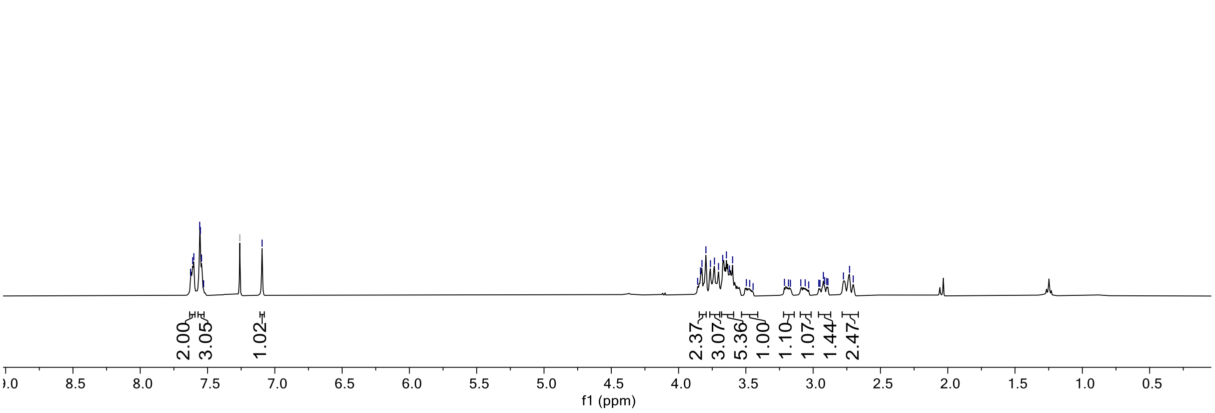

2z – ^{13}C NMR (101 MHz, $CDCl_3$)

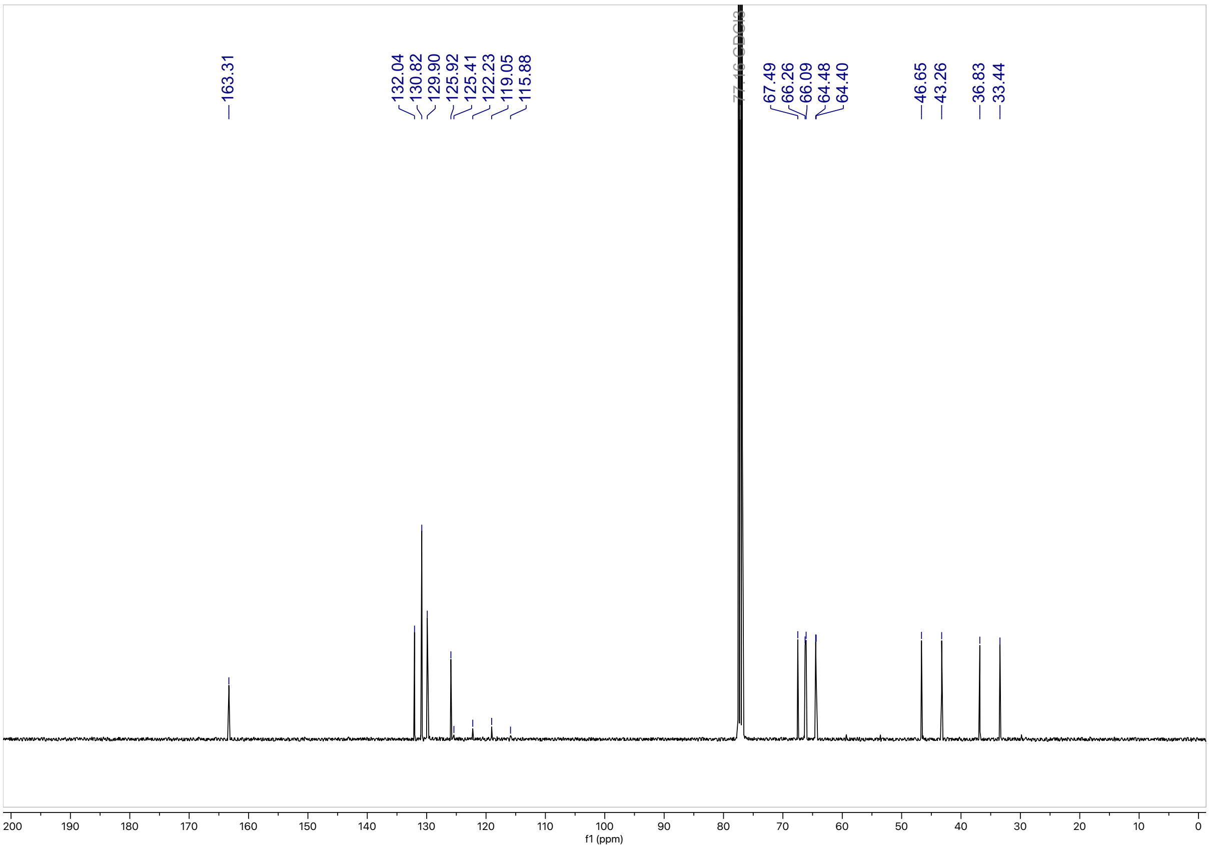

2z – ^{19}F NMR (376 MHz, $CDCl_3$)

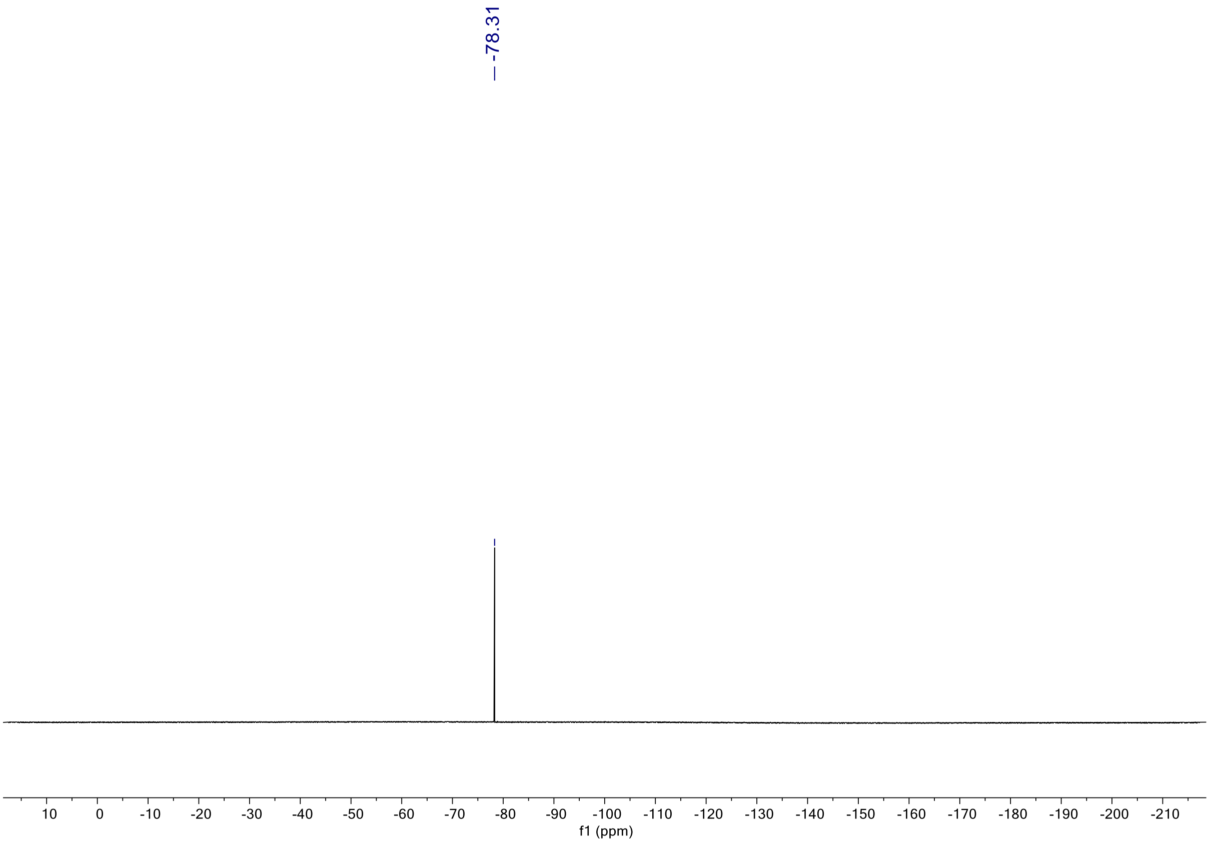

3z – ^{1}H NMR (400 MHz, $CDCl_3$)

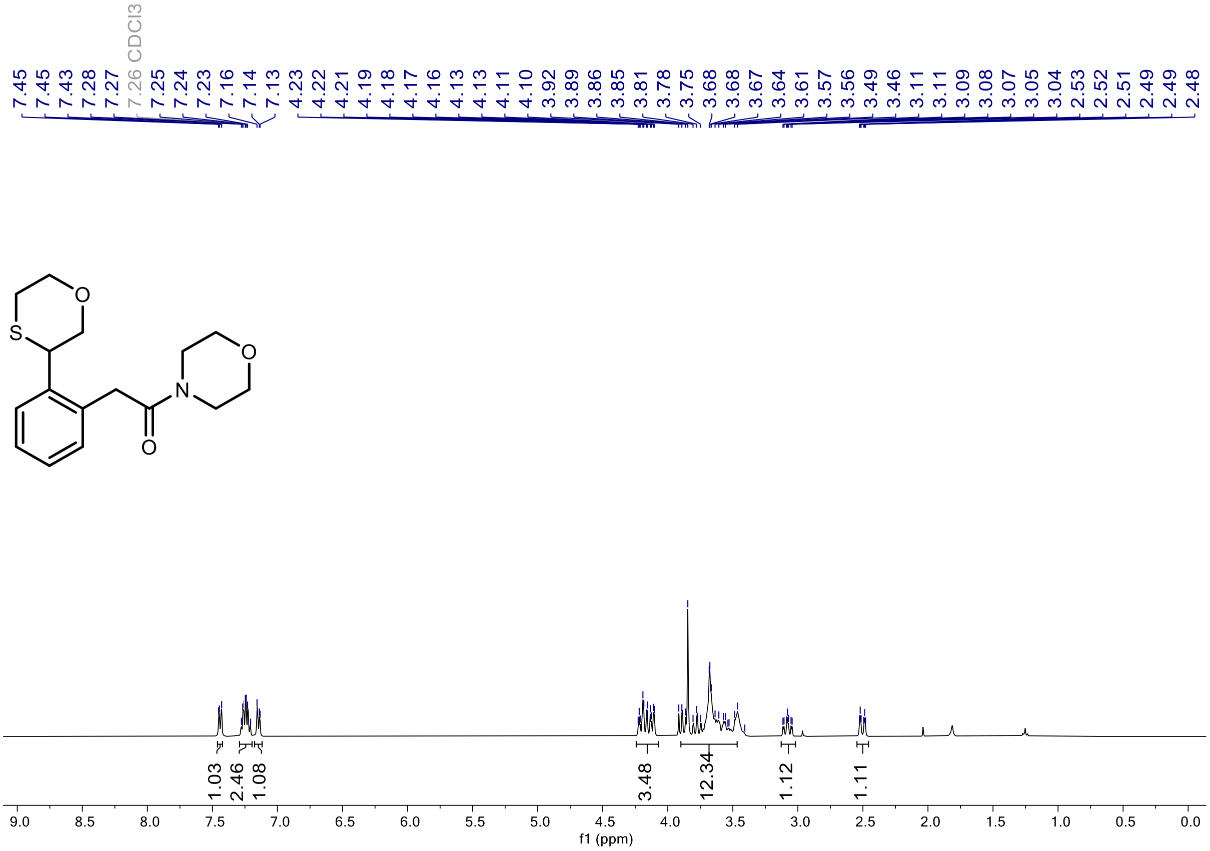

3z – ^{13}C NMR (101 MHz, $CDCl_3$)

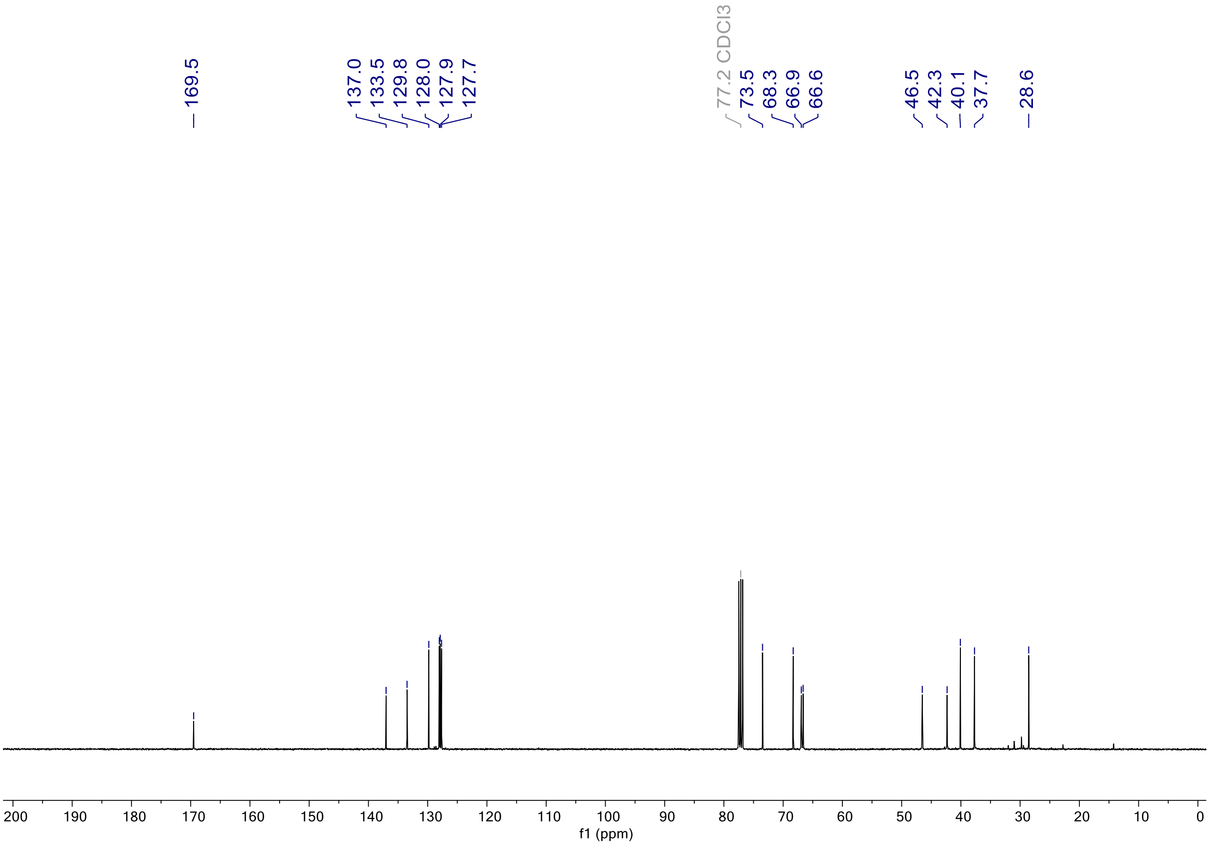

2aa – ^{1}H NMR (400 MHz, CDCl$_3$)

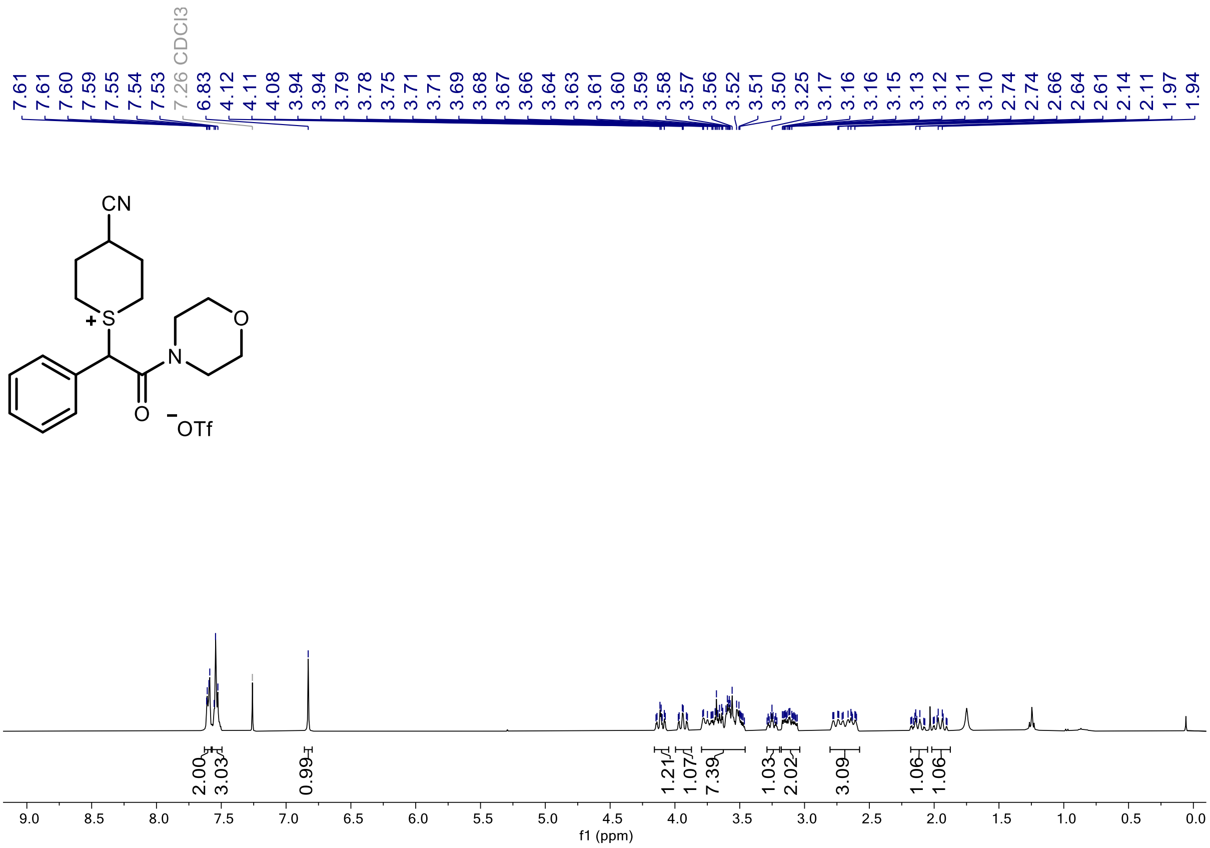

2aa – ^{13}C NMR (101 MHz, CDCl$_3$)

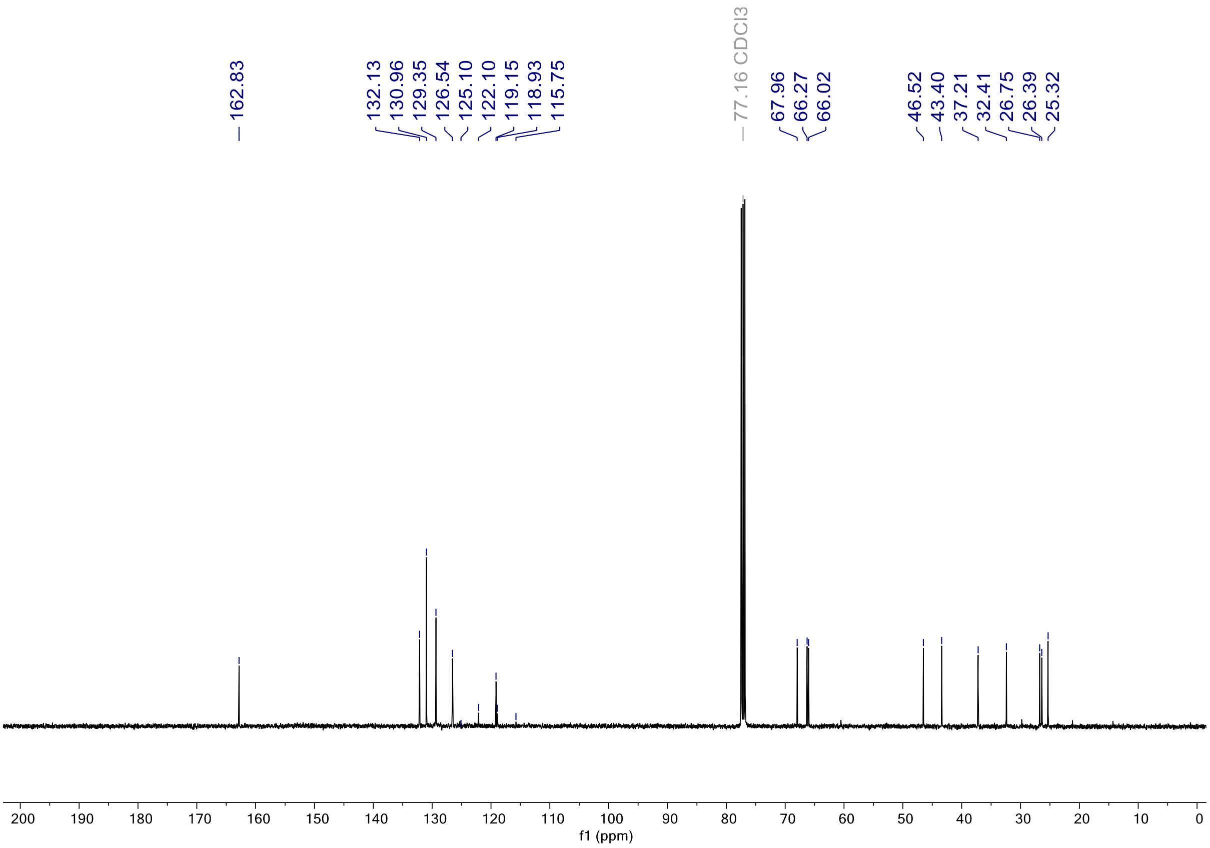

2aa – ^{19}F NMR (376 MHz, $CDCl_3$)

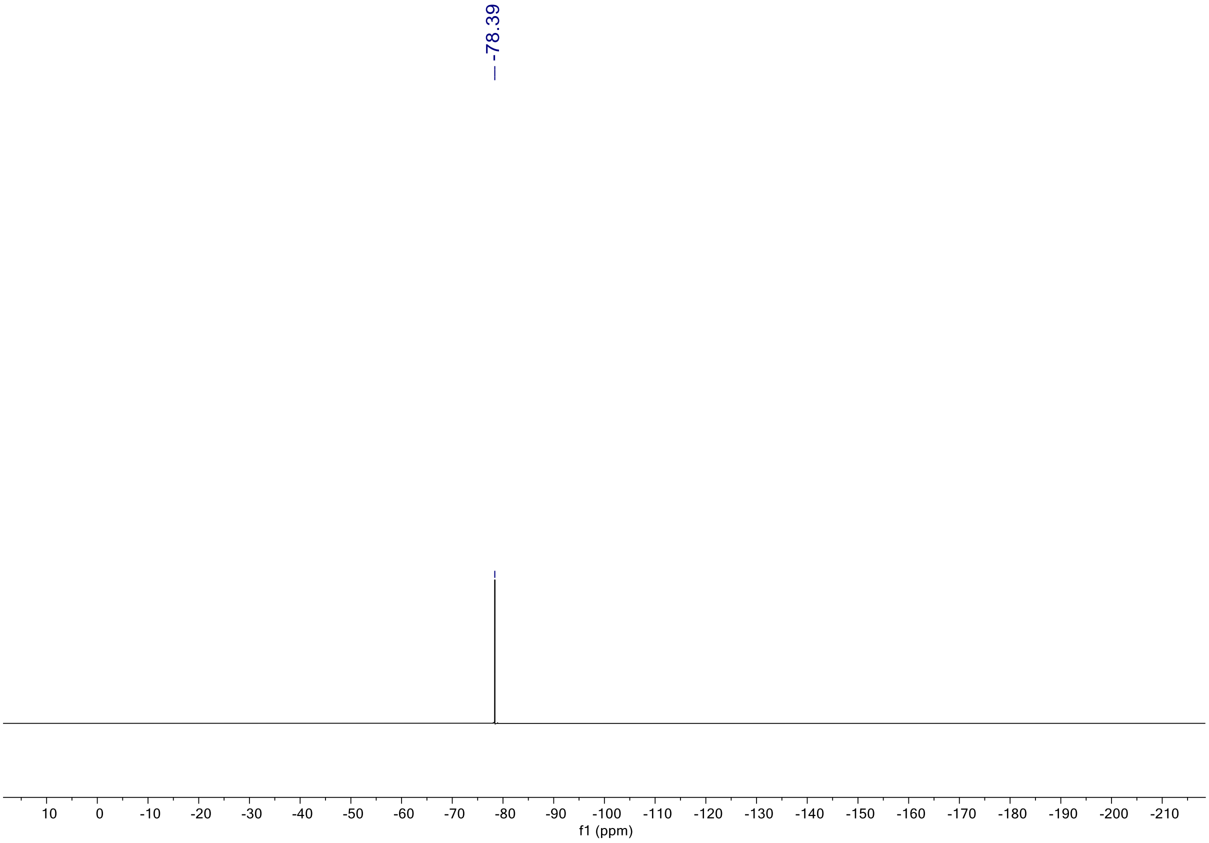

3aa – ^{1}H NMR (400 MHz, CDCl$_3$)

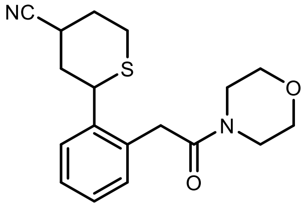

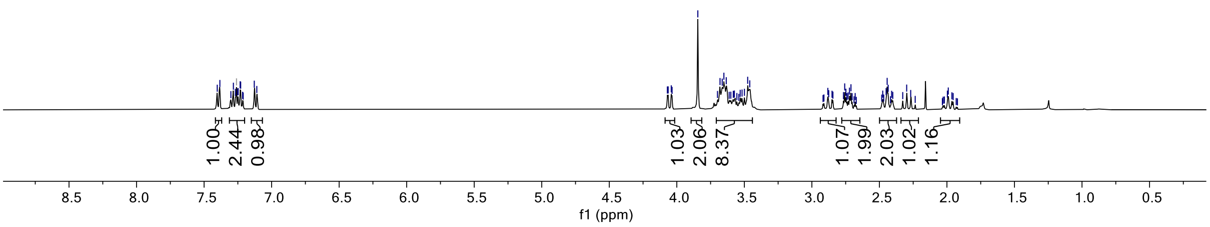

3aa – ^{13}C NMR (101 MHz, CDCl$_3$)

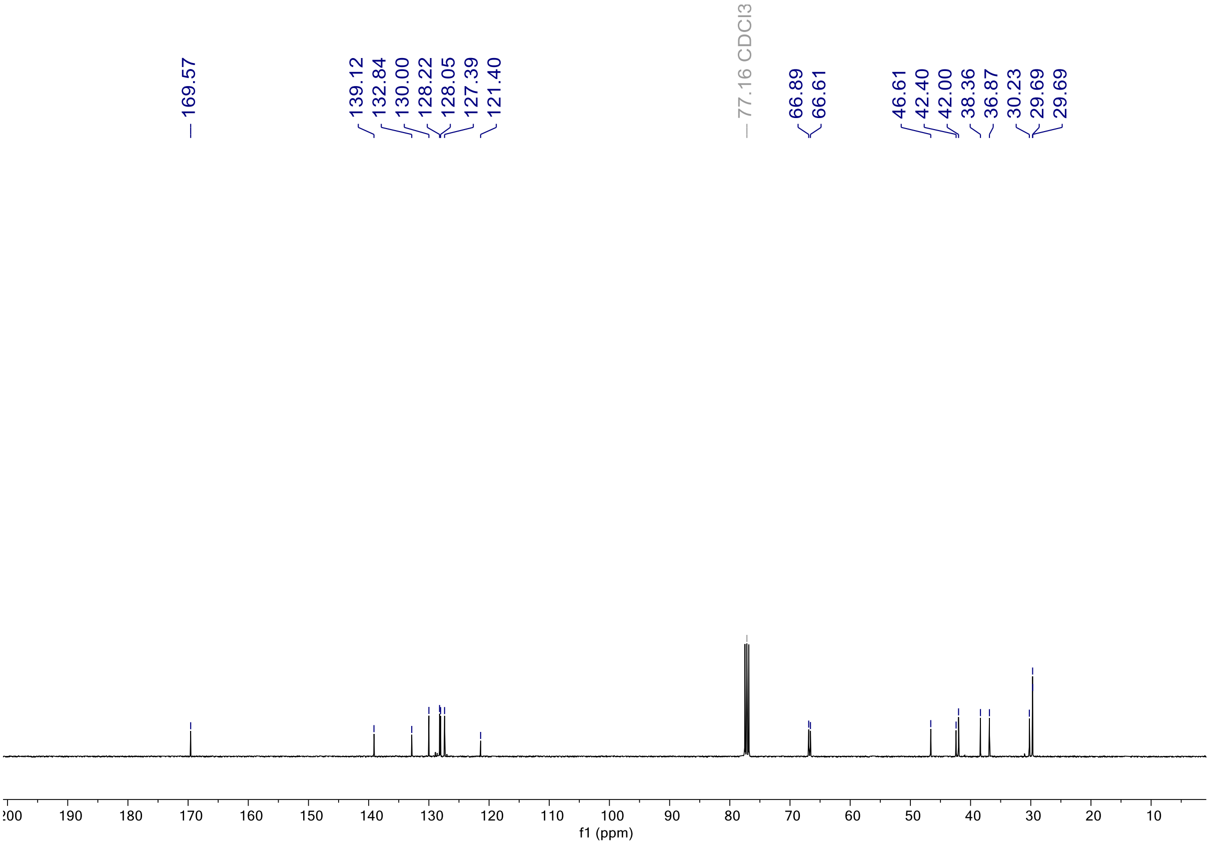

2ab – ^{1}H NMR (400 MHz, $CDCl_3$, mixture of rotamers)

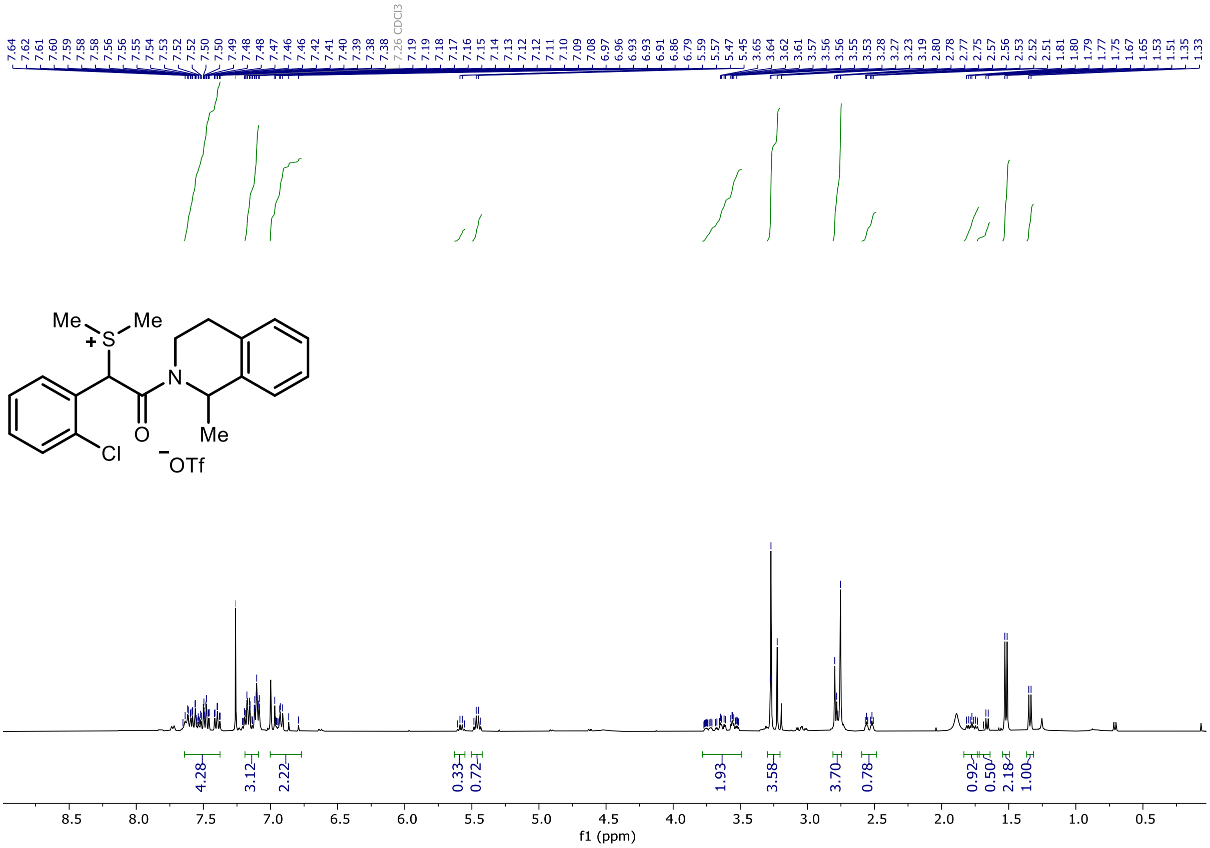

2ab – ^{13}C NMR (101 MHz, $CDCl_3$, mixture of rotamers)

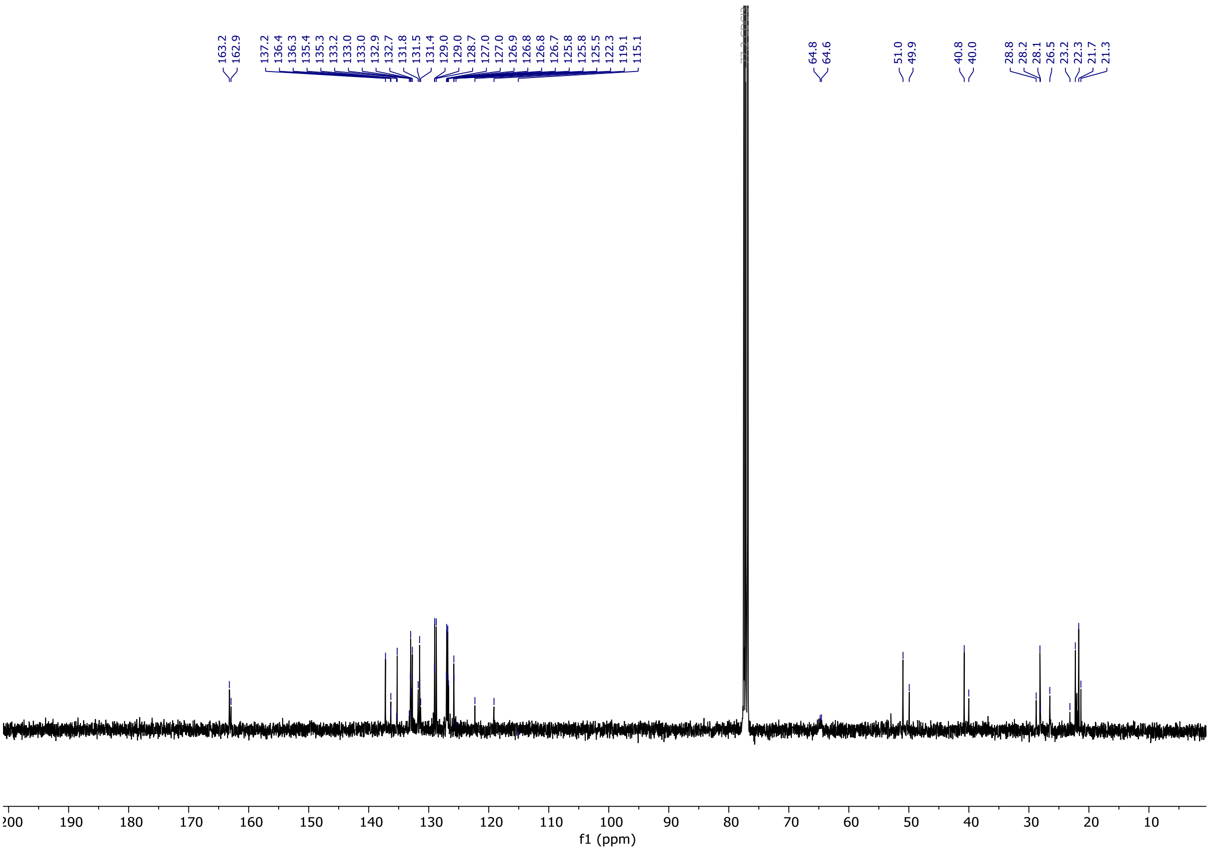

2ab – ^{19}F NMR (376 MHz, $CDCl_3$)

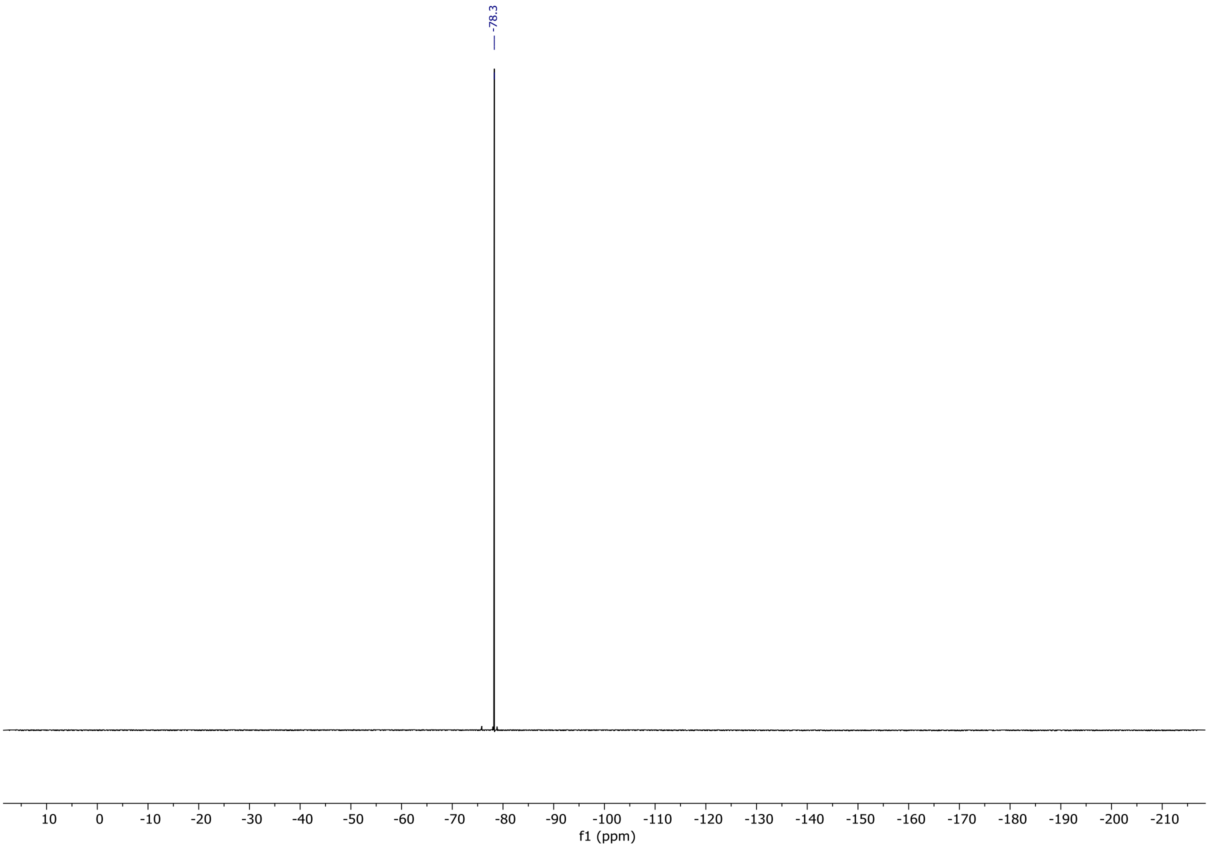

3ab – ^{1}H NMR (400 MHz, $CDCl_3$, mixture of rotamers)

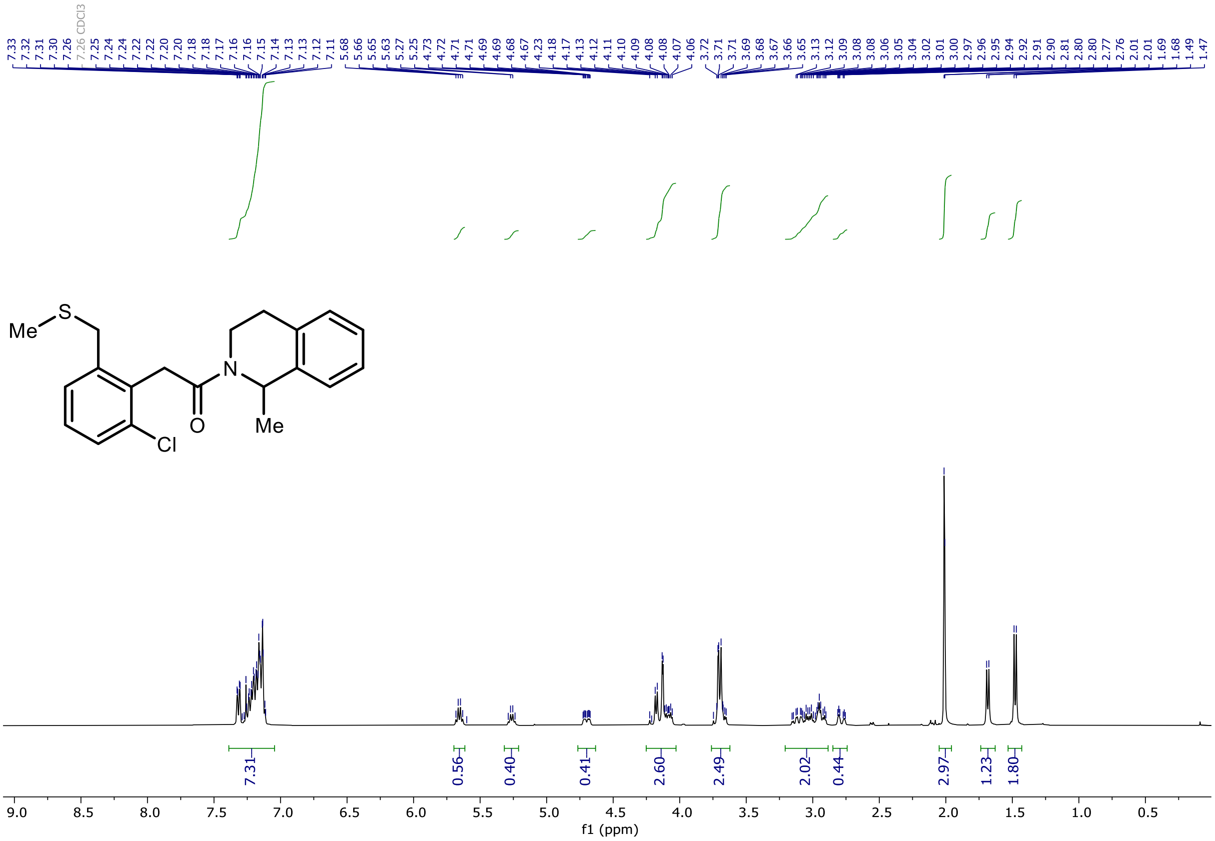

3ab – ^{13}C NMR (101 MHz, $CDCl_3$, mixture of rotamers)

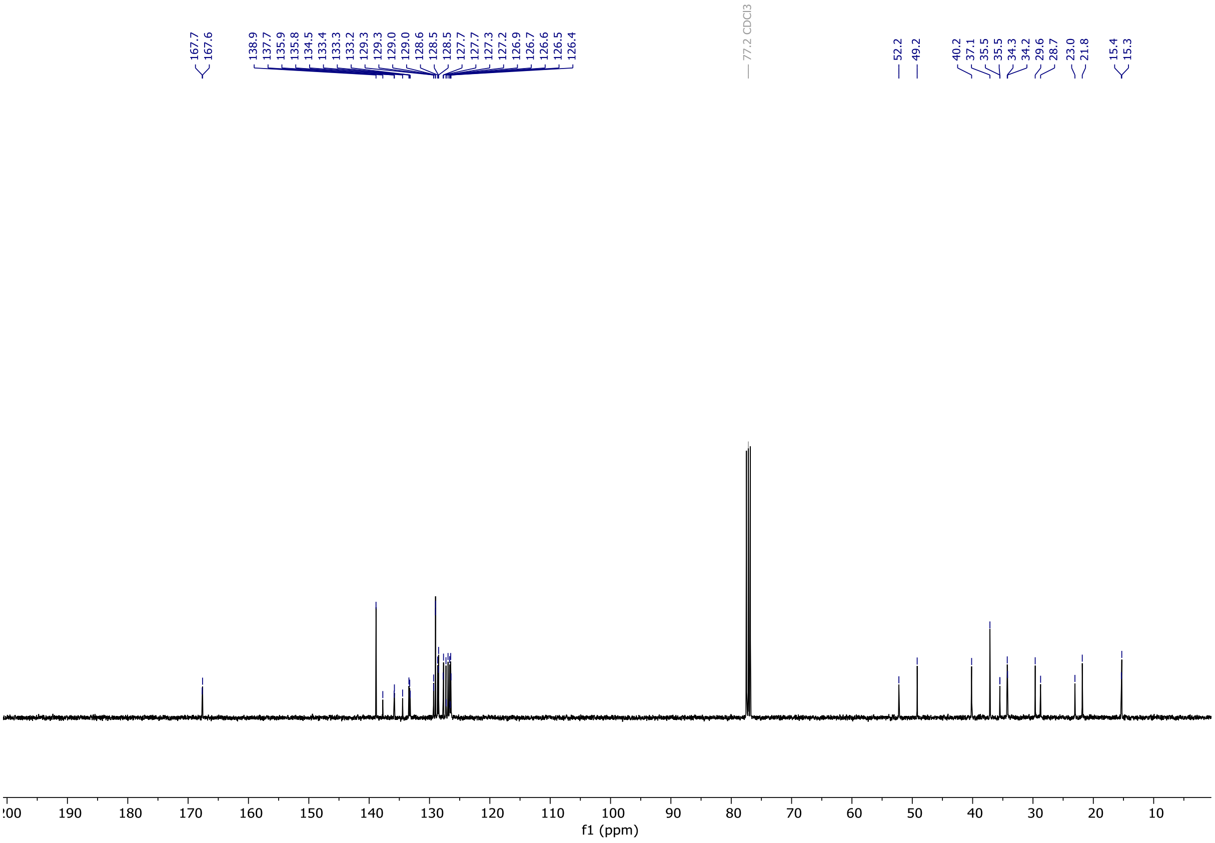

I – ¹H NMR (400 MHz, $CDCl_3$, mixture of rotamers)

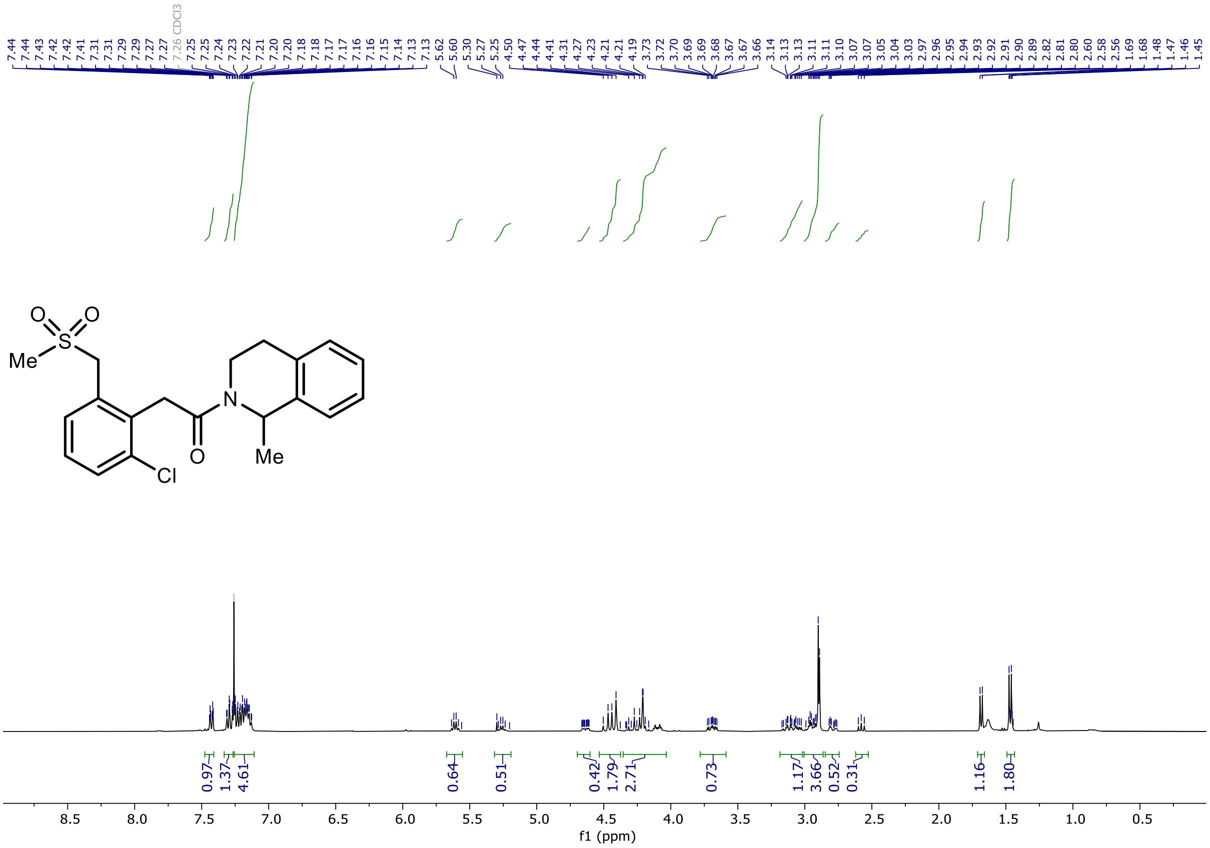

I – ¹³C NMR (101 MHz, $CDCl_3$, mixture of rotamers)

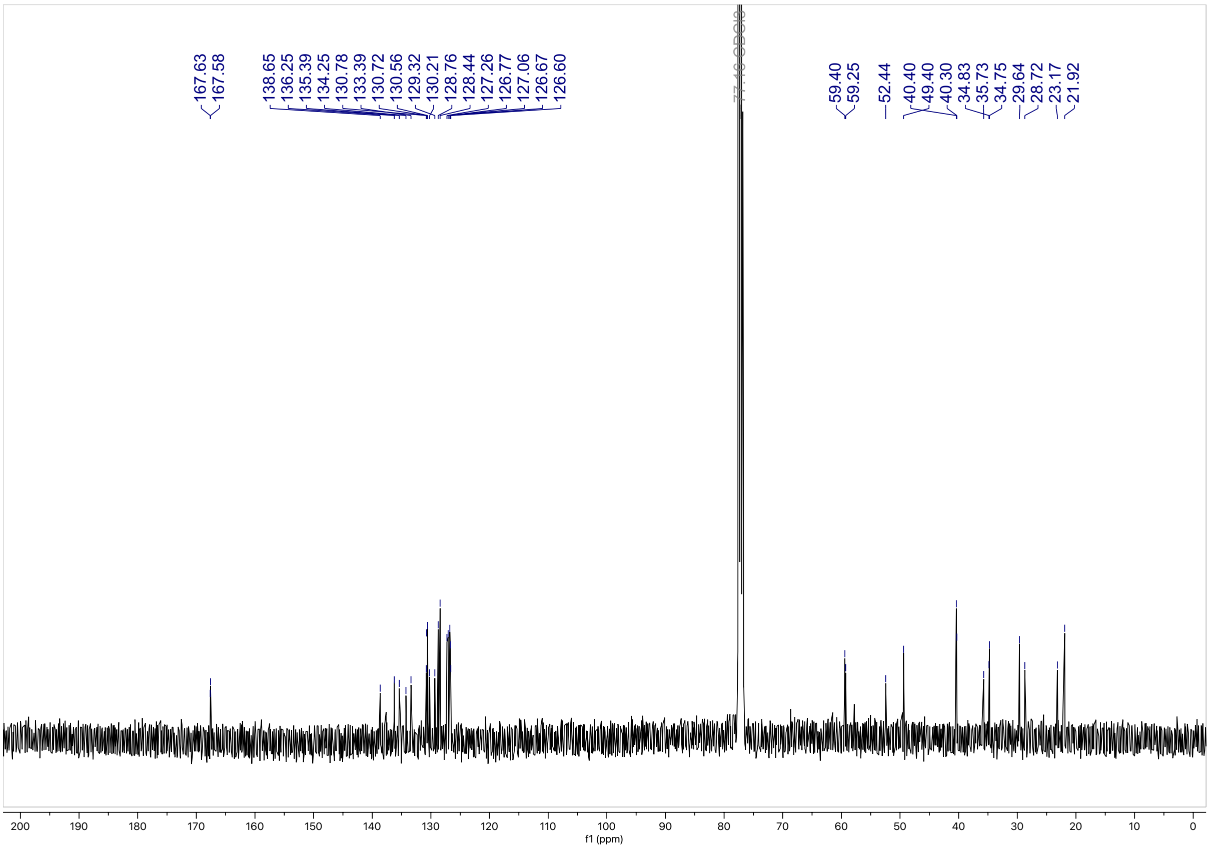

4 – ^{1}H NMR (400 MHz, CDCl$_3$)

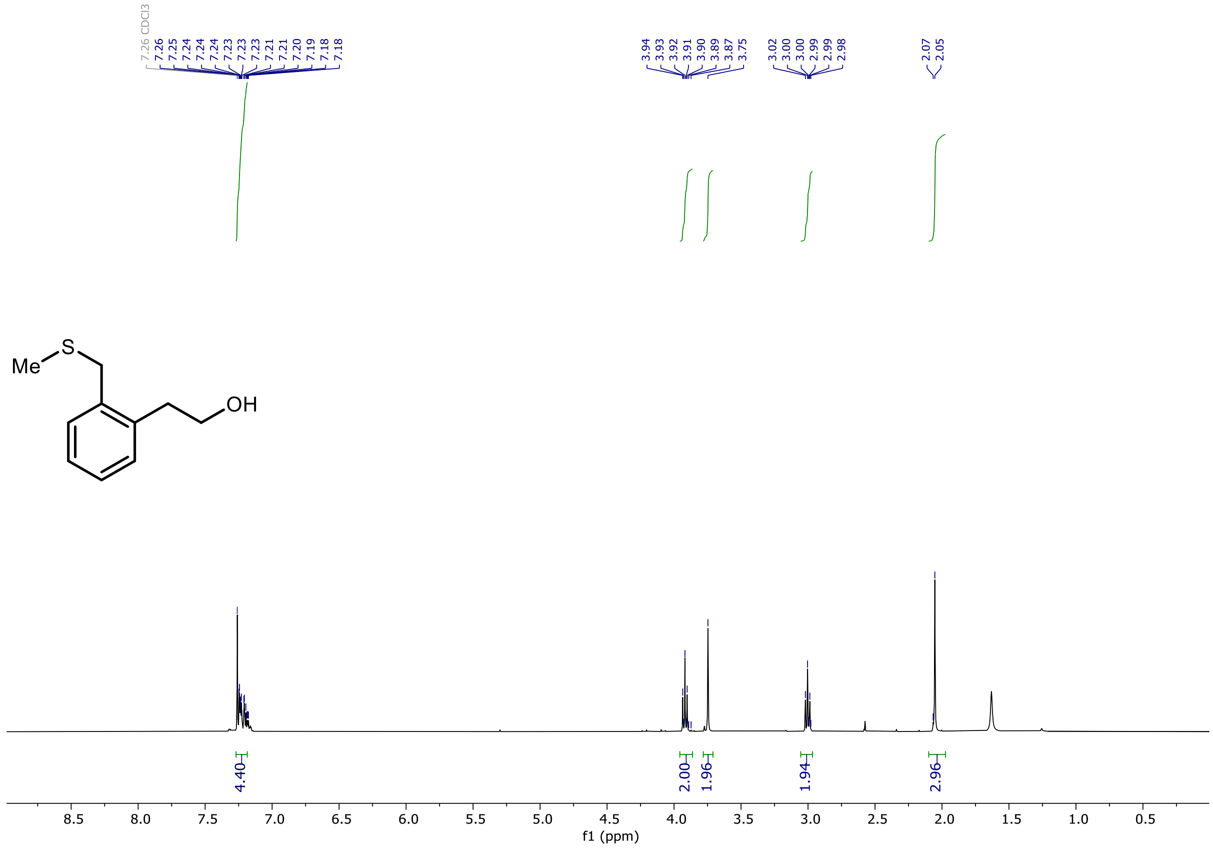

4 – ^{13}C NMR (101 MHz, CDCl$_3$)

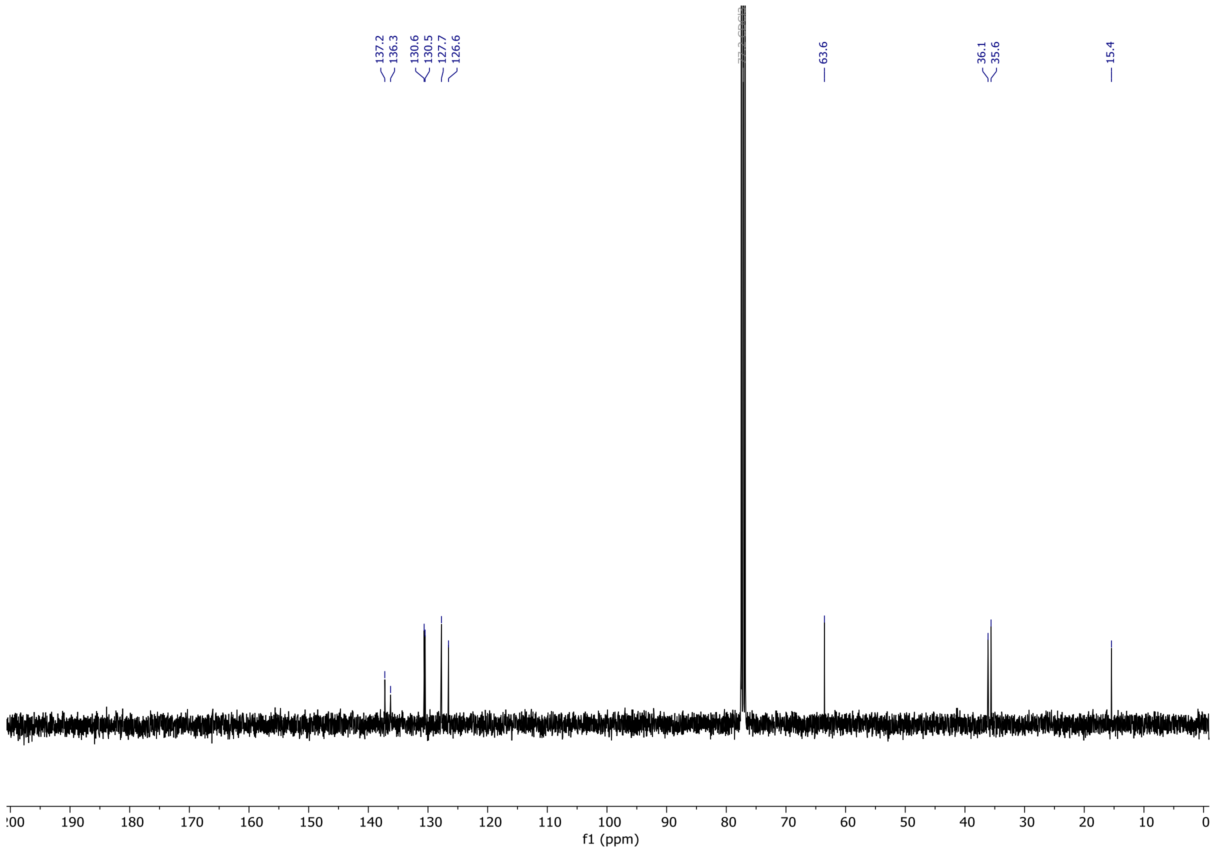

5 – ^{1}H NMR (400 MHz, $CDCl_3$)

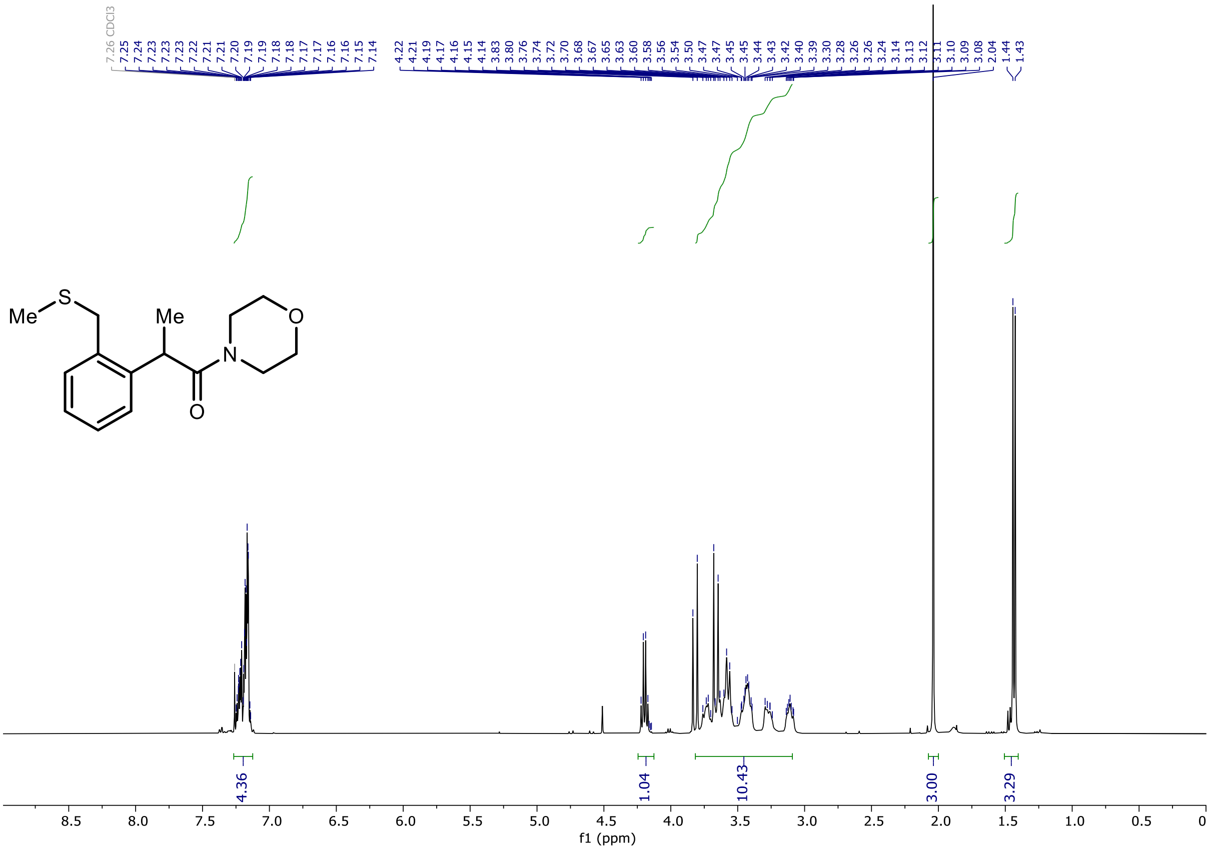

5 – ^{13}C NMR (101 MHz, $CDCl_3$)

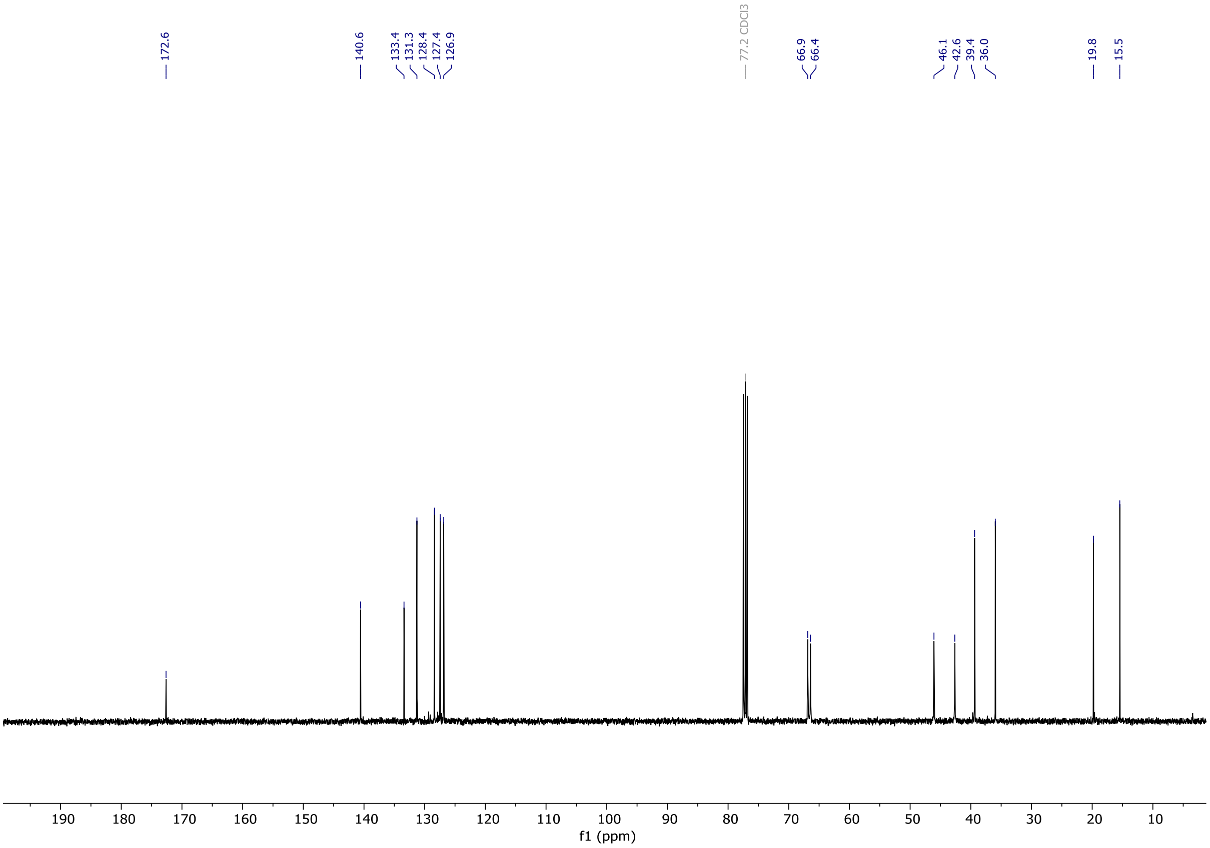

6 – ¹H NMR (400 MHz, CD_3CN)

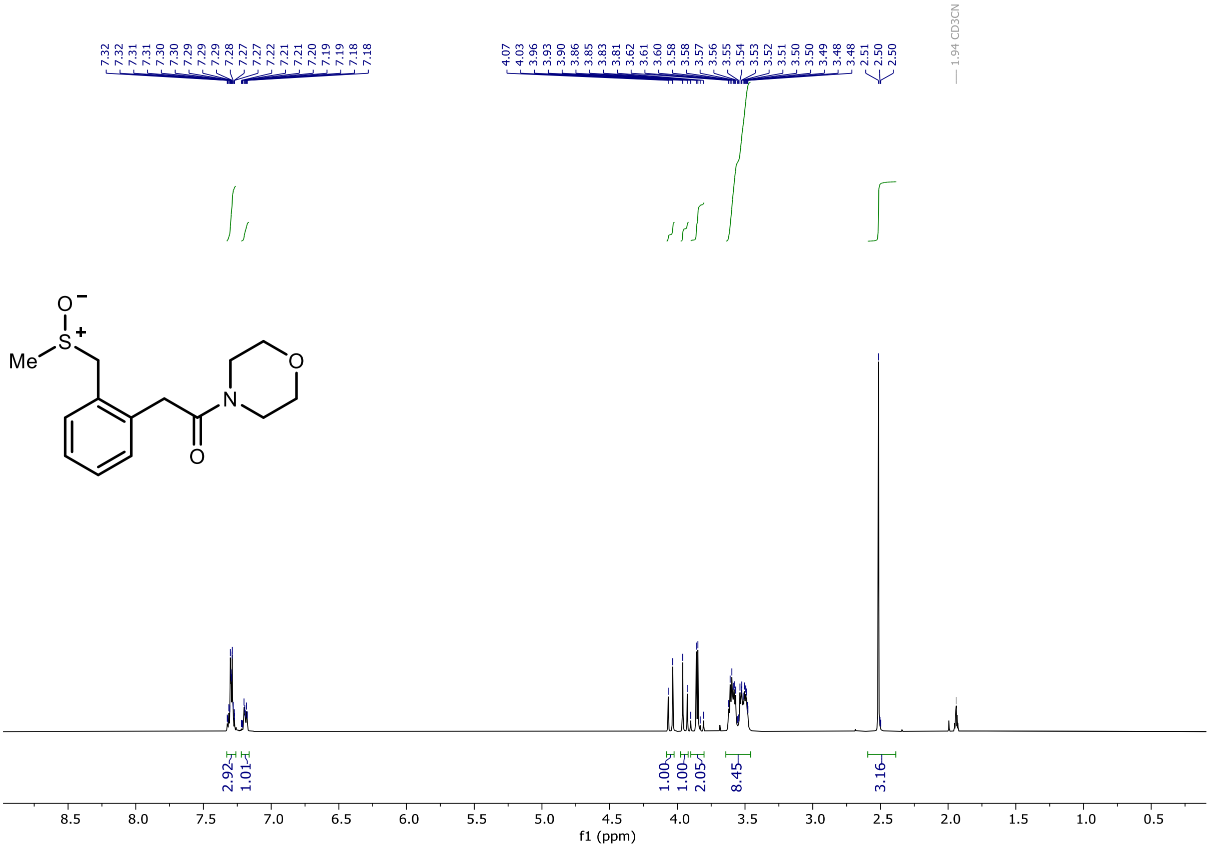

6 – ¹³C NMR (101 MHz, CD_3CN)

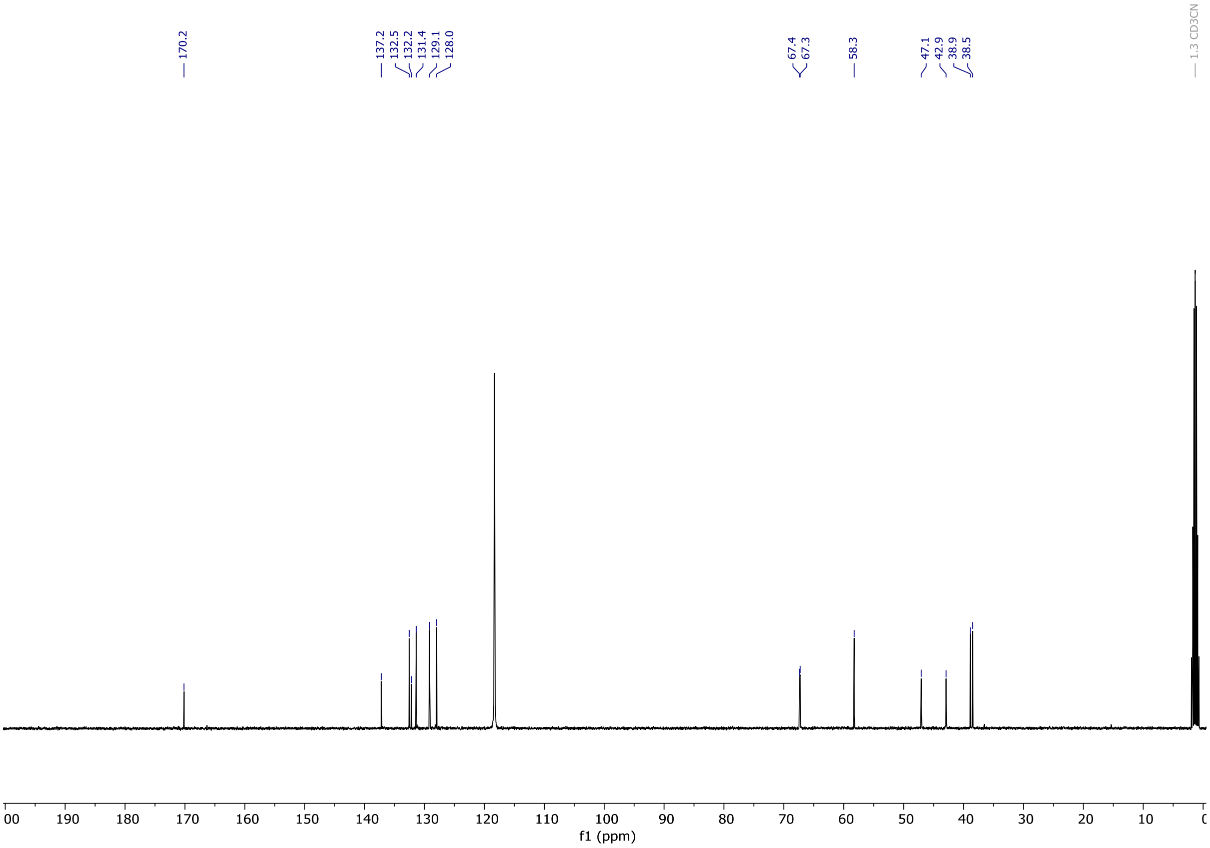

7 – ^{1}H NMR (400 MHz, $CDCl_3$, mixture of rotamers)

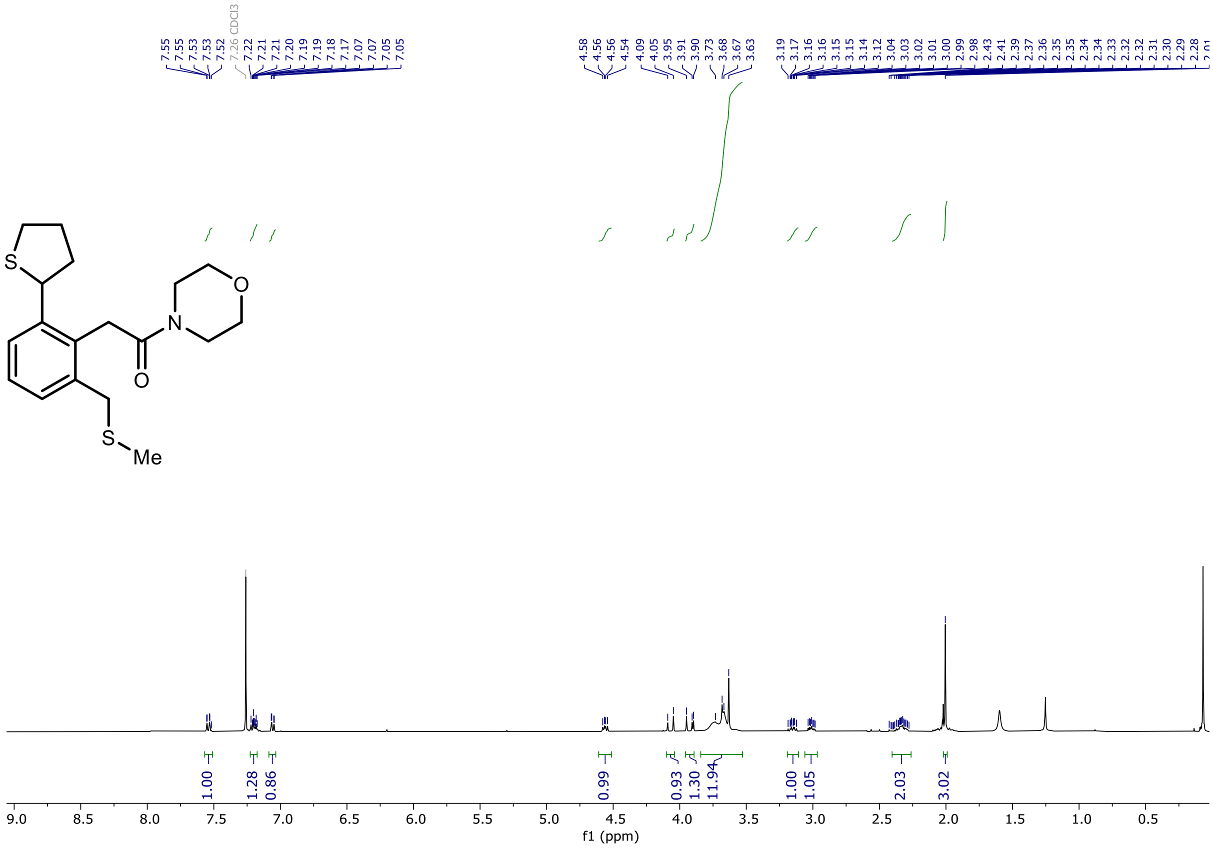

7 – ^{13}C NMR (101 MHz, $CDCl_3$, mixture of rotamers)

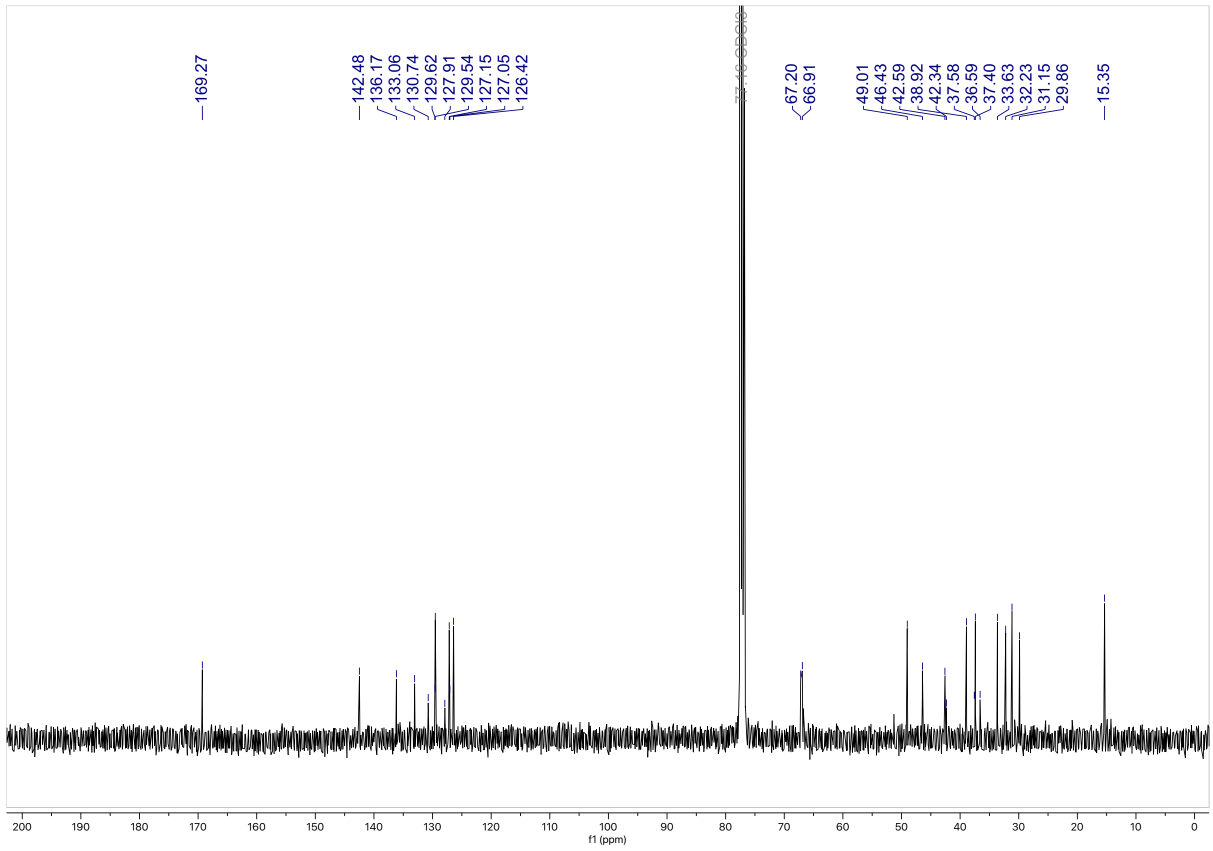

Supplement: Supplementary file 1 — Supporting Information [file ANIE-64-e202511703-s001.pdf]
